# NHC‐Au‐Catalyzed Isomerization of Propargylic B(MIDA)s to Allenes and Double Isomerization of Alkynes to 1,3‐Dienes

**DOI:** 10.1002/advs.202308710

**Published:** 2024-03-13

**Authors:** Li‐Cai Liu, Shuang Lin, Kangwei Xu, Jiasheng Qian, Ruibo Wu, Qingjiang Li, Honggen Wang

**Affiliations:** ^1^ Guangdong Provincial Key Laboratory of Chiral Molecule and Drug Discovery School of Pharmaceutical Sciences Sun Yat‐Sen University Guangzhou 510006 China

**Keywords:** 1,3‐diene, alkyne, allene, isomerization, NHC‐Au

## Abstract

The synthesis of allenyl boronates is an important yet challenging topic in organic synthesis. Reported herein is an NHC‐gold‐catalyzed 1,3‐H shift toward allenyl boronates synthesis from simple propargylic B(MIDA)s. Mechanistic studies suggest dual roles of the boryl moiety in the reaction: to activate the substrate for isomerization and at the same time, to prevent the allene product from further isomerization. These effects should be a result of α‐anion stabilization and α‐cation destabilization conferred by the B(MIDA) moiety, respectively. The NHC‐Au catalyst, which is commercially available, is also found to be reactive in alkyne‐to‐1,3‐diene isomerization reactions in an atom‐economic and base‐free manner.

## Graphical abstract

An NHC‐gold‐catalyzed 1,3‐H shift toward allenyl boronates synthesis from simple propargylic B(MIDA)s is reported. Mechanistic studies reveal dual roles of the boryl moiety in the reaction: to activate the substrate for isomerization and to prevent the allene product from further isomerization. These effects should be a result of α‐anion stabilization and α‐cation destabilization conferred by the B(MIDA) moiety, respectively.

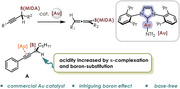

## 1 Introduction

**Scheme 1:**
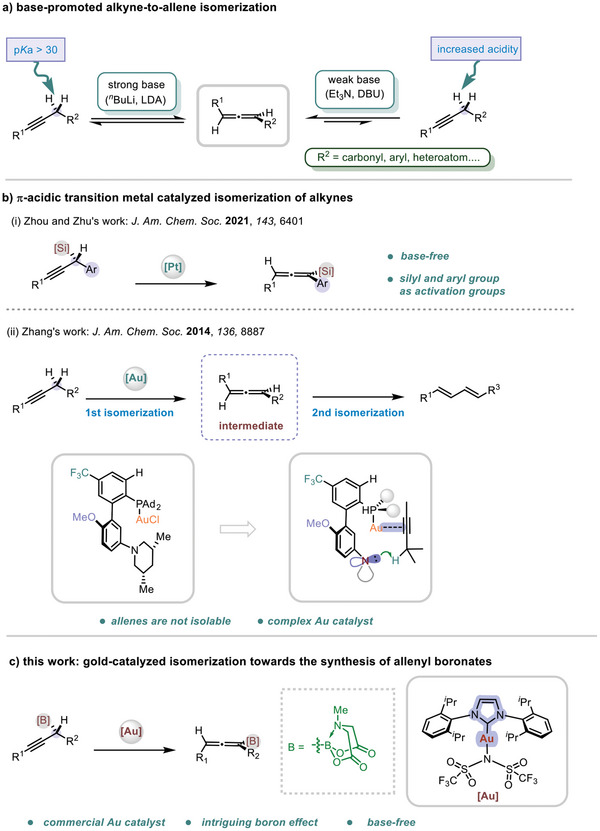
Synthesis of allenes via isomerization reaction of alkynes.

Allenes are among the most useful building blocks thanks to the fruitful chemistry of the unique cumulated double bonds.^[^ ^1^ ^]^ Not unexpectedly, the synthesis of allenes has been an actively explored research topic in modern organic chemistry.^[^ ^2^ ^]^ Among the numerous well‐developed methodologies, the isomerization of alkynes via a formal 1,3‐H shift represents the most straightforward and green one.^[^ ^3^ ^]^ Classically, strong bases such bases such *n*‐butyl lithium and alkali metal amide are needed to promote this process due to the weak acidity of the C─H bond (*pKa* in DMSO >30) at the propargylic position (**Scheme** **1**a, left).^[^ ^4^ ^]^ With proper activation, weaker bases are also feasible to facilitate the isomerization (Scheme 1a, right). So far, activation groups, including carbonyl group,^[^ ^5^ ^]^ alkene,^[^ ^6^ ^]^ alkyne,^[^ ^7^ ^]^ arene,^[^ ^8^ ^]^ and some heteroatom (oxygen,^[^ ^9^ ^]^ nitrogen,^[^ ^10^ ^]^ sulfur,^[^ ^11^ ^]^ and phosphorus),^[^ ^12^ ^]^ centered groups are reported. Such groups could have two benefits for the reaction: to enhance the acidity of the adjacent C─H bond, thereby lowering the kinetic barrier for deprotonation, and to provide a thermodynamic driving force for the formation of more stable allene as compared to the parent alkyne via conjugation. Alternatively, π‐acidic transition metal catalysts could also play a key role in alkyne‐to‐allene isomerization. For instance, Zhou and Zhu developed an elegant platinum‐catalyzed isomerization of propargylic silane toward the synthesis of allenyl silane under surprisingly base‐free conditions.^[^ ^13^ ^]^ Both the silyl and aryl groups serve as the activation groups for this transformation (Scheme 1b). By using a gold catalyst in combination with an amino‐decorated phosphine ligand, Zhang was able to develop several isomerization reactions of alkynes.^[^ ^14^ ^]^ However, allenes are not isolable under these conditions as they are prone to undergo further isomerization to 1,3‐dienes. Herein, we uncover that a boryl moiety alone could exert an intriguing effect on alkyne to allene isomerization reaction (Scheme 1d). This effect allows us to accomplish a simple NHC‐ligated cationic gold‐catalyzed isomerization of B(MIDA)‐substituted alkynes toward the synthesis of allenylic boronates under base‐free conditions. In particular, we showcase that the B(MIDA)‐substitution also prevents the undesired isomerization to 1,3‐dienes.

Allenyl boronates, with a boryl moiety attached to a cumulative system, are important synthons in a wide variety of transformations. Typical methods toward their synthesis include: the reaction of allenylmetallic reagents with trialkyl borate,^[^ ^15^ ^]^ metal‐catalyzed hydroboration of enynes,^[^ ^16^ ^]^ borylative displacement of propargylic electrophiles,^[^ ^17^ ^]^ and more recently the cross‐metathesis of allenes.^[^ ^18^ ^]^ Still, a greener method featuring a high atom economy under simple and mild reaction conditions is highly desirable. Method featuring a formal 1,3‐H shift was only possible for unsubstituted propargylic Bpin in the presence of strong bases such as Grignard reagent or KO*^t^* Bu.^[^ ^19^ ^]^

## 2 Results and Discussion

Our original goal was to develop a gold‐carbenoid^[^ ^20^ ^]^‐initiated boryl‐migrative rearrangement^[^ ^21^ ^]^ via a gold‐catalyzed oxidation of propargylic B(MIDA)s with *N*‐oxide. Out of our expectation, by using cationic IPrAuNTf_2_ (10 mol%) as a catalyst in the presence of 3,5‐dichloropyridine 1‐oxide in DMF at 100 °C, the anticipated borylated α,ß‐unsaturated ketone was only obtained in trace amount. Instead, the redox‐neutral 1,3‐H shift product **1** was isolated predominately (74% yield, entry 1, **Table** **1**). Realizing the later process is mechanistically interesting and synthetically useful, we then turned our attention to optimizing the synthesis of allenyl boronate **1**. We considered the pyridine *N*‐oxide must play the role of base as the reaction is essentially redox‐neutral. Thus, when replacing it with the 2,6‐dibromopyridine, a slightly increased yield was obtained (entry 2). However, control experiments showed that base is actually not required for the transformation. Heating **S1** with a catalytic amount of IPrAuNTf_2_ (10 mol%) in DMF at 100 °C delivered the product in a comparative yield, highlighting the simplicity of the conditions (entry 3). Further screening of solvents demonstrated nonpolar solvents gave inferior results (entries 7–9), with the original DMF being the best, implying a concerted pathway might be excluded. Control experiments concluded that IPrAuNTf_2_ was necessary for the transformation. No reaction occurred when it was omitted from the reaction (entry 10). Replacement of IPrAuNTf_2_ with IPrAuCl (entry 11) or Tf_2_NH (entry 12) led to no reaction as well, and finally, using a platinum catalytic system which was effective in the Zhou's isomerization of propargylic silane, no product could be detected (entries 13 and 14).

**Table 1:** Reaction optimization.

Entry^a)^	Catalyst	Solvent	Time [h]	Yield^b)^ [%]
1^c)^	IPrAuNTf_2_ (10%)	DMF	12	74^d)^
2^e)^	IPrAuNTf_2_ (10%)	DMF	2	81^d)^
3	IPrAuNTf_2_ (10%)	DMF	1	80^d)^
4	IPrAuNTf_2_ (10%)	DMSO	2	45
5	IPrAuNTf_2_ (10%)	Acetone	2	67
6	IPrAuNTf_2_ (10%)	DCM	2	70
7	IPrAuNTf_2_ (10%)	THF	1	NR
8	IPrAuNTf_2_ (10%)	Toluene	1	NR
9	IPrAuNTf_2_ (10%)	1,4‐dioxane	1	NR
10	–	DMF	1	NR
11	IPrAuCl (10%)	DMF	1	NR
12	Tf2NH (10%)	DMF	1	NR
13^f)^	Pt(PPh_3_)_4_ (5%)	Toluene	1	NR
14^f)^	Pt(PPh_3_)_4_ (5%)	DMF	1	NR

^a)^ General reaction conditions: **S1** (0.1 mmol, 1.0 equiv), catalyst (0.005–0.01 mmol, 5–10 mol%), solvent (1 mL, 0.1 m), 100 °C, 1–12 h;

^b)^ ^1^H NMR yield;

^c)^ 3,5‐dichloropyridine 1‐oxide (0.1 mmol, 1 equiv) was added;

^d)^ Isolated yield;

^e)^ 2,6‐dibromopyridine (0.02 mmol, 20 mol%) was added;

^f)^ Reaction under air conditions. NR = no reaction.

**Scheme 2:**
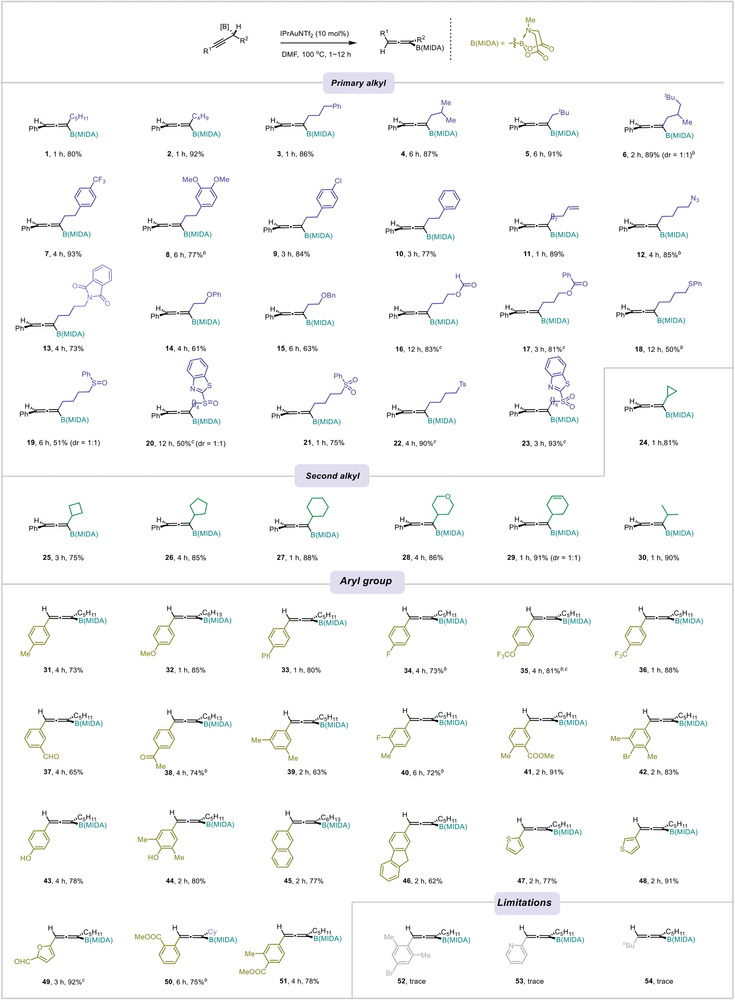
Substrate scope. a) General reaction conditions: propargylic B(MIDA) (0.1 mmol, 1 equiv), IPrAuNTf_2_ (0.01 mmol, 10 mol%), DMF (1 mL, 0.1 m), 100 °C, ≈2–12 h. b) Double catalyst (another 10 mol% IPrAuNTf_2_ was added after 2 h). c) 2,6‐Dibromopyridine (0.02 mmol, 20 mol%) was needed to facilitate the reaction.

The scope of the reaction was then explored (**Scheme** **2**). It was found a wide variety of primary alkyl substituents to the boryl moiety were compatible with the reaction. Many valuable functional groups, such as aryl (**7–10**), alkenyl (**11**), azido (**12**), imide (**13**), ether (**14 and 15**), ester (**16, 17**), and sulfonyl (**19–23**), were well tolerated. Of note, thioether (**18**), sulfoxide (**19, 20**), and *N*‐heteroaromatics (**20, 23**), which are potentially poisonous to the metal catalyst, survived as well, although lowered yields were observed. Secondary alkyl groups, which are sterically more hindered, were also compatible. As such, it was found both cyclic ones and acyclic ones gave the corresponding products in generally high yields (**24–30**). Substrates bearing different aryl groups, regardless of the electronic properties, were applicable as well. The formyl (**37**), acetyl (**38**), ester (**41, 50, 51**), and bromo (**42**) groups provided ample room for further derivatization. Phenols can be used directly without the necessitate for protection. Heteroaromatics, such as thiophene (**47, 48**) and furan (**49**) and fused rings (**45, 46**), underwent reaction without difficulty. However, the use of 2,6‐disubstituted phenyl (**52**), and pyridine (**53**) shut down the reactivity completely. Replacing the aryl group with an alkyl group turned out to be unfruitful either (**54**). Of note, the addition of pyridine base (**16, 17, 20, 22, 23, 35, 49**) or doubling the catalyst loading (**6, 8, 12, 18, 34, 35, 38, 40, 50**) facilitated full conversion for those substrates that are poisonous to the metal catalyst.

**Scheme 3:**
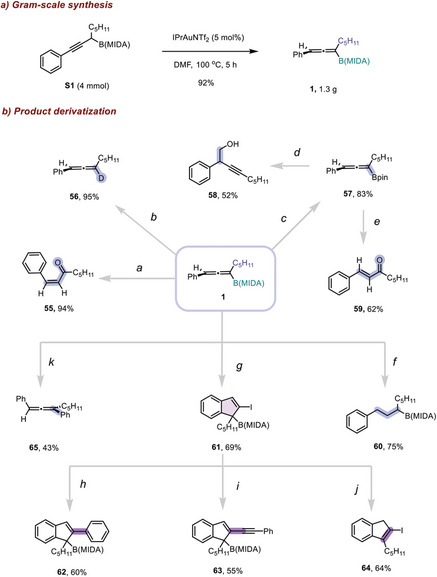
Gram‐scale synthesis and product derivatizations. Reaction conditions: a) NaOH, H_2_O_2_, THF, rt, 1 h. b) Cs_2_CO_3_, D_2_O, dioxane, 80 °C, 5 h. c) Pinnacol, H_2_SO_4_, THF, 60 °C, 5 h. d) Paraformaldehyde, *rac*‐CPA, toluene, rt, 2.5 h. e) Py·HBr_3_, H_2_O/HFIP (1:1), 70 °C, overnight. f) Pd/C, H_2_, MeOH, rt, 0.5 h. g) DIH, DCM, rt, 0.5 h. h) Phenylboronic acid, Pd(PPh_3_)_2_Cl_2_, sphos, K_2_CO_3_, Ag_2_O, THF, 60 °C, 2 h. i) Phenylacetylene, Pd(PPh_3_)_2_Cl_2_, CuI, Et_3_N, DMF, 60 °C, overnight. j) Sodium thiophenol, Cs_2_CO_3_, dioxane, H_2_O, 100 °C, 0.5 h. k) PhI, Pd(PPh_3_)_2_Cl_2_, Na_2_CO_3_, MeOH/Tol (4:1), rt, 12 h.

Derivatizations of the formed allenyl boronates were then conducted (**Scheme** **3**). Interestingly, by using a 5 mol% catalyst, **1** could be synthesized on a gram scale and in a higher yield of 92% (Scheme 3a). Chemoselective oxidation of the C─B bond in **1** led to the selective formation of *cis* a,ß‐unsaturated ketone **55**. Base promoted‐protodeborylaion in D_2_O gave a deuterated allene **56** in excellent yield. Ligand exchange with pinacol in the presence of H_2_SO_4_ furnished a sp^2^‐B allenyl boronate **57** without difficulty. A *rac*‐CPA‐catalyzed propargylation reaction of **57** with paraformaldehyde gave a propargyl methanol product (**58**) in good efficiency. Treatment of **57** with Py·HBr_3_ gave *trans*‐**59** in a moderate yield. Upon catalytic hydrogenation with H_2_, a secondary alkyl boronates **60** was obtained. Treatment of **1** with DIH, an intramolecular cyclization occurred to give an indene product **61** bearing a boryl and an iodo functional group. Transformations of the iodo moiety with Pd‐catalyzed Suzuki–Miyaura (**62**) or Sonagashira cross‐coupling (**63**) reaction were successful. Protodeborylaion with sodium thiophenol led to a double bond migrated indene product **64**. In another vein, **1** could undergo a Suzuki–Miyaura coupling reaction to give a tri‐substituted allene **65** in moderate yield.

Efforts were then devoted to elucidating the reaction mechanism. In Zhang's gold‐catalyzed isomerization reaction of alkynes, an internal base decorated with the phosphine ligand was important to facilitate the proton transform,^[^ ^14^ ^]^ and except for terminal alkynes, the allene products were not isolable as they underwent even faster isomerization to 1,3‐dienes. We, therefore, raise two questions: what is responsible for the base‐free catalytic system, the B(MIDA)‐substitution or a different NHC‐ligated Au catalyst? And why does no further isomerization of allenyl boronates occur?

**Scheme 4:**
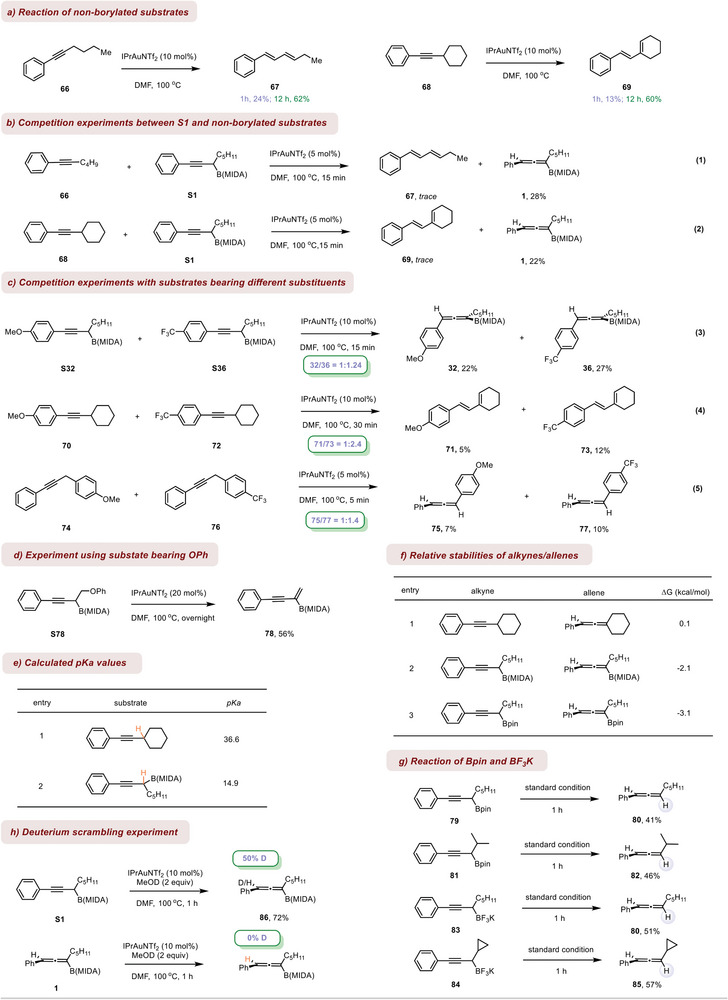
Mechanism studies.

We first tried a reaction using non‐borylated substrates with our standard gold‐catalytic system. It was found the isomerizations did occur but with low conversions (**Scheme** **4**a). Similar to Zhang's observation, it was 1,3‐diene, not the allene product that was isolated. These results suggested dual roles of the boryl moiety in our reaction: to activate the substrate for isomerization and at the same time, to stabilize the allene product to avoid further reaction. Indeed, competition experiments between the borylated and non‐borylated substrates conformed a drastic activation effect of boron (Scheme 4b). It should be noted that by prolonging the reaction time to 12 h, both secondary and primary alkyl‐substituted alkynes underwent smooth isomerization to provide the 1,3‐diene products with good yields (Scheme 4a). These results suggested that the NHC‐ligated cationic Au complex could serve as a quite alternative catalyst for alkyne isomerization to Zhang's catalyst.

Previous studies from others and us have unveiled that the B(MIDA) moiety could exert interesting reactivity to the substrate by the ß‐boron effect,^[^ ^21^, ^22^ ^]^ or by α‐anion stabilization.^[^ ^23^ ^]^ Thus, in our case, the (C─B) bond could potentially hyperconjugate with the triple bond (electron‐donating), making the latter more nucleophilic toward π‐complexation with the metal. Or, the B(MIDA)‐substitution at the propargylic position could increase the acidity of the adjacent C─H bond (electron‐withdrawing), thereby facilitating a hydrogen shift. To distinguish the two potential roles, several competition experiments were conducted. It was shown that substrates bearing electron‐withdrawing CF_3_ reacted faster than the electron‐donating OMe ones, regardless of whether there is a boryl substituent at the propargylic position (Scheme 4c). The same trend was seen for substrates bearing different aryl groups at the propargylic position, wherein the acidity of the propargylic C─H bond correlates with the electronic property of the aryl ring (eq 5). The above results should suggest the acidity of the C─H bond is crucial for reactivity and the role of B(MIDA) might be to stabilize its adjacent carbon anion thereby increasing the acidity of the corresponding C─H bond. This acidity was reflected in the reaction of substrate **S78** bearing a relatively poor leaving group (OPh). The elimination reaction dominated the anticipated hydrogen shift process (Scheme 4d). Indeed, in accordance with our expectation, DFT calculations suggest the B(MIDA)‐substitution leads to a significantly increased acidity of the adjacent C─H bond (Scheme 4e). The reluctancy of B(MIDA)‐substituted allene to undergo further isomerization could be due to its higher thermo‐stability as compared to alkyl‐substituted one (Scheme 4f). Our calculation also suggests the propargylic Bpin should favor the allene product. But under our catalytic system, this substrate, along with BF_3_K congeners, only gave the deborylated allenes product (Scheme 4g), which is an indication that the protected B(MIDA) is important for success. The deuterium scrambling experiment showed that the proton on allene stemmed from the solvent, possibly excluding a concerted reaction pathway (Scheme 4h).

**Scheme 5:**
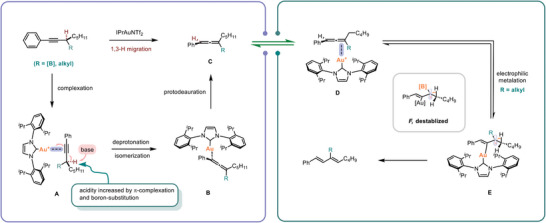
Proposed mechanism.

Based on the above experimental results and literature precedents,^[^ ^14a^ ^]^ a reaction mechanism was proposed as shown in **Scheme** **5**. Initially, the π‐acidic cationic gold catalyst coordinates with the triple bond. This complexation leads to a lowering of π* and thereby increases the acidity of the propargylic C─H bond. The presence of B(MIDA) also contributes to the increase of acidity due to the hemi‐labile nature of the MIDA ligand (R = B(MIDA).^[^ ^21e^ ^]^ Subsequently, upon deprotonation, an allenylgold intermediate **B** is formed. The *ipso*‐protodeauration then delivers the allenyl boronate product **C**. For substrates free of B(MIDA) substitution, an electrophilic metalation gives an allylic carbocation **E**, which undergoes deprotonation and follow‐up *ipso*‐protodeauration to form the 1,3‐diene product. For propargylic B(MIDA), however, the allenyl B(MIDA) would be reluctant to undergo electrophilic metalation as this would result in the formation of a carbon cation **F** that can be destabilized by the α‐boron moiety.

## 3 Conclusion

In summary, a gold‐catalyzed alkyne‐to‐allene isomerization reaction is developed. The protocol provides atom‐economic and base‐free access to a wide variety of allenyl boronates from simple propargylic B(MIDA)s. A unique boron effect was observed as the boron moiety not only activates the substrates, presumably by stabilizing its α‐anion, but also prevents further isomerization to 1,3‐dienes due to α‐cation destabilization. We also showcase that the NHC‐Au catalytic system is powerful in an alkyne‐to‐1,3‐diene isomerization reaction, providing a simple alternative to Zhang's phosphine‐Au method.

## Conflict of Interest

The authors declare no conflict of interest.

## Supporting information

Supporting Information

## Data Availability

The data that support the findings of this study are available in the supplementary material of this article.
