# Supplementary material for: NHC‐Au‐Catalyzed Isomerization of Propargylic B(MIDA)s to Allenes and Double Isomerization of Alkynes to 1,3‐Dienes
Source: Adv Sci (Weinh). 2024 Mar 13;11(21):2308710. doi: 10.1002/advs.202308710 (PMC11151053; doi:10.1002/advs.202308710)

ADVANCED SCIENCE
Open Access

Supporting Information

for *Adv. Sci.*, DOI 10.1002/advs.202308710

NHC-Au-Catalyzed Isomerization of Propargylic B(MIDA)s to Allenes and Double Isomerization of Alkynes to 1,3-Dienes

*Li-Cai Liu, Shuang Lin, Kangwei Xu, Jiasheng Qian, Ruibo Wu, Qingjiang Li and Honggen Wang**

Supporting Information

NHC-Au-Catalyzed Isomerization of Propargylic B(MIDA)s to Allenes and Double Isomerization of Alkynes to 1,3-Dienes

Li-Cai Liu[a], Shuang Lin[a], Kangwei Xu[a], Jiasheng Qian[a], Ruibo Wu[a], Qingjiang Li[a], Honggen Wang*[a]

[a]Guangdong Provincial Key Laboratory of Chiral Molecule and Drug Discovery, School of Pharmaceutical Sciences, Sun Yat-Sen University, Guangzhou 510006, China

Table of Contents

1. General information………………………………………………………………...3

2. Preparation of the starting materials…………………………………………….4

3. General procedure for the synthesis of allenyl boranes……………………34

4. Derivatization of the product **1**……………………………………….……..60

5. Mechanism study………………………………………………………........69

6. DFT studies…………………………………………………………………….. 77

7. NMR spectrum…………………………………………………………………..80

1. General information

THF was dried over Na with benzophenone-ketyl intermediate as indicator; For chromatography, 200-300 mesh silica gel (Qingdao, China) was employed. NMR–spectra were recorded on Bruker Avance III–400M and AscendTM 500M in solvents as indicate. Chemical shifts are reported in ppm using tetramethylsilane as internal standard when using $CDCl_3$, $(CD_3)_2CO$, $(CD_3)_2SO$ CD_3CN as the solvent, and coupling constants (*J*) were in Hertz (Hz). Multiplicities are reported using the following abbreviations: s = singlet, d = doublet, t = triplet, q = quartet, m = multiplet. The carbon directly attached to the boron atom was not detected by ^{13}C NMR, likely due to quadropolar relaxation. High–resolution mass spectra (HRMS) were recorded on a Bruker VPEXII spectrometer with ESI mode unless otherwise stated. Unless otherwise noted, all commercially available materials were used without further purification. PE: petroleum ether; EA: ethyl acetate; DMF: *N, N*-dimethylformamide; THF: tetrahydrofuran, DMSO: dimethyl sulfoxide.

2. Preparation of the starting materials

General procedure A: Propargylic Boronates **S1-S17, S24-S30** and **S78** were prepared according to the known method reported by Tao Xu and our group. [22c, 24]

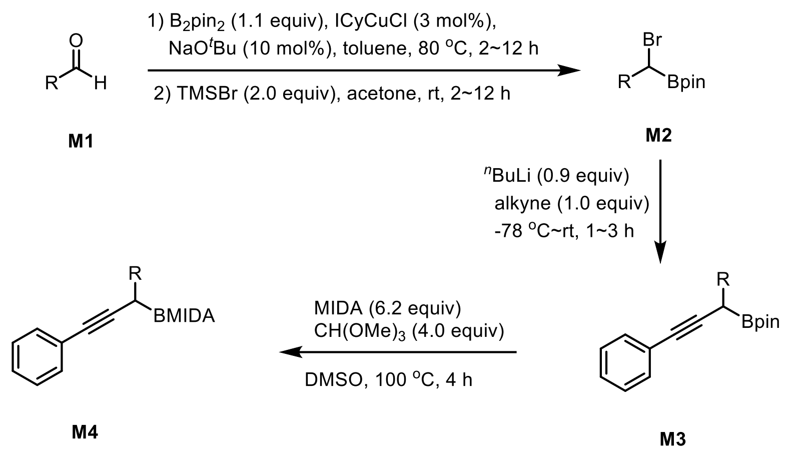

In a glove box, a 25 mL Schleck tube equipped with a stirrer bar was charged with B_2Pin_2 (22.0 mmol, 22.0 equiv, 5.59 g), NaO^{t}Bu (2.0 mmol, 10.0 mol%, 0.192 g), ICyCuCl (0.6 mmol, 3.0 mol%, 0.193 g) and **M1** (20.0 mmol, 1.0 equiv), toluene (40 mL). The tube was sealed and heated at 80 °C with stirring until total consumption of the starting material, as monitored by GC-MS. The reaction solution was cooled to room temperature, and the solvent was concentrated in vacuo. The crude mixture was dissolved with acetone (40 mL). TMSBr (40.0 mmol, 2.0 equiv, 6.12 g) was added to the solution. After stirring for 12 h at room temperature, the solvent was concentrated in vacuo. The crude material was purified by flash column chromatography (SiO_2, PE/EA) or by reduced pressure distillation to give the product **M2**. Note: generally, the flash column chromatography should be done fast and is better to finish within 30 min; otherwise, the product could be decomposed to give a lower yield.

To a stirred solution of phenylacetylene (10.0 mmol, 1.0 equiv, 1.02 g) and THF (20 mL), *n*-butyl lithium in hexane (2.5 M, 9.0 mmol, 0.9 equiv) was added dropwise at −78 °C. The resulting solution was stirred at the same temperature for 1 h, and then α-bromo boronic esters **M2** (10.0 mmol, 1.0 equiv) was added dropwise and the mixture was stirred at room temperature for 2 h. After the reaction, saturated NH_4Cl (aq) was added to the reaction mixture at 0 °C and the solution was stirred for 20 min. The reaction mixture was mixed with water and the product was extracted repeatedly with DCM. The organic phase was separated and the aqueous layer was extracted with DCM (30 mL) for three times. The combined organic

layer was dried over anhydrous Na_2SO_4 and concentrated under reduced pressure to give the product **M3**.

Anhydrous DMSO (40 mL) was added to dissolve the solid (or viscous oil) **M3** which was then added via syringe to a suspension of *N*-methyliminodiacetic acid (MIDA, 62.0 mmol, 6.2 equiv, 9.11 g) and $CH(OMe)_3$ (40.0 mmol, 4.0 equiv, 4.24 g) in DMSO. The resulting mixture was stirred at 100 °C until the propargyl boronic esters was used up by GC-MS monitoring. After cooling to room temperature, the reaction mixture was diluted with ethyl acetate (30 mL) and water (50 mL). The organic phase was separated and the aqueous layer was extracted with ethyl acetate (30 mL) for three times. The combined organic layer was dried over anhydrous Na_2SO_4 and concentrated under reduced pressure. The resulting crude product was purified by flash chromatography on silica gel with an appropriate solvent as eluent to afford the pure product **M4**.

6-methyl-2-(1-phenyloct-1-yn-3-yl)-1,3,6,2-dioxazaborocane-4,8-dione

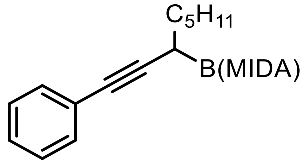

S1

The product **S1** was obtained in 23% (1.56 g) overall yield as a yellow solid after column chromatography (eluent = ethyl acetate/petroleum ether v/v 2:1).

^{1}H NMR (400 MHz, DMSO-*d*$_6$) δ 7.32 (s, 5H), 4.41 (d, *J* = 17.3 Hz, 1H), 4.27 (d, *J* = 16.8 Hz, 1H), 4.04 (d, *J* = 17.3 Hz, 1H), 3.91 (d, *J* = 17.0 Hz, 1H), 3.09 (s, 3H), 2.06 – 1.98 (m, 1H), 1.64 – 1.30 (m, 8H), 0.88 (t, *J* = 6.9 Hz, 3H).

^{13}C NMR (101 MHz, DMSO-*d*$_6$) δ 164.4, 163.7, 126.4, 123.9, 123.1, 118.9, 88.7, 76.7, 58.0, 57.7, 41.3, 26.5, 24.6, 23.6, 17.4, 9.3.

HRMS (ESI): calculated for $C_{19}H_{24}BNO_4$ $[M+Na]^+$, 364.1691; Found, 364.1687.

6-methyl-2-(1-phenylhept-1-yn-3-yl)-1,3,6,2-dioxazaborocane-4,8-dione

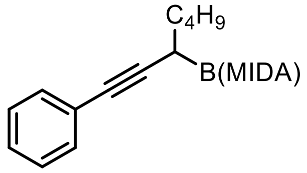

S2

The product **S2** was obtained in 11% (0.72 g) overall yield as a yellow solid after column chromatography

(eluent = ethyl acetate/petroleum ether v/v 2:1).

^{1}H NMR (400 MHz, DMSO-*d$_6$*) δ 7.32 (s, 5H), 4.42 (d, *J* = 17.3 Hz, 1H), 4.28 (d, *J* = 17.0 Hz, 1H), 4.05 (d, *J* = 17.3 Hz, 1H), 3.92 (d, *J* = 16.9 Hz, 1H), 3.09 (s, 3H), 2.03 (dd, *J* = 10.3, 3.3 Hz, 1H), 1.65 – 1.25 (m, 6H), 0.90 (t, *J* = 7.1 Hz, 3H).

^{13}C NMR NMR (101 MHz, DMSO-*d$_6$*) δ 168.9, 168.3, 131.1, 128.5, 127.7, 123.5, 93.3, 81.3, 62.6, 62.4, 45.9, 30.9, 29.0, 22.0, 14.0.

HRMS (ESI): calculated for $C_{18}H_{22}BNO_4$ [M+Na]$^+$, 350.1534; Found, 350.1538.

2-(1,6-diphenylhex-1-yn-3-yl)-6-methyl-1,3,6,2-dioxazaborocane-4,8-dione

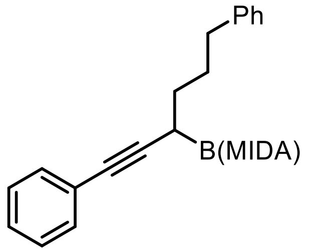

S3

The product **S3** was obtained in 28% (2.18 g) overall yield as a yellow solid after column chromatography (eluent = ethyl acetate/petroleum ether v/v 2:1).

^{1}H NMR (400 MHz, DMSO-*d$_6$*) δ 7.31 – 7.15 (m, 10H), 4.40 (d, *J* = 16.4 Hz, 1H), 4.26 (d, *J* = 17.9 Hz, 1H), 4.04 (d, *J* = 17.1 Hz, 1H), 3.91 (d, *J* = 17.1 Hz, 1H), 3.08 (s, 3H), 2.69 – 2.58 (m, 2H), 2.07 (dd, *J* = 10.3, 3.7 Hz, 1H), 1.76 – 1.44 (m, 4H).

^{13}C NMR (101 MHz, DMSO-*d$_6$*) δ 168.9, 168.3, 142.3, 131.1, 128.5, 128.3, 128.3, 127.7, 125.6, 123.5, 93.1, 81.5, 62.6, 62.4, 45.9, 35.0, 30.7, 28.9.

HRMS (ESI): calculated for $C_{23}H_{24}BNO_4$ [M+Na]$^+$, 412.1691; Found, 412.1689.

6-methyl-2-(5-methyl-1-phenylhex-1-yn-3-yl)-1,3,6,2-dioxazaborocane-4,8-dione

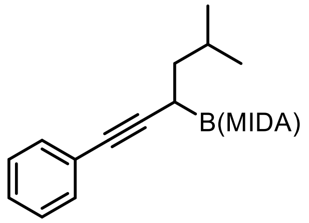

S4

The product **S4** was obtained in 18% (1.17 g) overall yield as a yellow solid after column chromatography (eluent = ethyl acetate/petroleum ether v/v 2:1).

^{1}H NMR (400 MHz, DMSO-*d$_6$*) δ 7.34 – 7.29 (m, 5H), 4.41 (d, *J* = 17.4 Hz, 1H), 4.27 (d, *J* = 16.9 Hz, 1H),

4.05 (d, *J* = 17.3 Hz, 1H), 3.92 (d, *J* = 16.9 Hz, 1H), 3.11 (s, 3H), 2.10 (dd, *J* = 11.7, 3.8 Hz, 1H), 1.94 – 1.84 (m, 1H), 1.56 – 1.49 (m, 1H), 1.31 – 1.23 (m, 1H), 0.96 (d, *J* = 6.7 Hz, 3H), 0.91 (d, *J* = 6.6 Hz, 3H).

^{13}C NMR (126 MHz, DMSO-*d$_6$*) δ 169.0, 168.3, 131.1, 128.6, 127.7, 123.5, 93.2, 81.2, 62.7, 62.5, 46.0, 38.3, 26.6, 23.7, 21.0.

HRMS (ESI): calculated for $C_{18}H_{22}BNO_4$ $[M+Na]^+$, 350.1534; Found, 350.1531.

2-(5,5-dimethyl-1-phenylhex-1-yn-3-yl)-6-methyl-1,3,6,2-dioxazaborocane-4,8-dione

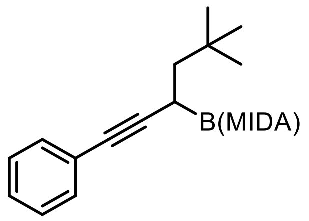

S5

The product **S5** was obtained in 32% (2.18 g) overall yield as a yellow solid after column chromatography (eluent = ethyl acetate/petroleum ether v/v 2:1).

^{1}H NMR (400 MHz, Chloroform-*d*) δ 7.40 – 7.34 (m, 1H), 7.23 – 7.17 (m, 4H), 4.10 (dq, *J* = 14.7, 5.9 Hz, 2H), 3.96 – 3.81 (m, 2H), 3.12 (dd, *J* = 8.3, 3.2 Hz, 3H), 1.97 (d, *J* = 10.2 Hz, 1H), 1.57 (dq, *J* = 9.5, 4.7, 4.1 Hz, 2H), 1.02 – 0.91 (m, 9H).

^{13}C NMR (101 MHz, DMSO-*d$_6$*) δ 168.9, 168.4, 130.8, 128.6, 127.6, 123.7, 95.1, 80.9, 62.9, 62.8, 45.9, 42.9, 31.7, 29.7.

HRMS (ESI): calculated for $C_{19}H_{24}BNO_4$ $[M+Na]^+$, 364.1691; Found, 364.1691.

6-methyl-2-(5,7,7-trimethyl-1-phenyloct-1-yn-3-yl)-1,3,6,2-dioxazaborocane-4,8-dione

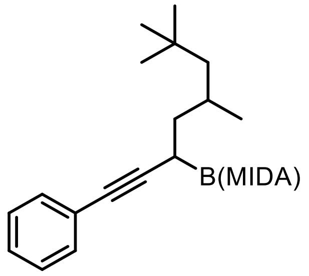

S6

The product **S6** was obtained in 16% (1.22 g) overall yield as a yellow solid after column chromatography (eluent = ethyl acetate/petroleum ether v/v 2:1), dr = 1:1.

^{1}H NMR (500 MHz, DMSO-*d$_6$*) δ 7.37 – 7.25 (m, 5H), 4.45 – 4.24 (m, 2H), 3.91 – 4.07 (m, 2H), 3.11 (s, 3H), 2.09 (dd, *J* = 11.4, 3.8 Hz, 1H), 1.93 – 1.79 (m, 1H), 1.45 – 1.11 (m, 4H), 1.02 – 0.91 (m, 12H).

^{13}C (126 MHz, DMSO-*d$_6$*) δ 169.0, 168.9, 168.3, 168.3, 131.0, 130.9, 128.6, 123.6, 93.6, 93.2, 81.3, 81.1,

62.7, 62.7, 62.5, 62.4, 51.5, 49.0, 46.0, 45.9, 31.2, 30.7, 30.1, 29.8, 28.0, 27.6, 24.1, 21.4.

HRMS (ESI): calculated for $C_{22}H_{30}BNO_4$ $[M+Na]^+$, 406.2160; Found, 406.2165.

6-methyl-2-(1-phenyl-5-(4-(trifluoromethyl)phenyl)pent-1-yn-3-yl)-1,3,6,2-dioxazaborocane-4,8-dione

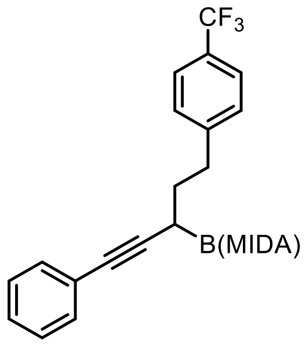

S7

The product **S7** was obtained in 18% (1.59 g) overall yield as a yellow solid after column chromatography (eluent = ethyl acetate/petroleum ether v/v 2:1).

^{1}H NMR (400 MHz, DMSO-*d$_6$*) δ 7.65 (d, *J* = 8.0 Hz, 2H), 7.49 (d, *J* = 7.8 Hz, 2H), 7.39 – 7.33 (m, 5H), 4.41 (d, *J* = 17.3 Hz, 1H), 4.29 (d, *J* = 16.9 Hz, 1H), 4.04 (d, *J* = 17.3 Hz, 1H), 3.95 (d, *J* = 17.0 Hz, 1H), 3.09 (s, 3H), 3.06 – 3.00 (m, 1H), 2.91 – 2.80 (m, 1H), 2.05 (dd, *J* = 10.8, 4.0 Hz, 1H), 1.92 – 1.80 (m, 2H).

^{13}C NMR (101 MHz, DMSO- *d$_6$*) δ 168.8, 168.3, 146.9, 131.2, 129.2, 128.5, 127.8, 126.6 (d, *J* = 31.7 Hz), 125.1 (t, *J* = 3.9 Hz), 123.4, 92.4, 82.1, 62.7, 62.4, 46.0, 34.4, 31.0.

HRMS (ESI): calculated for $C_{23}H_{21}BF_3NO_4$ $[M+Na]^+$, 466.1408; Found, 466.1407.

2-(5-(3,4-dimethoxyphenyl)-1-phenylpent-1-yn-3-yl)-6-methyl-1,3,6,2-dioxazaborocane-4,8-dione

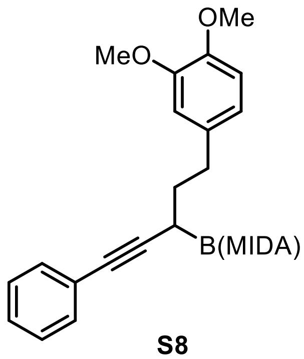

S8

The product **S8** was obtained in 16% (1.39 g) overall yield as a yellow solid after column chromatography (eluent = ethyl acetate/petroleum ether v/v 2:1).

^{1}H NMR (400 MHz, DMSO-*d$_6$*) δ 7.39 – 7.32 (m, 5H), 6.89 – 6.80 (m, 2H), 6.75 (dd, *J* = 8.1, 2.0 Hz, 1H),

4.41 (d, J = 17.4 Hz, 1H), 4.28 (d, J = 16.9 Hz, 1H), 4.04 (d, J = 17.3 Hz, 1H), 3.94 (d, J = 17.0 Hz, 1H), 3.74 (s, 3H), 3.71 (s, 3H), 3.08 (s, 3H), 2.92 – 2.84 (m, 1H), 2.72 – 2.59 (m, 1H), 2.03 (dd, J = 10.7, 4.1 Hz, 1H), 1.88 – 1.71 (m, 2H).

^{13}C NMR (126 MHz, Chloroform-*d*) δ 167.0, 166.5, 148.9, 147.2, 134.6, 131.6, 128.7, 128.2, 123.4, 120.6, 112.1, 111.3, 91.7, 83.3, 63.0, 63.0, 56.1, 56.0, 45.5, 34.7, 31.3.

HRMS (ESI): calculated for $C_{24}H_{26}BNO_6$ $[M+Na]^+$, 458.1745; Found, 458.1741.

2-(5-(4-chlorophenyl)-1-phenylpent-1-yn-3-yl)-6-methyl-1,3,6,2-dioxazaborocane-4,8-dione

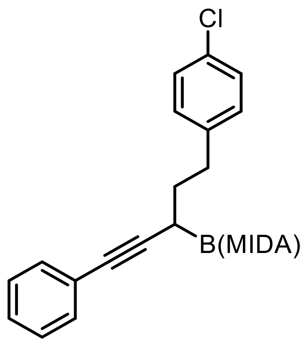

S9

The product **S9** was obtained in 27% (2.21 g) overall yield as a yellow solid after column chromatography (eluent = ethyl acetate/petroleum ether v/v 2:1).

^{1}H NMR (500 MHz, DMSO-*d*$_6$) δ 7.42 – 7.21 (m, 9H), 4.41 (d, J = 17.4 Hz, 1H), 4.29 (d, J = 17.0 Hz, 1H), 4.04 (d, J = 17.3 Hz, 1H), 3.94 (d, J = 16.9 Hz, 1H), 3.08 (s, 3H), 2.92 (dt, J = 13.8, 5.2 Hz, 1H), 2.72 (dt, J = 13.7, 8.3 Hz, 1H), 2.02 (dd, J = 10.9, 4.0 Hz, 1H), 1.90 – 1.72 (m, 2H).

^{13}C NMR (126 MHz, DMSO-*d*$_6$) δ 168.9, 168.3, 140.9, 131.2, 130.4, 130.3, 128.5, 128.3, 127.8, 123.4, 92.5, 82.0, 62.7, 62.4, 46.0, 33.9, 31.3.

HRMS (ESI): calculated for $C_{22}H_{21}BClNO_4$ $[M+Na]^+$, 432.1144; Found, 432.1140.

2-(1,5-diphenylpent-1-yn-3-yl)-6-methyl-1,3,6,2-dioxazaborocane-4,8-dione

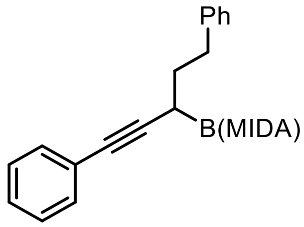

S10

The product **S10** was obtained in 22% (1.65 g) overall yield as a yellow solid after column chromatography (eluent = ethyl acetate/petroleum ether v/v 2:1).

¹H NMR (400 MHz, Acetonitrile-*d₃*) δ 7.41 – 7.26 (m, 9H), 7.22 – 7.14 (m, 1H), 4.04 (dd, *J* = 17.0, 11.1 Hz, 2H), 3.94 (d, *J* = 16.8 Hz, 1H), 3.87 (d, *J* = 17.2 Hz, 1H), 3.07 (s, 3H), 3.04 – 2.94 (m, 1H), 2.82 – 2.74 (m, 1H), 2.02 (dd, *J* = 10.9, 3.9 Hz, 1H), 1.93 – 1.80 (m, 2H)

^{13}C NMR (101 MHz, Acetonitrile-d_3) δ 169.1, 168.6, 143.2, 132.2, 129.5, 129.4, 129.2, 128.6, 126.7, 124.6, 93.2, 83.3, 63.7, 63.5, 46.7, 35.7, 32.5.

HRMS (ESI): calculated for $C_{22}H_{22}BNO_4$ $[M+Na]^+$, 398.1534; Found, 398.1537.

6-methyl-2-(1-phenylhept-6-en-1-yn-3-yl)-1,3,6,2-dioxazaborocane-4,8-dione--methane (1/6)

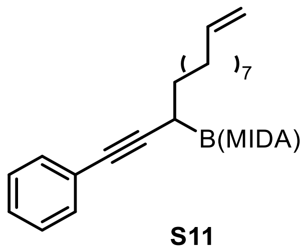

S11

The product **S11** was obtained in 43% (3.50 g) overall yield as a yellow solid after column chromatography (eluent = ethyl acetate/petroleum ether v/v 2:1).

^{1}H NMR (400 MHz, DMSO-d_6) δ 7.31 (s, 5H), 5.78 – 5.72 (m, 1H), 5.03 – 4.87 (m, 2H), 4.41 (d, *J* = 17.3 Hz, 1H), 4.27 (d, *J* = 16.9 Hz, 1H), 4.04 (d, *J* = 17.3 Hz, 1H), 3.91 (d, *J* = 16.9 Hz, 1H), 3.08 (s, 3H), 2.06 – 1.96 (m, 3H), 1.63 – 1.26 (m, 12H).

^{13}C NMR (101 MHz, DMSO-d_6) δ 169.0, 168.3, 138.8, 131.1, 128.5, 127.7, 123.6, 114.6, 93.3, 81.3, 62.6, 62.4, 45.9, 33.2, 29.3, 29.0, 28.9, 28.8, 28.6, 28.5, 28.3.

HRMS (ESI): calculated for $C_{24}H_{32}BNO_4$ $[M+Na]^+$, 432.2317; Found, 432.2319.

6-methyl-2-(5-phenoxy-1-phenylpent-1-yn-3-yl)-1,3,6,2-dioxazaborocane-4,8-dione

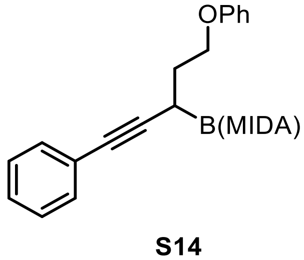

S14

The product **S14** was obtained in 10% (0.78 g) overall yield as a yellow solid after column chromatography (eluent = ethyl acetate/petroleum ether v/v 2:1).

^{1}H NMR (500 MHz, DMSO-d_6) δ 7.34 – 7.27 (m, 7H), 6.94 (dd, *J* = 26.2, 7.6 Hz, 3H), 4.45 (d, *J* = 17.3 Hz, 1H), 4.31 (d, *J* = 17.0 Hz, 1H), 4.22 – 4.16 (t, *J* = 5.4, 2H), 4.08 (d, *J* = 17.3 Hz, 1H), 3.96 (d, *J* = 17.1 Hz,

1H), 3.13 (s, 3H), 2.30 (dd, *J* = 11.3, 3.7 Hz, 1H), 2.13 – 2.03 (m, 1H), 1.93 – 1.81 (m, 1H).

^{13}C NMR (126 MHz, DMSO-*d$_6$*) δ 168.9, 168.3, 158.7, 131.2, 129.5, 128.5, 127.9, 123.2, 120.5, 114.5, 92.1, 81.7, 66.3, 62.8, 62.5, 46.1, 29.1.

HRMS (ESI): calculated for $C_{22}H_{22}BNO_5$ $[M+Na]^+$, 414.1483; Found, 414.1481.

2-(5-(benzyloxy)-1-phenylpent-1-yn-3-yl)-6-methyl-1,3,6,2-dioxazaborocane-4,8-dione

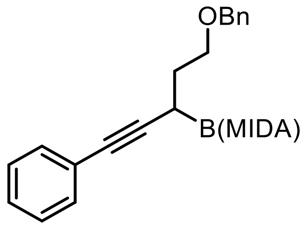

S15

The product **S15** was obtained in 47% (3.81 g) overall yield as a yellow solid after column chromatography (eluent = ethyl acetate/petroleum ether v/v 2:1).

^{1}H NMR (500 MHz, DMSO-*d$_6$*) δ 7.37 – 7.27 (m, 10H), 4.55 – 4.48 (m, 2H), 4.42 (d, *J* = 17.3 Hz, 1H), 4.29 (d, *J* = 17.0 Hz, 1H), 4.06 (d, *J* = 17.3 Hz, 1H), 3.94 (d, *J* = 16.9 Hz, 1H), 3.65 (dd, *J* = 7.8, 5.2 Hz, 2H), 3.10 (s, 3H), 2.20 (dd, *J* = 11.3, 3.6 Hz, 1H), 1.94 – 1.64 (m, 2H).

^{13}C NMR (101 MHz, DMSO-*d$_6$*) δ 168.9, 168.3, 138.7, 131.2, 128.5, 128.2, 127.8, 127.5, 127.3, 123.4, 92.5, 81.4, 71.9, 68.6, 62.7, 62.5, 46.0, 29.6.

HRMS (ESI): calculated for $C_{23}H_{24}BNO_5$ $[M+Na]^+$, 428.1640; Found, 428.1643.

4-(6-methyl-4,8-dioxo-1,3,6,2-dioxazaborocan-2-yl)-6-phenylhex-5-yn-1-yl formate

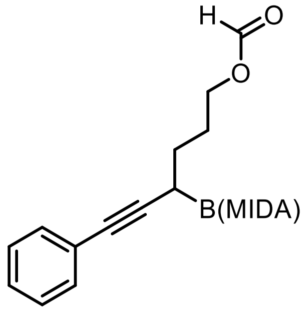

S16

The product **S16** was obtained in 9% (0.64 g) overall yield as a yellow solid after column chromatography (eluent = ethyl acetate/petroleum ether v/v 2:1).

^{1}H NMR (400 MHz, Acetonitrile-*d$_3$*) δ 8.08 (s, 1H), 7.36 – 7.30 (m, 5H), 4.21 (t, *J* = 6.0 Hz, 2H), 4.05 (dd, *J* = 17.0, 8.3 Hz, 2H), 3.91 (t, *J* = 17.3 Hz, 2H), 3.10 (s, 3H), 2.08 (dd, *J* = 10.3, 3.7 Hz, 1H), 1.88 – 1.53 (m, 4H).

^{13}C NMR (126 MHz, Acetonitrile-*d$_3$*) δ 169.1, 168.6, 162.5, 132.1, 129.4, 128.8, 124.5, 93.0, 83.1, 64.4, 63.7, 63.5, 46.7, 28.9, 26.9.

HRMS (ESI): calculated for $C_{18}H_{20}BNO_6$ [M+Na]$^+$, 380.1276; Found, 380.1277.

4-(6-methyl-4,8-dioxo-1,3,6,2-dioxazaborocan-2-yl)-6-phenylhex-5-yn-1-yl benzoate

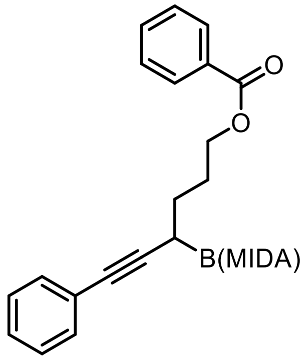

S17

The product **S17** was obtained in 13% (1.12 g) overall yield as a yellow solid after column chromatography (eluent = ethyl acetate/petroleum ether v/v 2:1).

^{1}H NMR (400 MHz, DMSO-*d$_6$*) δ 7.98 (d, *J* = 6.9 Hz, 2H), 7.66 (td, *J* = 7.3, 1.5 Hz, 1H), 7.52 (t, *J* = 7.7 Hz, 2H), 7.32 (s, 5H), 4.43 (d, *J* = 17.4 Hz, 1H), 4.36 (t, *J* = 6.4 Hz, 2H), 4.28 (d, *J* = 16.9 Hz, 1H), 4.06 (d, *J* = 17.3 Hz, 1H), 3.93 (d, *J* = 16.9 Hz, 1H), 3.10 (s, 3H), 2.15 (dd, *J* = 10.6, 4.0 Hz, 1H), 2.11 – 2.01 (m, 1H), 1.94 – 1.85 (m, 1H), 1.80 – 1.75 (m, 1H), 1.67 – 1.59 (m, 1H).

^{13}C NMR (101 MHz, DMSO-*d$_6$*) δ 168.9, 168.3, 165.8, 133.2, 131.1, 129.9, 129.1, 128.7, 128.5, 127.8, 123.4, 92.8, 81.7, 64.7, 62.6, 62.4, 46.0, 27.9, 26.0.

HRMS (ESI): calculated for $C_{24}H_{24}BNO_6$ [M+Na]$^+$, 456.1589; Found, 456.1584.

2-(1-cyclopropyl-3-phenylprop-2-yn-1-yl)-6-methyl-1,3,6,2-dioxazaborocane-4,8-dione

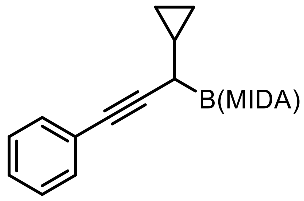

S24

The product **S24** was obtained in 36% (2.24 g) overall yield as a yellow solid after column chromatography (eluent = ethyl acetate/petroleum ether v/v 2:1).

^{1}H NMR (400 MHz, DMSO-*d$_6$*) δ 7.34 – 7.29 (m, 5H), 4.41 (d, *J* = 17.3 Hz, 1H), 4.28 (d, *J* = 16.9 Hz, 1H), 4.04 (d, *J* = 17.3 Hz, 1H), 3.92 (d, *J* = 16.9 Hz, 1H), 3.07 (s, 3H), 1.96 (d, *J* = 6.3 Hz, 1H), 0.47 – 0.42 (m,

2H), 0.34 – 0.30 (m, 2H).

^{13}C NMR (101 MHz, DMSO-*d$_6$*) δ 169.0, 168.4, 131.1, 128.6, 127.8, 123.4, 91.1, 81.6, 62.5, 62.3, 46.0, 10.1, 3.8, 3.2.

HRMS (ESI): calculated for $C_{17}H_{18}BNO_4$ [M+Na]$^+$, 334.1221; Found, 334.1226.

2-(1-cyclobutyl-3-phenylprop-2-yn-1-yl)-6-methyl-1,3,6,2-dioxazaborocane-4,8-dione

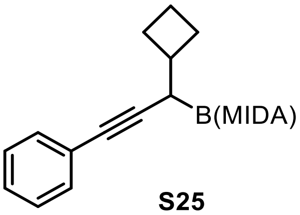

S25

The product **S25** was obtained in 30% (1.95 g) overall yield as a yellow solid after column chromatography (eluent = ethyl acetate/petroleum ether v/v 2:1).

^{1}H NMR (400 MHz, Acetonitrile-*d$_3$*) δ 7.36 – 7.32 (m, 5H), 4.36 (d, *J* = 17.3 Hz, 1H), 4.24 (d, *J* = 16.9 Hz, 1H), 4.02 (d, *J* = 17.2 Hz, 1H), 3.89 (d, *J* = 16.9 Hz, 1H), 3.07 (s, 3H), 2.66 – 2.57 (m, 1H), 2.09 (d, *J* = 6.7 Hz, 1H), 2.02 – 1.93 (m, 4H), 1.87 – 1.70 (m, 2H).

^{13}C NMR (126 MHz, DMSO-*d$_6$*) δ 169.0, 168.3, 131.2, 128.6, 127.7, 123.6, 91.6, 82.3, 62.4, 62.0, 45.9, 35.5, 27.9, 27.0, 18.0.

HRMS (ESI): calculated for $C_{18}H_{20}BNO_4$ [M+Na]$^+$, 348.1378; Found, 348.1378.

2-(1-cyclopentyl-3-phenylprop-2-yn-1-yl)-6-methyl-1,3,6,2-dioxazaborocane-4,8-dione

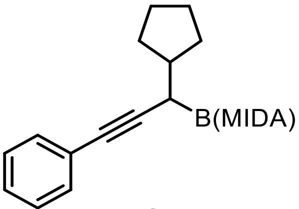

S26

The product **S26** was obtained in 30% (2.03 g) overall yield as a yellow solid after column chromatography (eluent = ethyl acetate/petroleum ether v/v 2:1).

^{1}H NMR (400 MHz, DMSO-*d$_6$*) δ 7.32 (s, 5H), 4.38 (d, *J* = 17.3 Hz, 1H), 4.24 (d, *J* = 16.9 Hz, 1H), 4.04 (d, *J* = 17.3 Hz, 1H), 3.88 (d, *J* = 16.9 Hz, 1H), 3.06 (s, 3H), 2.04 (d, *J* = 3.3 Hz, 1H), 1.88 (d, *J* = 9.2 Hz, 1H), 1.75 – 1.58 (m, 4H), 1.46 – 1.19 (m, 4H).

^{13}C NMR (101 MHz, DMSO-*d$_6$*) δ 169.1, 168.3, 131.1, 128.5, 127.6, 123.7, 91.7, 82.8, 62.3, 62.1, 45.9, 37.6, 33.5, 29.8, 26.4, 26.1.

HRMS (ESI): calculated for $C_{19}H_{22}BNO_4$ [M+Na]$^+$, 362.1534; Found, 362.1539.

2-(1-cyclohexyl-3-phenylprop-2-yn-1-yl)-6-methyl-1,3,6,2-dioxazaborocane-4,8-dione

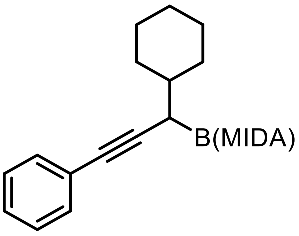

S27

The product **S27** was obtained in 58% (4.10 g) overall yield as a yellow solid after column chromatography (eluent = ethyl acetate/petroleum ether v/v 2:1).

^{1}H NMR (400 MHz, DMSO-*d$_6$*) δ 7.32 (s, 5H), 4.38 (d, *J* = 17.3 Hz, 1H), 4.23 (d, *J* = 16.9 Hz,1H), 4.04 (d, *J* = 17.2 Hz, 1H), 3.87 (d, *J* = 16.9 Hz, 1H), 3.06 (s, 3H), 2.04 (d, *J* = 3.3 Hz, 1H), 1.87 (d, *J* = 8.9 Hz, 1H), 1.75 – 1.59 (m, 5H), 1.46 – 1.19 (m, 5H).

^{13}C NMR (101 MHz, DMSO-*d$_6$*) δ 169.0, 168.3, 131.1, 128.5, 127.6, 123.7, 91.9, 82.7, 62.3, 62.0, 45.9, 37.5, 33.5, 29.8, 26.3, 26.0, 25.8.

HRMS (ESI): calculated for $C_{20}H_{24}BNO_4$ [M+Na]$^+$, 376.1691; Found, 376.1694.

6-methyl-2-(3-phenyl-1-(tetrahydro-2H-pyran-4-yl)prop-2-yn-1-yl)-1,3,6,2-dioxazaborocane-4,8-dione

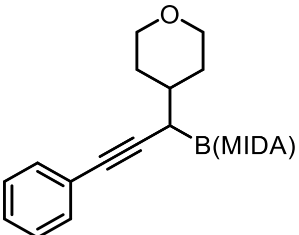

S28

The product **S28** was obtained in 46% (3.26 g) overall yield as a yellow solid after column chromatography (eluent = ethyl acetate/petroleum ether v/v 2:1).

^{1}H NMR (400 MHz, DMSO-*d$_6$*) δ 7.33 (s, 5H), 4.39 (d, *J* = 17.4 Hz, 1H), 4.25 (d, *J* = 16.9 Hz, 1H), 4.05 (d, *J* = 17.3 Hz, 1H), 3.93 – 3.83 (m, 3H), 3.28 (dd, *J* = 11.7, 2.2 Hz, 2H), 3.07 (s, 3H), 2.10 (d, *J* = 4.0 Hz, 1H), 1.85 (dt, *J* = 11.7, 3.9 Hz, 1H), 1.68 (td, *J* = 12.7, 4.3 Hz, 2H), 1.60 – 1.48 (m, 2H).

^{13}C NMR (101 MHz, DMSO-*d$_6$*) δ 169.0, 168.3, 131.1, 128.6, 127.8, 123.5, 91.2, 83.1, 67.3, 67.2, 62.4, 62.1, 46.0, 34.8, 32.9, 30.2.

HRMS (ESI): calculated for $C_{19}H_{22}BNO_5$ [M+Na]$^+$, 378.1483; Found, 378.1482.

2-(1-(cyclohex-3-en-1-yl)-3-phenylprop-2-yn-1-yl)-6-methyl-1,3,6,2-dioxazaborocane-4,8-dione

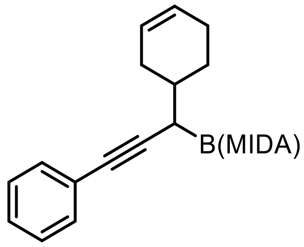

S29

The product **S29** was obtained in 32% (2.25 g) overall yield as a yellow solid after column chromatography (eluent = ethyl acetate/petroleum ether v/v 2:1), dr = 1:1.

^{1}H NMR (500 MHz, DMSO-*d$_6$*) δ 7.32 (s, 5H), 5.67 (dq, *J* = 22.9, 9.4, 7.5 Hz, 2H), 4.44 – 4.19 (m, 2H), 4.05 (dd, *J* = 17.3, 2.9 Hz, 1H), 3.90 (dd, *J* = 16.9, 3.5 Hz, 1H), 3.07 (d, *J* = 3.6 Hz, 3H), 2.21 – 1.63 (m, 8H).

^{13}C NMR (126 MHz, DMSO-*d$_6$*) δ 169.0, 169.0, 168.4, 168.3, 131.1, 128.5, 128.5, 127.7, 127.7, 127.1, 126.9, 126.6, 126.2, 123.6, 123.6, 91.5, 91.3, 82.8, 82.6, 62.4, 62.3, 62.1, 46.0, 45.9, 33.6, 33.5, 31.8, 29.3, 28.6, 25.9, 25.4, 25.3.

HRMS (ESI): calculated for $C_{20}H_{22}BNO_4$ [M+Na]$^+$, 374.1534; Found, 374.1530.

6-methyl-2-(4-methyl-1-phenylpent-1-yn-3-yl)-1,3,6,2-dioxazaborocane-4,8-dione

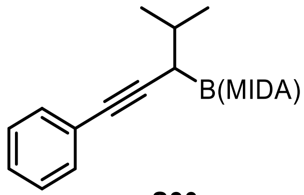

S30

The product **S30** was obtained in 18% (1.13 g) overall yield as a yellow solid after column chromatography (eluent = ethyl acetate/petroleum ether v/v 2:1).

^{1}H NMR (400 MHz, DMSO-*d$_6$*) δ 7.32 (s, 5H), 4.37 (d, *J* = 17.3 Hz, 1H), 4.24 (d, *J* = 16.8 Hz, 1H), 4.04 (d, *J* = 17.3 Hz, 1H), 3.89 (d, *J* = 17.0 Hz, 1H), 3.06 (s, 3H), 2.08 (d, *J* = 3.6 Hz, 1H), 2.05 – 1.96 (m, 1H), 1.07 (d, *J* = 6.7 Hz, 3H), 1.01 (d, *J* = 6.7 Hz, 3H).

^{13}C NMR (126 MHz, Chloroform-*d*) δ 167.4, 166.5, 131.6, 128.6, 128.1, 123.6, 90.4, 84.4, 62.8, 62.7, 45.5, 28.8, 23.9, 20.2.

HRMS (ESI): calculated for $C_{17}H_{20}BNO_4$ [M+Na]$^+$, 336.1378; Found, 336.1374.

6-methyl-2-(1-phenoxy-4-phenylbut-3-yn-2-yl)-1,3,6,2-dioxazaborocane-4,8-dione

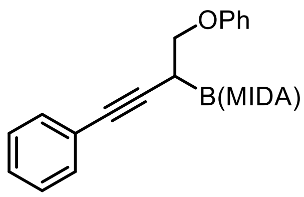

S78

The product **S78** was obtained in 16% (1.21 g) overall yield as a yellow solid after column chromatography (eluent = ethyl acetate/petroleum ether v/v 2:1).

^{1}H NMR (500 MHz, DMSO-*d*$_6$) δ 7.33 – 7.28 (m, 7H), 7.02 – 6.92 (m, 3H), 4.46 (d, *J* = 17.2 Hz, 1H), 4.35 (d, *J* = 17.0 Hz, 1H), 4.21 – 4.06 (m, 3H), 3.99 (d, *J* = 17.1 Hz, 1H), 3.17 (s, 3H), 2.72 (dd, *J* = 10.0, 4.1 Hz, 1H).

^{13}C NMR (126 MHz, Chloroform-*d*) δ 168.7, 168.3, 158.6, 131.2, 129.5, 128.5, 127.9, 123.2, 120.7, 114.7, 91.2, 81.6, 68.5, 62.7, 62.4, 46.4.

HRMS (ESI): calculated for $C_{21}H_{20}BNO_5$ $[M+H]^+$, 378.1507; Found, 378.1511.

General procedure B

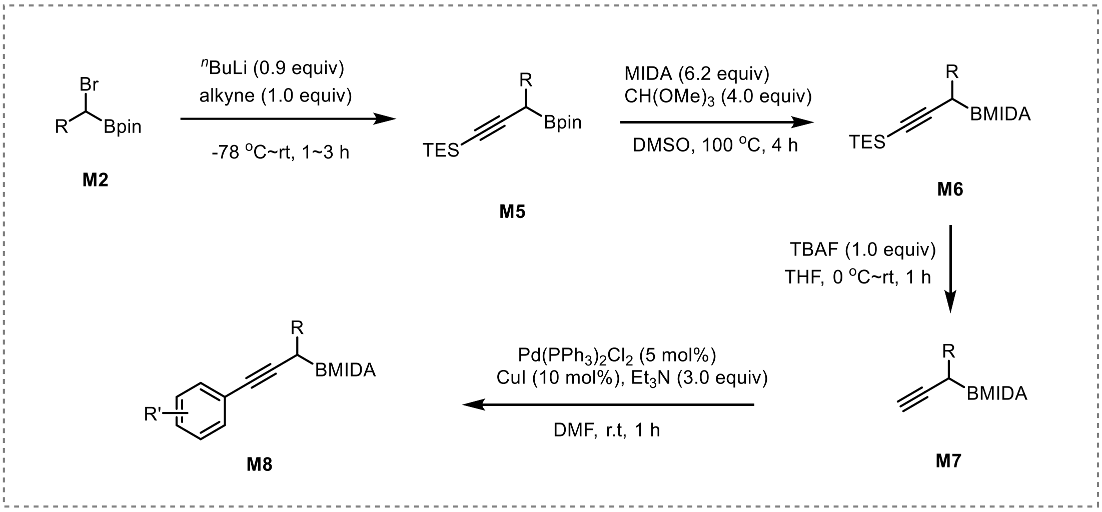

To a stirred solution of triethylsilylacetylene (10.0 mmol, 1.0 equiv, 1.43 g) and THF (20 mL), *n*-butyl lithium in hexane (2.5 M, 9.0 mmol, 0.9 equiv) was added dropwise at −78 °C (over 0.5 h). The resulting solution was stirred at the same temperature for 1 h, and then α-bromo boronic esters **M2** (10.0 mmol, 1.0 equiv) was added dropwise and the mixture was stirred at room temperature overnight. After the reaction, saturated NH_4Cl (aq) was added to the reaction mixture at 0 °C and the solution was stirred for 20 min. The reaction mixture was mixed with water and the product **M5** was extracted repeatedly with DCM. The organic phase was separated and the aqueous layer was extracted with DCM (30 mL) for three times.

The combined organic layer was dried over anhydrous Na_2SO_4 and concentrated under reduced pressure to afford the crude product **M5**.

Anhydrous DMSO (40 mL) was added to dissolve **M5** which was then added via syringe to a suspension of *N*-methyliminodiacetic acid (MIDA, 62.0 mmol, 6.2 equiv, 9.11 g) and $CH(OMe)_3$ (40.0 mmol, 4.0 equiv, 4.24 g) in DMSO. The resulting mixture was stirred at 100 °C until the propargyl boronic esters was used up by GC-MS monitoring. After cooling to room temperature, the reaction mixture was diluted with ethyl acetate (30 mL) and water (50 mL). The organic phase was separated and the aqueous layer was extracted with ethyl acetate (30 mL) for three times. The combined organic layer was dried over anhydrous Na_2SO_4 and concentrated under reduced pressure. The resulting crude product was purified by flash chromatography on silica gel with an appropriate solvent as eluent to afford the pure product **M6**.

To a stirred solution of **M6** (10.0 mmol, 1.0 equiv) in THF (30 mL) was added TBAF (10.0 mmol, 1.0 equiv, 2.61 g) at 0 °C and the mixture was stirred at the same temperature for 0.5 hour. Then, the mixture was stirred at room temperature for 0.5 h. After the **M6** was totally consumed, water was added to quench the reaction and the reaction mixture was diluted with EtOAc (20 mL). The organic phase was separated and the aqueous layer was extracted with EtOAc (20 mL) for three times. The combined organic layers were evaporated to give the crude products **M7**.

To a mixture of **M7** (1.0 mmol, 1.0 equiv), $Pd(PPh_3)_2Cl_2$ (0.05 mmol, 5 mol%, 35 mg) and CuI (0.1 mmol, 10 mol%, 19 mg) in dry DMF (2 mL, 0.5 M), were added Et_3N (3.0 equiv) and iodobenzene (1.2 mmol, 1.2 equiv) under the protection of nitrogen. The reaction mixture was stirred at room temperature until total consumption of the starting material, as monitored by TLC (about 1 hour). The reaction mixture was diluted with EA (20 mL) and water (10 mL). The organic phase was separated and the aqueous layer was extracted with EA (20 mL) for three times. The combined organic layer was dried over anhydrous Na_2SO_4 and concentrated under reduced pressure. The crude residue was purified by flash column (EA/PE as the eluent) to afford the product **M8**.

6-methyl-2-(1-(p-tolyl)oct-1-yn-3-yl)-1,3,6,2-dioxazaborocane-4,8-dione

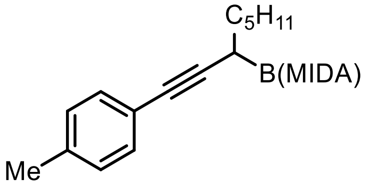

S31

The product **S31** was obtained in 16% (0.19 g) overall yield as a yellow solid after column chromatography

(eluent = ethyl acetate/petroleum ether v/v 2:1).

^{1}H NMR (400 MHz, Acetone-*d$_6$*) δ 7.24 (d, *J* = 8.1 Hz, 2H), 7.12 (d, *J* = 7.9 Hz, 2H), 4.30 (dd, *J* = 18.3, 16.9 Hz, 2H), 4.09 (dd, *J* = 16.9, 5.5 Hz, 2H), 3.34 (s, 3H), 2.30 (s, 3H), 1.72 – 1.31 (m, 10H), 0.91 (t, *J* = 6.9 Hz, 3H).

^{13}C NMR (126 MHz, Chloroform-*d*) δ 167.7, 167.1, 138.1, 131.4, 129.3, 120.5, 91.5, 82.6, 62.9, 62.9, 45.5, 31.7, 29.6, 29.0, 22.7, 21.5, 14.3.

HRMS (ESI): calculated for $C_{20}H_{26}BNO_4$ $[M+Na]^+$, 378.1847; Found, 378.1846.

2-(1-(4-methoxyphenyl)non-1-yn-3-yl)-6-methyl-1,3,6,2-dioxazaborocane-4,8-dione

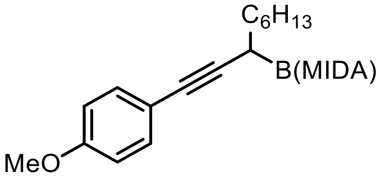

S32

The product **S32** was obtained in 17% (0.22 g) overall yield as a yellow solid after column chromatography (eluent = ethyl acetate/petroleum ether v/v 2:1).

^{1}H NMR (400 MHz, DMSO-*d$_6$*) δ 7.23 (d, *J* = 8.7 Hz, 2H), 6.89 (d, *J* = 8.7 Hz, 2H), 4.39 (d, *J* = 17.3 Hz, 1H), 4.25 (d, *J* = 16.9 Hz, 1H), 4.03 (d, *J* = 17.3 Hz, 1H), 3.89 (d, *J* = 16.9 Hz, 1H), 3.75 (s, 3H), 3.08 (s, 1H), 1.98 (dd, *J* = 10.2, 3.7 Hz, 1H), 1.64 – 1.27 (m, 10H), 0.87 (t, *J* = 6.4 Hz, 3H).

^{13}C NMR (101 MHz, DMSO-*d$_6$*) δ 168.9, 168.2, 158.7, 132.4, 115.6, 114.2, 91.4, 81.1, 62.6, 55.2, 55.1, 45.9, 31.2, 29.3, 28.5, 28.5, 22.1, 13.9.

HRMS (ESI): calculated for $C_{21}H_{28}BNO_5$ $[M+Na]^+$, 408.1953; Found, 408.1956.

2-(1-([1,1'-biphenyl]-4-yl)oct-1-yn-3-yl)-6-methyl-1,3,6,2-dioxazaborocane-4,8-dione

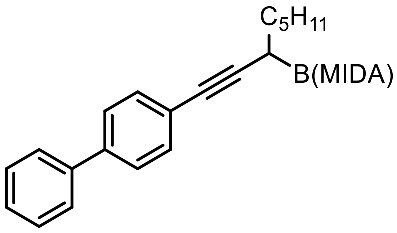

S33

The product **S33** was obtained in 14% (0.19 g) overall yield as a yellow solid after column chromatography (eluent = ethyl acetate/petroleum ether v/v 2:1).

^{1}H NMR (400 MHz, DMSO-*d$_6$*) δ 7.68 – 7.62 (m, 4H), 7.46 (dd, *J* = 8.3, 6.9 Hz, 2H), 7.42 – 7.35 (m, 3H),

4.42 (d, *J* = 17.4 Hz, 1H), 4.29 (d, *J* = 16.9 Hz, 1H), 4.06 (d, *J* = 17.3 Hz, 1H), 3.94 (d, *J* = 16.9 Hz, 1H), 3.11 (s, 3H), 2.06 (dd, *J* = 10.1, 3.8 Hz, 1H), 1.66 – 1.32 (m, 8H), 0.89 (t, *J* = 6.9 Hz, 3H).

^{13}C NMR (101 MHz, DMSO-d_6) δ 168.9, 168.3, 139.3, 139.3, 131.6, 129.0, 127.7, 126.7, 126.5, 122.6, 94.2, 81.2, 62.6, 45.9, 31.1, 29.2, 28.3, 22.0, 13.9.

HRMS (ESI): calculated for $C_{25}H_{28}BNO_4$ [M+Na]$^+$, 440.2004; Found, 440.2005.

2-(1-(4-fluorophenyl)oct-1-yn-3-yl)-6-methyl-1,3,6,2-dioxazaborocane-4,8-dione

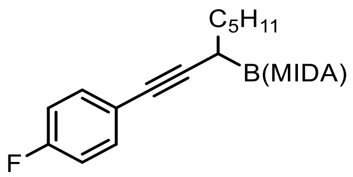

S34

The product **S34** was obtained in 14% (0.17 g) overall yield as a yellow solid after column chromatography (eluent = ethyl acetate/petroleum ether v/v 2:1).

^{1}H NMR (500 MHz, DMSO-d_6) δ 7.38 – 7.33 (m, 2H), 7.20 – 7.14 (m, 2H), 4.40 (d, *J* = 17.4 Hz, 1H), 4.26 (d, *J* = 16.9 Hz, 1H), 4.04 (d, *J* = 17.3 Hz, 1H), 3.92 (d, *J* = 17.0 Hz, 1H), 3.07 (s, 3H), 2.01 (dd, *J* = 10.4, 3.7 Hz, 1H), 1.65 – 1.27 (m, 8H), 0.88 (t, *J* = 6.9 Hz, 3H).

^{13}C NMR (126 MHz, DMSO-d_6) δ 169.0, 168.4, 161.4 (d, *J* = 246.0 Hz), 133.2 (d, *J* = 8.2 Hz), 120.0 (d, *J* = 3.3 Hz), 115.7 (d, *J* = 22.0 Hz), 93.0, 80.3, 62.6, 62.4, 46.0, 31.1, 29.2, 28.3, 22.1, 14.0.

HRMS (ESI): calculated for $C_{19}H_{23}BFNO_4$ [M+Na]$^+$, 382.1596; Found, 382.1592.

6-methyl-2-(1-(4-(trifluoromethoxy)phenyl)oct-1-yn-3-yl)-1,3,6,2-dioxazaborocane-4,8-dione

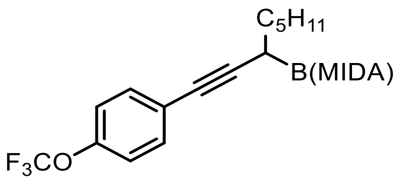

S35

The product **S35** was obtained in 12% (0.17 g) overall yield as a yellow solid after column chromatography (eluent = ethyl acetate/petroleum ether v/v 2:1).

^{1}H NMR (500 MHz, DMSO-d_6) δ 7.43 (d, *J* = 8.2 Hz, 2H), 7.33 (d, *J* = 8.3 Hz, 2H), 4.40 (d, *J* = 17.3 Hz, 1H), 4.26 (d, *J* = 17.0 Hz, 1H), 4.04 (d, *J* = 17.3 Hz, 1H), 3.94 (d, *J* = 16.9 Hz, 1H), 3.07 (s, 3H), 2.04 (dd, *J* = 10.4, 3.6 Hz, 1H), 1.64 – 1.29 (m, 8H), 0.88 (t, *J* = 6.7 Hz, 3H).

^{13}C NMR (101 MHz, DMSO-d_6) δ 169.0, 168.4, 147.5, 133.1, 1 0.1 (q, *J* = 277 Hz), 123.0, 121.3, 94.6, 80.0, 62.6, 62.4, 46.0, 31.1, 29.2, 28.3, 22.1, 14.0.

HRMS (ESI): calculated for $C_{20}H_{23}BF_3NO_5$ $[M+Na]^+$, 448.1514; Found, 448.1508.

6-methyl-2-(1-(4-(trifluoromethyl)phenyl)oct-1-yn-3-yl)-1,3,6,2-dioxazaborocane-4,8-dione

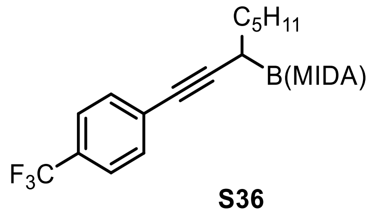

S36

The product **S36** was obtained in 18% (0.25 g) overall yield as a yellow solid after column chromatography (eluent = ethyl acetate/petroleum ether v/v 2:1).

^{1}H NMR (400 MHz, DMSO-*d$_6$*) δ 7.69 (d, *J* = 8.0 Hz, 2H), 7.53 (d, *J* = 8.1 Hz, 2H), 4.41 (d, *J* = 17.4 Hz, 1H), 4.29 (d, *J* = 17.0 Hz, 1H), 4.07 (d, *J* = 17.3 Hz, 1H), 3.96 (d, *J* = 17.0 Hz, 1H), 3.10 (s, 3H), 2.10 (dd, *J* = 10.2, 3.5 Hz, 1H), 1.66 – 1.30 (m, 8H), 0.89 (t, *J* = 7.0 Hz).

^{13}C NMR (101 MHz, DMSO-*d$_6$*) δ 168.8, 168.3, 131.8, 125.4, 125.4, 125.3, 125.3, 122.7, 96.8, 80.3, 62.6, 62.5, 46.0, 31.0, 29.2, 28.2, 22.0, 13.9.

HRMS (ESI): calculated for $C_{20}H_{23}BF_3NO_4$ $[M+Na]^+$, 432.1564; Found, 432.1564.

3-(3-(6-methyl-4,8-dioxo-1,3,6,2-dioxazaborocan-2-yl)oct-1-yn-1-yl)benzaldehyde

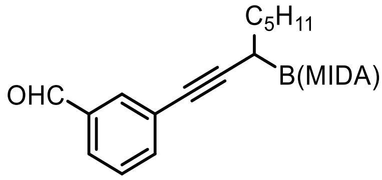

S37

The product **S37** was obtained in 10% (0.12 g) overall yield as a yellow solid after column chromatography (eluent = ethyl acetate/petroleum ether v/v 2:1).

^{1}H NMR (400 MHz, Chloroform-*d*) δ 9.94 (s, 1H), 7.78 (s, 1H), 7.75 (d, *J* = 7.6 Hz, 1H), 7.56 (d, *J* = 7.7 Hz, 1H), 7.44 (t, *J* = 7.7 Hz, 1H), 4.08 – 3.95 (m, 3H), 3.87 (d, *J* = 16.8 Hz, 1H), 3.18 (s, 3H), 2.00 (dd, *J* = 10.3, 3.3 Hz, 1H), 1.79 – 1.58 (m, 4H), 1.48 – 1.29 (m, 4H), 0.88 (t, *J* = 6.44 Hz, 3H).

^{13}C NMR (101 MHz, Chloroform-*d*) δ 191.9, 167.9, 167.3, 137.2, 136.6, 132.2, 129.4, 129.2, 124.8, 94.3, 81.34, 63.0, 45.8, 31.7, 29.8, 29.0, 22.7, 14.2.

HRMS (ESI): calculated for $C_{20}H_{24}BNO_5$ $[M+Na]^+$, 392.1640; Found, 392.1635.

2-(1-(4-acetylphenyl)non-1-yn-3-yl)-6-methyl-1,3,6,2-dioxazaborocane-4,8-dione

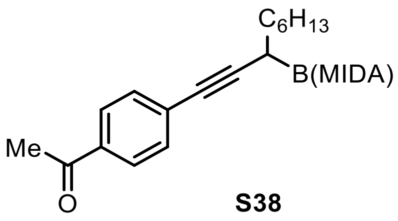

S38

The product **S38** was obtained in 14% (0.18 g) overall yield as a yellow solid after column chromatography (eluent = ethyl acetate/petroleum ether v/v 2:1).

^{1}H NMR (400 MHz, DMSO-*d$_6$*) δ 7.90 (d, *J* = 8.4 Hz, 2H), 7.44 (d, *J* = 8.4 Hz, 2H), 4.40 (d, *J* = 17.3 Hz, 1H), 4.28 (d, *J* = 17.0 Hz, 1H), 4.05 (d, *J* = 17.3 Hz, 1H), 3.93 (d, *J* = 17.0 Hz, 1H), 3.09 (s, 3H), 2.56 (s, 3H), 2.09 (dd, *J* = 10.2, 3.4 Hz, 1H), 1.63 – 1.28 (m, 10H), 0.91 – 0.84 (t, *J* = 6.7 Hz, 3H).

^{13}C NMR (101 MHz, DMSO-*d$_6$*) δ 197.2, 168.8, 168.3, 135.5, 131.3, 128.3, 97.3, 81.0, 62.6, 46.0, 31.2, 29.2, 28.6, 26.7, 22.1, 13.9.

HRMS (ESI): calculated for $C_{22}H_{28}BNO_5$ [M+Na]$^+$, 420.1953; Found, 420.1950.

2-(1-(3,5-dimethylphenyl)oct-1-yn-3-yl)-6-methyl-1,3,6,2-dioxazaborocane-4,8-dione

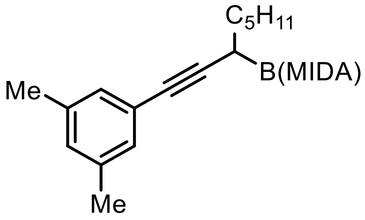

S39

The product **S39** was obtained in 7% (0.09 g) overall yield as a yellow solid after column chromatography (eluent = ethyl acetate/petroleum ether v/v 2:1).

1H NMR (400 MHz, Chloroform-*d*) δ 7.18 (s, 1H), 6.86 (s, 1H), 6.83 (s, 1H), 3.98 (d, *J* = 15.8 Hz, 1H), 3.82 – 3.71 (m, 3H), 3.07 (s, 3H), 2.18 (s, 6H), 1.88 (dd, *J* = 10.5, 3.3 Hz, 1H), 1.66 – 1.58 (m, 2H), 1.39 – 1.21 (m, 6H), 0.85 – 0.77 (m, 3H).

^{13}C NMR (101 MHz, Chloroform-*d*) δ 167.7, 167.0, 138.2, 129.9, 129.1, 123.2, 91.5, 82.9, 63.0, 62.9, 45.5, 31.7, 29.6, 29.0, 22.7, 21.2, 14.3.

HRMS (ESI): calculated for $C_{21}H_{28}BNO_4$ [M+Na]$^+$, 392.2004; Found, 392.2006.

2-(1-(3-fluoro-4-methylphenyl)oct-1-yn-3-yl)-6-methyl-1,3,6,2-dioxazaborocane-4,8-dione

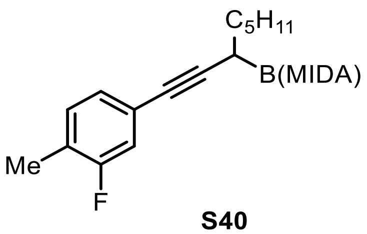

S40

The product **S40** was obtained in 9% (0.11 g) overall yield as a yellow solid after column chromatography (eluent = ethyl acetate/petroleum ether v/v 2:1).

^{1}H NMR (400 MHz, DMSO-*d$_6$*) δ 7.23 (t, *J* = 8.0 Hz, 1H), 7.10 – 7.01 (m, 2H), 4.40 (d, *J* = 17.3 Hz, 1H), 4.26 (d, *J* = 17.0 Hz, 1H), 4.04 (d, *J* = 17.3 Hz, 1H), 3.92 (d, *J* = 17.0 Hz, 1H), 3.07 (s, 3H), 2.21 (d, *J* = 1.9 Hz, 3H), 2.02 (dd, *J* = 10.2, 3.7 Hz, 1H), 1.61 – 1.28 (m, 8H), 0.88 (t, *J* = 6.8 Hz, 3H).

^{13}C NMR (101 MHz, DMSO-d$_6$) δ 164.3, 163.7, 155.6 (d, *J* = 243.3 Hz), 127.1 (d, *J* = 5.8 Hz), 122.5 (d, *J* = 3.0 Hz), 119.7 (d, *J* = 17.0 Hz), 118.1 (d, *J* = 9.7 Hz), 112.6 (d, *J* = 23.1 Hz), 89.1, 75.7, 75.6, 58.0, 57.8, 41.4, 26.4, 24.5, 23.6, 17.4, 9.3.

HRMS (ESI): calculated for $C_{20}H_{25}BFNO_4$ $[M+Na]^+$, 396.1753; Found, 396.1751.

Methyl 2-methyl-5-(3-(6-methyl-4,8-dioxo-1,3,6,2-dioxazaborocan-2-yl)oct-1-yn-1-yl)benzoate

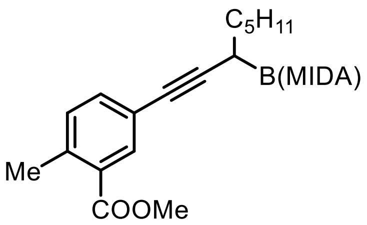

S41

The product **S41** was obtained in 10% (0.14 g) overall yield as a yellow solid after column chromatography (eluent = ethyl acetate/petroleum ether v/v 2:1).

^{1}H NMR (400 MHz, DMSO-*d$_6$*) δ 7.70 (s, 1H), 7.40 (d, *J* = 7.8 Hz, 1H), 7.29 (d, *J* = 7.9 Hz, 1H), 4.39 (d, *J* = 17.3 Hz, 1H), 4.26 (d, *J* = 16.9 Hz, 1H), 4.04 (d, *J* = 17.3 Hz, 1H), 3.92 (d, *J* = 17.0 Hz, 1H), 3.08 (s, 3H), 2.48 (s, 3H), 2.04 (dd, *J* = 10.2, 3.6 Hz, 1H), 1.62 – 1.29 (m, 8H), 0.91 – 0.86 (t, *J* = 7.5 Hz, 3H).

^{13}C NMR (126 MHz, DMSO-*d$_6$*) δ 169.0, 168.4, 166.7, 138.5, 134.4, 132.3, 132.0, 129.8, 121.3, 93.8, 80.3, 62.6, 62.4, 52.1, 46.0, 31.1, 29.2, 28.3, 22.1, 20.9, 14.0.

HRMS (ESI): calculated for $C_{22}H_{28}BNO_6$ $[M+Na]^+$, 436.1902; Found, 436.1903.

2-(1-(4-bromo-3,5-dimethylphenyl)oct-1-yn-3-yl)-6-methyl-1,3,6,2-dioxazaborocane-4,8-dione

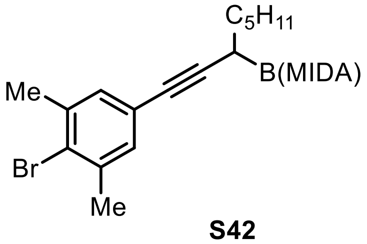

S42

The product **S42** was obtained in 5% (0.07 g) overall yield as a yellow solid after column chromatography (eluent = ethyl acetate/petroleum ether v/v 2:1).

^{1}H NMR (500 MHz, Chloroform-*d*) δ 7.19 (s, 1H), 6.96 (s, 1H), 3.96 (d, *J* = 15.9 Hz, 1H), 3.83 – 3.73 (m, 3H), 3.08 (s, 3H), 2.29 (s, 6H), 1.88 (dd, *J* = 10.4, 3.4 Hz, 1H), 1.66 – 1.59 (m, 2H), 1.38 – 1.21 (m, 6H), 0.82 (t, *J* = 6.8 Hz, 3H).

^{13}C NMR (126 MHz, Chloroform-*d*) δ 167.0, 166.4, 138.8, 130.9, 127.6, 121.8, 92.6, 82.1, 63.01, 45.5, 31.7, 29.5, 29.1, 23.8, 22.7, 14.3.

HRMS (ESI): calculated for $C_{21}H_{27}BBrNO_4$ $[M+Na]^+$, 470.1109; Found, 470.1111.

2-(1-(4-hydroxyphenyl)oct-1-yn-3-yl)-6-methyl-1,3,6,2-dioxazaborocane-4,8-dione

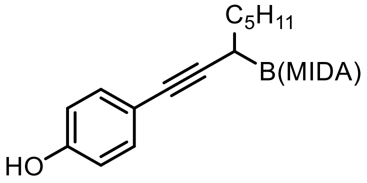

S43

The product **S43** was obtained in 9% (0.11 g) overall yield as a yellow solid after column chromatography (eluent = ethyl acetate/petroleum ether v/v 2:1).

^{1}H NMR (400 MHz, DMSO-*d₆*) δ 9.65 (s, 1H), 7.12 (d, *J* = 8.6 Hz, 2H), 6.70 (d, *J* = 8.6 Hz, 2H), 4.39 (d, *J* = 17.4 Hz, 1H), 4.25 (d, *J* = 16.8 Hz, 1H), 4.02 (d, *J* = 17.3 Hz, 1H), 3.88 (d, *J* = 16.8 Hz, 1H), 3.07 (s, 3H), 1.96 (dd, *J* = 10.1, 3.8 Hz, 1H), 1.60 – 1.27 (m, 8H), 0.88 (t, *J* = 6.8 Hz, 3H).

^{13}C NMR (126 MHz, DMSO-d_6) δ 169.1, 168.3, 157.1, 132.5, 115.5, 113.9, 90.5, 81.5, 62.6, 62.3, 45.8, 31.1, 29.3, 28.3, 22.1, 14.0.

HRMS (ESI): calculated for $C_{19}H_{24}BNO_5$ $[M+Na]^+$, 380.1640; Found, 380.1636.

2-(1-(4-hydroxy-3,5-dimethylphenyl)oct-1-yn-3-yl)-6-methyl-1,3,6,2-dioxazaborocane-4,8-dione

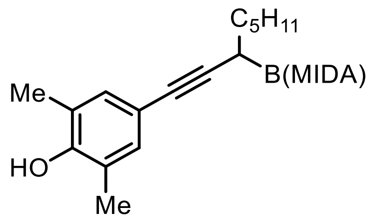

S44

The product **S44** was obtained in 10% (0.13 g) overall yield as a yellow solid after column chromatography (eluent = ethyl acetate/petroleum ether v/v 2:1).

^{1}H NMR (400 MHz, Chloroform-*d*) δ 6.96 (s, 2H), 4.08 (d, *J* = 15.8 Hz, 1H), 3.85 – 3.78 (m, 3H), 3.15 (s, 3H), 2.19 (s, 6H), 1.94 (d, *J* = 10.2 Hz, 1H), 1.72 – 1.67 (m, 2H), 1.32 (q, *J* = 6.8 Hz, 6H), 0.93 – 0.85 (m, 3H).

^{13}C NMR (126 MHz, Chloroform-*d*) δ 167.1, 166.6, 152.4, 131.8, 123.4, 115.0, 89.8, 82.7, 63.0, 63.0, 45.4, 31.8, 29.6, 29.0, 22.7, 15.8, 14.3.

HRMS (ESI): calculated for $C_{21}H_{28}BNO_5$ $[M+Na]^+$, 408.1953; Found, 408.1959.

6-methyl-2-(1-(naphthalen-2-yl)non-1-yn-3-yl)-1,3,6,2-dioxazaborocane-4,8-dione

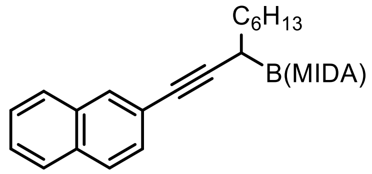

S45

The product **S45** was obtained in 8% (0.11 g) overall yield as a yellow solid after column chromatography (eluent = ethyl acetate/petroleum ether v/v 2:1).

^{1}H NMR (500 MHz, Chloroform-*d*) δ 7.84 – 7.72 (m, 4H), 7.50 – 7.44 (m, 2H), 7.36 (dd, *J* = 8.4, 1.6 Hz, 1H), 4.06 (d, *J* = 15.9 Hz, 1H), 3.90 – 3.82 (m, 2H), 3.79 (d, *J* = 16.6 Hz, 1H), 3.15 (s, 3H), 2.01 (dd, *J* = 10.2, 3.4 Hz, 1H), 1.76 – 1.70 (m, 2H), 1.46 – 1.29 (m, 6H), 0.88 (t, *J* = 5.79 Hz, 3H).

^{13}C NMR (126 MHz, Chloroform-*d*) δ 167.4, 166.8, 133.1, 132.7, 131.0, 128.5, 128.3, 127.9, 127.7, 126.8, 126.7, 120.8, 92.7, 83.0, 63.0, 63.0, 45.5, 31.9, 29.7, 29.4, 29.2, 22.8, 14.3.

HRMS (ESI): calculated for $C_{24}H_{28}BNO_4$ $[M+Na]^+$, 428.2004; Found, 428.2003.

2-(1-(9H-fluoren-2-yl)oct-1-yn-3-yl)-6-methyl-1,3,6,2-dioxazaborocane-4,8-dione

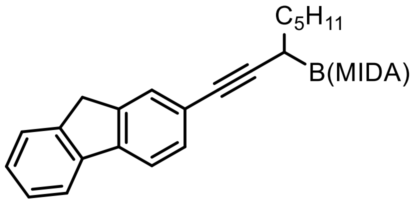

S46

The product **S46** was obtained in 8% (0.11 g) overall yield as a yellow solid after column chromatography (eluent = ethyl acetate/petroleum ether v/v 2:1).

^{1}H NMR (400 MHz, DMSO-*d$_6$*) δ 7.87 (dd, *J* = 16.0, 7.7 Hz, 2H), 7.61 (dd, *J* = 19.5, 7.6 Hz, 2H), 7.35 (dd, *J* = 18.3, 7.6 Hz, 3H), 4.42 (d, *J* = 17.3 Hz, 1H), 4.29 (d, *J* = 16.9 Hz, 1H), 4.06 (d, *J* = 17.2 Hz, 1H), 3.96 (d, *J* = 17.0 Hz, 1H), 3.91 (s, 2H), 3.12 (s, 3H), 2.09 – 2.01 (m, 1H), 1.67 – 1.33 (m, 8H), 0.94 – 0.87 (m, 3H).

^{13}C NMR (126 MHz, DMSO-*d$_6$*) δ 169.0, 168.4, 143.3, 143.2, 140.6, 140.5, 130.0, 127.8, 127.1, 126.9, 125.2, 121.7, 120.3, 120.1, 93.2, 82.0, 62.6, 62.4, 46.0, 36.2, 31.1, 29.3, 28.3, 22.1, 14.0.

HRMS (ESI): calculated for $C_{26}H_{28}BNO_4$ $[M+Na]^+$, 452.2004; Found, 452.2002.

6-methyl-2-(1-(thiophen-2-yl)oct-1-yn-3-yl)-1,3,6,2-dioxazaborocane-4,8-dione

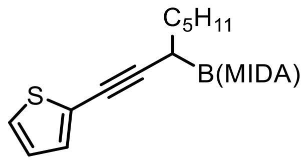

S47

The product **S47** was obtained in 13% (0.15 g) overall yield as a yellow solid after column chromatography (eluent = ethyl acetate/petroleum ether v/v 2:1).

^{1}H NMR (400 MHz, DMSO-*d$_6$*) δ 7.46 (dd, *J* = 5.2, 1.2 Hz, 1H), 7.12 (dd, *J* = 3.6, 1.2 Hz, 1H), 7.01 (dd, *J* = 5.2, 3.6 Hz, 1H), 4.39 (d, *J* = 17.3 Hz, 1H), 4.28 (d, *J* = 16.9 Hz, 1H), 4.04 (d, *J* = 17.3 Hz, 1H), 3.87 (d, *J* = 16.9 Hz, 1H), 3.06 (s, 3H), 2.07 (dd, *J* = 10.2, 3.4 Hz, 1H), 1.61 – 1.28 (m, 8H), 0.88 (t, *J* = 6.7 Hz, 3H).

^{13}C NMR (101 MHz, DMSO-*d$_6$*) δ 168.9, 168.3, 131.1, 127.4, 127.0, 123.3, 97.5, 74.5, 62.6, 62.4, 46.0, 31.1, 29.1, 28.3, 22.1, 14.0.

HRMS (ESI): calculated for $C_{17}H_{22}BNO_4S$ $[M+Na]^+$, 370.1255; Found, 370.1254.

6-methyl-2-(1-(thiophen-3-yl)oct-1-yn-3-yl)-1,3,6,2-dioxazaborocane-4,8-dione

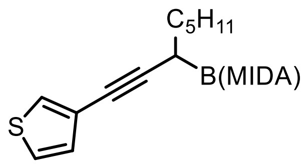

S48

The product **S48** was obtained in 12% (0.14 g) overall yield as a yellow solid after column chromatography (eluent = ethyl acetate/petroleum ether v/v 2:1).

^{1}H NMR (400 MHz, DMSO-*d6*) δ 7.57 – 7.52 (m, 2H), 7.03 (dd, *J* = 5.0, 1.3 Hz, 1H), 4.39 (d, *J* = 17.3 Hz, 1H), 4.26 (d, *J* = 16.9 Hz, 1H), 4.03 (d, *J* = 17.3 Hz, 1H), 3.90 (d, *J* = 16.9 Hz, 1H), 3.07 (s, 3H), 1.99 (dd, *J* = 10.2, 3.8 Hz, 1H), 1.62 – 1.29 (m, 8H), 0.91 – 0.86 (t, *J* = 6.9 Hz, 3H).

^{13}C NMR (101 MHz, DMSO-*d6*) δ 168.9, 168.2, 129.6, 127.9, 126.3, 122.3, 92.4, 76.6, 62.6, 62.3, 45.9, 31.1, 29.1, 28.2, 22.0, 13.9.

HRMS (ESI): calculated for $C_{17}H_{22}BNO_4S$ $[M+Na]^+$, 370.1255; Found, 370.1252.

5-(3-(6-methyl-4,8-dioxo-1,3,6,2-dioxazaborocan-2-yl)oct-1-yn-1-yl)furan-2-carbaldehyde

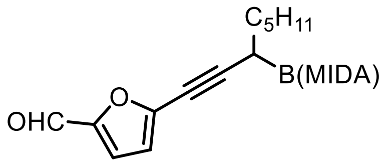

S49

The product **S49** was obtained in 9% (0.11 g) overall yield as a yellow solid after column chromatography (eluent = ethyl acetate/petroleum ether v/v 2:1).

^{1}H NMR (400 MHz, DMSO-*d6*) δ 9.52 (s, 1H), 7.53 (d, *J* = 3.7 Hz, 1H), 6.85 (d, *J* = 3.7 Hz, 1H), 4.38 (d, *J* = 17.2 Hz, 1H), 4.30 (d, *J* = 17.1 Hz, 1H), 4.07 (d, *J* = 17.2 Hz, 1H), 3.96 (d, *J* = 17.1 Hz, 1H), 3.06 (s, 3H), 2.21 (dd, *J* = 10.4, 2.9 Hz, 1H), 1.58 (dd, *J* = 10.5, 6.8 Hz, 2H), 1.44 – 1.23 (m, 6H), 0.88 (t, *J* = 6.8 Hz, 3H).

^{13}C NMR (126 MHz, DMSO-*d6*) δ 177.7, 168.7, 168.4, 151.5, 141.3, 124.0, 116.5, 102.4, 71.1, 62.5, 62.4, 46.2, 31.0, 28.9, 28.3, 22.0, 14.0.

HRMS (ESI): calculated for $C_{18}H_{22}BNO_6$ $[M+Na]^+$, 382.1432; Found, 382.1437.

methyl 2-(3-cyclohexyl-3-(6-methyl-4,8-dioxo-1,3,6,2-dioxazaborocan-2-yl)prop-1-yn-1-yl)benzoate

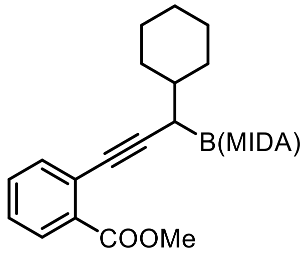

S50

The product **S50** was obtained in 12% (0.16 g) overall yield as a yellow solid after column chromatography (eluent = ethyl acetate/petroleum ether v/v 2:1).

^{1}H NMR (500 MHz, Chloroform-*d*) δ 7.96 (s, 1H), 7.92 (d, *J* = 7.9 Hz, 1H), 7.50 (d, *J* = 7.8 Hz, 1H), 7.35 (t, *J* = 7.8 Hz, 1H), 4.02 – 3.89 (m, 6H), 3.80 (d, *J* = 16.9 Hz, 1H), 3.14 (s, 3H), 2.00 (d, *J* = 9.9 Hz, 1H), 1.92 (d, *J* = 4.1 Hz, 1H), 1.82 – 1.59 (m, 6H), 1.47 – 1.24 (m, 4H).

^{13}C NMR (126 MHz, Chloroform-*d*) δ 167.8, 166.9, 166.6, 135.9, 132.4, 130.6, 128.9, 128.8, 124.1, 92.2, 83.2, 62.8, 62.7, 52.5, 45.6, 38.2, 34.1, 30.6, 26.9, 26.5, 26.2.

HRMS (ESI): calculated for $C_{22}H_{26}BNO_6$ [M+Na]$^+$, 434.1745; Found, 434.1748.

methyl 2-methyl-4-(3-(6-methyl-4,8-dioxo-1,3,6,2-dioxazaborocan-2-yl)oct-1-yn-1-yl)benzoate

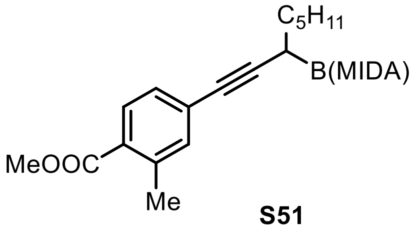

S51

The product **S51** was obtained in 4% (0.06 g) overall yield as a yellow solid after column chromatography (eluent = ethyl acetate/petroleum ether v/v 2:1).

^{1}H NMR (500 MHz, Chloroform-*d*) δ 7.83 (d, *J* = 8.0 Hz, 1H), 7.20 – 7.16 (m, 2H), 4.01 (d, *J* = 15.9 Hz, 1H), 3.91 – 3.81 (m, 6H), 3.16 (s, 3H), 1.99 (dd, *J* = 10.3, 3.8 Hz, 1H), 1.73 – 1.66 (m, 2H), 1.46 – 1.29 (m, 6H), 0.89 (t, *J* = 6.9 Hz, 3H).

^{13}C NMR (126 MHz, Chloroform-*d*) δ 167.6, 167.0, 166.4, 140.7, 134.5, 131.0, 128.9, 128.7, 127.2, 95.1, 82.2, 63.0, 63.0, 52.1, 45.6, 31.7, 29.5, 29.1, 22.7, 21.7, 14.3.

HRMS (ESI): calculated for $C_{22}H_{28}BNO_6$ [M+Na]$^+$, 436.1902; Found, 436.1902.

General procedure C

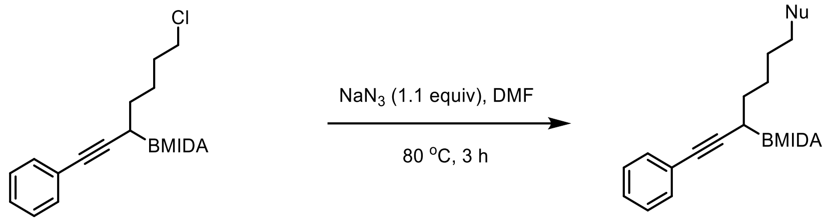

M9

To a stirred solution of **M9** (0.1 mmol, 1 equiv) in anhydrous DMF (2 mL) was added NaN_3 (0.22 mmol, 1.1 equiv). The reaction mixture was then stirred at 80 °C for 3 h. After the reaction was complete, the reaction mixture was quenched with water (5 mL) and extracted with EtOAc. The combined organic layers were concentrated in vacuo and the residue was purified by flash chromatography on silica gel with a mixture of Petroleum ether and EtOAc as eluent to afford the pure product.

2-(7-azido-1-phenylhept-1-yn-3-yl)-6-methyl-1,3,6,2-dioxazaborocane-4,8-dione

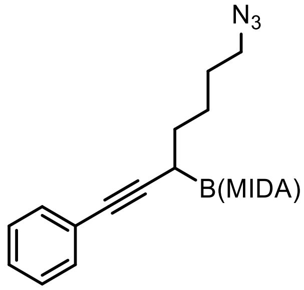

S12

The product **S12** was obtained in 91% (33 mg) yield as a yellow solid after column chromatography (eluent = ethyl acetate/petroleum ether v/v 2:1).

^{1}H NMR (400 MHz, DMSO-*d₆*) δ 7.32 (s, 5H), 4.41 (d, *J* = 17.3 Hz, 1H), 4.27 (d, *J* = 16.9 Hz, 1H), 4.05 (d, *J* = 17.4 Hz, 1H), 3.92 (d, *J* = 16.9 Hz, 1H), 3.36 (td, *J* = 6.8, 1.8 Hz, 2H), 3.09 (s, 3H), 2.08 – 2.03 (m, 1H), 1.76 – 1.43 (m, 6H).

^{13}C NMR (126 MHz, Chloroform-*d*) δ 167.2, 166.6, 131.5, 128.7, 128.2, 123.3, 91.6, 83.1, 63.0, 51.5, 45.5, 29.2, 28.8, 26.4.

HRMS (ESI): calculated for $C_{18}H_{21}BN_4O_4$ [M+Na]$^+$, 391.1548; Found, 391.1551.

6-methyl-2-(1-phenyl-7-tosylhept-1-yn-3-yl)-1,3,6,2-dioxazaborocane-4,8-dione

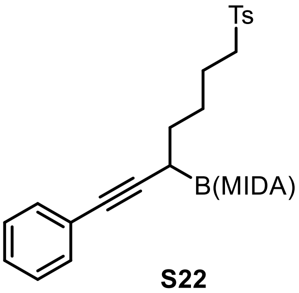

S22

The product **S22** was obtained in 42% (20 mg) yield as a yellow solid after column chromatography (eluent = ethyl acetate/petroleum ether v/v 2:1).

^{1}H NMR (400 MHz, Chloroform-*d*) δ 7.74 (d, *J* = 8.1 Hz, 2H), 7.26 – 7.34 (m, 7H), 3.98 (d, *J* = 16.0 Hz, 1H), 3.93 – 3.76 (m, 3H), 3.15 – 3.05 (m, 5H), 2.41 (s, 3H), 1.92 (dd, *J* = 9.7, 3.7 Hz, 1H), 1.73 – 1.62 (m, 6H).

^{13}C NMR (101 MHz, Chloroform-*d*) δ 167.3, 166.7, 144.8, 136.3, 131.5, 130.1, 128.6, 128.2, 123.3, 91.5, 83.1, 63.0, 56.4, 45.6, 29.1, 27.7, 22.6, 21.7.

HRMS (ESI): calculated for $C_{25}H_{28}BNO_6S$ $[M+Na]^+$, 504.1623; Found, 504.1625.

General procedure D

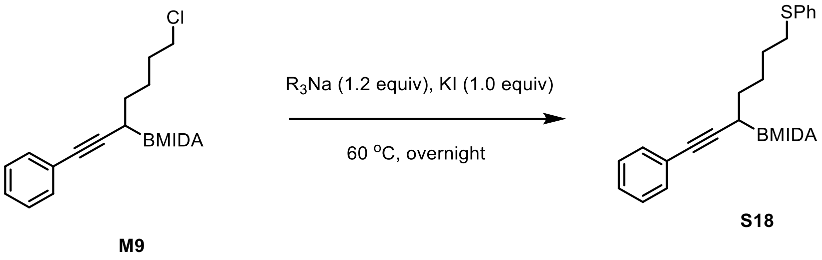

To the 15 mL Schlenk tube were added **M9** (0.1 mmol, 1.0 equiv), sodium thiophenolate (0.24 mmol, 1.2 equiv) and DMF (2 mL). The reaction mixture was vigorously stirred at 60 °C until the reaction was complete as monitored by TLC analysis. The reaction mixture was quenched with water (5 mL) and extracted with EtOAc. The combined organic layers were concentrated in vacuo and the residue was purified by flash chromatography on silica gel with a mixture of Petroleum ether and EtOAc as eluent (eluent = Petroleum ether/EtOAc 1:3 v/v) to afford the pure product.

6-methyl-2-(1-phenyl-7-(phenylthio)hept-1-yn-3-yl)-1,3,6,2-dioxazaborocane-4,8-dione

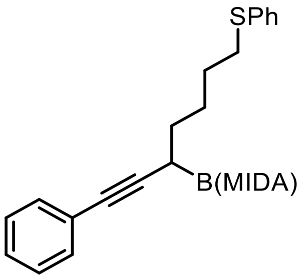

S18

The product **S18** was obtained in 80% (35 mg) yield as a yellow solid after column chromatography (eluent = ethyl acetate/petroleum ether v/v 2:1).

^{1}H NMR (400 MHz, DMSO-*d*$_6$) δ 7.36 – 7.26 (m, 9H), 7.19 – 7.13 (m, 1H), 4.41 (d, *J* = 17.4 Hz, 1H), 4.27 (d, *J* = 17.0 Hz, 1H), 4.04 (d, *J* = 17.3 Hz, 1H), 3.92 (d, *J* = 16.9 Hz, 1H), 3.08 (s, 3H), 2.99 (td, *J* = 7.0, 2.2 Hz, 2H), 2.03 (dd, *J* = 10.1, 3.6 Hz, 1H), 1.82 – 1.47 (m, 6H).

^{13}C NMR (126 MHz, DMSO-*d*$_6$) δ 169.0, 168.4, 136.6, 131.1, 129.0, 128.5, 127.9, 127.7, 125.5, 123.5, 93.1, 81.5, 62.6, 62.4, 46.0, 32.0, 28.9, 28.4, 27.8.

HRMS (ESI): calculated for $C_{24}H_{26}BNO_4S$ $[M+Na]^+$, 458.1568; Found, 458.1570.

General procedure E

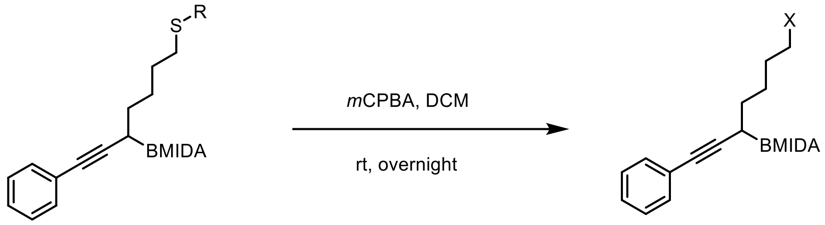

General procedure E$_1$

To the 15 mL Schlenk tube were added starting material (0.1 mmol, 1.0 equiv), *m*CPBA (3-chloroperoxybenzoic acid) (0.1 mmol, 1.0 equiv) and DCM (1 mL). The reaction mixture was vigorously stirred at rt until the reaction was complete as monitored by TLC analysis. The reaction mixture was quenched with water (5 mL) and extracted with EtOAc. The combined organic layers were concentrated in vacuo and the residue was purified by flash chromatography on silica gel with a mixture of Petroleum ether and EtOAc as eluent (eluent = Petroleum ether/EtOAc 1:3 v/v) to afford the pure product.

General procedure E$_2$

To the 15 mL Schlenk tube were added starting material (0.1 mmol, 1.0 equiv), *m*CPBA (3-chloroperoxybenzoic acid) (0.2 mmol, 2.0 equiv) and DCM (1.0 mL). The reaction mixture was vigorously stirred at rt until the reaction was complete as monitored by TLC analysis. The reaction mixture was

quenched with water (5 mL) and extracted with EtOAc. The combined organic layers were concentrated in vacuo and the residue was purified by flash chromatography on silica gel with a mixture of Petroleum ether and EtOAc as eluent (eluent = Petroleum ether/EtOAc 1:3 v/v) to afford the pure product.

6-methyl-2-(1-phenyl-7-(phenylsulfinyl)hept-1-yn-3-yl)-1,3,6,2-dioxazaborocane-4,8-dione

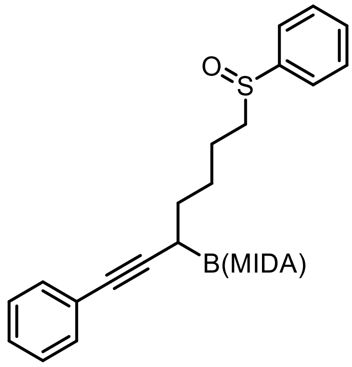

S19

Fellowing the procedure E_1, the product **S19** was obtained in 75% (34 mg) yield as a yellow solid after column chromatography (eluent = ethyl acetate/petroleum ether v/v 2:1), dr = 1:1.

^{1}H NMR (400 MHz, DMSO-*d*$_6$) δ 7.70 – 7.49 (m, 5H), 7.32 (s, 5H), 4.41 (d, *J* = 17.3 Hz, 1H), 4.27 (d, *J* = 16.9 Hz, 1H), 4.04 (d, *J* = 17.4 Hz, 1H), 3.91 (d, *J* = 17.0 Hz, 1H), 3.08 (s, 3H), 3.03 – 2.74 (m, 2H), 2.07 – 1.96 (m, 1H), 1.77 – 1.43 (m, 6H).

^{13}C NMR (101 MHz, DMSO-*d*$_6$) δ 168.9, 168.3, 144.3, 131.1, 130.6, 129.1, 128.5, 127.7, 124.0, 123.5, 93.0, 81.5, 62.6, 62.4, 55.5, 46.0, 29.1, 27.7, 21.5.

HRMS (ESI): calculated for $C_{24}H_{26}BNO_5S$ $[M+Na]^+$, 474.1517; Found, 474.1517.

2-(7-(benzo[d]thiazol-2-ylsulfinyl)-1-phenylhept-1-yn-3-yl)-6-methyl-1,3,6,2-dioxazaborocane-4,8-dione

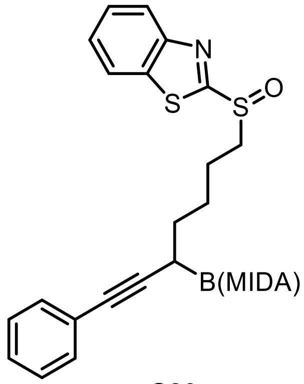

S20

Fellowing the procedure E_1, the product **S20** was obtained in 56% (28 mg) yield as a yellow solid after

column chromatography (eluent = ethyl acetate/petroleum ether v/v 2:1), dr = 1:1.

^{1}H NMR (400 MHz, DMSO-*d$_6$*) δ 8.25 (d, *J* = 8.0 Hz, 1H), 8.11 (d, *J* = 8.1 Hz, 1H), 7.64 – 7.54 (m, 2H), 7.30 (s, 5H), 4.40 (d, *J* = 17.3 Hz, 1H), 4.27 (d, *J* = 16.9 Hz, 1H), 4.04 (d, *J* = 17.4 Hz, 1H), 3.91 (d, *J* = 16.9 Hz, 1H), 3.30 – 3.19 (m, 2H), 3.07 (s, 3H), 2.03 (dd, *J* = 10.1, 3.7 Hz, 1H), 1.96 – 1.51 (m, 6H).

^{13}C NMR (126 MHz, Chloroform-*d*) δ 177.6, 177.5, 167.2, 166.7, 154.0, 154.0, 136.1, 136.1, 131.5, 128.6, 128.2, 127.1, 126.3, 124.1, 123.2, 122.4, 91.4, 91.4, 83.1, 83.1, 63.0, 56.6, 56.5, 45.5, 29.1, 29.0, 28.1, 28.0, 21.5, 21.3.

HRMS (ESI): calculated for $C_{25}H_{25}BN_2O_5S_2$ [M+Na]$^+$, 531.1190; Found, 531.1186.

6-methyl-2-(1-phenyl-7-(phenylsulfonyl)hept-1-yn-3-yl)-1,3,6,2-dioxazaborocane-4,8-dione

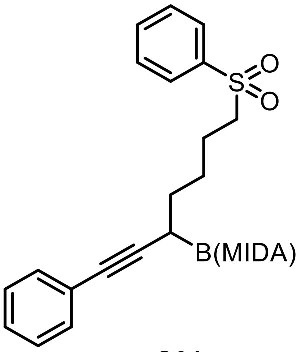

S21

Fellowing the procedure E$_2$, the product **S21** was obtained in 57% (27 mg) yield as a yellow solid after column chromatography (eluent = ethyl acetate/petroleum ether v/v 2:1).

^{1}H NMR (400 MHz, DMSO-*d$_6$*) δ 7.93 – 7.84 (m, 2H), 7.76 – 7.70 (m, 1H), 7.64 (dd, *J* = 8.3, 6.8 Hz, 2H), 7.31 (s, 5H), 4.41 (d, *J* = 17.4 Hz, 1H), 4.27 (d, *J* = 16.9 Hz, 1H), 4.08 – 4.01 (m, 1H), 3.91 (d, *J* = 16.9 Hz, 1H), 3.42 – 3.36 (m, 2H), 3.07 (s, 3H), 2.02 – 1.95 (m, 1H), 1.70 – 1.42 (m, 6H).

^{13}C NMR (126 MHz, DMSO-*d$_6$*) δ 169.0, 168.3, 139.2, 133.7, 131.2, 129.4, 128.5, 127.8, 127.7, 123.5, 92.9, 81.6, 62.6, 62.4, 54.6, 46.0, 28.9, 27.3, 22.3.

HRMS (ESI): calculated for $C_{24}H_{26}BNO_6S$ [M+Na]$^+$, 490.1466; Found, 490.1462.

2-(7-(benzo[d]thiazol-2-ylsulfonyl)-1-phenylhept-1-yn-3-yl)-6-methyl-1,3,6,2-dioxazaborocane-4,8-dione

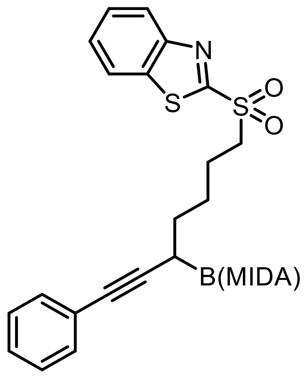

S23

Fellowing the procedure E_1, the product **S23** was obtained in 63% (33 mg) yield as a yellow solid after column chromatography (eluent = ethyl acetate/petroleum ether v/v 2:1).

^{1}H NMR (400 MHz, DMSO-*d$_6$*) δ 8.35 – 8.25 (m, 2H), 7.75 – 7.67 (m, 2H), 7.33 – 7.28 (m, 6H), 4.40 (d, *J* = 17.3 Hz, 1H), 4.26 (d, *J* = 17.0 Hz, 1H), 4.04 (d, *J* = 17.0 Hz, 1H), 3.91 (d, *J* = 17.0 Hz, 1H), 3.79 – 3.72 (m, 2H), 3.06 (s, 3H), 2.01 (dd, *J* = 10.5, 3.7 Hz, 1H), 1.83 – 1.76 (m, 2H), 1.63 – 1.44 (m, 4H).

^{13}C NMR (126 MHz, DMSO-*d$_6$*) δ 168.9, 168.3, 166.4, 152.2, 136.2, 131.1, 128.5, 128.1, 128.0, 127.8, 124.9, 123.5, 123.4, 92.9, 81.6, 62.6, 62.4.

HRMS (ESI): calculated for $C_{25}H_{25}BN_2O_6S_2$ $[M+Na]^+$, 547.1139; Found, 547.1136.

General procedure F

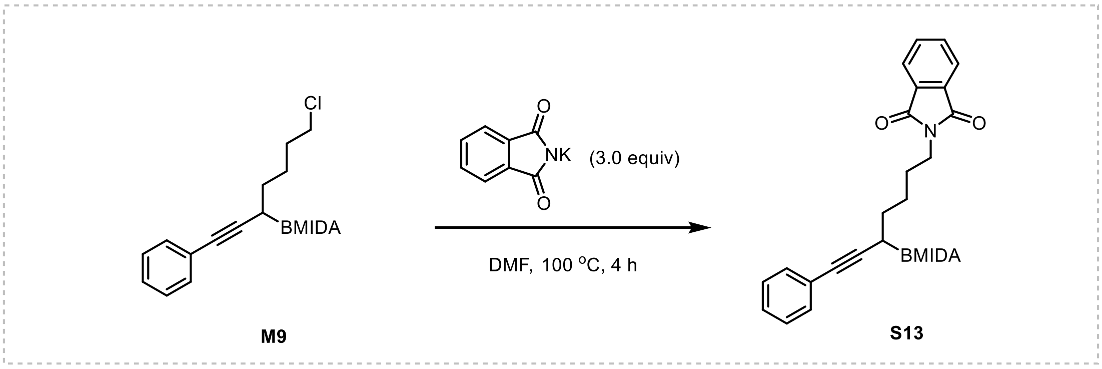

To the 15 mL Schlenk tube were added **M9** (0.1 mmol, 1.0 equiv), potassium phthalimide (0.3 mmol, 3.0 equiv) and DMF (1 mL). The reaction mixture was vigorously stirred at 100 °C until the reaction was complete as monitored by TLC analysis. The reaction mixture was quenched with water (5 mL) and extracted with EtOAc. The combined organic layers were concentrated in vacuo and the residue was purified by flash chromatography on silica gel with a mixture of Petroleum ether and EtOAc as eluent (eluent = Petroleum ether/EtOAc 1:3 v/v) to afford the pure product.

2-(7-(1,3-dioxoisoindolin-2-yl)-1-phenylhept-1-yn-3-yl)-6-methyl-1,3,6,2-dioxazaborocane-4,8-dione

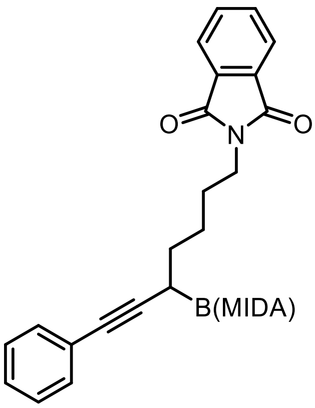

S13

The product **S13** was obtained in 16% (8 mg) yield as a yellow solid after column chromatography (eluent = ethyl acetate/petroleum ether v/v 2:1).

^{1}H NMR (400 MHz, Chloroform-*d*) δ 7.74 (dd, *J* = 5.4, 3.1 Hz, 2H), 7.61 (dd, *J* = 5.4, 3.1 Hz, 2H), 7.19 (s, 5H), 3.98 (d, *J* = 15.9 Hz, 1H), 3.84 – 3.73 (m, 3H), 3.64 (t, *J* = 6.8 Hz, 2H), 3.09 (s, 3H), 1.93 (t, *J* = 6.3 Hz, 1H), 1.74 – 1.61 (m, 6H).

^{13}C NMR (126 MHz, Chloroform-*d*) δ 168.6, 167.1, 166.6, 134.0, 132.3, 131.5, 128.6, 128.1, 123.4, 123.3, 91.7, 83.0, 63.0, 45.5, 38.0, 29.0, 28.3, 26.4.

HRMS (ESI): calculated for $C_{26}H_{25}BN_2O_6$ $[M+Na]^+$, 495.1698; Found, 495.1697.

3. General procedure for the synthesis of allenyl boranes

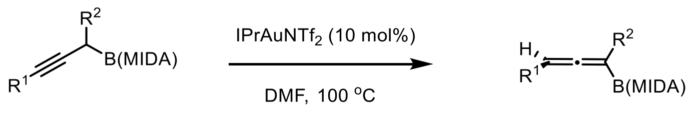

In a glove box, to a 5 mL Schleck tube equipped with a stirrer bar, were added the starting material (0.1 mmol, 1.0 equiv), $IPrAuNTf_2$ (0.01 mmol, 10 mol%, 8.6 mg) and DMF (1 mL). The tube was sealed and heated at 100 ºC with stirring until total consumption of the starting material, as monitored by TLC. The reaction mixture was diluted with EA (20 mL) and water (10 mL). The organic phase was separated and the aqueous layer was extracted with EA (20 mL) for three times. The combined organic layer was dried over anhydrous Na_2SO_4 and concentrated under reduced pressure. The crude residue was purified by flash column (EA/PE as the eluent) to afford the products.

(*R*)-6-methyl-2-(1-phenylocta-1,2-dien-3-yl)-1,3,6,2-dioxazaborocane-4,8-dione

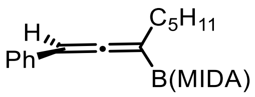

1

The product **1** was obtained in 80% (27 mg) yield as a colorless semisolid after column chromatography (eluent = ethyl acetate/petroleum ether v/v 2:1).

^{1}H NMR (400 MHz, DMSO-*d*$_6$) δ 7.32 – 7.26 (m, 2H), 7.23 – 7.20 (m, 2H), 7.14 (td, *J* = 7.1, 1.4 Hz, 1H), 6.10 (t, *J* = 3.3 Hz, 1H), 4.26 (dd, *J* = 17.2, 15.4 Hz, 2H), 4.02 (d, *J* = 17.1 Hz, 1H), 3.94 (d, *J* = 17.2 Hz, 1H), 2.89 (s, 3H), 2.00 (qd, *J* = 6.7, 3.3 Hz, 2H), 1.46 – 1.40 (m, 2H), 1.31 – 1.23 (m, 4H), 0.80 (t, *J* = 7.1 Hz, 3H).

^{13}C NMR (101 MHz, DMSO-*d*$_6$) δ 207.1, 168.8, 168.5, 135.2, 128.4, 125.9, 125.7, 91.6, 61.4, 61.3, 46.5, 30.9, 28.7, 27.8, 21.8, 13.8.

HRMS (ESI): calculated for $C_{19}H_{24}BNO_4$ [M+Na]$^+$, 364.1691; Found, 364.1687.

(*R*)-6-methyl-2-(1-phenylhepta-1,2-dien-3-yl)-1,3,6,2-dioxazaborocane-4,8-dione

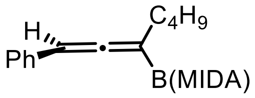

2

The product **2** was obtained in 92% (30 mg) yield as a colorless semisolid after column chromatography (eluent = ethyl acetate/petroleum ether v/v 2:1).

^{1}H NMR (400 MHz, DMSO-*d*$_6$) δ 7.29 (t, *J* = 7.6 Hz, 2H), 7.24 – 7.20 (m, 2H), 7.18 – 7.12 (m, 1H), 6.11 (t, *J* = 3.3 Hz, 1H), 4.26 (dd, *J* = 17.2, 14.7 Hz, 2H), 4.02 (d, *J* = 17.1 Hz, 1H), 3.94 (d, *J* = 17.2 Hz, 1H), 2.90 (s, 3H), 2.05 – 1.98 (m, 2H), 1.46 – 1.39 (m, 2H), 1.36 – 1.29 (m, 2H), 0.84 (t, *J* = 7.2 Hz, 3H).

^{13}C NMR (126 MHz, DMSO-*d*$_6$) δ 207.3, 169.0, 168.7, 135.5, 128.7, 126.1, 125.9, 91.8, 61.6, 61.6, 46.7, 30.7, 28.7, 22.1, 14.0.

HRMS (ESI): calculated for $C_{18}H_{22}BNO_4$ [M+Na]$^+$, 350.1534; Found, 350.1532.

(*R*)-2-(1,6-diphenylhexa-1,2-dien-3-yl)-6-methyl-1,3,6,2-dioxazaborocane-4,8-dione

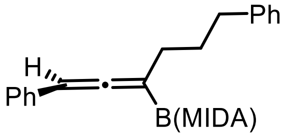

3

The product **3** was obtained in 86% (33 mg) yield as a colorless semisolid after column chromatography

(eluent = ethyl acetate/petroleum ether v/v 2:1).

^{1}H NMR (400 MHz, Chloroform-*d*) δ 7.19 – 7.03 (m, 10H), 5.99 (t, *J* = 3.3 Hz, 1H), 3.77 – 3.69 (m, 2H), 3.49 (d, *J* = 16.8 Hz, 1H), 3.39 (d, *J* = 16.8 Hz, 1H), 2.63 (s, 3H), 2.56 (t, *J* = 7.7 Hz, 2H), 2.08 – 1.96 (m, 2H), 1.80 -1.72 (m, 2H).

^{13}C NMR (101 MHz, DMSO-*d$_6$*) δ 207.3, 168.9, 168.7, 142.2, 135.3, 128.7, 128.2, 128.2, 126.1, 125.9, 125.6, 92.0, 61.6, 61.5, 46.7, 34.9, 30.5, 28.6

HRMS (ESI): calculated for $C_{23}H_{24}BNO_4$ $[M+Na]^+$, 412.1691; Found, 412.1690.

(*R*)-6-methyl-2-(5-methyl-1-phenylhexa-1,2-dien-3-yl)-1,3,6,2-dioxazaborocane-4,8-dione

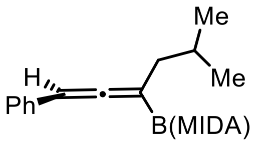

4

The product **4** was obtained in 87% (28 mg) yield as a colorless semisolid after column chromatography (eluent = ethyl acetate/petroleum ether v/v 2:1).

^{1}H NMR (400 MHz, Chloroform-*d*) δ 7.23 – 7.17 (m, 2H), 7.17 – 7.13 (m, 2H), 7.11 – 7.04 (m, 1H), 5.97 (t, *J* = 3.1 Hz, 1H), 3.75 (dd, *J* = 16.7, 1.9 Hz, 2H), 3.54 (d, *J* = 16.6 Hz, 1H), 3.45 (d, *J* = 16.7 Hz, 1H), 2.73 (s, 3H), 1.95 – 1.89 (m, 1H), 1.86 – 1.75 (m, 2H), 0.86 (dd, *J* = 6.4, 3.3 Hz, 6H).

^{13}C NMR (126 MHz, Chloroform-*d*) δ 208.3, 168.1, 168.1, 135.1, 128.9, 126.7, 126.5, 92.6, 61.8, 61.7, 46.5, 39.3, 27.9, 22.9, 22.9.

HRMS (ESI): calculated for $C_{18}H_{22}BNO_4$ $[M+Na]^+$, 350.1534; Found, 350.1532.

(*R*)-2-(5,5-dimethyl-1-phenylhexa-1,2-dien-3-yl)-6-methyl-1,3,6,2-dioxazaborocane-4,8-dione

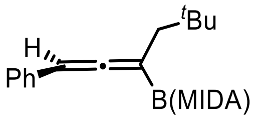

5

The product **5** was obtained in 91% (31 mg) yield as a colorless semisolid after column chromatography (eluent = ethyl acetate/petroleum ether v/v 2:1).

^{1}H NMR (500 MHz, DMSO-*d$_6$*) δ 7.30 (t, *J* = 7.6 Hz, 2H), 7.22 (d, *J* = 7.6 Hz, 2H), 7.14 (t, *J* = 7.3 Hz, 1H), 6.09 (t, *J* = 2.8 Hz, 1H), 4.27 (d, *J* = 17.2 Hz, 1H), 4.20 (d, *J* = 17.0 Hz, 1H), 3.98 (d, *J* = 17.0 Hz, 1H), 3.92 (d, *J* = 17.2 Hz, 1H), 2.89 (s, 3H), 1.95 (d, *J* = 2.8 Hz, 2H), 0.93 (s, 9H).

^{13}C NMR (126 MHz, DMSO-*d*$_6$) δ 208.1, 169.1, 168.6, 135.4, 128.6, 126.1, 126.0, 90.9, 61.6, 61.6, 46.6, 42.3, 31.9, 29.3.

HRMS (ESI): calculated for $C_{19}H_{24}BNO_4$ [M+Na]$^+$, 364.1691; Found, 364.1691.

6-methyl-2-((2*R*)-5,7,7-trimethyl-1-phenylocta-1,2-dien-3-yl)-1,3,6,2-dioxazaborocane-4,8-dione

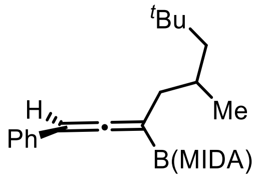

6

The product **6** was obtained in 89% (34 mg) yield as a colorless semisolid after column chromatography (eluent = ethyl acetate/petroleum ether v/v 2:1), dr = 1:1.

^{1}H NMR (500 MHz, Acetone-*d*$_6$) δ 7.33 – 7.24 (m, 4H), 7.15 – 7.13 (m, 1H), 6.12 (dt, *J* = 8.6, 2.9 Hz, 1H), 4.23 (dd, *J* = 16.9, 4.7 Hz, 2H), 4.05 (dd, *J* = 17.0, 1.7 Hz, 1H), 3.96 (dd, *J* = 17.0, 1.7 Hz, 1H), 3.16 (d, *J* = 2.0 Hz, 3H), 2.27 – 2.10 (m, 1H), 1.88 (td, *J* = 8.5, 4.2 Hz, 1H), 1.45 – 1.27 (m, 2H), 1.09 – 1.00 (m, 1H), 0.99 (dd, *J* = 6.4, 4.3 Hz, 3H), 0.88 (d, *J* = 22.4 Hz, 9H).

^{13}C NMR (126 MHz, Acetone-*d*$_6$) δ 209.3, 209.0, 168.8, 168.8, 168.5, 168.5, 136.6, 136.5, 129.3, 127.1, 127.1, 126.9, 92.6, 92.5, 62.6, 62.4, 51.8, 51.7, 47.3, 40.9, 40.9, 31.7, 31.6, 30.5, 30.4, 23.3, 23.2.

HRMS (ESI): calculated for $C_{22}H_{30}BNO_4$ [M+Na]$^+$, 406.2160; Found, 406.2158.

(*R*)-6-methyl-2-(1-phenyl-5-(4-(trifluoromethyl)phenyl)penta-1,2-dien-3-yl)-1,3,6,2-dioxazaboro cane-4,8-dione

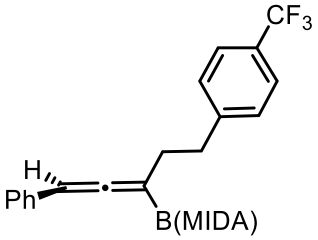

7

The product **7** was obtained in 93% (41 mg) yield as a colorless semisolid after column chromatography (eluent = ethyl acetate/petroleum ether v/v 2:1).

^{1}H NMR (500 MHz, DMSO-*d*$_6$) δ 7.56 (d, *J* = 7.9 Hz, 2H), 7.44 (d, *J* = 7.9 Hz, 2H), 7.24 (t, *J* = 7.2 Hz, 2H), 7.13 (dt, *J* = 12.9, 5.1 Hz, 3H), 6.13 (t, *J* = 3.4 Hz, 1H), 4.27 (t, *J* = 17.7 Hz, 2H), 4.04 (d, *J* = 17.6 Hz, 1H), 3.95 (d, *J* = 17.2 Hz, 1H), 2.91 (s, 3H), 2.87 – 2.80 (m, 2H), 2.47 – 2.31 (m, 2H).

^{13}C NMR (126 MHz, DMSO-*d$_6$*) δ 207.5, 169.0, 168.7, 146.8, 135.1, 129.3, 128.6, 126.6 (q, *J* = 31.6 Hz), 126.2, 126.1, 125.0 (q, *J* = 3.8 Hz), 124.5 (q, *J* = 272.1 Hz), 92.4, 61.7, 61.6, 46.8, 34.1, 30.5.

HRMS (ESI): calculated for $C_{23}H_{21}BF_3NO_4$ $[M+Na]^+$, 466.1408; Found, 466.1411.

(*R*)-2-(5-(3,4-dimethoxyphenyl)-1-phenylpenta-1,2-dien-3-yl)-6-methyl-1,3,6,2-dioxazaborocane-4,8-dione

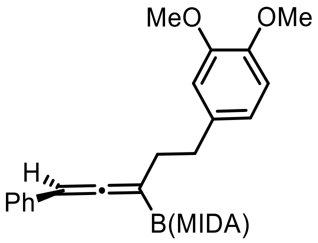

8

The product **8** was obtained in 77% (33 mg) yield as a colorless semisolid after column chromatography (eluent = ethyl acetate/petroleum ether v/v 2:1).

^{1}H NMR (400 MHz, Acetone-*d$_6$*) δ 7.28 – 7.20 (m, 4H), 7.16 – 7.10 (m, 1H), 6.86 – 6.73 (m, 3H), 6.13 (t, *J* = 3.3 Hz, 1H), 4.23 (d, *J* = 17.4 Hz, 2H), 4.04 (d, *J* = 16.9 Hz, 1H), 3.95 (d, *J* = 17.0 Hz, 1H), 3.74 (d, *J* = 4.7 Hz, 6H), 3.14 (s, 3H), 2.81 – 2.76 (m, 2H), 2.54 – 2.34 (m, 2H).

^{13}C NMR (101 MHz, Acetone-*d$_6$*) δ 208.9, 168.8, 168.6, 150.3, 148.6, 136.5, 135.8, 129.4, 127.1, 127.0, 121.2, 113.7, 113.0, 93.4, 62.6, 62.5, 56.2, 56.0, 47.4, 35.4, 32.8.

HRMS (ESI): calculated for $C_{24}H_{26}BNO_6$ $[M+Na]^+$, 458.1745; Found, 458.1743.

(*R*)-2-(5-(4-chlorophenyl)-1-phenylpenta-1,2-dien-3-yl)-6-methyl-1,3,6,2-dioxazaborocane-4,8-dione

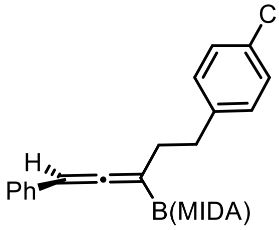

9

The product **9** was obtained in 84% (34 mg) yield as a colorless semisolid after column chromatography (eluent = ethyl acetate/petroleum ether v/v 2:1).

^{1}H NMR (400 MHz, Chloroform-*d*) δ 7.19 – 7.14 (m, 3H), 7.13 – 7.09 (m, 2H), 7.05 – 7.00 (m, 4H), 5.98 (t, *J* = 3.3 Hz, 1H), 3.73 (dd, *J* = 16.7, 2.4 Hz, 2H), 3.49 (d, *J* = 16.6 Hz, 1H), 3.39 (d, *J* = 16.7 Hz, 2H), 2.75 – 2.70 (m, 3H), 2.64 (s, 2H), 2.40 – 2.20 (m, 2H).

^{13}C NMR (101 MHz, Chloroform-*d*) δ 208.2, 167.8, 167.8, 140.4, 134.7, 131.7, 130.1, 128.9, 128.5, 126.9, 126.5, 93.7, 61.8, 61.7, 46.5, 34.2, 31.6.

HRMS (ESI): calculated for $C_{22}H_{21}BClNO_4$ [M+Na]$^+$, 432.1144; Found, 432.1143.

(*R*)-2-(1,5-diphenylpenta-1,2-dien-3-yl)-6-methyl-1,3,6,2-dioxazaborocane-4,8-dione

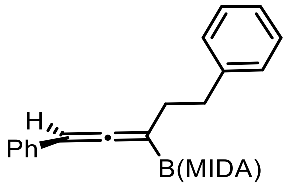

10

The product **10** was obtained in 77% (29 mg) yield as a colorless semisolid after column chromatography (eluent = ethyl acetate/petroleum ether v/v 2:1).

^{1}H NMR (400 MHz, DMSO-*d*$_6$) δ 7.31 – 7.12 (m, 10H), 6.14 (t, *J* = 3.3 Hz, 1H), 4.26 (dd, *J* = 17.2, 12.4 Hz, 2H), 4.03 (d, *J* = 17.1 Hz, 1H), 3.93 (d, *J* = 17.2 Hz, 1H), 2.89 (s, 3H), 2.76 – 2.71 (m, 2H), 2.40 – 2.24 (m, 2H).

^{13}C NMR (101 MHz, DMSO-*d*$_6$) δ 207.4, 168.9, 168.7, 141.9, 135.2, 128.6, 128.4, 128.2, 126.1, 126.0, 125.7, 92.23, 61.6, 61.6, 46.7, 34.5, 31.1.

HRMS (ESI): calculated for $C_{22}H_{22}BNO_4$ [M+Na]$^+$, 398.1534; Found, 398.1532.

2-(4,4,4,4,4,4-hexamethyl-1-phenyl-4l8-hepta-1,2,6-trien-3-yl)-6-methyl-1,3,6,2-dioxazaborocane-4,8-dione

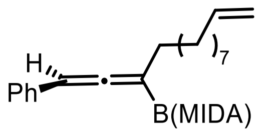

11

The product **11** was obtained in 89% (36 mg) yield as a colorless semisolid after column chromatography (eluent = ethyl acetate/petroleum ether v/v 2:1).

^{1}H NMR (400 MHz, DMSO-*d*$_6$) δ 7.28 (t, *J* = 7.5 Hz, 2H), 7.23 – 7.19 (m, 2H), 7.16 – 7.11 (m, 1H), 6.10 (t, *J* = 3.3 Hz, 1H), 5.81 – 5.70 (m, 1H), 4.99 – 4.88 (m, 2H), 4.26 (dd, *J* = 17.2, 15.1 Hz, 2H), 4.02 (d, *J* = 17.1 Hz, 1H), 3.94 (d, *J* = 17.3 Hz, 1H), 2.90 (s, 3H), 2.05 – 1.93 (m, 4H), 1.44 – 1.41 (m, 2H), 1.31 – 1.16 (m, 10H).

^{13}C NMR (126 MHz, DMSO-*d*$_6$) δ 207.3, 169.0, 168.7, 138.8, 135.4, 128.6, 126.1, 125.9, 114.6, 91.8, 61.6, 61.6, 46.7, 33.2, 29.0, 28.9, 28.9, 28.5, 28.4, 28.3.

HRMS (ESI): calculated for $C_{24}H_{30}BNO_4$ [M+Na]$^+$, 432.2317; Found, 432.2320.

(*R*)-2-(7-azido-1-phenylhepta-1,2-dien-3-yl)-6-methyl-1,3,6,2-dioxazaborocane-4,8-dione

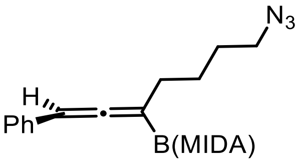

12

The product **12** was obtained in 85% (31 mg) yield as a colorless semisolid after column chromatography (eluent = ethyl acetate/petroleum ether v/v 2:1).

^{1}H NMR (400 MHz, Chloroform-*d*) δ 7.29 – 7.07 (m, 5H), 6.02 (t, *J* = 3.2 Hz, 1H), 3.75 (dd, *J* = 16.6, 6.1 Hz, 2H), 3.52 (dd, *J* = 39.7, 16.6 Hz, 2H), 3.17 (t, *J* = 6.4 Hz, 2H), 2.77 (s, 3H), 2.13 – 1.95 (m, 2H), 1.67 – 1.53 (m, 4H).

^{13}C NMR (126 MHz, Chloroform-*d*) δ 208.0, 167.8, 167.7, 134.8, 129.0, 126.9, 126.4, 93.4, 61.8, 61.7, 51.3, 46.6, 29.5, 28.7, 26.0.

HRMS (ESI): calculated for $C_{18}H_{21}BN_4O_4$ [M+Na]$^+$, 391.1548; Found, 391.1548.

(*R*)-2-(7-(1,3-dioxoisoindolin-2-yl)-1-phenylhepta-1,2-dien-3-yl)-6-methyl-1,3,6,2-dioxazaborocane-4,8-dione

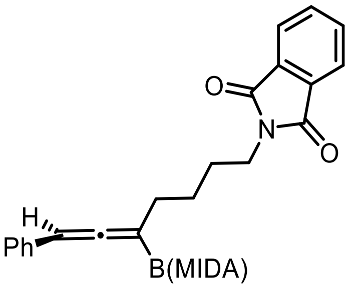

13

The product **13** was obtained in 73% (34 mg) yield as a colorless semisolid after column chromatography (eluent = ethyl acetate/petroleum ether v/v 2:1).

^{1}H NMR (400 MHz, DMSO-*d*$_6$) δ 7.83 – 7.80 (m, 4H), 7.22 (t, *J* = 7.5 Hz, 2H), 7.16 – 7.12 (m, 2H), 7.11 – 7.06 (m, 1H), 6.06 (t, *J* = 3.3 Hz, 1H), 4.26 (dd, *J* = 17.2, 15.3 Hz, 2H), 4.02 (d, *J* = 17.1 Hz, 1H), 3.92 (d, *J* = 17.2 Hz, 1H), 3.55 (t, *J* = 7.1 Hz, 2H), 2.90 (s, 3H), 2.16 – 2.00 (m, 2H), 1.71 – 1.57 (m, 2H), 1.49 – 1.41 (m, 2H).

^{13}C NMR (126 MHz, DMSO-*d*$_6$) δ 207.2, 169.0, 168.7, 167.9, 135.2, 134.3, 131.5, 128.6, 126.1, 125.9, 123.0, 92.0, 61.6, 61.5, 46.7, 37.2, 28.3, 27.7, 25.5.

HRMS (ESI): calculated for $C_{26}H_{25}BN_2O_6$ $[M+Na]^+$, 495.1698; Found, 495.1698.

(*R*)-6-methyl-2-(5-phenoxy-1-phenylpenta-1,2-dien-3-yl)-1,3,6,2-dioxazaborocane-4,8-dione

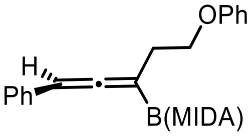

14

The product **14** was obtained in 61% (24 mg) yield as a colorless semisolid after column chromatography (eluent = ethyl acetate/petroleum ether v/v 2:1).

^{1}H NMR (400 MHz, Chloroform-*d*) δ 7.21 – 7.11 (m, 6H), 7.08 – 7.03 (m, 1H), 6.84 (t, *J* = 7.3 Hz, 1H), 6.76 (d, *J* = 7.5 Hz, 2H), 6.03 (t, *J* = 2.9 Hz, 1H), 4.06 (t, *J* = 6.3 Hz, 2H), 3.75 (dd, *J* = 16.7, 9.4 Hz, 2H), 3.57 (d, *J* = 16.7 Hz, 1H), 3.45 (d, *J* = 16.6 Hz, 1H), 2.77 (s, 3H), 2.51 – 2.46 (m, 2H).

^{13}C NMR (126 MHz, Chloroform-*d*) δ 208.9, 168.0, 167.9, 158.7, 134.5, 129.7, 128.9, 126.9, 126.6, 121.0, 114.6, 93.4, 67.0, 61.9, 61.8, 46.7, 29.7.

HRMS (ESI): calculated for $C_{22}H_{22}BNO_5$ $[M+Na]^+$, 414.1483; Found, 414.1483.

(*R*)-2-(5-(benzyloxy)-1-phenylpenta-1,2-dien-3-yl)-6-methyl-1,3,6,2-dioxazaborocane-4,8-dione

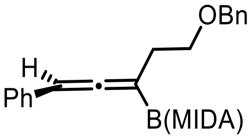

15

The product **15** was obtained in 63% (26 mg) yield as a colorless semisolid after column chromatography (eluent = ethyl acetate/petroleum ether v/v 2:1).

^{1}H NMR (500 MHz, Chloroform-*d*) δ 7.27 – 7.17 (m, 6H), 7.14 (d, *J* = 5.7 Hz, 4H), 5.99 (t, *J* = 3.0 Hz, 1H), 4.37 (d, *J* = 3.1 Hz, 1H), 3.69 – 3.58 (m, 4H), 3.51 (d, *J* = 16.6 Hz, 1H), 3.38 (d, *J* = 16.6 Hz, 1H), 2.67 (s, 3H), 2.31 (tt, *J* = 6.0, 2.5 Hz, 2H).

^{13}C NMR (101 MHz, DMSO-d_6) δ 207.4, 168.7, 168.5, 138.3, 134.9, 128.4, 127.9, 127.2, 127.1, 125.9, 125.9, 91.5, 71.7, 68.9, 61.4, 46.5, 29.1.

HRMS (ESI): calculated for $C_{23}H_{24}BNO_5$ $[M+Na]^+$, 428.1640; Found, 428.1636.

(*R*)-4-(6-methyl-4,8-dioxo-1,3,6,2-dioxazaborocan-2-yl)-6-phenylhexa-4,5-dien-1-yl formate

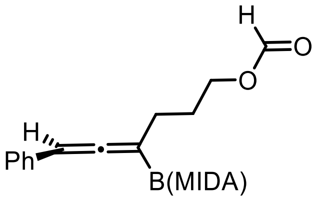

16

The product **16** was obtained in 83% (30 mg) yield as a colorless semisolid after column chromatography (eluent = ethyl acetate/petroleum ether v/v 2:1).

^{1}H NMR (400 MHz, Acetone-*d₆*) δ 8.09 (s, 1H), 7.32 – 7.24 (m, 4H), 7.17 – 7.12 (m, 1H), 6.17 (t, *J* = 3.4 Hz, 1H), 4.28 – 4.15 (m, 4H), 4.07 (d, *J* = 16.9 Hz, 1H), 3.98 (d, *J* = 17.0 Hz, 1H), 3.18 (s, 3H), 2.32 – 2.15(m, 2H), 1.95 – 1.84 (m, 2H).

^{13}C NMR (101 MHz, Acetone-*d₆*) δ 208.7, 168.8, 168.6, 162.0, 136.3, 129.5, 127.1, 127.1, 93.66, 63.9, 62.6, 62.5, 47.4, 28.6, 26.6.

HRMS (ESI): calculated for $C_{19}H_{22}BNO_6$ [M+Na]$^+$, 380.1276; Found, 380.1276.

(*R*)-4-(6-methyl-4,8-dioxo-1,3,6,2-dioxazaborocan-2-yl)-6-phenylhexa-4,5-dien-1-yl benzoate

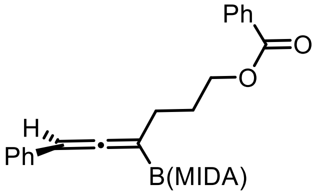

17

The product **17** was obtained in 81% (35 mg) yield as a colorless semisolid after column chromatography (eluent = ethyl acetate/petroleum ether v/v 2:1).

^{1}H NMR (400 MHz, Acetone-*d₆*) δ 8.01 (d, *J* = 8.1 Hz, 2H), 7.63 – 7.58 (m, 1H), 7.48 (t, *J* = 7.7 Hz, 2H), 7.33 – 7.23 (m, 4H), 7.17 – 7.12 (m, 1H), 6.19 (t, *J* = 3.4 Hz, 1H), 4.38 (td, *J* = 6.5, 2.5 Hz, 2H), 4.25 (dd, *J* = 16.9, 1.5 Hz, 2H), 4.06 (d, *J* = 16.9 Hz, 1H), 3.97 (d, *J* = 17.0 Hz, 1H), 3.19 (s, 3H), 2.39 – 2.30 (m, 2H), 2.02 (t, *J* = 3.3 Hz, 2H).

^{13}C NMR (126 MHz, Chloroform-*d*) δ 208.0, 168.0, 168.0, 166.8, 134.8, 133.0, 130.3, 129.6, 129.0, 128.5, 126.9, 126.4, 93.6, 64.6, 61.8, 61.7, 46.5, 28.0, 26.3.

HRMS (ESI): calculated for $C_{24}H_{24}BNO_6$ [M+Na]$^+$, 456.1589; Found, 456.1593.

(*R*)-6-methyl-2-(1-phenyl-7-(phenylthio)hepta-1,2-dien-3-yl)-1,3,6,2-dioxazaborocane-4,8-dione

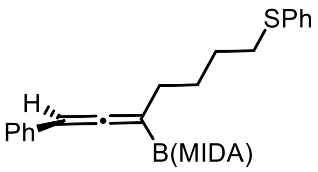

18

The product **18** was obtained in 50% (22 mg) yield as a colorless semisolid after column chromatography (eluent = ethyl acetate/petroleum ether v/v 2:1).

^{1}H NMR (400 MHz, DMSO-*d$_6$*) δ 7.34 – 7.24 (m, 6H), 7.23 – 7.17 (m, 2H), 7.15 (td, *J* = 7.3, 6.5, 2.8 Hz, 2H), 6.10 (t, *J* = 3.4 Hz, 1H), 4.26 (dd, *J* = 17.2, 14.3 Hz, 2H), 4.02 (d, *J* = 17.1 Hz, 1H), 3.93 (d, *J* = 17.2 Hz, 1H), 2.95 – 2.87 (m, 5H), 2.06 – 1.99 (m, 2H), 1.70 – 1.50 (m, 4H).

^{13}C NMR (101 MHz, DMSO-*d$_6$*) δ 207.2, 168.9, 168.7, 136.5, 135.4, 129.0, 128.7, 127.9, 126.1, 125.9, 125.4, 92.0, 61.6, 61.6, 46.7, 31.8, 28.5, 28.5, 27.5.

HRMS (ESI): calculated for $C_{24}H_{26}BNO_4S$ $[M+Na]^+$, 458.1568; Found, 458.1565.

6-methyl-2-((2*R*)-1-phenyl-7-(phenylsulfinyl)hepta-1,2-dien-3-yl)-1,3,6,2-dioxazaborocane-4,8-dione

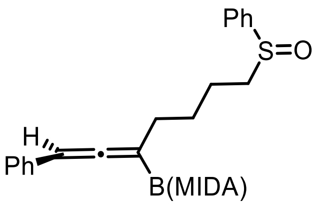

19

The product **19** was obtained in 51% (23 mg) yield as a colorless semisolid after column chromatography (eluent = ethyl acetate/petroleum ether v/v 2:1), dr = 1:1..

^{1}H NMR (400 MHz, Acetone-*d$_6$*) δ 7.68 – 7.59 (m, 2H), 7.54 (q, *J* = 8.3, 7.3 Hz, 3H), 7.27 (d, *J* = 4.4 Hz, 4H), 7.16 – 7.11 (m, 1H), 6.09 (dt, *J* = 8.0, 3.2 Hz, 1H), 4.23 (d, *J* = 17.0 Hz, 2H), 4.05 (d, *J* = 16.8 Hz, 1H), 3.96 (d, *J* = 17.0 Hz, 1H), 3.14 (s, 3H), 2.95 – 2.89 (m, 1H), 2.77 - 2.69 (m, 1H), 2.23 – 2.08 (m, 2H), 1.86 – 1.61 (m, 4H).

^{13}C NMR (126 MHz, Acetone-*d$_6$*) δ 208.8, 168.8, 168.6, 146.0, 136.5, 131.4, 129.9, 129.4, 127.1, 124.8, 124.7, 93.3, 62.6, 62.5, 57.2, 47.4, 28.5, 28.5, 22.4.

HRMS (ESI): calculated for $C_{24}H_{26}BNO_5S$ $[M+Na]^+$, 474.1517; Found, 474.1510.

2-((2*R*)-7-(benzo[d]thiazol-2-ylsulfinyl)-1-phenylhepta-1,2-dien-3-yl)-6-methyl-1,3,6,2-dioxaza

borocane-4,8-dione

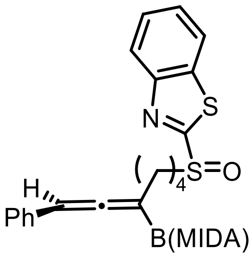

20

The product **20** was obtained in 50% (25 mg) yield as a colorless semisolid after column chromatography (eluent = ethyl acetate/petroleum ether v/v 2:1), dr = 1:1.

^{1}H NMR (400 MHz, Chloroform-*d*) δ 7.99 – 7.87 (m, 2H), 7.50 – 7.37 (m, 2H), 7.21 – 7.15 (m, 3H), 7.12 – 7.07 (m, 2H), 5.95 (dt, *J* = 7.7, 3.2 Hz, 1H), 3.80 – 3.73 (m, 2H), 3.57 (dd, *J* = 16.6, 2.4 Hz, 1H), 3.48 (dd, *J* = 16.6, 1.8 Hz, 1H), 3.19 – 3.05 (m, 2H), 2.76 (d, *J* = 1.9 Hz, 3H), 2.13 – 2.00 (m, 2H), 1.75 – 1.56 (m, 4H).

^{13}C NMR (101 MHz, Chloroform-*d*) δ 208.0, 177.4, 167.7, 167.6, 154.0, 136.0, 134.8, 129.0, 127.1, 126.9, 126.4, 126.3, 124.1, 122.4, 93.5, 61.9, 61.8, 56.4, 46.6, 29.5, 27.7, 21.5.

HRMS (ESI): calculated for $C_{25}H_{25}BN_2O_5S_2$ [M+Na]$^+$, 531.1190; Found, 531.1191.

(*R*)-6-methyl-2-(1-phenyl-7-(phenylsulfonyl)hepta-1,2-dien-3-yl)-1,3,6,2-dioxazaborocane-4,8-dione

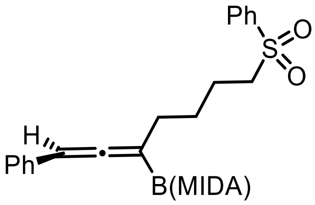

21

The product **21** was obtained in 75% (35 mg) yield as a colorless semisolid after column chromatography (eluent = ethyl acetate/petroleum ether v/v 2:1).

^{1}H NMR (400 MHz, DMSO-*d$_6$*) δ 7.85 (d, *J* = 7.1 Hz, 2H), 7.75 – 7.68 (m, 1H), 7.63 (dd, *J* = 8.4, 7.0 Hz, 2H), 7.28 (t, *J* = 7.6 Hz, 2H), 7.21 – 7.12 (m, 3H), 6.04 (t, *J* = 3.3 Hz, 1H), 4.25 (dd, *J* = 17.2, 14.0 Hz, 2H), 4.02 (d, *J* = 17.1 Hz, 1H), 3.92 (d, *J* = 17.2 Hz, 1H), 3.29 (t, *J* = 7.7 Hz, 2H), 2.87 (s, 3H), 2.06 – 1.92 (m,

2H), 1.64 – 1.41 (m, 4H).

^{13}C NMR (126 MHz, DMSO-*d$_6$*) δ 207.1, 168.9, 168.7, 139.1, 135.3, 133.7, 129.4, 128.7, 127.6, 126.2, 126.0, 92.0, 61.6, 61.6, 54.4, 46.7, 28.3, 26.7, 22.2.

HRMS (ESI): calculated for $C_{24}H_{26}BNO_6S$ $[M+Na]^+$, 490.1466; Found, 490.1469.

(*R*)-6-methyl-2-(1-phenyl-7-tosylhepta-1,2-dien-3-yl)-1,3,6,2-dioxazaborocane-4,8-dione

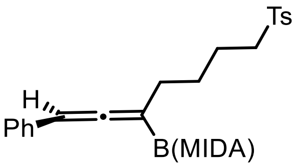

22

The product **22** was obtained in 90% (43 mg) yield as a colorless semisolid after column chromatography (eluent = ethyl acetate/petroleum ether v/v 2:1).

^{1}H NMR (400 MHz, DMSO-*d$_6$*) δ 7.72 (d, *J* = 8.2 Hz, 2H), 7.42 (d, *J* = 8.0 Hz, 2H), 7.28 (t, *J* = 7.6 Hz, 2H), 7.22 – 7.12 (m, 3H), 6.05 (t, *J* = 3.3 Hz, 1H), 4.25 (dd, *J* = 17.2, 12.8 Hz, 2H), 4.01 (d, *J* = 17.1 Hz, 1H), 3.92 (d, *J* = 17.2 Hz, 1H), 3.24 (t, *J* = 7.7 Hz, 2H), 2.87 (s, 3H), 2.39 (s, 3H), 2.04 – 1.93 (m, 2H), 1.61 – 1.44 (m, 4H).

^{13}C NMR (101 MHz, DMSO-*d$_6$*) δ 207.2, 168.9, 168.7, 144.2, 136.2, 135.3, 129.9, 128.8, 127.7, 126.2, 126.0, 92., 61.6, 61.6, 54.6, 46.7, 28.4, 26.7, 22.3, 21.1.

HRMS (ESI): calculated for $C_{25}H_{28}BNO_6S$ $[M+Na]^+$, 504.1623; Found, 504.1615.

(*R*)-2-(7-(benzo[d]thiazol-2-ylsulfonyl)-1-phenylhepta-1,2-dien-3-yl)-6-methyl-1,3,6,2-dioxazaboro cane-4,8-dione

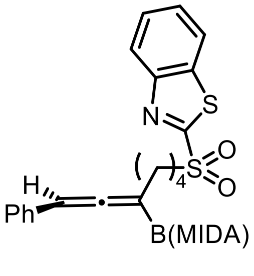

23

The product **23** was obtained in 93% (49 mg) yield as a colorless semisolid after column chromatography (eluent = ethyl acetate/petroleum ether v/v 2:1).

^{1}H NMR (500 MHz, DMSO-*d$_6$*) δ 8.33 (d, *J* = 7.8 Hz, 1H), 8.25 (d, *J* = 7.8 Hz, 1H), 7.68 – 7.74 (m, 2H),

7.23 (q, J = 9.4, 8.5 Hz, 2H), 7.18 – 7.09 (m, 3H), 6.03 (d, J = 3.5 Hz, 1H), 4.26 (t, J = 17.3 Hz, 2H), 4.02 (d, J = 17.5 Hz, 1H), 3.93 (d, J = 17.2 Hz, 1H), 3.70 (t, J = 7.8 Hz, 2H), 2.88 (s, 3H), 2.10 – 1.99 (m, 2H), 1.82 – 1.75 (m, 2H), 1.60 – 1.50 (m, 2H).

^{13}C NMR (126 MHz, DMSO-*d$_6$*) δ 207.1, 168.9, 168.7, 166.3, 152.3, 136.3, 135.2, 128.6, 128.2, 128.0, 126.2, 125.9, 124.9, 123.5, 92.1, 61.6, 61.6, 53.6, 46.7, 28.2, 26.6, 22.0.

HRMS (ESI): calculated for $C_{25}H_{25}BN_2O_6S_2$ [M+Na]$^+$, 547.1139; Found, 547.1145.

(*R*)-2-(1-cyclopropyl-3-phenylpropa-1,2-dien-1-yl)-6-methyl-1,3,6,2-dioxazaborocane-4,8-dione

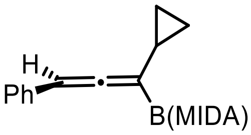

24

The product **24** was obtained in 81% (25 mg) yield as a colorless semisolid after column chromatography (eluent = ethyl acetate/petroleum ether v/v 2:1).

^{1}H NMR (400 MHz, DMSO-*d$_6$*) δ 7.28 (t, J = 7.5 Hz, 2H), 7.21 – 7.11 (m, 3H), 6.16 (s, 1H), 4.31 (dd, J = 17.2, 15.0 Hz, 2H), 4.05 (d, J = 17.1 Hz, 1H), 3.98 (d, J = 17.2 Hz, 1H), 2.97 (s, 3H), 1.20 – 1.13 (m, 1H), 0.77 – 0.71 (m, 2H), 0.36 (dt, J = 5.6, 3.6 Hz, 2H).

^{13}C NMR (101 MHz, DMSO-*d$_6$*) δ 205.4, 169.0, 168.8, 135.1, 128.7, 126.4, 125.9, 93.7, 61.6, 47.1, 9.2, 9.0, 8.7.

HRMS (ESI): calculated for $C_{17}H_{18}BNO_4$ [M+Na]$^+$, 334.1221; Found, 334.1218.

(*R*)-2-(1-cyclobutyl-3-phenylpropa-1,2-dien-1-yl)-6-methyl-1,3,6,2-dioxazaborocane-4,8-dione

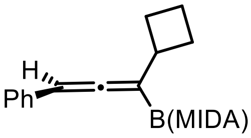

25

The product **25** was obtained in 75% (24 mg) yield as a colorless semisolid after column chromatography (eluent = ethyl acetate/petroleum ether v/v 2:1).

^{1}H NMR (400 MHz, Acetonitrile-*d$_3$*) δ 7.18 (d, J = 5.9 Hz, 4H), 7.06 (td, J = 5.9, 2.8 Hz, 1H), 6.07 (d, J = 2.7 Hz, 1H), 3.84 (dd, J = 17.0, 2.3 Hz, 2H), 3.68 (d, J = 17.0 Hz, 1H), 3.61 (d, J = 17.1 Hz, 1H), 2.76 (s, 3H), 2.10 – 1.96 (m, 3H), 1.85 – 1.77 (m, 4H).

^{13}C NMR (101 MHz, Acetonitrile-*d$_3$*) δ 208.8, 169.1, 168.8, 136.4, 129.7, 127.3, 126.9, 94.1, 62.5, 62.4,

47.7, 36.4, 30.9, 30.0, 18.7.

HRMS (ESI): calculated for $C_{18}H_{20}BNO_4$ $[M+Na]^+$, 348.1378; Found, 348.1373.

(*R*)-2-(1-cyclopentyl-3-phenylpropa-1,2-dien-1-yl)-6-methyl-1,3,6,2-dioxazaborocane-4,8-dione

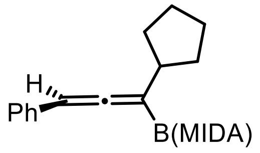

26

The product **26** was obtained in 85% (29 mg) yield as a colorless semisolid after column chromatography (eluent = ethyl acetate/petroleum ether v/v 2:1).

^{1}H NMR (400 MHz, DMSO-*d₆*) δ 7.34 – 7.26 (m, 2H), 7.24 – 7.19 (m, 2H), 7.17 – 7.11 (m, 1H), 6.14 (d, *J* = 2.0 Hz, 1H), 4.32 – 4.20 (m, 2H), 4.01 (d, *J* = 17.1 Hz, 1H), 3.93 (d, *J* = 17.2 Hz, 1H), 2.89 (s, 3H), 2.42 – 2.33 (m, 1H), 1.97 – 1.78 (m, 2H), 1.64 – 1.48 (m, 4H), 1.44 – 1.35 (m, 2H).

^{13}C NMR (101 MHz, DMSO-*d₆*) δ 206.2, 168.4, 168.1, 135.1, 128.3, 125.8, 125.6, 92.6, 61.5, 61.4, 46.6, 33.6, 32.7, 24.4, 24.4.

HRMS (ESI): calculated for $C_{19}H_{22}BNO_4$ $[M+Na]^+$, 362.1534; Found, 362.1535.

(*R*)-2-(1-cyclohexyl-3-phenylpropa-1,2-dien-1-yl)-6-methyl-1,3,6,2-dioxazaborocane-4,8-dione

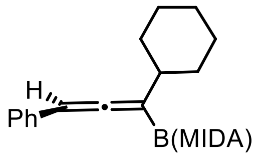

27

The product **27** was obtained in 88% (31 mg) yield as a colorless semisolid after column chromatography (eluent = ethyl acetate/petroleum ether v/v 2:1).

^{1}H NMR (500 MHz, Chloroform-*d*) δ 7.24 – 7.16 (m, 2H), 7.15 (d, J = 7.1 Hz, 2H), 7.07 (t, *J* = 7.2 Hz, 1H), 6.01 (d, *J* = 1.6 Hz, 1H), 3.75 (dd, *J* = 16.8, 9.4 Hz, 2H), 3.55 (d, *J* = 16.7 Hz, 1H), 3.44 (d, *J* = 16.7 Hz, 1H), 2.71 (s, 3H), 1.94 – 1.53 (m, 6H), 1.20 – 1.04 (m, 5H).

^{13}C NMR (126 MHz, Chloroform-*d*) δ 207.8, 168.4, 168.3, 135.1, 129.0, 126.7, 126.2, 93.8, 61.8, 61.7, 46.7, 39.4, 34.7, 33.7, 26.9, 26.8, 26.1.

HRMS (ESI): calculated for $C_{20}H_{24}BNO_4$ $[M+Na]^+$, 376.1691; Found, 376.1692.

(*R*)-6-methyl-2-(3-phenyl-1-(tetrahydro-2H-pyran-4-yl)propa-1,2-dien-1-yl)-1,3,6,2-dioxazaboro cane-4,8-dione

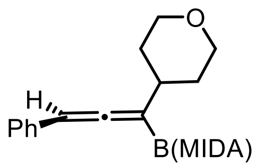

28

The product **28** was obtained in 86% (31 mg) yield as a colorless semisolid after column chromatography (eluent = ethyl acetate/petroleum ether v/v 2:1).

^{1}H NMR (500 MHz, Chloroform-*d*) δ 7.20 (t, *J* = 7.6 Hz, 2H), 7.14 (d, *J* = 7.5 Hz, 2H), 7.09 (t, *J* = 7.3 Hz, 1H), 6.07 (s, 1H), 3.87 (d, *J* = 7.6 Hz, 2H), 3.76 (d, *J* = 16.7 Hz, 2H), 3.54 (d, *J* = 16.6 Hz, 1H), 3.45 (d, *J* = 16.6 Hz, 1H), 3.36 (t, *J* = 11.7 Hz, 2H), 2.74 (s, 3H), 2.14 (t, *J* = 12.1 Hz, 1H), 1.79 (d, *J* = 13.3 Hz, 1H), 1.64 (d, *J* = 13.4 Hz, 1H), 1.57 – 1.46 (m, 2H).

^{13}C NMR (126 MHz, Chloroform-*d*) δ 207.7, 167.8, 167.7, 134.5, 129.0, 127.0, 126.2, 94.5, 68.2, 68.2, 61.8, 61.7, 46.7, 36.4, 34.0, 33.1.

HRMS (ESI): calculated for $C_{19}H_{22}BNO_5$ $[M+Na]^+$, 378.1483; Found, 378.1480.

2-((2*R*)-1-(cyclohex-3-en-1-yl)-3-phenylpropa-1,2-dien-1-yl)-6-methyl-1,3,6,2-dioxazaborocane-4,8-dione

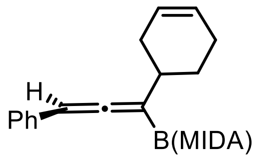

29

The product **29** was obtained in 91% (32 mg) yield as a colorless semisolid after column chromatography (eluent = ethyl acetate/petroleum ether v/v 2:1), dr = 1:1.

^{1}H NMR (400 MHz, Acetone-*d$_6$*) δ 7.28 (q, *J* = 7.9, 7.5 Hz, 8H), 7.14 (t, *J* = 7.5 Hz, 2H), 6.22 – 6.16 (m, 2H), 5.64 (dt, *J* = 6.3, 3.4 Hz, 4H), 4.22 (dd, *J* = 17.0, 3.8 Hz, 4H), 4.10 – 3.88 (m, 4H), 3.16 (d, *J* = 2.3 Hz, 6H), 2.38 – 2.17 (m, 4H), 2.11 (t, *J* = 9.1 Hz, 4H), 2.03 – 1.95 (m, 2H), 1.45 – 1.28 (m, 4H).

^{13}C NMR (126 MHz, Acetone-*d$_6$*) δ 208.6, 208.5, 168.9, 168.8, 168.6, 168.5, 136.4, 136.4, 129.5, 129.4, 127.5, 127.2, 127.1, 127.1, 127.0, 126.9, 126.9, 94.3, 94.2, 62.7, 62.6, 47.5, 47.5, 36.0, 35.9, 33.8, 32.8, 31.1, 30.2, 26.6, 26.6.

HRMS (ESI): calculated for $C_{20}H_{22}BNO_4$ $[M+Na]^+$, 374.1534; Found, 374.1539.

(*R*)-6-methyl-2-(4-methyl-1-phenylpenta-1,2-dien-3-yl)-1,3,6,2-dioxazaborocane-4,8-dione

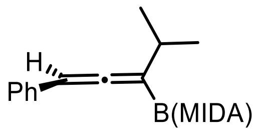

30

The product **30** was obtained in 90% (28 mg) yield as a colorless semisolid after column chromatography (eluent = ethyl acetate/petroleum ether v/v 2:1).

^{1}H NMR (500 MHz, DMSO-*d$_6$*) δ 7.35 – 7.21 (m, 4H), 7.14 (t, *J* = 7.3 Hz, 1H), 6.17 (s, 1H), 4.26 (dd, *J* = 23.6, 17.2 Hz, 2H), 4.01 (d, *J* = 17.1 Hz, 1H), 3.91 (d, *J* = 17.3 Hz, 1H), 2.89 (s, 3H), 2.30 – 2.22 (m, 1H), 1.10 (d, *J* = 6.6 Hz, 3H), 1.05 (d, *J* = 6.6 Hz, 3H).

^{13}C NMR (126 MHz, DMSO-*d$_6$*) δ 206.6, 169.0, 168.7, 135.4, 128.7, 126.2, 125.8, 93.1, 61.6, 61.6, 46.9, 28.8, 24.0, 23.1.

HRMS (ESI): calculated for $C_{17}H_{20}BNO_4$ [M+Na]$^+$, 336.1378; Found, 336.1380.

(*S*)-6-methyl-2-(1-(p-tolyl)octa-1,2-dien-3-yl)-1,3,6,2-dioxazaborocane-4,8-dione

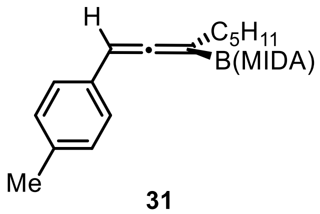

31

The product **31** was obtained in 73% (26 mg) yield as a colorless semisolid after column chromatography (eluent = ethyl acetate/petroleum ether v/v 2:1).

^{1}H NMR (400 MHz, Acetone-*d$_6$*) δ 7.19 – 7.15 (m, 2H), 7.08 (d, *J* = 7.9 Hz, 2H), 6.07 (t, *J* = 3.3 Hz, 1H), 4.24 – 4.17 (m, 2H), 3.98 (dd, *J* = 37.6, 16.9 Hz, 2H), 3.15 (s, 3H), 2.27 (s, 3H), 2.18 – 2.07 (m, 2H), 1.59 – 1.50 (m, 2H), 1.38 – 1.30 (m, 4H), 0.84 (t, *J* = 7.1 Hz, 3H).

^{13}C NMR (101 MHz, Acetone-*d$_6$*) δ 208.7, 168.7, 168.6, 136.5, 133.6, 130.1, 127.0, 92.9, 62.6, 62.5, 47.3, 32.5, 30.5, 29.4, 23.2, 21.1, 14.3.

HRMS (ESI): calculated for $C_{20}H_{26}BNO_4$ [M+Na]$^+$, 378.1847; Found, 378.1843.

(*S*)-2-(1-(4-methoxyphenyl)nona-1,2-dien-3-yl)-6-methyl-1,3,6,2-dioxazaborocane-4,8-dione

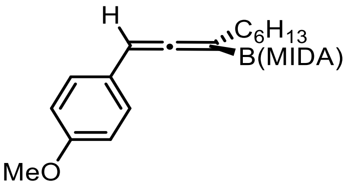

32

The product **32** was obtained in 85% (33 mg) yield as a colorless semisolid after column chromatography (eluent = ethyl acetate/petroleum ether v/v 2:1).

^{1}H NMR (500 MHz, Acetone-*d$_6$*) δ 7.20 (d, *J* = 8.7 Hz, 2H), 6.85 (d, *J* = 8.7 Hz, 2H), 6.07 (t, *J* = 3.3 Hz, 1H), 4.22 (dd, *J* = 16.9, 3.2 Hz, 2H), 4.04 (d, *J* = 16.8 Hz, 1H), 3.95 (d, *J* = 16.9 Hz, 1H), 3.77 (s, 3H), 3.15 (s, 3H), 2.18 – 2.07 (m, 2H), 1.58 – 1.50 (m, 2H), 1.39 – 1.33 (m, 2H), 1.31 – 1.26 (m, 4H), 0.87 – 0.81 (m, 3H).

^{13}C NMR (101 MHz, Acetone-*d$_6$*) δ 208.5, 168.8, 168.6, 159.3, 128.6, 128.1, 114.9, 92.6, 62.5, 62.4, 55.5, 47.3, 32.5, 30.6, 29.9, 23.3, 14.3.

HRMS (ESI): calculated for $C_{21}H_{28}BNO_5$ [M+Na]$^+$, 408.1953; Found, 408.1954.

(*S*)-2-(1-([1,1'-biphenyl]-4-yl)octa-1,2-dien-3-yl)-6-methyl-1,3,6,2-dioxazaborocane-4,8-dione

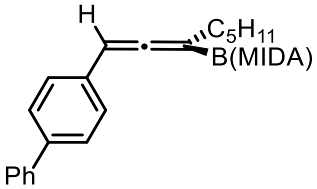

33

The product **33** was obtained in 80% (33 mg) yield as a colorless semisolid after column chromatography (eluent = ethyl acetate/petroleum ether v/v 2:1).

^{1}H NMR (500 MHz, Chloroform-*d*) δ 7.48 (d, *J* = 7.6 Hz, 2H), 7.44 (d, *J* = 8.2 Hz, 2H), 7.35 (t, *J* = 7.7 Hz, 2H), 7.25 (t, *J* = 7.4 Hz, 1H), 7.22 – 7.17 (m, 2H), 6.00 (t, *J* = 3.4 Hz, 1H), 3.76 (d, *J* = 16.6 Hz, 2H), 3.55 (d, *J* = 16.6 Hz, 1H), 3.46 (d, *J* = 16.6 Hz, 1H), 2.74 (s, 3H), 2.06 – 1.88 (m, 2H), 1.49 – 1.40 (m, 2H), 1.25 – 1.17 (m, 4H), 0.77 (t, *J* = 7.0 Hz, 3H).

^{13}C NMR (126 MHz, Chloroform-*d*) δ 208.3, 167.8, 167.8, 140.7, 139.5, 134.2, 129.0, 127.6, 127.4, 126.9, 126.8, 92.7, 61.8, 61.7, 46.5, 31.9, 30.0, 28.7, 22.7, 14.2.

HRMS (ESI): calculated for $C_{25}H_{28}BNO_4$ [M+Na]$^+$, 440.2004; Found, 440.2004.

(*S*)-2-(1-(4-fluorophenyl)octa-1,2-dien-3-yl)-6-methyl-1,3,6,2-dioxazaborocane-4,8-dione

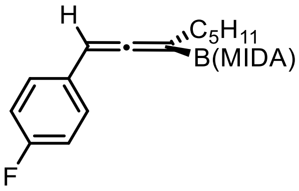

34

The product **34** was obtained in 73% (26 mg) yield as a colorless semisolid after column chromatography (eluent = ethyl acetate/petroleum ether v/v 2:1).

^{1}H NMR (400 MHz, Chloroform-*d*) δ 7.21 – 7.15 (m, 2H), 7.01 – 6.94 (m, 2H), 6.03 (t, *J* = 3.3 Hz, 1H), 3.83 (dd, *J* = 16.4, 3.5 Hz, 2H), 3.66 (d, *J* = 16.4 Hz, 1H), 3.57 (d, *J* = 16.5 Hz, 1H), 2.88 (s, 3H), 2.15 – 1.99 (m, 2H), 1.55 – 1.47 (m, 2H), 1.33 – 1.27 (m, 4H), 0.84 (t, *J* = 7.0 Hz, 3H).

^{13}C NMR (126 MHz, Chloroform-*d*) δ 207.9 (d, *J* = 2.2 Hz), 167.2, 167.1, 161.8 (d, *J* = 245.7 Hz), 132.2 (d, *J* = 10.0 Hz), 130.9 (d, *J* = 3.2 Hz), 128.7 (d, *J* = 12.1 Hz), 127.7 (d, *J* = 8.0 Hz), 115.8 (d, *J* = 21.7 Hz), 92.3, 61.9, 61.8, 46.5, 31.8, 30.1, 28.7, 22.6, 14.2.

HRMS (ESI): calculated for $C_{19}H_{23}BFNO_4$ $[M+Na]^+$, 382.1596; Found, 382.1597.

6-methyl-2-(1-(4-(trifluoromethoxy)phenyl)octa-1,2-dien-3-yl)-1,3,6,2-dioxazaborocane-4,8-dione

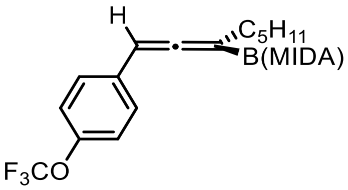

35

The product **35** was obtained in 81% (34 mg) yield as a colorless semisolid after column chromatography (eluent = ethyl acetate/petroleum ether v/v 2:1).

^{1}H NMR (500 MHz, Acetone-*d$_6$*) δ 7.40 (d, *J* = 8.5 Hz, 2H), 7.24 (d, *J* = 8.2 Hz, 2H), 6.17 (t, *J* = 3.5 Hz, 1H), 4.27 (dd, *J* = 16.9, 7.9 Hz, 2H), 4.05 (dd, *J* = 24.1, 17.0 Hz, 2H), 3.18 (s, 3H), 2.15 (m, 2H), 1.59 – 1.51 (m, 2H), 1.35 – 1.28 (m, 4H), 0.83 (t, *J* = 7.0 Hz, 3H).

^{13}C NMR (126 MHz, Acetone-*d$_6$*) δ 208.9, 168.9, 168.5, 148.0 (d, *J* = 1.9 Hz), 136.2, 132.7 (d, *J* = 9.8 Hz), 129.5 (d, *J* = 11.9 Hz), 128.4, 122.1, 121.5 (q, *J* = 255.6 Hz), 92.0, 62.6, 62.5, 47.4, 32.4, 30.3, 29.3, 23.2, 14.3.

HRMS (ESI): calculated for $C_{20}H_{23}BF_3NO_5$ $[M+Na]^+$, 448.1514; Found, 448.1512.

(*S*)-6-methyl-2-(1-(4-(trifluoromethyl)phenyl)octa-1,2-dien-3-yl)-1,3,6,2-dioxazaborocane-4,8-dione

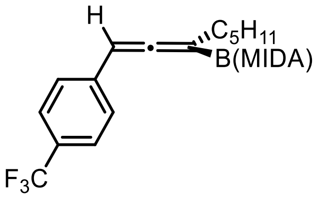

36

The product **36** was obtained in 88% (36 mg) yield as a colorless semisolid after column chromatography (eluent = ethyl acetate/petroleum ether v/v 2:1).

^{1}H NMR (500 MHz, Chloroform-*d*) δ 7.51 (d, *J* = 8.1 Hz, 2H), 7.31 (d, *J* = 8.1 Hz, 2H), 6.07 (t, *J* = 3.4 Hz, 1H), 3.91 (dd, *J* = 16.7, 3.0 Hz, 2H), 3.60 (dd, *J* = 27.2, 16.7 Hz, 2H), 2.85 (s, 3H), 2.12 – 2.00 (m, 2H), 1.53 – 1.48 (m, 2H), 1.30 – 1.25 (m, 4H), 0.83 (t, *J* = 7.0 Hz, 3H).

^{13}C NMR (126 MHz, Chloroform-*d*) δ 210.6, 167.9, 167.8, 139.3, 132.2 (d, *J* = 9.9 Hz), 128.7 (d, *J* = 12.2 Hz), 128.4 (d, *J* = 32.3 Hz), 126.4, 125.8 (d, *J* = 4.0 Hz), 124.4 (q, *J* = 272.3 Hz), 92.3, 61.8, 61.7, 46.7, 31.8, 29.8, 28.6, 22.6, 14.1.

HRMS (ESI): calculated for $C_{20}H_{23}BF_3NO_4$ $[M+Na]^+$, 432.1564; Found, 432.1568.

(*S*)-3-(3-(6-methyl-4,8-dioxo-1,3,6,2-dioxazaborocan-2-yl)octa-1,2-dien-1-yl)benzaldehyde

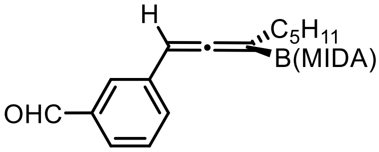

37

The product **37** was obtained in 65% (24 mg) yield as a colorless semisolid after column chromatography (eluent = ethyl acetate/petroleum ether v/v 2:1).

^{1}H NMR (400 MHz, Chloroform-*d*) δ 9.95 (s, 1H), 7.70 (s, 1H), 7.63 (d, *J* = 7.3 Hz, 2H), 7.52 – 7.41 (m, 1H), 6.11 (t, *J* = 3.3 Hz, 1H), 3.91 (dd, *J* = 16.6, 3.2 Hz, 2H), 3.70 (d, *J* = 16.6 Hz, 1H), 3.63 (d, *J* = 16.7 Hz, 1H), 2.93 (s, 3H), 2.15 – 2.00 (m, 2H), 1.55 – 1.45 (m, 2H), 1.31 – 1.25 (m, 4H), 0.82 (t, *J* = 7.1 Hz, 3H).

^{13}C NMR (101 MHz, Chloroform-*d*) δ 208.4, 192.6, 167.7, 167.5, 136.9, 136.5, 132.3, 129.6, 128.5, 126.5, 92.2, 61.9, 61.8, 46.7, 31.8, 29.9, 28.6, 22.6.

HRMS (ESI): calculated for $C_{20}H_{24}BNO_5$ $[M+Na]^+$, 392.1640; Found, 392.1636.

(*S*)-2-(1-(4-acetylphenyl)nona-1,2-dien-3-yl)-6-methyl-1,3,6,2-dioxazaborocane-4,8-dione

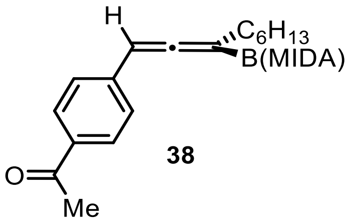

38

The product **38** was obtained in 74% (29 mg) yield as a colorless semisolid after column chromatography (eluent = ethyl acetate/petroleum ether v/v 2:1).

^{1}H NMR (400 MHz, Acetone-*d6*) δ 7.76 (d, *J* = 8.4 Hz, 2H), 7.26 (d, *J* = 8.2 Hz, 2H), 6.07 (t, *J* = 3.3 Hz, 1H), 4.12 (dd, *J* = 16.9, 3.6 Hz, 2H), 3.93 (d, *J* = 16.9 Hz, 1H), 3.87 (d, *J* = 17.0 Hz, 1H), 3.05 (s, 3H), 2.40 (s, 3H), 2.08 – 1.99 (m, 2H), 1.46 – 1.38 (m, 2H), 1.24 – 1.20 (m, 2H), 1.13 – 1.10 (m, 4H), 0.73 – 0.65 (m, 3H).

^{13}C NMR (101 MHz, Acetone-*d6*) δ 209.6, 197.2, 168.7, 168.5, 146.6, 142.0, 136.0, 129.5, 126.9, 125.0, 92.7, 62.6, 62.6, 47.4, 32.5, 26.6, 26.5, 23.3, 14.3.

HRMS (ESI): calculated for $C_{22}H_{28}BNO_5$ $[M+Na]^+$, 420.1953; Found, 420.1949.

(*S*)-2-(1-(3,5-dimethylphenyl)octa-1,2-dien-3-yl)-6-methyl-1,3,6,2-dioxazaborocane-4,8-dione

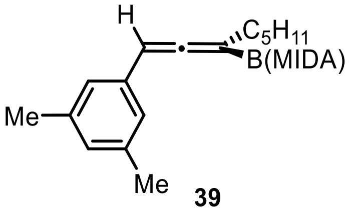

39

The product **39** was obtained in 63% (23 mg) yield as a colorless semisolid after column chromatography (eluent = ethyl acetate/petroleum ether v/v 2:1).

^{1}H NMR (400 MHz, Acetone-d6) δ 6.89 (s, 2H), 6.78 (s, 1H), 6.03 (t, *J* = 3.3 Hz, 1H), 4.22 (d, *J* = 16.9 Hz, 2H), 4.03 (d, *J* = 16.9 Hz, 1H), 3.94 (d, *J* = 17.0 Hz, 1H), 3.16 (s, 3H), 2.23 (s, 6H), 2.18 – 2.07 (m, 2H), 1.59 – 1.51 (m, 2H), 1.35- -1.29 (m, 4H), 0.85 (t, *J* = 7.1 Hz, 3H).

^{13}C NMR (126 MHz, Acetone-*d6*) δ 208.8, 168.8, 138.6, 136.4, 128.6, 124.9, 93.1, 62.5, 62.4, 47.2, 32.5, 30.4, 29.3, 23.2, 21.3, 14.4.

HRMS (ESI): calculated for $C_{21}H_{28}BNO_4$ $[M+Na]^+$, 392.2004; Found, 392.2003.

(*S*)-2-(1-(3-fluoro-4-methylphenyl)octa-1,2-dien-3-yl)-6-methyl-1,3,6,2-dioxazaborocane-4,8-dione

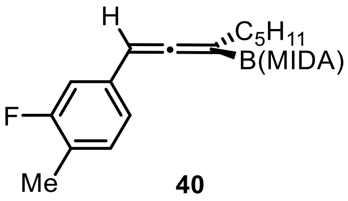

40

The product **40** was obtained in 72% (27 mg) yield as a colorless semisolid after column chromatography (eluent = ethyl acetate/petroleum ether v/v 2:1).

^{1}H NMR (400 MHz, Chloroform-*d*) δ 7.07 (t, *J* = 7.9 Hz, 1H), 6.90 – 6.83 (m, 2H), 6.00 (t, *J* = 3.3 Hz, 1H), 3.84 (dd, *J* = 16.5, 4.9 Hz, 2H), 3.66 (d, *J* = 16.5 Hz, 1H), 3.57 (d, *J* = 16.6 Hz, 1H), 2.88 (s, 3H), 2.22 (s, 3H), 2.12 – 2.02 (m, 2H), 1.55 – 1.47 (m, 2H), 1.33 – 1.27 (m, 4H), 0.85 (t, *J* = 7.0 Hz, 3H).

^{13}C NMR (101 MHz, Chloroform-*d*) δ 207.9, 167.6, 161.7 (d, *J* = 244.3 Hz), 134.8 (d, *J* = 7.7 Hz), 131.8 (d, *J* = 5.5 Hz), 123.1 (d, *J* = 17.6 Hz), 121.9 (d, *J* = 3.0 Hz), 112.4 (d, *J* = 23.1 Hz), 92.4 (d, *J* = 2.5 Hz), 61.8, 61.7, 46.6, 31.8, 30.0, 28.6, 22.6, 14.4 (d, *J* = 3.3 Hz), 14.2.

HRMS (ESI): calculated for $C_{20}H_{25}BFNO_4$ $[M+Na]^+$, 396.1753; Found, 396.1750.

methyl (*S*)-2-methyl-5-(3-(6-methyl-4,8-dioxo-1,3,6,2-dioxazaborocan-2-yl)octa-1,2-dien-1-yl)benzoate

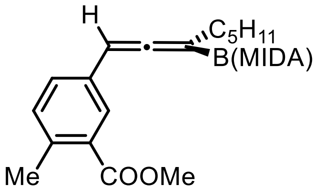

41

The product **41** was obtained in 91% (38 mg) yield as a colorless semisolid after column chromatography (eluent = ethyl acetate/petroleum ether v/v 2:1).

^{1}H NMR (500 MHz, Chloroform-*d*) δ 7.72 (s, 1H), 7.26 (d, *J* = 8.4 Hz, 1H), 7.15 (d, *J* = 7.9 Hz, 1H), 6.05 (t, *J* = 3.4 Hz, 1H), 3.90 – 3.85 (m, 5H), 3.66 (d, *J* = 16.6 Hz, 1H), 3.57 (d, *J* = 16.6 Hz, 1H), 2.89 (s, 3H), 2.53 (s, 3H), 2.13 – 1.98 (m, 2H), 1.56 – 1.46 (m, 2H), 1.32 – 1.26 (m, 4H), 0.84 (t, *J* = 7.0 Hz, 3H).

^{13}C NMR (126 MHz, Chloroform-*d*) δ 208.1, 168.2, 167.8, 167.7, 138.1, 132.9, 132.4, 130.1, 129.7, 128.1, 92.2, 61.8, 61.6, 52.1, 46.5, 31.8, 29.9, 28.6, 22.6, 21.5, 14.2.

HRMS (ESI): calculated for $C_{22}H_{28}BNO_6$ $[M+Na]^+$, 436.1902; Found, 436.1908.

(*S*)-2-(1-(4-bromo-3,5-dimethylphenyl)octa-1,2-dien-3-yl)-6-methyl-1,3,6,2-dioxazaborocane-4,8-dione

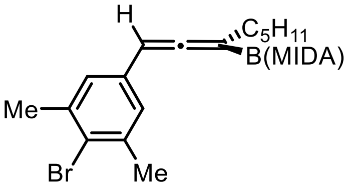

42

The product **42** was obtained in 83% (37 mg) yield as a colorless semisolid after column chromatography (eluent = ethyl acetate/petroleum ether v/v 2:1).

^{1}H NMR (400 MHz, Chloroform-*d*) δ 6.85 (s, 2H), 5.87 (t, *J* = 3.3 Hz, 1H), 3.79 (dd, *J* = 16.6, 1.7 Hz, 2H), 3.49 (dd, *J* = 25.1, 16.7 Hz, 2H), 2.73 (s, 3H), 2.28 (s, 6H), 2.08 – 1.87 (m, 2H), 1.48 – 1.38 (m, 2H), 1.26 – 1.19 (m, 4H), 0.78 (t, *J* = 7.0 Hz, 3H).

^{13}C NMR (126 MHz, Chloroform-*d*) δ 208.0, 167.6, 167.5, 138.7, 133.5, 126.1, 125.5, 92.4, 61.8, 61.7, 46.5, 31.8, 29.9, 28.6, 24.0, 22.7, 14.2.

HRMS (ESI): calculated for $C_{21}H_{27}BBrNO_4$ $[M+Na]^+$, 470.1109; Found, 470.1007.

(S)-2-(1-(4-hydroxyphenyl)octa-1,2-dien-3-yl)-6-methyl-1,3,6,2-dioxazaborocane-4,8-dione

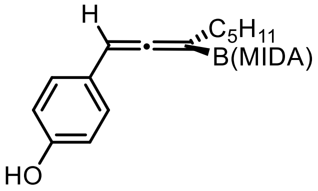

43

The product **43** was obtained in 78% (28 mg) yield as a colorless semisolid after column chromatography (eluent = ethyl acetate/petroleum ether v/v 2:1).

^{1}H NMR (400 MHz, Acetone-*d6*) δ 8.29 (s, 1H), 7.11 (d, *J* = 8.2 Hz, 2H), 6.76 (d, *J* = 8.2 Hz, 2H), 6.04 (t, *J* = 3.4 Hz, 1H), 4.21 (d, *J* = 16.9 Hz, 2H), 4.02 (d, *J* = 17.1 Hz, 1H), 3.93 (d, *J* = 16.9 Hz, 1H), 3.14 (s, 3H), 2.17 – 2.07 (m, 2H), 1.58 – 1.51 (m, 2H), 1.36 – 1.28 (m, 4H), 0.84 (t, *J* = 7.0 Hz, 3H).

^{13}C NMR (101 MHz, Acetone-*d6*) δ 208.5, 168.8, 168.7, 157.0, 128.2, 127.4, 116.3, 92.8, 62.6, 62.5, 47.3, 32.5, 30.6, 29.4, 23.2, 14.4.

HRMS (ESI): calculated for $C_{19}H_{24}BNO_5$ $[M+Na]^+$, 380.1640; Found, 380.1645.

(*S*)-2-(1-(4-hydroxy-3,5-dimethylphenyl)octa-1,2-dien-3-yl)-6-methyl-1,3,6,2-dioxazaborocane-4,8-dione

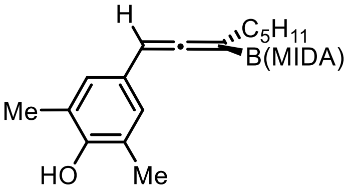

44

The product **44** was obtained in 80% (31 mg) yield as a colorless semisolid after column chromatography (eluent = ethyl acetate/petroleum ether v/v 2:1).

^{1}H NMR (400 MHz, Chloroform-*d*) δ 7.82 (d, *J* = 8.1 Hz, 1H), 7.12 – 7.04 (m, 2H), 6.03 (t, *J* = 3.3 Hz, 1H), 3.90 – 3.81 (m, 5H), 3.63 (d, *J* = 16.6 Hz, 1H), 3.55 (d, *J* = 16.6 Hz, 1H), 2.85 (s, 3H), 2.55 (s, 3H), 2.15 – 1.98 (m, 2H), 1.50 (q, *J* = 7.3 Hz, 2H), 1.32 – 1.26 (m, 4H), 0.84 (t, *J* = 7.0 Hz, 3H).

^{13}C NMR (101 MHz, Chloroform-*d*) δ 208.7, 167.9, 167.6, 167.6, 141.0, 139.3, 131.4, 129.6, 127.6, 92.6, 61.8 (d, *J* = 11.1 Hz), 51.8, 46.6, 31.8, 29.9, 28.6, 22.6, 22.0, 14.2.

HRMS (ESI): calculated for $C_{21}H_{28}BNO_5$ $[M+Na]^+$, 408.1953; Found, 408.1954.

(*S*)-6-methyl-2-(1-(naphthalen-2-yl)nona-1,2-dien-3-yl)-1,3,6,2-dioxazaborocane-4,8-dione

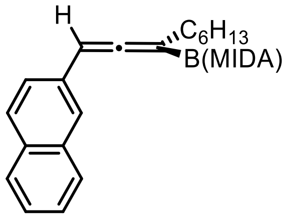

45

The product **45** was obtained in 77% (31 mg) yield as a colorless semisolid after column chromatography (eluent = ethyl acetate/petroleum ether v/v 2:1).

^{1}H NMR (500 MHz, Chloroform-*d*) δ 7.78 – 7.69 (m, 3H), 7.57 (s, 1H), 7.47 – 7.35 (m, 3H), 6.17 (t, *J* = 3.4 Hz, 1H), 3.72 (dd, *J* = 16.8, 3.9 Hz, 2H), 3.48 (d, *J* = 16.8 Hz, 1H), 3.38 (d, *J* = 16.8 Hz, 1H), 2.60 (s, 3H), 2.09 – 2.04 (m, 1H), 1.98 – 1.91 (m, 1H), 1.55 – 1.46 (m, 2H), 1.34 – 1.23 (m, 6H), 0.83 (t, *J* = 6.4, 3H).

^{13}C NMR (126 MHz, Chloroform-*d*) δ 208.6, 168.5, 168.4, 133.9, 132.8, 132.4, 128.5, 127.8, 127.7, 126.6, 125.7, 124.7, 124.6, 93.3, 61.7, 61.6, 46.3, 31.9, 29.8, 29.4, 28.9, 22.8, 14.2.

HRMS (ESI): calculated for $C_{24}H_{28}BNO_4$ $[M+Na]^+$, 428.2004; Found, 428.2001.

(*S*)-2-(1-(9H-fluoren-2-yl)octa-1,2-dien-3-yl)-6-methyl-1,3,6,2-dioxazaborocane-4,8-dione

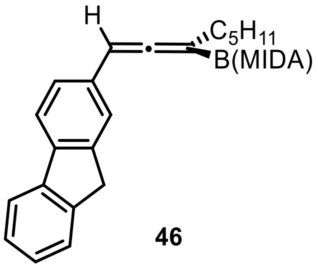

46

The product **46** was obtained in 62% (27 mg) yield as a colorless semisolid after column chromatography (eluent = ethyl acetate/petroleum ether v/v 2:1).

^{1}H NMR (400 MHz, Acetone-*d$_6$*) δ 7.78 (dd, *J* = 15.3, 7.8 Hz, 2H), 7.54 (d, *J* = 7.5 Hz, 1H), 7.49 (d, *J* = 1.5 Hz, 1H), 7.38 – 7.23 (m, 3H), 6.21 (t, *J* = 3.3 Hz, 1H), 4.24 (d, *J* = 16.9 Hz, 2H), 4.06 (d, *J* = 16.9 Hz, 1H), 3.98 (d, *J* = 17.0 Hz, 1H), 3.87 (s, 2H), 3.19 (s, 3H), 2.20 – 2.12 (m, 2H), 1.64 – 1.55 (m, 2H), 1.41 – 1.29 (m, 4H), 0.85 (t, *J* = 7.1 Hz, 3H).

^{13}C NMR (101 MHz, Acetone-*d$_6$*) δ 209.0, 168.8, 168.6, 144.7, 144.1, 142.4, 140.8, 135.4, 127.6, 127.3, 125.9, 125.9, 123.6, 120.8, 120.4, 93.5, 62.6, 62.5, 47.3, 37.2, 32.5, 30.5, 29.4, 23.2, 14.4.

HRMS (ESI): calculated for $C_{26}H_{28}BNO_4$ [M+Na]$^+$, 452.2004; Found, 452.2006.

(*S*)-6-methyl-2-(1-(thiophen-2-yl)octa-1,2-dien-3-yl)-1,3,6,2-dioxazaborocane-4,8-dione

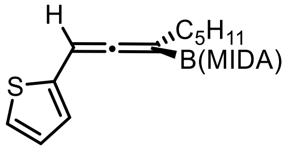

47

The product **47** was obtained in 77% (27 mg) yield as a colorless semisolid after column chromatography (eluent = ethyl acetate/petroleum ether v/v 2:1).

^{1}H NMR (400 MHz, Acetone-*d$_6$*) δ 7.21 (d, *J* = 5.1 Hz, 1H), 6.94 (dd, *J* = 5.1, 3.5 Hz, 1H), 6.88 (d, *J* = 2.5 Hz, 1H), 6.39 (t, *J* = 3.4 Hz, 1H), 4.24 (dd, *J* = 16.9, 4.8 Hz, 2H), 4.08 (d, *J* = 16.9 Hz, 1H), 3.94 (d, *J* = 16.9 Hz, 1H), 3.19 (s, 3H), 2.16 – 2.09 (m, 2H), 1.60 – 1.52 (m, 2H), 1.37 – 1.30 (m, 4H), 0.86 (t, *J* = 7.1 Hz, 3H).

^{13}C NMR (126 MHz, Acetone-*d$_6$*) δ 208.3, 168.8, 168.5, 140.8, 128.3, 124.6, 124.1, 87.8, 62.5, 62.4, 47.3, 32.4, 30.4, 29.2, 23.2, 14.3.

HRMS (ESI): calculated for $C_{17}H_{22}BNO_4S$ [M+Na]$^+$, 370.1255; Found, 370.1250.

(*S*)-6-methyl-2-(1-(thiophen-3-yl)octa-1,2-dien-3-yl)-1,3,6,2-dioxazaborocane-4,8-dione

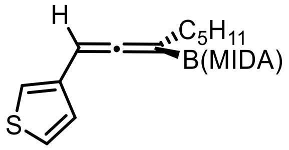

48

The product **48** was obtained in 91% (32 mg) yield as a colorless semisolid after column chromatography (eluent = ethyl acetate/petroleum ether v/v 2:1).

^{1}H NMR (400 MHz, Chloroform-*d*) δ 7.17 – 7.14 (m, 1H), 6.91 (d, *J* = 4.1 Hz, 2H), 6.06 (t, *J* = 3.4 Hz, 1H), 3.82 (dd, *J* = 16.7, 3.6 Hz, 2H), 3.58 (d, *J* = 16.8 Hz, 1H), 3.49 (d, *J* = 16.6 Hz, 1H), 2.76 (s, 3H), 2.01 – 1.90 (m, 2H), 1.42 (q, *J* = 8.0, 7.5 Hz, 2H), 1.26 – 1.18 (m, 4H), 0.78 (t, *J* = 6.9 Hz, 3H).

^{13}C NMR (101 MHz, Chloroform-*d*) δ 208.5, 168.4, 168.3, 136.2, 126.3, 126.2, 119.8, 87.7, 61.8, 61.7, 46.6, 31.8, 29.9, 28.6, 22.7, 14.2.

HRMS (ESI): calculated for $C_{17}H_{22}BNO_4S$ $[M+Na]^+$, 370.1255; Found, 370.1254.

(*S*)-5-(3-(6-methyl-4,8-dioxo-1,3,6,2-dioxazaborocan-2-yl)octa-1,2-dien-1-yl)furan-2-carbaldehyde

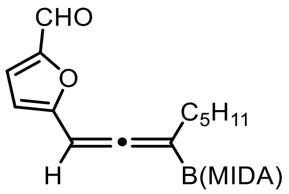

49

The product **49** was obtained in 92% (33 mg) yield as a colorless semisolid after column chromatography (eluent = ethyl acetate/petroleum ether v/v 2:1).

^{1}H NMR (400 MHz, Chloroform-*d*) δ 9.45 (s, 1H), 7.32 (d, *J* = 2.3 Hz, 1H), 6.41 (d, *J* = 2.3 Hz, 1H), 6.14 (d, *J* = 3.7 Hz, 1H), 4.24 (t, *J* = 17.1 Hz, 2H), 3.99 (d, *J* = 17.2 Hz, 1H), 3.82 (d, *J* = 16.8 Hz, 1H), 3.13 (s, 3H), 2.15 – 2.06 (m, 2H), 1.59 – 1.40 (m, 2H), 1.38 – 1.22 (m, 4H), 0.85 (t, *J* = 6.4 Hz, 3H).

^{13}C NMR (126 MHz, Chloroform-*d*) δ 207.5, 174.9, 167.7, 166.8, 155.0, 150.1, 108.0, 81.9, 60.7, 60.2, 45.6, 30.2, 28.2, 27.0, 21.2, 13.0.

HRMS (ESI): calculated for $C_{18}H_{22}BNO_6$ $[M+Na]^+$, 382.1432; Found, 382.1428.

methyl (*S*)-2-(3-cyclohexyl-3-(6-methyl-4,8-dioxo-1,3,6,2-dioxazaborocan-2-yl)propa-1,2-dien-1-yl) benzoate

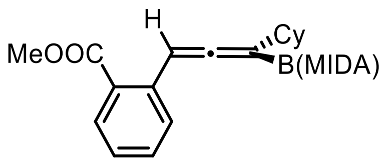

50

The product **50** was obtained in 75% (31 mg) yield as a colorless semisolid after column chromatography (eluent = ethyl acetate/petroleum ether v/v 2:1).

^{1}H NMR (400 MHz, Acetone-*d$_6$*) δ 7.94 (t, *J* = 1.8 Hz, 1H), 7.78 (dt, *J* = 7.7, 1.5 Hz, 1H), 7.57 – 7.54 (m, 1H), 7.42 (t, *J* = 7.7 Hz, 1H), 6.24 (d, *J* = 1.6 Hz, 1H), 4.24 (dd, *J* = 17.0, 2.9 Hz, 2H), 4.07 (d, *J* = 16.9 Hz, 1H), 3.96 (d, *J* = 17.0 Hz, 1H), 3.87 (s, 3H), 3.17 (s, 3H), 2.10 (dt, *J* = 14.5, 3.3 Hz, 1H), 2.03 – 1.90 (m, 2H), 1.73 (dt, *J* = 12.2, 3.2 Hz, 2H), 1.36 – 1.16 (m, 6H).

^{13}C NMR (126 MHz, Acetone-d$_6$) δ 208.8, 168.8, 168.5, 167.2, 137.4, 131.5, 131.1, 129.7, 127.8, 127.5, 93.3, 62.6, 62.5, 52.4, 47.5, 40.0, 34.4, 27.5, 27.4, 26.8.

HRMS (ESI): calculated for $C_{22}H_{26}BNO_6$ $[M+Na]^+$, 434.1745; Found, 434.1747.

methyl (*S*)-2-methyl-4-(3-(6-methyl-4,8-dioxo-1,3,6,2-dioxazaborocan-2-yl)octa-1,2-dien-1-yl)benzoate

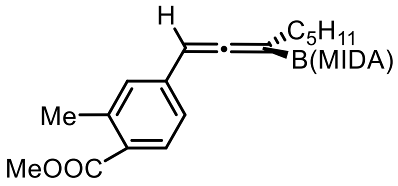

51

The product **51** was obtained in 78% (32 mg) yield as a colorless semisolid after column chromatography (eluent = ethyl acetate/petroleum ether v/v 2:1).

^{1}H NMR (400 MHz, Chloroform-*d*) δ 7.82 (d, *J* = 8.1 Hz, 1H), 7.11 – 7.04 (m, 2H), 6.03 (t, *J* = 3.3 Hz, 1H), 3.90 – 3.83 (m, 5H), 3.63 (d, *J* = 16.6 Hz, 1H), 3.55 (d, *J* = 16.6 Hz, 1H), 2.85 (s, 3H), 2.54 (s, 3H), 2.15 – 1.97 (m, 2H), 1.50 (td, *J* = 8.7, 8.3, 3.8 Hz, 2H), 1.32 – 1.25 (m, 4H), 0.83 (t, *J* = 7.0 Hz, 3H).

^{13}C NMR (126 MHz, Chloroform-*d*) δ 208.7, 167.9, 167.8, 141.0, 139.3, 131.4, 129.6, 127.5, 123.4, 92.6, 61.8, 61.7, 51.9, 46.6, 31.8, 29.8, 28.6, 22.6, 22.0, 14.2.

HRMS (ESI): calculated for $C_{22}H_{28}BNO_6$ $[M+Na]^+$, 436.1902; Found, 436.1897.

4. Derivatization of the product 1

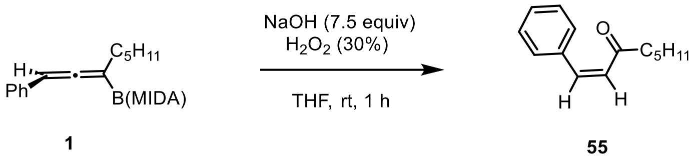

In a flask under nitrogen, **1** (0.1 mmol, 1 equiv, 34.1 mg), NaOH (3 M, 0.75 mmol, 7.5 equiv) and H_2O_2 (30%, 0.25 mL) were added to THF (1.0 mL, 0.1 M) at 0 °C. The reaction mixture was stirred at rt until total consumption of the starting material, as monitored by TLC (about 1 hour). The reaction mixture was diluted with EA (5 mL) and water (5 mL). The organic phase was separated and the aqueous layer was extracted with EA (5 mL) for three times. The combined organic layer was dried over anhydrous Na_2SO_4 and concentrated under reduced pressure. The crude residue was purified on flash chromatography.

(*Z)*-1-phenyloct-1-en-3-one

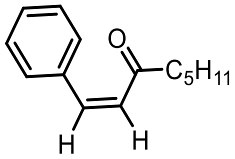

55

The product **55** was obtained in 94% (19 mg) yield as a colorless oil after column chromatography (eluent = ethyl acetate/petroleum ether v/v 0:1).

^{1}H NMR (400 MHz, Chloroform-*d*) δ 7.51 (dd, *J* = 7.0, 2.7 Hz, 2H), 7.37 – 7.31 (m, 3H), 6.82 (d, *J* = 12.7 Hz, 1H), 6.18 (d, *J* = 12.8 Hz, 1H), 2.44 (t, *J* = 7.4 Hz, 2H), 1.62 – 1.56 (m, 2H), 1.30 – 1.23 (m, 4H), 0.86 (t, *J* = 6.9 Hz, 3H).

^{13}C NMR (126 MHz, Chloroform-*d*) δ 203.8, 139.7, 135.5, 129.7, 129.2, 128.8, 128.4, 43.7, 31.4, 24.0, 22.6, 14.0.

HRMS (ESI): calculated for $C_{14}H_{18}O$ $[M+Na]^+$, 225.1250; Found, 225.1252.

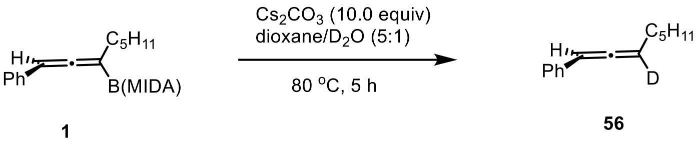

In a flask under air, **1** (0.1 mmol, 1 equiv, 34.1 mg), Cs_2CO_3 (1.0 mmol, 10.0 equiv, 0.33 g) were added to dioxane (1 mL, 0.1 M) and D_2O (0.2 mL). The reaction mixture was stirred at 80 °C until total consumption of the starting material, as monitored by TLC (about 5 hours). The reaction mixture was diluted with EA (5 mL) and water (5 mL). The organic phase was separated and the aqueous layer was extracted with EA (5 mL) for three times. The combined organic layer was dried over anhydrous Na_2SO_4 and concentrated under reduced pressure. The crude residue was purified on flash chromatography.

(*R*)-(octa-1,2-dien-1-yl-3-d)benzene

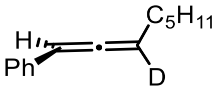

56

The product **56** was obtained in 93% (17 mg) yield as a colorless oil after column chromatography (eluent = ethyl acetate/petroleum ether v/v 2:1).

^{1}H NMR (400 MHz, Chloroform-*d*) δ 7.30 (d, *J* = 4.3 Hz, 4H), 7.18 (dt, *J* = 8.7, 4.2 Hz, 1H), 6.13 (t, *J* = 3.0 Hz, 1H), 2.13 (td, *J* = 7.4, 3.0 Hz, 2H), 1.54 – 1.46 (m, 2H), 1.41 – 1.29 (m, 4H), 0.90 (t, *J* = 7.2 Hz, 2H).

^{13}C NMR (126 MHz, Chloroform-*d*) δ 205.3, 135.3, 128.7, 126.7, 126.7, 94.7, 31.5, 29.0, 28.8, 22.6, 14.2.

HRMS (APCI): calculated for $C_{15}H_{21}D$ $[M+H]^+$, 188.1544; Found, 188.1542.

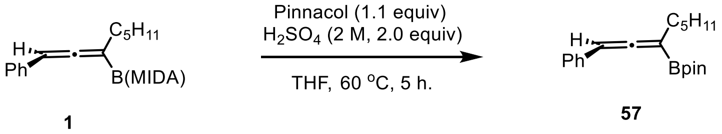

In a flask under nitrogen, **1** (0.1 mmol, 1 equiv, 34.1 mg), pinnacol (0.11 mmol, 1.1 equiv, 13 mg) and H_2SO_4 (2 M, 2 equiv) were added to THF (1.0 mL, 0.1 M). The reaction mixture was stirred at 60 °C until total consumption of the starting material, as monitored by TLC (about 5 hour). The reaction mixture was diluted with EA (5 mL) and water (5 mL). The organic phase was separated and the aqueous layer was extracted with EA (5 mL) for three times. The combined organic layer was dried over anhydrous Na_2SO_4 and concentrated under reduced pressure. The crude residue was purified on flash chromatography.

(*R*)-4,4,5,5-tetramethyl-2-(1-phenylocta-1,2-dien-3-yl)-1,3,2-dioxaborolane

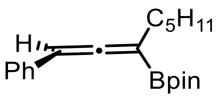

57

The product **57** was obtained in 83% (26 mg) yield as a yellow oil after column chromatography (eluent = ethyl acetate/petroleum ether v/v 1:10).

^{1}H NMR (500 MHz, Chloroform-*d*) δ 7.30 – 7.22 (m, 4H), 7.15 (t, *J* = 7.0 Hz, 1H), 6.12 (t, *J* = 2.8 Hz, 1H), 2.17 (qd, *J* = 5.0, 2.3 Hz, 2H), 1.52 – 1.46 (m, 2H), 1.27 (d, *J* = 9.8 Hz, 16H), 0.85 (t, *J* = 7.0 Hz, 3H).

^{13}C NMR (126 MHz, Chloroform-*d*) δ 213.0, 135.0, 128.6, 126.7, 126.4, 91.6, 83.9, 31.7, 30.0, 29.9, 29.1, 25.0, 24.7, 22.6, 14.2.

HRMS (ESI): calculated for $C_{20}H_{29}BO_2$ $[M+Na]^+$, 335.2153; Found, 335.2156.

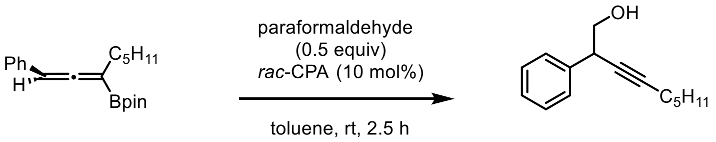

In a flask under nitrogen, **57** (0.1 mmol, 1 equiv, 31.2 mg), paraformaldehyde (0.05 mmol, 0.5 equiv, 4.5 mg) and *rac*-CPA (0.01 mmol, 10 mol%, 3.5 mg) were added to toluene (1.0 mL, 0.1 M). The reaction mixture was stirred at rt until total consumption of the starting material, as monitored by TLC (about 2.5 hours). The reaction mixture was diluted with EA (5 mL) and water (5 mL). The organic phase was separated and the aqueous layer was extracted with EA (5 mL) for three times. The combined organic layer was dried over anhydrous Na_2SO_4 and concentrated under reduced pressure. The crude residue

was purified on flash chromatography.

2-phenylnon-3-yn-1-ol

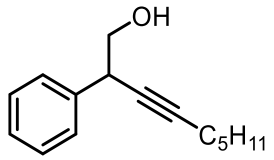

58

The product **58** was obtained in 52% (11 mg) yield as a yellow solid after column chromatography (eluent = ethyl acetate/petroleum ether v/v 1:10)

^{1}H NMR (400 MHz, Chloroform-*d*) δ 7.33 – 7.24 (m, 4H), 7.21 - 7.17 (m, 1H), 3.80 - 3.75 (m, 1H), 3.68 - 3.58 (m, 2H), 2.21 - 2.16 (m, 2H), 1.52 - 1.45 (m, 2H), 1.37 – 1.22 (m, 4H), 0.84 (t, *J* = 7.0 Hz, 3H).

^{13}C NMR (101 MHz, Chloroform-*d*) δ 138.8, 128.7, 128.0, 127.4, 85.6, 78.6, 68.1, 41.7, 31.3, 28.8, 22.3, 18.9, 14.1.

HRMS (ESI): calculated for $C_{15}H_{20}O$ $[M+H]^{+,}$ 217.1587; Found, 217.1592.

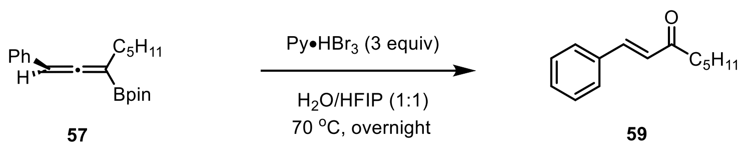

In a flask under nitrogen, **57** (0.1 mmol, 1 equiv, 31.2 mg), Py·HBr_3 (0.3 mmol, 3 equiv, 48 mg) were added to HFIP/H_2O (1:1, 1.0 mL, 0.1 M). The reaction mixture was stirred at 70 °C until total consumption of the starting material, as monitored by TLC. The reaction mixture was diluted with EA (5 mL) and water (5 mL). The organic phase was separated and the aqueous layer was extracted with EA (5 mL) for three times. The combined organic layer was dried over anhydrous Na_2SO_4 and concentrated under reduced pressure. The crude residue was purified on flash chromatography.

(*E*)-1-phenyloct-1-en-3-one

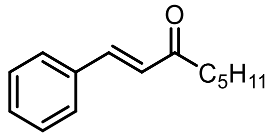

59

The product **59** was obtained in 62% (12 mg) yield as a yellow solid after column chromatography (eluent = ethyl acetate/petroleum ether v/v 1:10

^{1}H NMR (400 MHz, Chloroform-*d*) δ 7.59 – 7.51 (m, 3H), 7.40 (q, *J* = 2.9 Hz, 3H), 6.74 (d, *J* = 16.2 Hz, 1H), 2.66 (t, *J* = 7.5 Hz, 2H), 1.73 - 1.65 (m, 2H), 1.39 – 1.31 (m, 4H), 0.91 (t, *J* = 7.0 Hz, 3H).

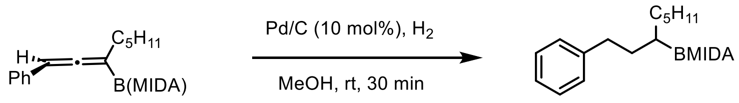

1

60

In a flask under H_2, **1** (0.1 mmol, 1 equiv, 34.1 mg) and Pd/C (0.01 mmol, 10 mol%) were added to MeOH (1.0 mL, 0.1 M). The reaction mixture was stirred at rt until total consumption of the starting material, as monitored by TLC (about 0.5 hour). The reaction mixture was diluted with EA (5 mL) and water (5 mL). The organic phase was separated and the aqueous layer was extracted with EA (5 mL) for three times. The combined organic layer was dried over anhydrous Na_2SO_4 and concentrated under reduced pressure. The crude residue was purified on flash chromatography.

6-methyl-2-(1-phenyloctan-3-yl)-1,3,6,2-dioxazaborocane-4,8-dione

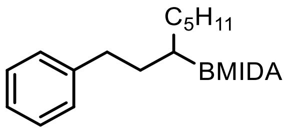

60

The product **60** was obtained in 75% (26 mg) yield as a yellow solid after column chromatography (eluent = ethyl acetate/petroleum ether v/v 3:1)

^{1}H NMR (400 MHz, Chloroform-*d*) δ 7.21 – 7.17 (m, 2H), 7.14 – 7.06 (m, 3H), 3.74 (dd, *J* = 20.4, 16.5 Hz, 2H), 3.56 (dd, *J* = 16.5, 3.6 Hz, 2H), 2.66 (s, 3H), 1.61 – 1.70 (m, 1H), 1.45 – 1.53 (m, 2H), 1.42 – 1.19 (m, 10H), 0.83 (t, *J* = 7.0 Hz, 3H).

^{13}C NMR (126 MHz, Chloroform-*d*) δ 167.1, 167.1, 143.2, 128.7, 128.5, 125.9, 62.9, 62.8, 45.6, 34.2, 32.7, 31.4, 29.2, 27.8, 22.8, 14.3.

HRMS (ESI): calculated for $C_{19}H_{28}BNO_4$ $[M+Na]^+$, 368.2004; Found, 368.2010.

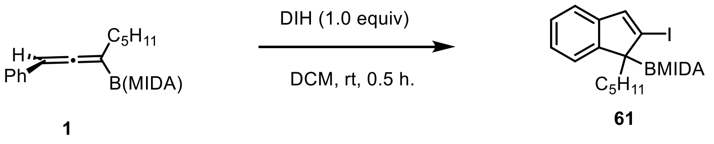

In a flask under nitrogen, **1** (0.1 mmol, 1.0 equiv, 34.1 mg), DIH (0.1 mmol, 1.0 equiv) were added to DCM (1.0 mL, 0.1 M). The reaction mixture was stirred at rt until total consumption of the starting material, as monitored by TLC (about 0.5 hour). The reaction mixture was diluted with EA (5 mL) and water (5 mL). The organic phase was separated and the aqueous layer was extracted with EA (5 mL) for three times. The combined organic layer was dried over anhydrous Na_2SO_4 and concentrated under reduced pressure. The crude residue was purified on flash chromatography.

2-(2-iodo-1-pentyl-1H-inden-1-yl)-6-methyl-1,3,6,2-dioxazaborocane-4,8-dione

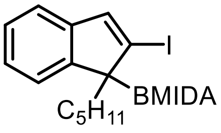

61

The product **61** was obtained in 69% (32 mg) yield as a yellow solid after column chromatography (eluent = ethyl acetate/petroleum ether v/v 2:1).

^{1}H NMR (400 MHz, Acetone-d$_6$) δ 7.54 (d, *J* = 7.5 Hz, 1H), 7.39 (d, *J* = 10.7 Hz, 2H), 7.24 (td, *J* = 7.5, 1.2 Hz, 1H), 7.17 (td, *J* = 7.5, 1.3 Hz, 1H), 4.16 (d, *J* = 16.9 Hz, 1H), 4.08 (d, *J* = 17.0 Hz, 1H), 3.84 (d, *J* = 16.8 Hz, 1H), 3.29 (d, *J* = 16.9 Hz, 1H), 2.25 – 2.14 (m, 5H), 1.31 – 1.29 (m, 2H), 1.14 (dq, *J* = 12.1, 8.8, 7.9 Hz, 4H), 0.76 (t, *J* = 6.9 Hz, 3H).

^{13}C NMR (101 MHz, Acetone-*d$_6$*) δ 168.4, 167.9, 149.9, 146.7, 142.2, 126.9, 125.7, 124.1, 121.2, 109.6, 64.5, 64.2, 45.1, 34.6, 32.6, 23.0, 22.2, 14.2.

HRMS (ESI): calculated for $C_{19}H_{23}BNO_4$ $[M+Na]^+$, 490.0657; Found, 490.0658.

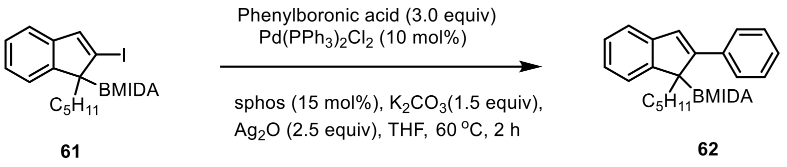

In a flask under nitrogen, **61** (0.1 mmol, 1.0 equiv, 46.7 mg), phenylboronic acid (0.3 mmol, 3.0 equiv, 41 mg), $Pd(PPh_3)_2Cl_2$ (0.01 mmol, 10 mol%, 7 mg), sphos (0.015 mmol, 15 mol%, 6 mg), K_2CO_3 (0.15 mmol, 1.5 equiv, 21 mg), Ag_2O (0.25 mmol, 2.5 equiv,58 mg), were added to dry THF (0.2 mL, 0.5 M). The reaction mixture was stirred at 60 °C until total consumption of the starting material, as monitored by TLC (about 2 hours). The reaction mixture was diluted with EA (20 mL) and water (10 mL). The organic phase was separated and the aqueous layer was extracted with EA (20 mL) for three times. The combined organic layer was dried over anhydrous Na_2SO_4 and concentrated under reduced pressure. The crude residue was purified by flash column (EA/PE as the eluent) to afford the product.

6-methyl-2-(1-pentyl-2-phenyl-1H-inden-1-yl)-1,3,6,2-dioxazaborocane-4,8-dione

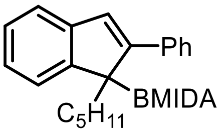

62

The product **62** was obtained in 60% (25 mg) yield as a yellow solid after column chromatography (eluent = ethyl acetate/petroleum ether v/v 2:1)

^{1}H NMR (400 MHz, Chloroform-*d*) δ 7.73 (dd, *J* = 14.7, 7.5 Hz, 3H), 7.51 – 7.28 (m, 7H), 3.63 (d, *J* = 16.5 Hz, 1H), 3.50 (d, *J* = 15.9 Hz, 1H), 3.23 (d, *J* = 16.2 Hz, 2H), 2.91 (td, *J* = 13.3, 4.5 Hz, 1H), 2.53 (td, *J* = 13.2, 4.2 Hz, 1H), 2.11 (s, 3H), 1.45 – 1.30 (m, 2H), 1.21 (q, *J* = 7.1 Hz, 4H), 0.82 (d, *J* = 6.3 Hz, 3H).

^{13}C NMR (101 MHz, Chloroform-*d*) δ 167.4, 165.7, 152.4, 151.1, 143.6, 137.4, 130.7, 129.0, 128.0, 127.5, 126.4, 125.7, 123.2, 121.3, 63.6, 63.1, 44.6, 35.2, 32.1, 22.3, 22.2, 14.1.

HRMS (ESI): calculated for $C_{25}H_{28}BNO_4$ $[M+Na]^+$, 440.2004; Found, 440.2010.

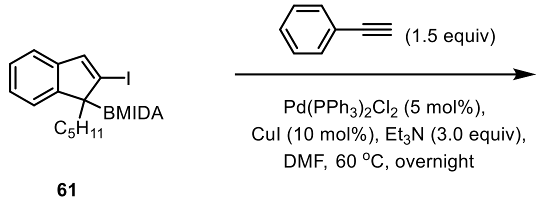

In a flask under nitrogen, iodobenzene (0.1 mmol, 1.0 equiv, 46.7 mg), phenylacetylene (0.15 mmol, 1.5 equiv, 15 mg), $Pd(PPh_3)_2Cl_2$ (0.005 mmol, 5 mol%, 4 mg) and CuI (0.01 mmol, 10 mol%, 2 mg) were added to dry DMF (1 mL, 0.1 M). Et_3N (0.3 mmol, 3.0 equiv, 30 mg) and were then added to the solution. The reaction mixture was stirred at 60 °C until total consumption of the starting material, as monitored by TLC (about 1 hour). The reaction mixture was diluted with EA (20 mL) and water (10 mL). The organic phase was separated and the aqueous layer was extracted with EA (20 mL) for three times. The combined organic layer was dried over anhydrous Na_2SO_4 and concentrated under reduced pressure. The crude residue was purified by flash column (EA/PE as the eluent) to afford the product.

6-methyl-2-(1-pentyl-2-(phenylethynyl)-1H-inden-1-yl)-1,3,6,2-dioxazaborocane-4,8-dione

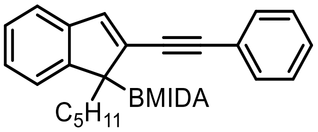

63

The product **63** was obtained in 55% (24 mg) yield as a yellow solid after column chromatography (eluent = ethyl acetate/petroleum ether v/v 2:1).

^{1}H NMR (500 MHz, Chloroform-*d*) δ 7.67 – 7.60 (m, 3H), 7.45 – 7.27 (m, 7H), 3.79 (d, *J* = 15.7 Hz, 1H), 3.74 – 3.66 (m, 2H), 3.29 (d, *J* = 16.5 Hz, 1H), 2.42 (dd, *J* = 12.6, 4.6 Hz, 1H), 2.33 (d, *J* = 4.5 Hz, 1H), 2.04 (s, 3H), 1.40 – 1.27 (m, 2H), 1.10 – 1.19 (m, 4H), 0.77 (t, *J* = 6.7 Hz, 3H).

^{13}C NMR (101 MHz, Chloroform-*d*) δ 167.3, 166.3, 149.5, 143.6, 137.0, 134.8, 132.0, 128.6, 128.5, 126.6, 126.5, 123.9, 123.2, 121.9, 97.9, 85.9, 63.4, 63.3, 44.2, 33.8, 32.0, 22.3, 22.0, 14.0.

HRMS (ESI): calculated for $C_{27}H_{28}BNO_4$ $[M+Na]^+$, 464.2004; Found, 464.1999.

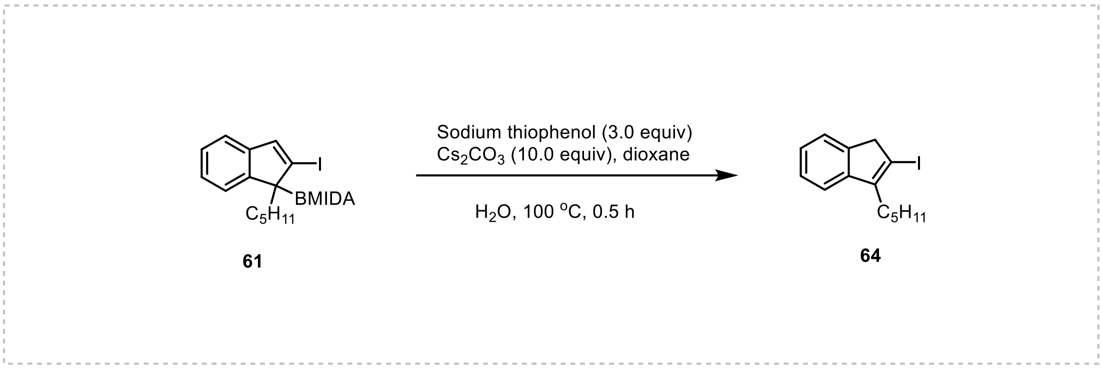

In a flask under nitrogen, **61** (0.1 mmol, 1.0 equiv, 46.7 mg), sodium thiophenol (0.3 mmol, 3.0 equiv, 40 mg), Cs_2CO_3 (1.0 mmol, 10.0 equiv, 0.33 g) and H_2O (0.2 mL) were added to dioxane (1.0 mL, 0.1 M). The reaction mixture was stirred at 100 °C until total consumption of the starting material, as monitored by TLC (about 0.5 hour). The reaction mixture was diluted with EA (5 mL) and water (5 mL). The organic phase was separated and the aqueous layer was extracted with EA (5 mL) for three times. The combined organic layer was dried over anhydrous Na_2SO_4 and concentrated under reduced pressure. The crude residue was purified on flash chromatography.

2-iodo-3-pentyl-1H-indene

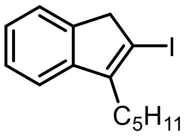

64

The product **64** was obtained in 64% (20 mg) yield as a colorless oil after column chromatography (eluent = ethyl acetate/petroleum ether v/v 0:1)

^{1}H NMR (400 MHz, Chloroform-*d*) δ 7.40 (d, *J* = 7.4 Hz, 1H), 7.35 (d, *J* = 7.6 Hz, 1H), 7.30 – 7.24 (m, 1H), 7.17 (t, *J* = 7.4 Hz, 1H), 3.61 (s, 2H), 2.61 (t, *J* = 7.8 Hz, 2H), 1.68 – 1.57 (m, 2H), 1.36 – 1.46 (m, 4H), 0.94 (t, *J* = 6.9 Hz, 3H).

^{13}C NMR (101 MHz, Chloroform-*d*) δ 149.2, 145.5, 143.7, 126.4, 124.7, 123.3, 118.7, 94.3, 48.5, 32.0, 29.7, 27.8, 22.7, 14.2.

HRMS (EI): calculated for $C_{14}H_{17}I$ [M], 312.0375; Found, 312.0369.

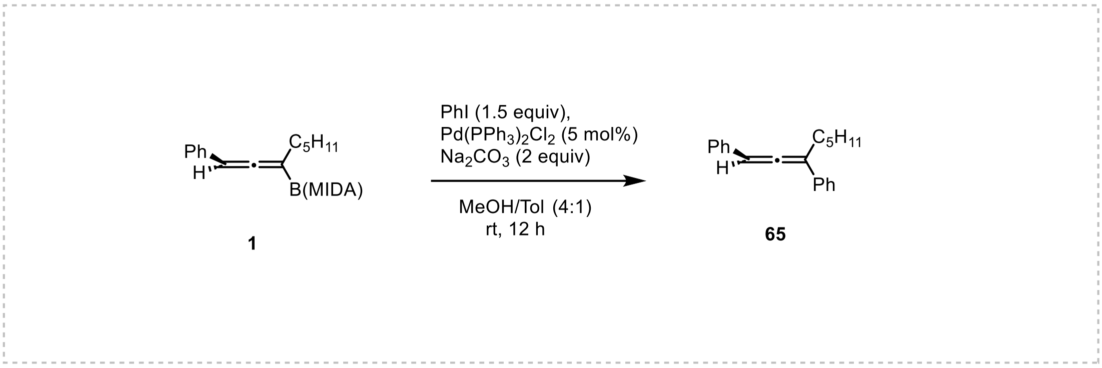

In a flask under nitrogen, **1** (0.1 mmol, 1.0 equiv, 35.3 mg), PhI (0.15 mmol, 1.5 equiv, 30.6 mg), $Pd(PPh_3)_2Cl_2$ (0.005 mmol, 5 mol%, 3.5 mg) and Na_2CO_3 (0.2 mmol, 2.0 equiv, 21.7 mg) were added to MeOH/Tolene (4:1, 0.5 mL, 0.2 M). The reaction mixture was stirred at rt until total consumption of the starting material, as monitored by TLC (about 12 hours). The reaction mixture was diluted with EA (5 mL) and water (5 mL). The organic phase was separated and the aqueous layer was extracted with EA (5 mL) for three times. The combined organic layer was dried over anhydrous Na_2SO_4 and concentrated under reduced pressure. The crude residue was purified on flash chromatography.

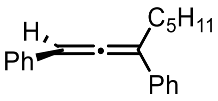

65

The product **65** was obtained in 43% (11 mg) yield as a colorless oil after column chromatography (eluent = ethyl acetate/petroleum ether v/v 0:1)

^{1}H NMR (400 MHz, Chloroform-*d*) δ 7.47 - 7.45 (m, 2H), 7.37 - 7.29 (m, 6H), 7.24 - 7.19 (m, 2H), 6.53 (t, *J* = 3.1 Hz, 1H), 2.60 - 2.54 (m, 2H), 1.68 – 1.55 (m, 2H), 1.42 – 1.32 (m, 4H), 0.88 (t, *J* = 7.1 Hz, 3H).

5. Mechanism study

(a) Reaction of non-borylated substrates was following the standard procedure.

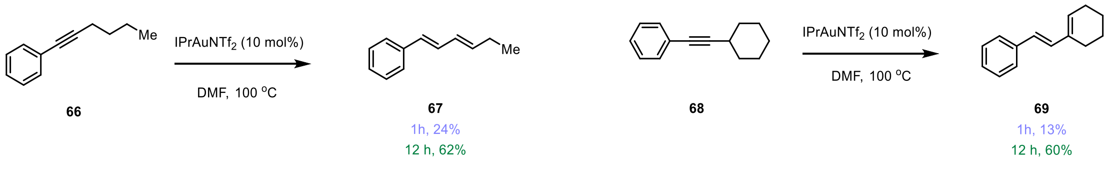

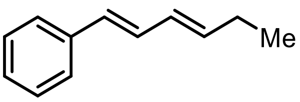

67

^{1}H NMR (400 MHz, Chloroform-*d*) δ 7.31 – 7.26 (m, 2H), 7.21 (dd, *J* = 8.5, 6.8 Hz, 2H), 7.13 – 7.08 (m, 1H), 6.67 (dd, *J* = 15.7, 10.4 Hz, 1H), 6.36 (d, *J* = 15.7 Hz, 1H), 6.17 – 6.08 (m, 1H), 5.82 – 5.75 (m, 1H), 2.13 – 2.04 (m, 1H), 0.97 (t, *J* = 7.5 Hz, 2H).

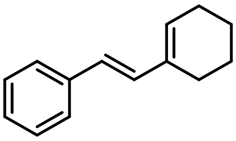

69

^{1}H NMR (400 MHz, Chloroform-*d*) δ 7.43 – 7.39 (m, 2H), 7.31 (dd, *J* = 8.5, 6.9 Hz, 2H), 7.23 – 7.17 (m, 1H), 6.79 (d, *J* = 16.2 Hz, 1H), 6.45 (d, *J* = 16.2 Hz, 1H), 5.91 (td, *J* = 4.1, 1.9 Hz, 1H), 2.31 – 2.27 (m, 2H), 2.23 – 2.18 (m, 2H), 1.78 – 1.71 (m, 2H), 1.69 – 1.63 (m, 2H).

(b) Competition experiments between **S1** and non-borylated substrates (following the standard procedure)

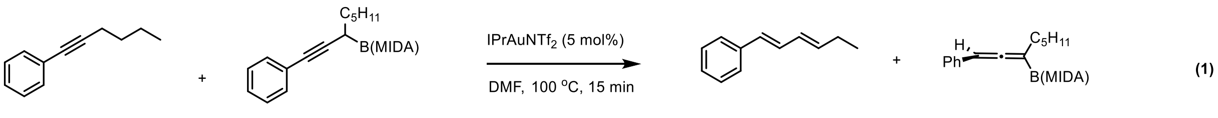

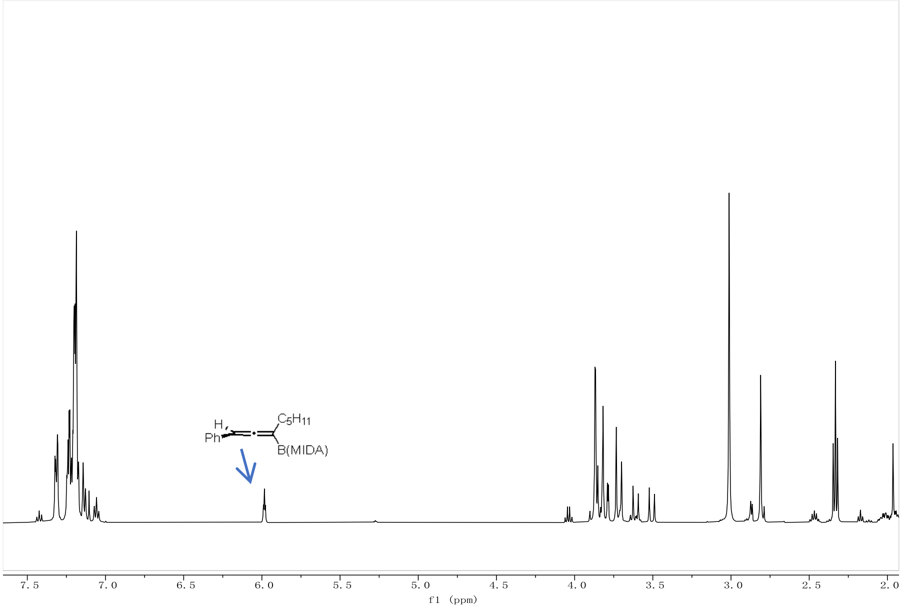

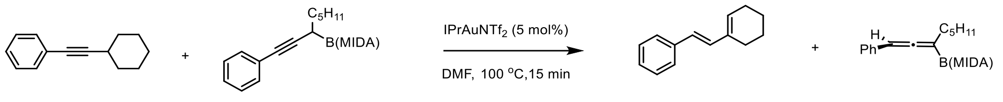

68 **S1** **69**, *trace* **1**, 22% (2)

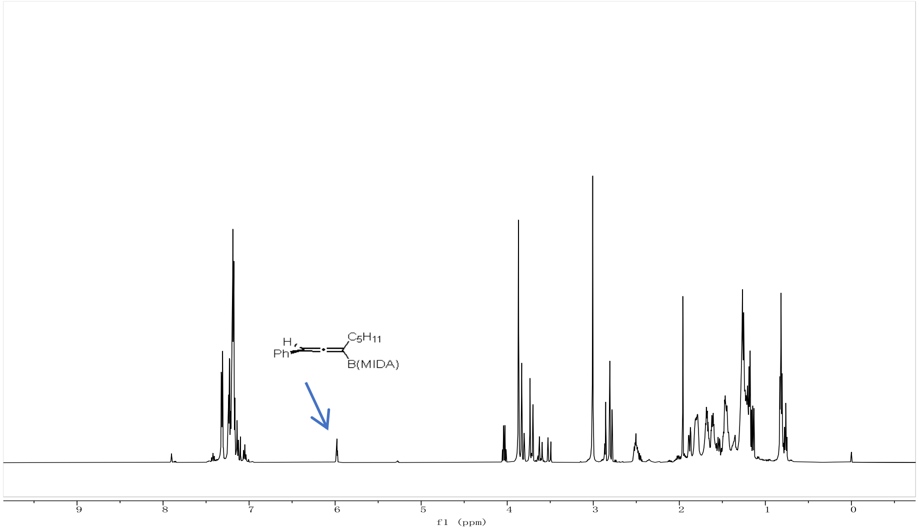

c) Competition experiments with substrates bearing different substituents

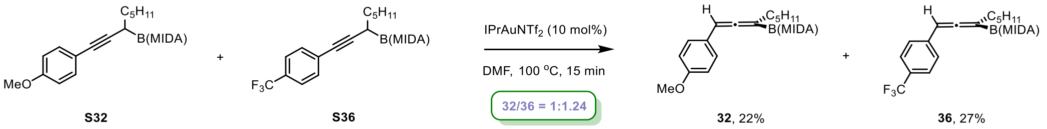

S32 **S36** **32**, 22% **36**, 27% (3)

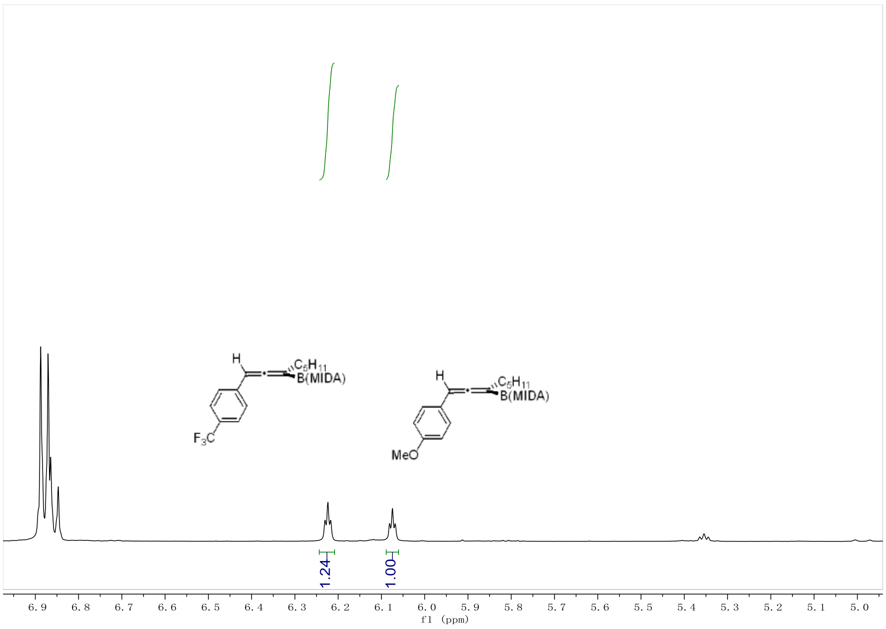

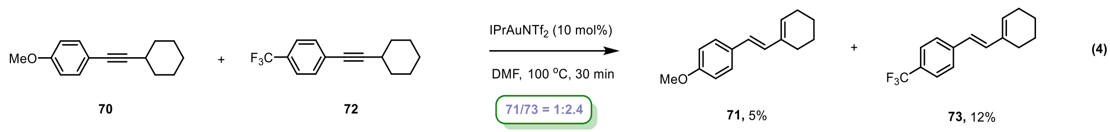
MeO
70
+
F3C
72
IPrAuNTf2 (10 mol%)
DMF, 100 °C, 30 min
71/73 = 1:2.4
MeO
71, 5%
+
F3C
73, 12%

(4)

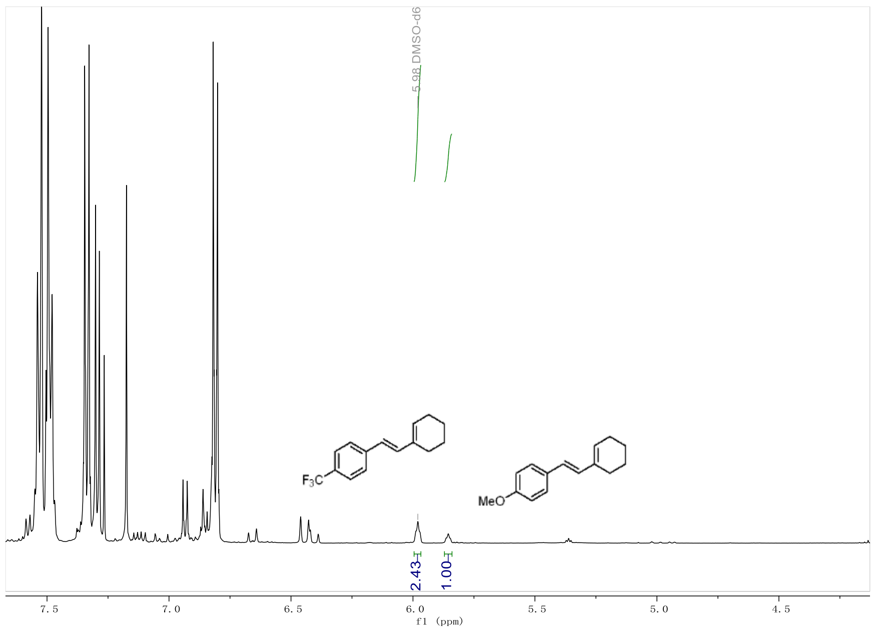
5.98 DMSO-d6
F3C
MeO
2.43
1.00
7.5
7.0
6.5
6.0
5.5
5.0
4.5
f1 (ppm)

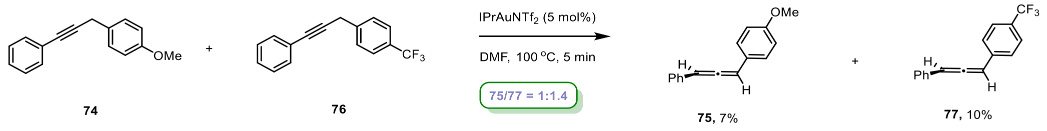
OMe
74
+
CF3
76
IPrAuNTf2 (5 mol%)
DMF, 100 °C, 5 min
75/77 = 1:1.4
OMe
H
Ph
H
75, 7%
+
CF3
H
Ph
H
77, 10%

(5)

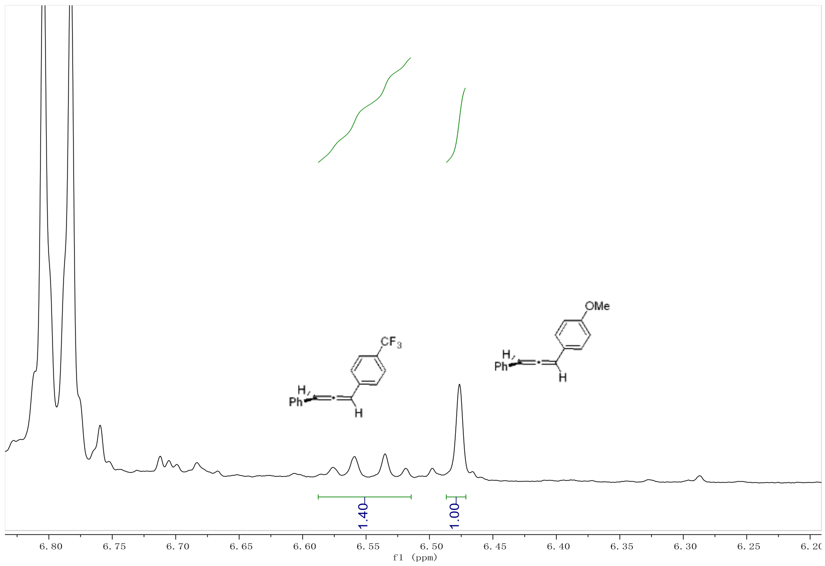
CF3
H
Ph
H
OMe
H
Ph
H
1.40
1.00
6.80
6.75
6.70
6.65
6.60
6.55
6.50
6.45
6.40
6.35
6.30
6.25
f1 (ppm)

d) Experiment using substate bearing OPh

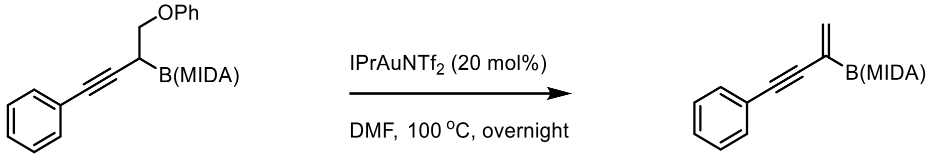

S78 **78**, 56%

6-methyl-2-(4-phenylbut-1-en-3-yn-2-yl)-1,3,6,2-dioxazaborocane-4,8-dione

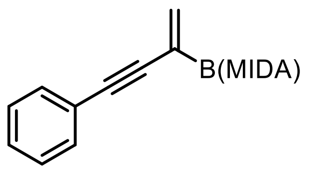

78

The product **78** was obtained in **56**% (16 mg) yield as a colorless semisolid after column chromatography (eluent = ethyl acetate/petroleum ether v/v 2:1)

^{1}H NMR (400 MHz, Chloroform-*d*) δ 7.40 – 7.35 (m, 2H), 7.33 – 7.28 (m, 3H), 6.11 (d, *J* = 3.3 Hz, 1H), 6.05 (d, *J* = 3.3 Hz, 1H), 3.97 (d, *J* = 16.5 Hz, 2H), 3.87 (d, *J* = 16.4 Hz, 2H), 2.97 (s, 3H).

^{13}C NMR (101 MHz, Acetone-*d$_6$*) δ 168.8, 133.4, 132.2, 129.4, 124.4, 93.0, 91.6, 63.1, 47.5.

HRMS (EI): calculated for $C_{15}H_{14}BNO_4$ [M], 283.1016; Found, 283.1010.

g) Reaction of Bpin and BF_3K

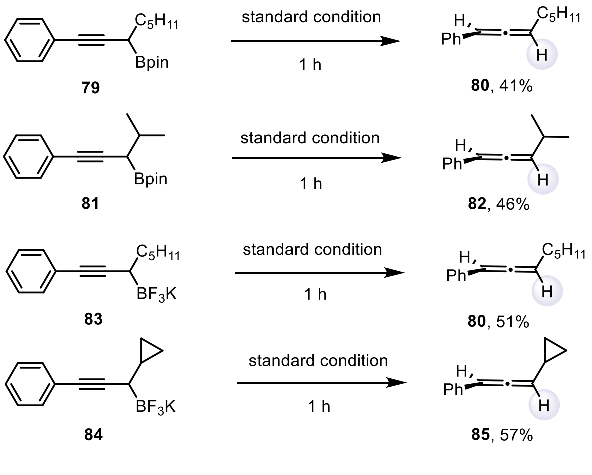

4,4,5,5-tetramethyl-2-(1-phenyloct-1-yn-3-yl)-1,3,2-dioxaborolane

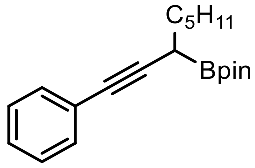

79

^{1}H NMR (400 MHz, Chloroform-*d*) δ 7.33 – 7.29 (m, 2H), 7.19 – 7.13 (m, 3H), 2.15 (t, *J* = 7.3 Hz, 1H), 1.59 (q, *J* = 7.6 Hz, 2H), 1.26 – 1.21 (m, 18H), 0.82 (t, *J* = 7.0 Hz, 3H).

^{13}C NMR (126 MHz, Chloroform-*d*) δ 131.8, 128.1, 127.2, 124.8, 91.1, 84.0, 83.2, 80.7, 31.7, 30.6, 28.8, 24.8, 24.7, 24.6, 22.7, 14.2.

HRMS (ESI): calculated for $C_{20}H_{29}O_2B$ $[M+H]^+$, 313.2333; Found, 313.2339.

4,4,5,5-tetramethyl-2-(4-methyl-1-phenylpent-1-yn-3-yl)-1,3,2-dioxaborolane

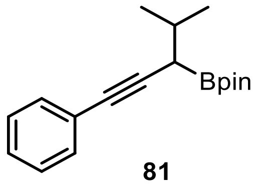

81

^{1}H NMR (500 MHz, Chloroform-*d*) δ 7.34 – 7.31 (m, 2H), 7.20 – 7.14 (m, 3H), 1.99 (dt, *J* = 19.6, 6.7 Hz, 2H), 1.21 (s, 12H), 1.02 (d, *J* = 6.4 Hz, 3H), 0.98 (d, *J* = 6.3 Hz, 3H).

^{13}C NMR (101 MHz, Chloroform-*d*) δ 131.8, 128.2, 127.2, 124.9, 89.9, 83.9, 81.8, 29.7, 24.8, 22.6, 21.7.

HRMS (ESI): calculated for $C_{18}H_{25}O_2B$ $[M+H]^+$, 285.2020; Found, 285.2023.

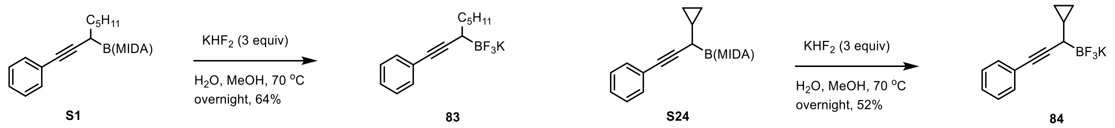

In a flask under nitrogen, B(MIDA) (**S1** or **S24**) (0.1 mmol, 1 equiv), KHF_2 (0.3 mmol, 3 equiv, 23.4 mg) and H_2O (3 M,1 mL) were added to MeOH (1.0 mL). The reaction mixture was stirred at 70 °C until total consumption of the starting material, as monitored by TLC. The solvent was removed under vacuo. Inorganic solids were removed via trituration with acetone, followed by filtration. Side product was removed via trituration with diethyl ether, affording the desired trifluoroborate salt as a yellow solid

trifluoro(1-phenyloct-1-yn-3-yl)borate potassium(I)

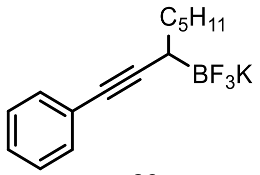

83

^{1}H NMR (400 MHz, DMSO-*d6*) δ 7.32 – 7.15 (m, 5H), 1.55 – 1.22 (m, 9H), 0.86 (t, *J* = 6.7 Hz, 3H).

^{13}C NMR (126 MHz, DMSO-*d6*) δ 130.7, 128.2, 126.2, 125.9, 101.6, 77.9, 31.6, 30.4, 29.0, 22.2, 14.1.

^{19}F NMR (376 MHz, DMSO-*d6*) δ -141.28.

^{11}B NMR (128 MHz, DMSO-*d6*) δ 3.50.

HRMS (ESI): calculated for $C_{14}H_{17}BF_3K$ [M-K]$^-$, 253.1381; Found, 253.1384.

(1- cyclopropyl-3-phenylprop-2-yn-1-yl)trifluoroborate potassium(I)

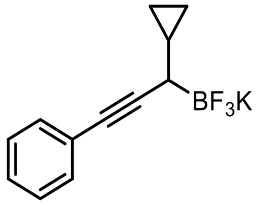

84

^{1}H NMR (400 MHz, DMSO-*d6*) δ 7.30 – 7.16 (m, 1H), 1.22 (d, *J* = 14.1 Hz, 1H), 0.82 (tq, *J* = 8.1, 5.6 Hz, 1H), 0.25 – 0.10 (m, 4H).

^{13}C NMR (101 MHz, DMSO-*d6*) δ 128.2, 126.1, 126.0, 99.0, 78.1, 11.3, 3.7, 3.2.

^{11}B NMR (128 MHz, DMSO-*d6*) δ 3.36.

^{19}F NMR (376 MHz, DMSO-*d6*) δ -140.23.

HRMS (ESI): calculated for $C_{12}H_{11}BF_3K$ [M-K]$^-$, 223.0911; Found, 223.0911.

(*R*)-octa-1,2-dien-1-ylbenzene

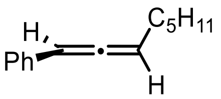

80

1H NMR (400 MHz, Chloroform-d) δ 7.32 – 7.28 (m, 2H), 7.21 – 7.15 (m, 1H), 6.14 – 6.10 (m, 1H), 5.59 – 5.54 (m, 1H), 2.16 – 2.10 (m, 2H), 1.48 (q, *J* = 7.1 Hz, 2H), 1.39 – 1.31 (m, 4H), 0.89 (t, *J* = 7.1 Hz, 3H).

(*R*)-(4-methylpenta-1,2-dien-1-yl)benzene

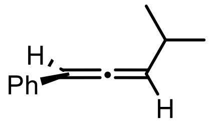

82

^{1}H NMR (400 MHz, Chloroform-d) δ 7.30 – 7.28 (m, 4H), 7.20 – 7.15 (m, 1H), 6.17 (dd, *J* = 6.4, 3.1 Hz, 1H), 5.59 (t, *J* = 6.1 Hz, 1H), 2.49 – 2.42 (m, 1H), 1.10 (dd, *J* = 6.7, 3.0 Hz, 6H).

(*R*)-(3-cyclopropylpropa-1,2-dien-1-yl)benzene

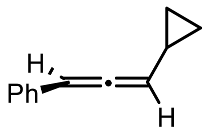

85

^{1}H NMR (400 MHz, Chloroform-d) δ 7.29 (d, *J* = 5.0 Hz, 4H), 7.22 – 7.16 (m, 1H), 6.20 (d, *J* = 6.4 Hz, 1H), 5.45 (t, *J* = 6.9 Hz, 1H), 1.37 (d, *J* = 7.6 Hz, 1H), 0.78 – 0.73 (m, 2H), 0.49 – 0.42 (m, 2H).

h) Deuterium scrambling experiment

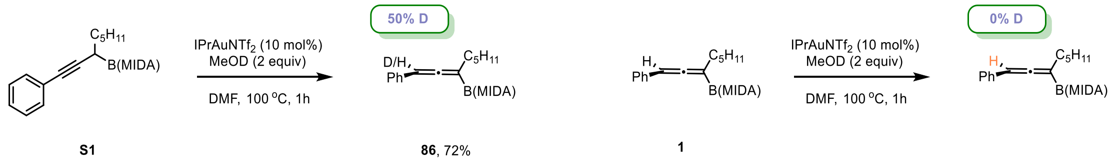

In a glove box, a 5 mL Schleck tube equipped with a stirrer bar was charged with starting material (**S1** or **1**) (0.1 mmol, 1.0 equiv), $IPrAuNTf_2$ (0.01 mmol, 10 mol%), MeOD (0.2 mmol, 2.0 equiv) and DMF (1 mL). The tube was sealed and heated at 100 ºC with stirring for 1 hour. The reaction mixture was diluted with EA (20 mL) and water (10 mL). The organic phase was separated and the aqueous layer was extracted with EA (20 mL) for three times. The combined organic layer was dried over anhydrous Na_2SO_4 and concentrated under reduced pressure. The crude residue was purified by flash column (EA/PE as the eluent) to afford the products.

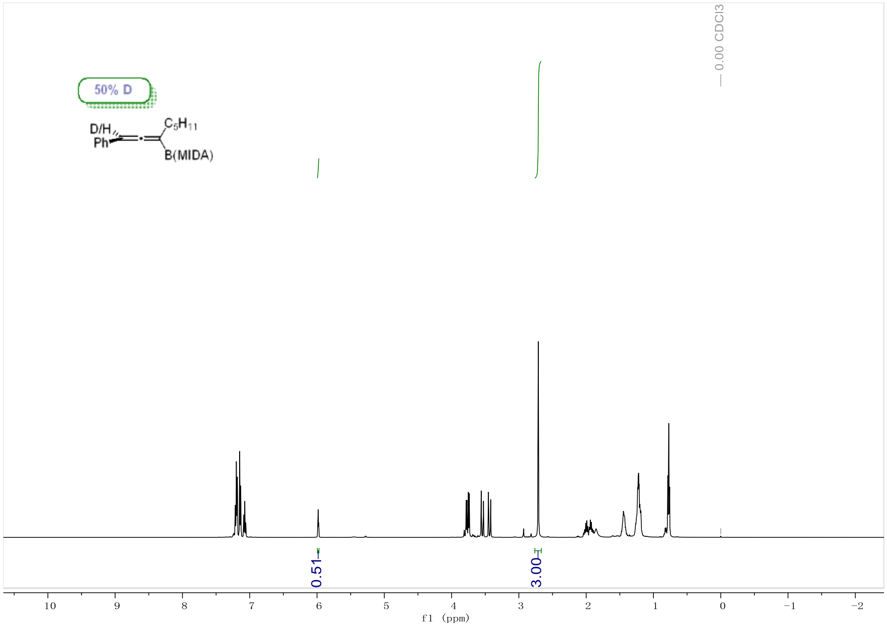

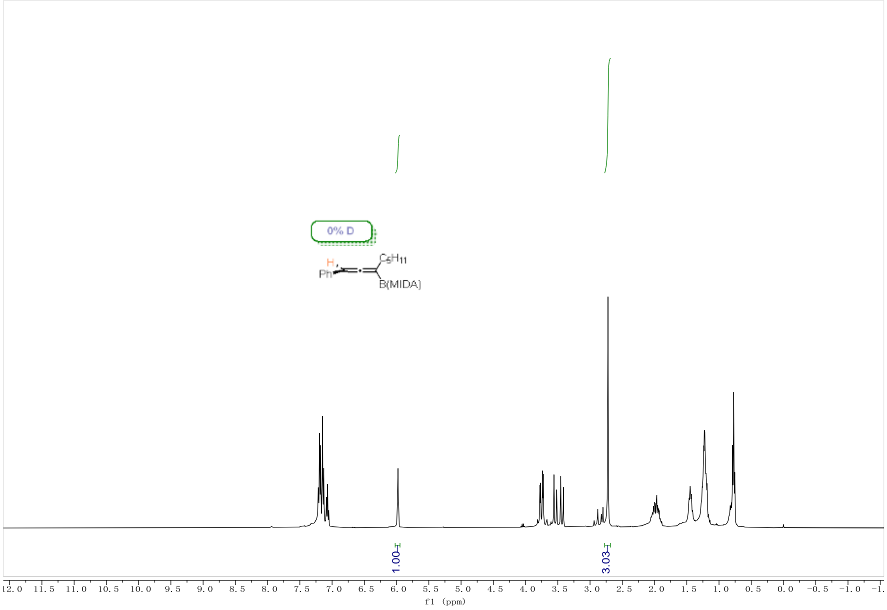

6. DFT studies

Method:

The calculation of pKa refers to the thermodynamic cycle process mentioned in the research of Matthew D. Liptak and George C. Shields,[25] as illustrated in Figure S1. The dissociation free energy of an acid in solvent, denoted as ΔG_{aq}^{1M} , can be derived from other relevant energies in the thermodynamic cycle, specifically:

$$\Delta G_{aq}^{1M} = \Delta G_{gas}^{1atm} + \Delta G_{solv}^{mod}(A^-) + \Delta G_{solv}^{mod}(H^+) - \Delta G_{solv}^{mod}(AH) + \Delta G^{1atm\rightarrow 1M} \quad (1)$$

Where the ΔG_{solv}^{mod} represents solvation free energy of substances; $\Delta G^{1atm\rightarrow 1M}$ represents the change in free energy when transitioning from the standard state in the gas phase to the standard state in the solution phase, which amounts to 1.89 kcal/mol.[26] The change in free energy under gas phase is determined using a composite method, which is:

$$\Delta G_{gas}^{1atm} = \Delta G_{gas}^{1atm}(A^-) + \Delta G_{gas}^{1atm}(H^+) - \Delta G_{gas}^{1atm}(AH) \quad (2)$$

Where the exact value of $\Delta G_{gas}^{1atm}(H^+)$ is -6.28 kcal/mol,[25] while the free energy of proton solvation is assumed to be -265.9 kcal/mol.[27] The relationship between *p*Ka and ΔG_{aq}^{1M} is :

$$\mathrm{pKa} = \frac{\Delta G_{aq}^{1M}}{2.303\mathrm{RT}} \quad (3)$$

All structures involved in this study were first optimized using the B3LYP[28]/6-31G** level of theory. Following this, single-point energy calculations were performed with the M05-2X[29]/6-31G* method, both in the gas phase and under the SMD implicit solvent model, to calculate the solvation free energy.[30] The choice of M05-2X/6-31G* for these calculations is due to its demonstrated suitability for estimating solvation free energy,[30] where the solvation free energy is determined by the difference in single-point

energies between the gas phase and solution phase. For accurately calculating free energy in the gas phase, the M062X[31]/6-311+G(2d,p) method was employed for calculating the single-point energy of the acid/base pair, followed by thermodynamic corrections using the Shermo software.[32] These corrections utilized frequency data obtained at the B3LYP/6-31G** level of theory. All calculations were carried out using the Gaussian 09 program.[33] To test the method's accuracy, cyanacetic acid was initially examined, yielding a computed pKa value of 2.55. This value closely aligns with the experimental value of 2.6 ± 0.1,**[34]** demonstrating the method’s reasonable approximation and underscoring its potential utility.

To evaluate the relative free energy of compounds in a solvent, single-point energy calculations are performed using the M06[31]/6-311++G(d,p) method with the SMD solvation model, employing *N*-Dimethylformamide as the solvent. Thermodynamic corrections are carried out using the same methodology as used in the calculation of *p*Ka, with the temperature set at 373.15K.

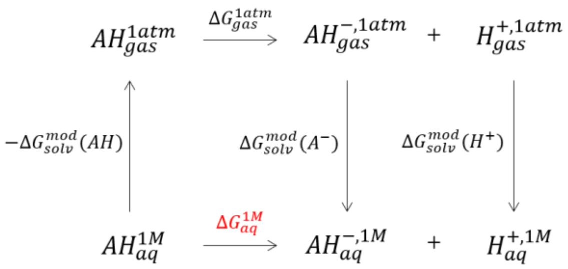

Figure S1. The thermodynamic cycle involved in the calculation of *p*Ka.

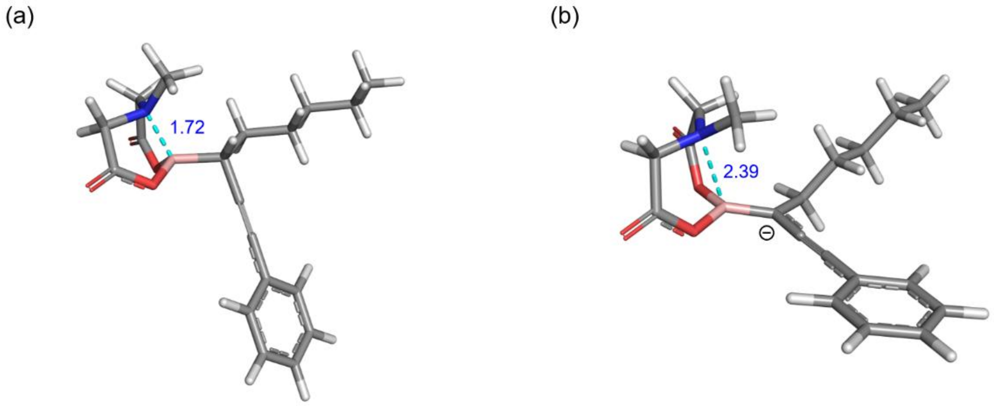

Figure S2. The structures of compound **S1** (a) and its conjugate base (b). In **S1**, the distance between the electron-rich N and the electron-deficient B is 1.72Å. However, in its conjugate base, this distance is elongated to 2.39Å. This elongation may be due to the elimination of a proton leading to the involvement of B in conjugation, resulting in increased planarity of the molecule and additional stability.

entry	substrate	*pKa*
1	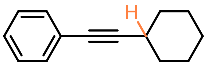	36.6
2	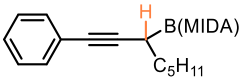	14.9

Figure S3. Calculated *pKa* values

entry	alkyne	allene	ΔG (kcal/mol)
1	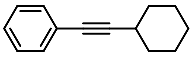	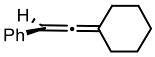	0.1
2	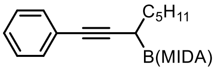	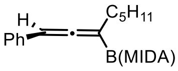	-2.1
3	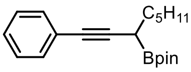	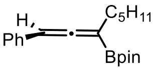	-3.1

Figure S4. Relative stabilities of alkynes/allenes

7. NMR spectra

S1: ^{1}H NMR (400 MHz, DMSO-*d6*)

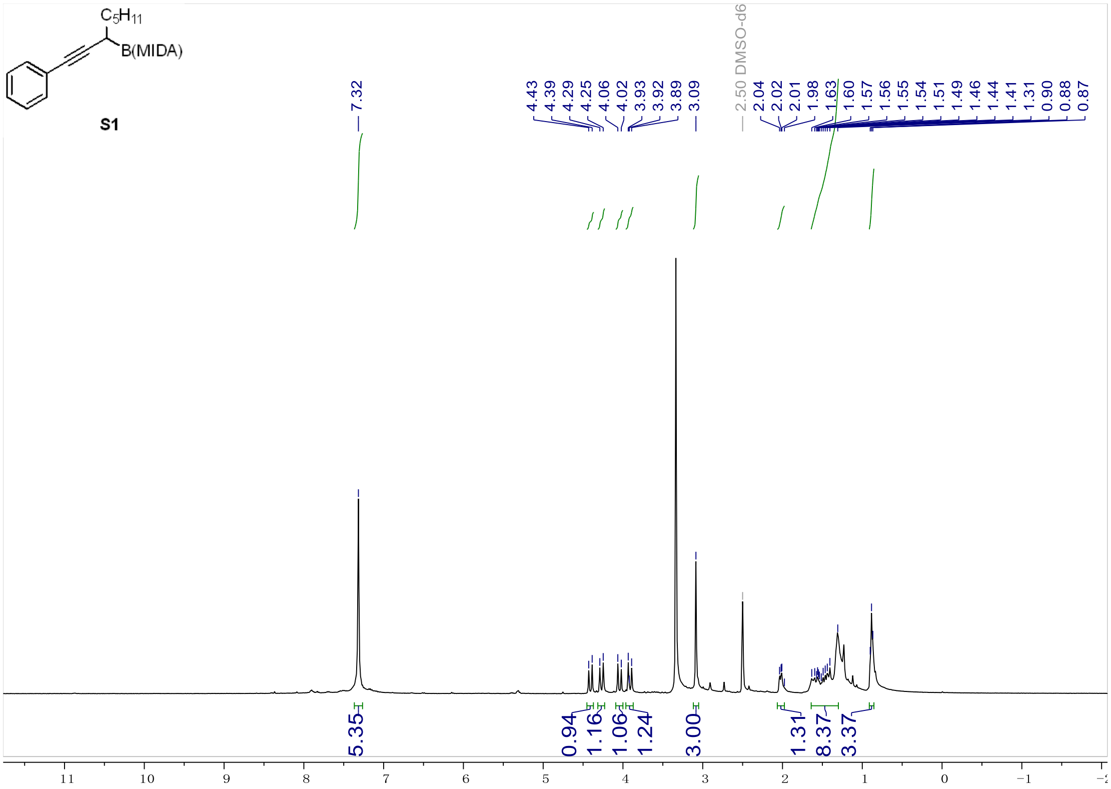

S1: ^{13}C NMR (101 MHz, DMSO-*d6*)

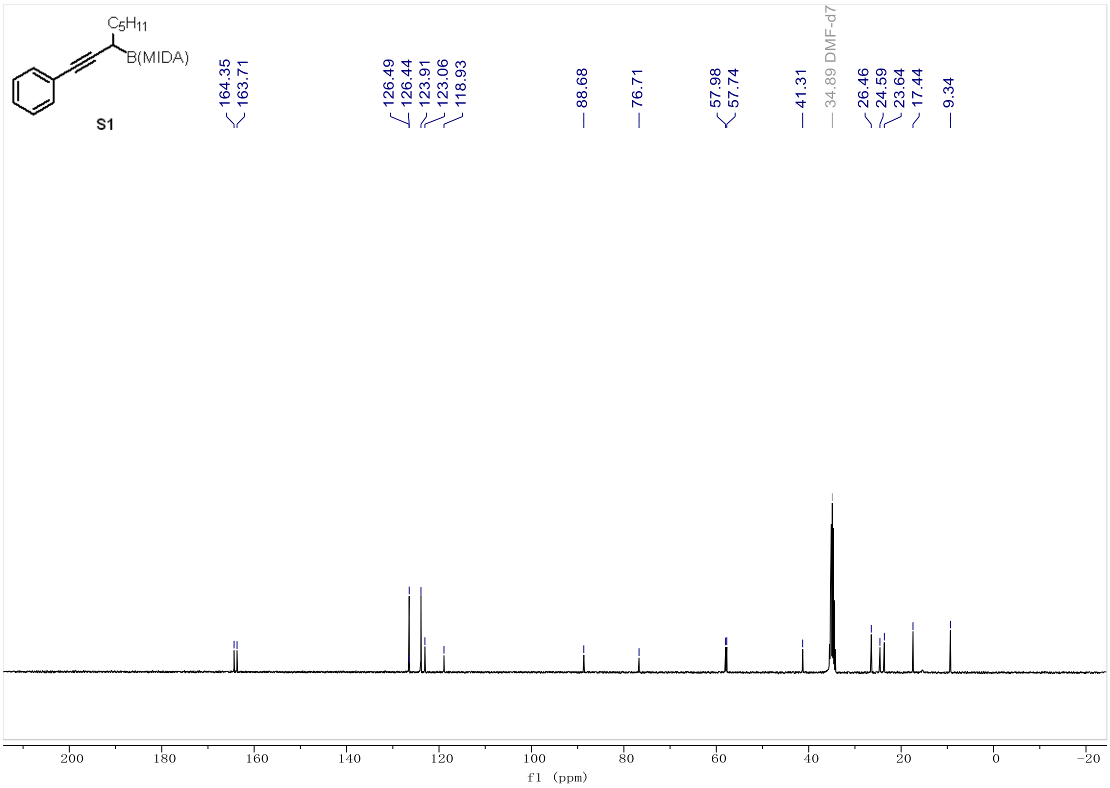

S2: ^{1}H NMR (400 MHz, DMSO-d_6)

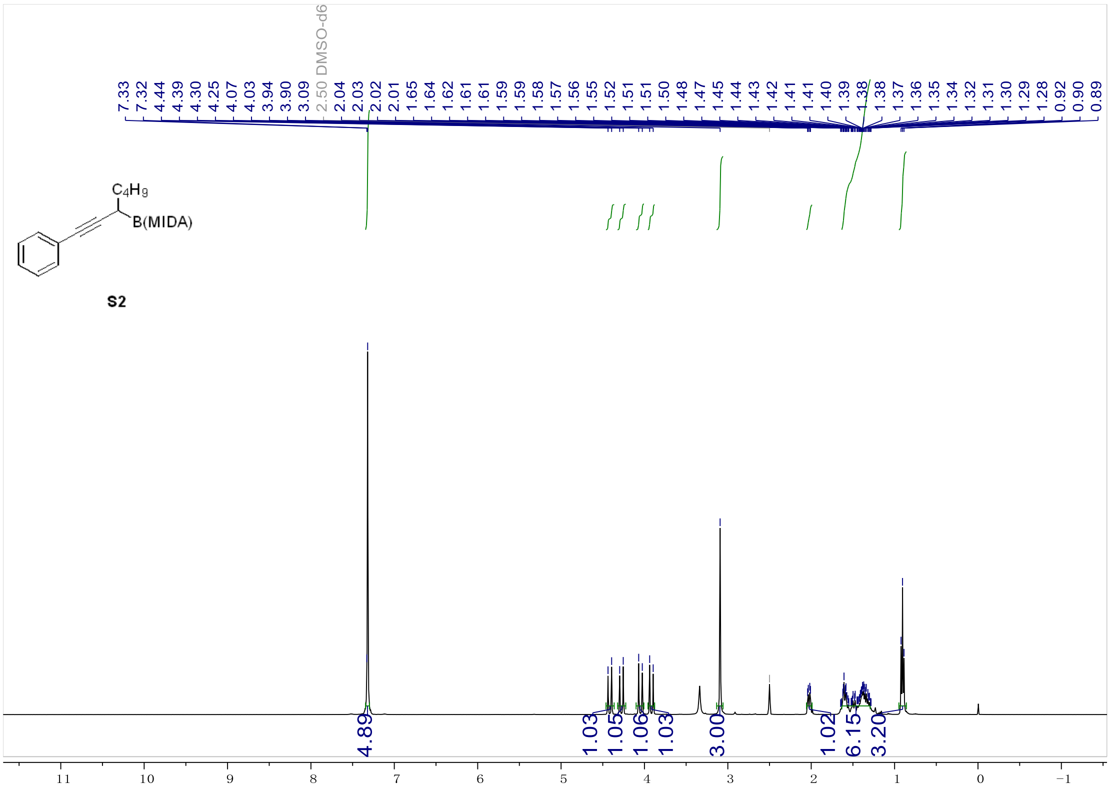

S2: ^{13}C NMR NMR (101 MHz, DMSO-d_6)

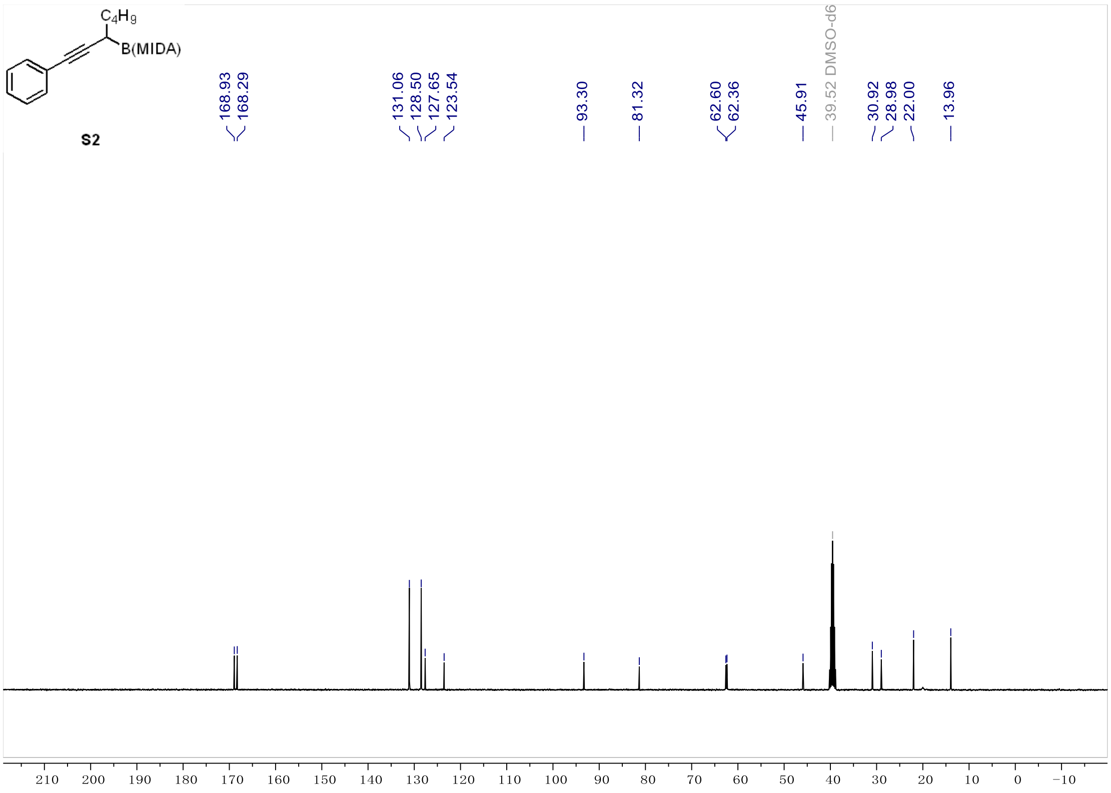

S3: ^{1}H NMR (400 MHz, DMSO-d_6)

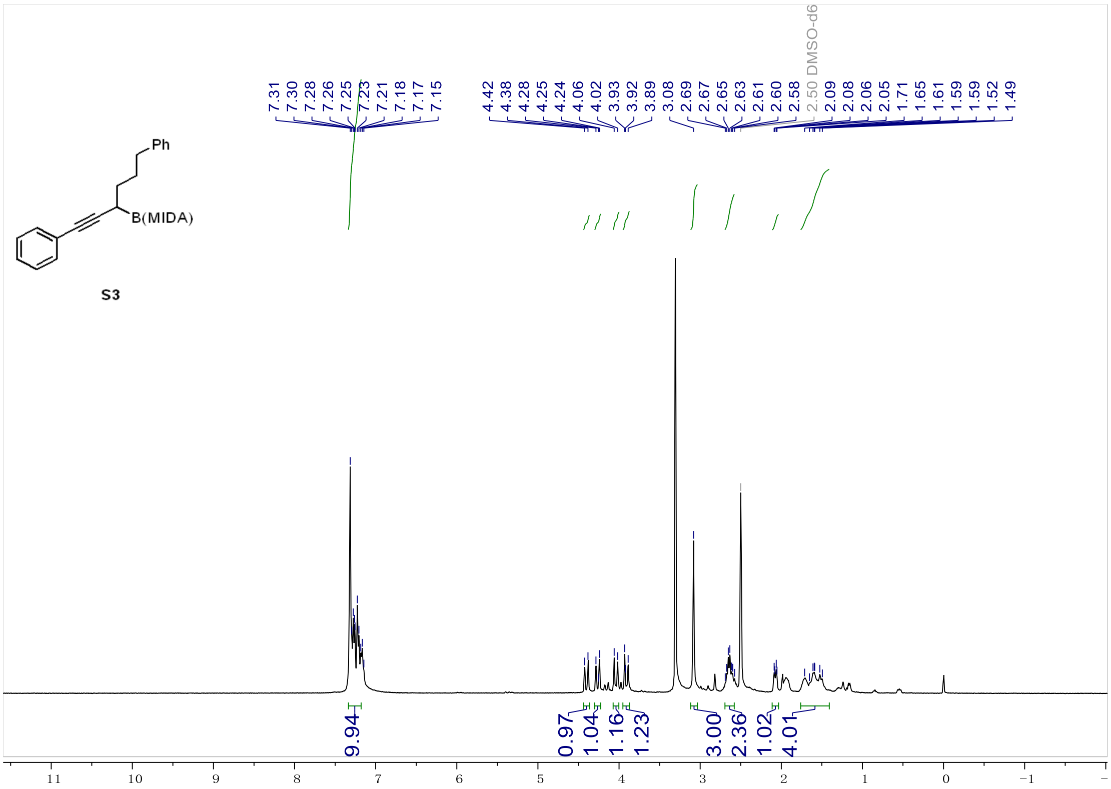

S3: ^{13}C NMR (101 MHz, DMSO-d_6)

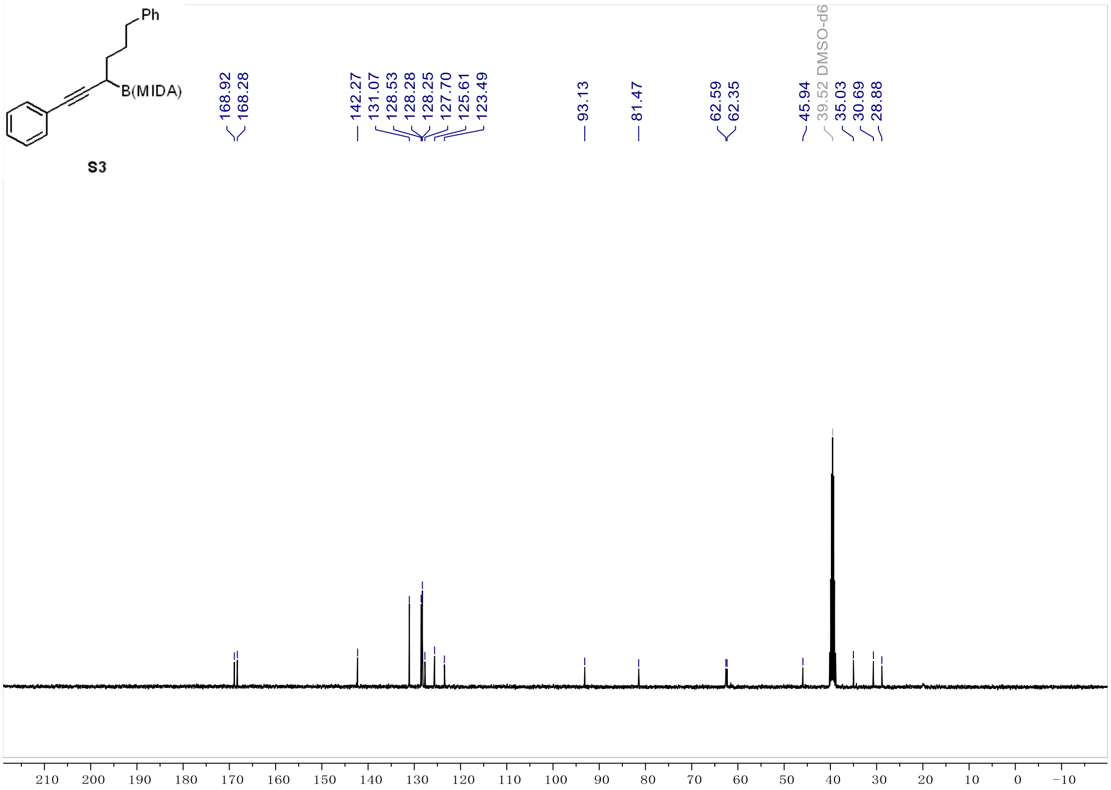

S4: ^{1}H NMR (400 MHz, DMSO-d_6)

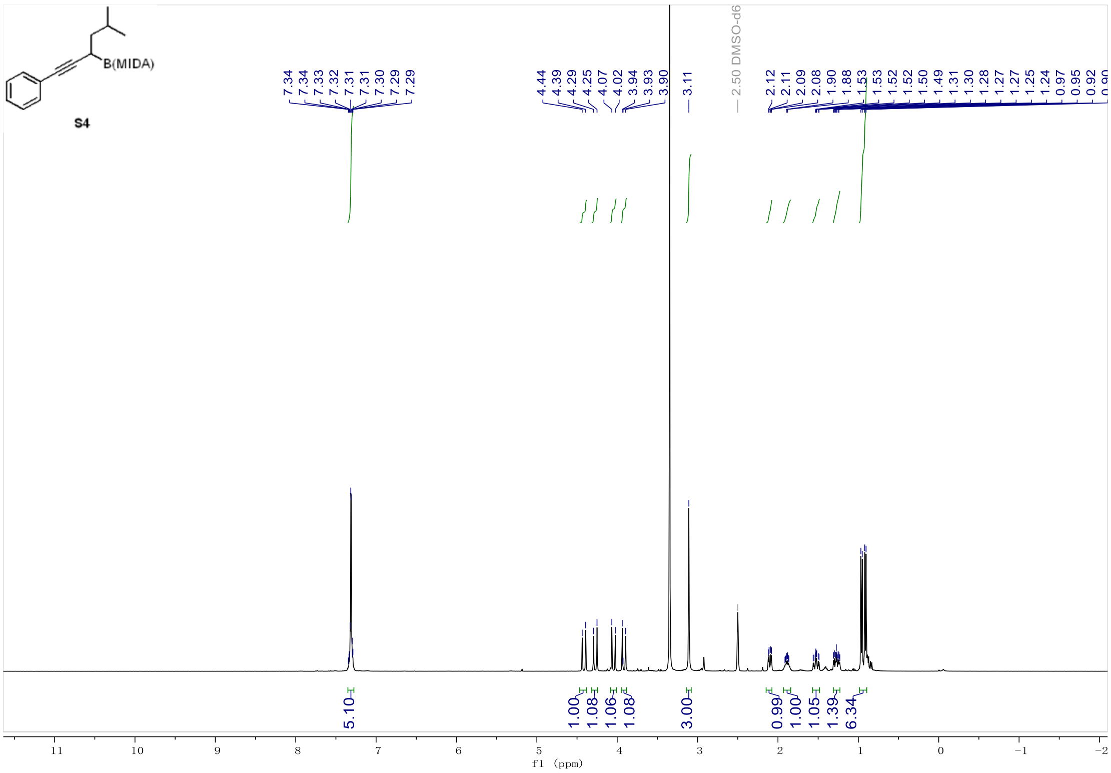

S4: ^{13}C NMR (126 MHz, DMSO-d_6)

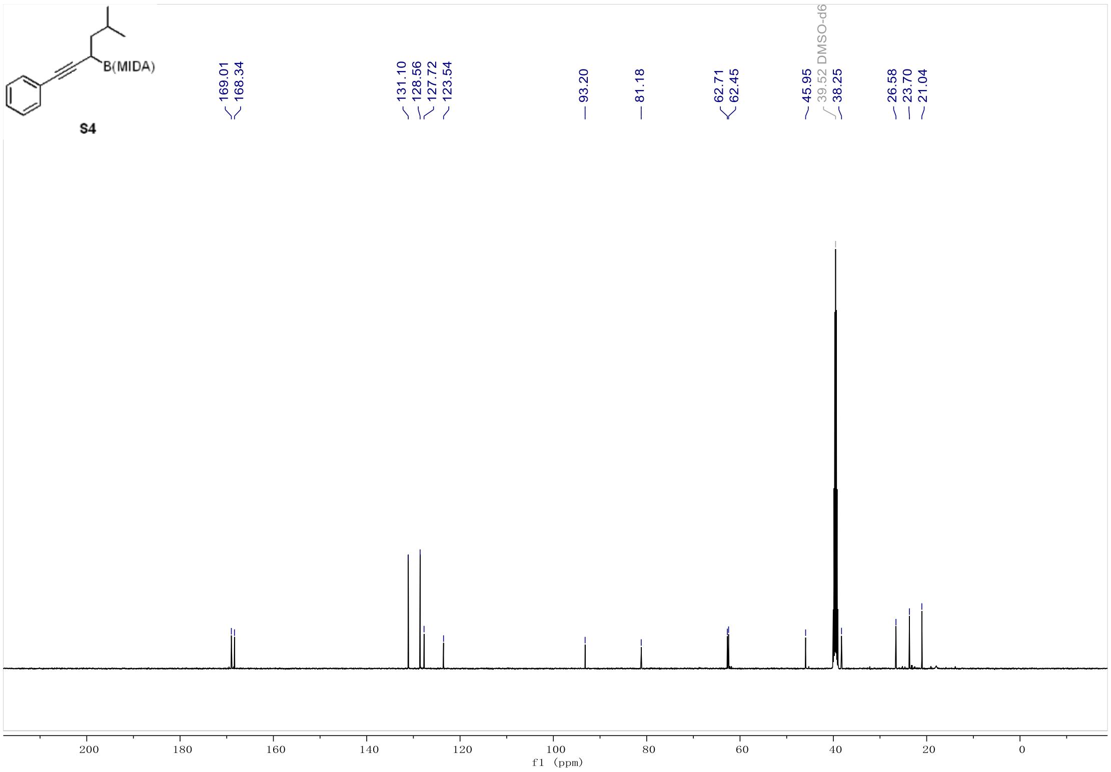

S5: ^{1}H NMR (400 MHz, Chloroform-*d*)

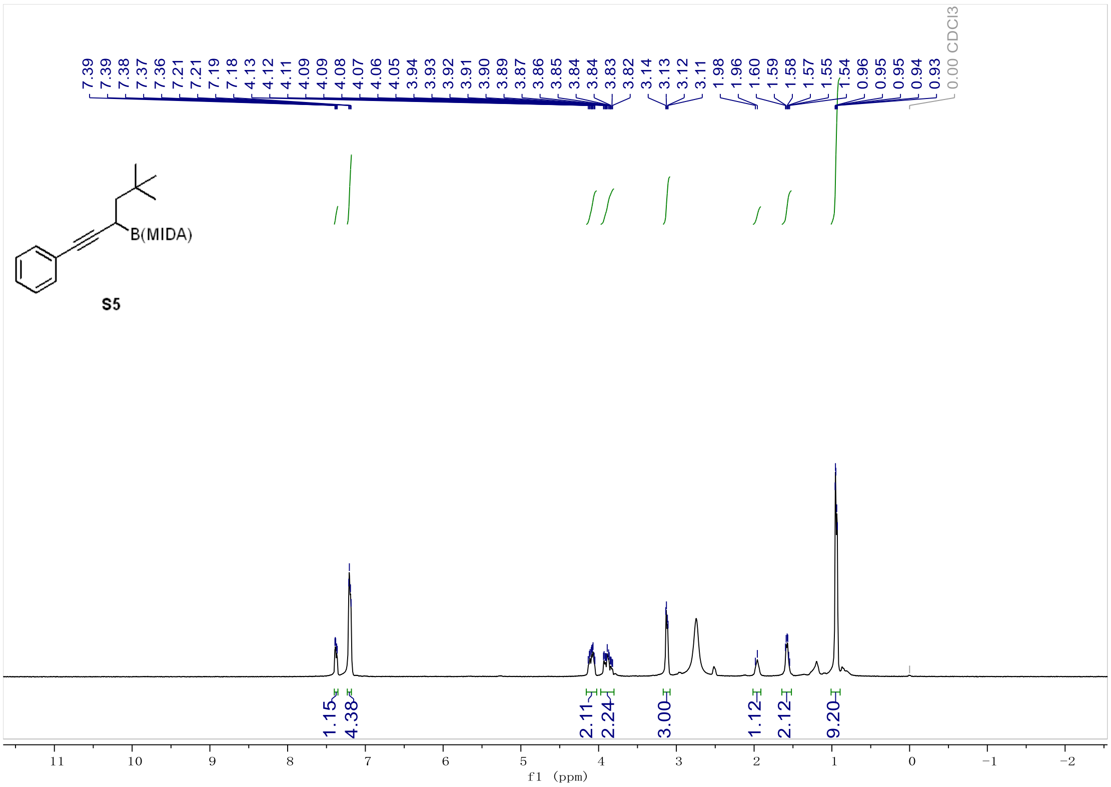

S5: ^{13}C NMR (101 MHz, DMSO-d_6)

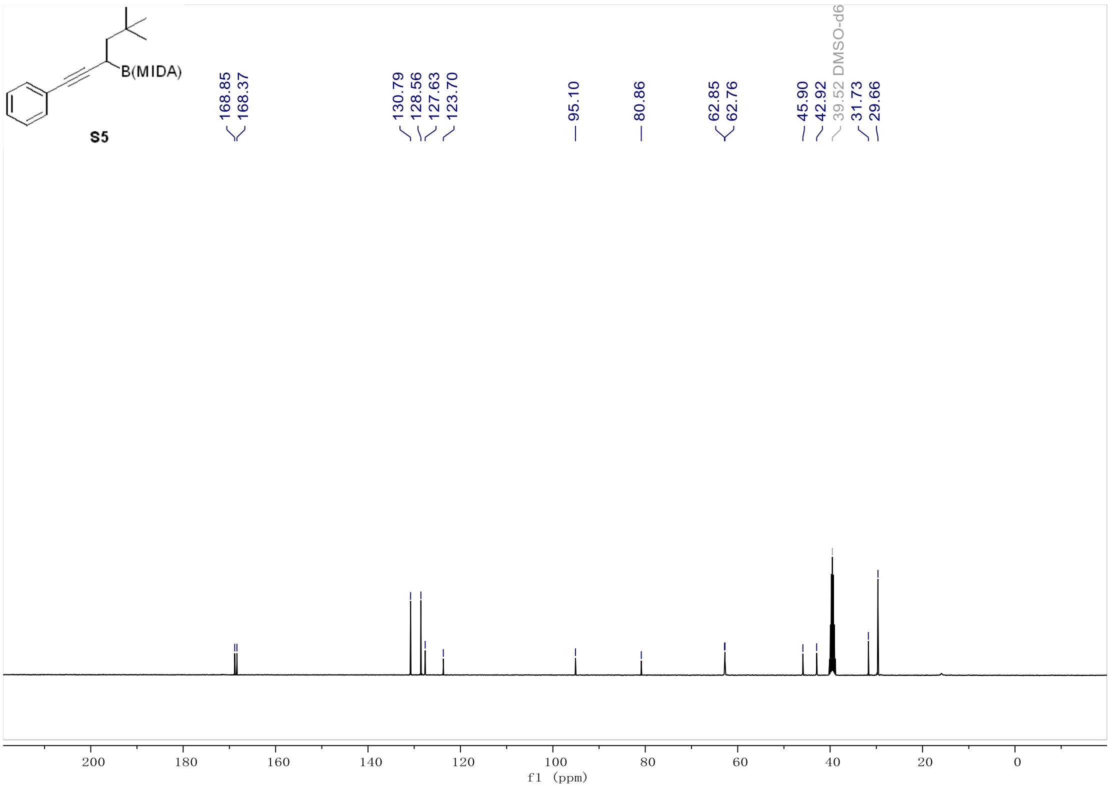

S6: ¹H NMR (500 MHz, DMSO-d_6)

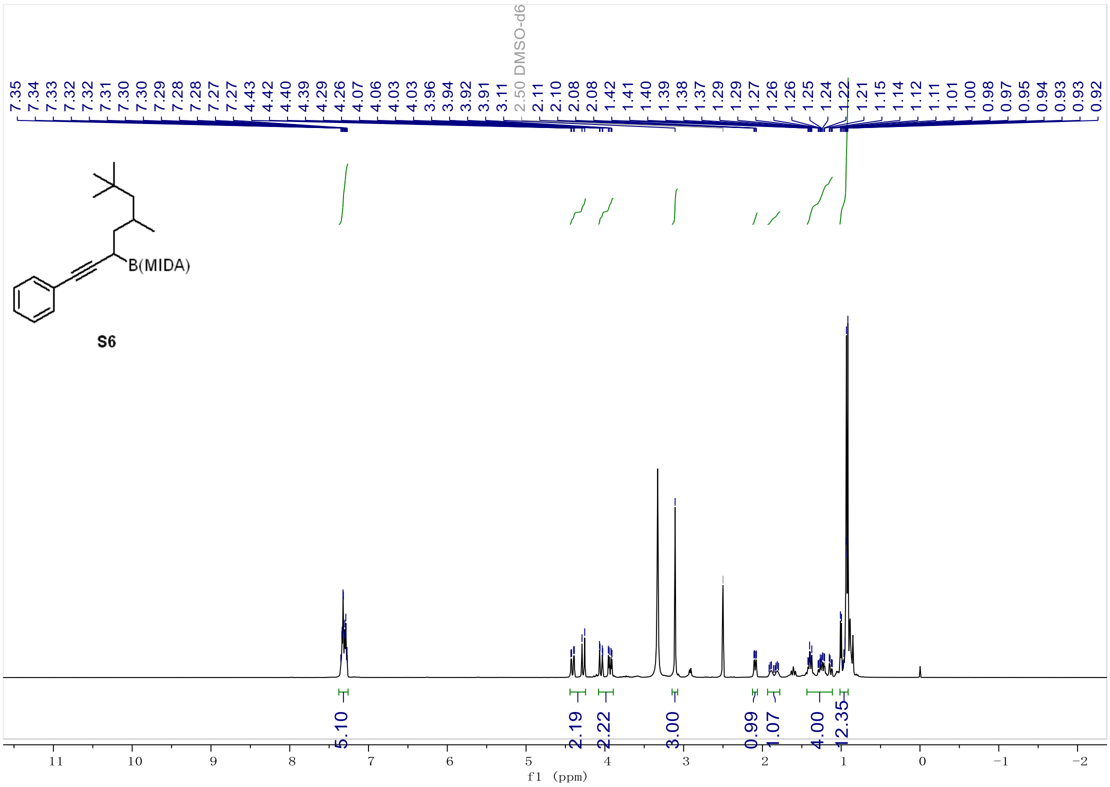

S6: ¹³C NMR (126 MHz, DMSO-d_6)

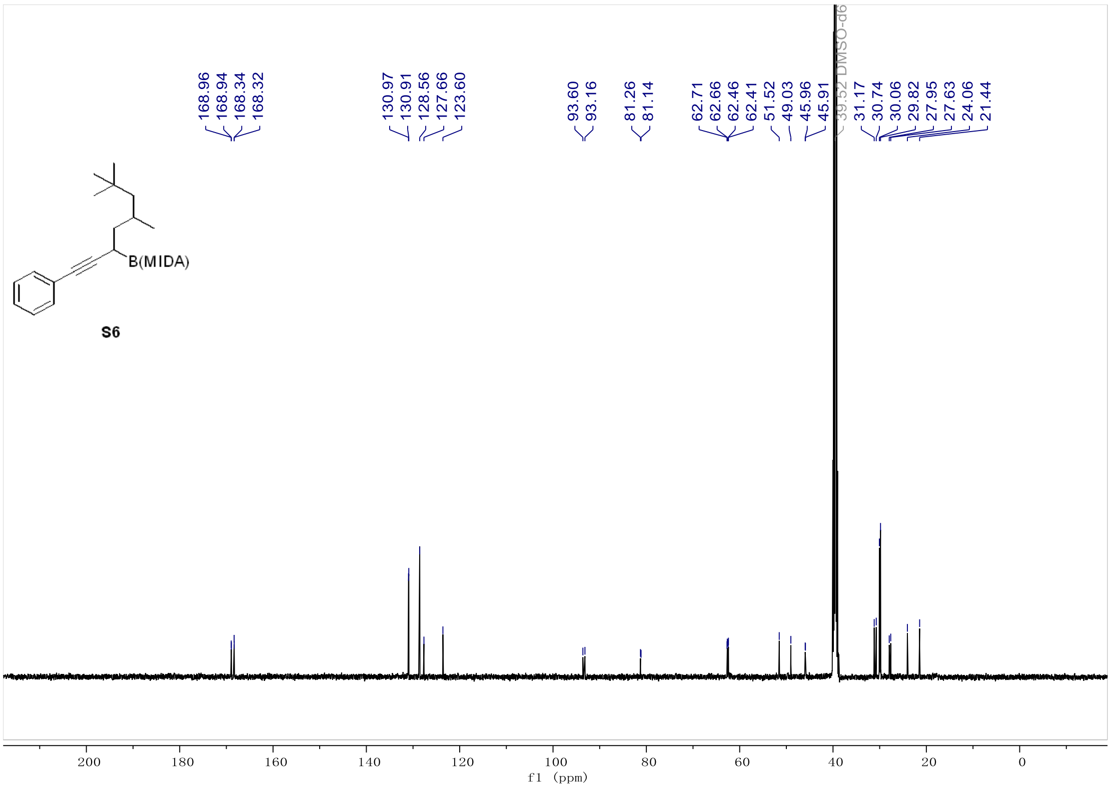

S7: ^{1}H NMR (400 MHz, DMSO-d_6)

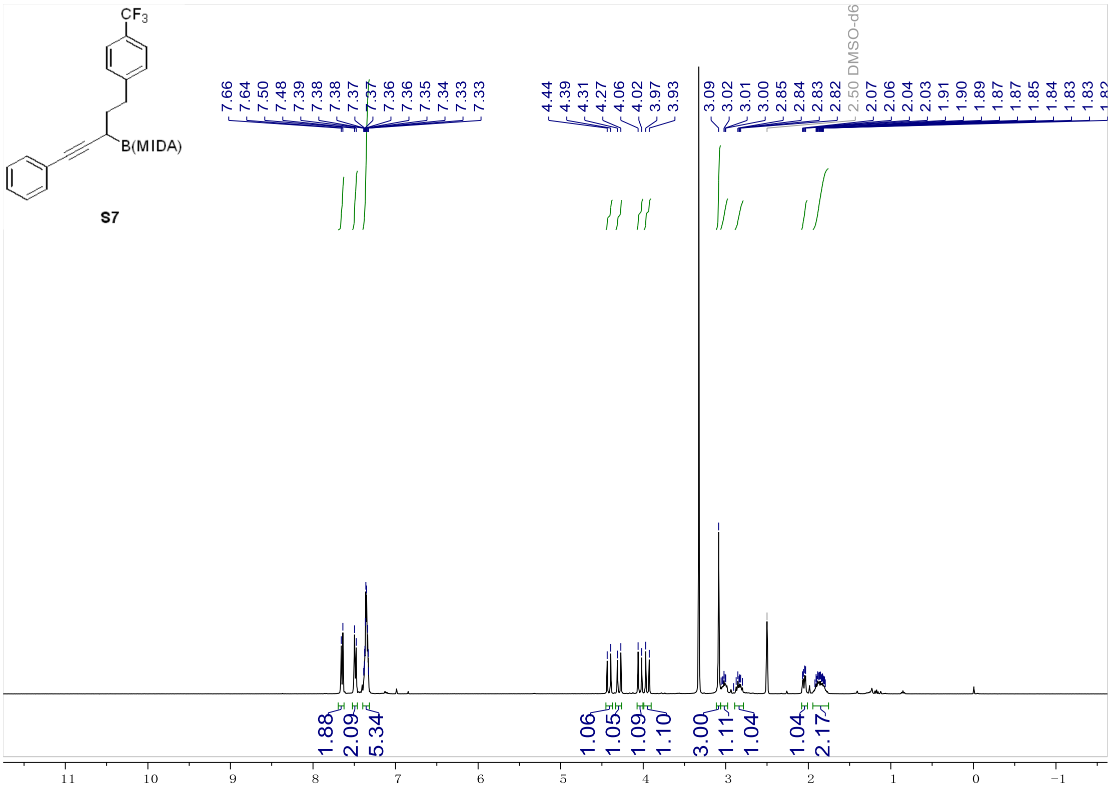

S7: ^{13}C NMR (101 MHz, DMSO- d_6)

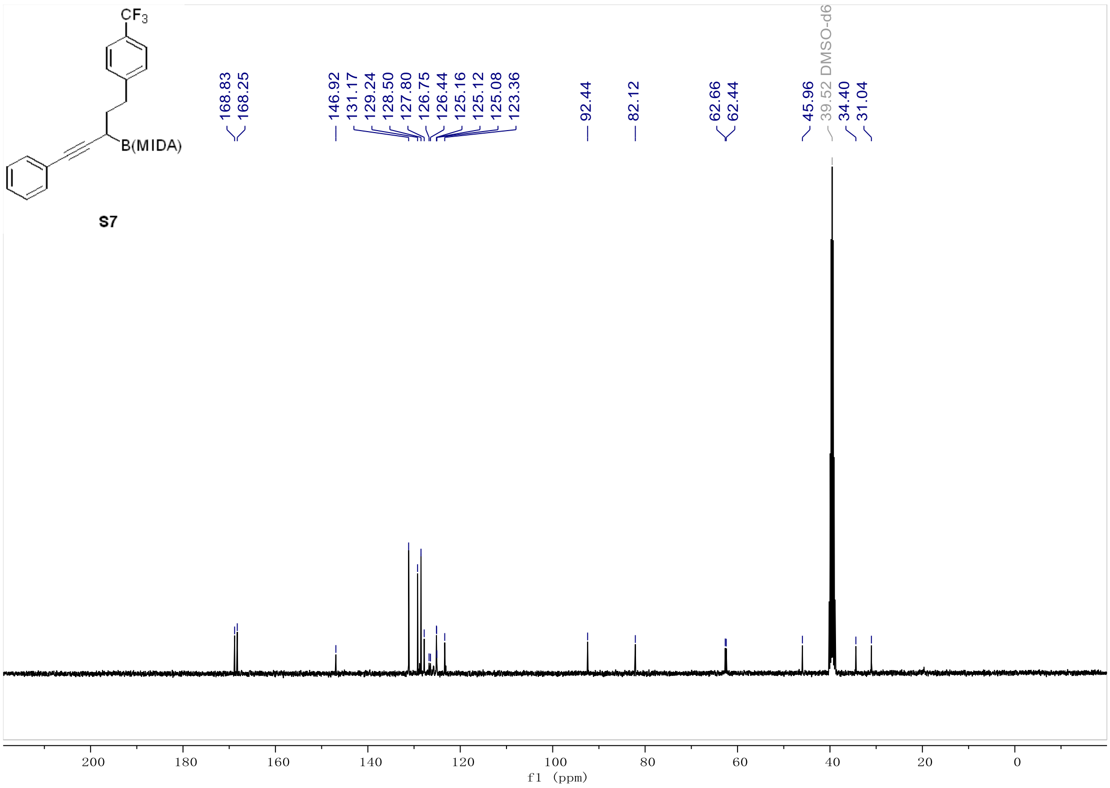

S8: ^{1}H NMR (400 MHz, DMSO-*d₆*)

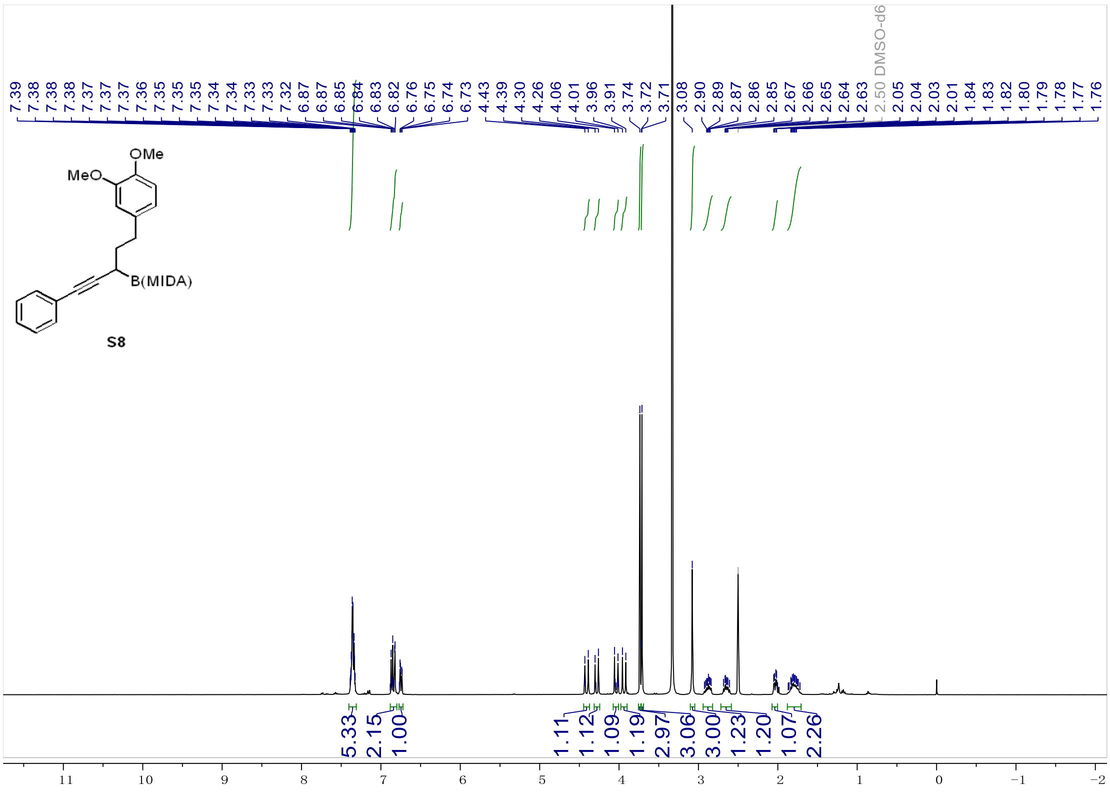

S8: ^{13}C NMR (126 MHz, Chloroform-*d*)

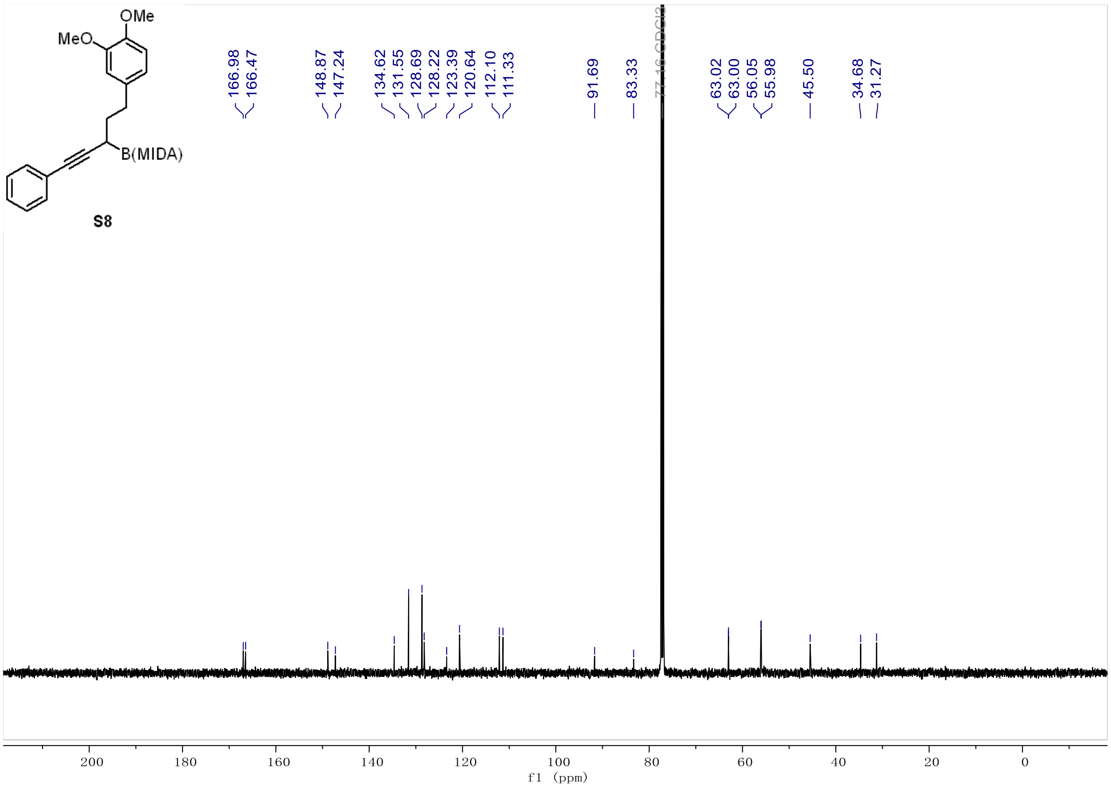

S9: ^{1}H NMR (500 MHz, DMSO-d_6)

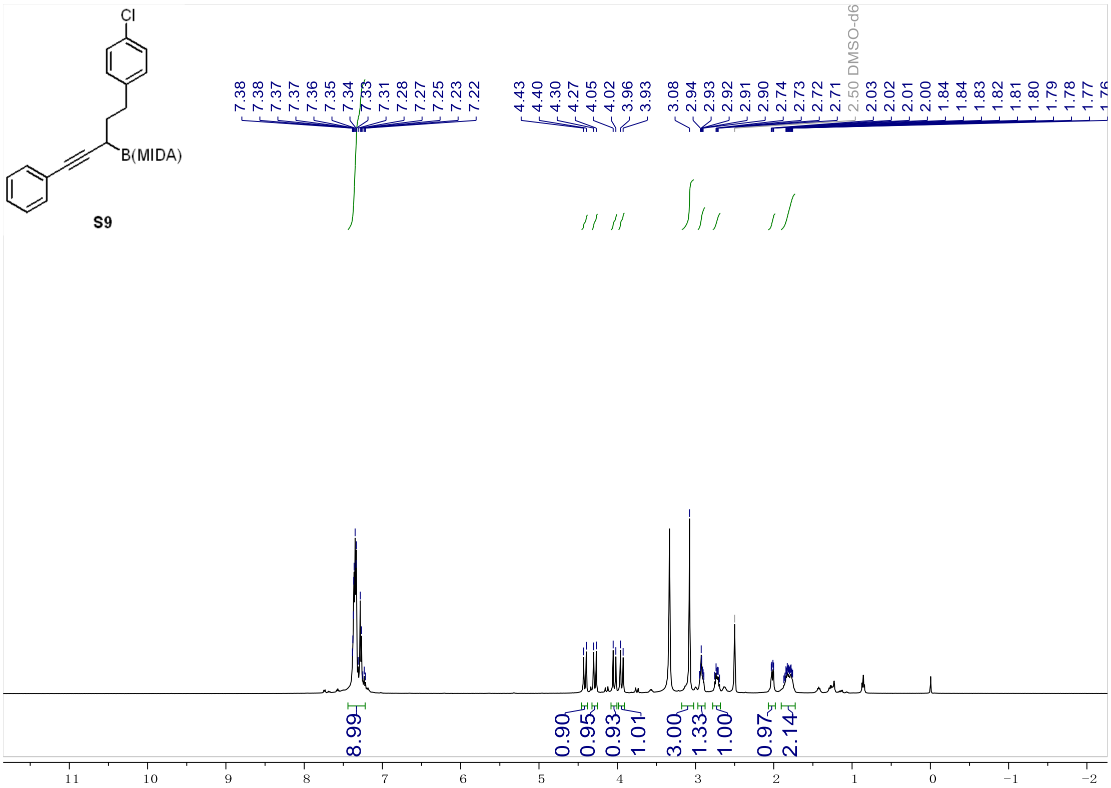

S9: ^{13}C NMR (126 MHz, DMSO-d_6)

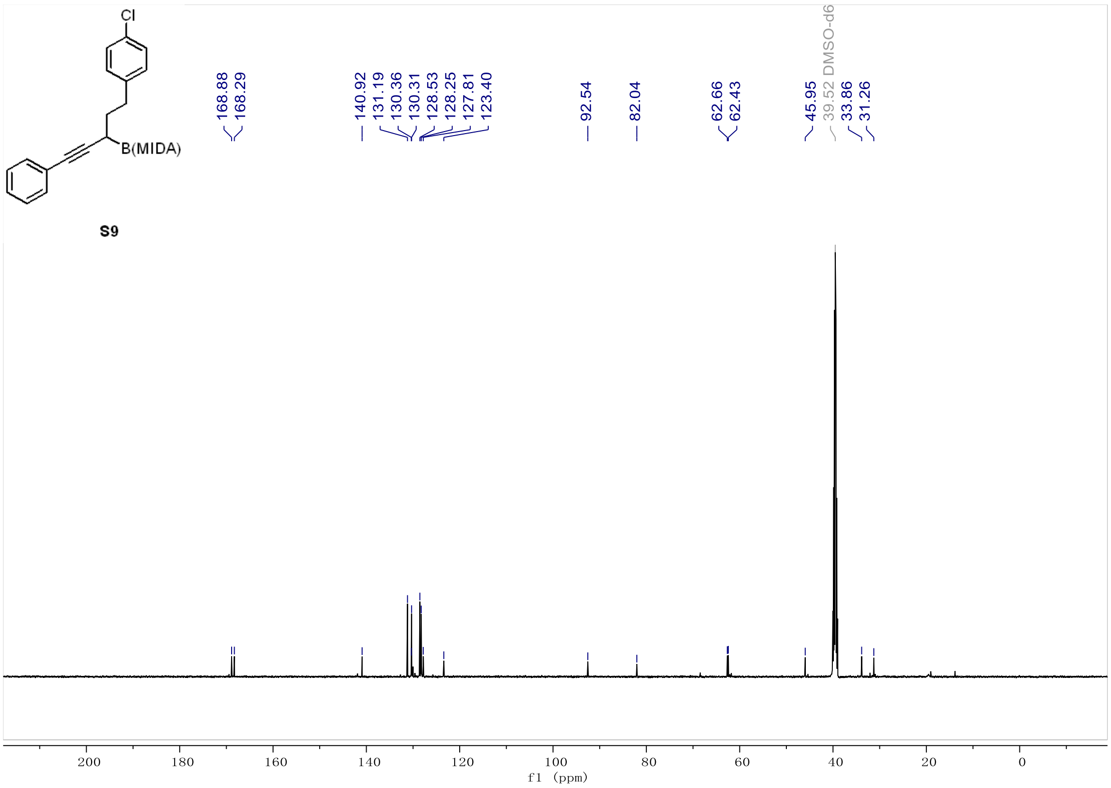

S10: ^{1}H NMR (400 MHz, Acetonitrile-*d₃*)

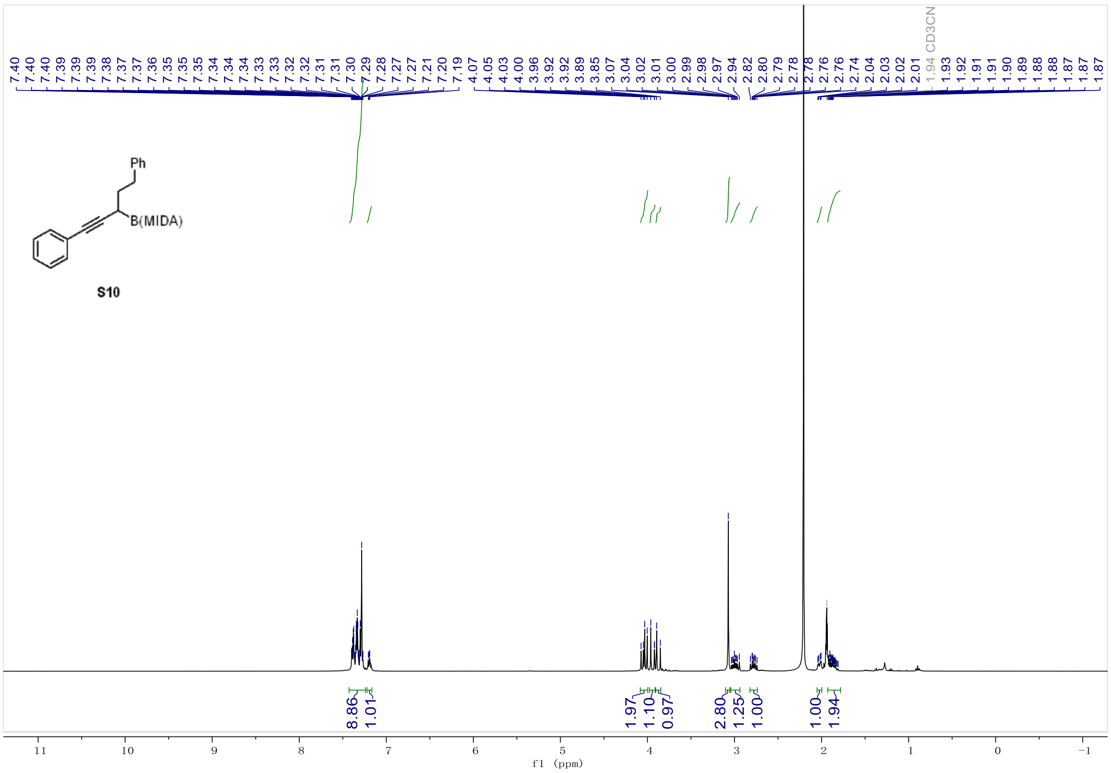

S10: ^{13}C NMR (101 MHz, Acetonitrile-*d₃*)

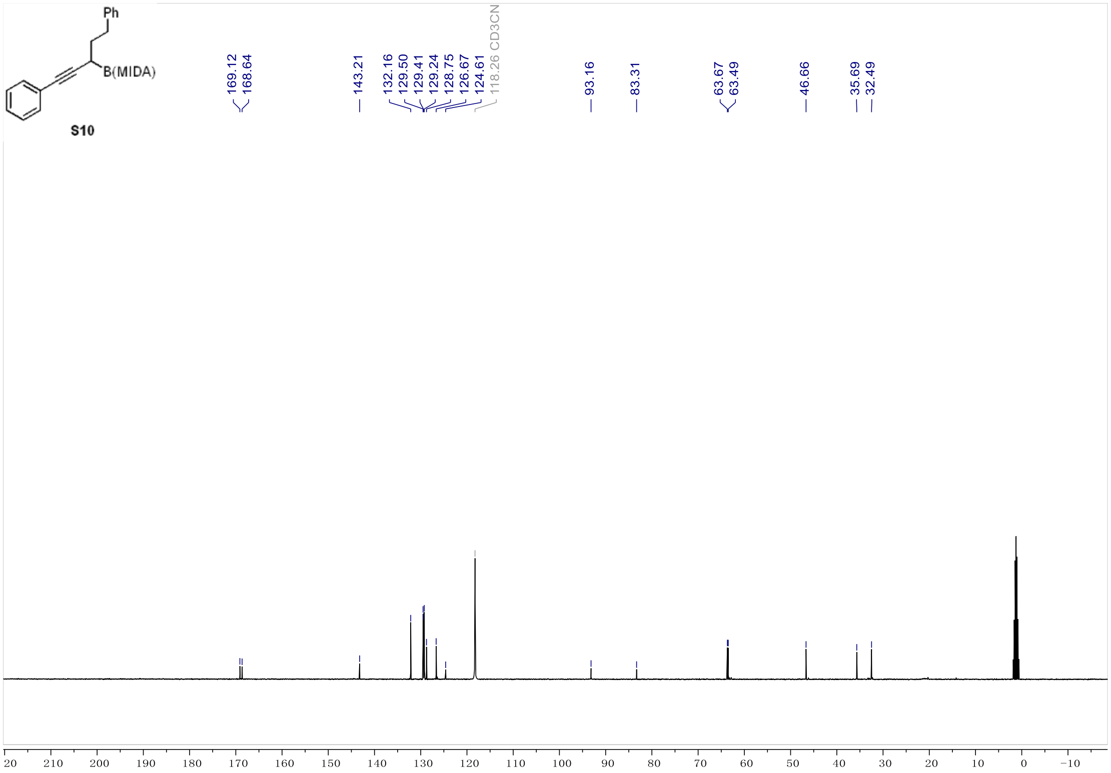

S11: ^{1}H NMR (400 MHz, DMSO-d_6)

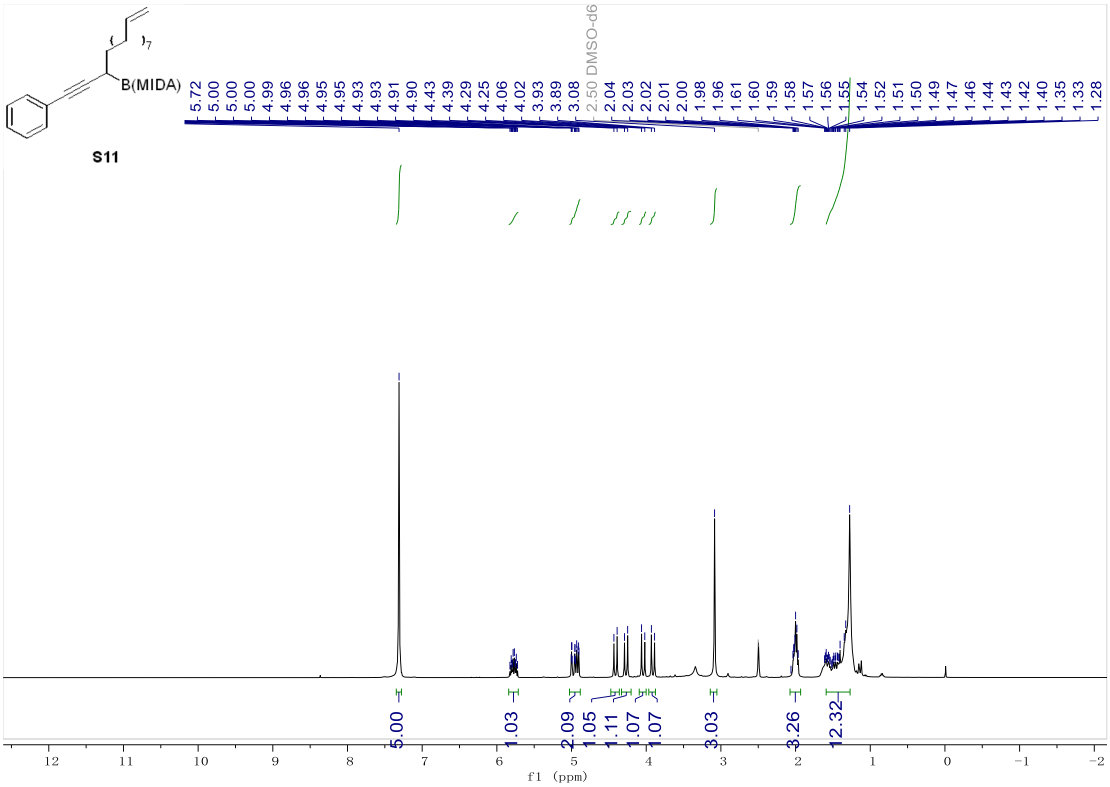

S11: ^{13}C NMR (101 MHz, DMSO-d_6)

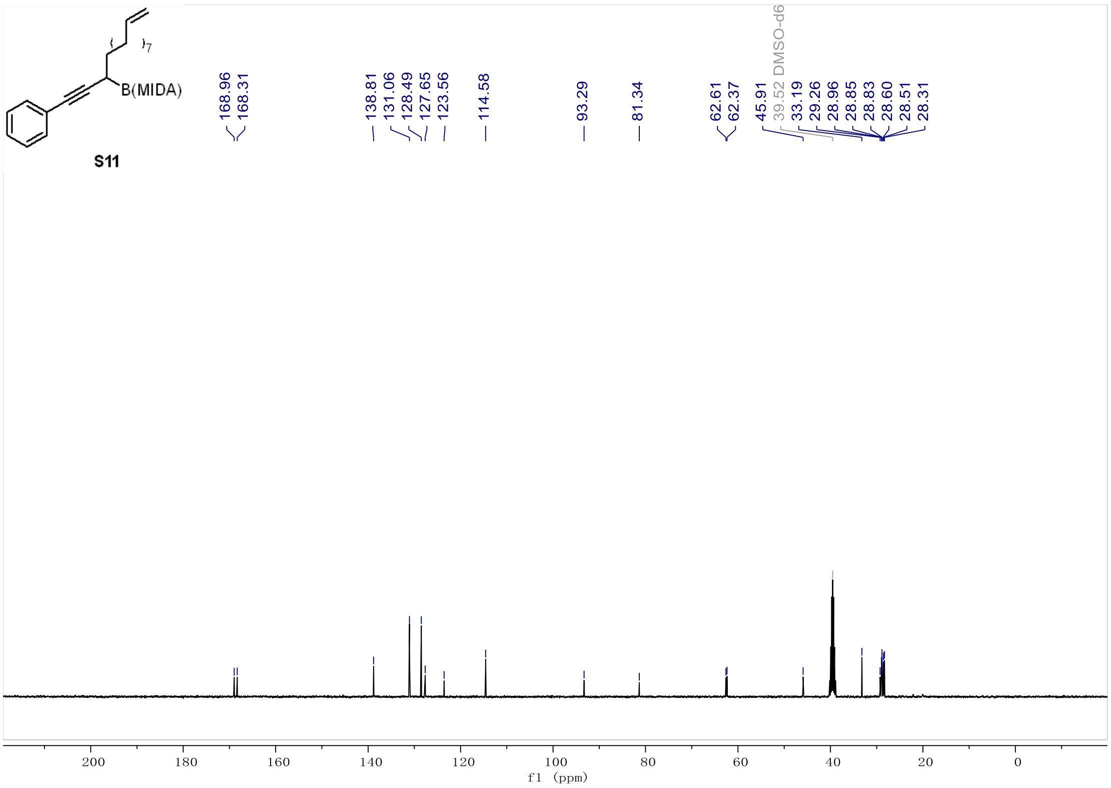

S12: ^{1}H NMR (400 MHz, DMSO-*d*$_6$)

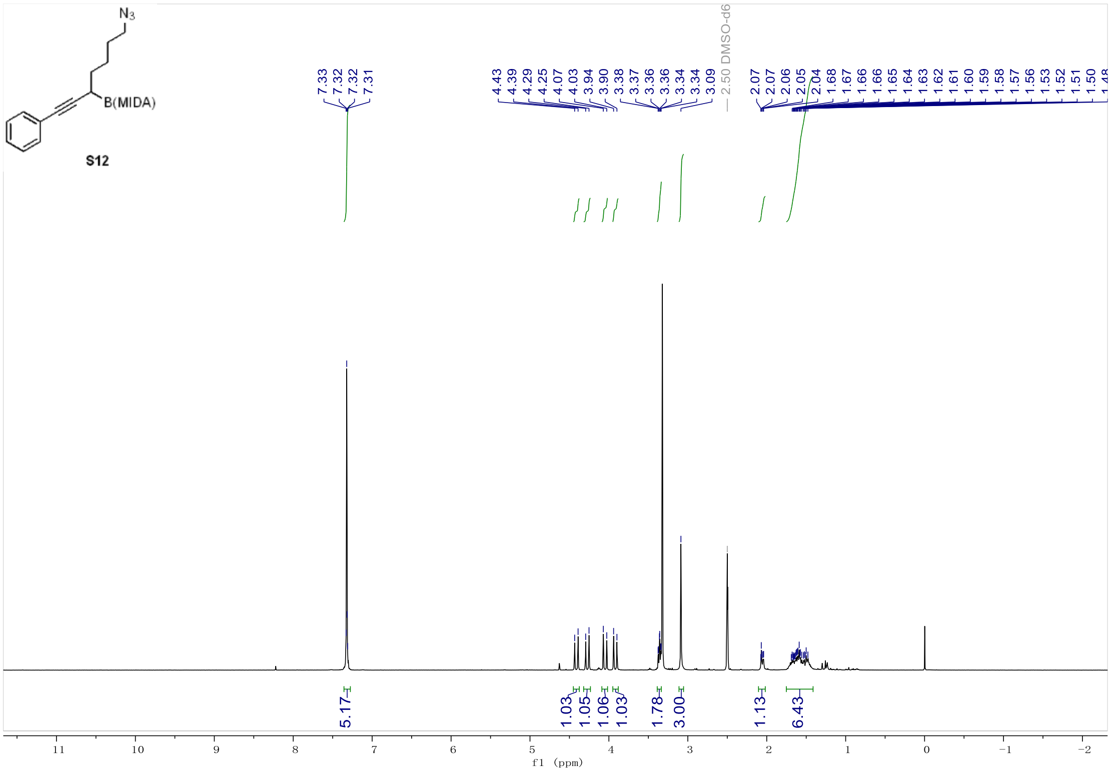

S12: ^{13}C NMR (126 MHz, Chloroform-*d*)

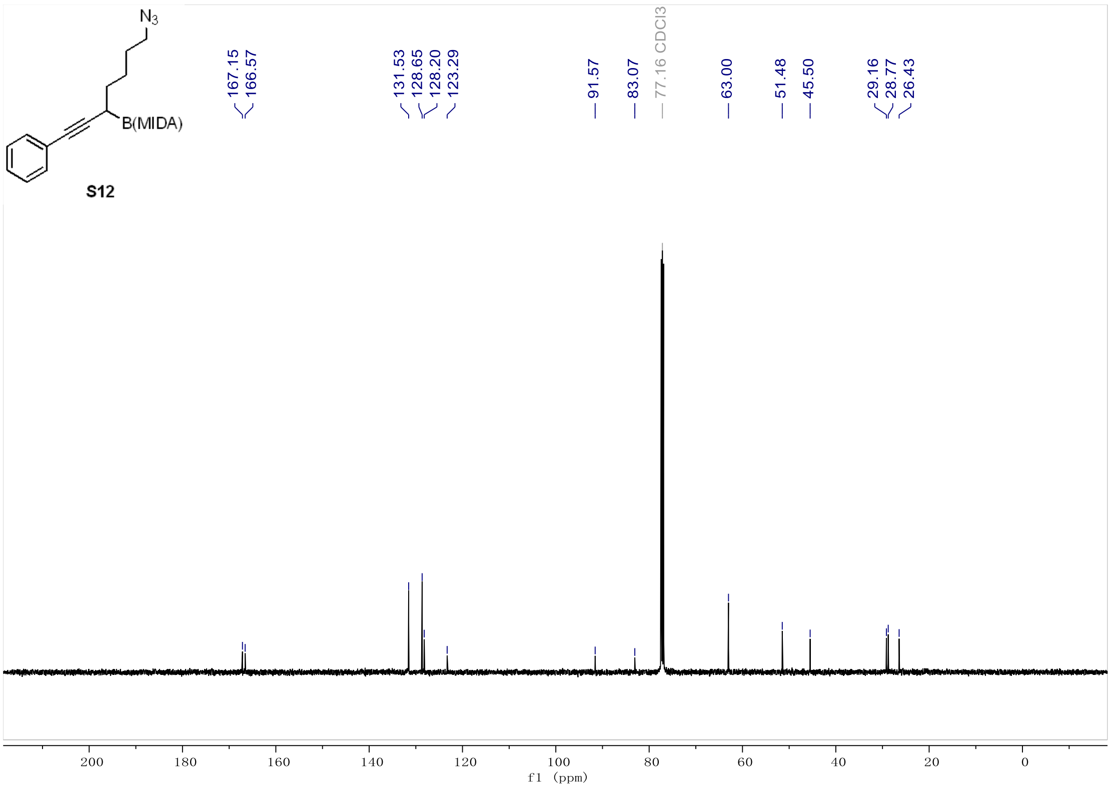

S13: ^{1}H NMR (400 MHz, Chloroform-*d*)

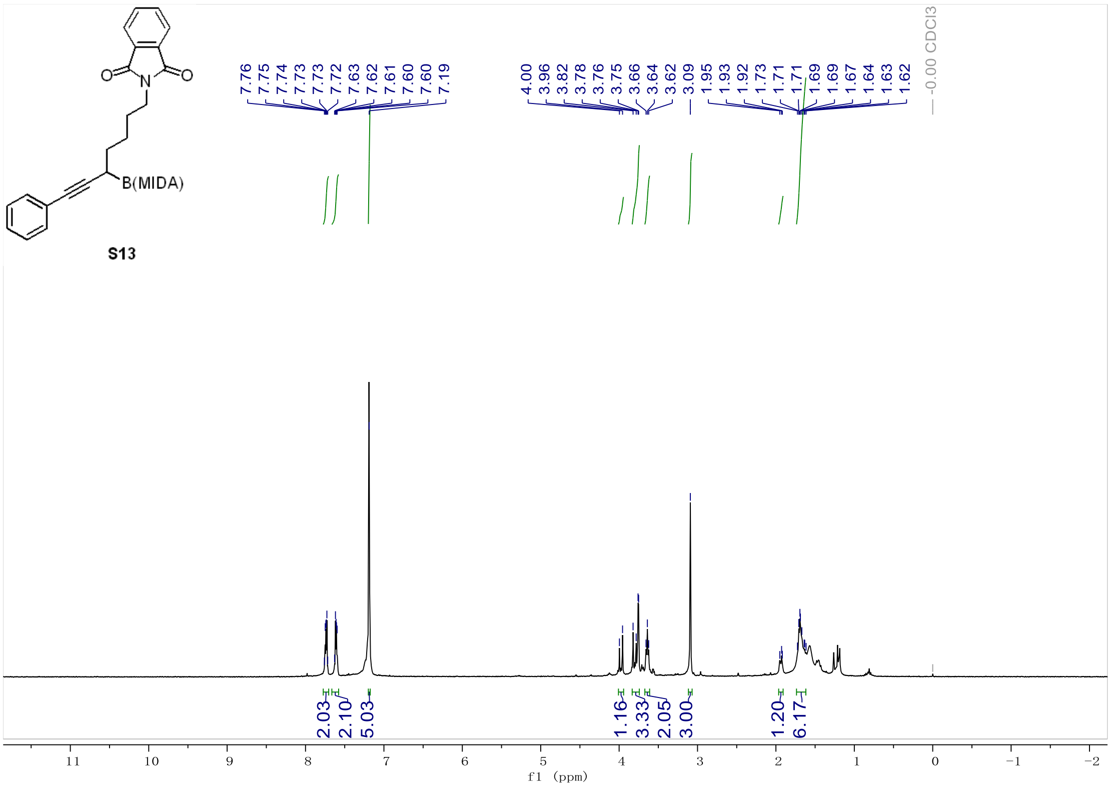

S13: ^{13}C NMR (126 MHz, Chloroform-*d*)

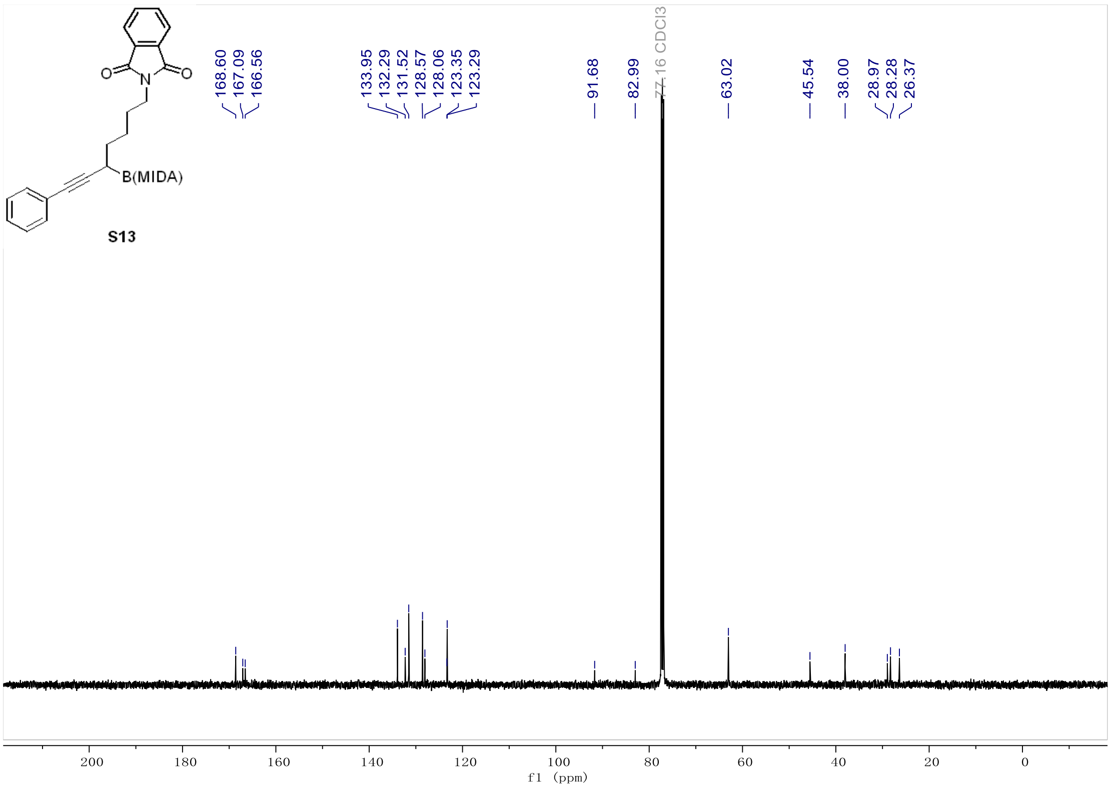

S14: ^{1}H NMR (500 MHz, DMSO-*d$_6$*)

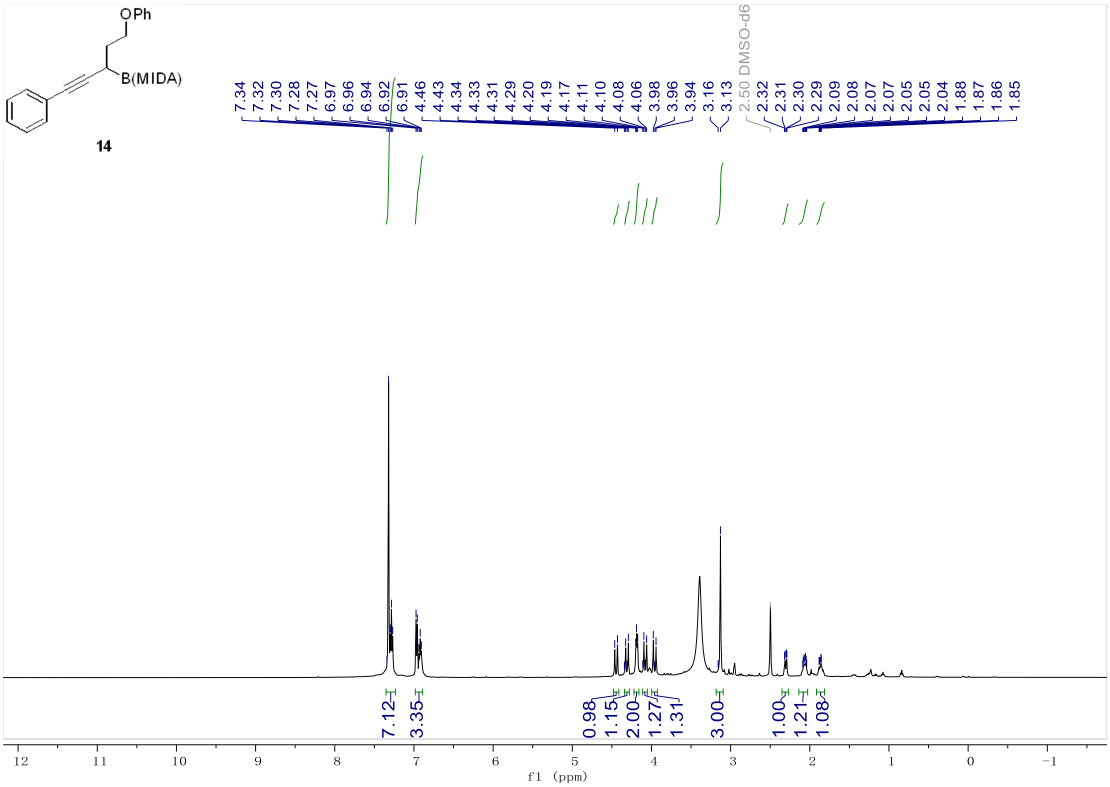

S14: ^{13}C NMR (126 MHz, DMSO-*d$_6$*)

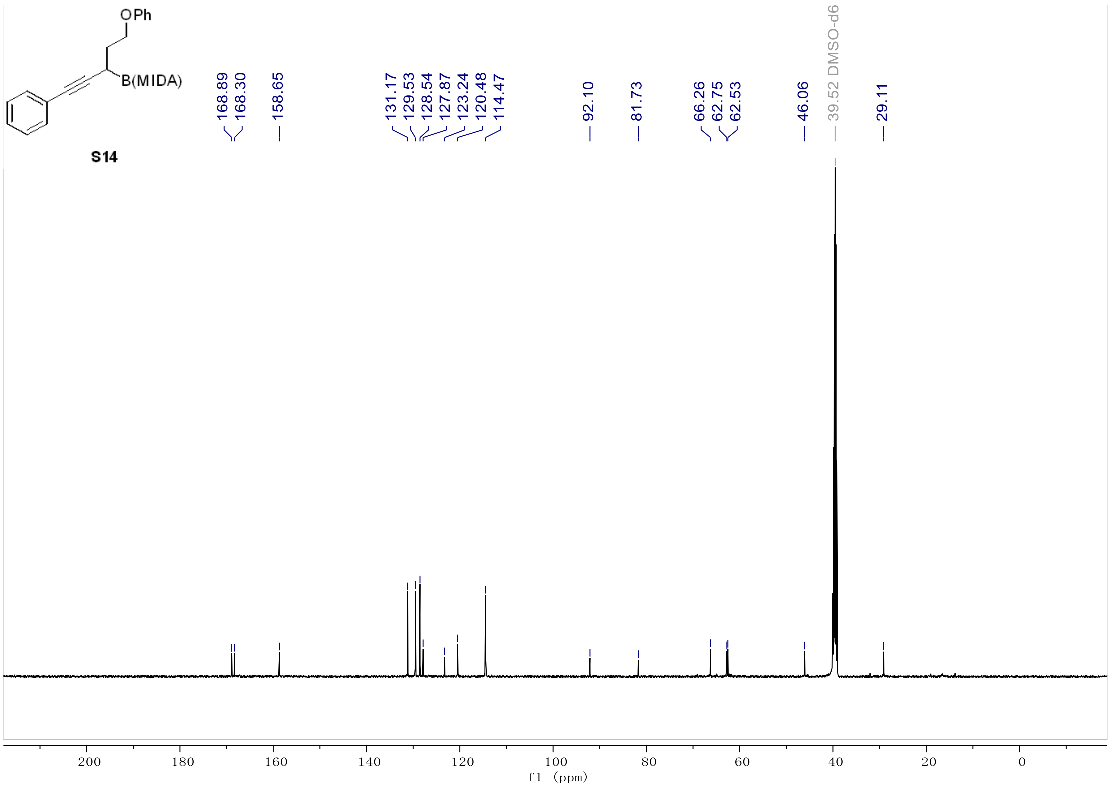

S15: ^{1}H NMR (500 MHz, DMSO-d_6)

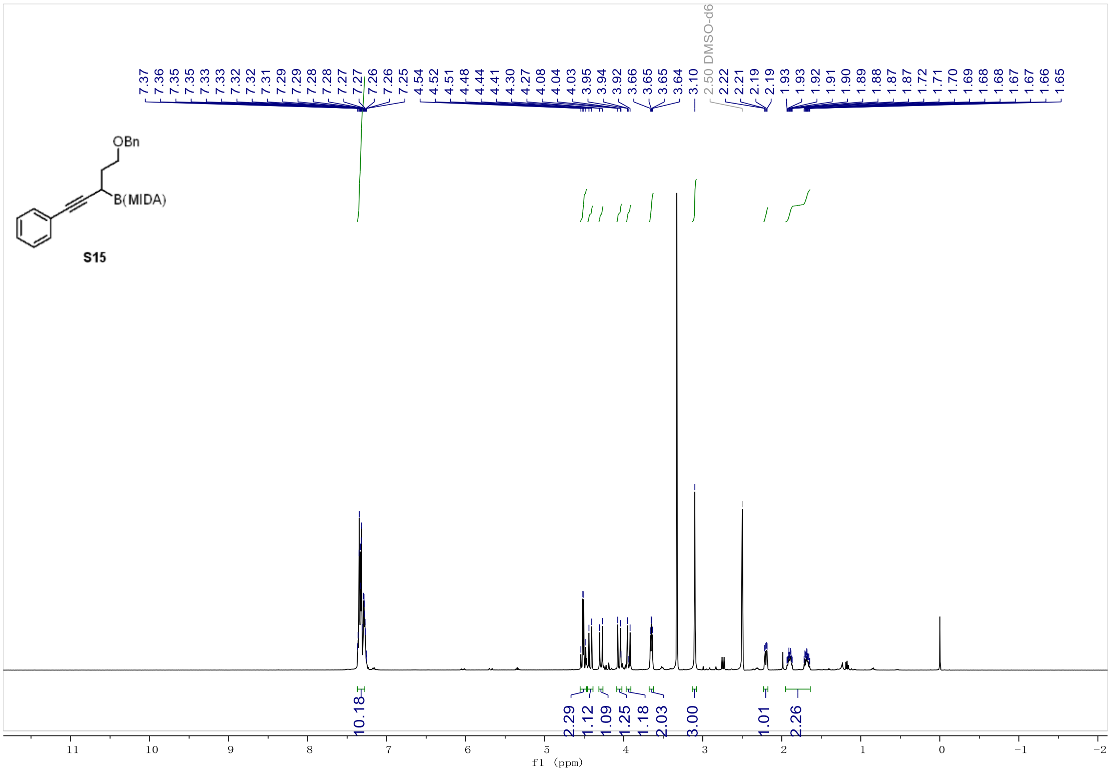

S15: ^{13}C NMR (101 MHz, DMSO-d_6)

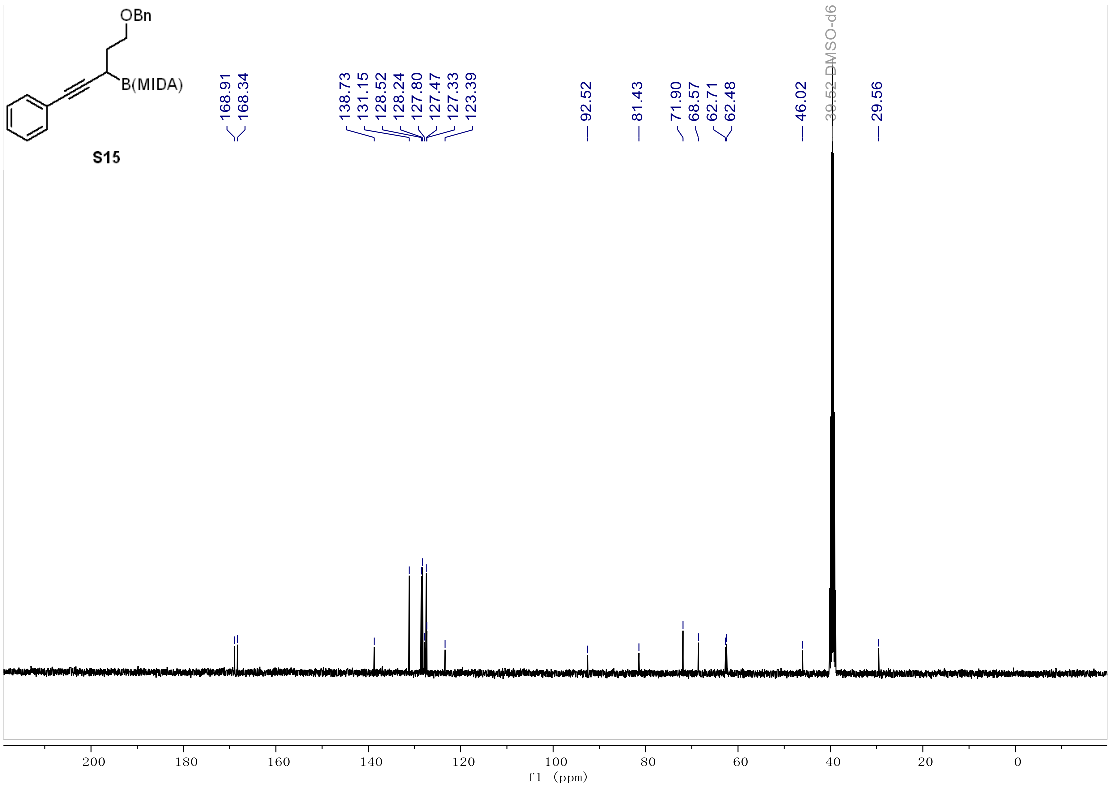

S16: ^{1}H NMR (400 MHz, Acetonitrile-d_3)

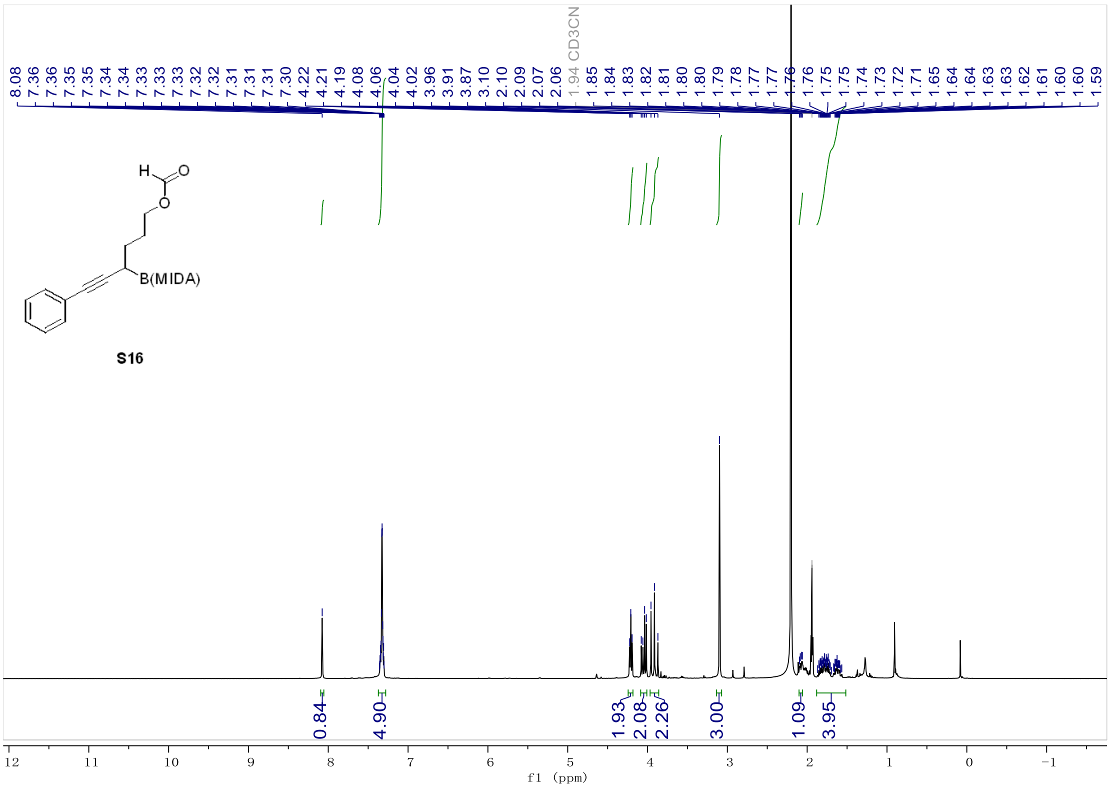

S16: ^{13}C NMR (126 MHz, Acetonitrile-d_3)

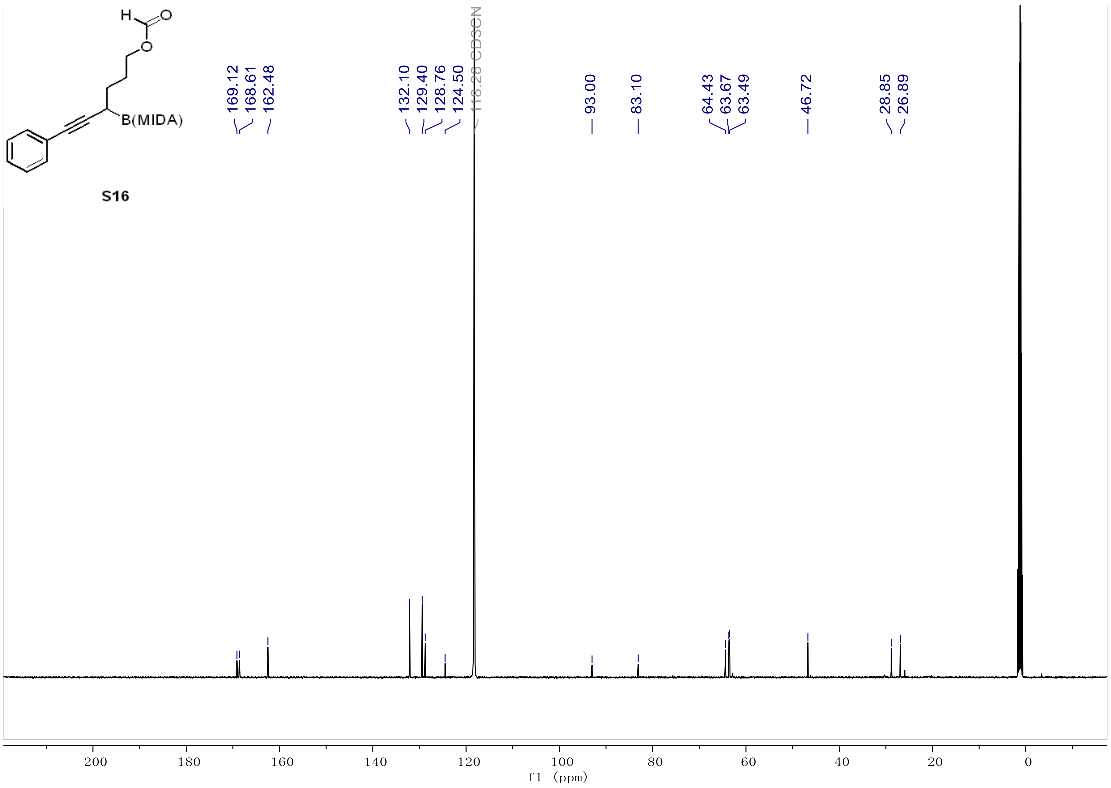

S17: ^{1}H NMR (400 MHz, DMSO-*d6*)

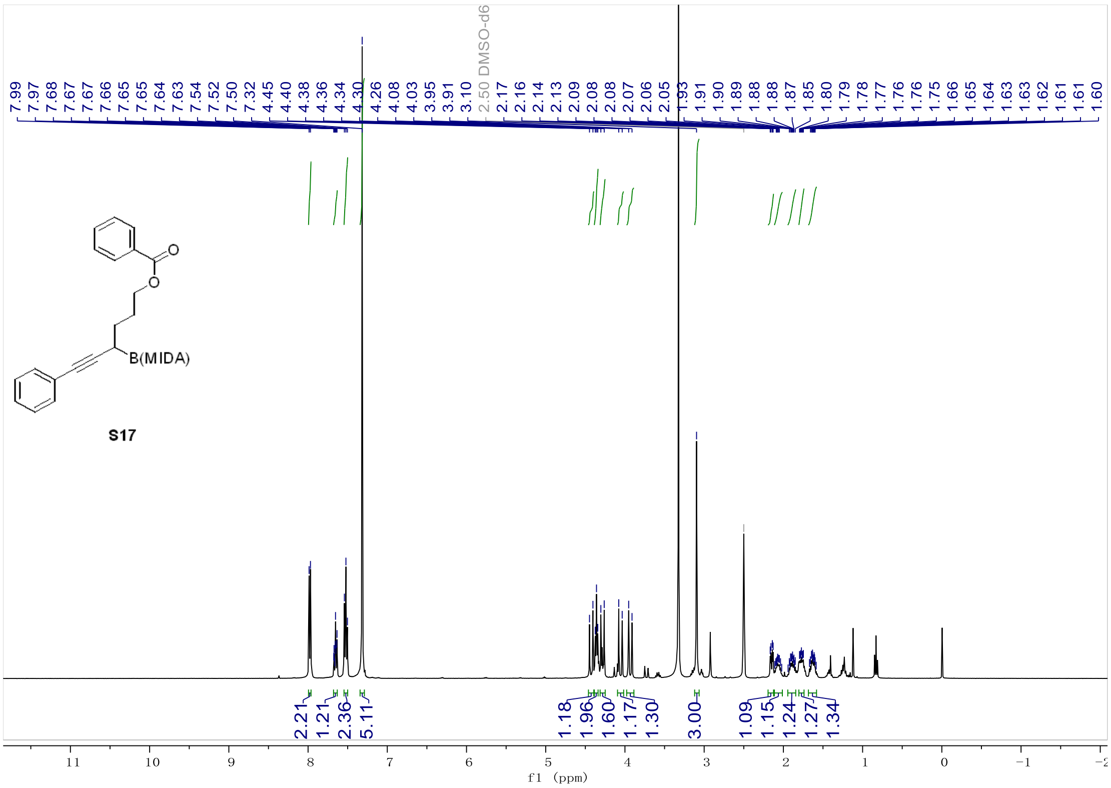

S17: ^{13}C NMR (101 MHz, DMSO-*d6*)

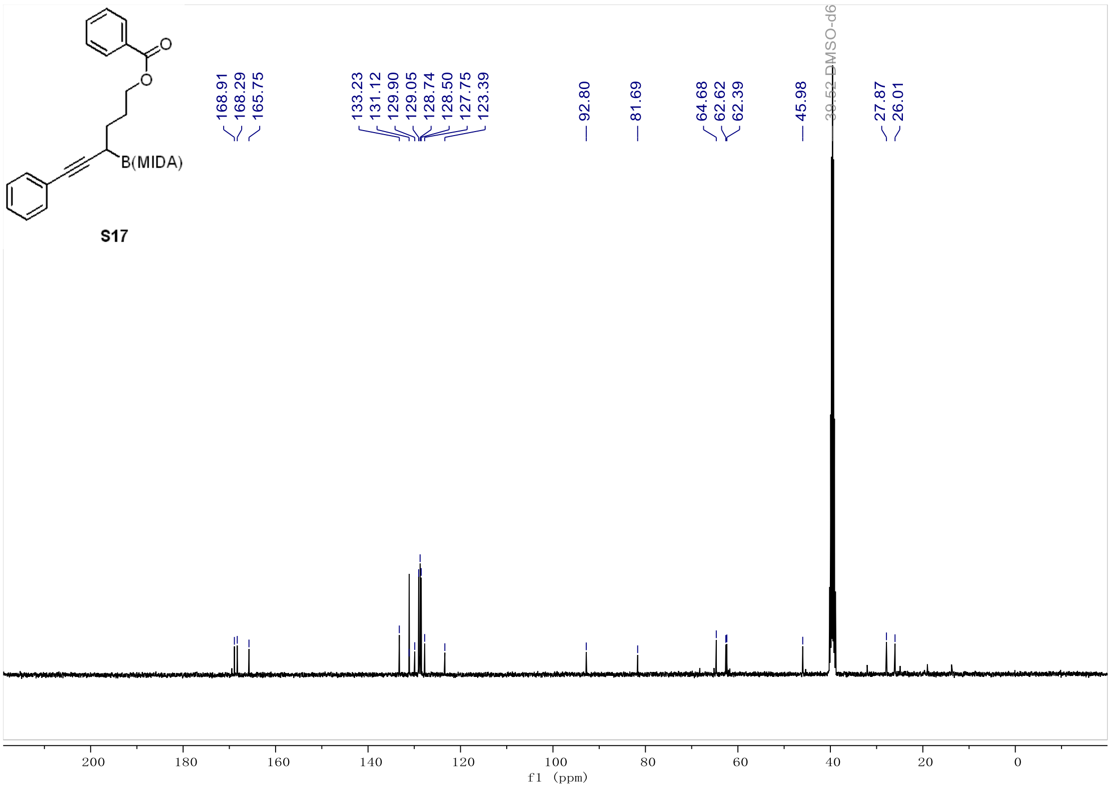

S18: ¹H NMR (400 MHz, DMSO-*d₆*)

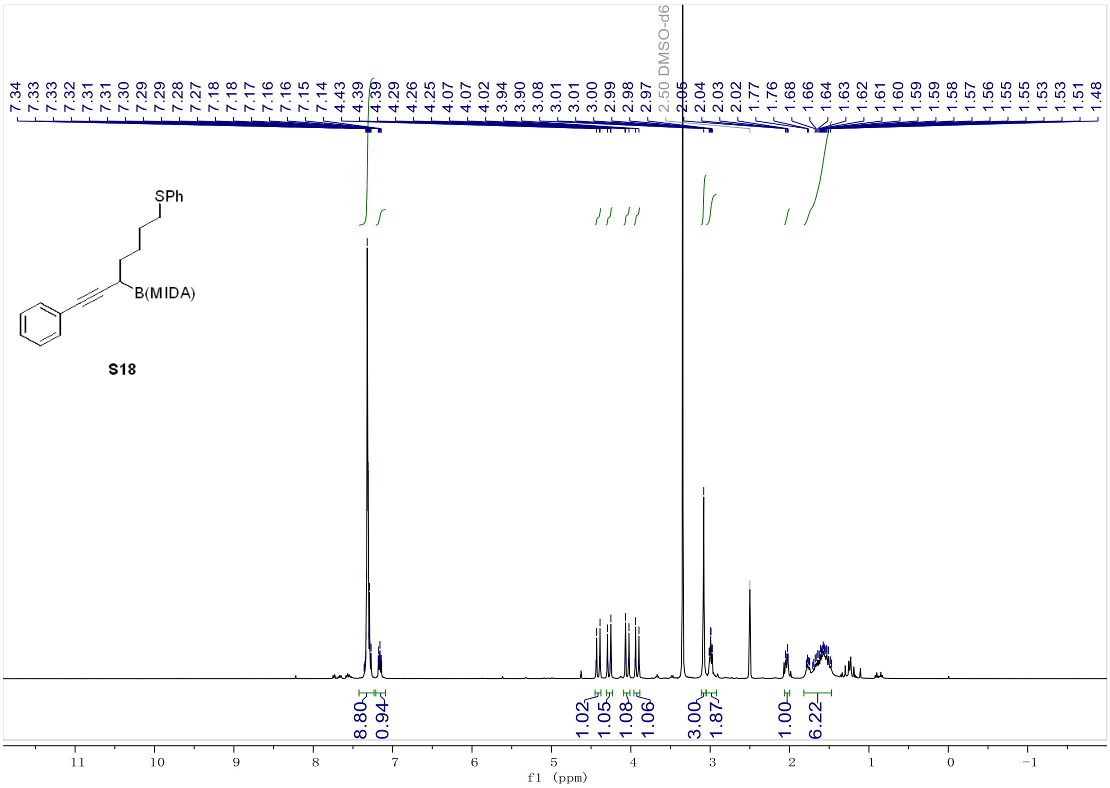

S18: ¹³C NMR (126 MHz, DMSO-*d₆*)

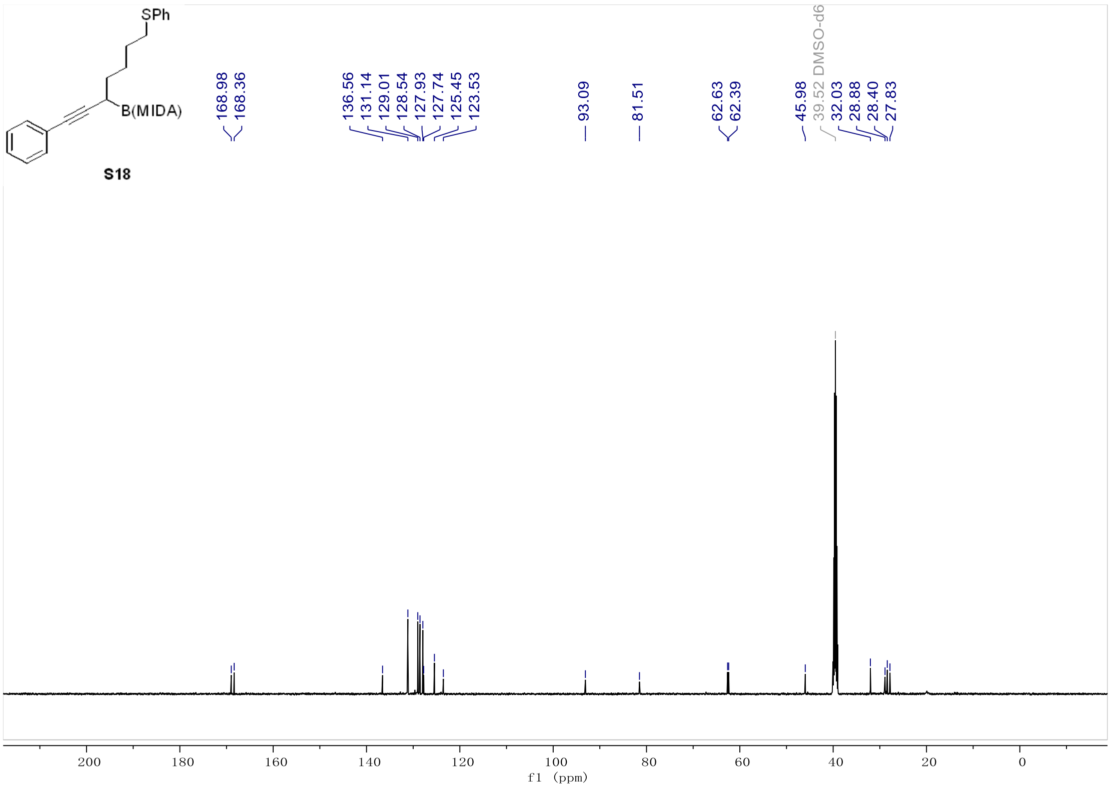

S19: ^{1}H NMR (400 MHz, DMSO-d_6)

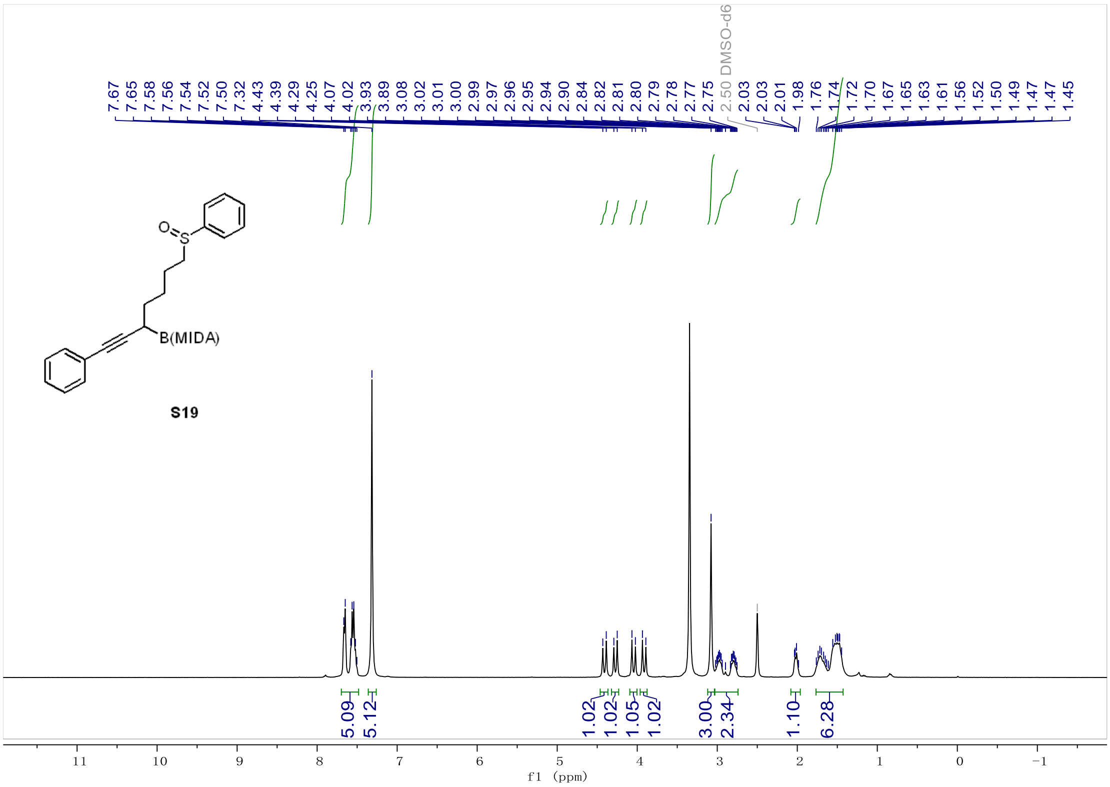

S19: ^{13}C NMR (101 MHz, DMSO-d_6)

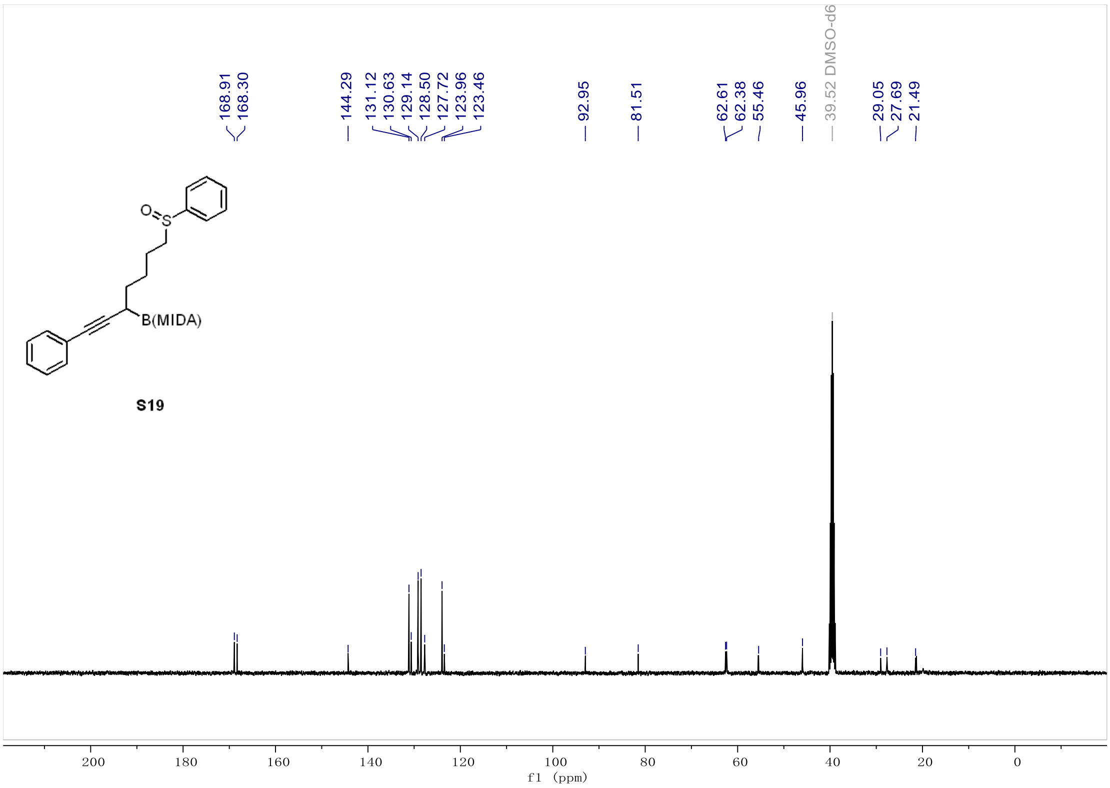

S20: ^{1}H NMR (400 MHz, DMSO-*d*$_6$)

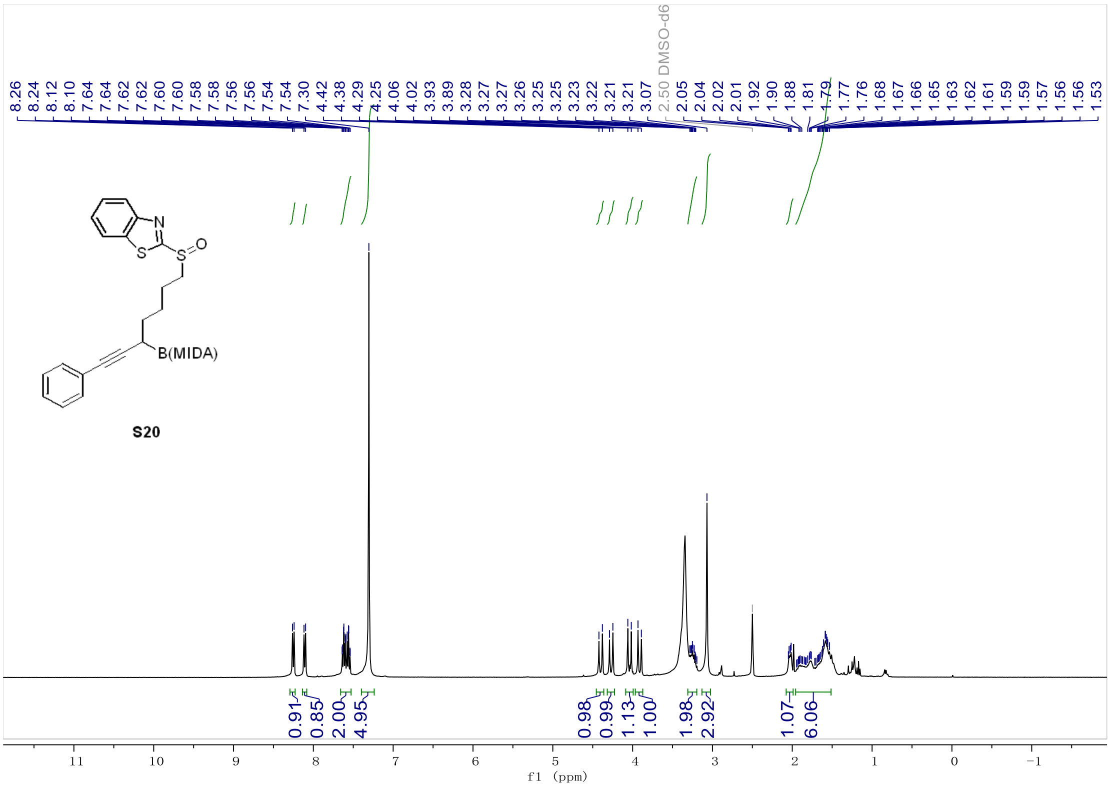

S20: ^{13}C NMR (126 MHz, Chloroform-*d*)

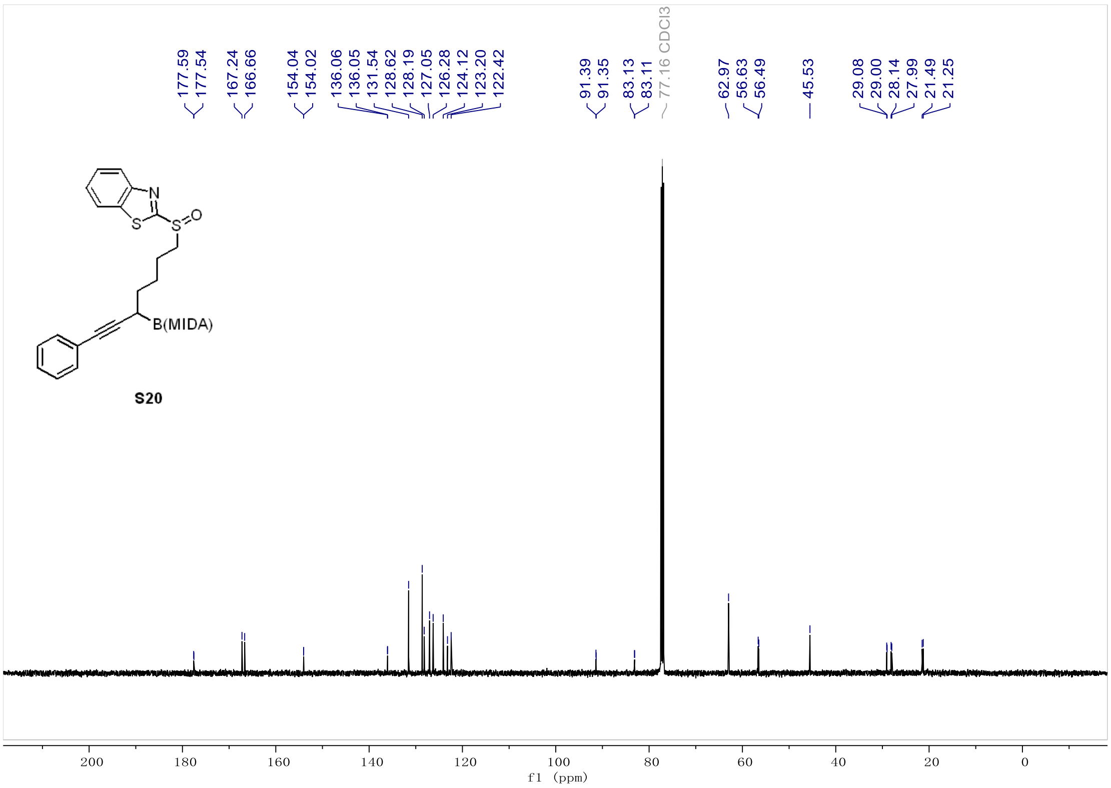

S21: ^{1}H NMR (400 MHz, DMSO-d_6)

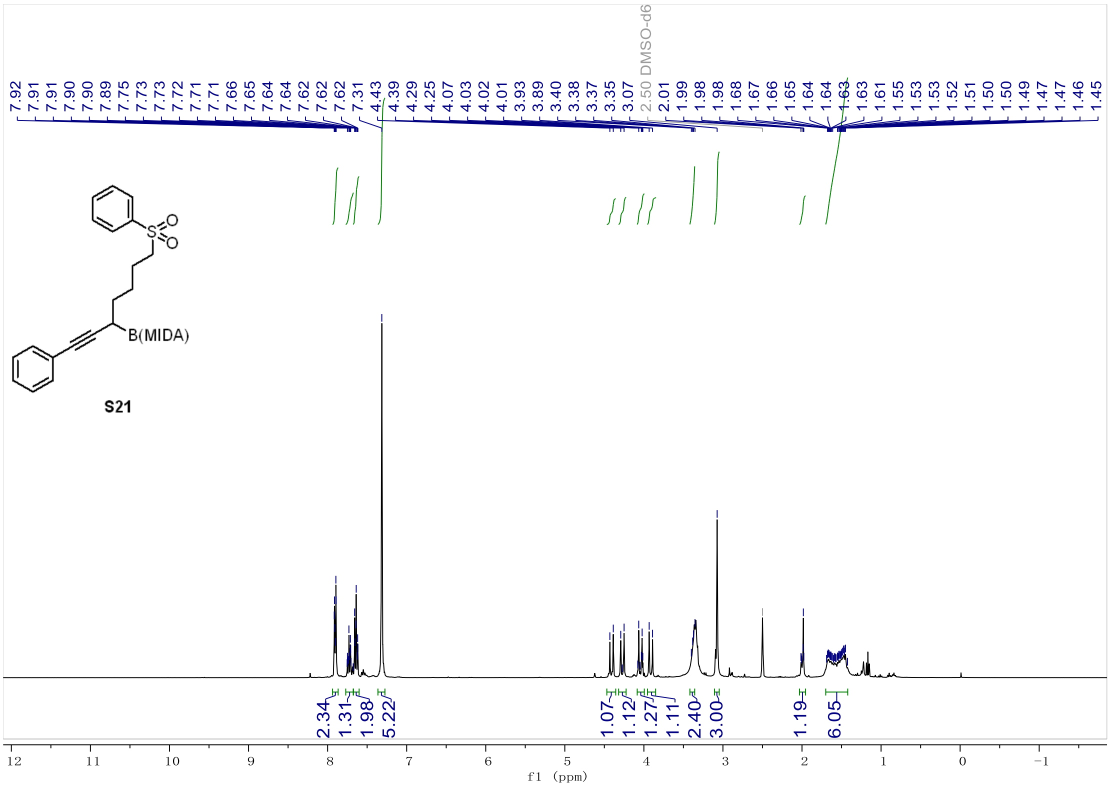

S21: ^{13}C NMR (126 MHz, DMSO-d_6)

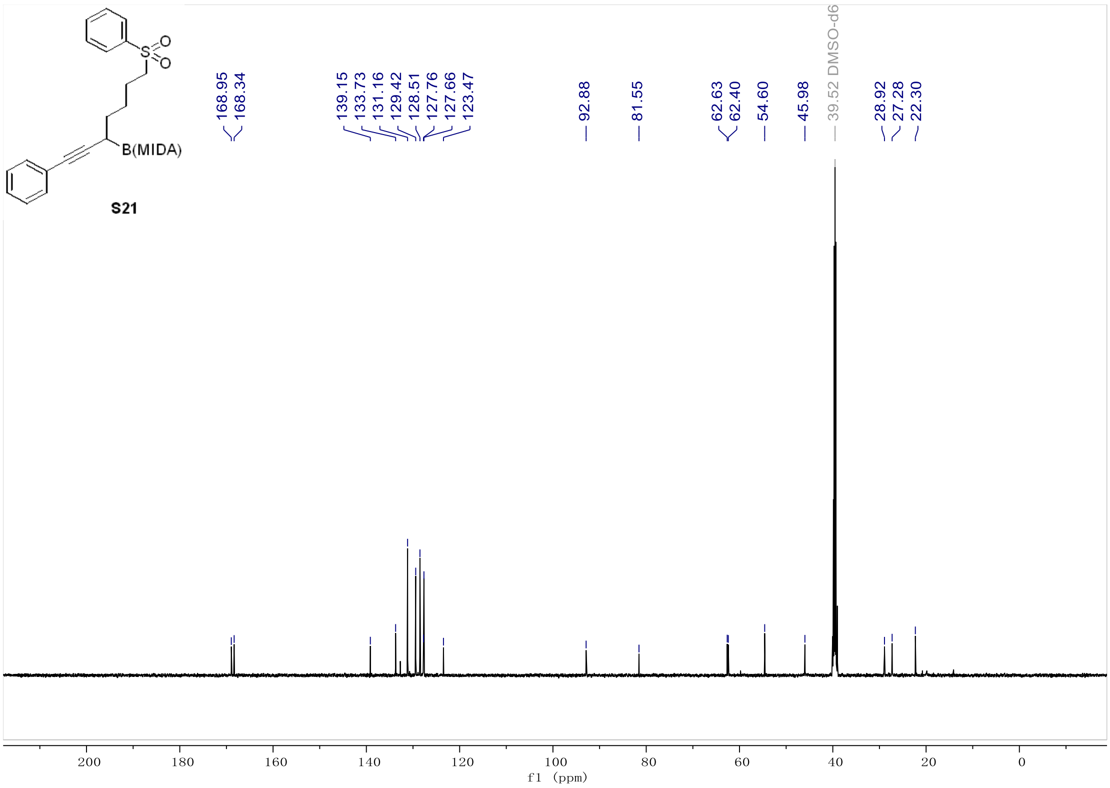

S22: ^{1}H NMR (400 MHz, Chloroform-*d*)

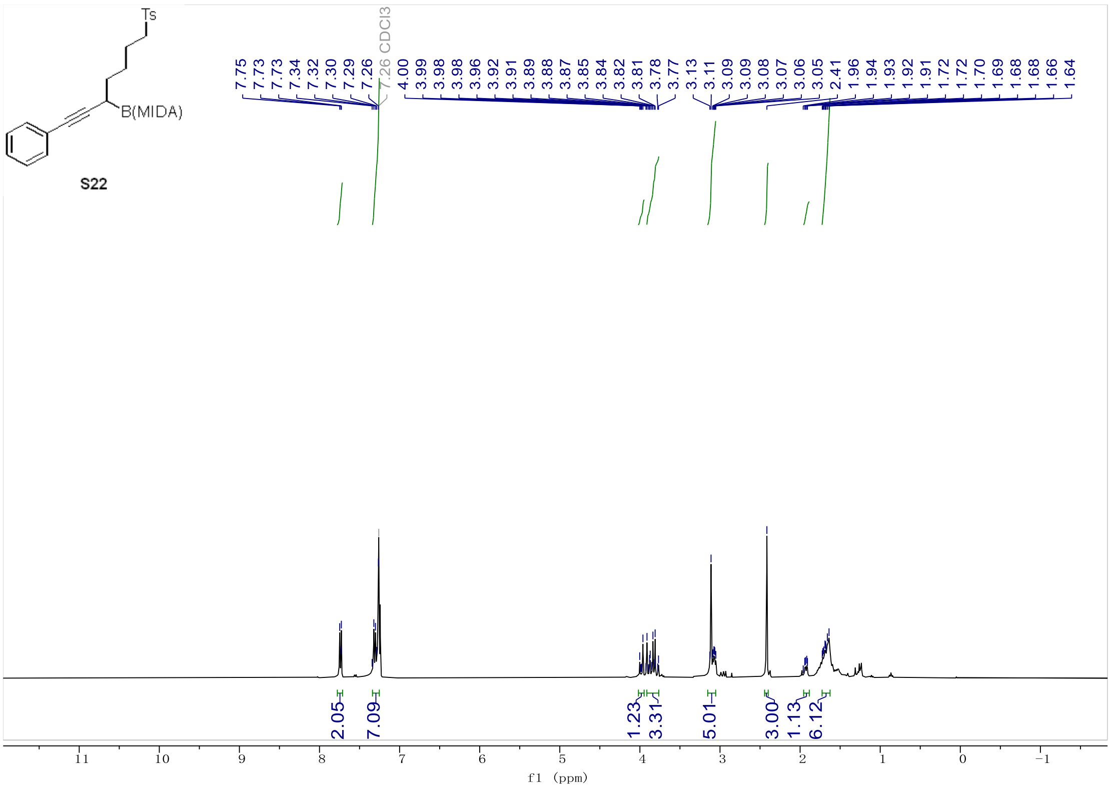

S22: ^{13}C NMR (101 MHz, Chloroform-*d*)

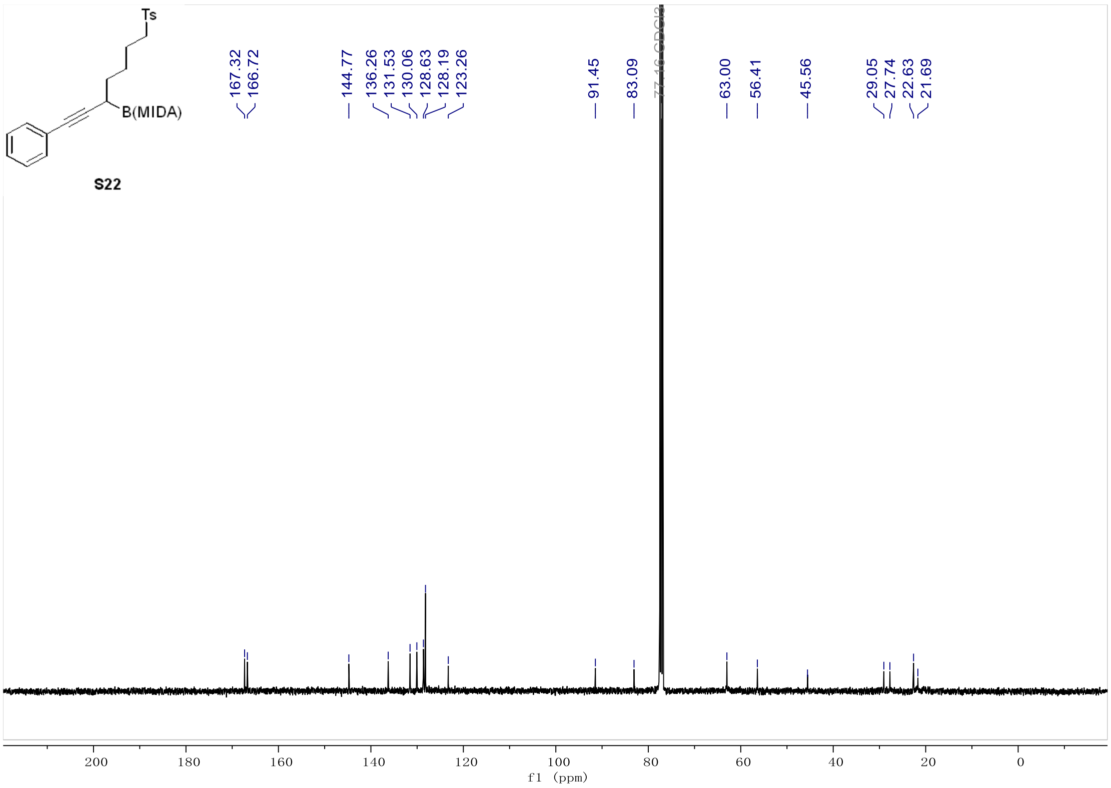

S23: ¹H NMR (400 MHz, DMSO-*d6*)

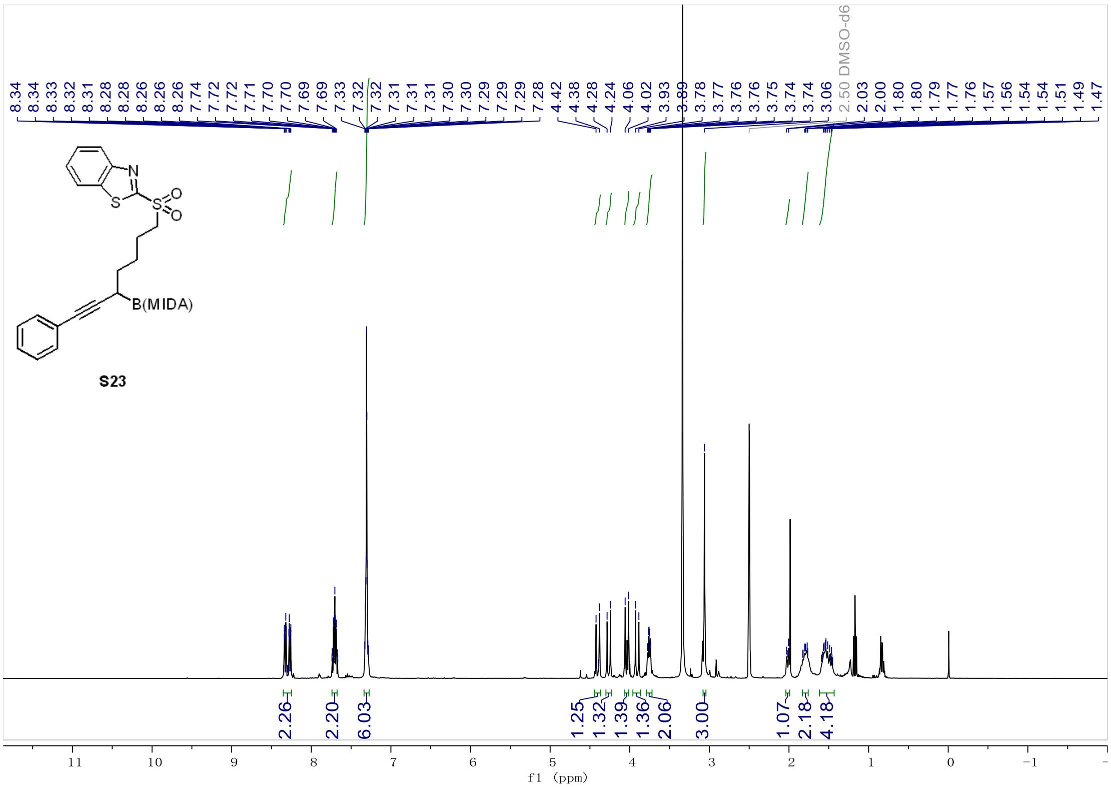

S23: ¹³C NMR (126 MHz, DMSO-*d6*)

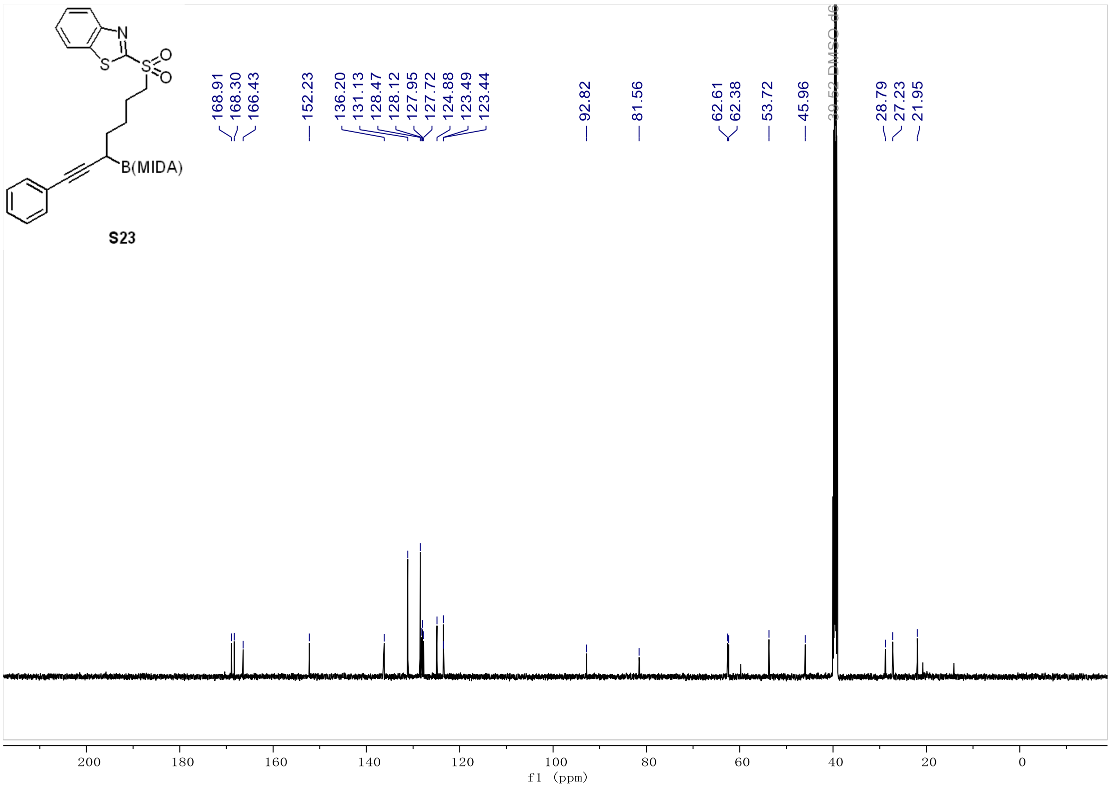

S24: ^{1}H NMR (400 MHz, DMSO-d_6)

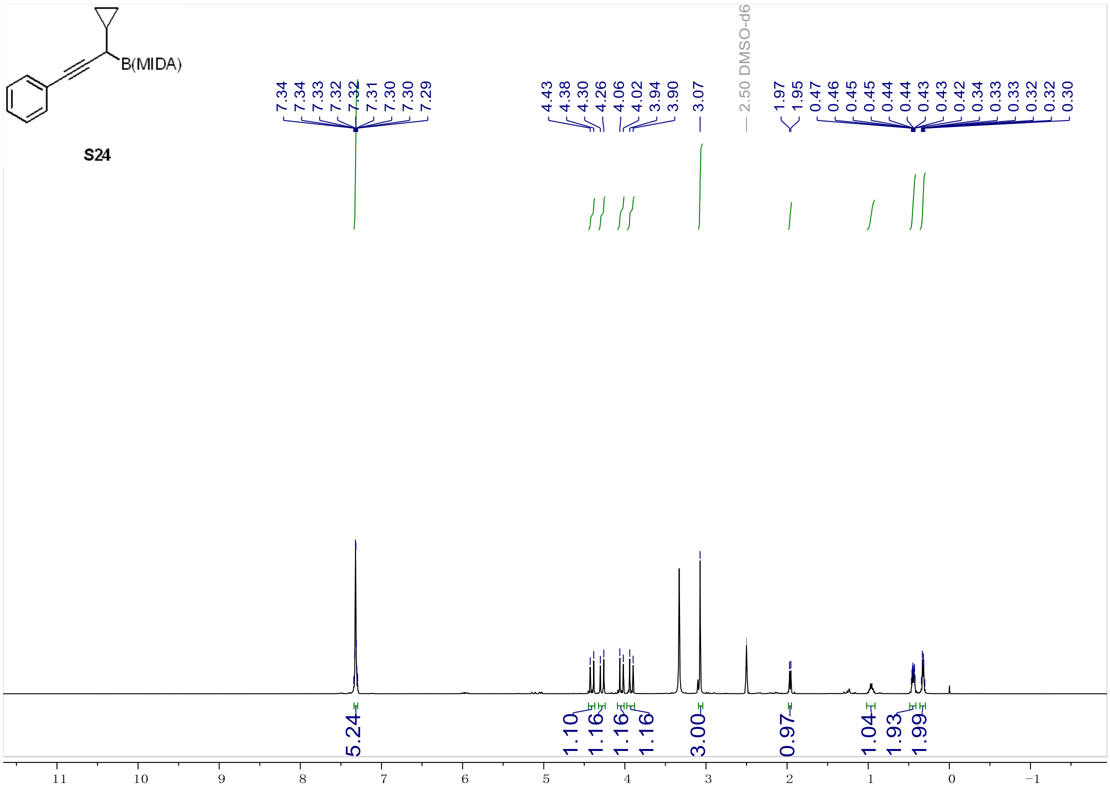

S24: ^{13}C NMR (101 MHz, DMSO-d_6)

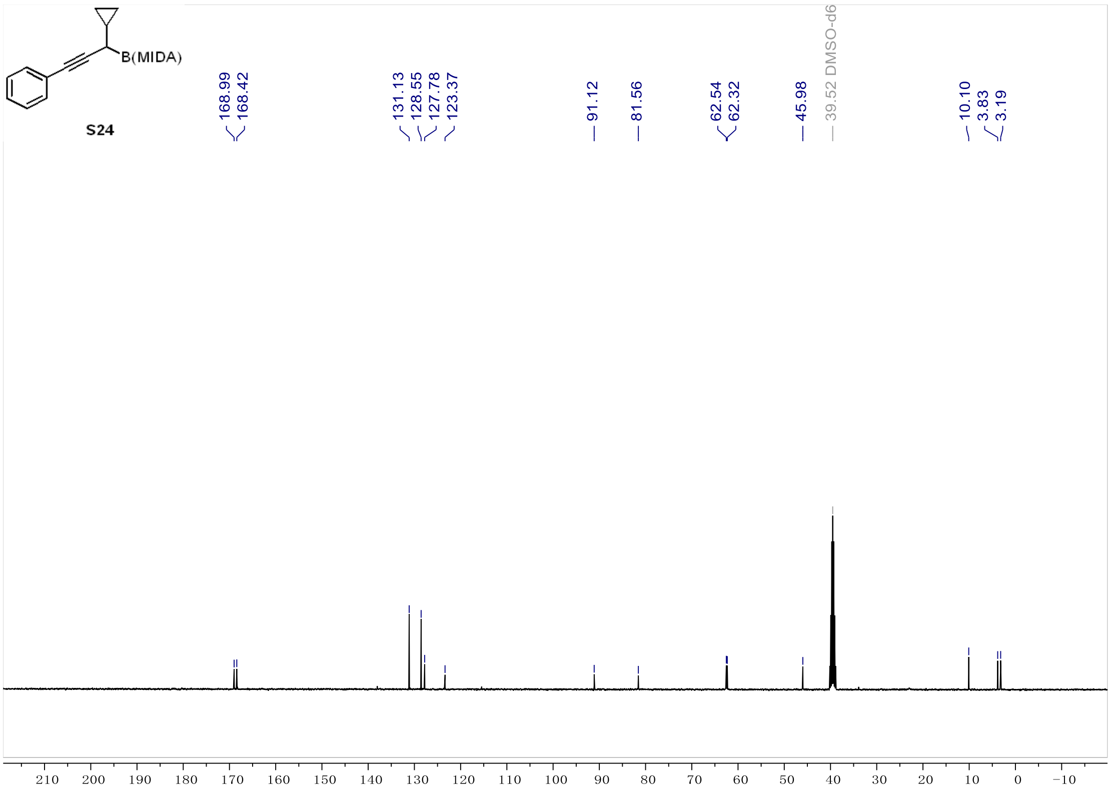

S25: ^{1}H NMR (400 MHz, Acetonitrile-d_3)

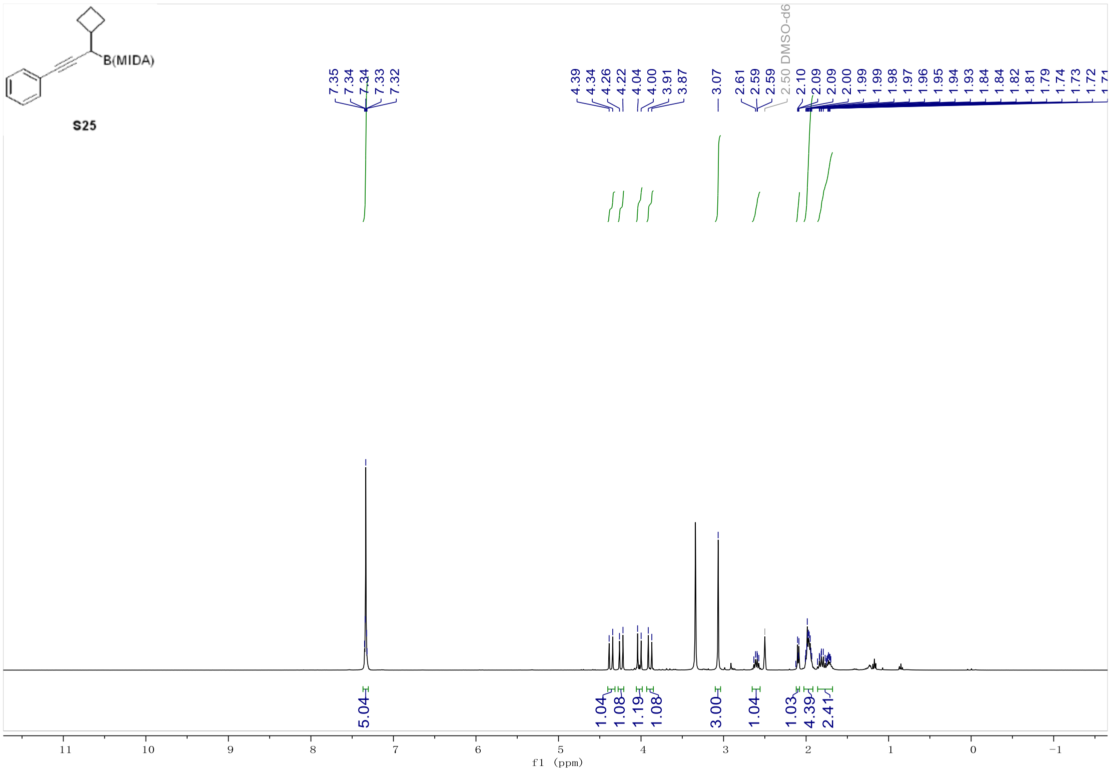

S25: ^{13}C NMR (126 MHz, DMSO-d_6)

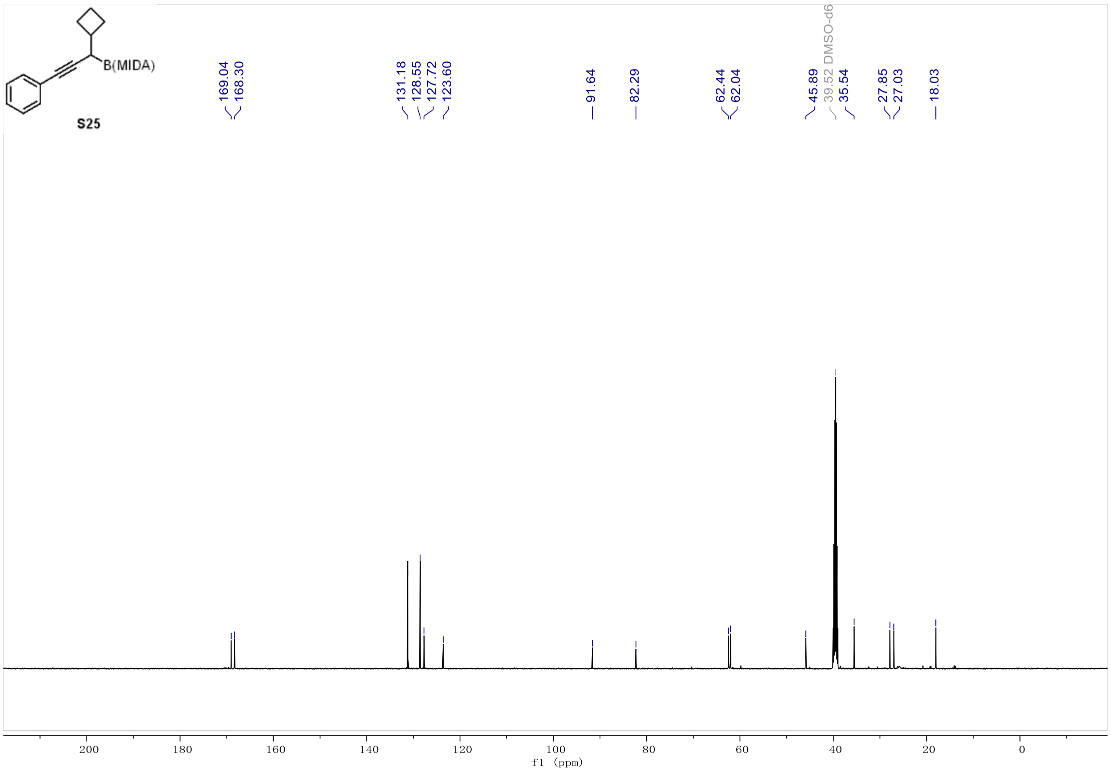

S26: ^{1}H NMR (400 MHz, DMSO-d_6)

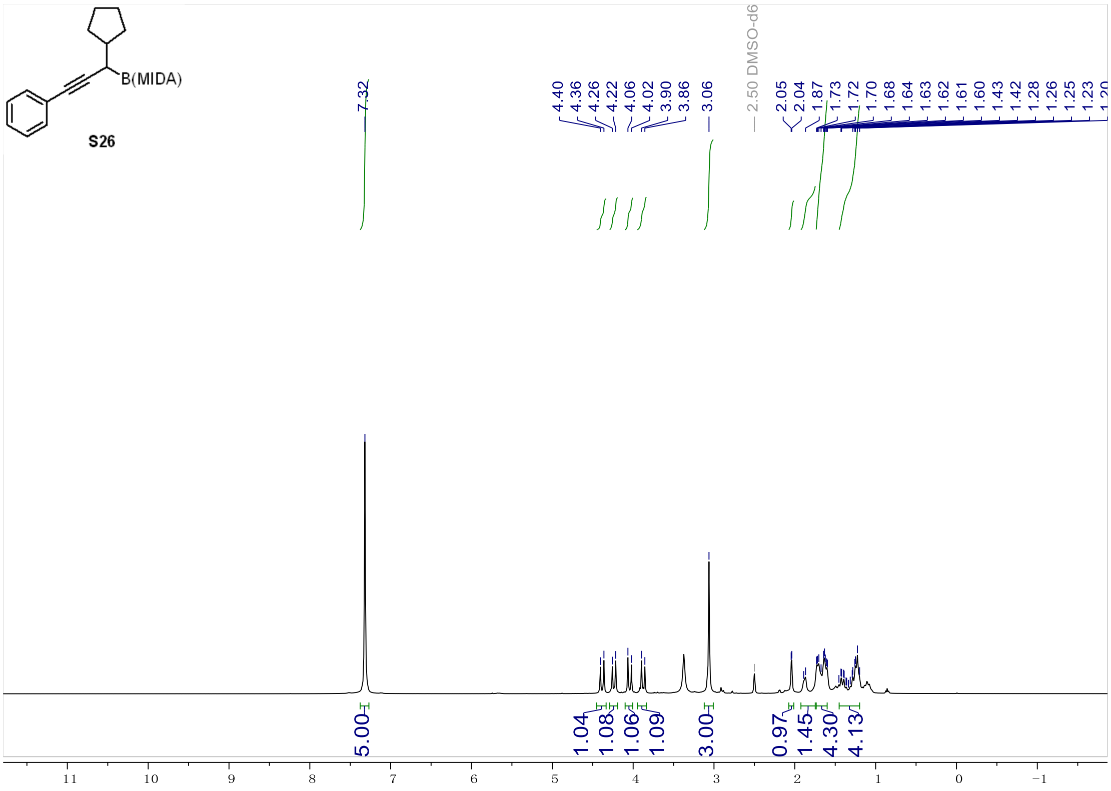

S26: ^{13}C NMR (101 MHz, DMSO-d_6)

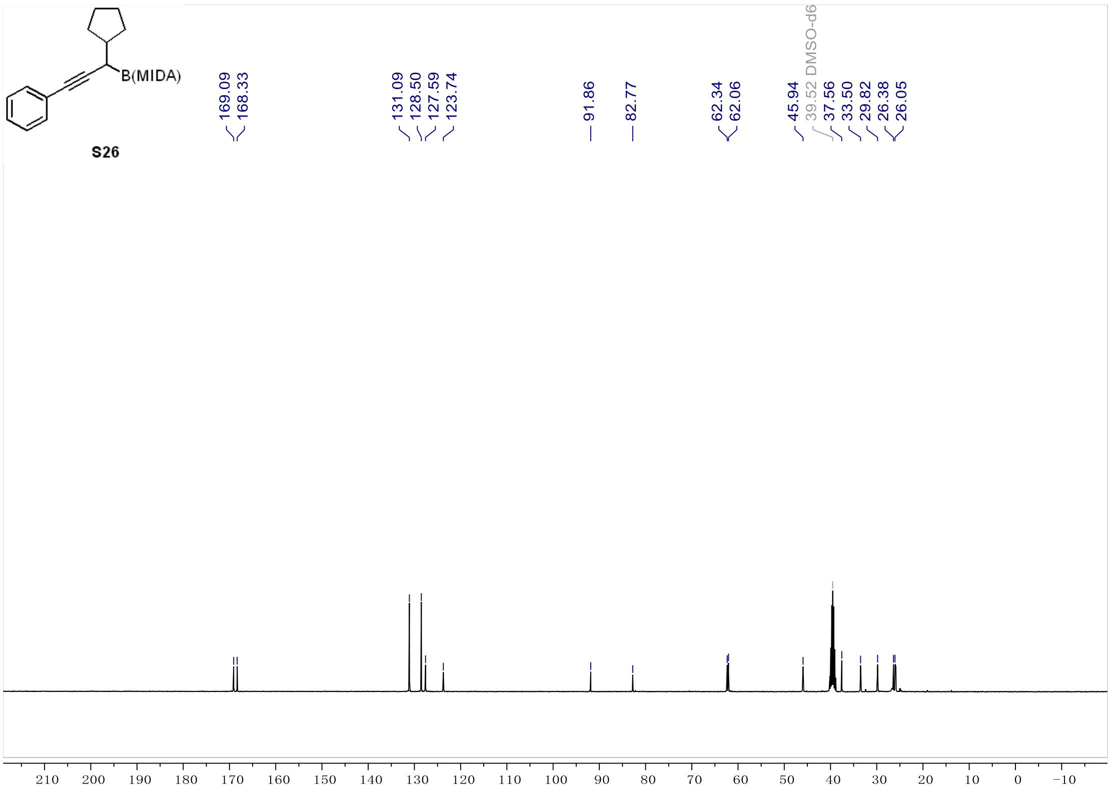

S27: ^{1}H NMR (400 MHz, DMSO-d_6)

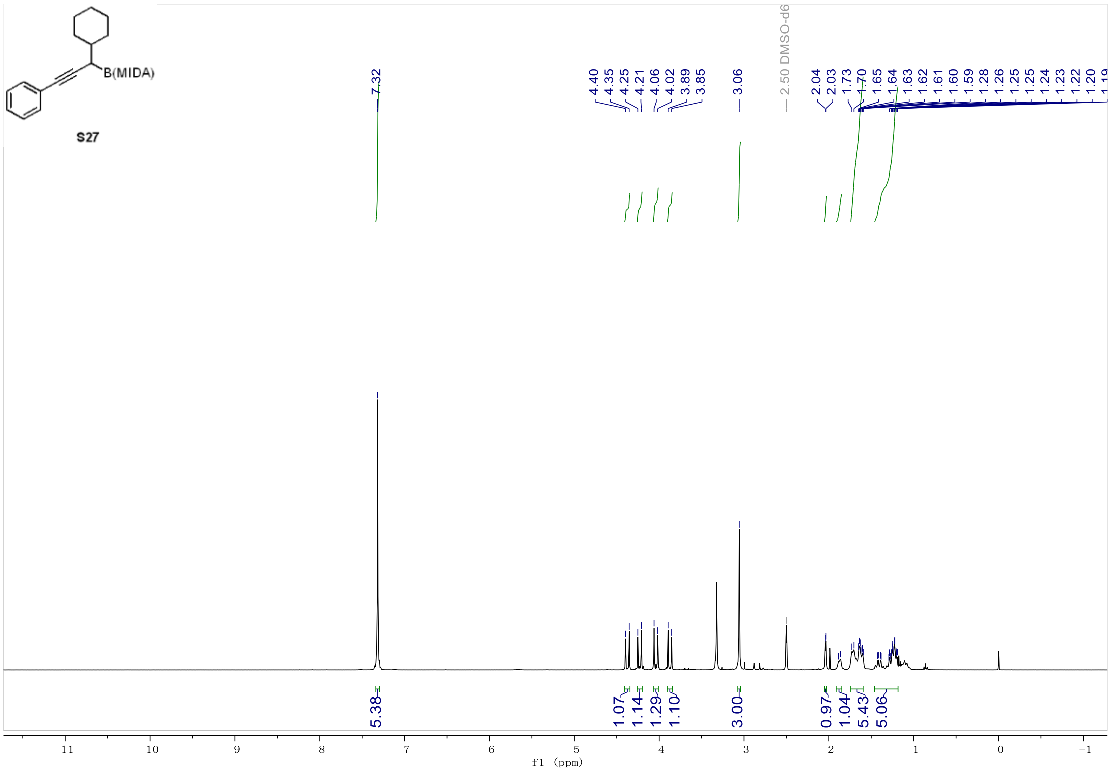

S27: ^{13}C NMR (101 MHz, DMSO-d_6)

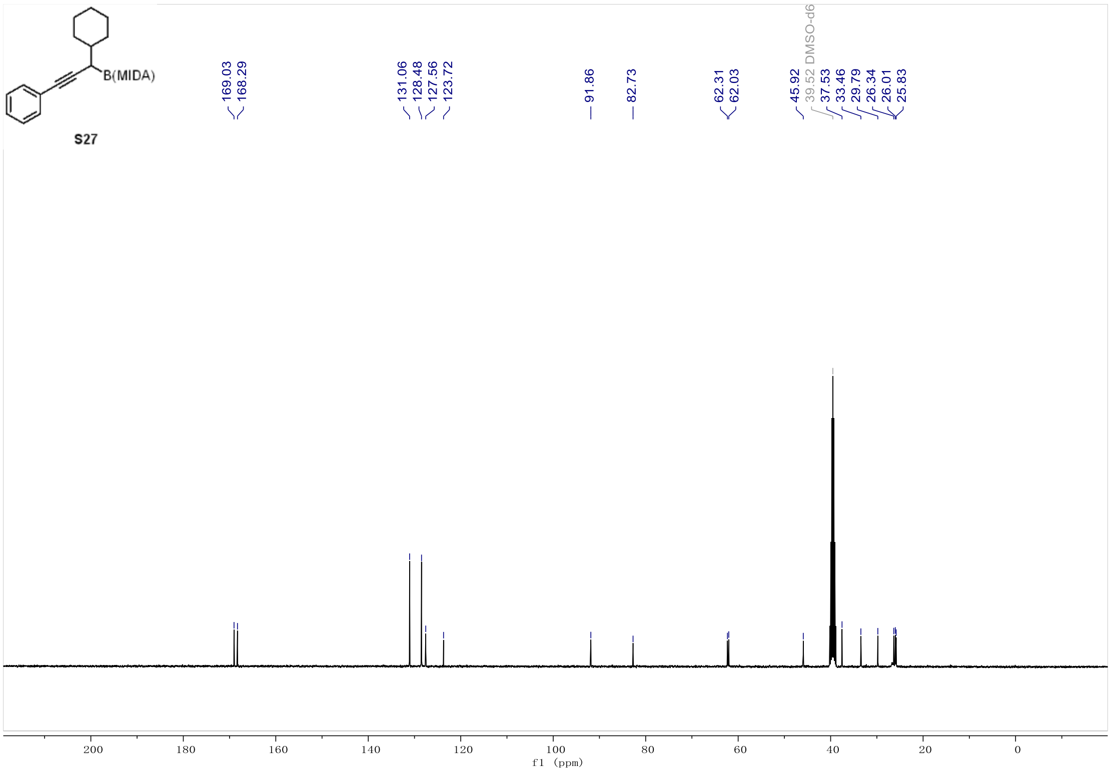

S28: ^{1}H NMR (400 MHz, DMSO-d_6)

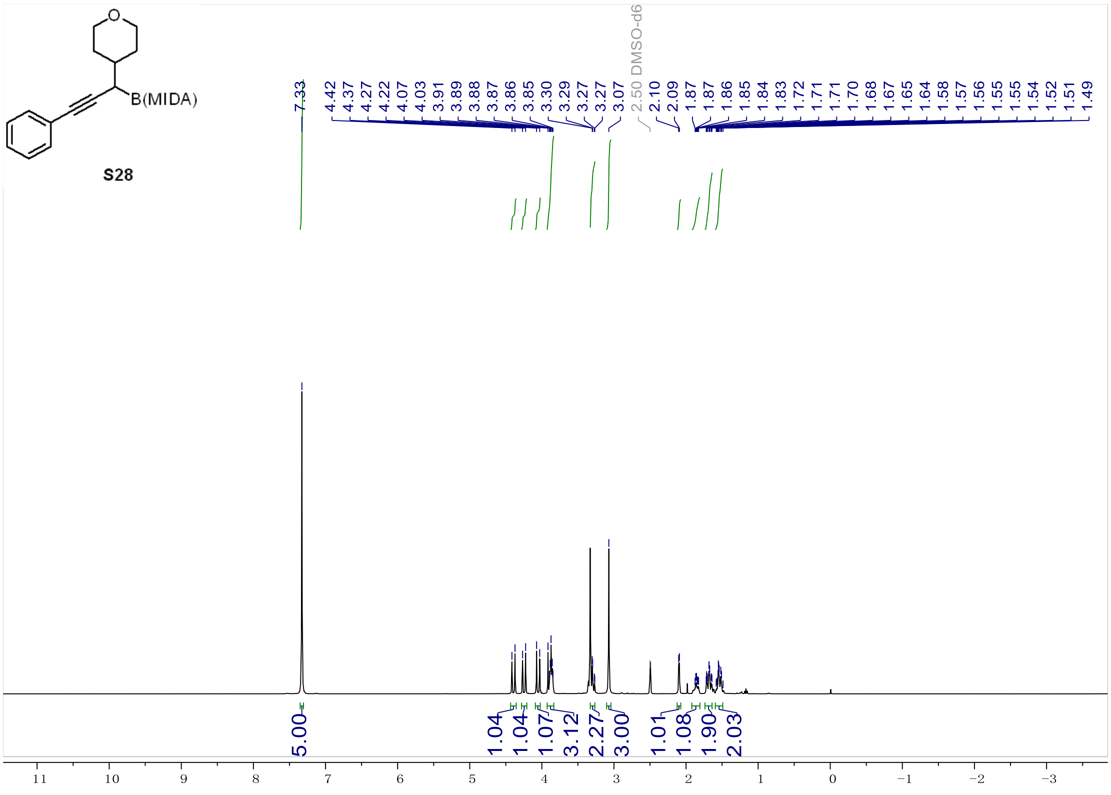

S28: ^{13}C NMR (101 MHz, DMSO-d_6)

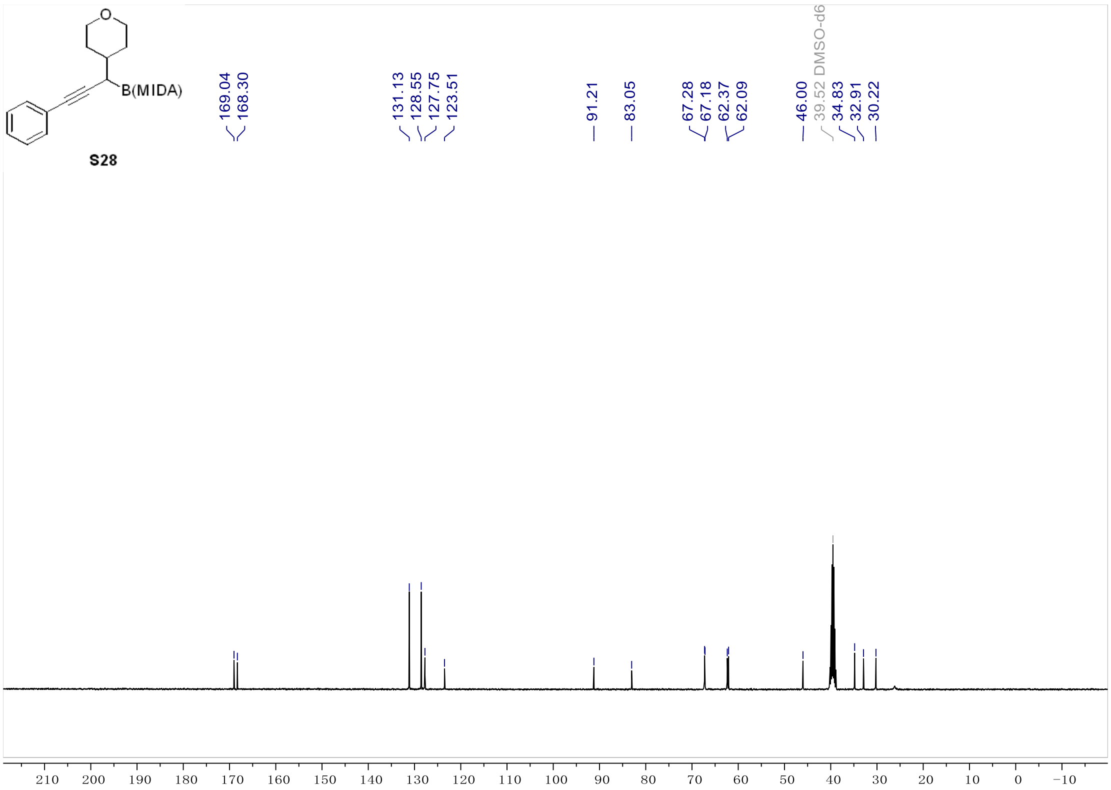

S29: ¹H NMR (500 MHz, DMSO-d_6)

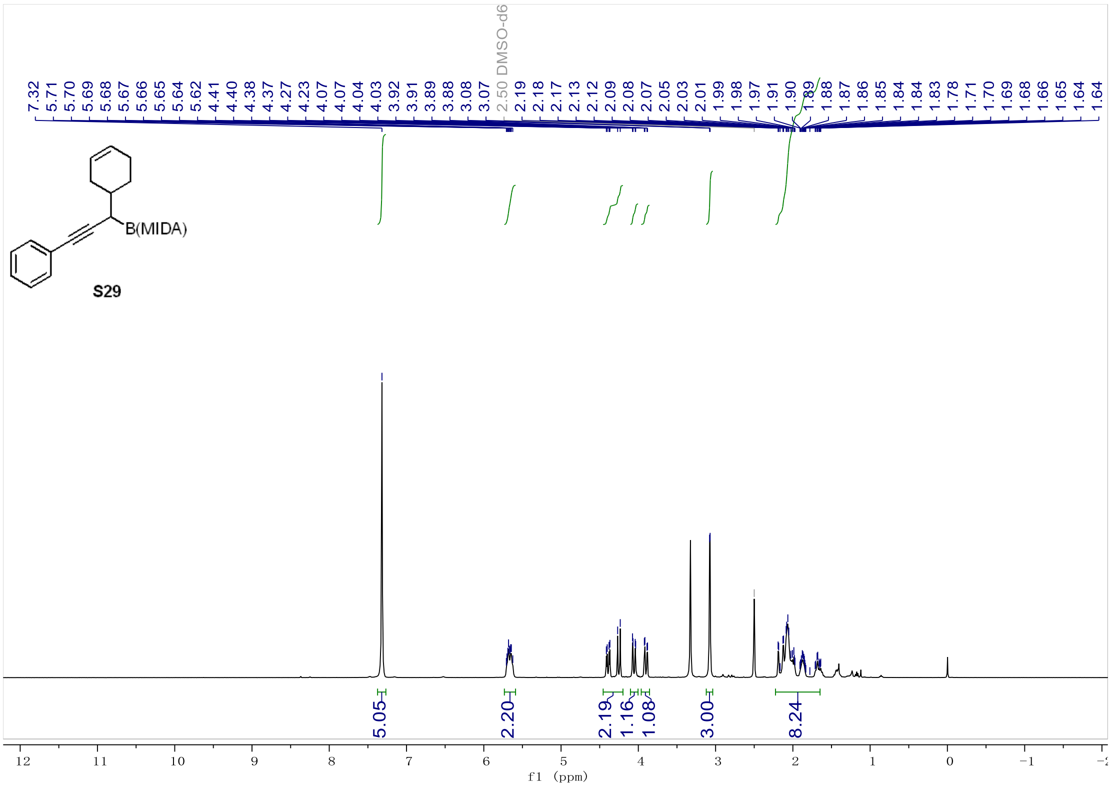

S29: ¹³C NMR (126 MHz, DMSO-d_6)

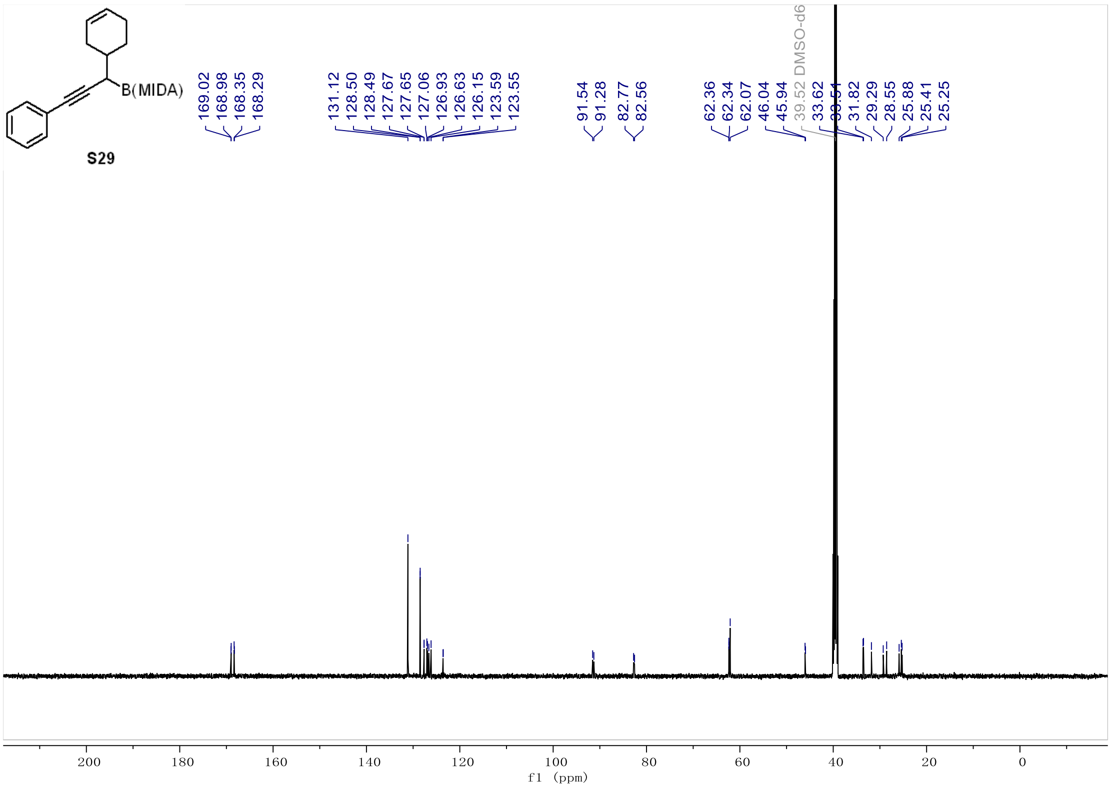

S30: ^{1}H NMR (400 MHz, DMSO-d_6)

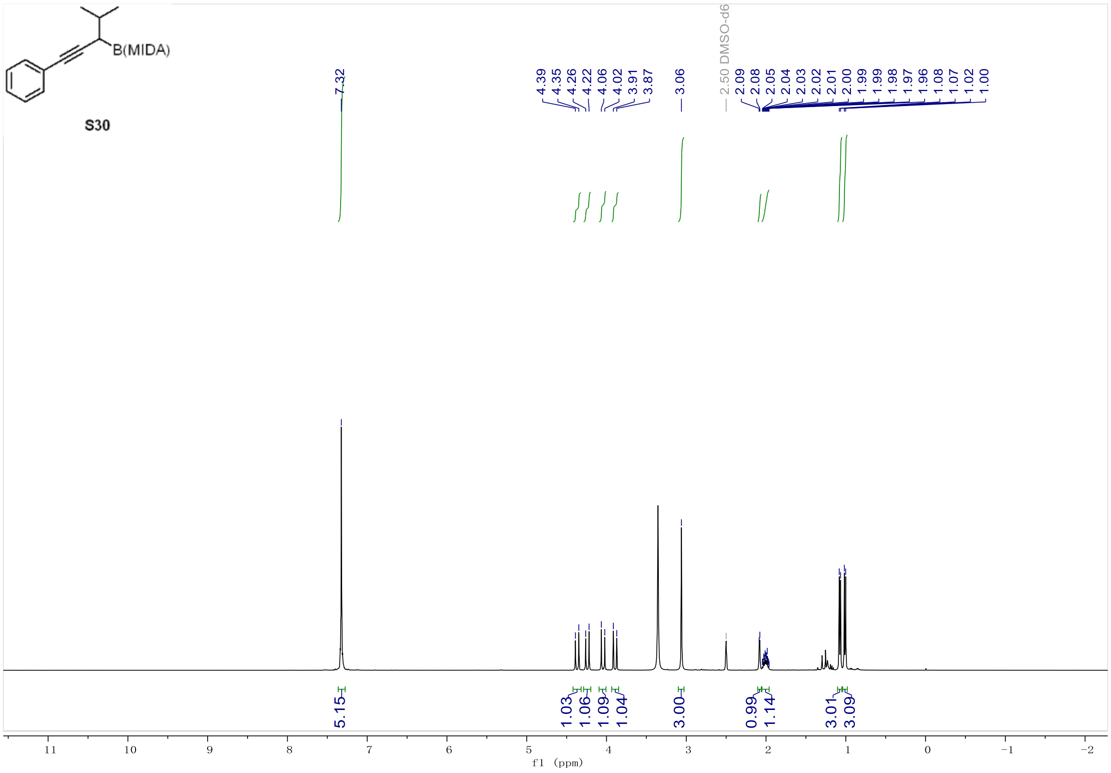

S30: ^{13}C NMR (126 MHz, Chloroform-*d*)

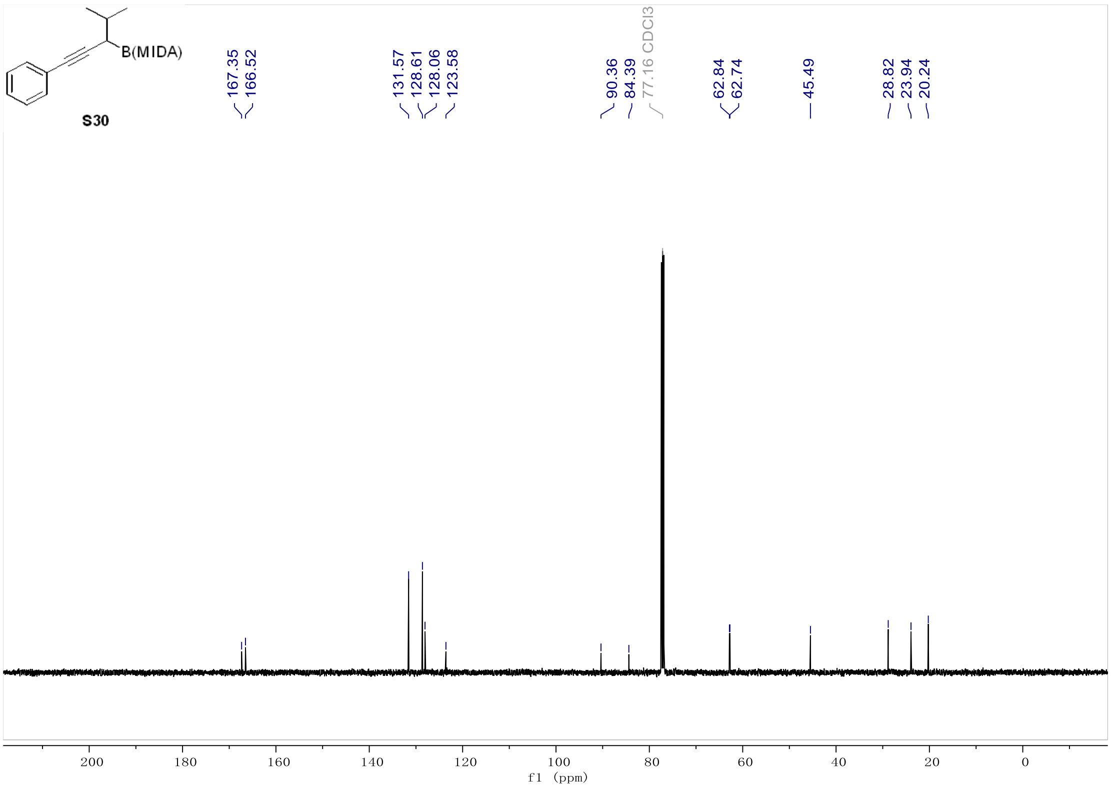

S31: ^{1}H NMR (400 MHz, Acetone-*d$_6$*)

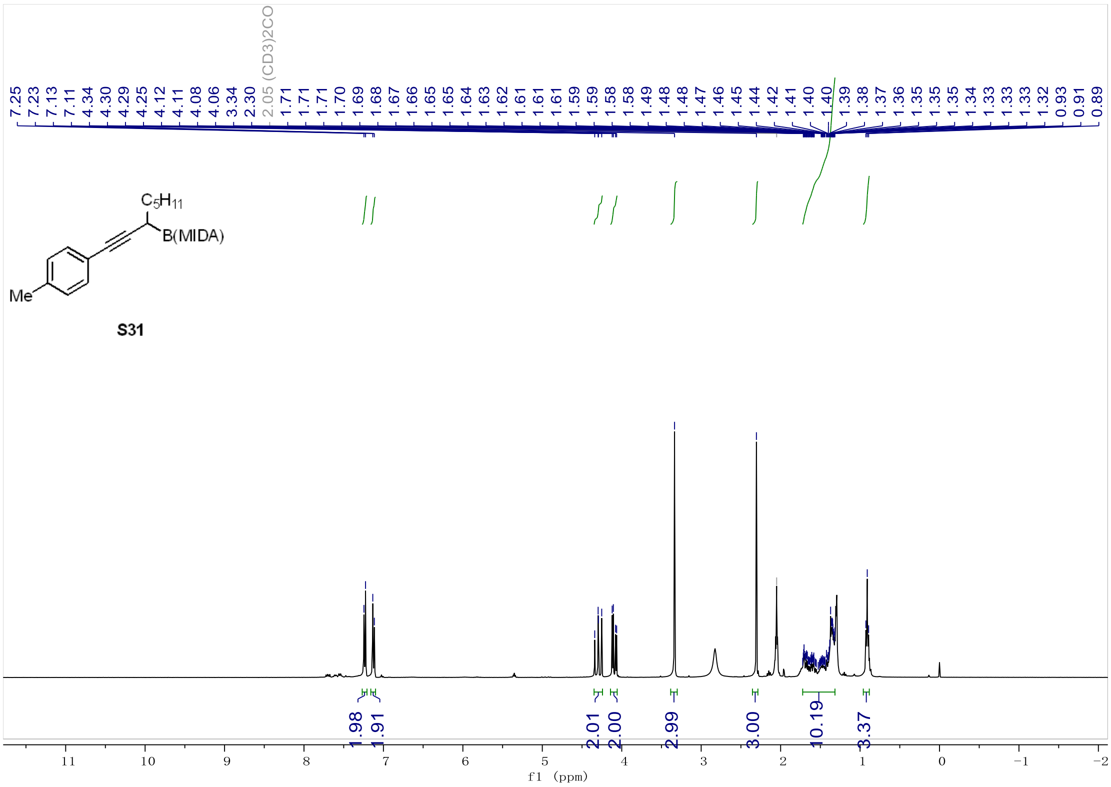

S31: ^{13}C NMR (126 MHz, Chloroform-*d*)

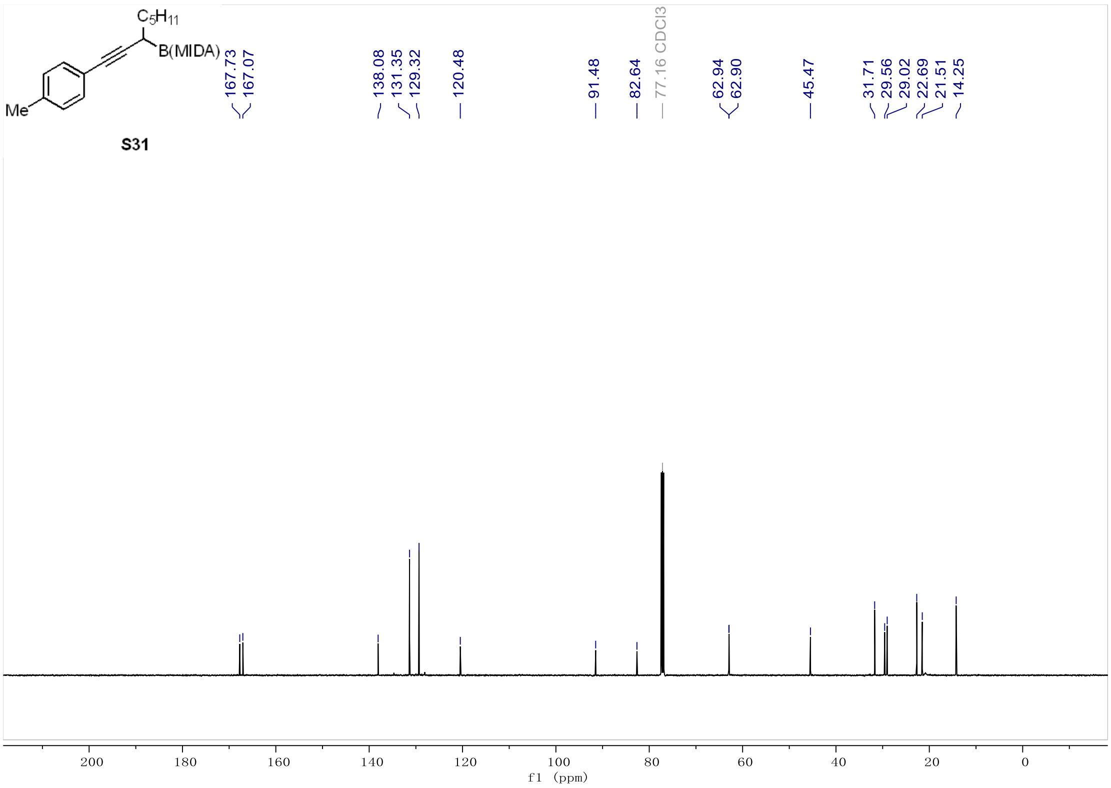

S32: ^{1}H NMR (400 MHz, DMSO-d_6)

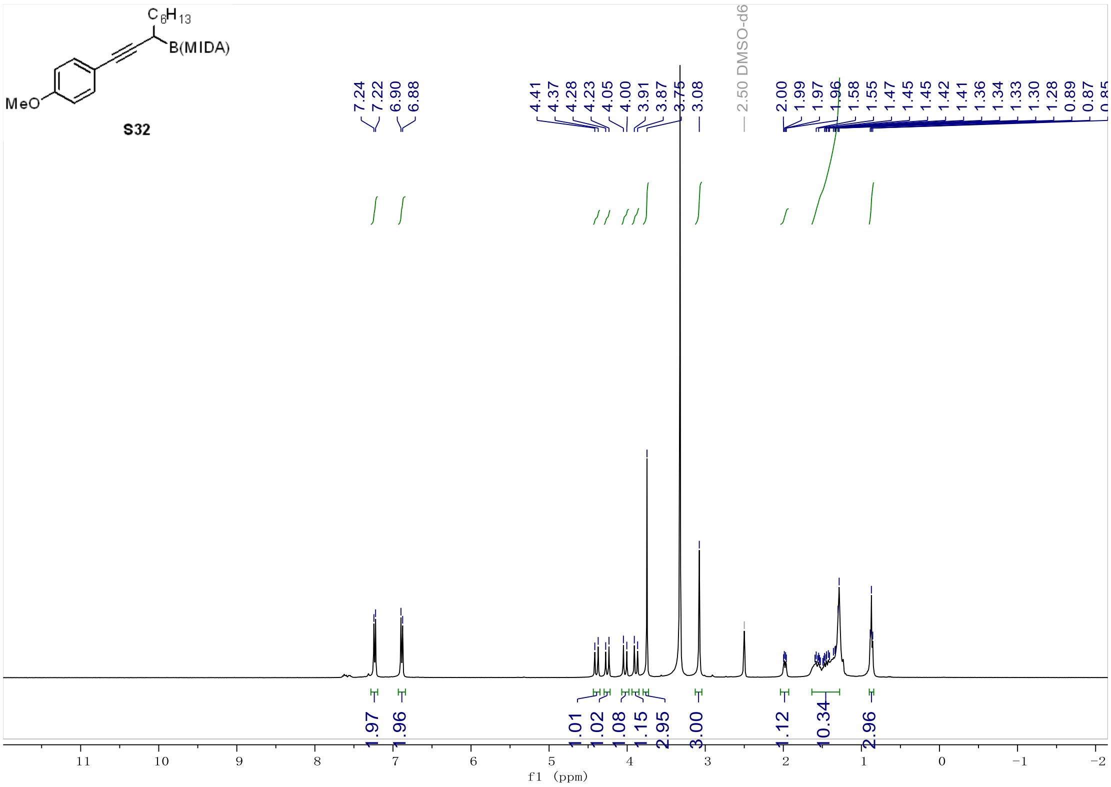

S32: ^{13}C NMR (101 MHz, DMSO-d_6)

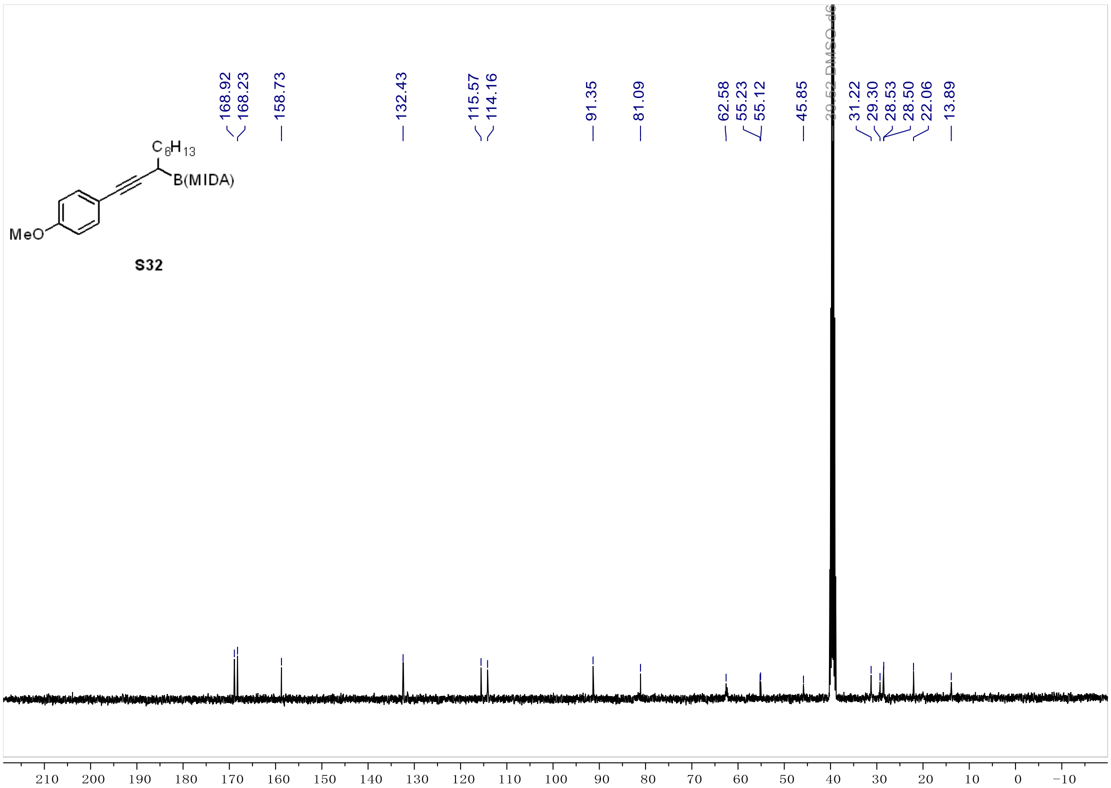

S33: ^{1}H NMR (400 MHz, DMSO-d_6)

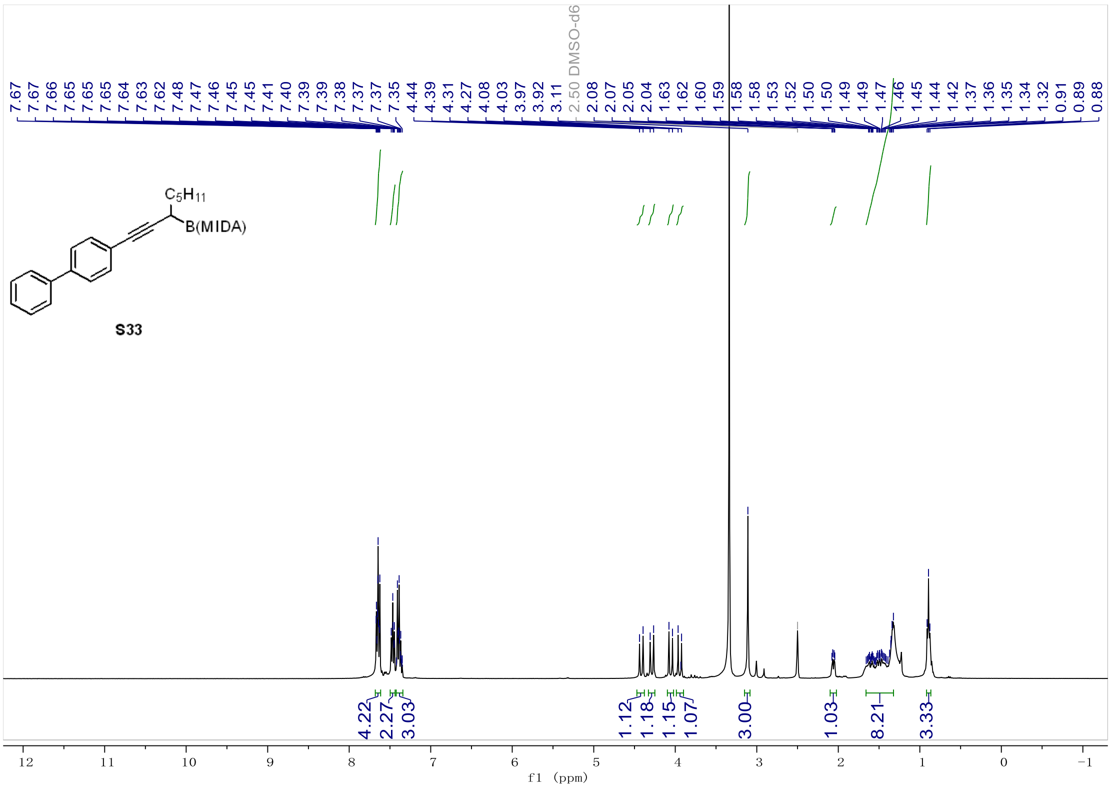

S33: ^{13}C NMR (101 MHz, DMSO-d_6)

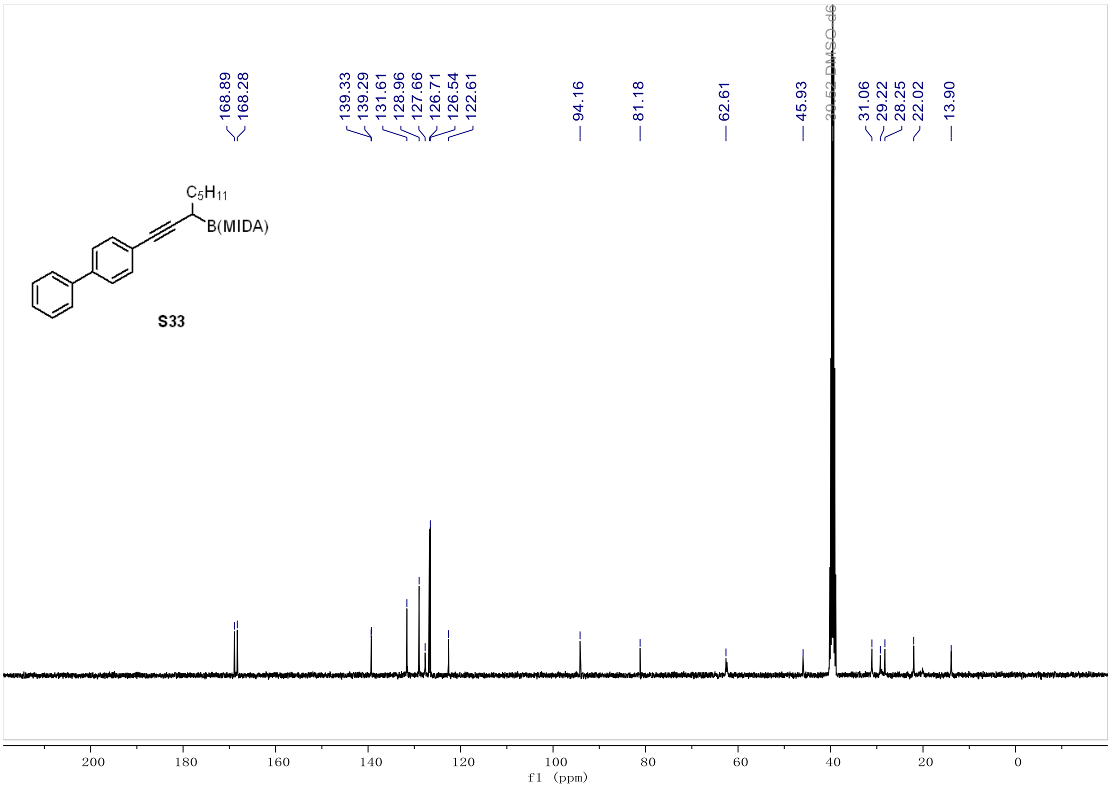

S34: ^{1}H NMR (500 MHz, DMSO-d_6)

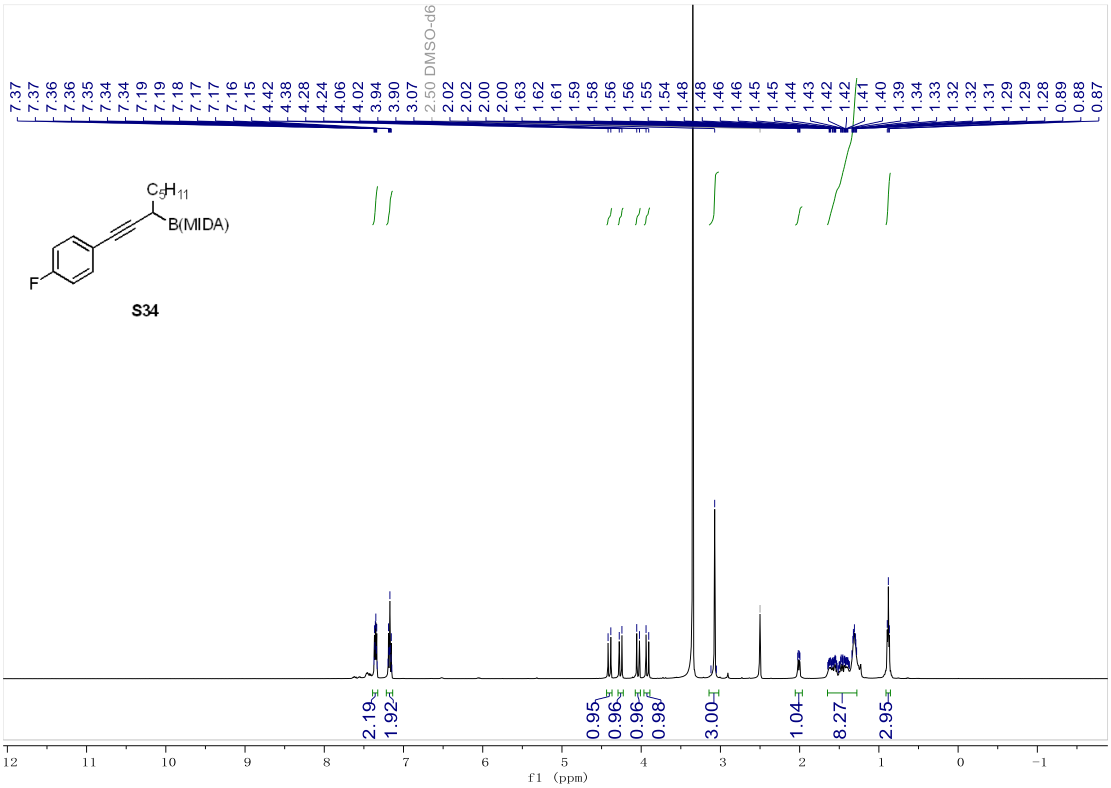

S34: ^{13}C NMR (126 MHz, DMSO-d_6)

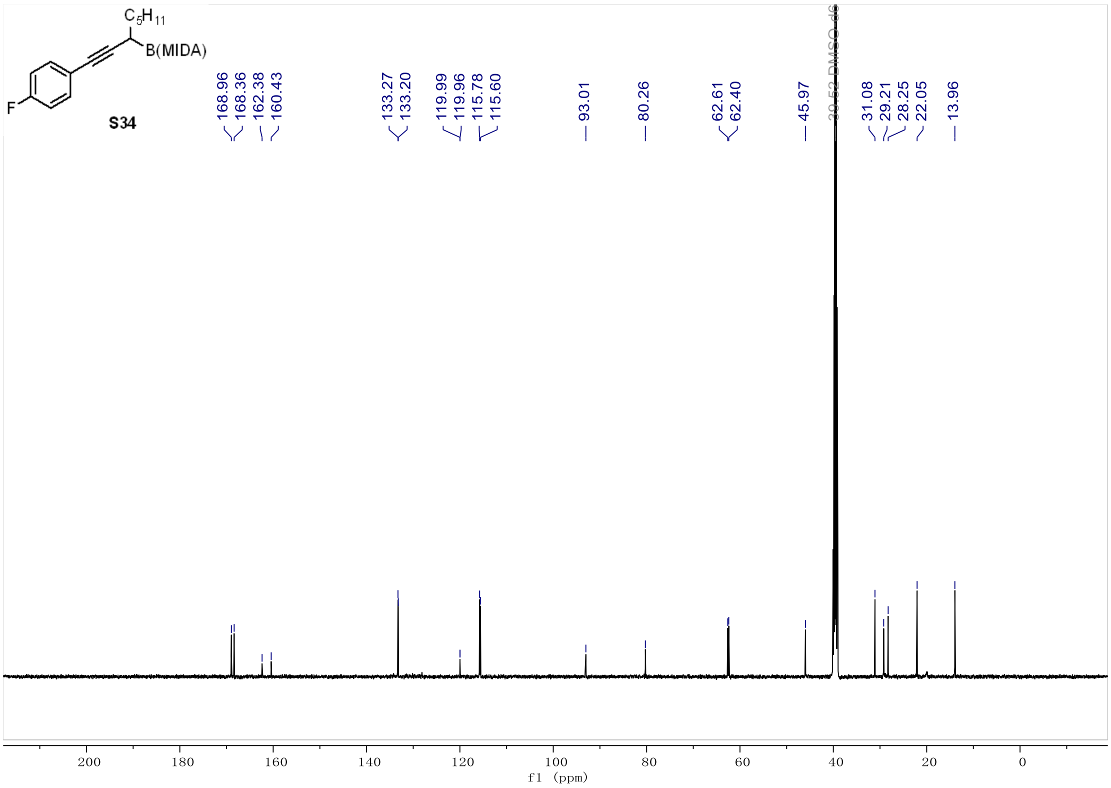

S35: ^{1}H NMR (500 MHz, DMSO-d_6)

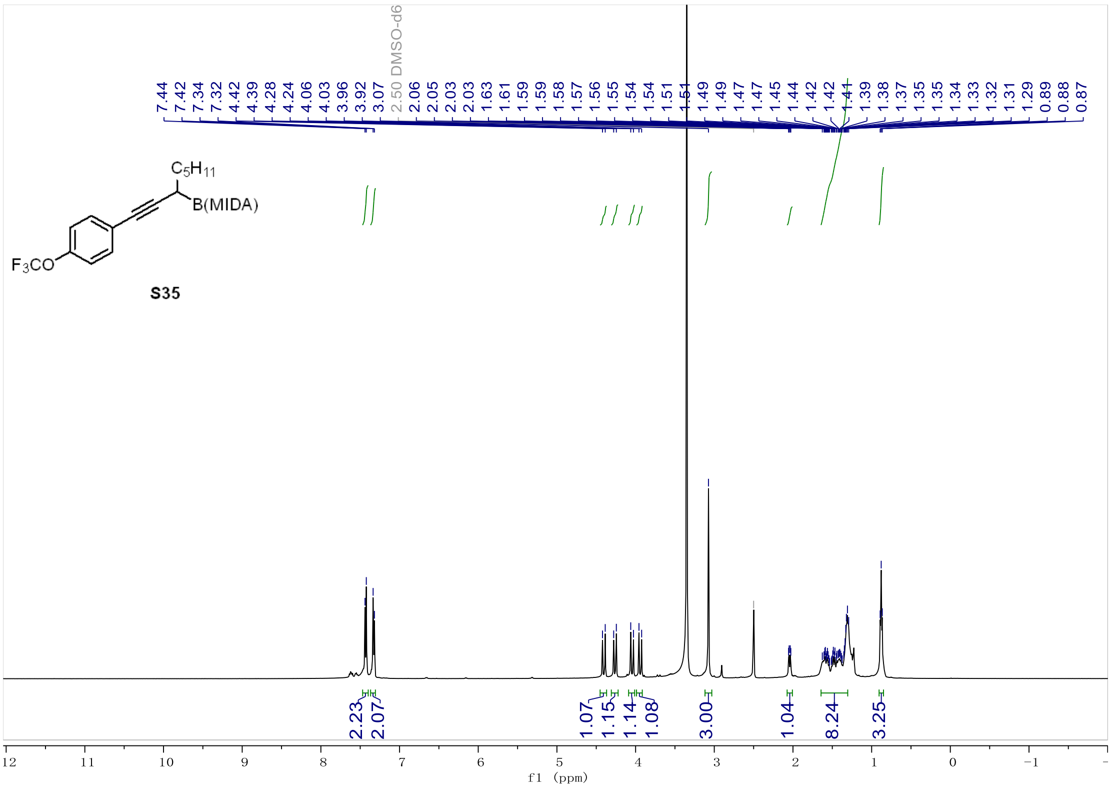

S35: ^{13}C NMR (101 MHz, DMSO-d_6)

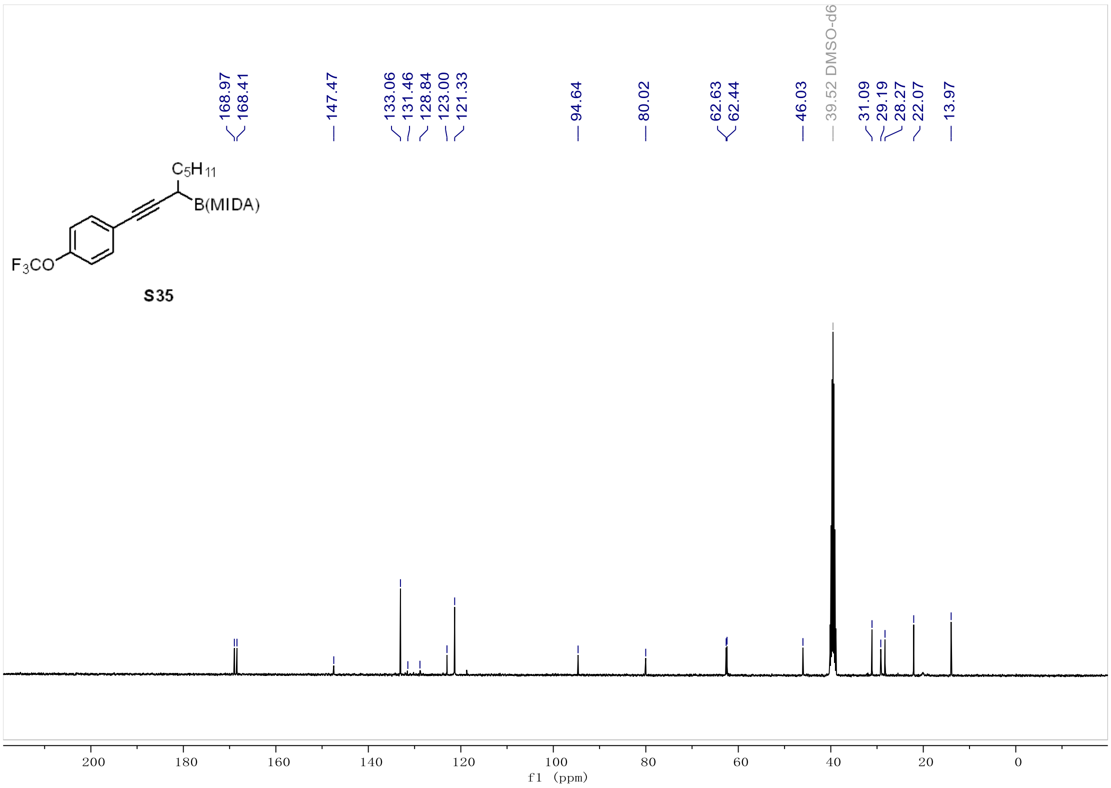

S36: ^{1}H NMR (400 MHz, DMSO-d_6)

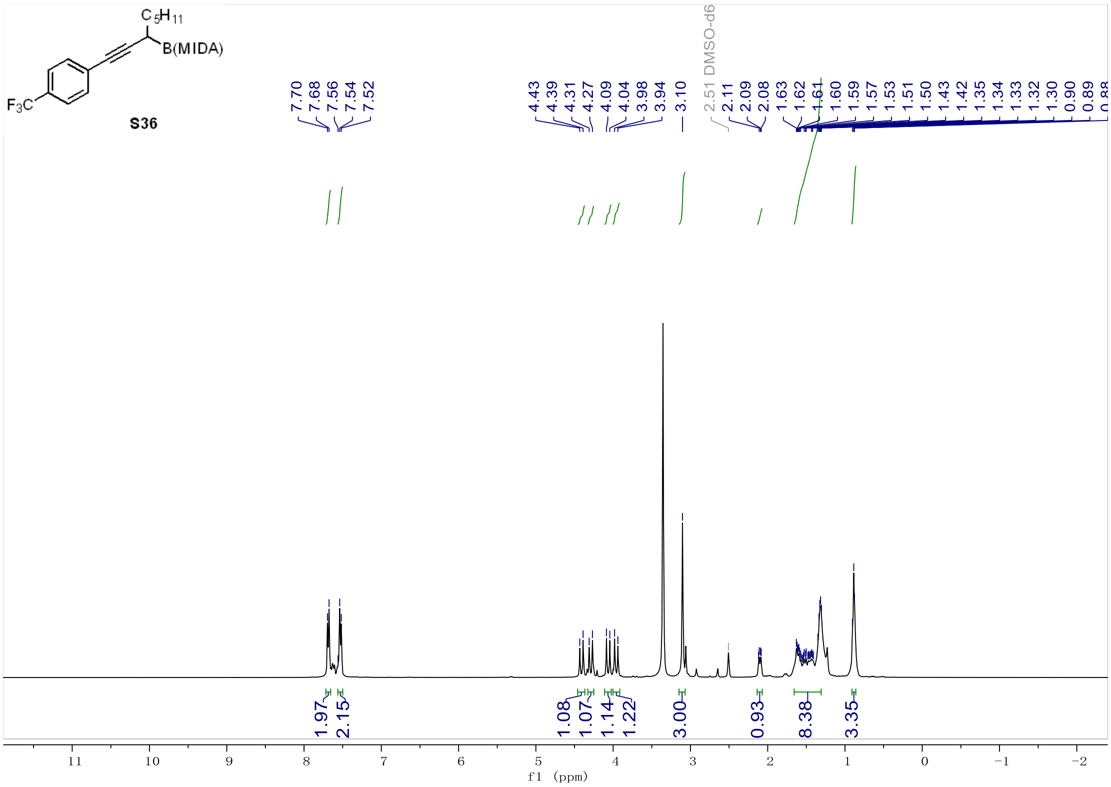

S36: ^{13}C NMR (101 MHz, DMSO-d_6)

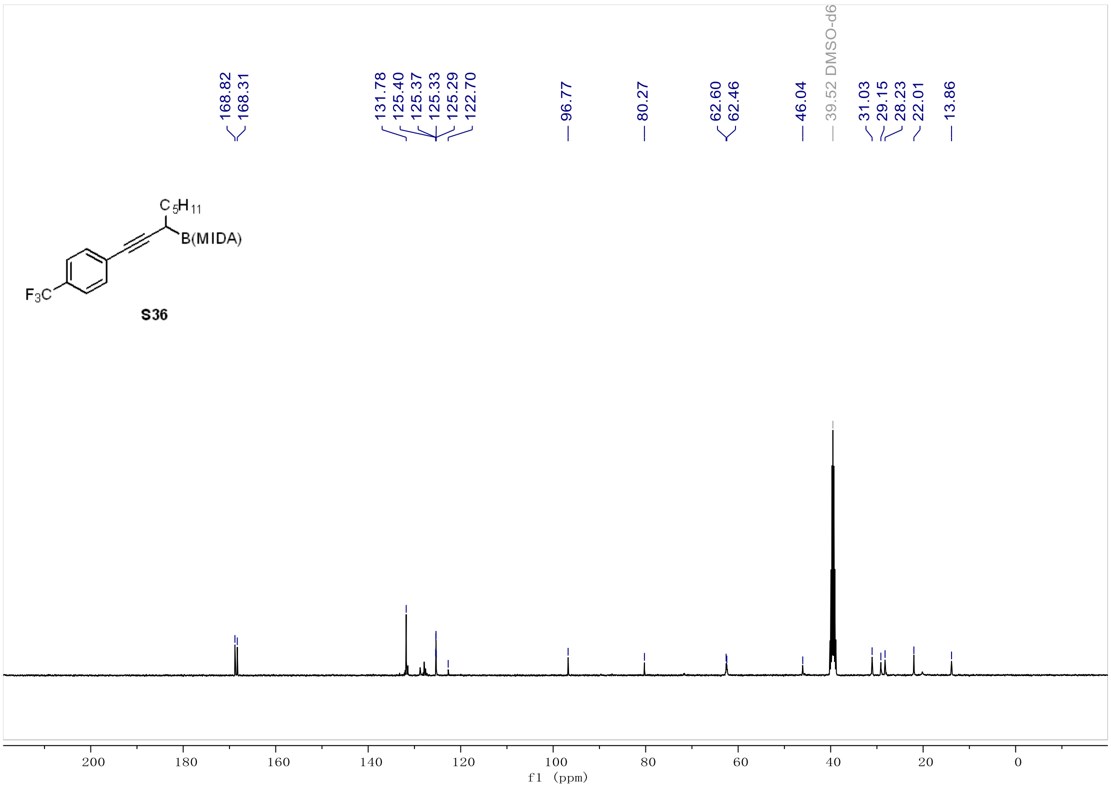

S37: ^{1}H NMR (400 MHz, Chloroform-*d*)

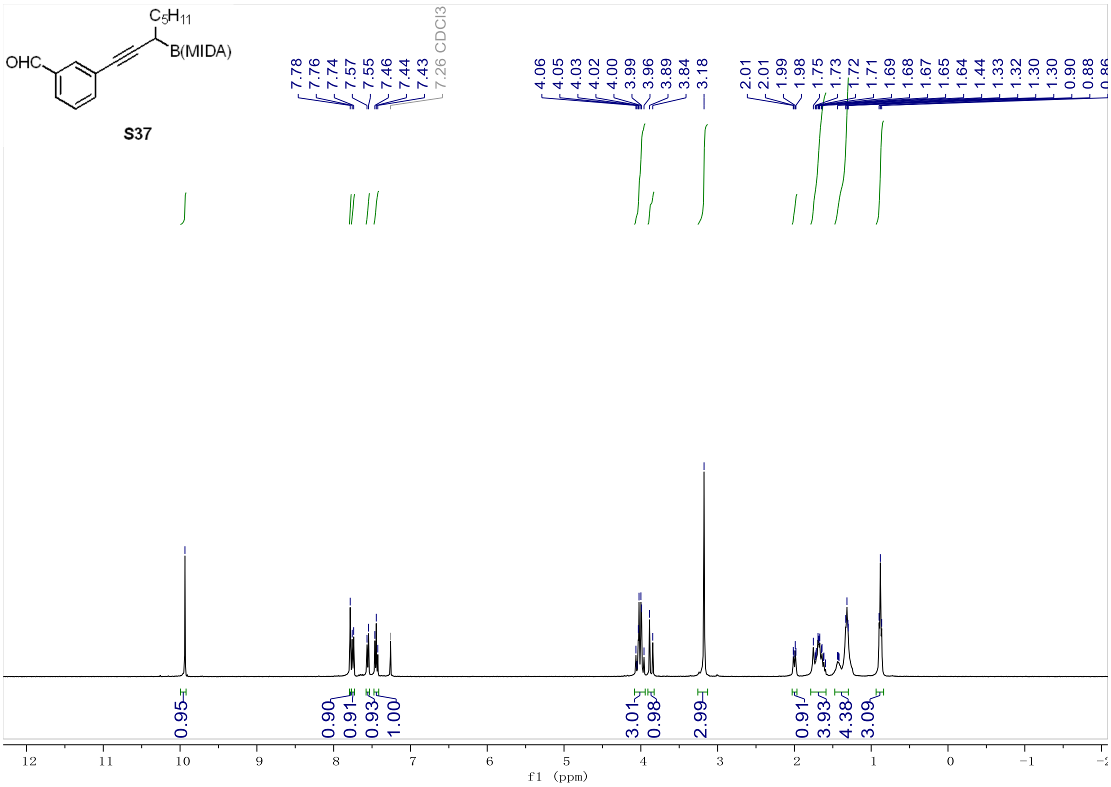

S37: ^{13}C NMR (101 MHz, Chloroform-*d*)

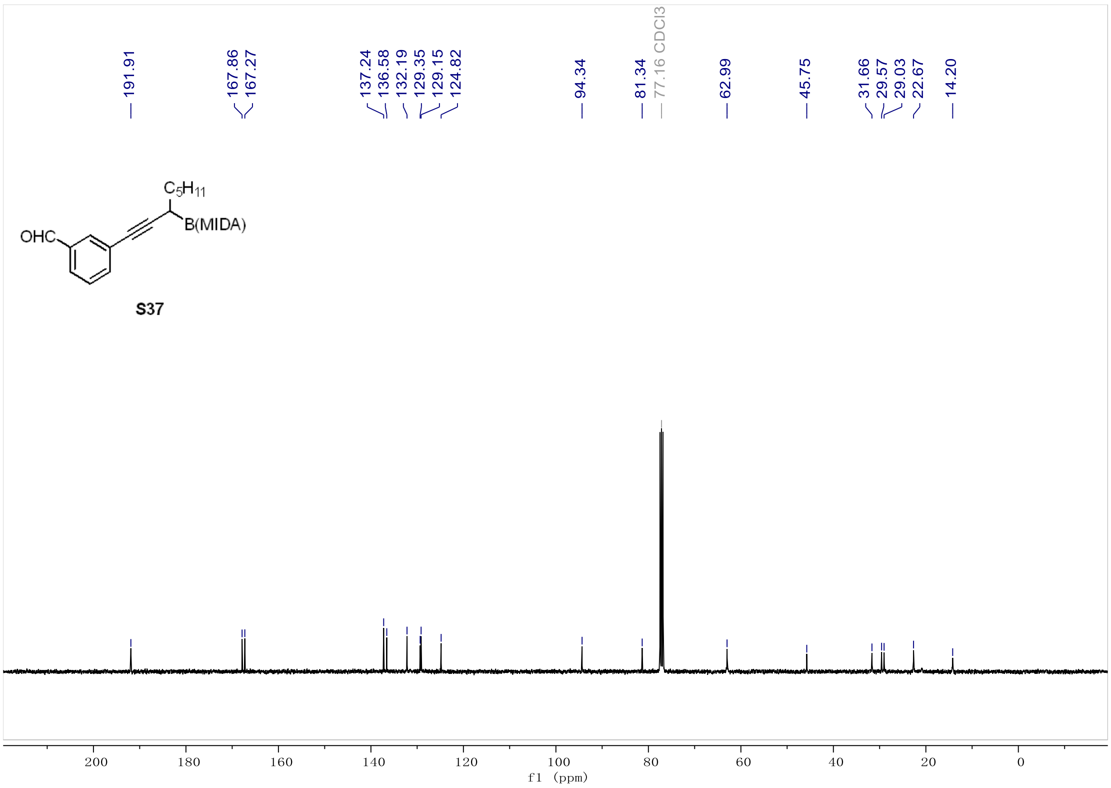

S38: ^{1}H NMR (400 MHz, DMSO-d_6)

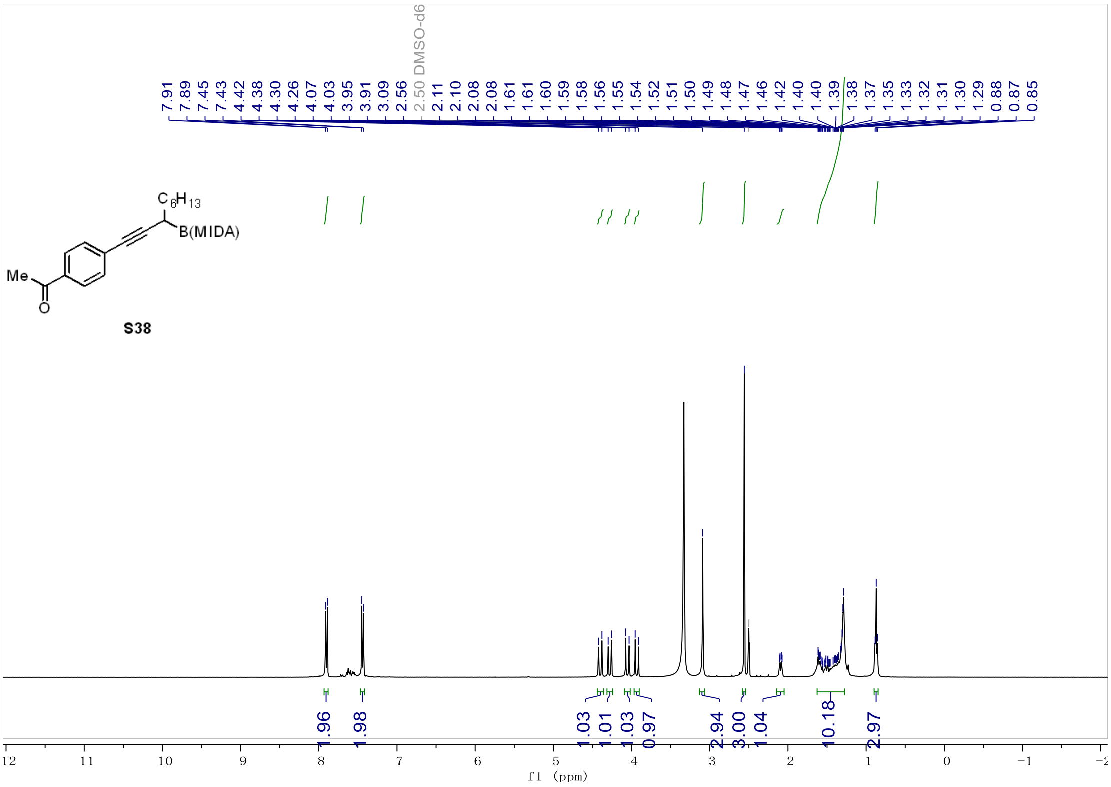

S38: ^{13}C NMR (101 MHz, DMSO-d_6)

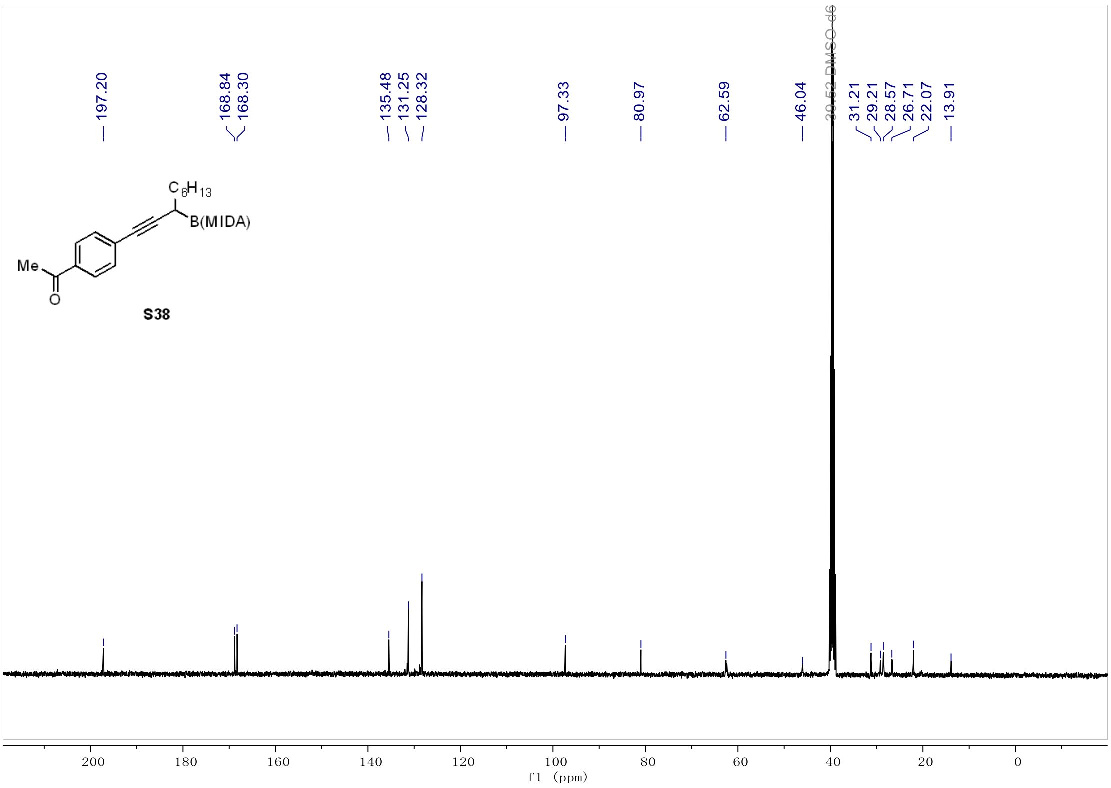

S39: ^{1}H NMR (400 MHz, Chloroform-*d*)

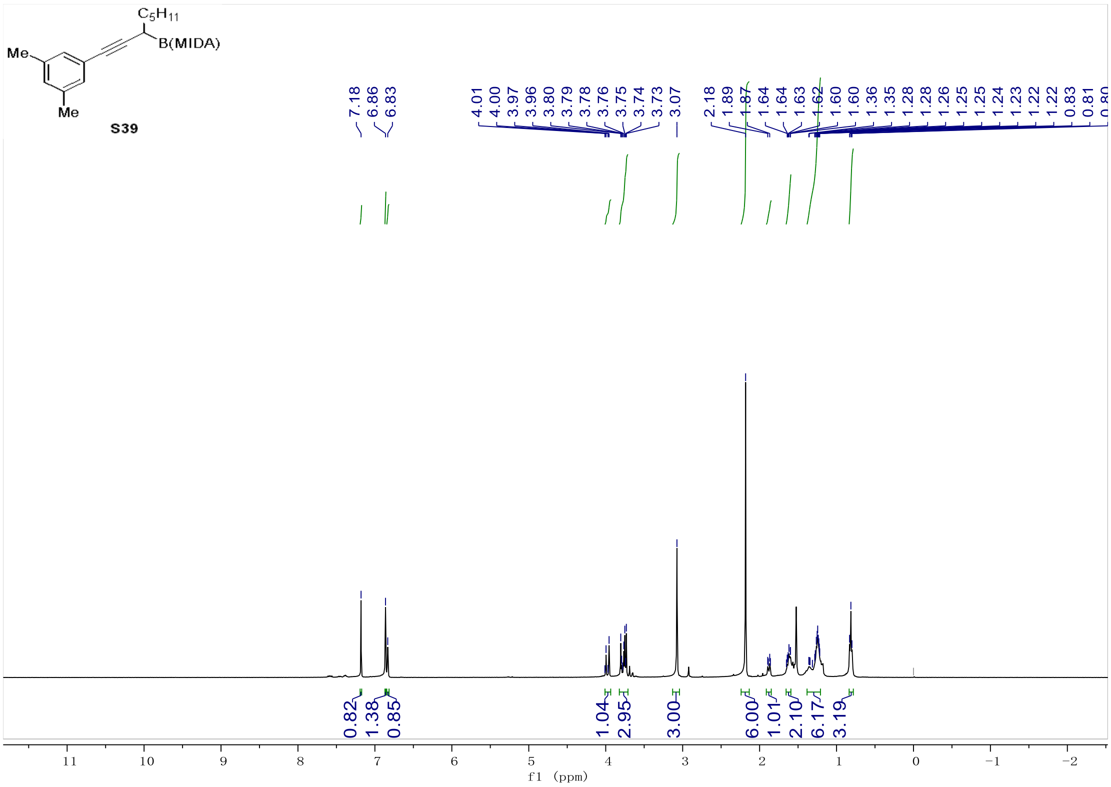

S39: ^{13}C NMR (101 MHz, Chloroform-*d*)

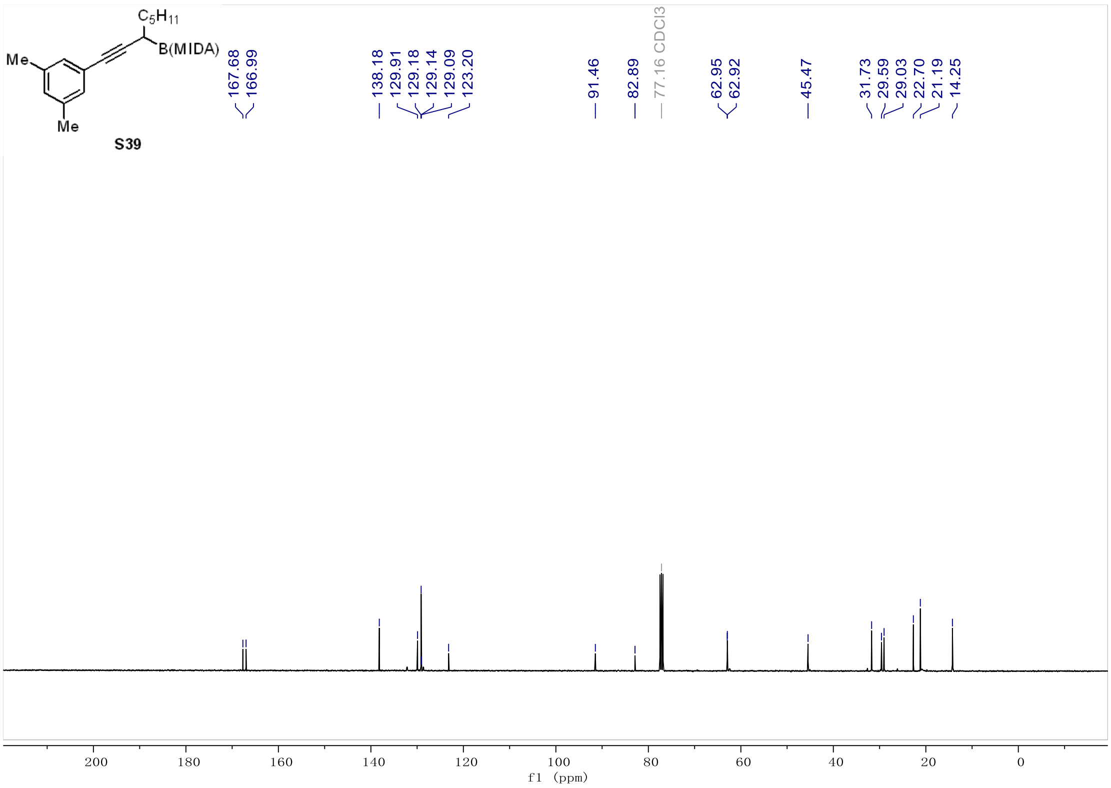

S40: ^{1}H NMR (400 MHz, DMSO-d_6)

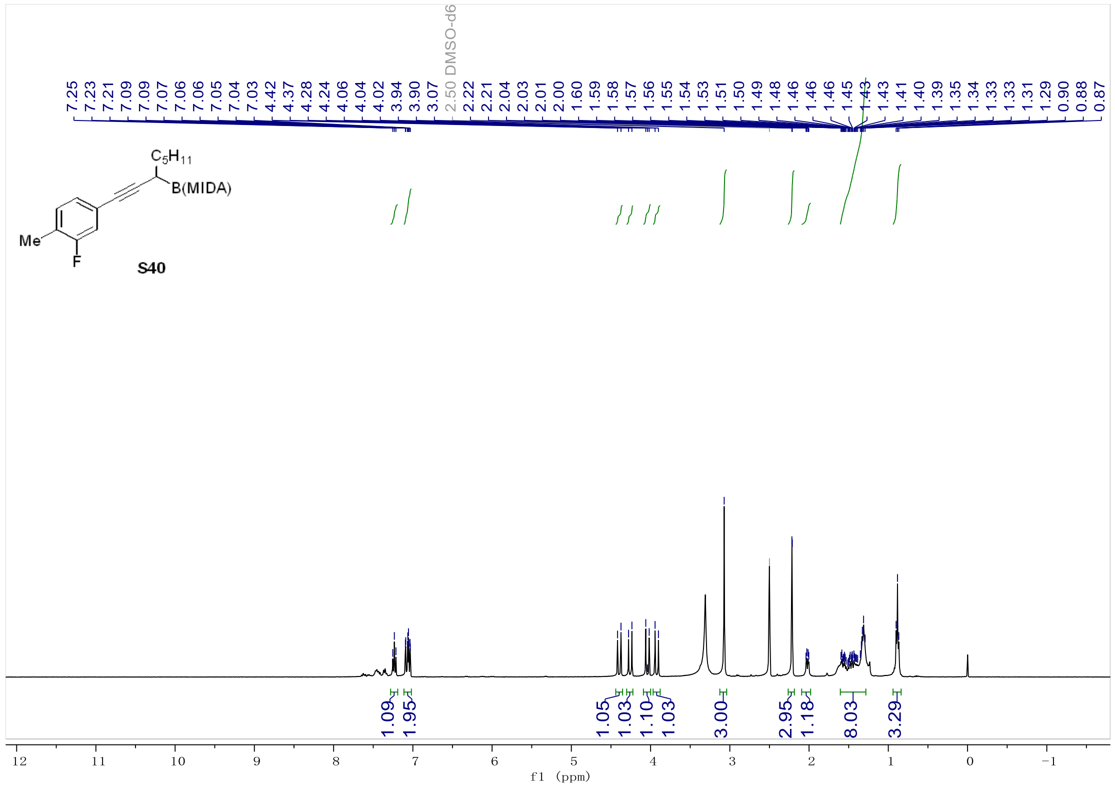

S40: ^{13}C NMR (101 MHz, DMSO-d_6)

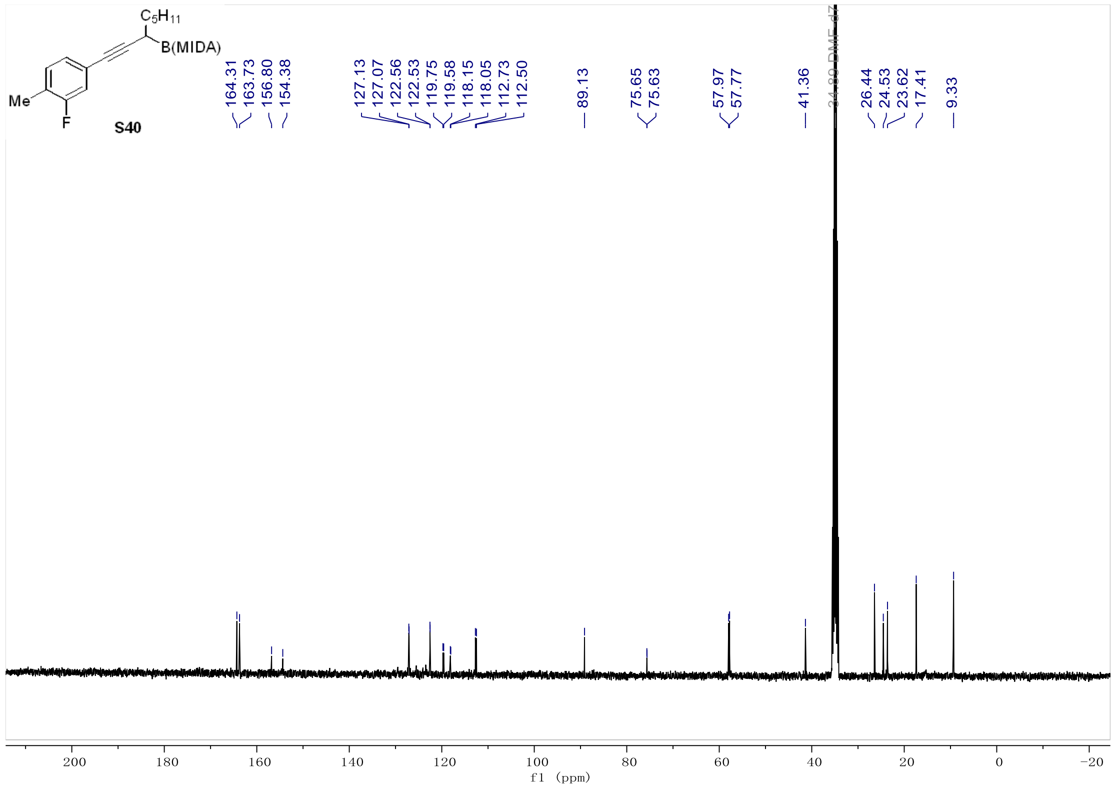

S41: ^{1}H NMR (400 MHz, DMSO-d_6)

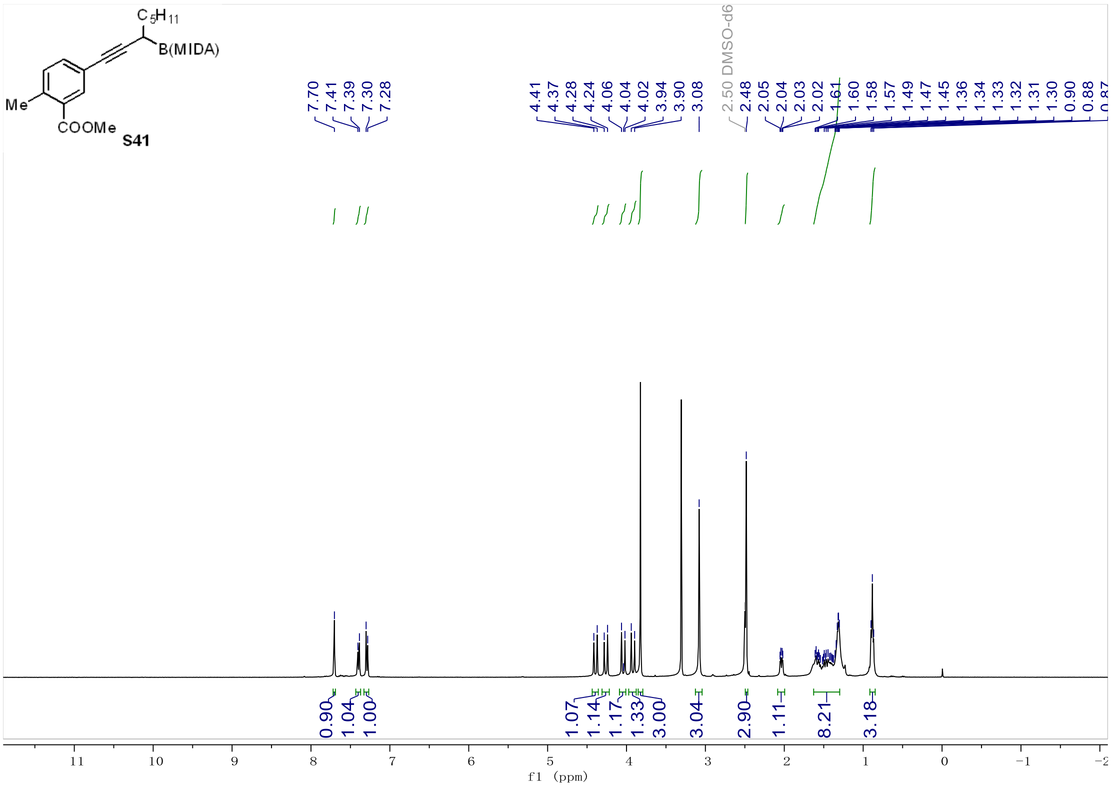

S41: ^{13}C NMR (126 MHz, DMSO-d_6)

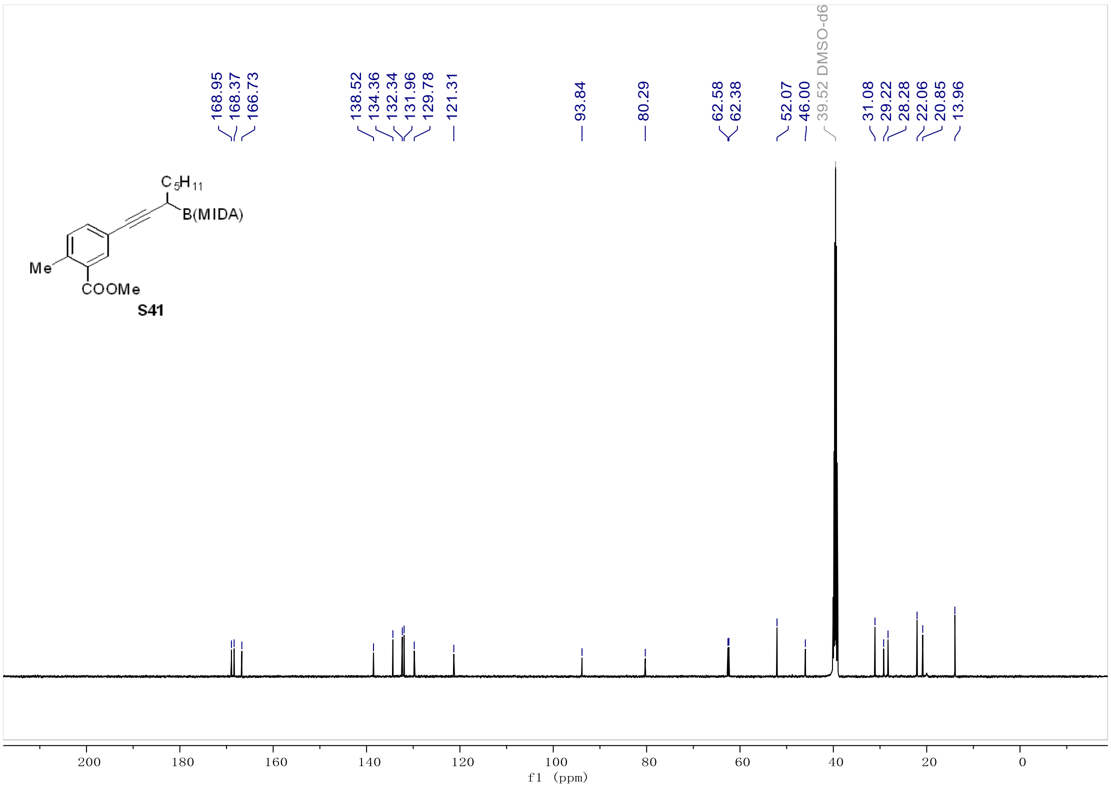

S42: ^{1}H NMR (500 MHz, Chloroform-*d*)

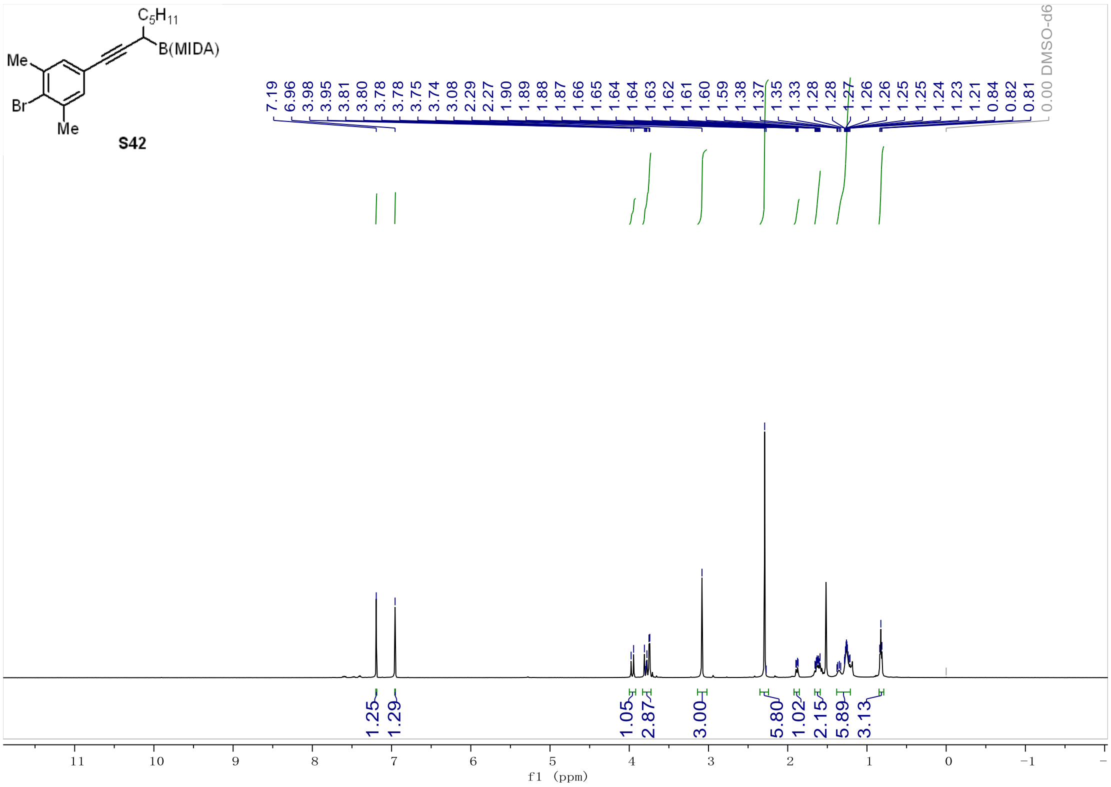

S42: ^{13}C NMR (126 MHz, Chloroform-*d*)

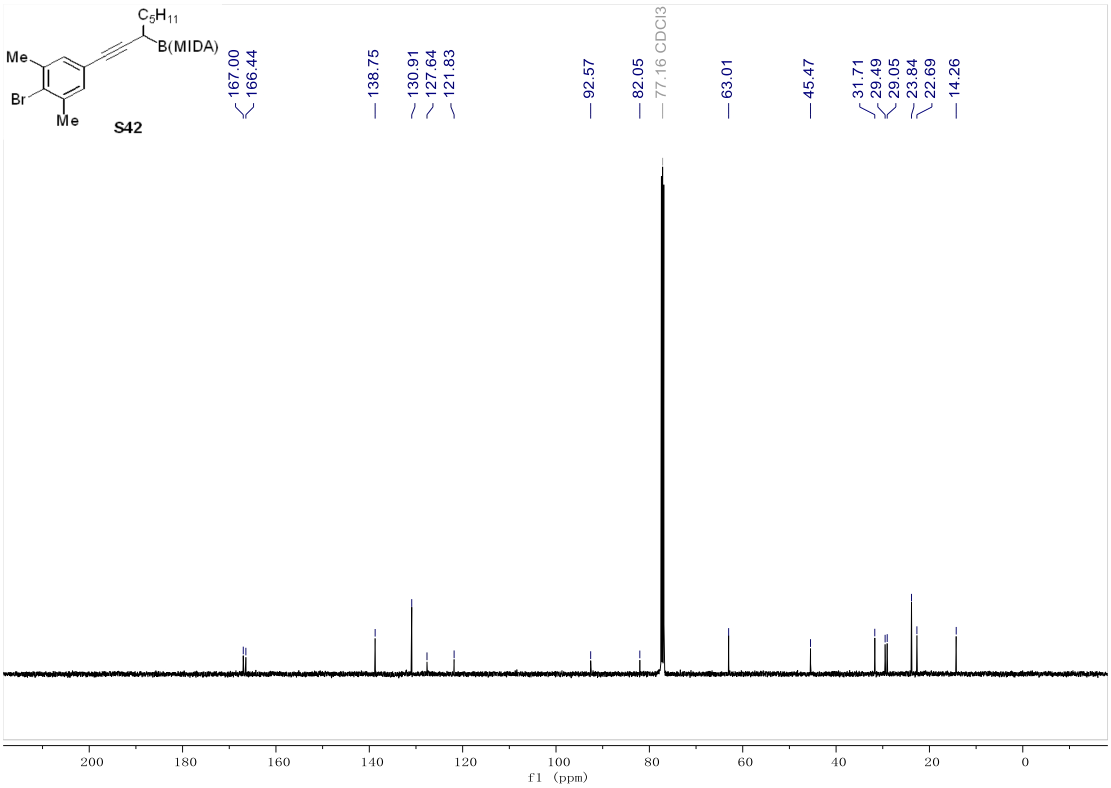

S43: ^{1}H NMR (400 MHz, DMSO-d_6)

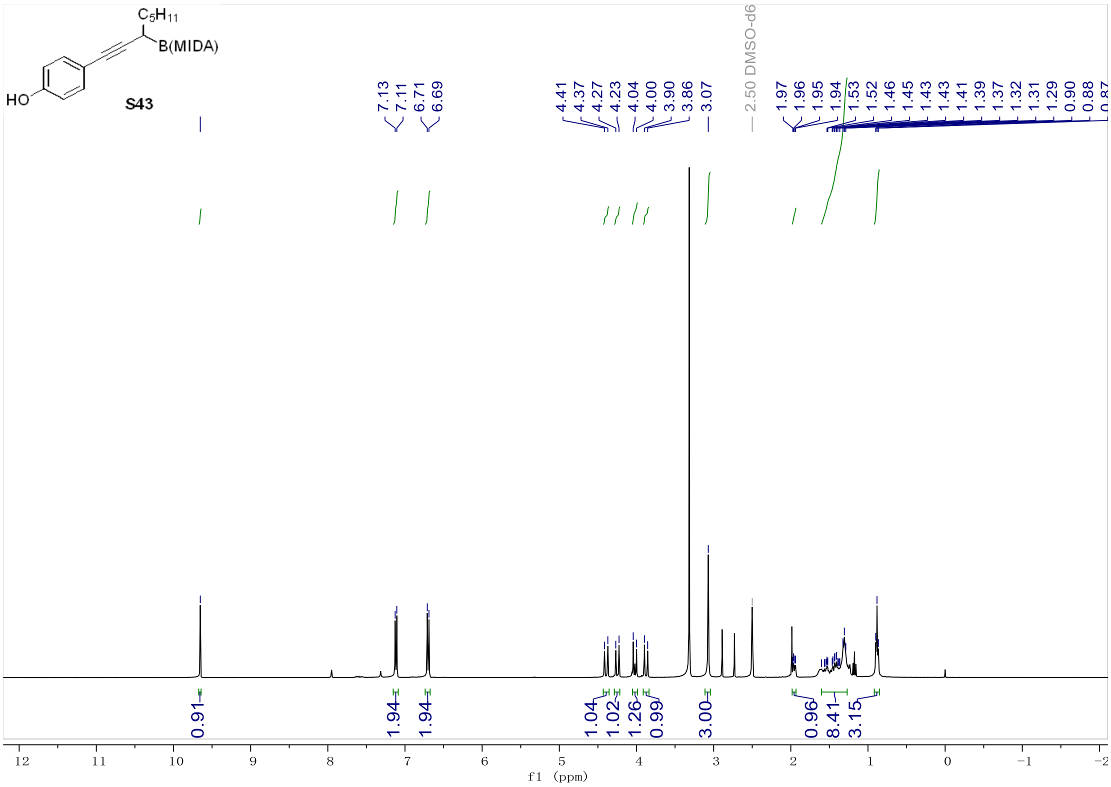

S43: ^{13}C NMR (126 MHz, DMSO-d_6)

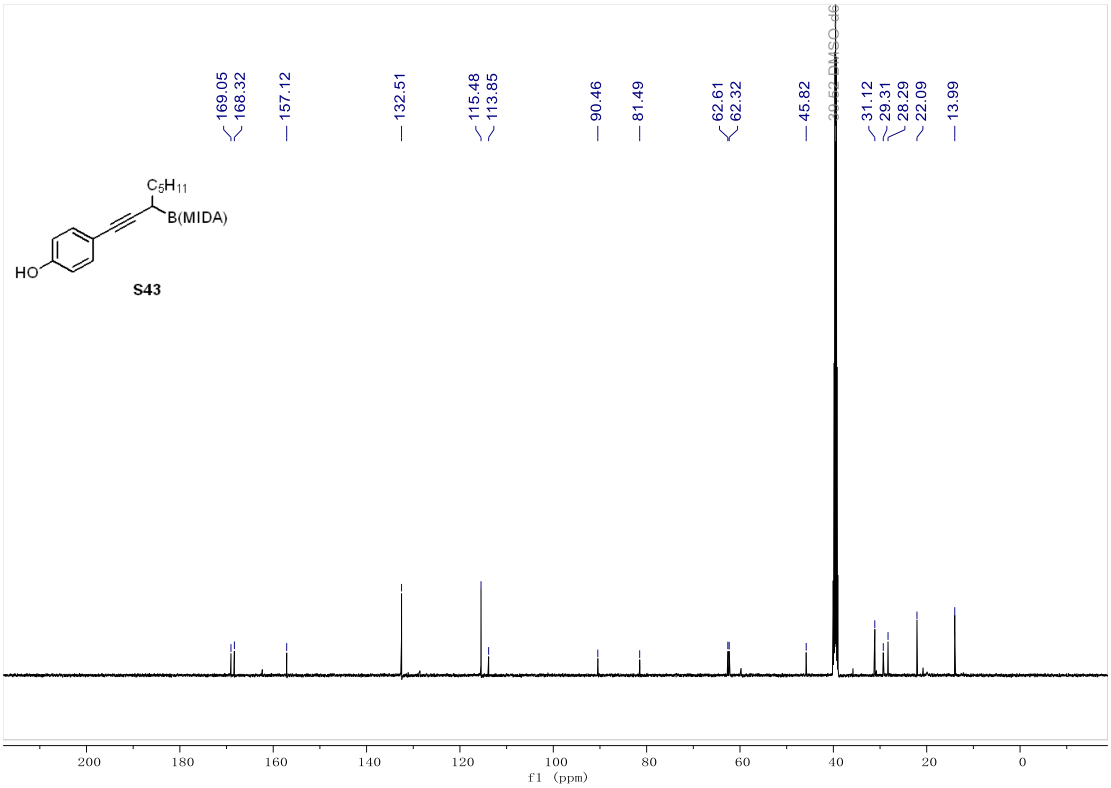

S44: ^{1}H NMR (400 MHz, Chloroform-*d*)

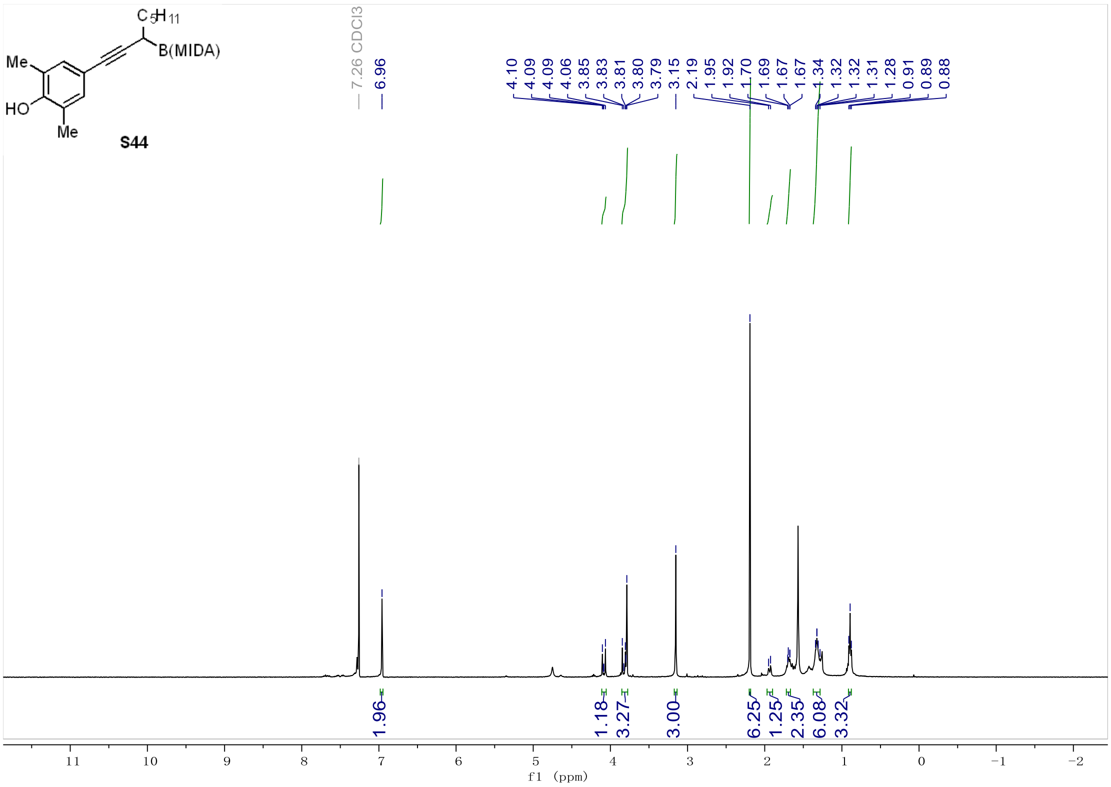

S44: ^{13}C NMR (126 MHz, Chloroform-*d*)

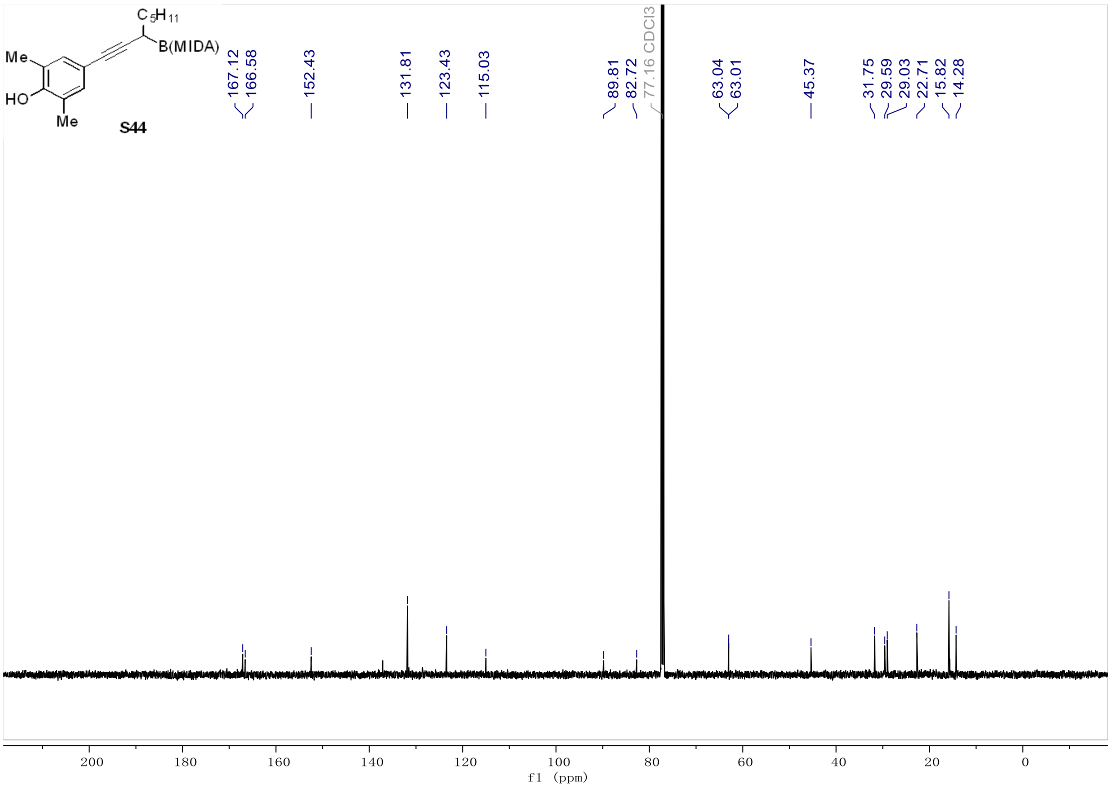

S45: ^{1}H NMR (500 MHz, Chloroform-*d*)

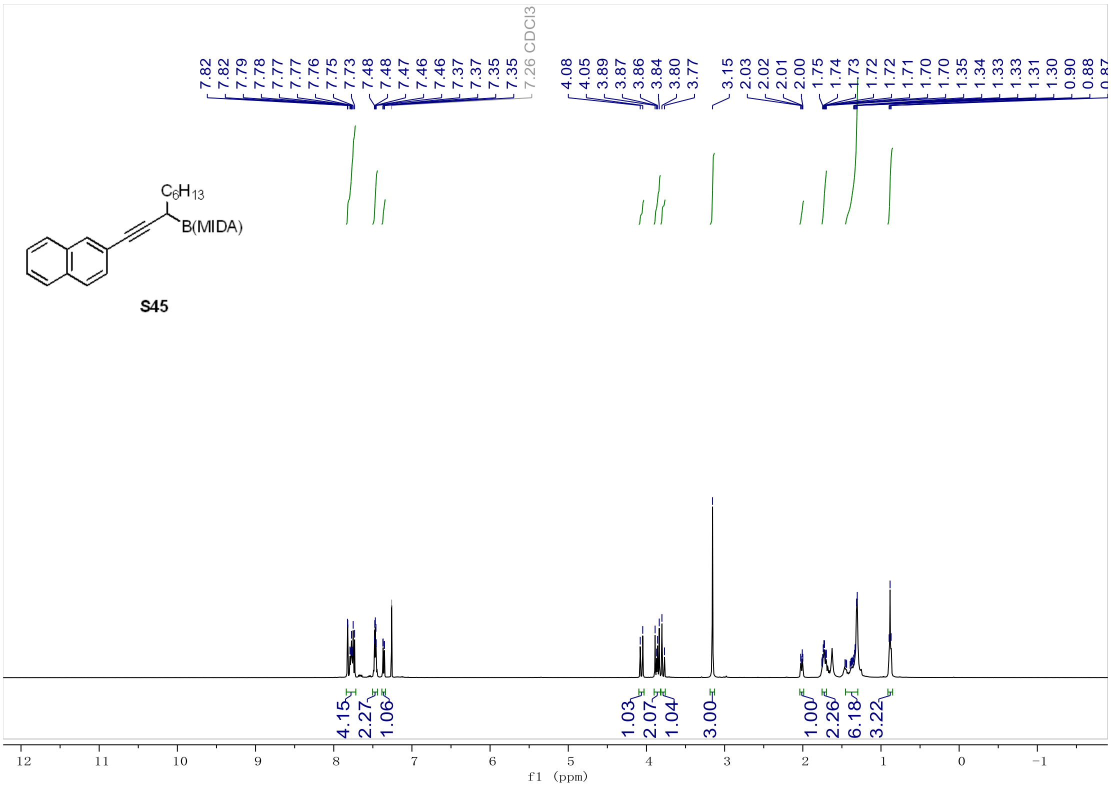

S45: ^{13}C NMR (126 MHz, Chloroform-*d*)

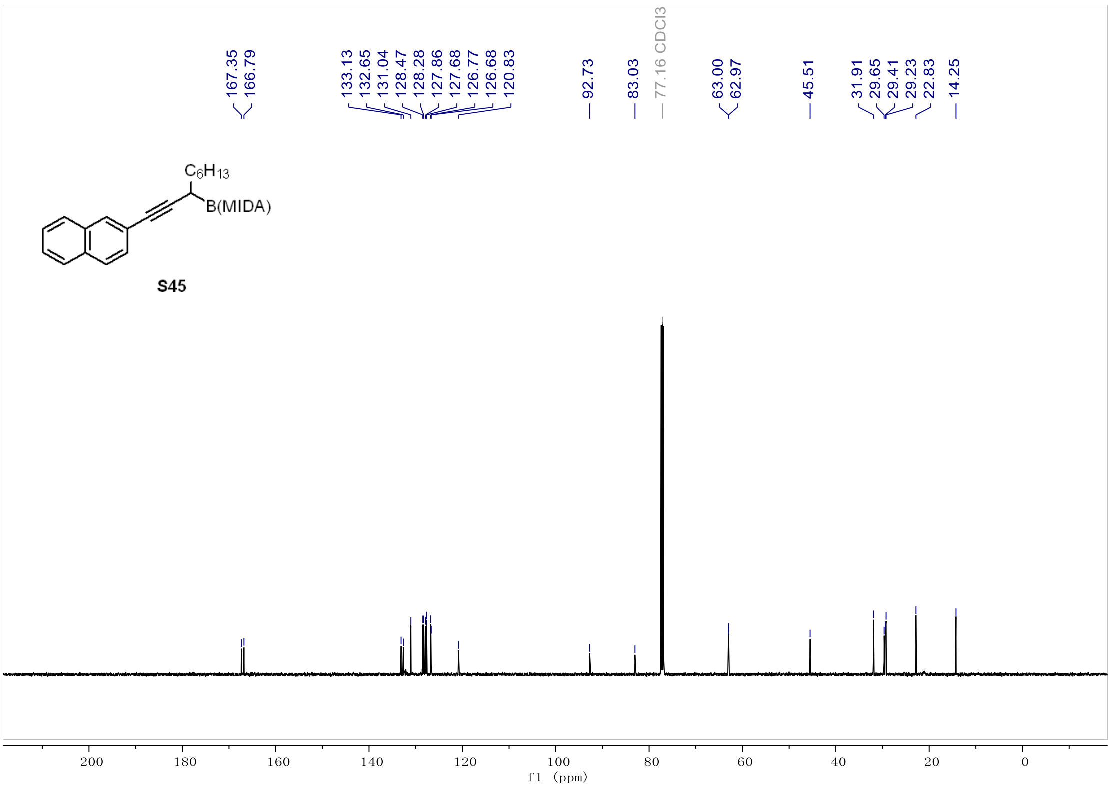

S46: ^{1}H NMR (400 MHz, DMSO-d_6)

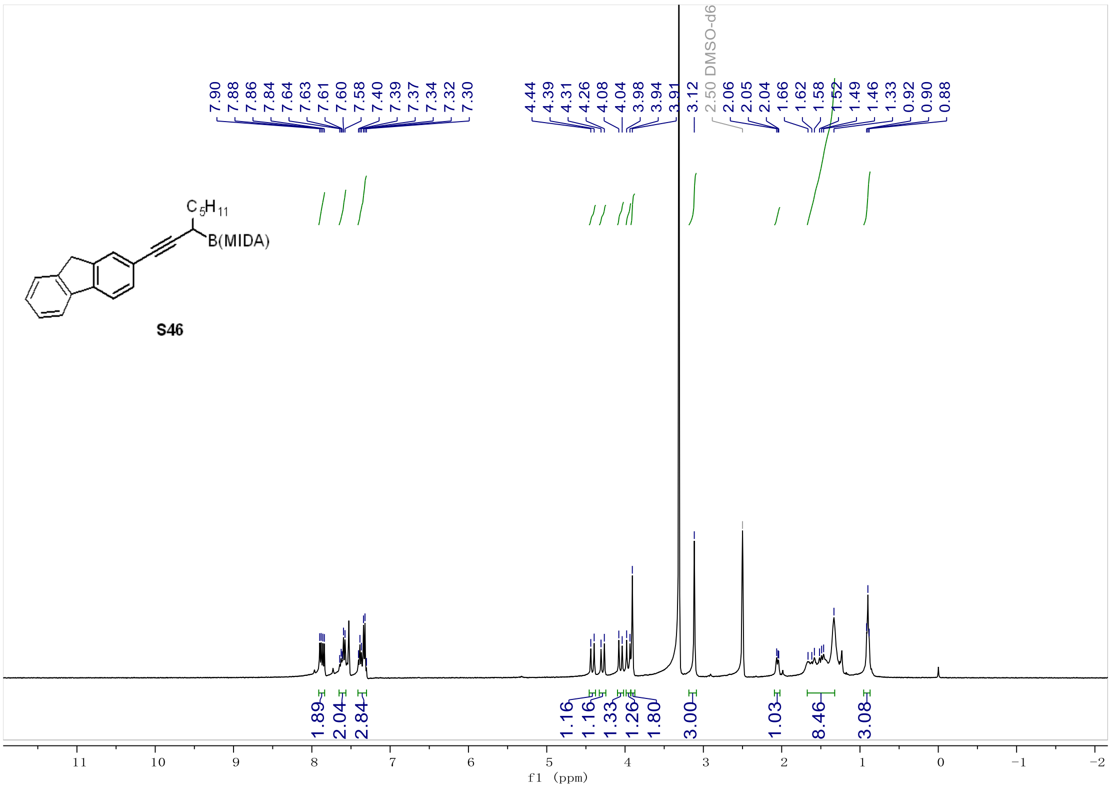

S46: ^{13}C NMR (126 MHz, DMSO-d_6)

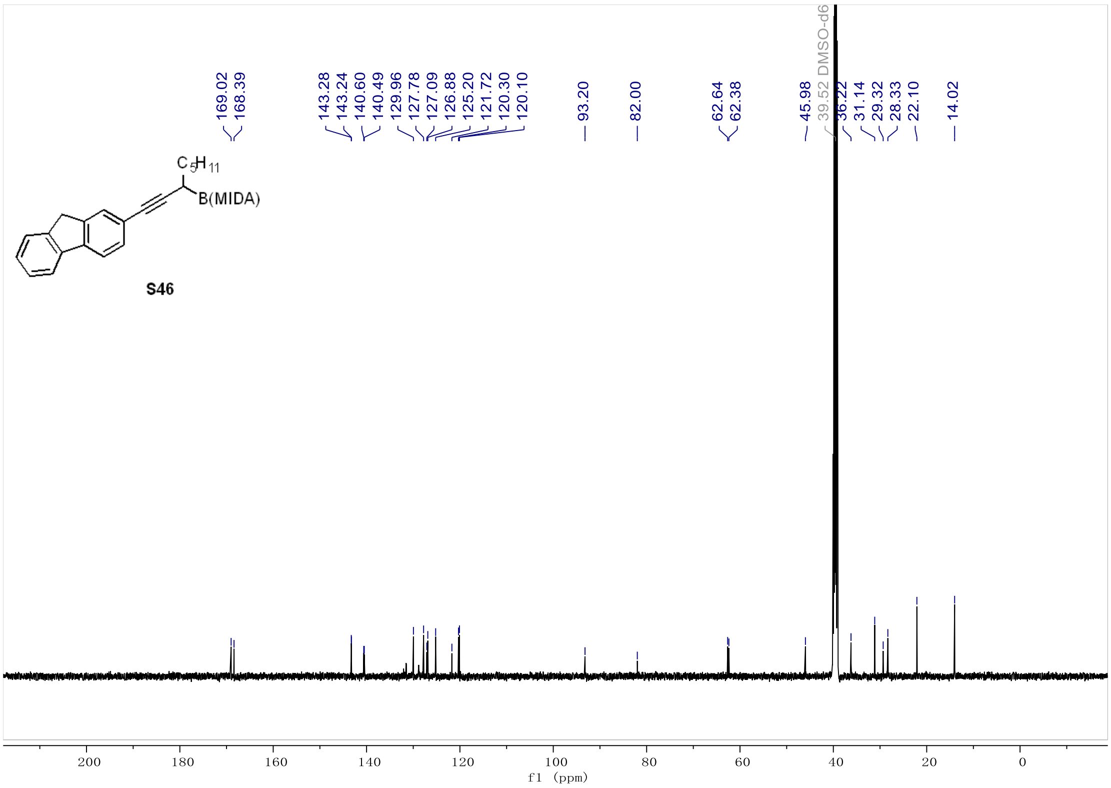

S47: ^{1}H NMR (400 MHz, DMSO-d_6)

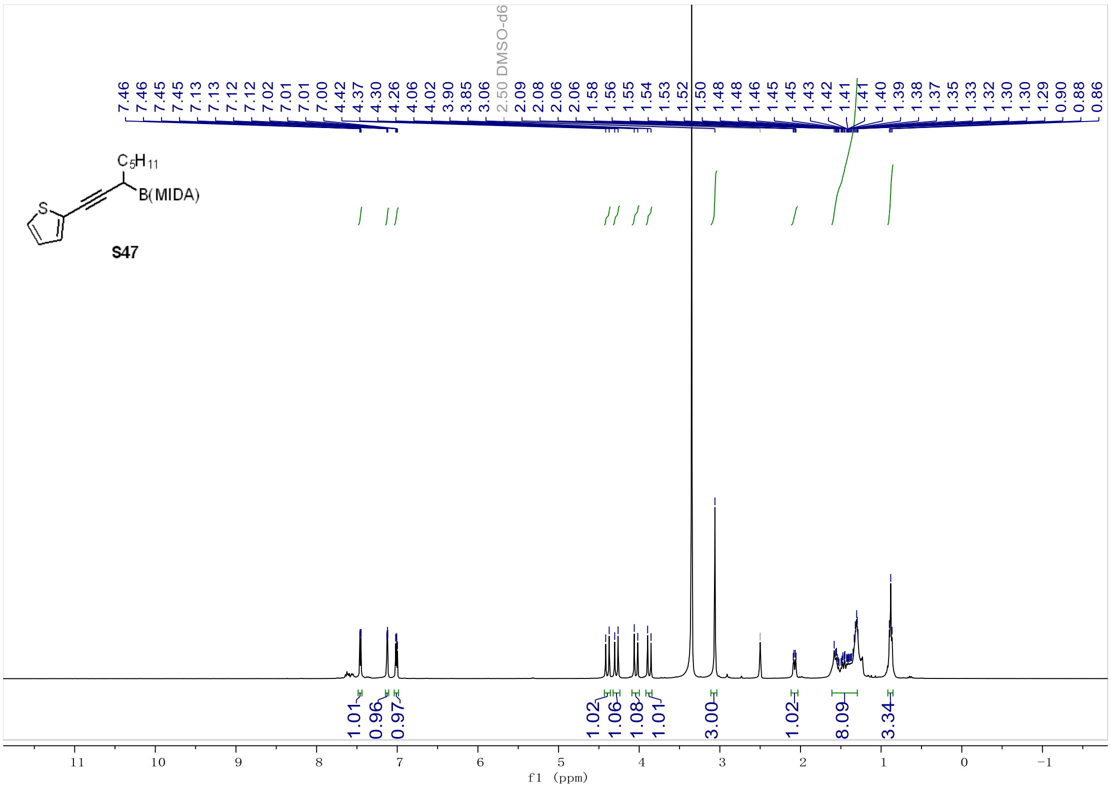

S47: ^{13}C NMR (101 MHz, DMSO-d_6)

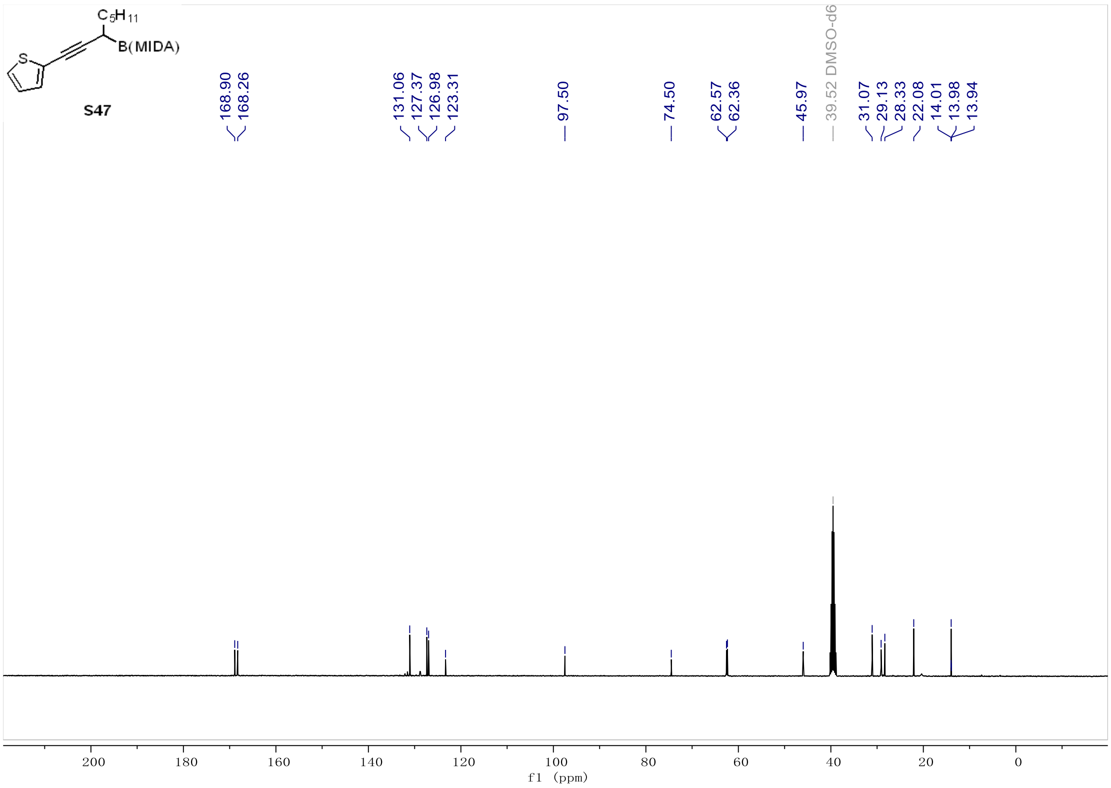

S48: ^{1}H NMR (400 MHz, DMSO-d_6)

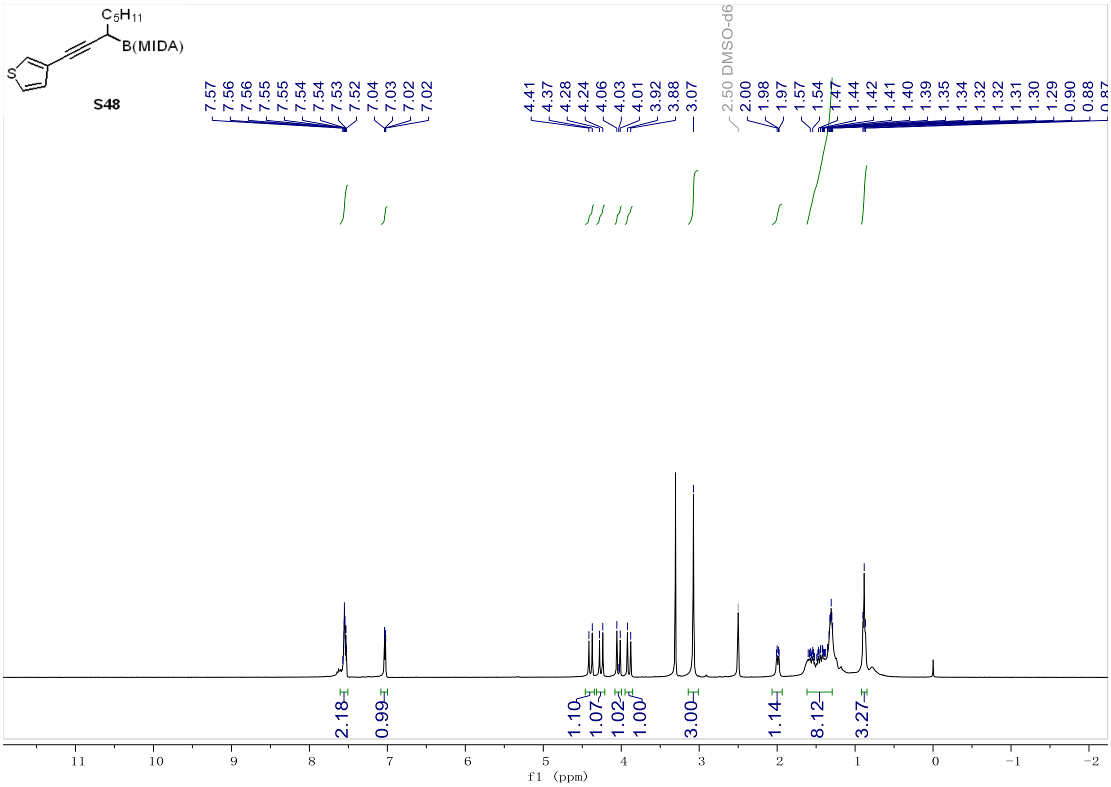

S48: ^{13}C NMR (101 MHz, DMSO-d_6)

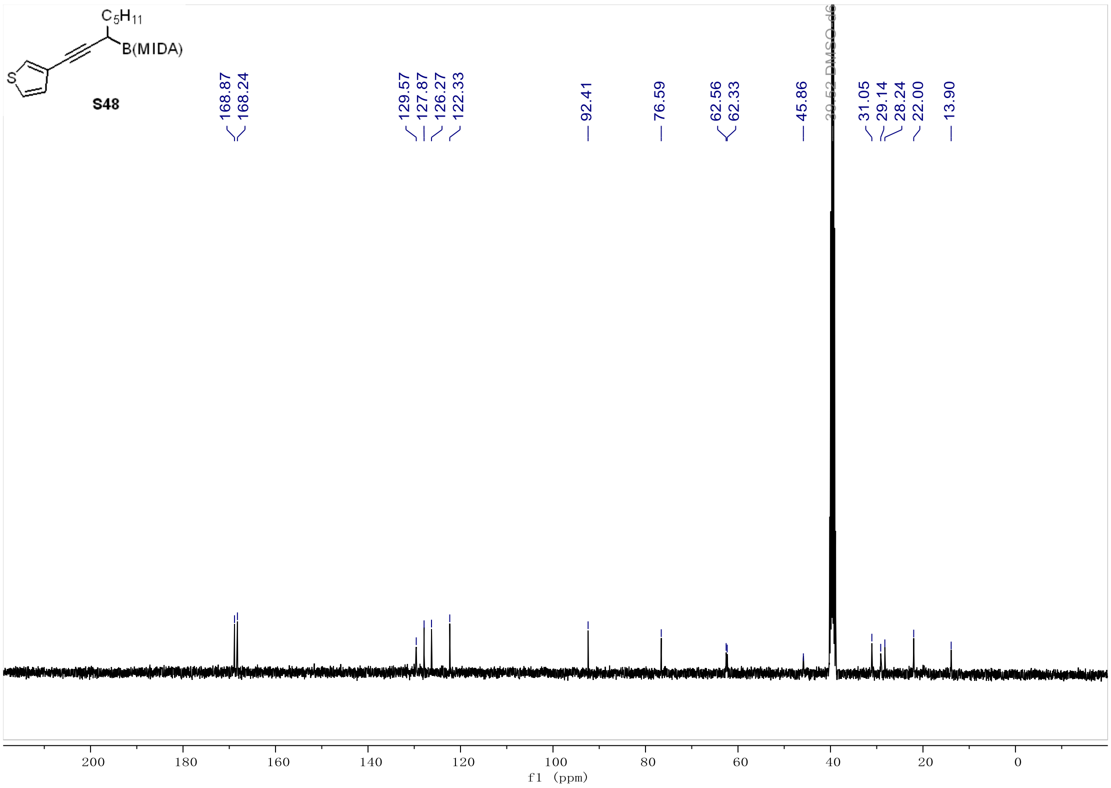

S49: ^{1}H NMR (400 MHz, DMSO-*d₆*)

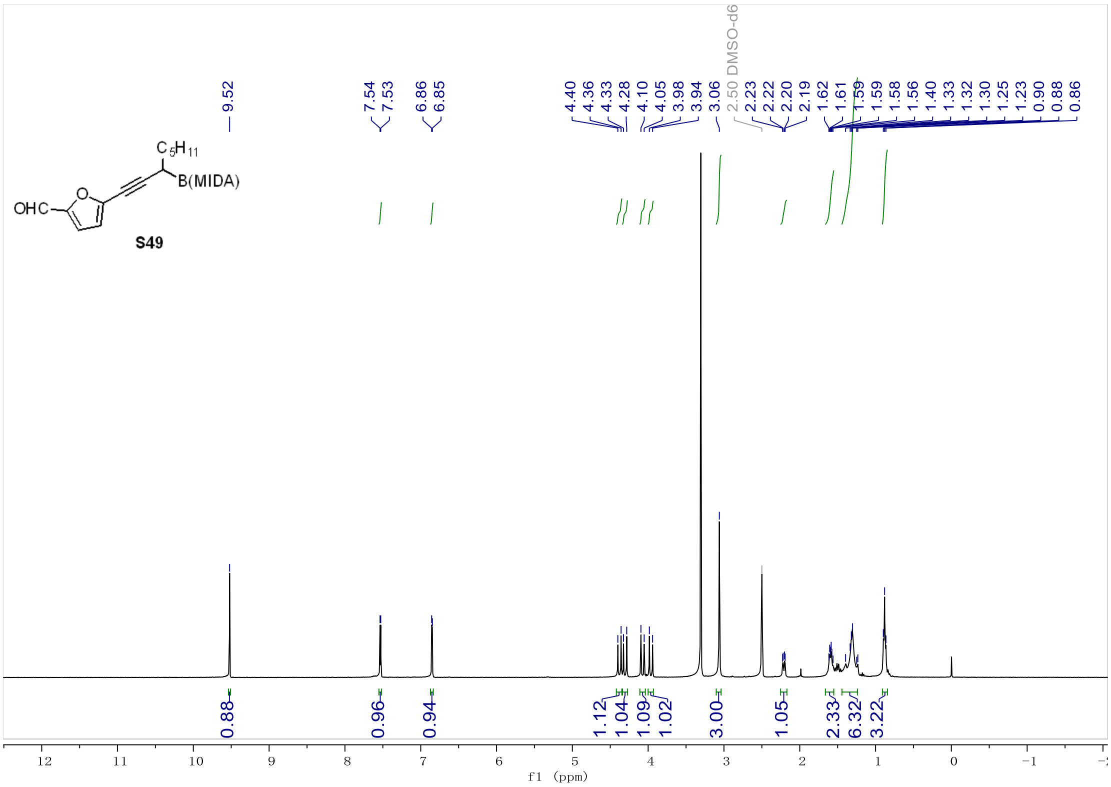

S49: ^{13}C NMR (126 MHz, DMSO-*d₆*)

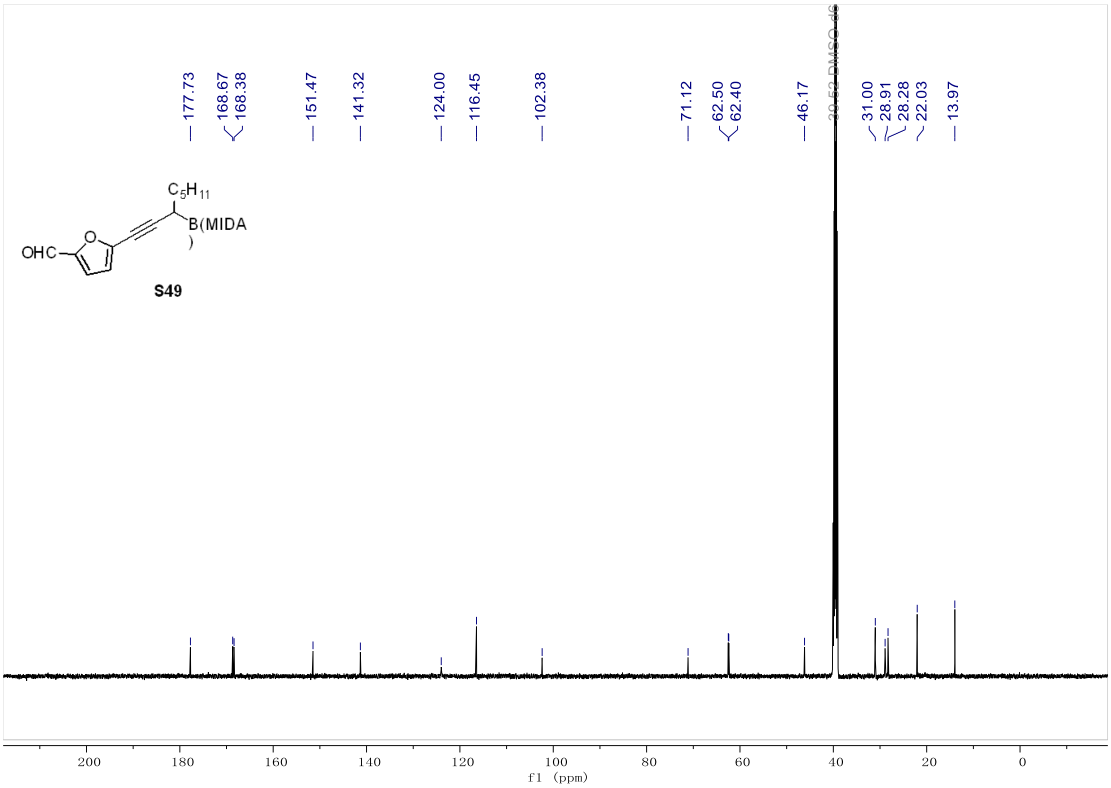

S50: ¹H NMR (500 MHz, Chloroform-*d*)

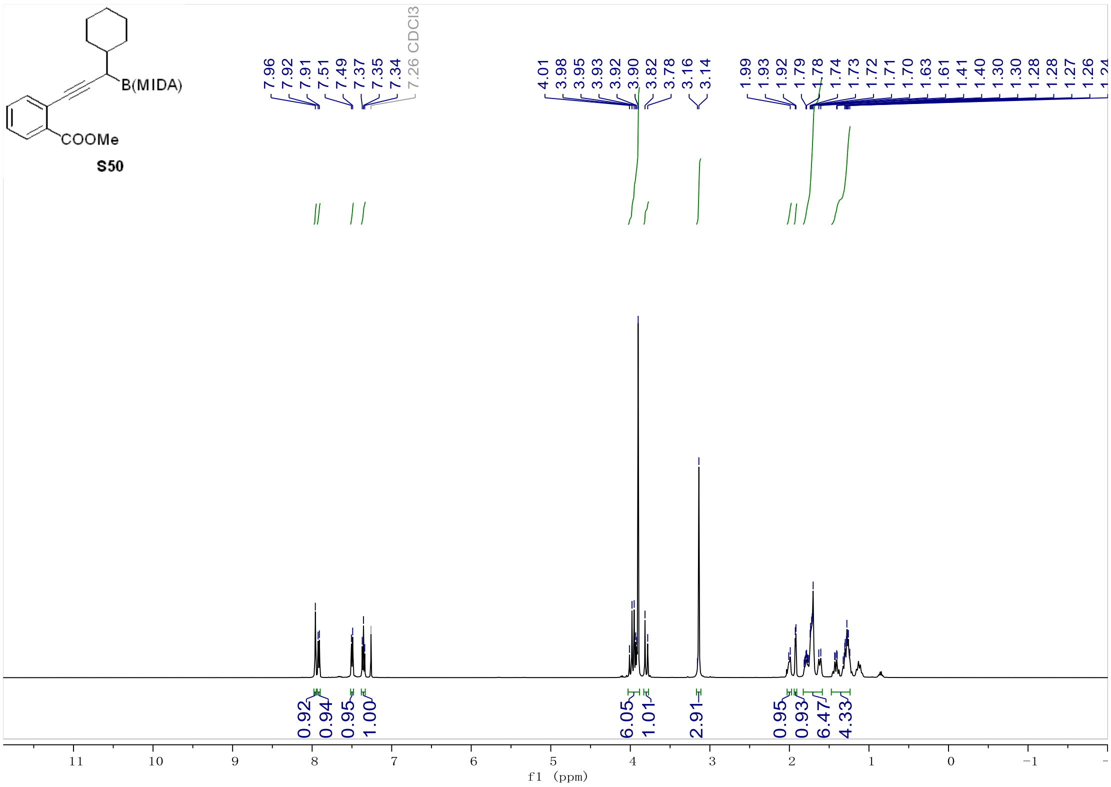

S50: ¹³C NMR (126 MHz, Chloroform-*d*)

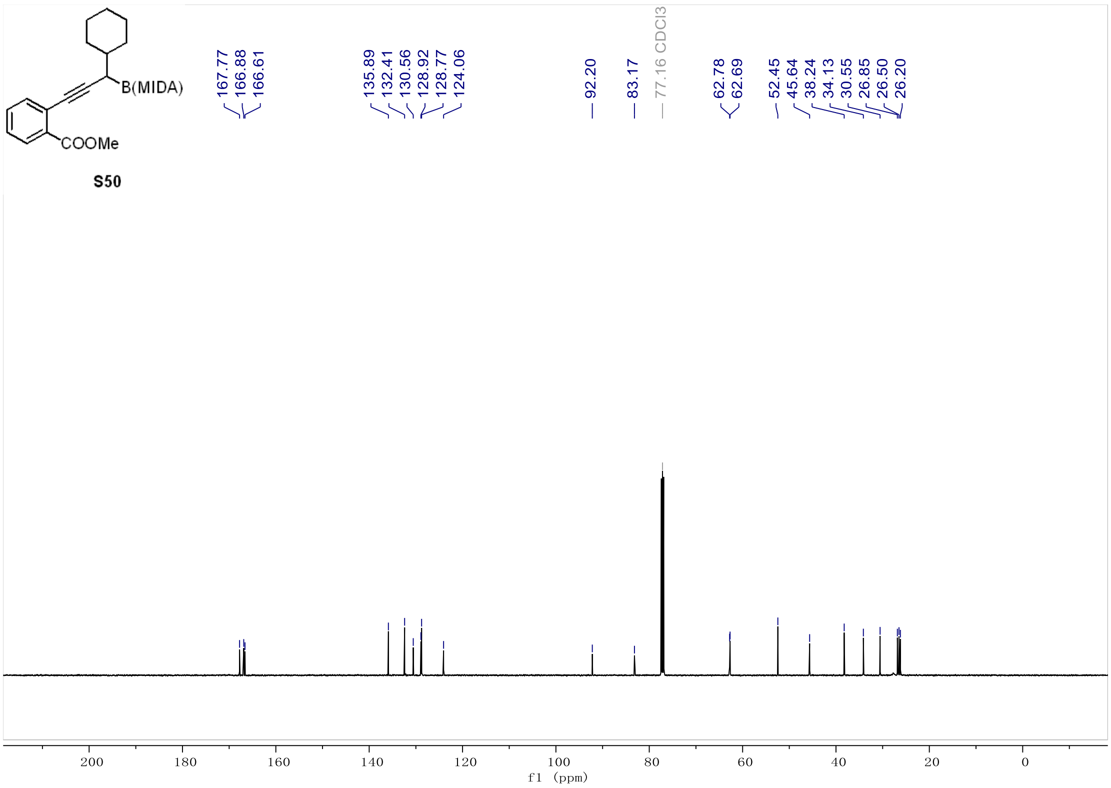

S51: ¹H NMR (500 MHz, Chloroform-*d*)

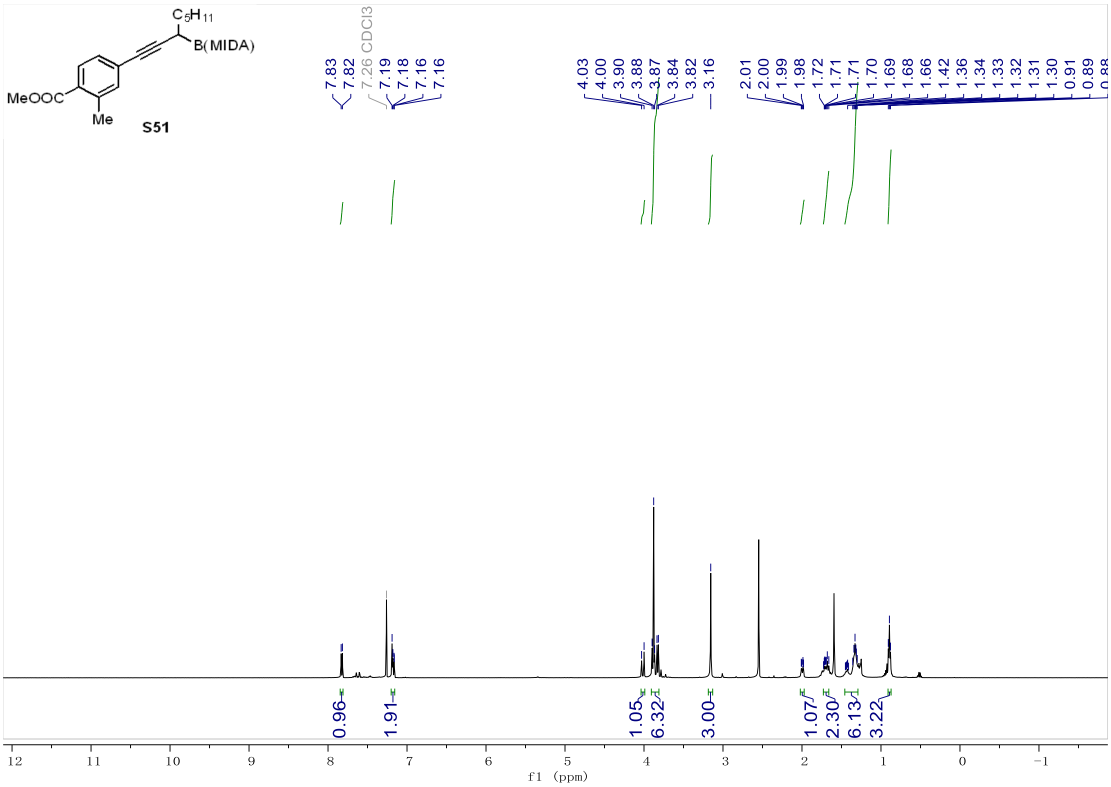

S51: ¹³C NMR (126 MHz, Chloroform-*d*)

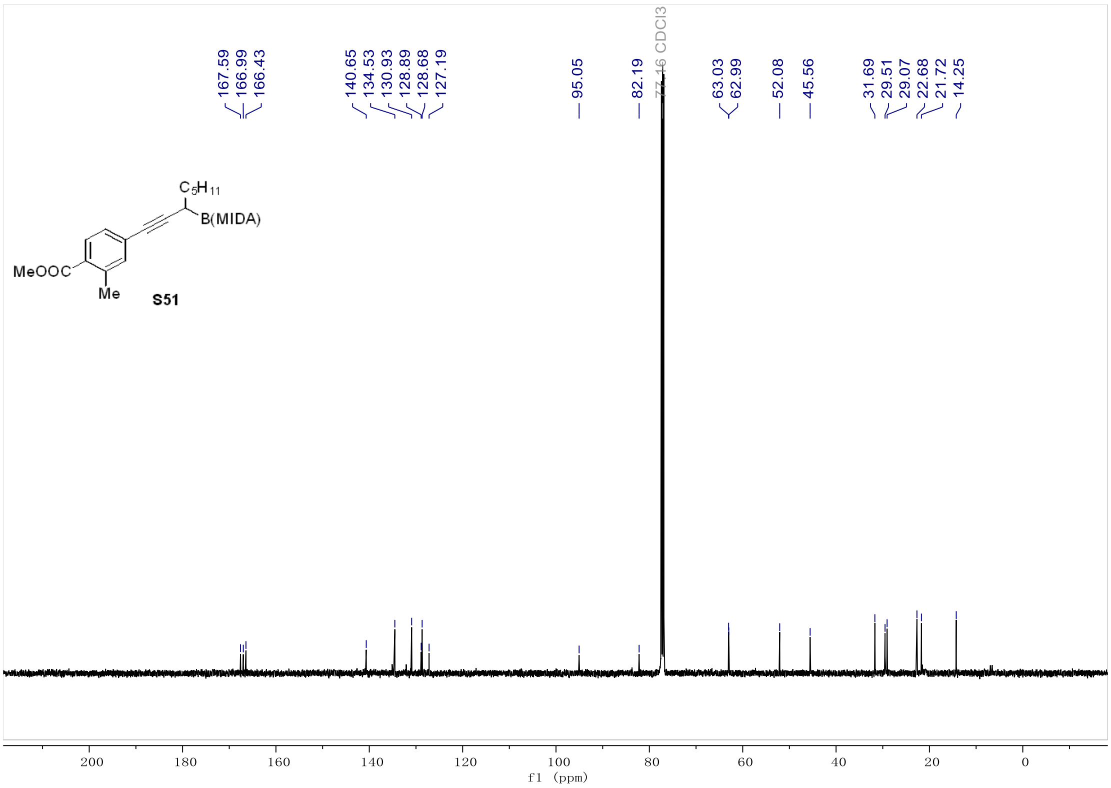

S78: ^{1}H NMR (500 MHz, DMSO-d_6)

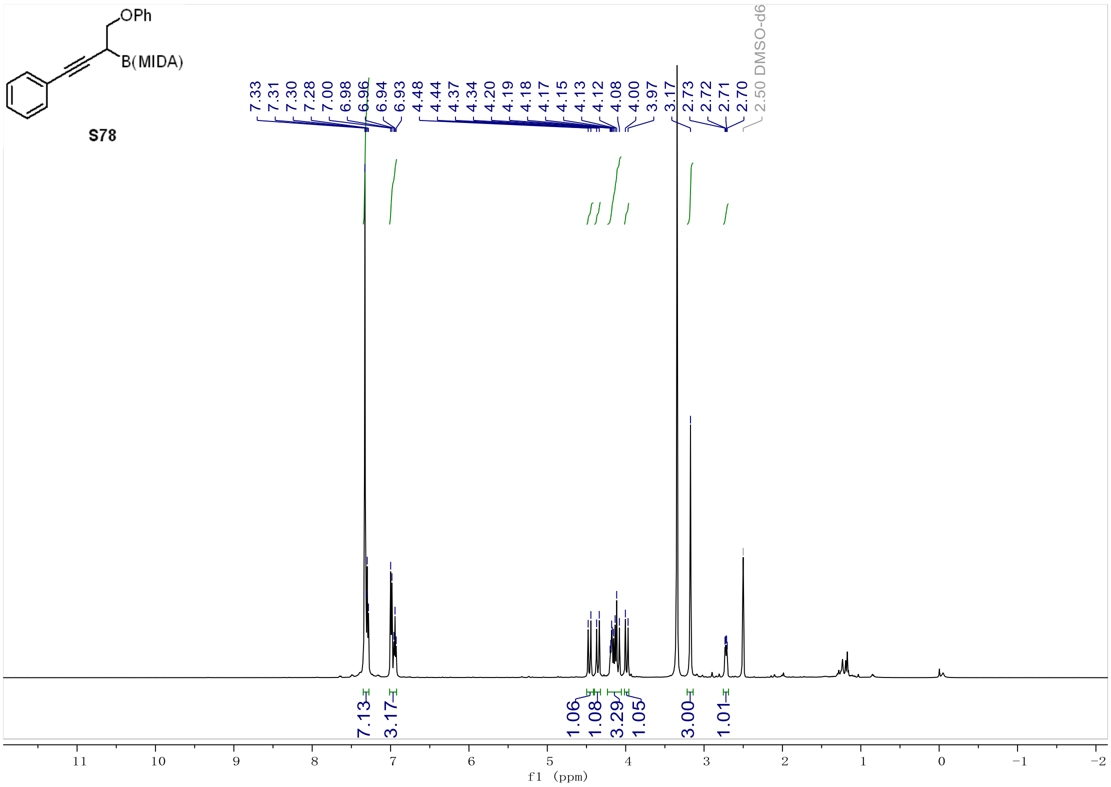

S78: ^{13}C NMR (126 MHz, Chloroform-d)

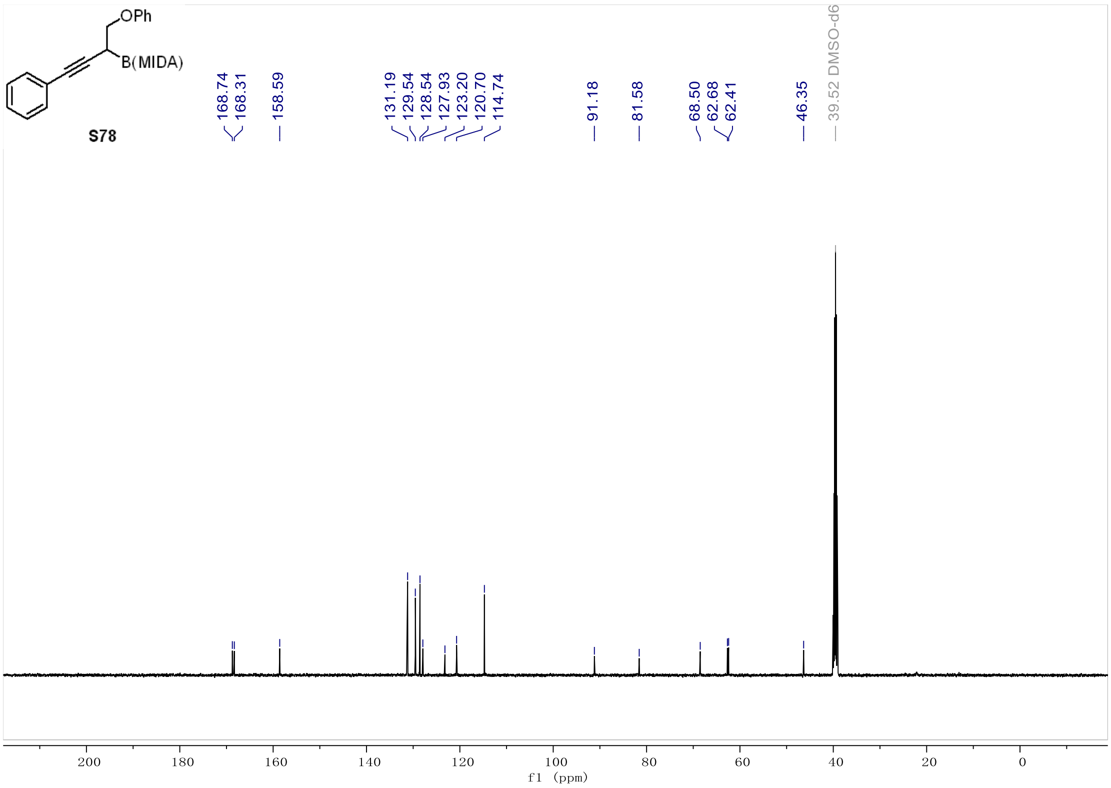

1: ^{1}H NMR (400 MHz, DMSO-d_6)

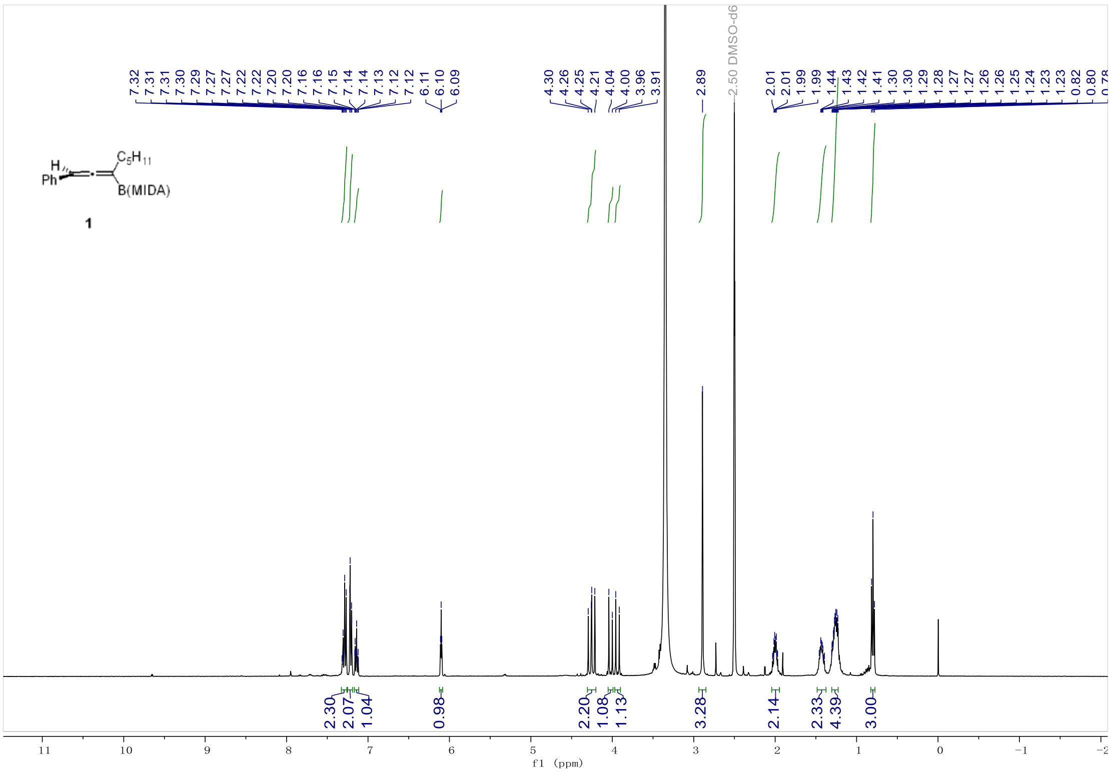

1: ^{13}C NMR (101 MHz, DMSO-d_6)

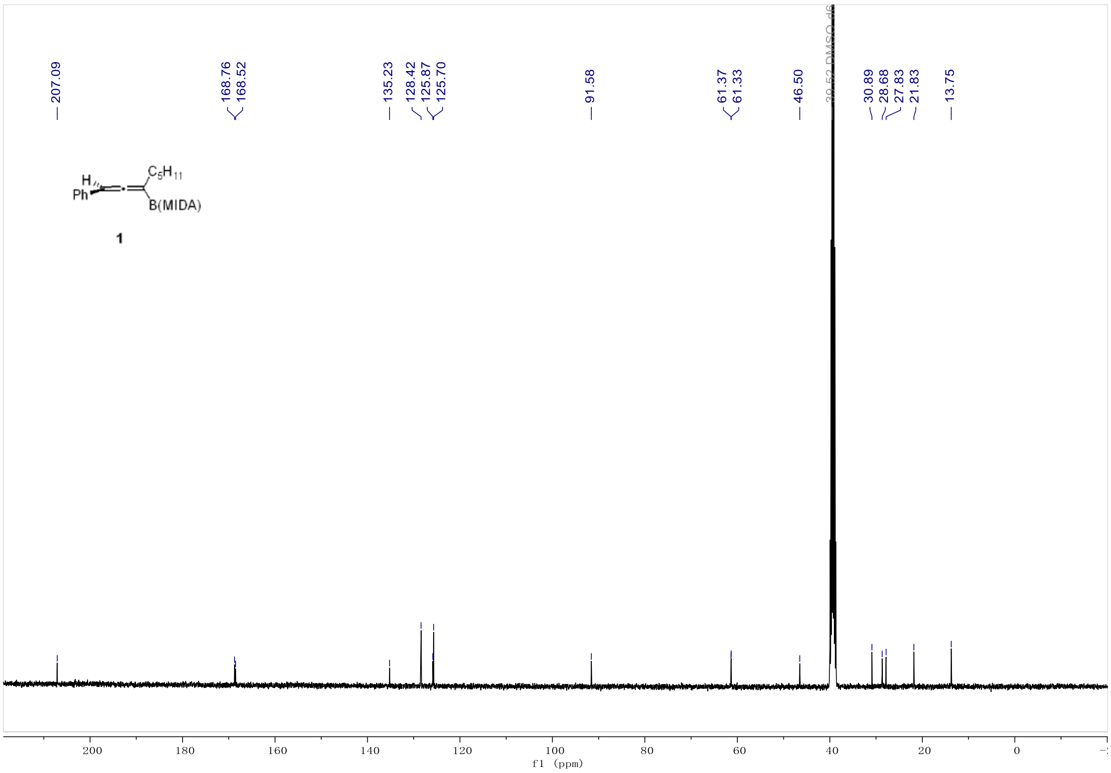

2: ^{1}H NMR (400 MHz, DMSO-d_6)

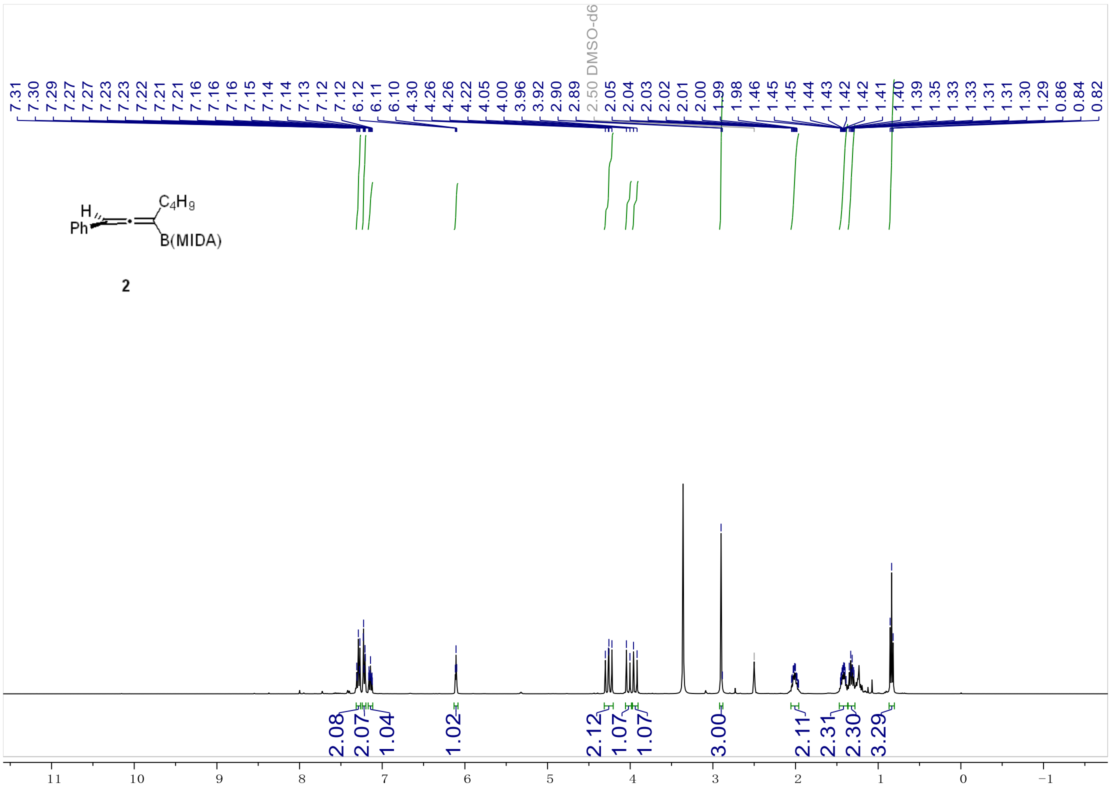

2: ^{13}C NMR (126 MHz, DMSO-d_6)

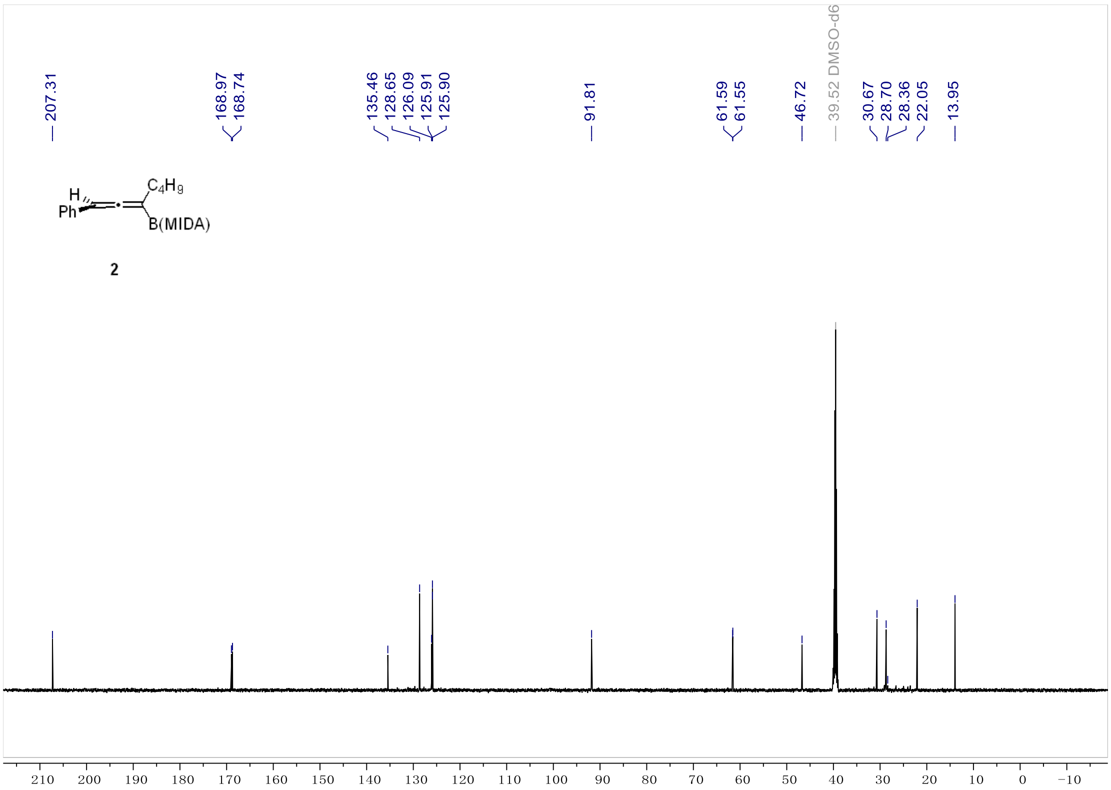

3: ^{1}H NMR (400 MHz, Chloroform-*d*)

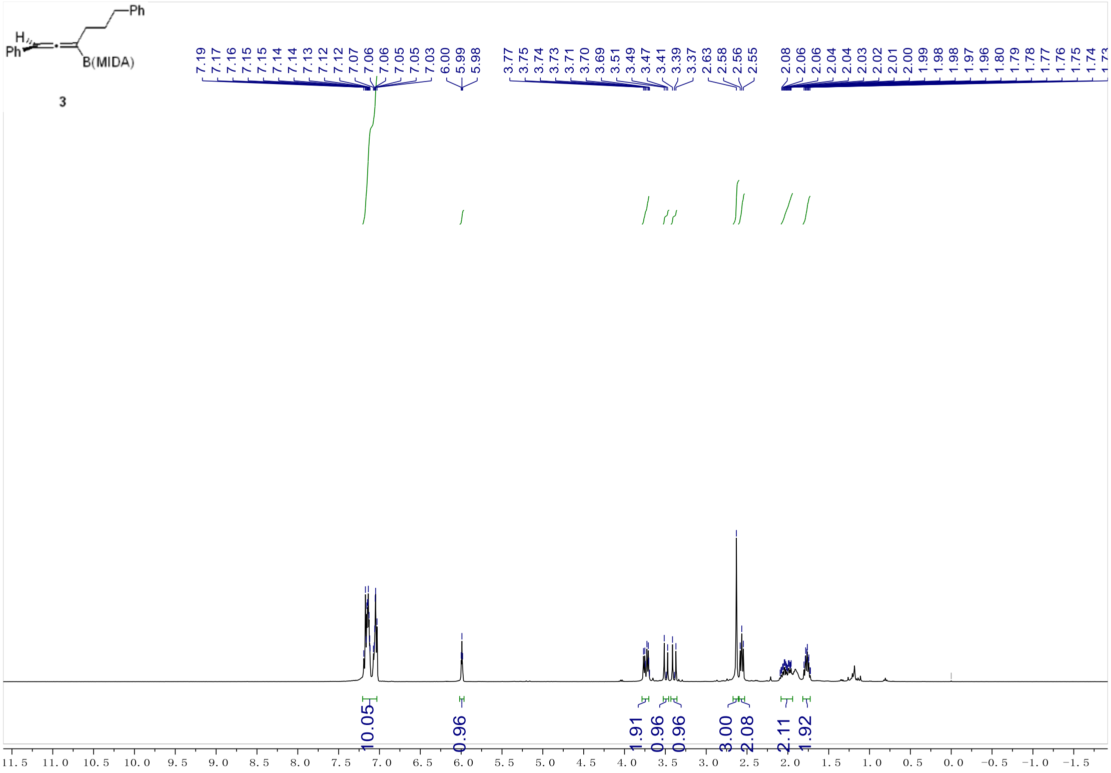

3: ^{13}C NMR (101 MHz, DMSO-*d*$_6$)

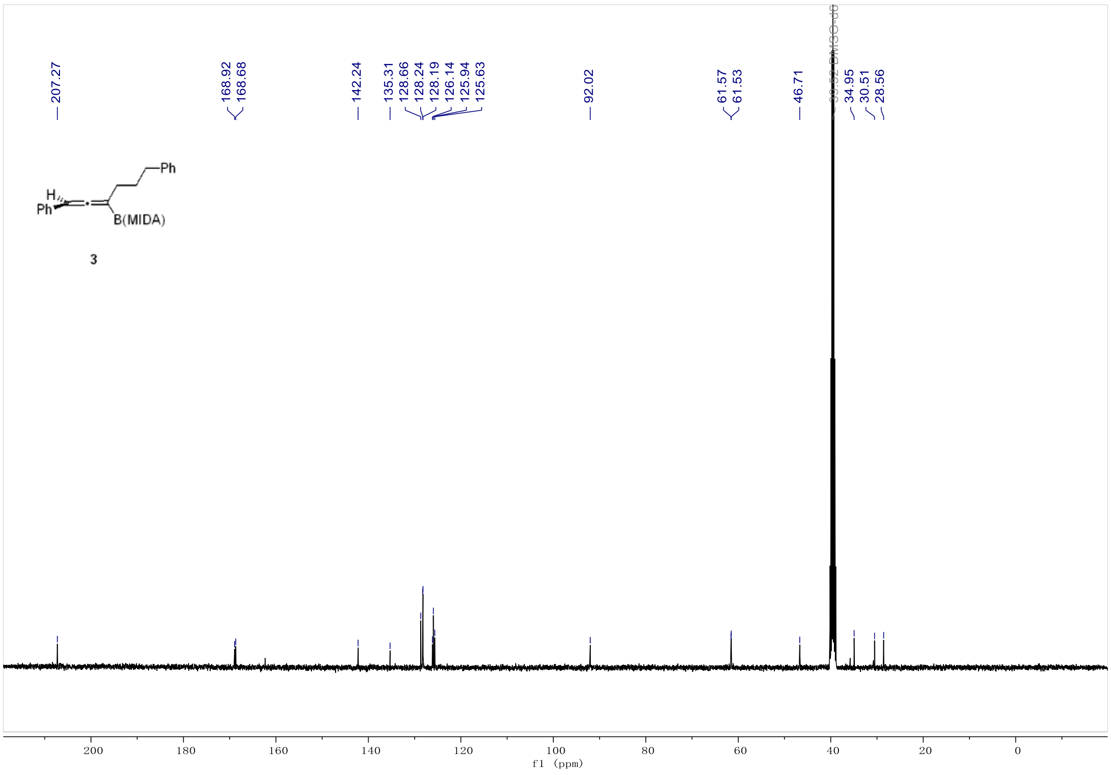

4: ^{1}H NMR (400 MHz, Chloroform-*d*)

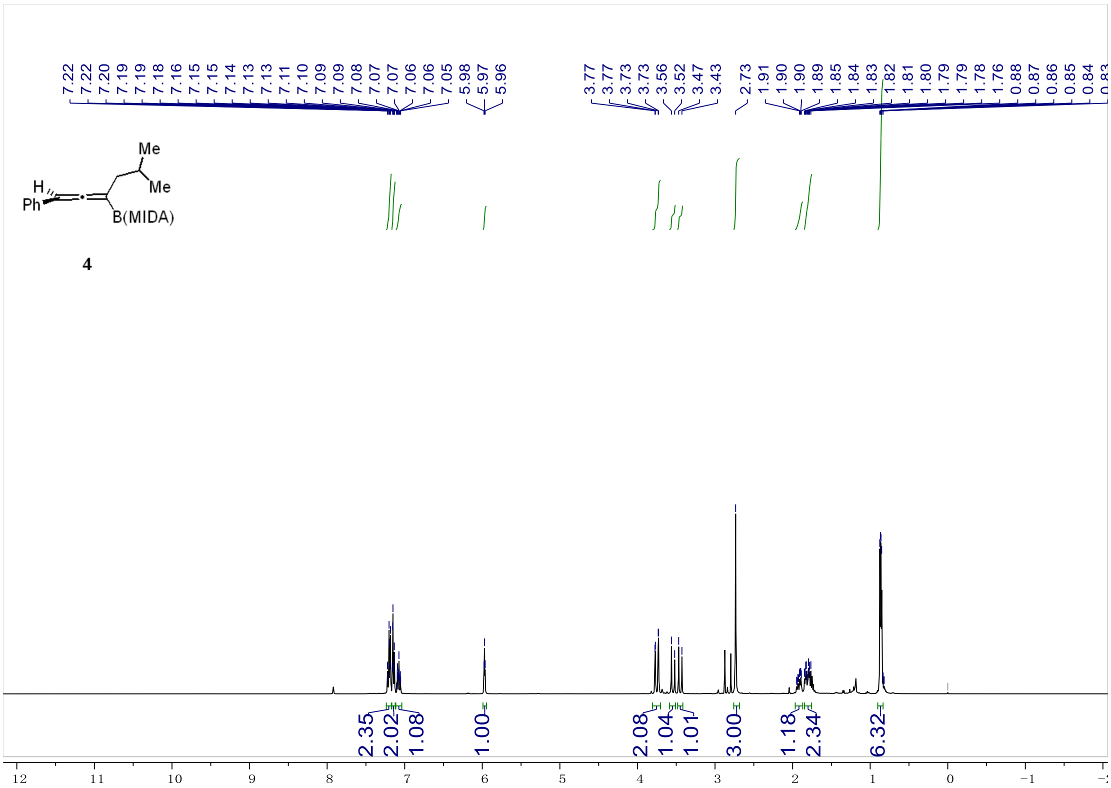

4: ^{13}C NMR (126 MHz, Chloroform-*d*)

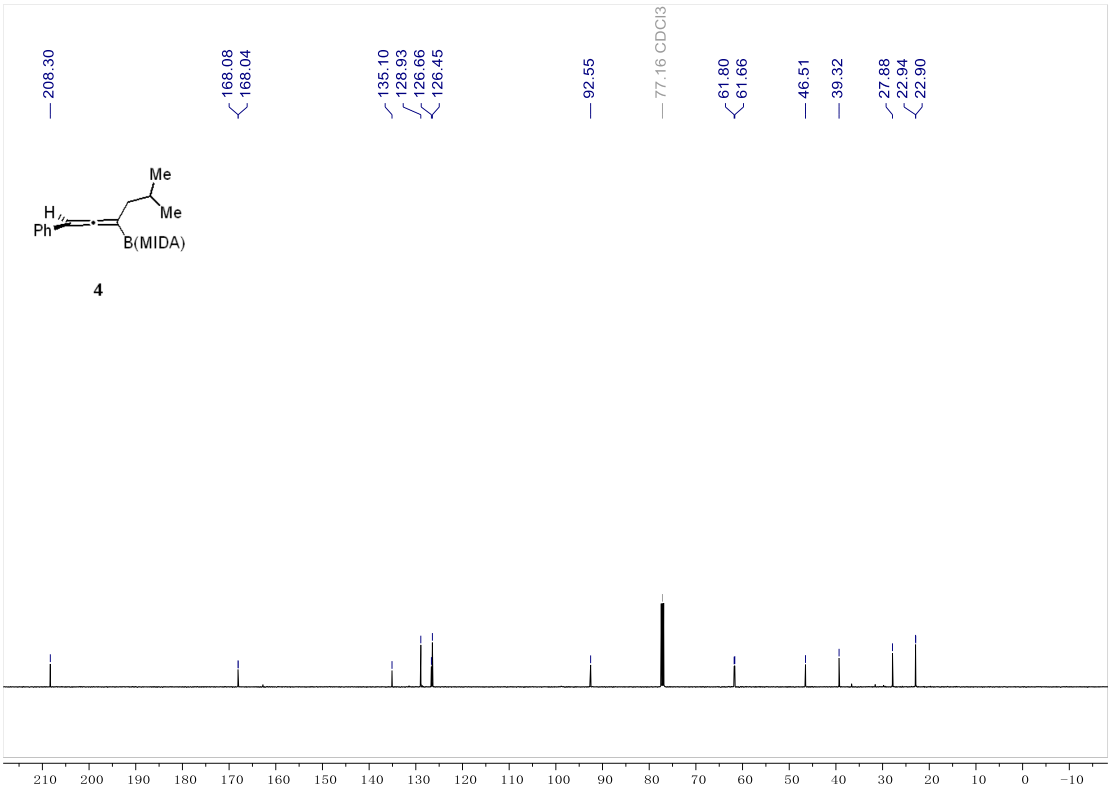

5: ^{1}H NMR (500 MHz, DMSO-d_6)

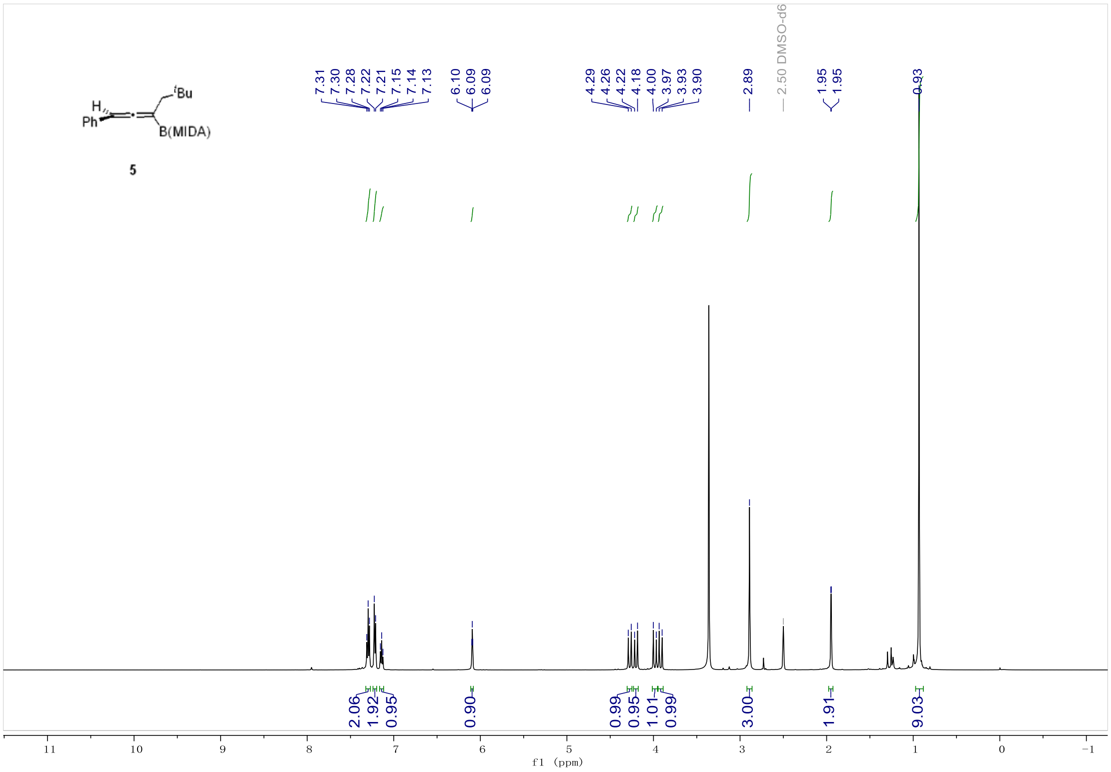

5: ^{13}C NMR (126 MHz, DMSO-d_6)

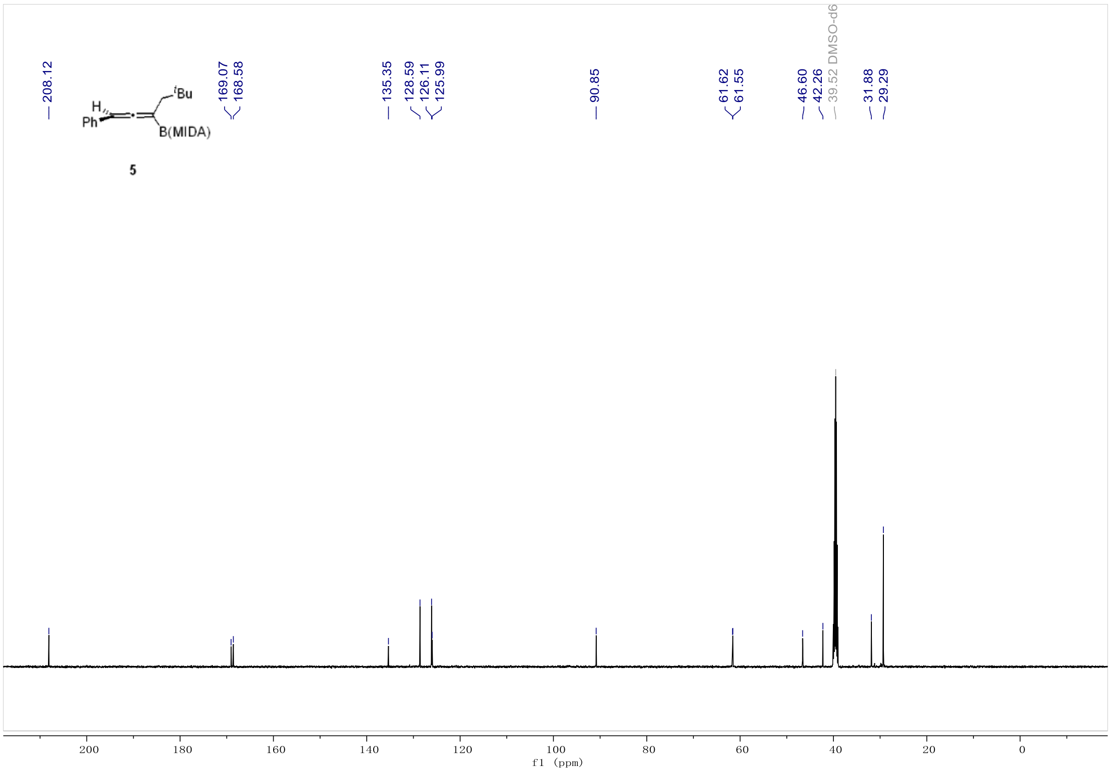

6: ^{1}H NMR (500 MHz, Acetone-d_6)

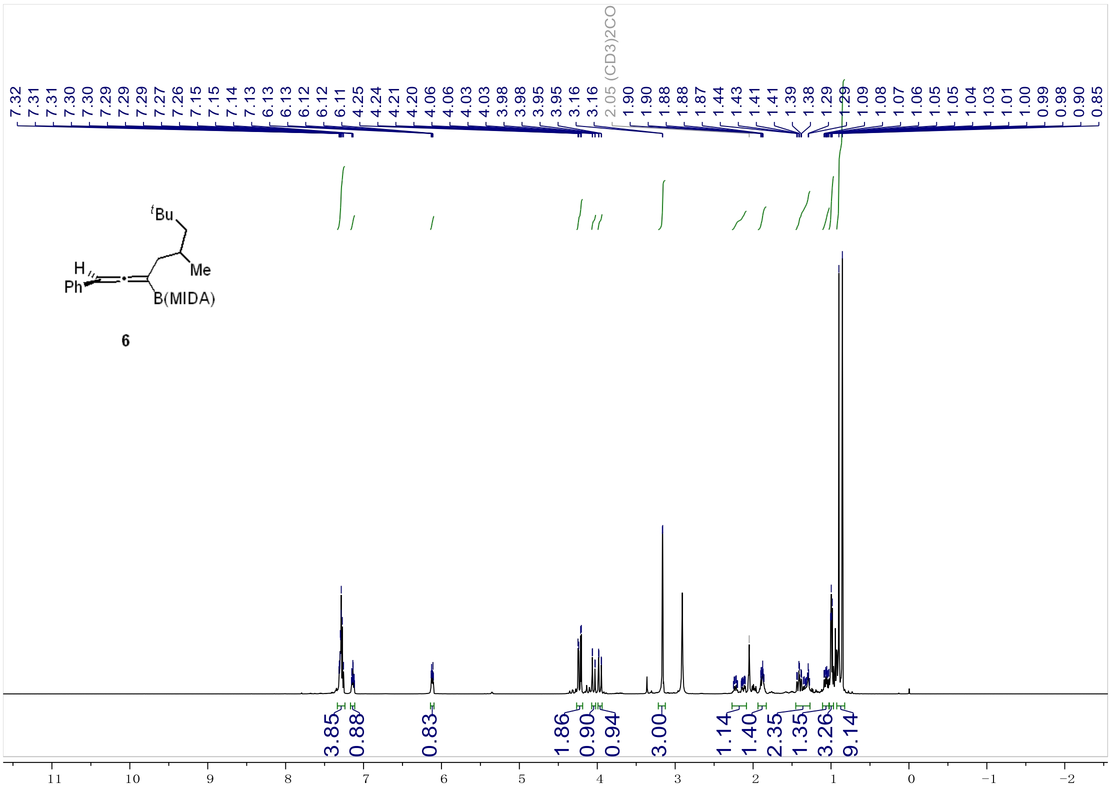

6: ^{13}C NMR (126 MHz, Acetone-d_6)

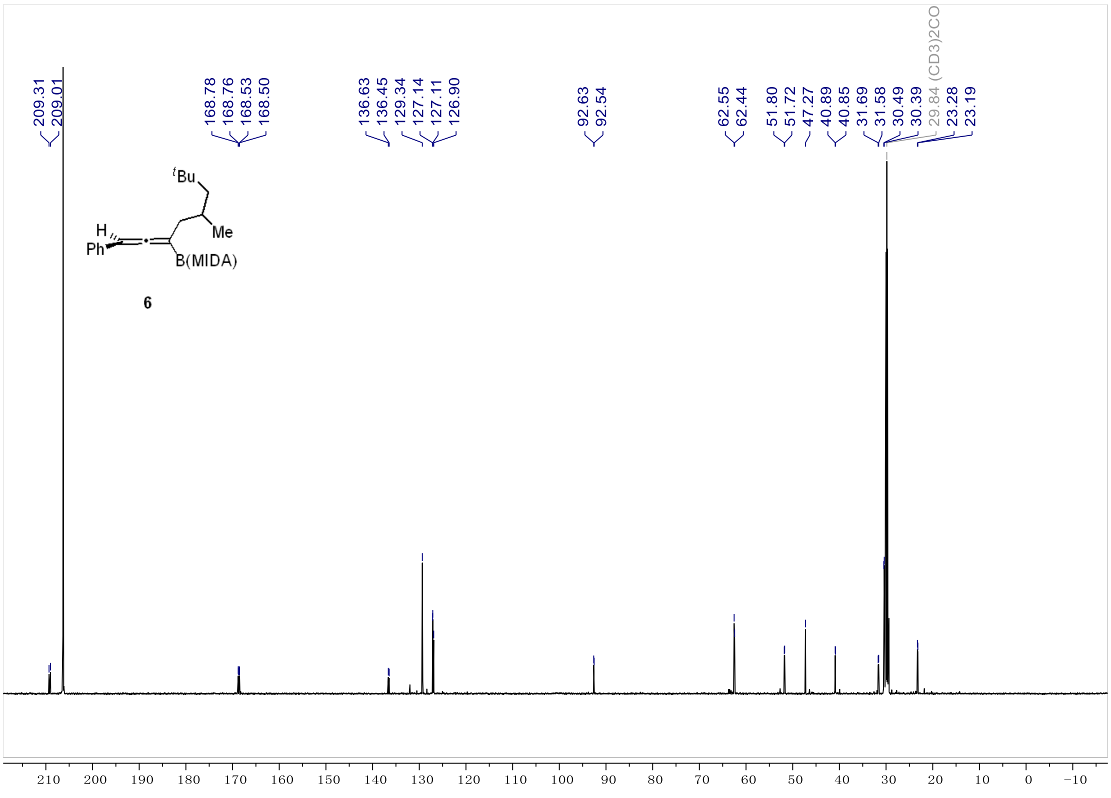

7: ^{1}H NMR (500 MHz, DMSO-d_6)

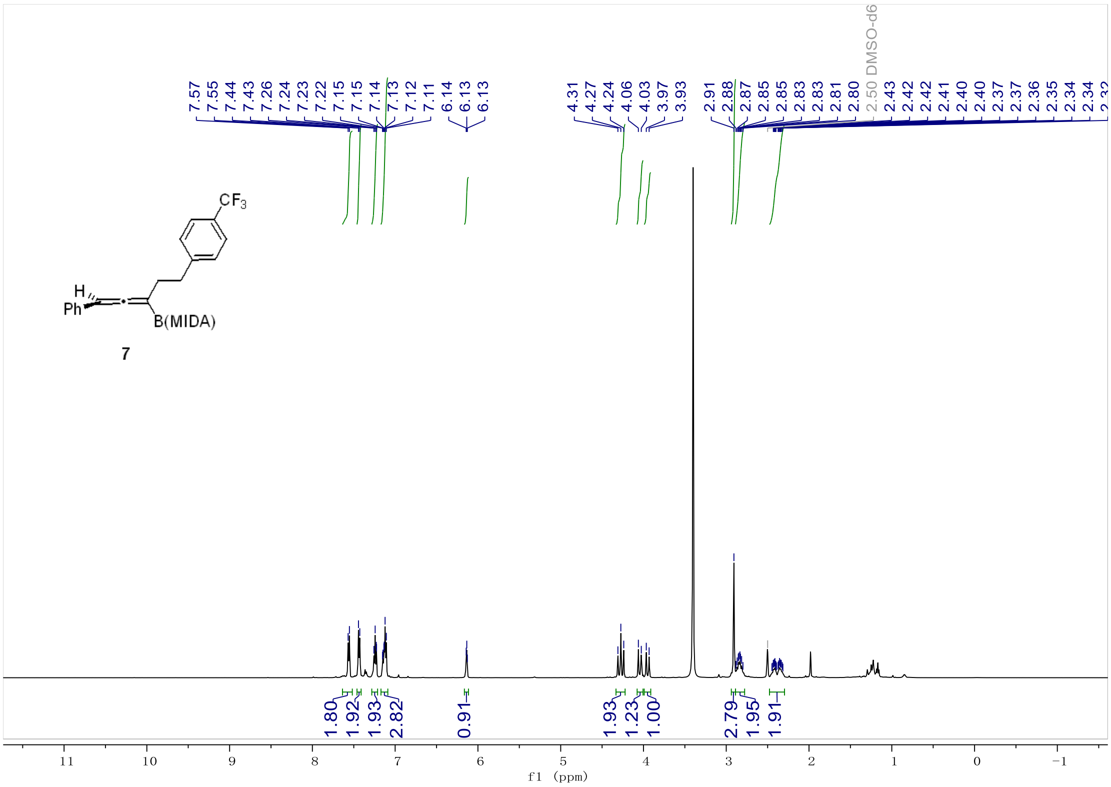

7: ^{13}C NMR (126 MHz, DMSO-d_6)

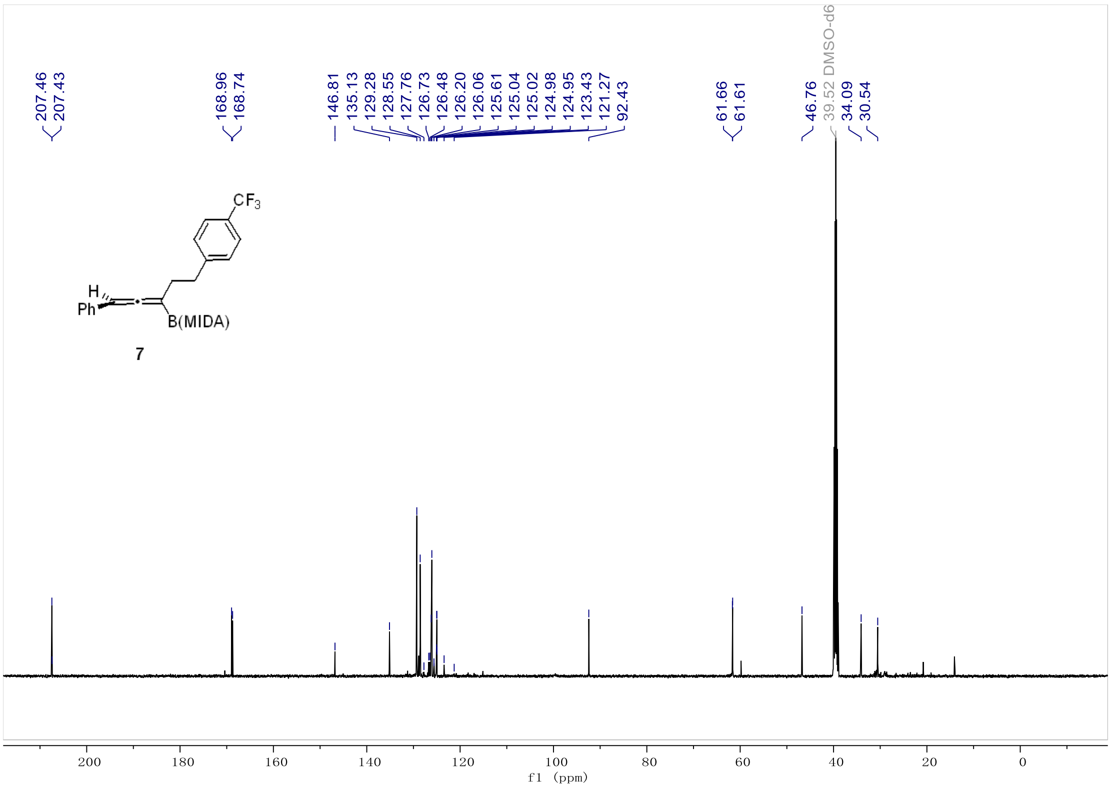

8: ^{1}H NMR (400 MHz, Acetone-d_6)

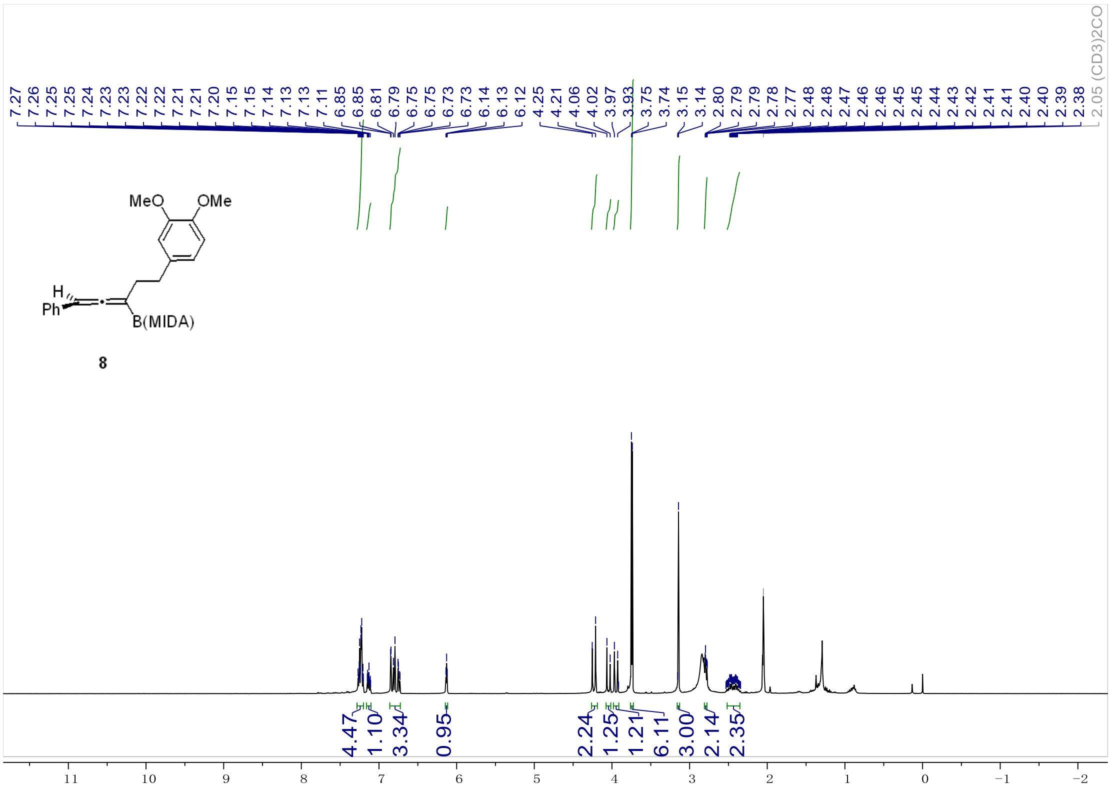

8: ^{13}C NMR (101 MHz, Acetone-d_6)

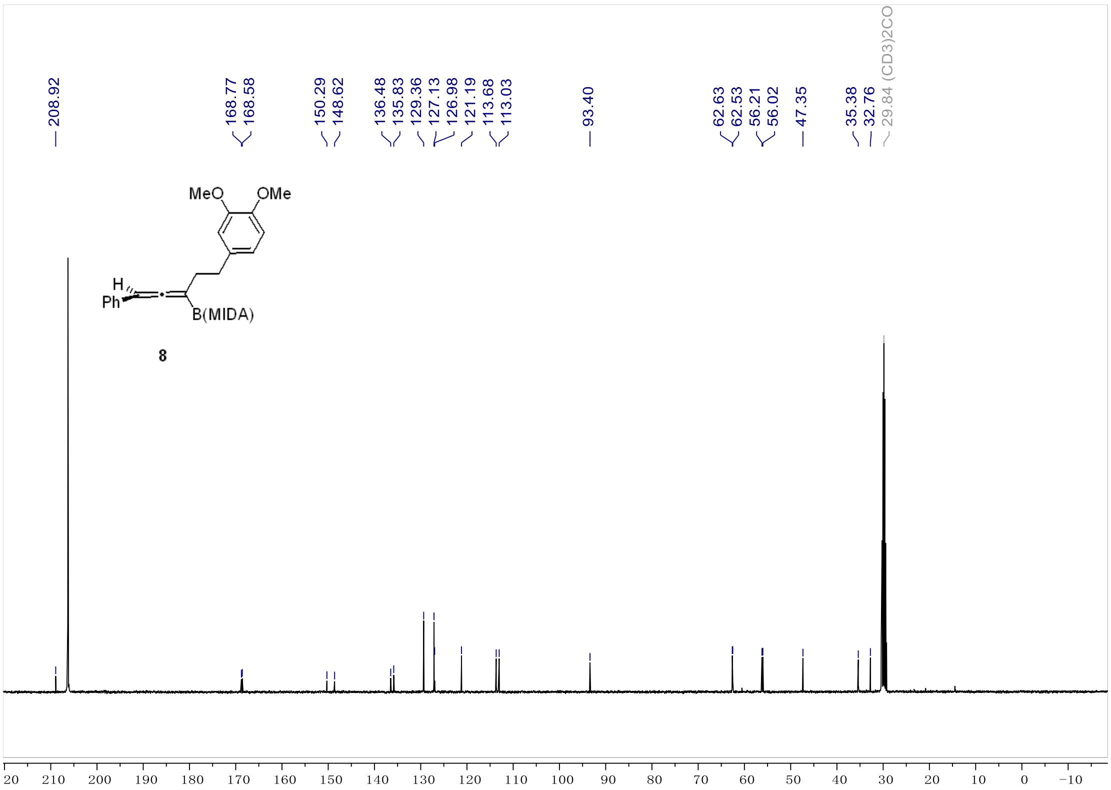

9: ^{1}H NMR (400 MHz, Chloroform-*d*)

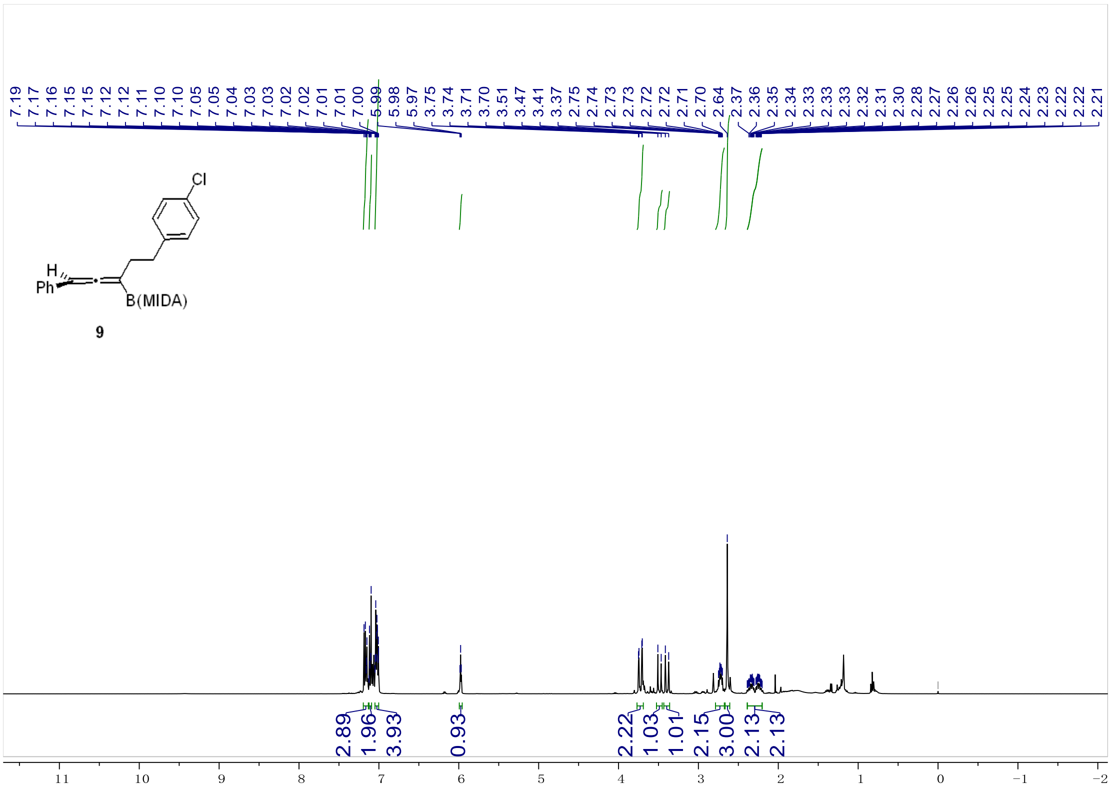

9: ^{13}C NMR (101 MHz, Chloroform-*d*)

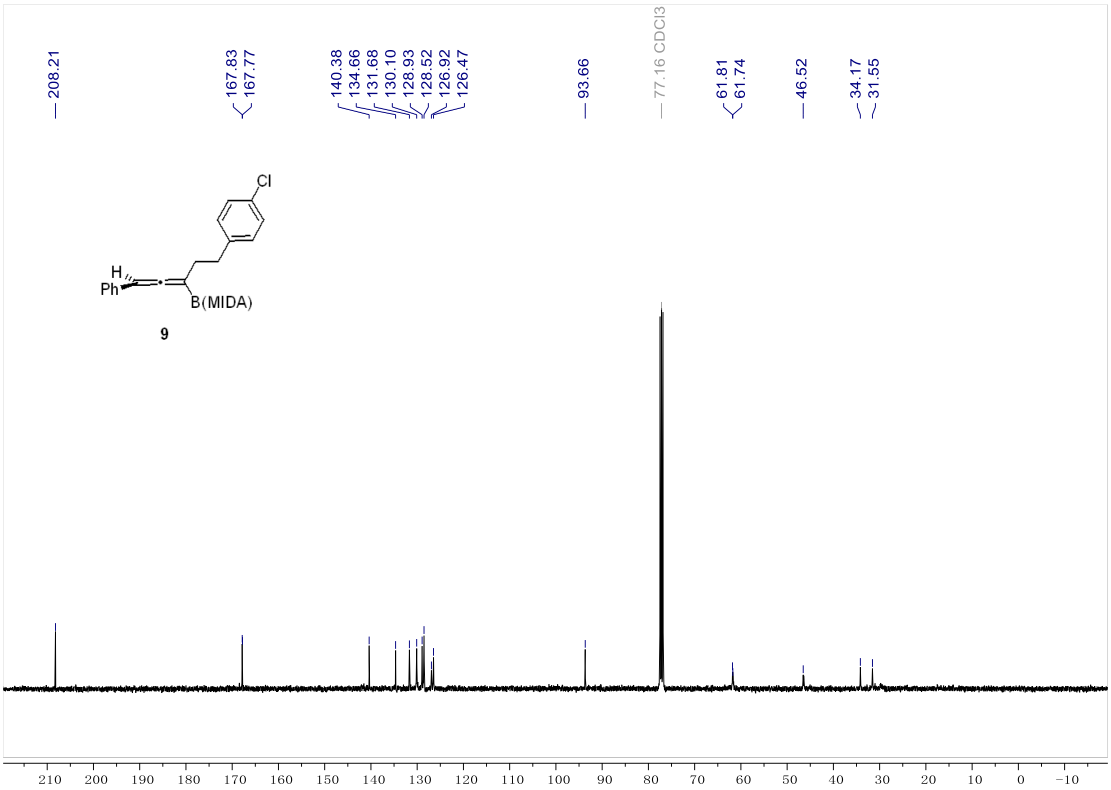

10: ^{1}H NMR (400 MHz, DMSO-d_6)

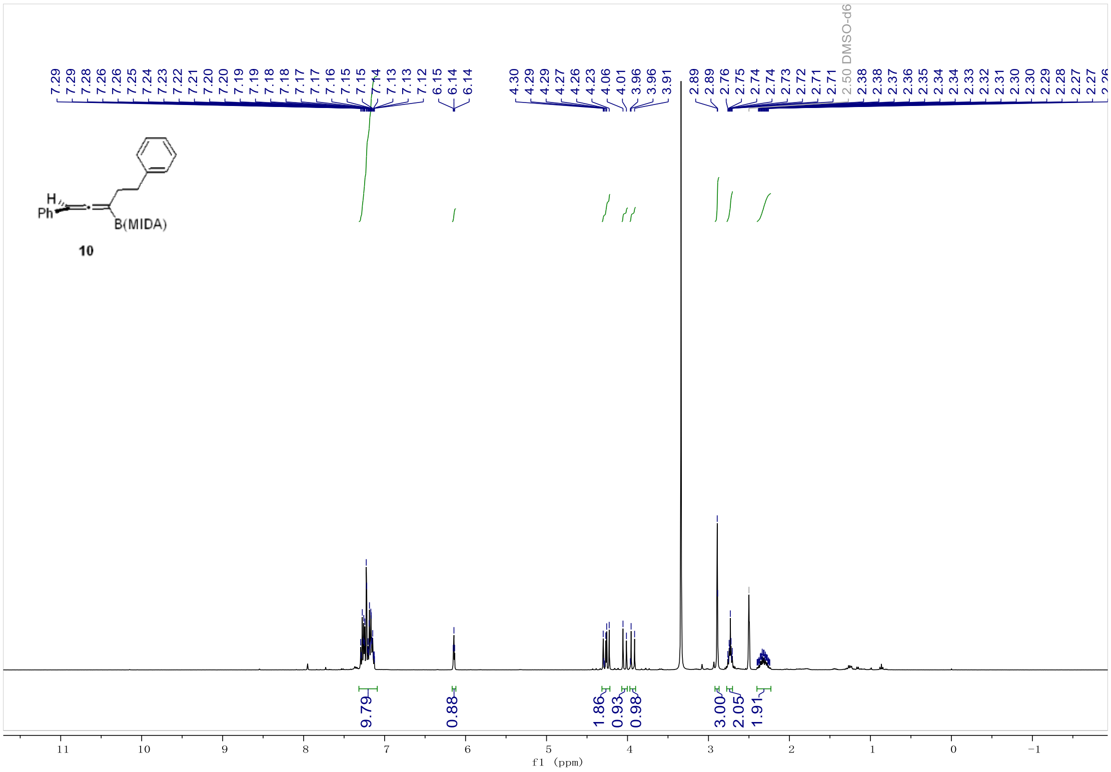

10: ^{13}C NMR (101 MHz, DMSO-d_6)

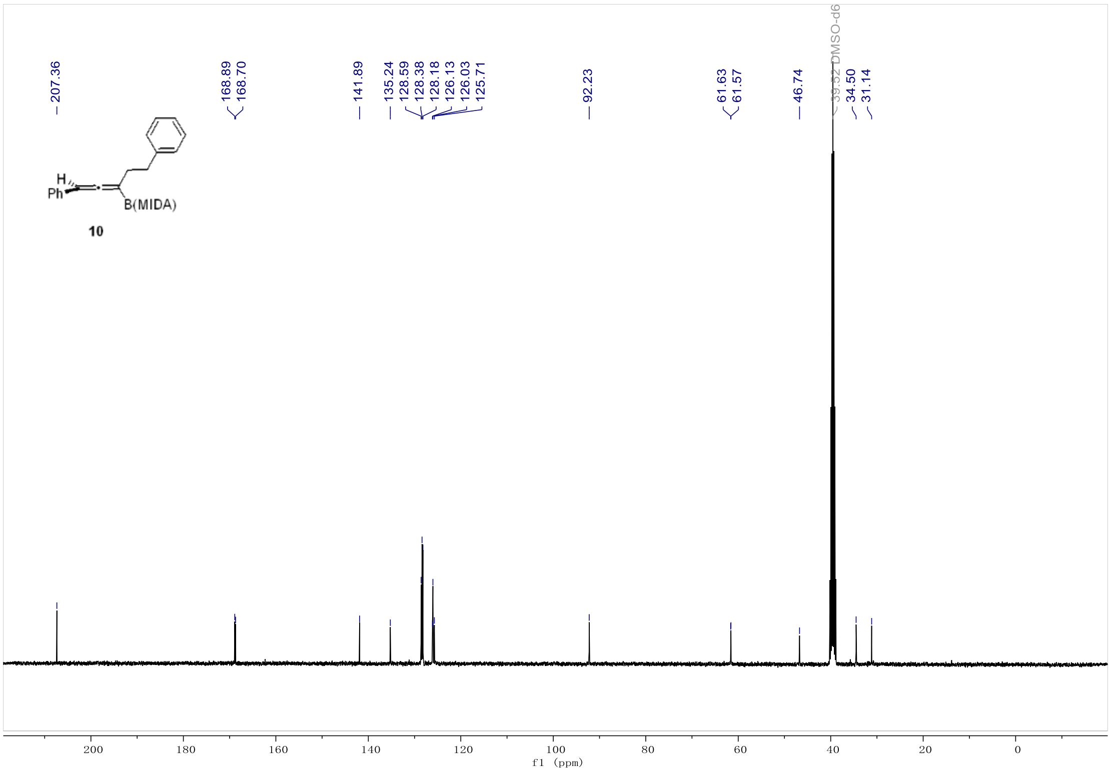

11: ^{1}H NMR (400 MHz, DMSO-d_6)

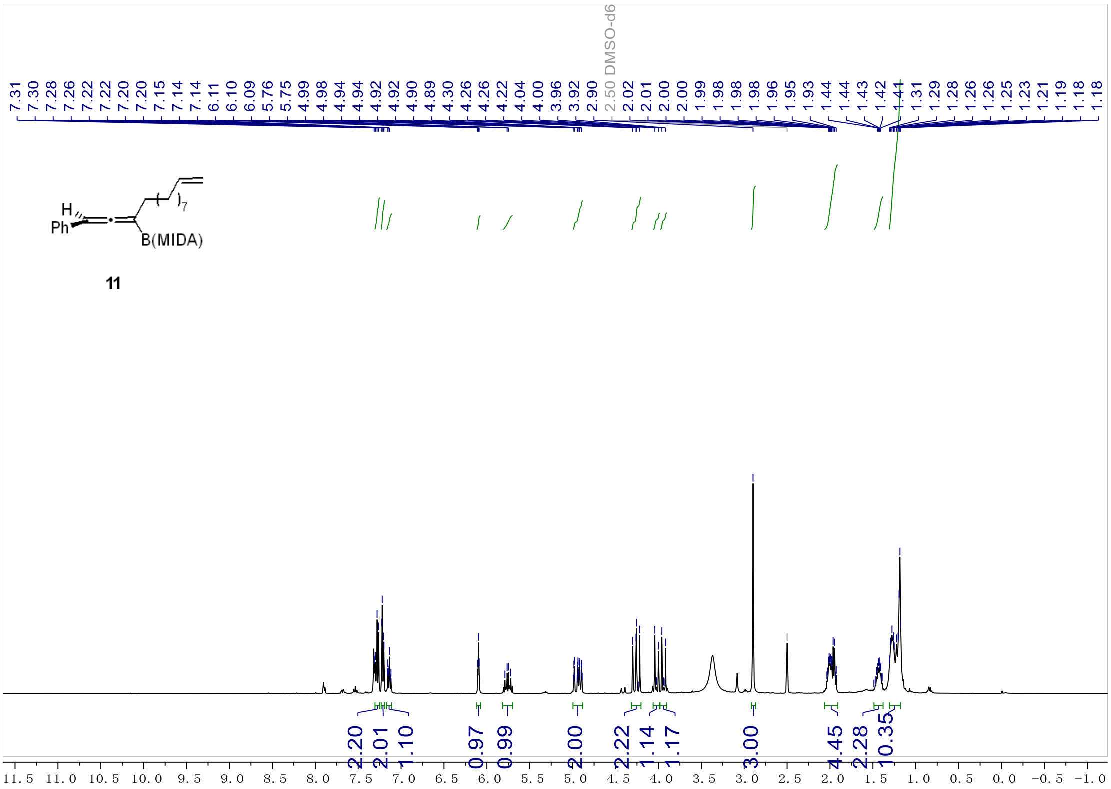

11: ^{13}C NMR (126 MHz, DMSO-d_6)

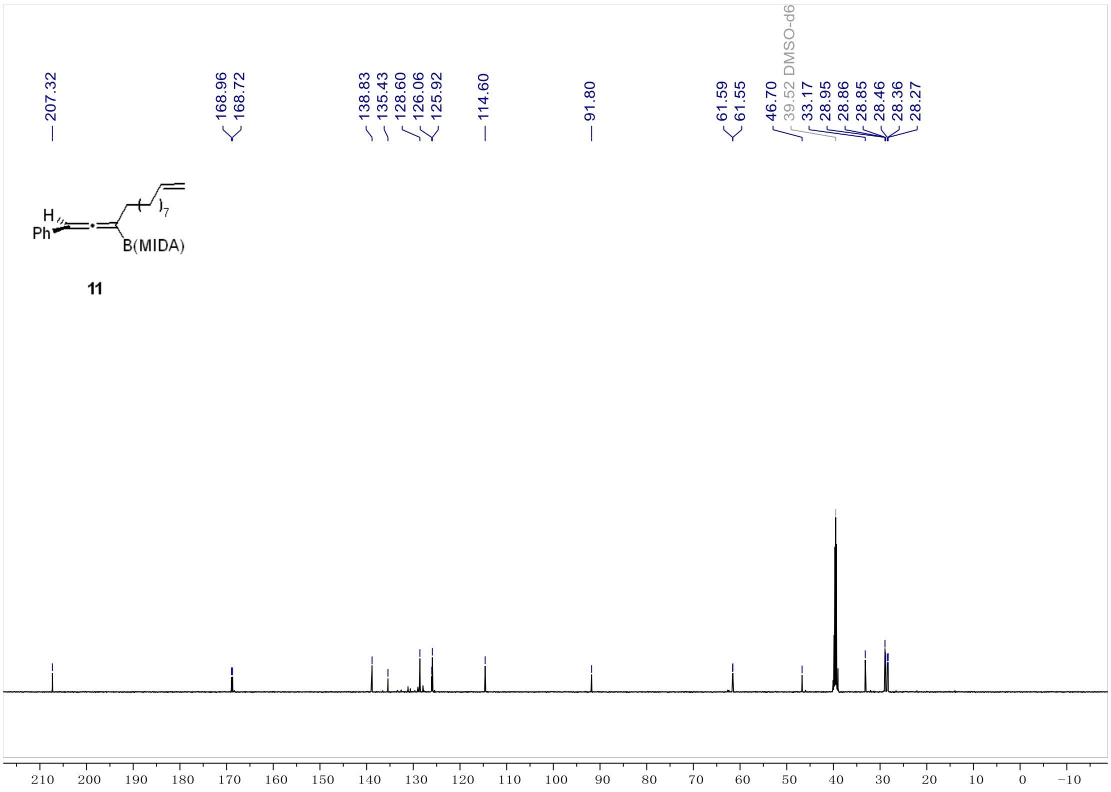

12: ^{1}H NMR (400 MHz, Chloroform-*d*)

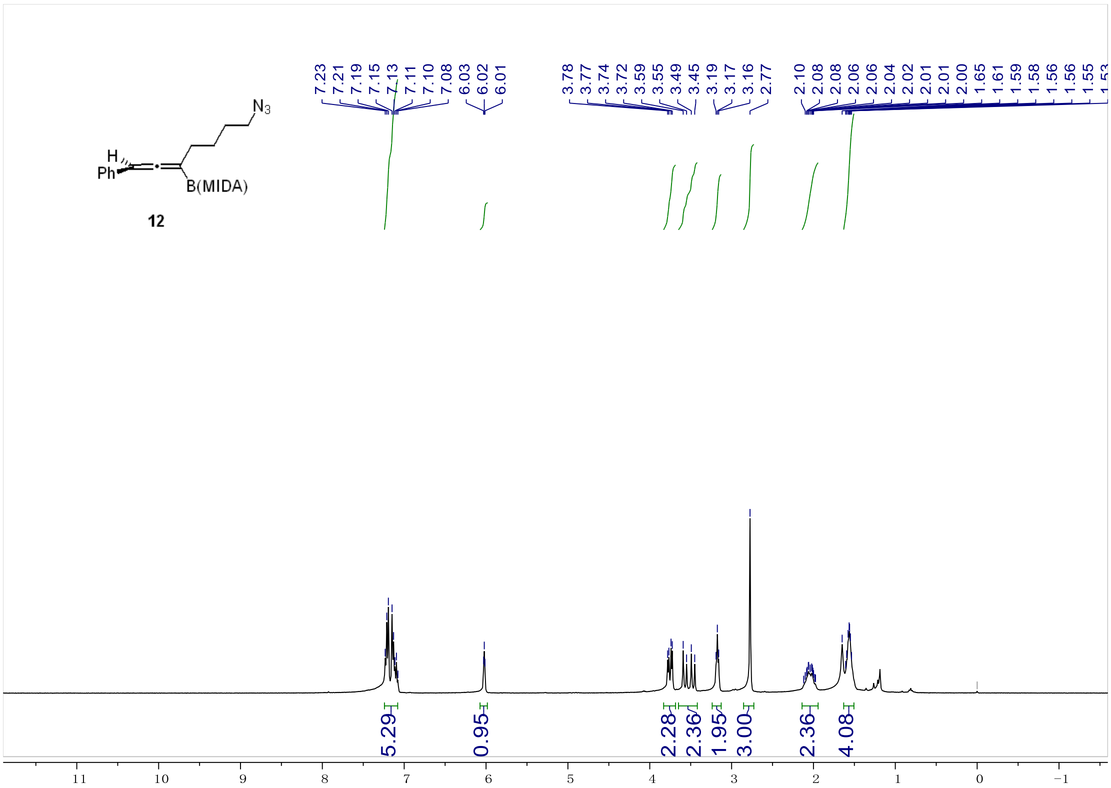

12: ^{13}C NMR (126 MHz, Chloroform-*d*)

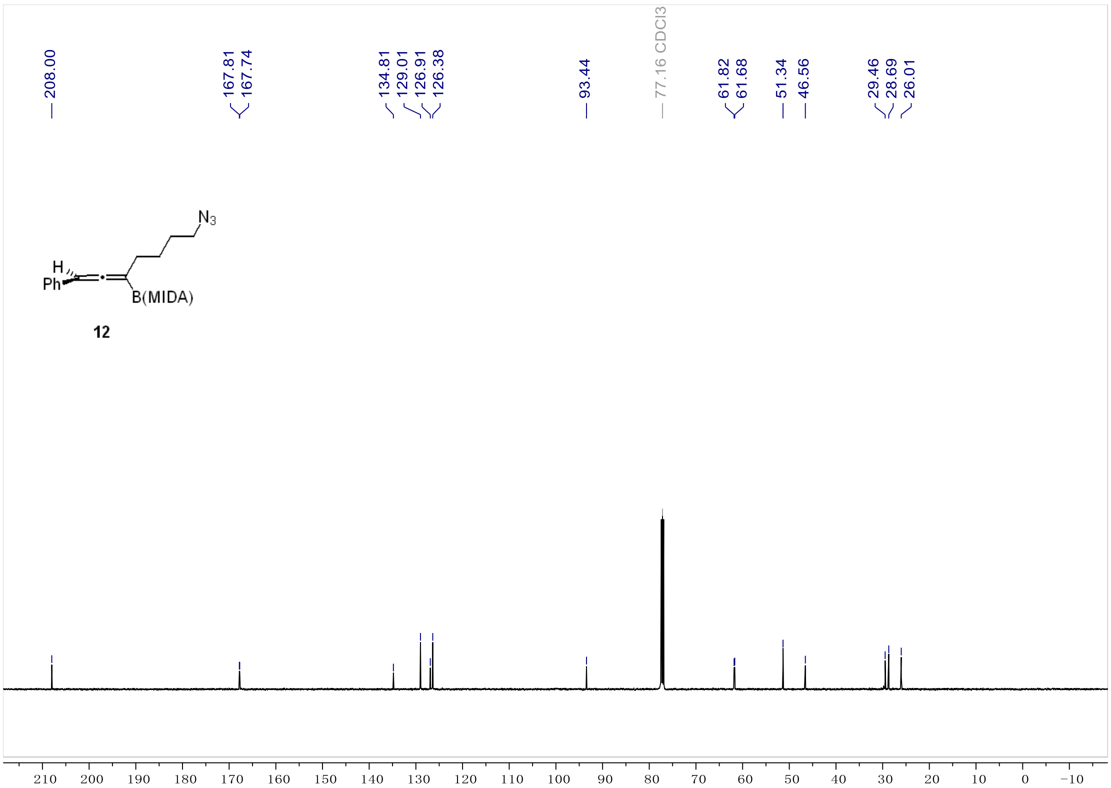

13: ^{1}H NMR (400 MHz, DMSO-d_6)

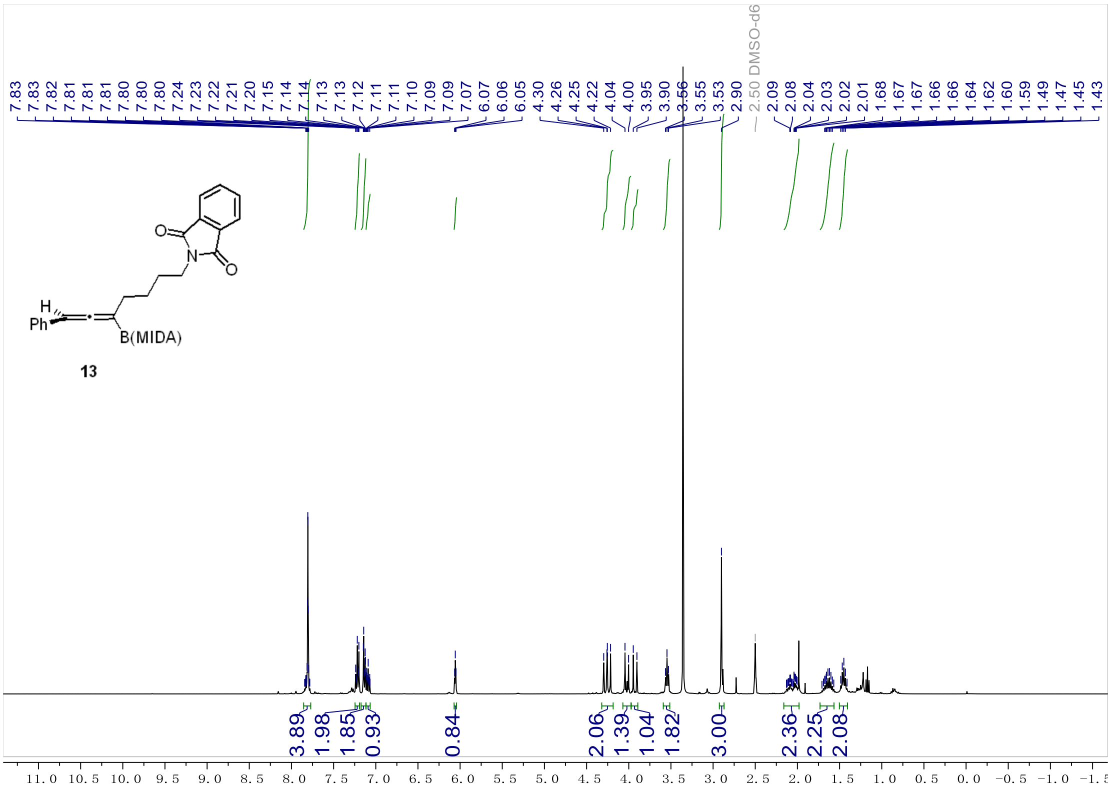

13: ^{13}C NMR (126 MHz, DMSO-d_6)

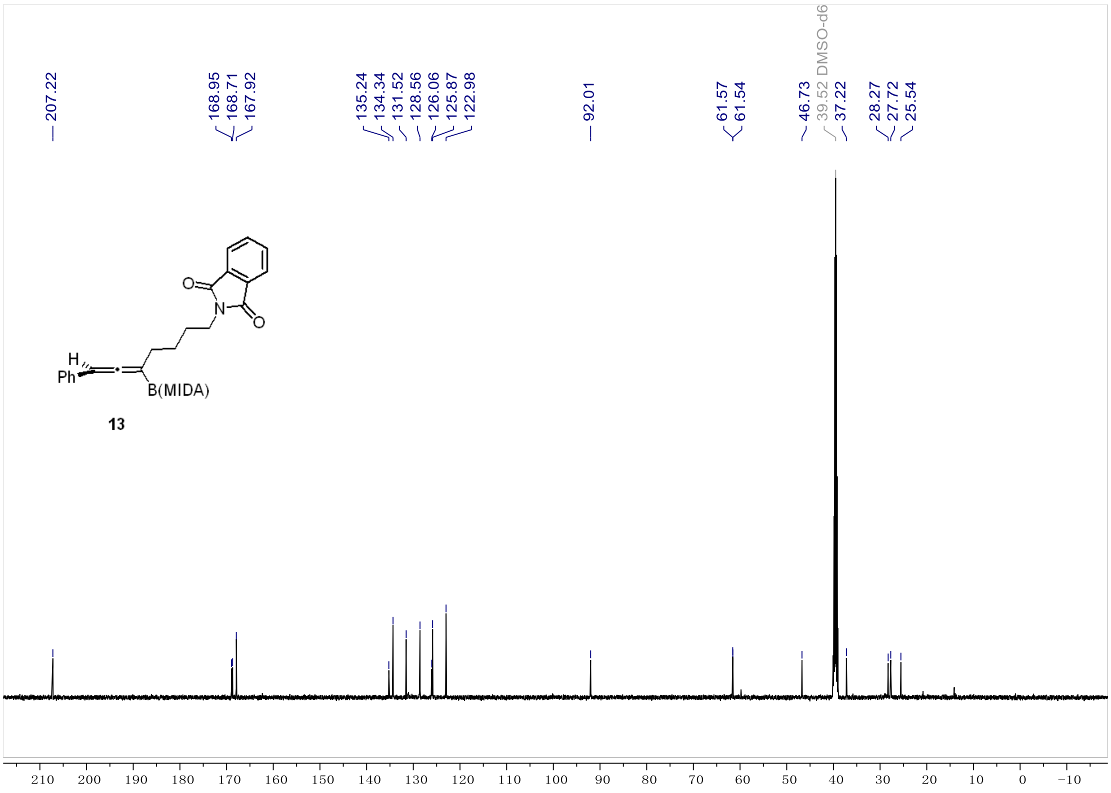

14: ^{1}H NMR (400 MHz, Chloroform-*d*)

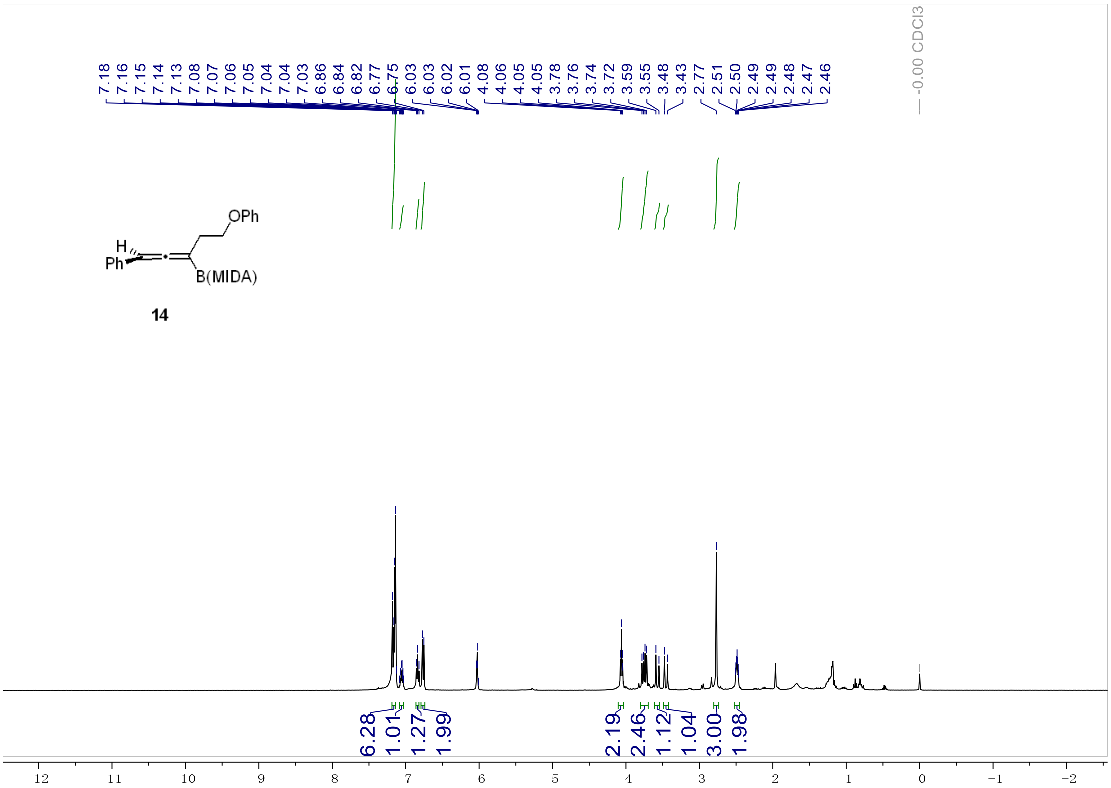

14: ^{13}C NMR (126 MHz, Chloroform-*d*)

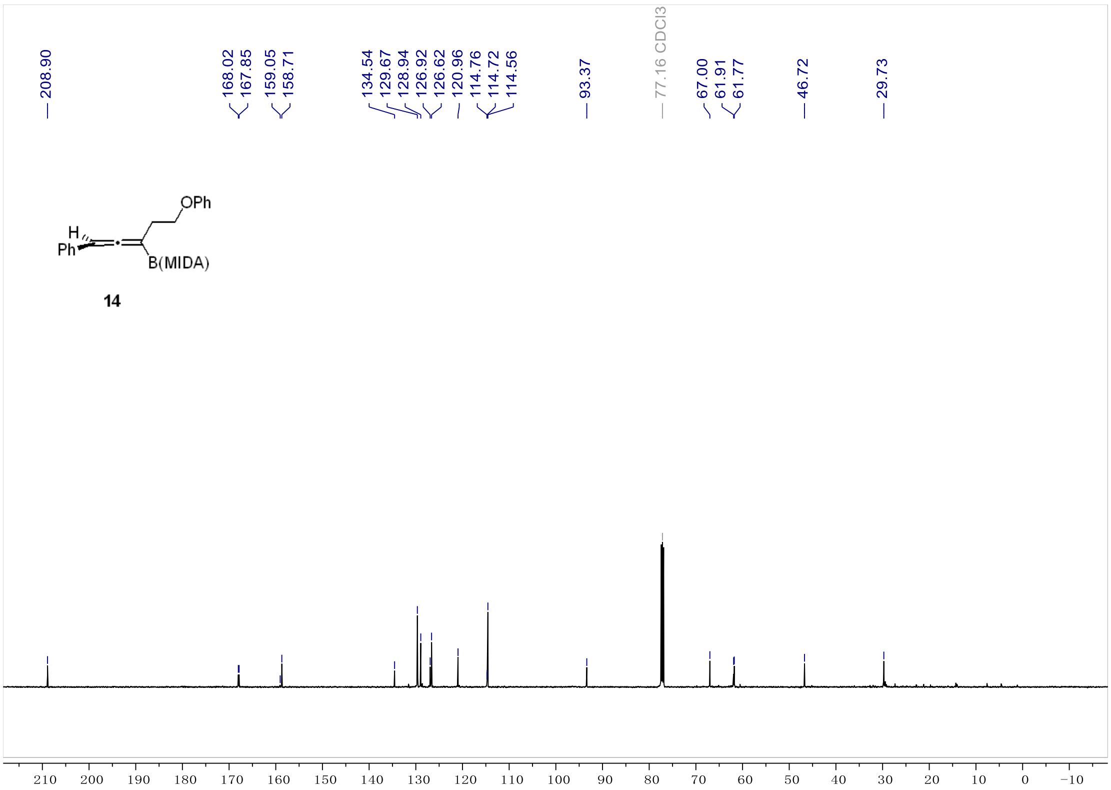

15: ^{1}H NMR (500 MHz, Chloroform-*d*)

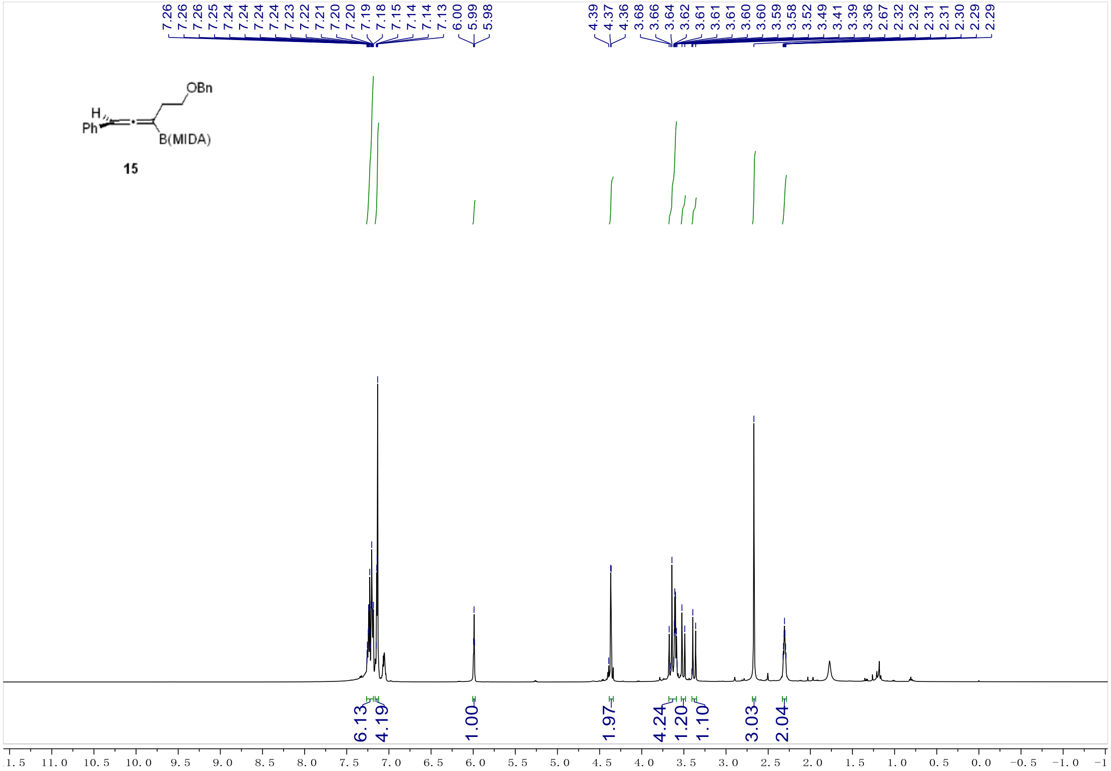

15: ^{13}C NMR (101 MHz, DMSO-*d*$_6$)

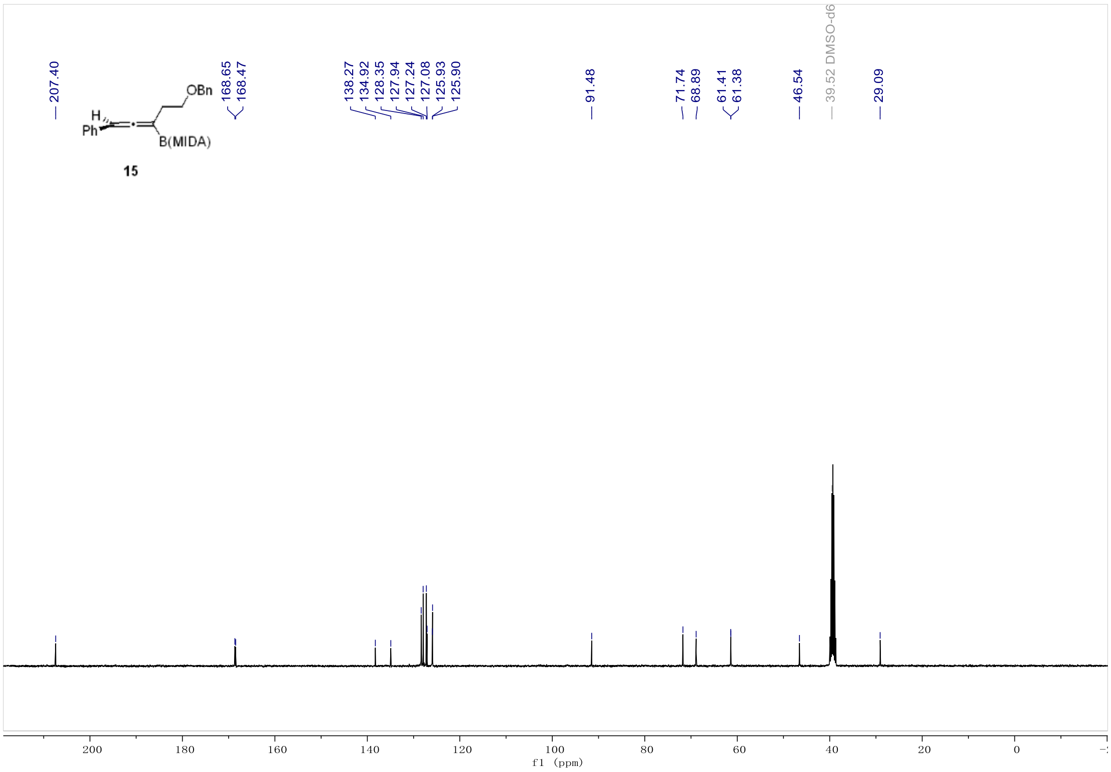

16: ^{1}H NMR (400 MHz, Acetone-*d*$_6$)

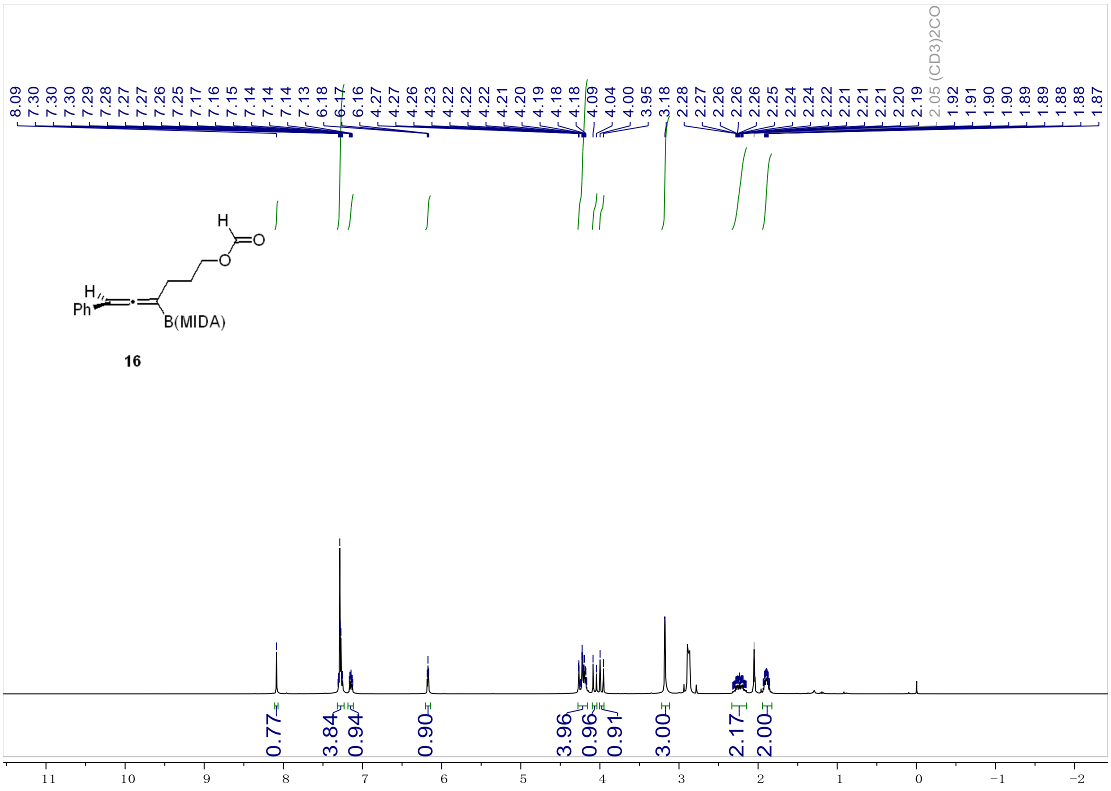

16: ^{13}C NMR (101 MHz, Acetone-*d*$_6$)

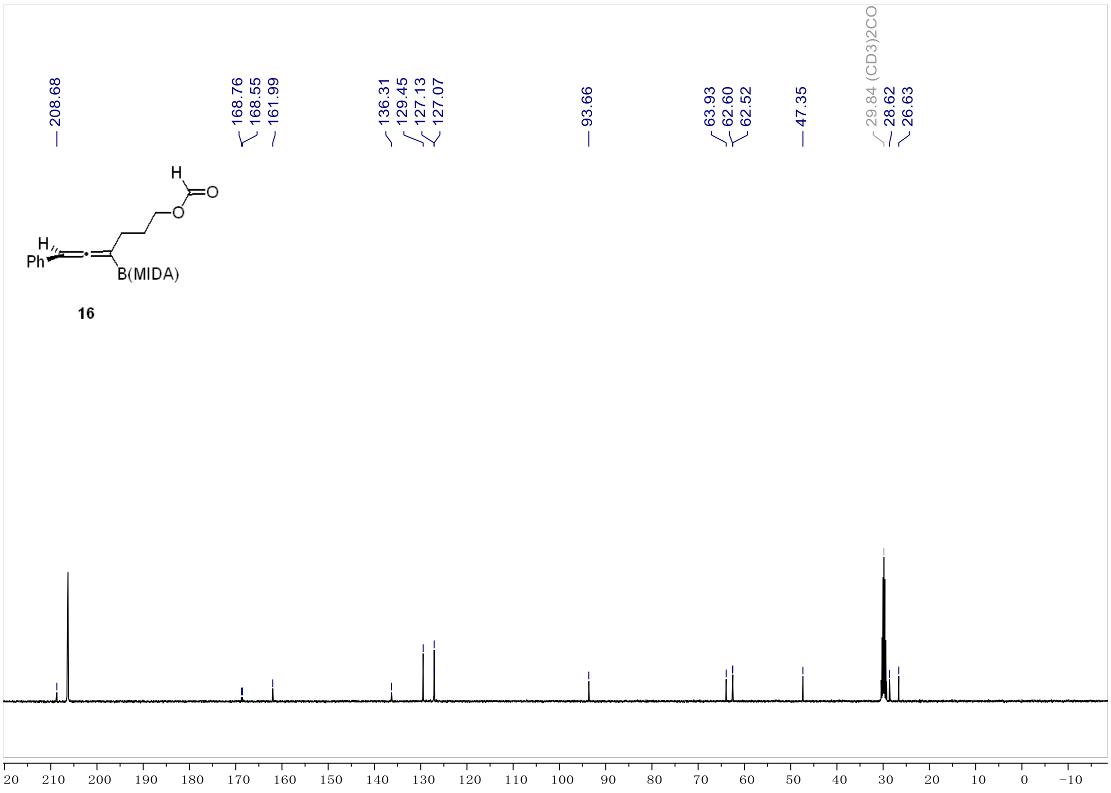

17: ^{1}H NMR (400 MHz, Acetone-d_6)

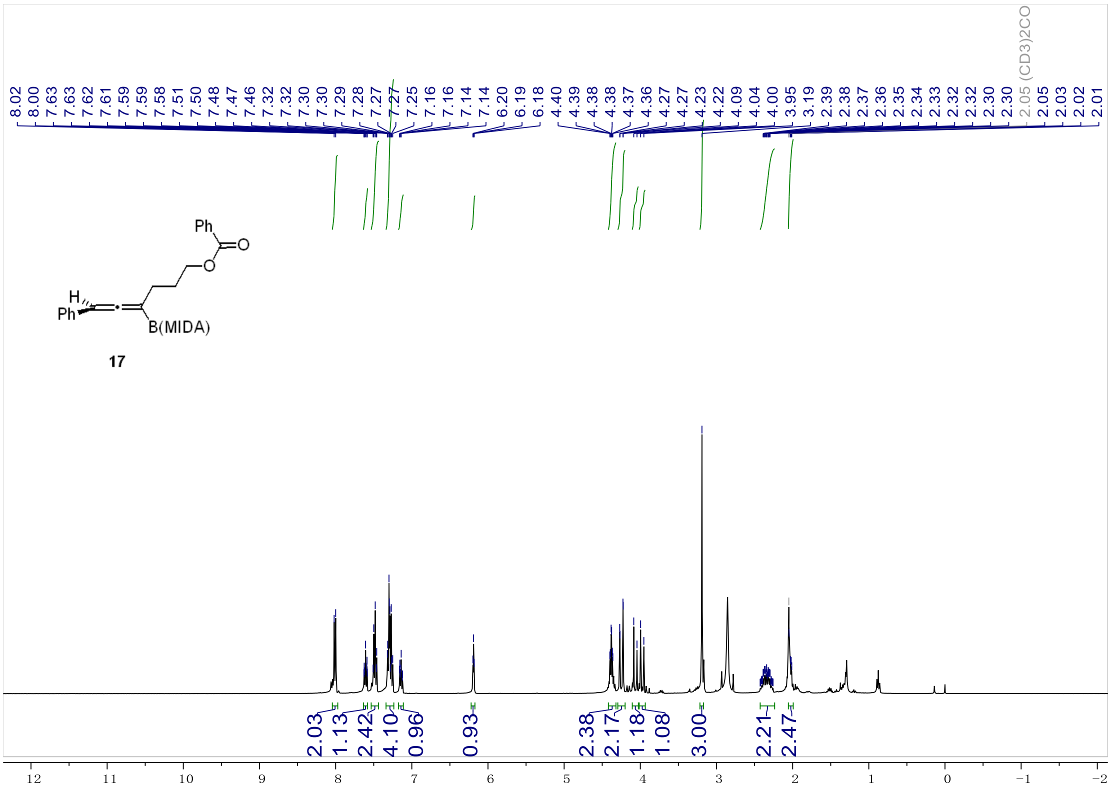

17: ^{13}C NMR (126 MHz, Chloroform-d)

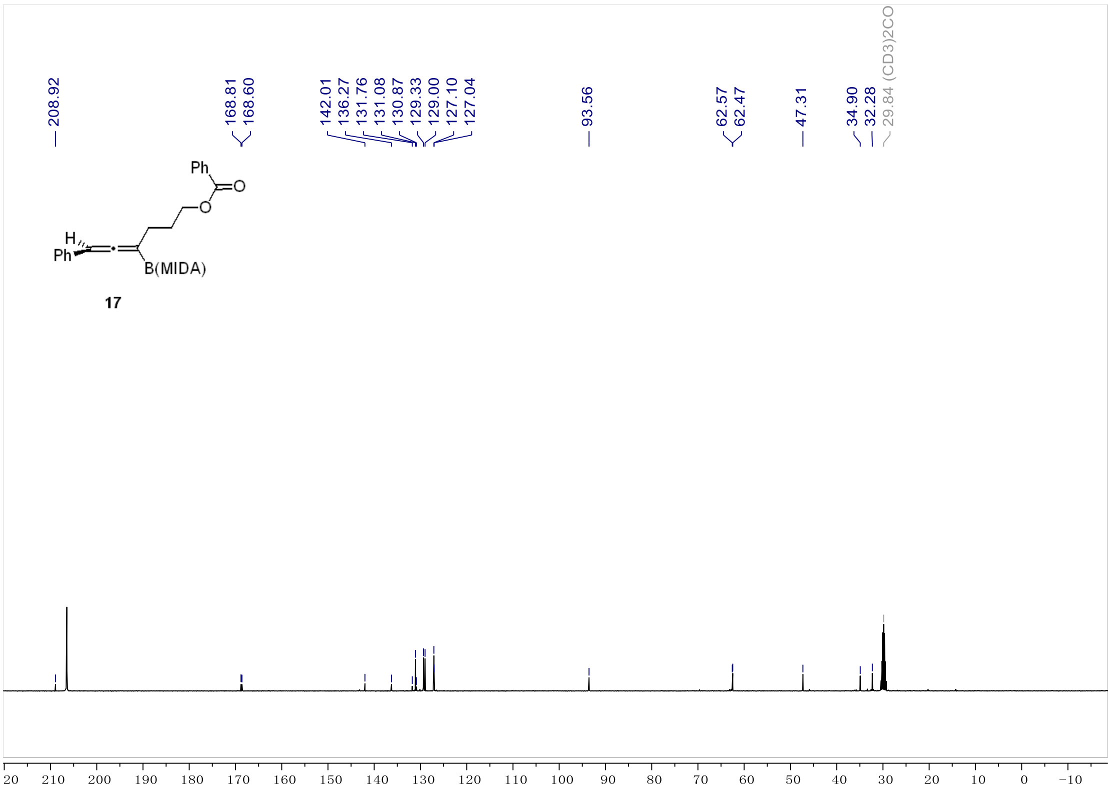

18: ^{1}H NMR (400 MHz, DMSO-d_6)

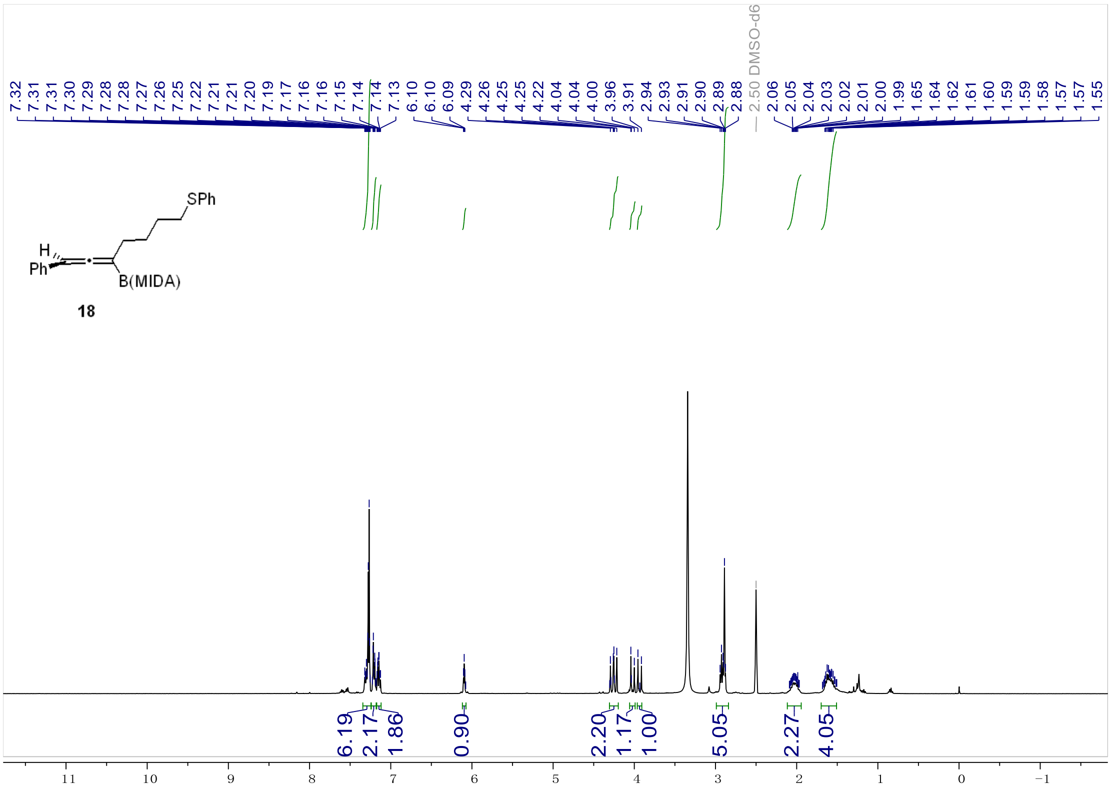

18: ^{13}C NMR (101 MHz, DMSO-d_6)

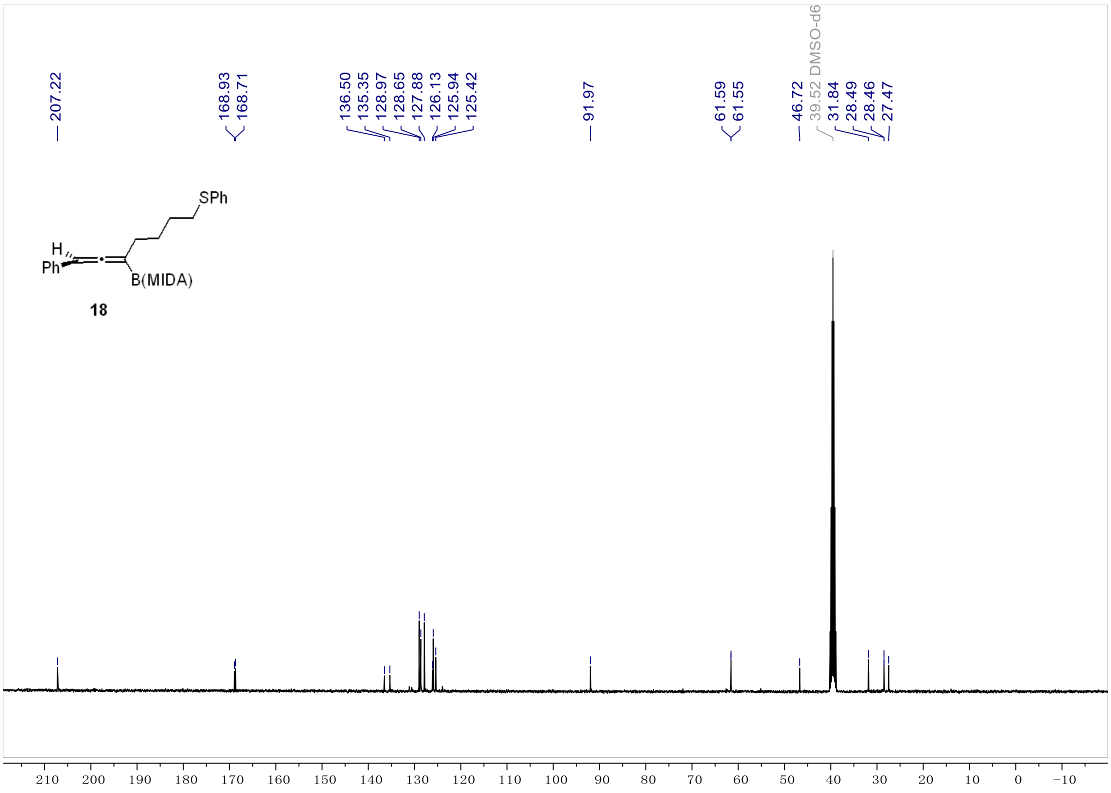

19: ^{1}H NMR (400 MHz, Acetone-d_6)

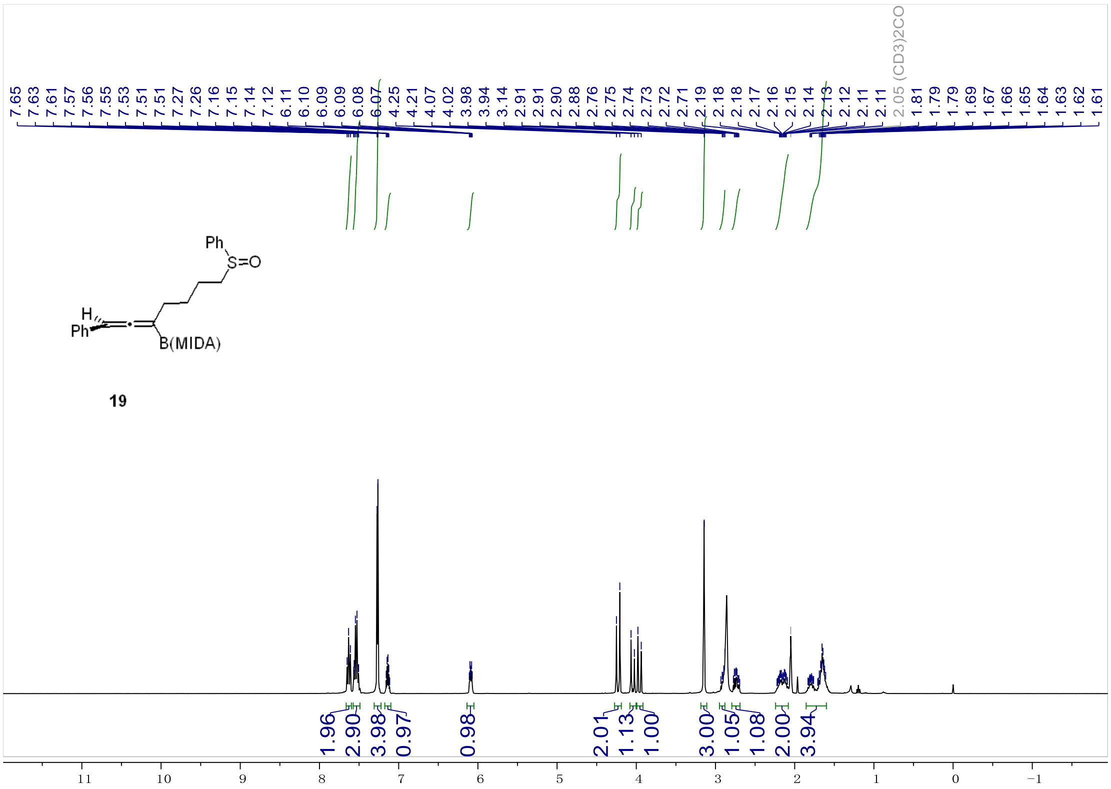

19: ^{13}C NMR (126 MHz, Acetone-d_6)

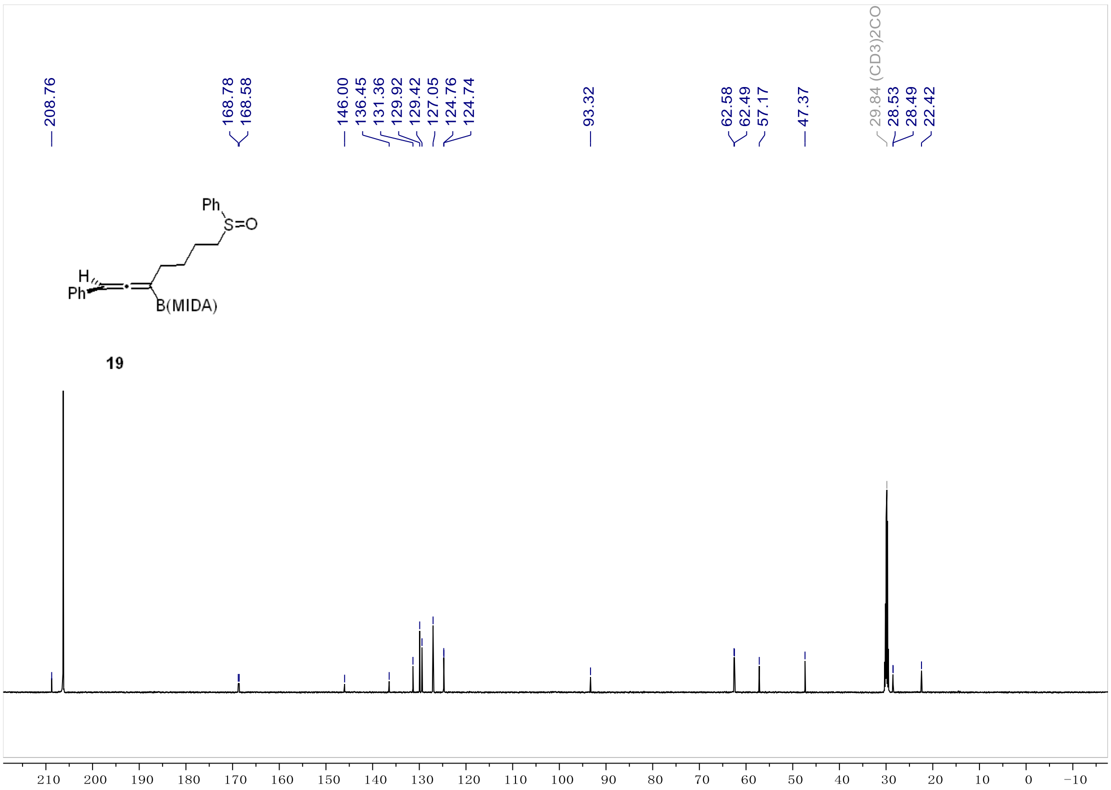

20: ^{1}H NMR (400 MHz, Chloroform-*d*)

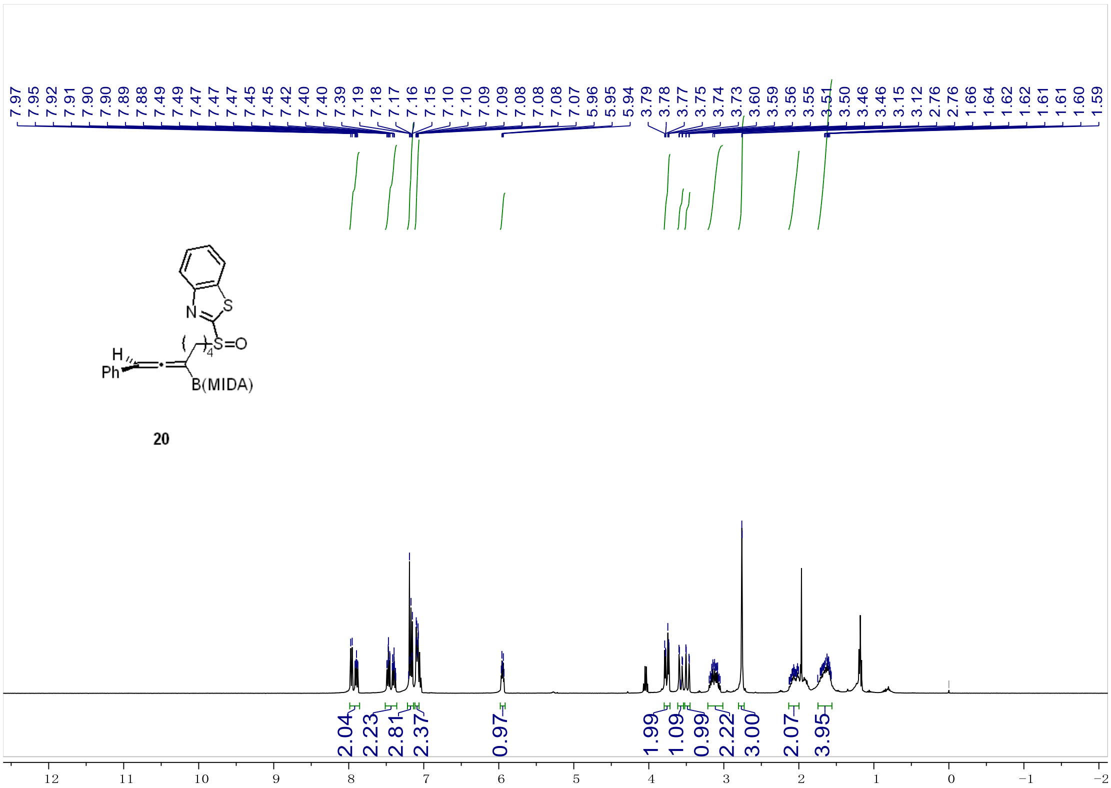

20: ^{13}C NMR (101 MHz, Chloroform-*d*)

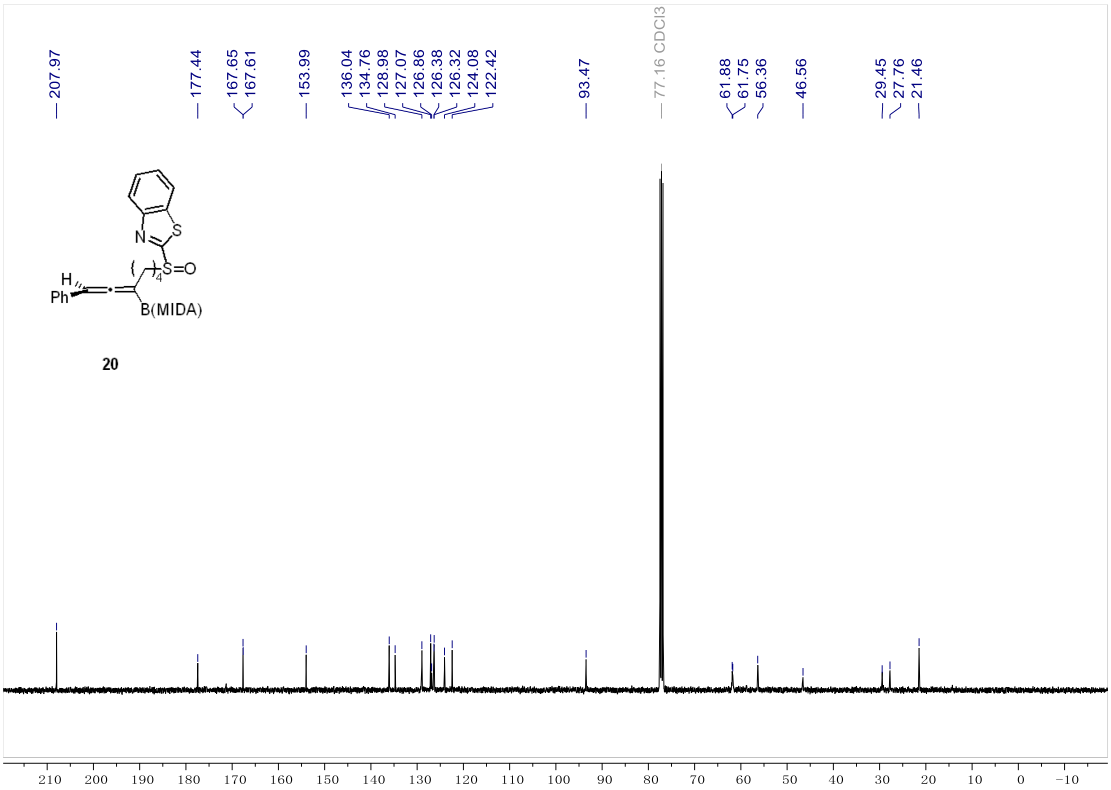

21: ^{1}H NMR (400 MHz, DMSO-d_6)

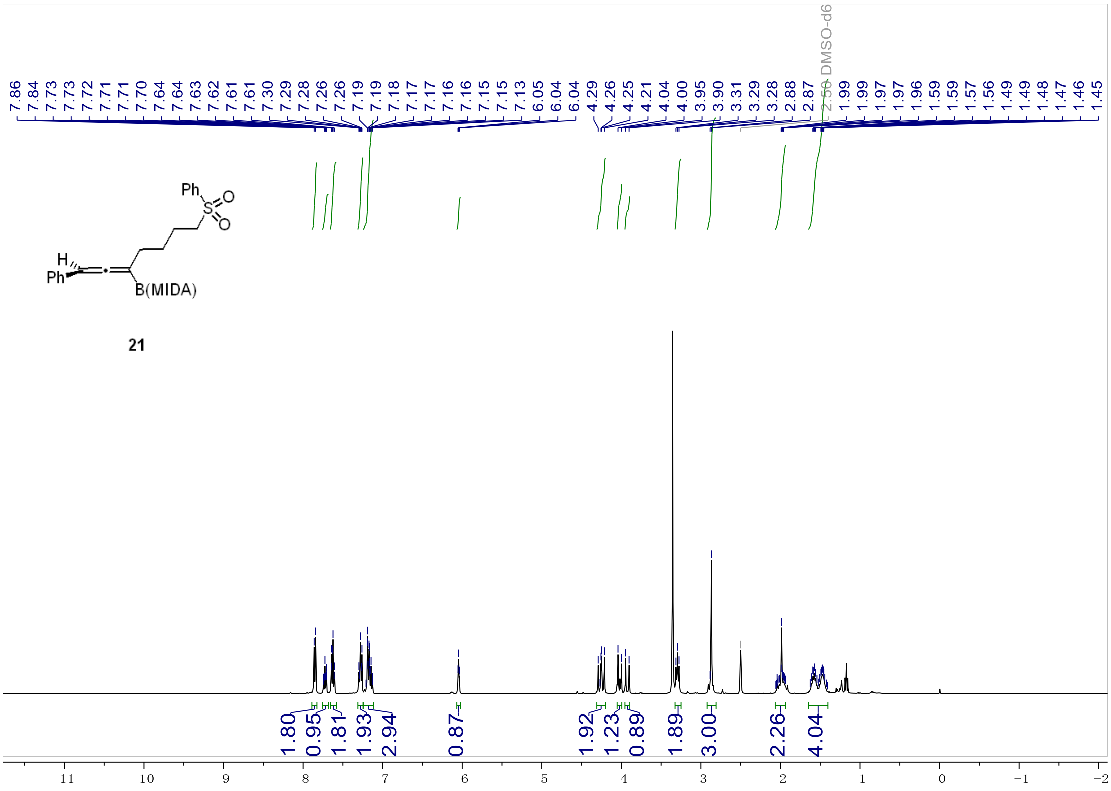

21: ^{13}C NMR (126 MHz, DMSO-d_6)

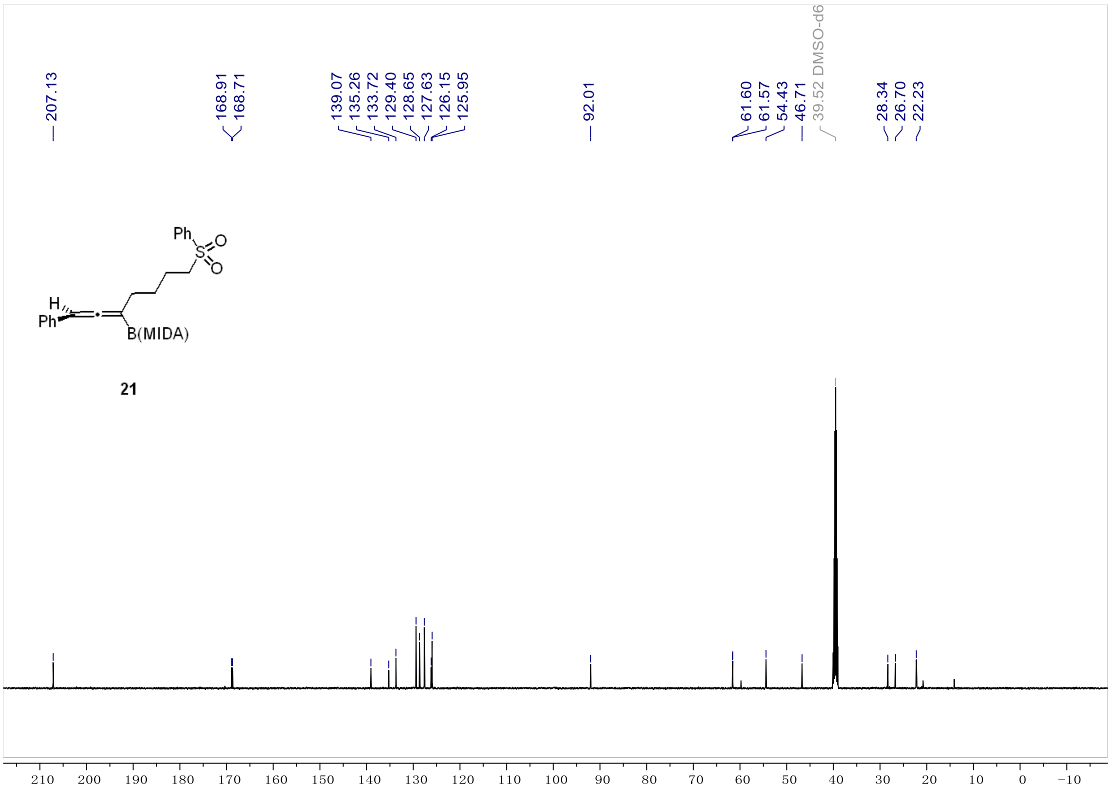

22: ^{1}H NMR (400 MHz, DMSO-*d$_6$*)

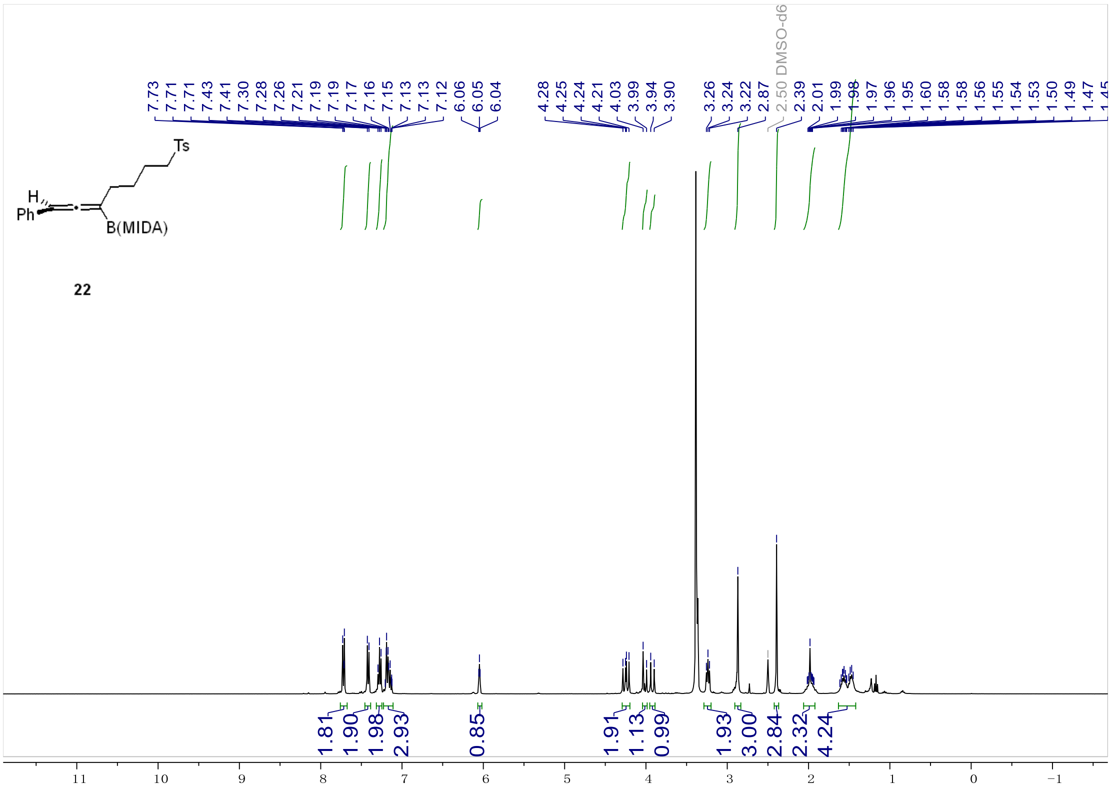

22: ^{13}C NMR (101 MHz, DMSO-*d$_6$*)

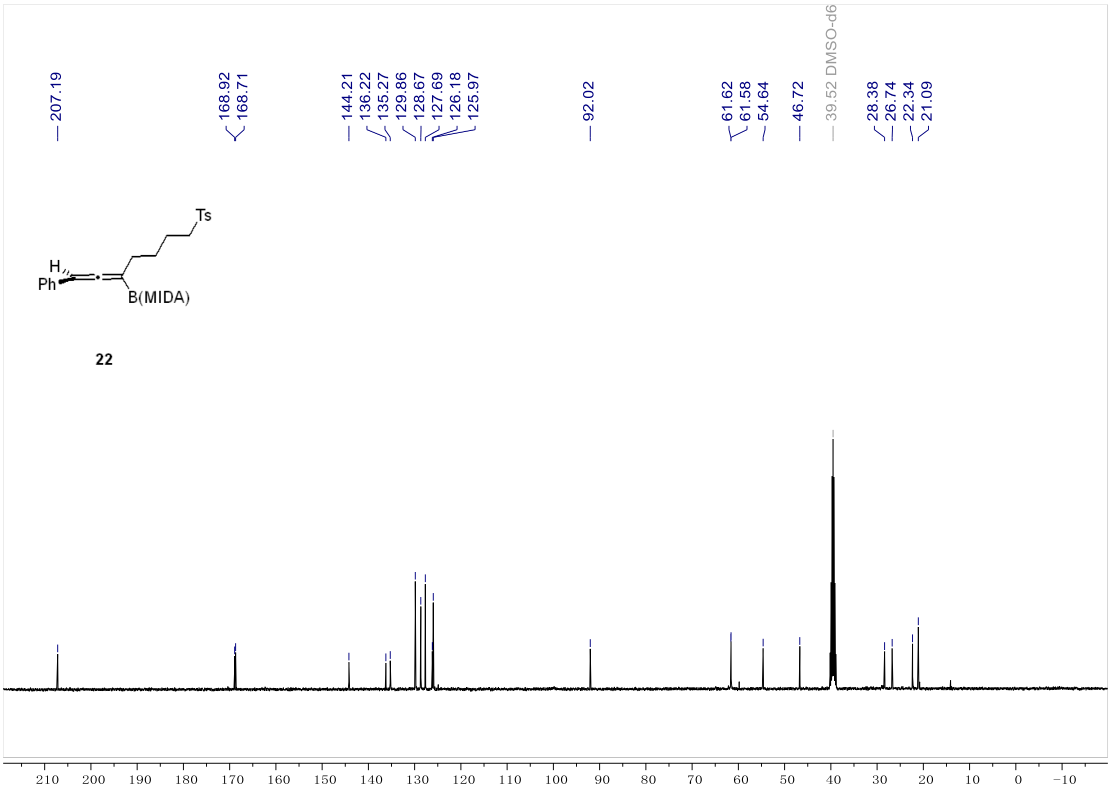

23: ^{1}H NMR (500 MHz, DMSO-d_6)

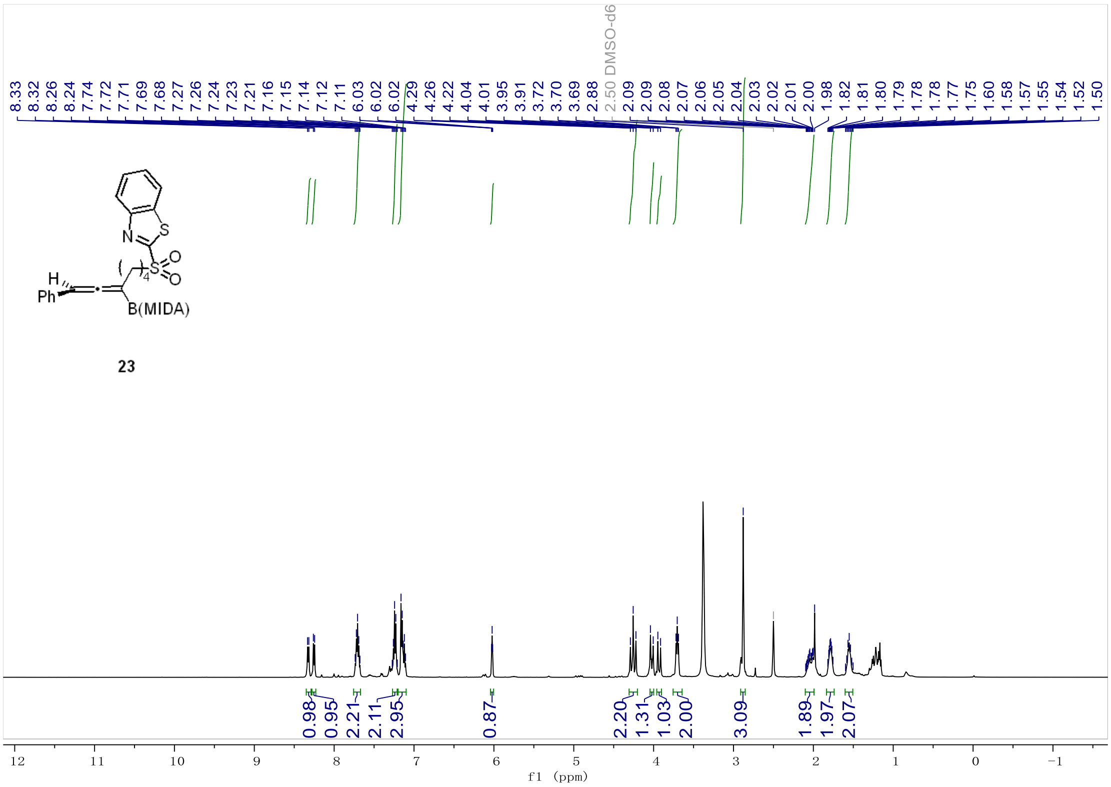

23: ^{13}C NMR (126 MHz, DMSO-d_6)

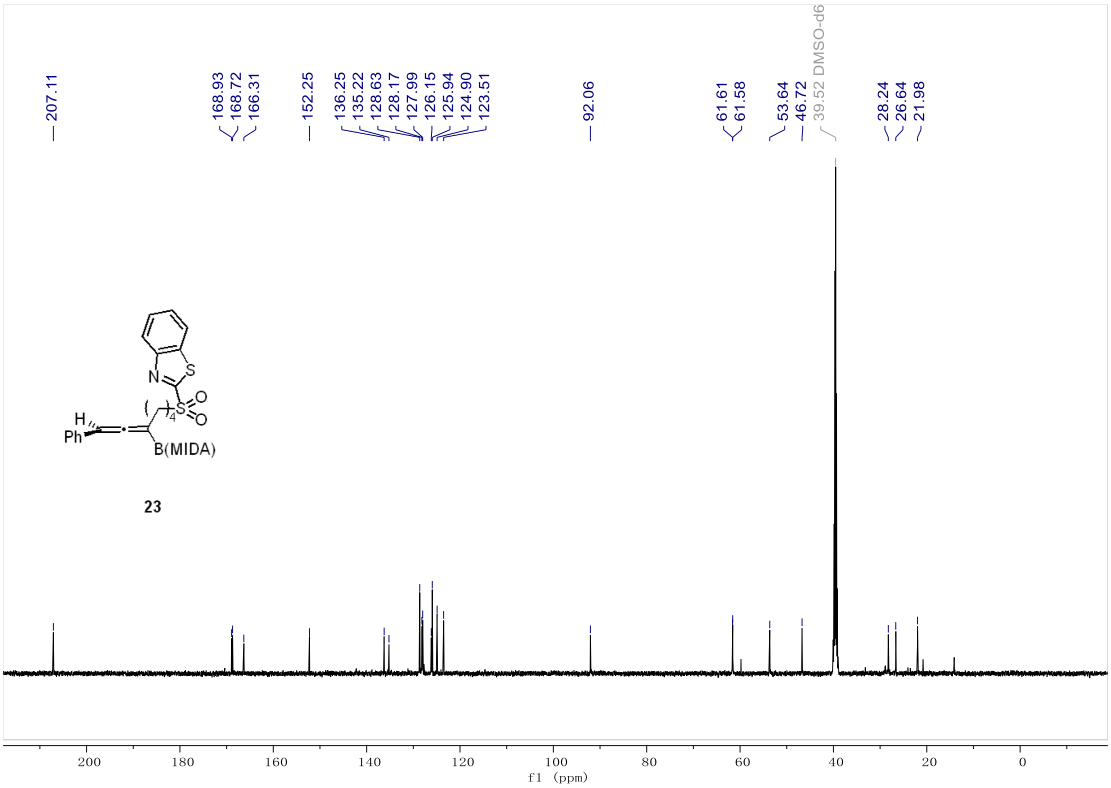

24: ^{1}H NMR (400 MHz, DMSO-d_6)

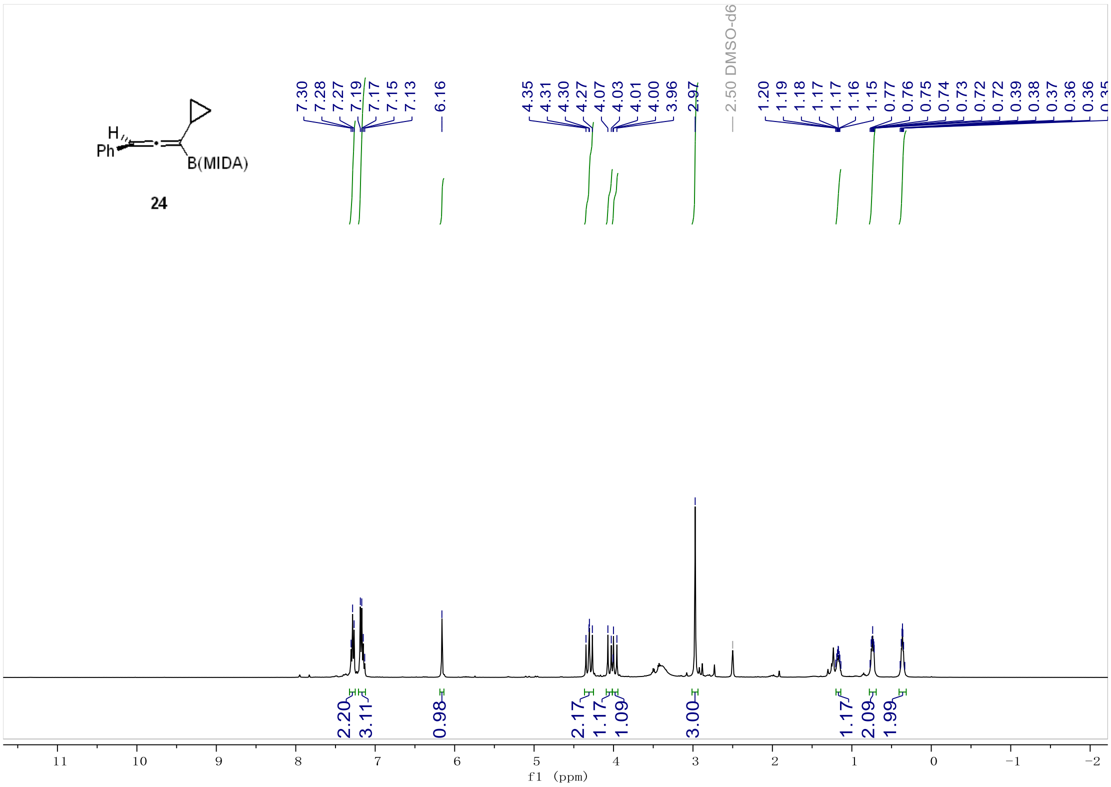

24: ^{13}C NMR (101 MHz, DMSO-d_6)

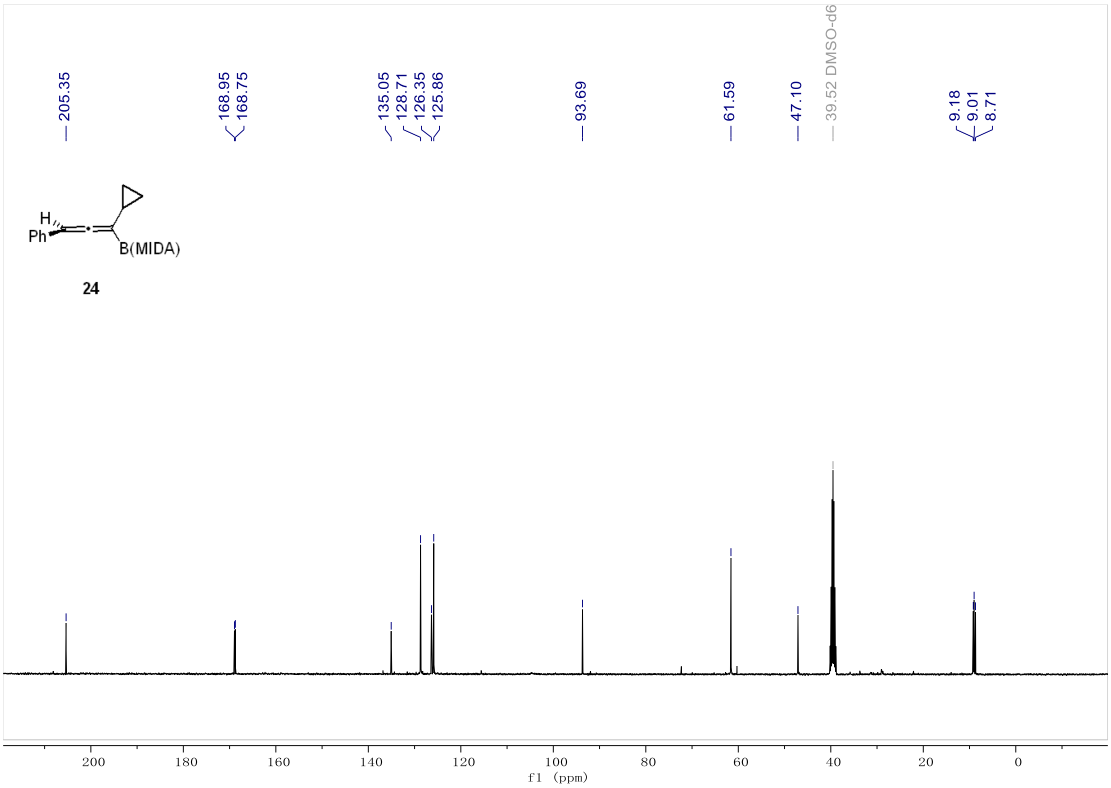

25: ^{1}H NMR (400 MHz, Acetonitrile-d_3)

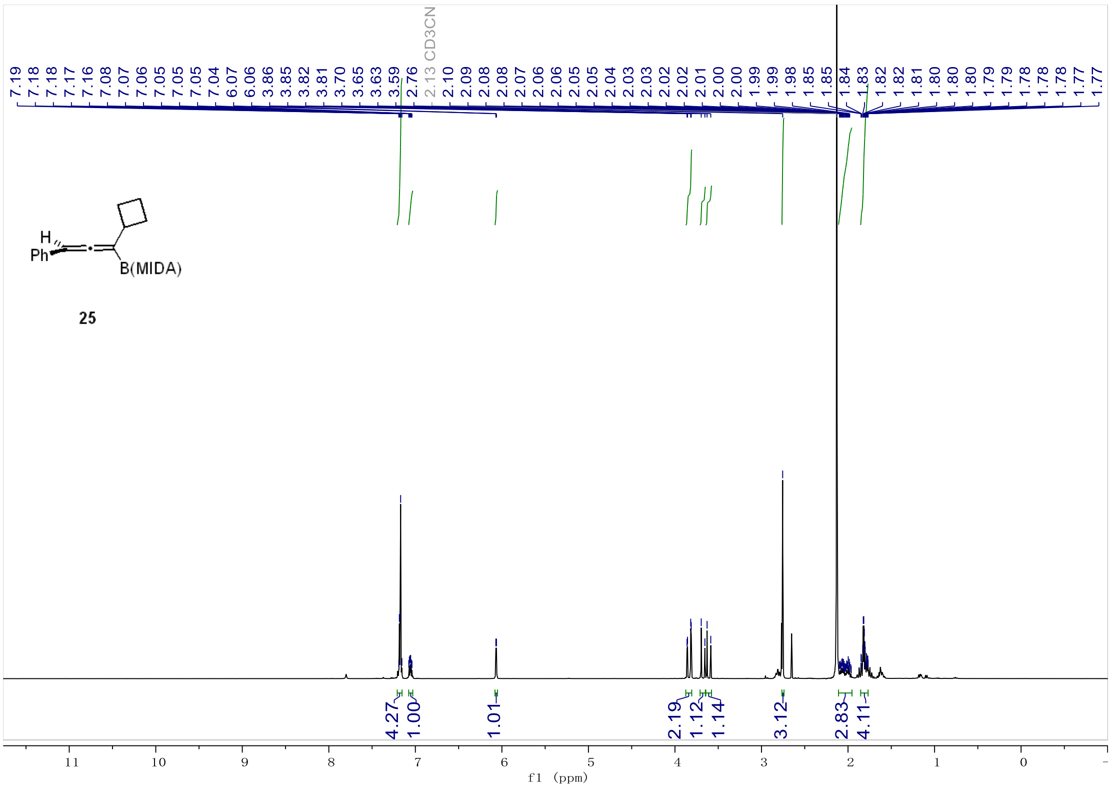

25: ^{13}C NMR (101 MHz, Acetonitrile-d_3)

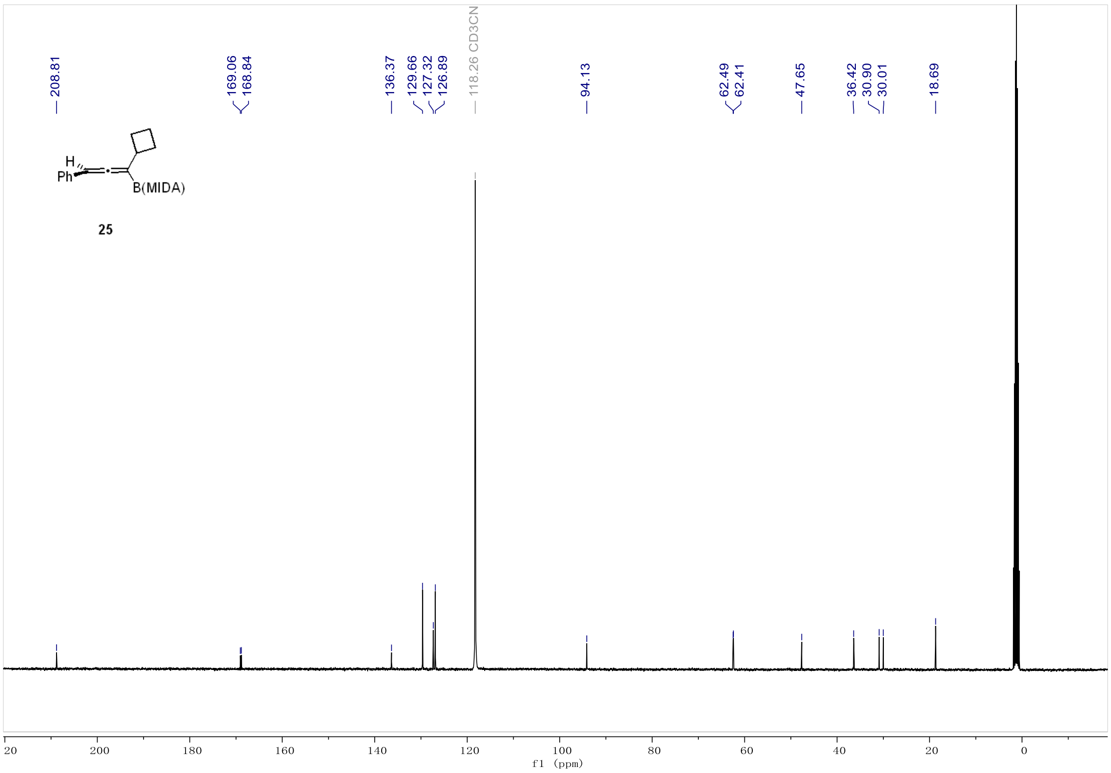

26: ^{1}H NMR (400 MHz, DMSO-d_6)

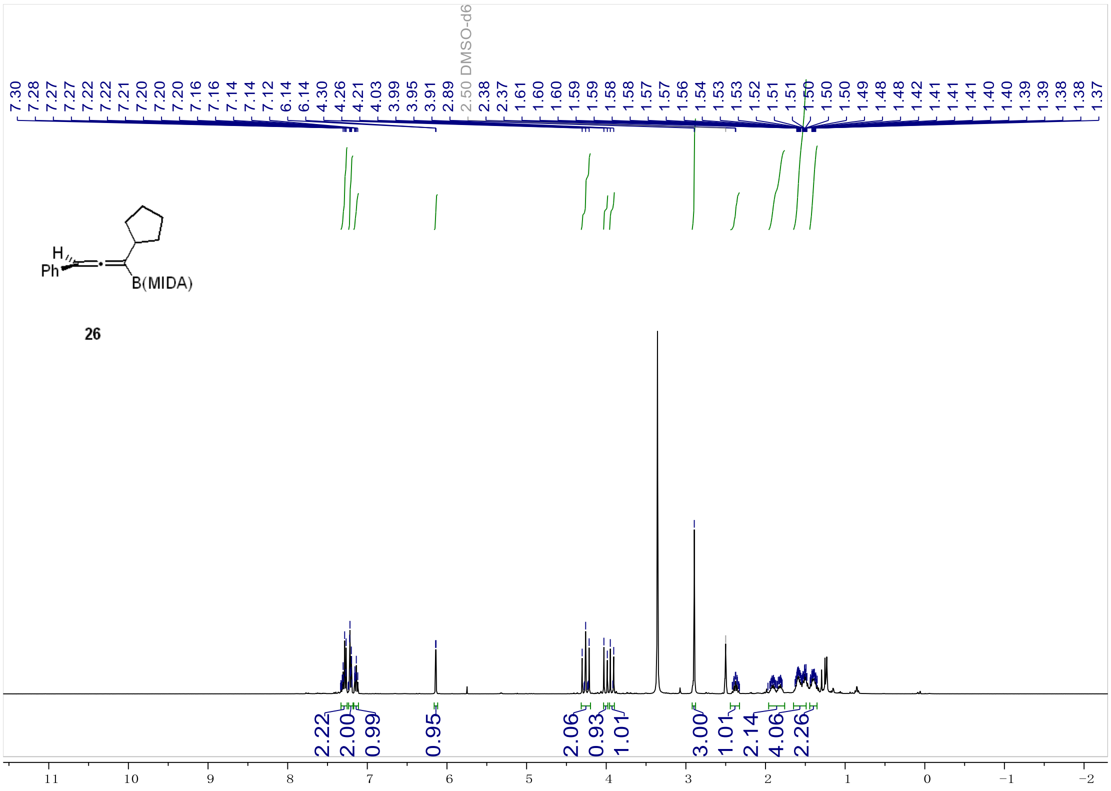

26: ^{13}C NMR (101 MHz, DMSO-d_6)

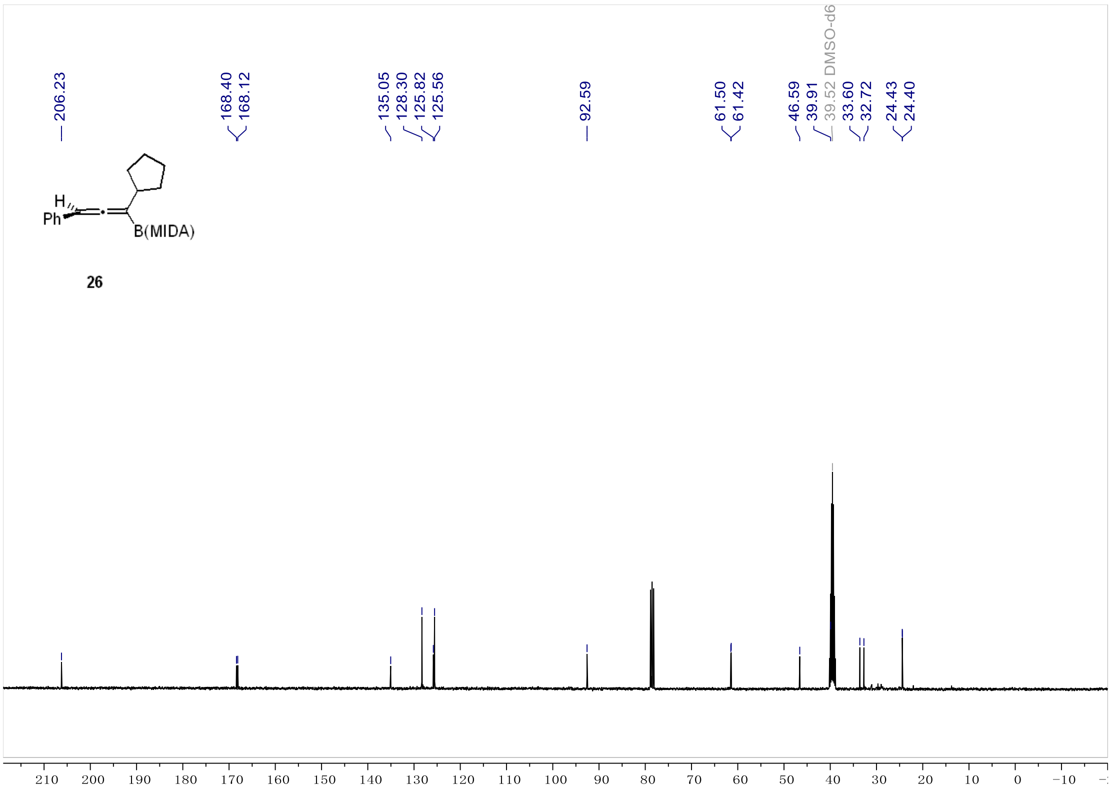

27: ^{1}H NMR (500 MHz, Chloroform-*d*)

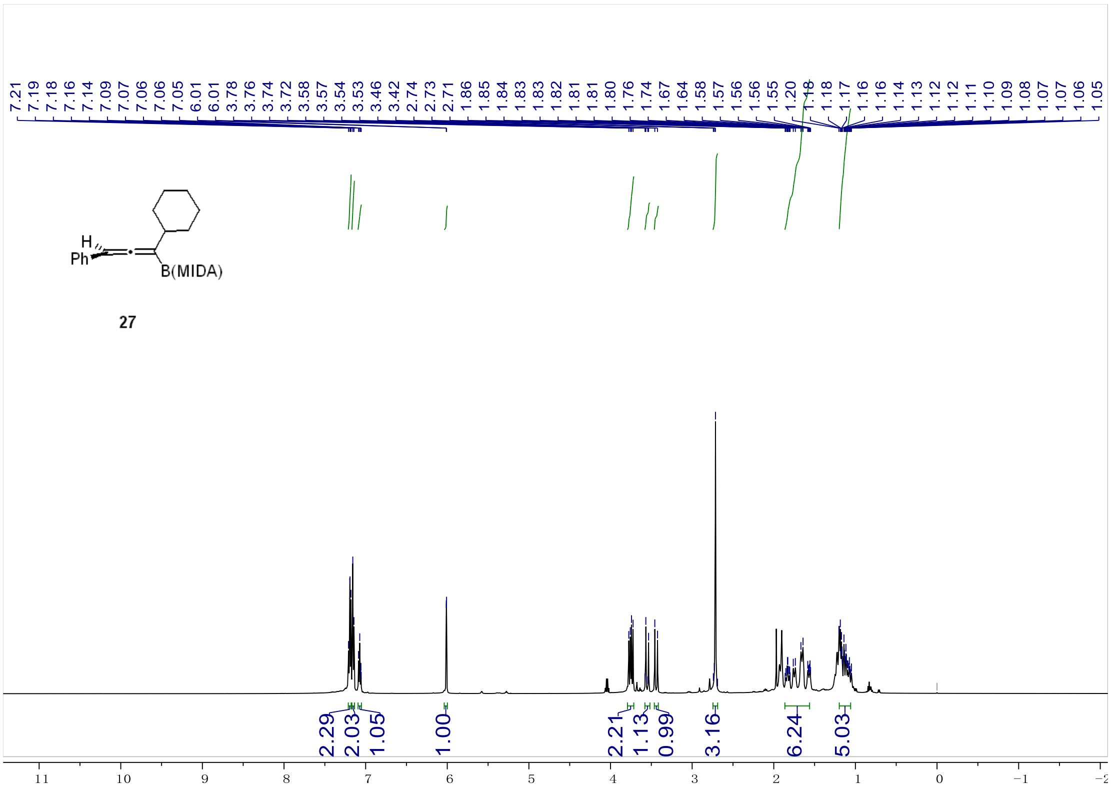

27: ^{13}C NMR (126 MHz, Chloroform-*d*)

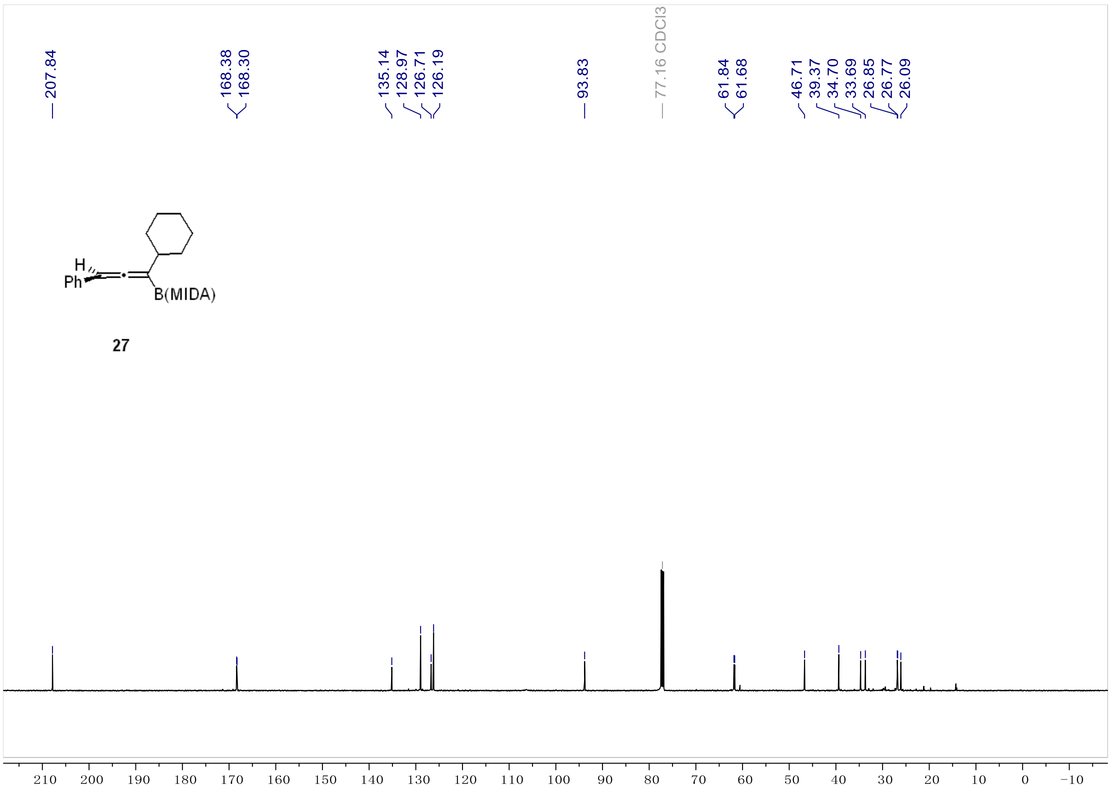

28: ^{1}H NMR (500 MHz, Chloroform-*d*)

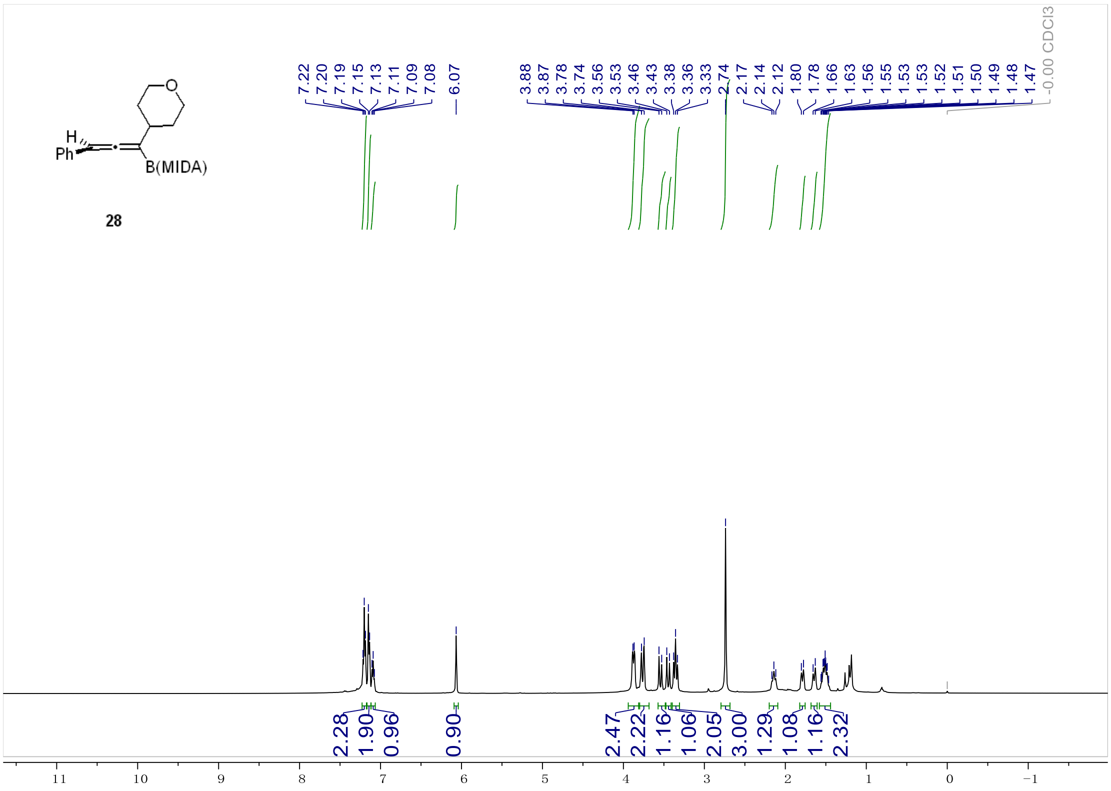

28: ^{13}C NMR (126 MHz, Chloroform-*d*)

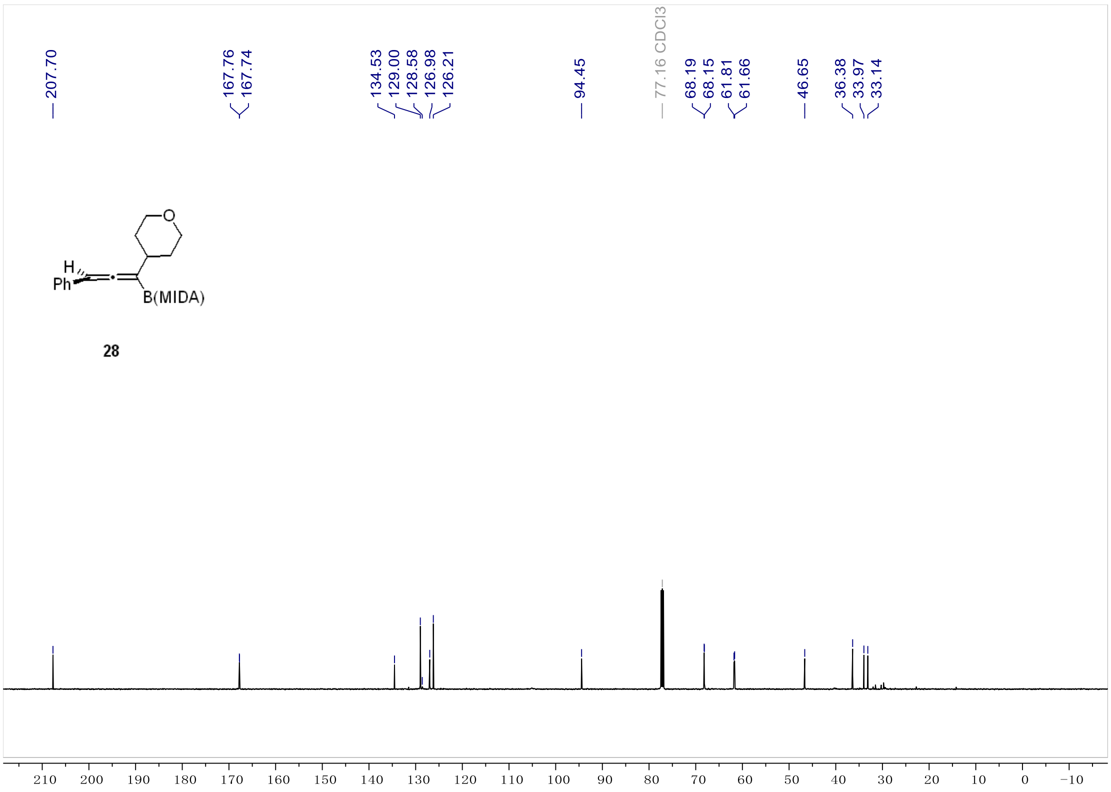

29: ¹H NMR (400 MHz, Acetone-*d*₆)

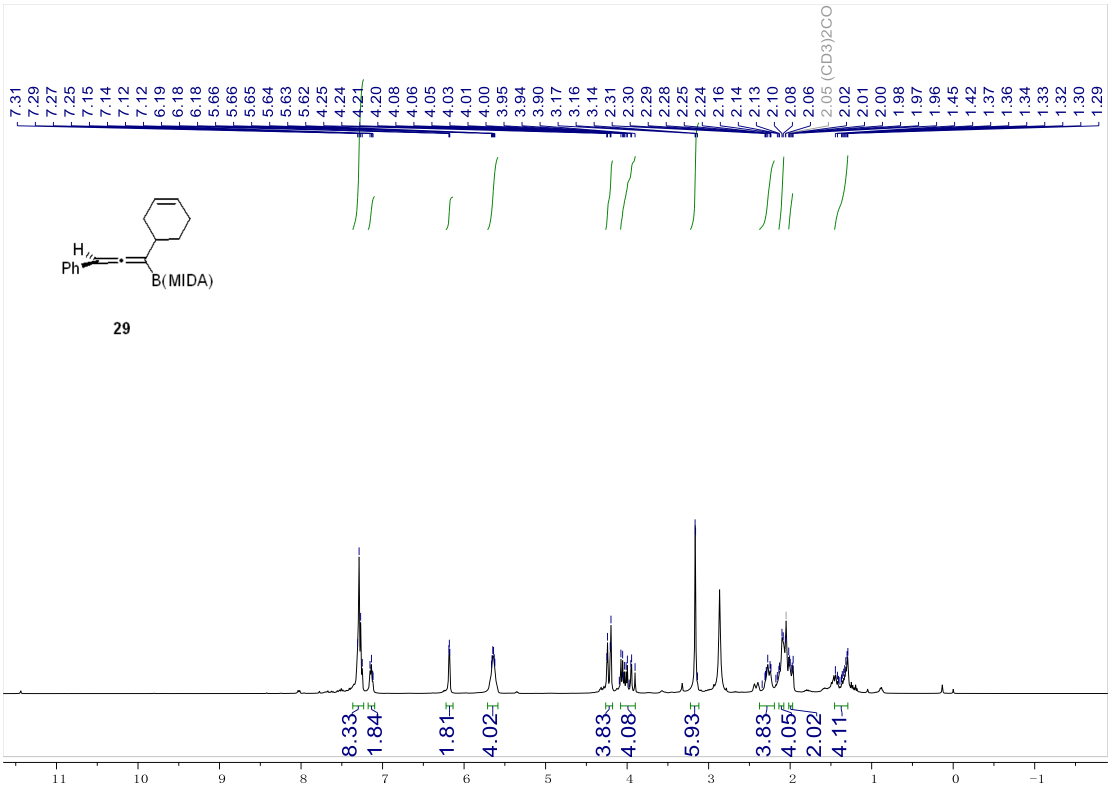

29: ¹³C NMR (126 MHz, Acetone-*d*₆)

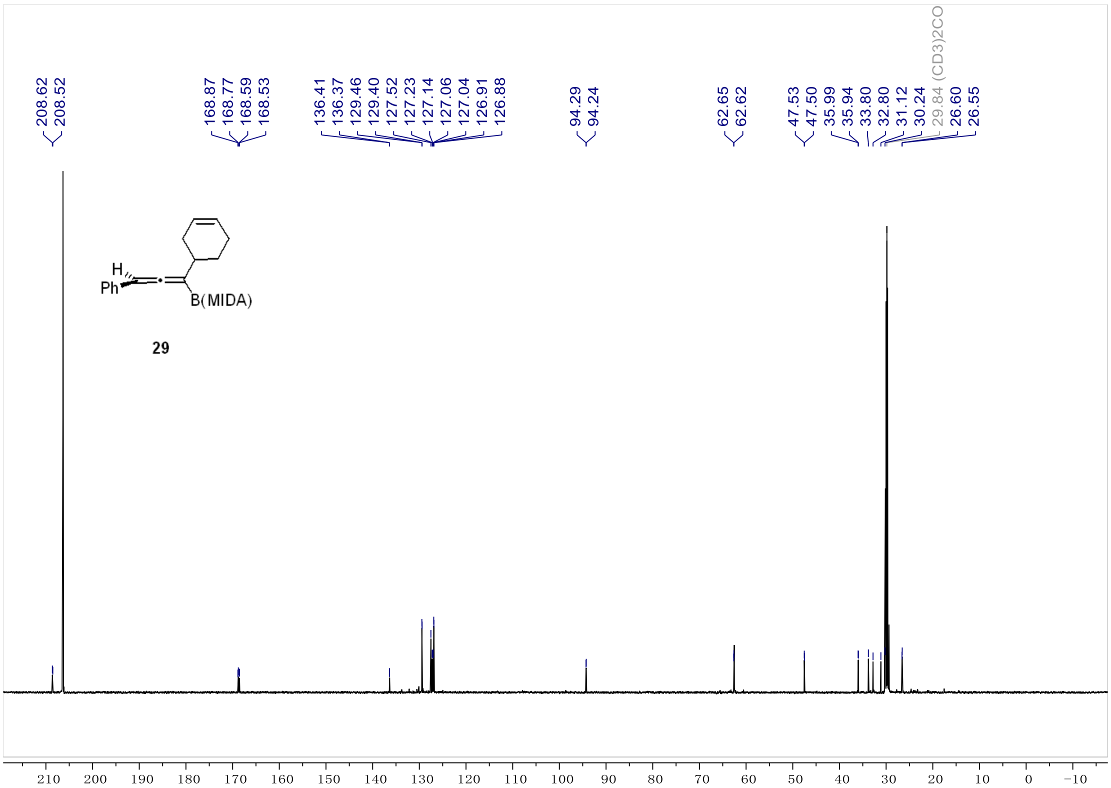

30: ^{1}H NMR (500 MHz, DMSO-d_6)

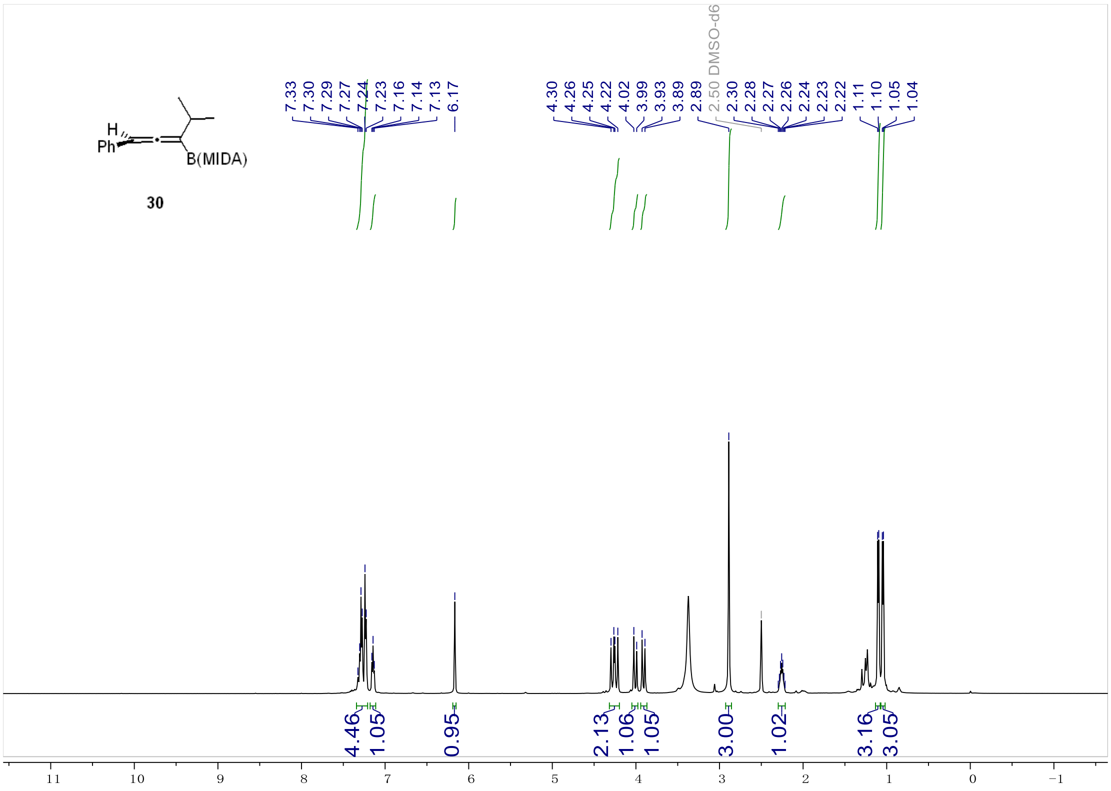

30: ^{13}C NMR (126 MHz, DMSO-d_6)

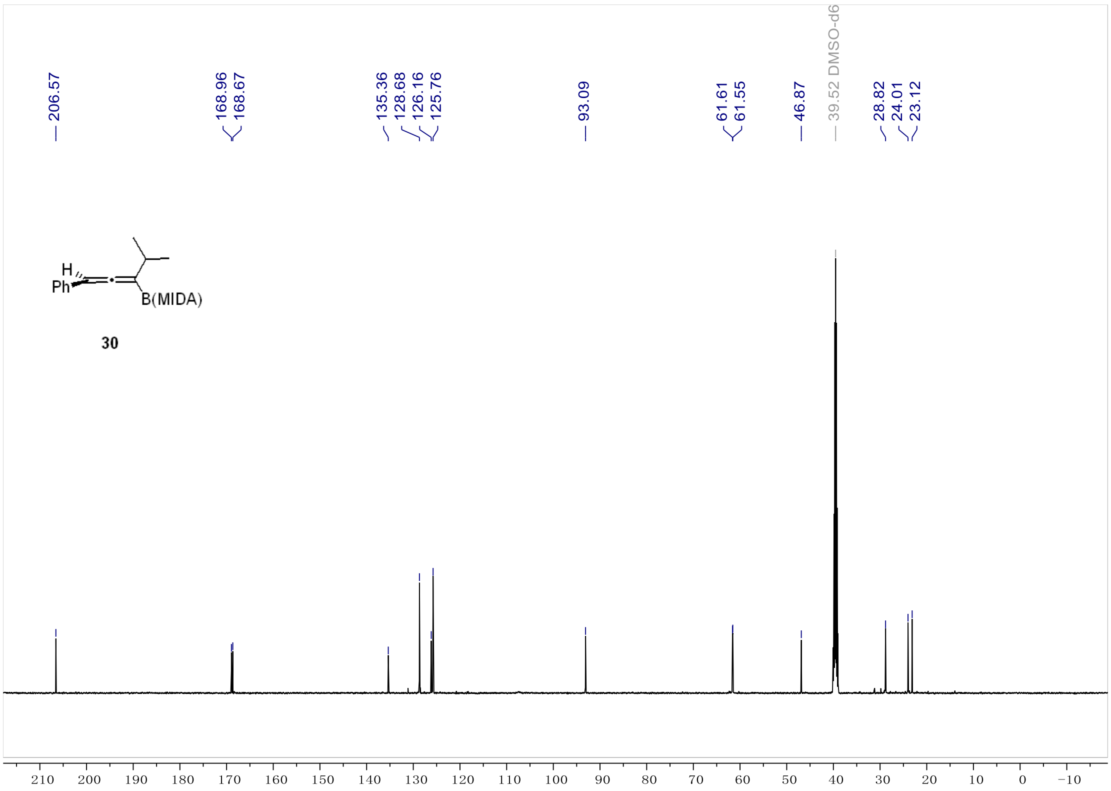

31: ^{1}H NMR (400 MHz, Acetone-d_6)

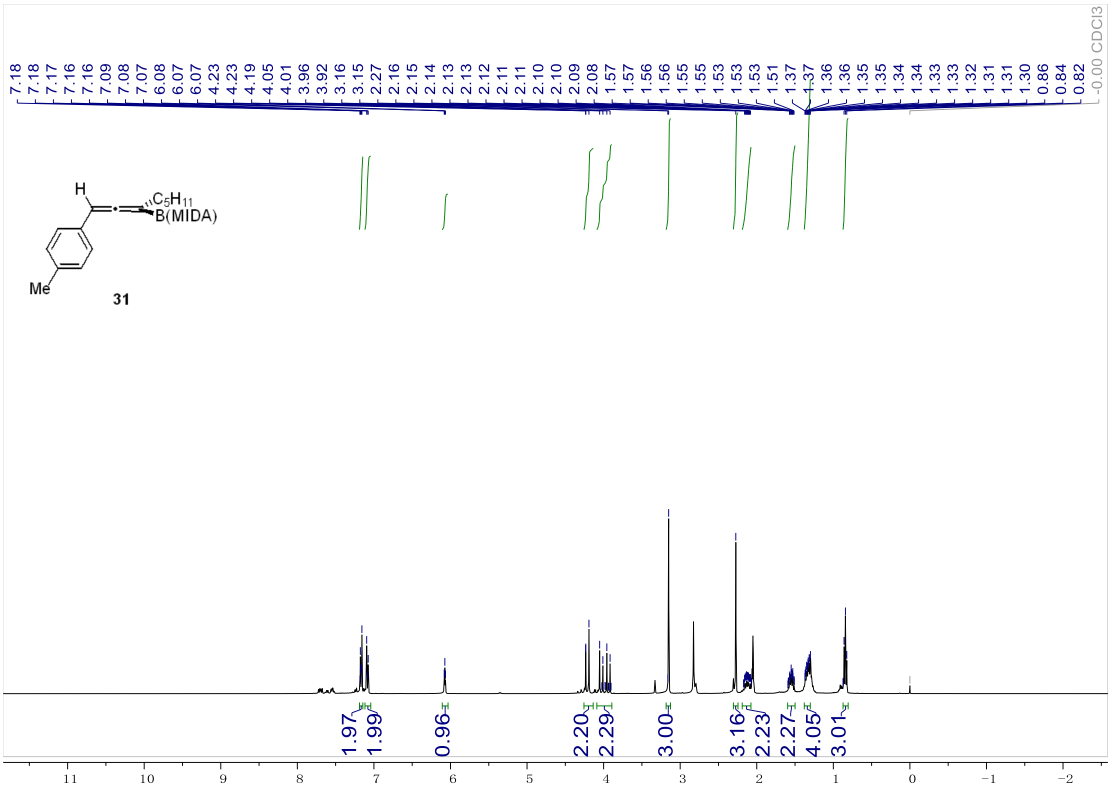

31: ^{13}C NMR (101 MHz, Acetone-d_6)

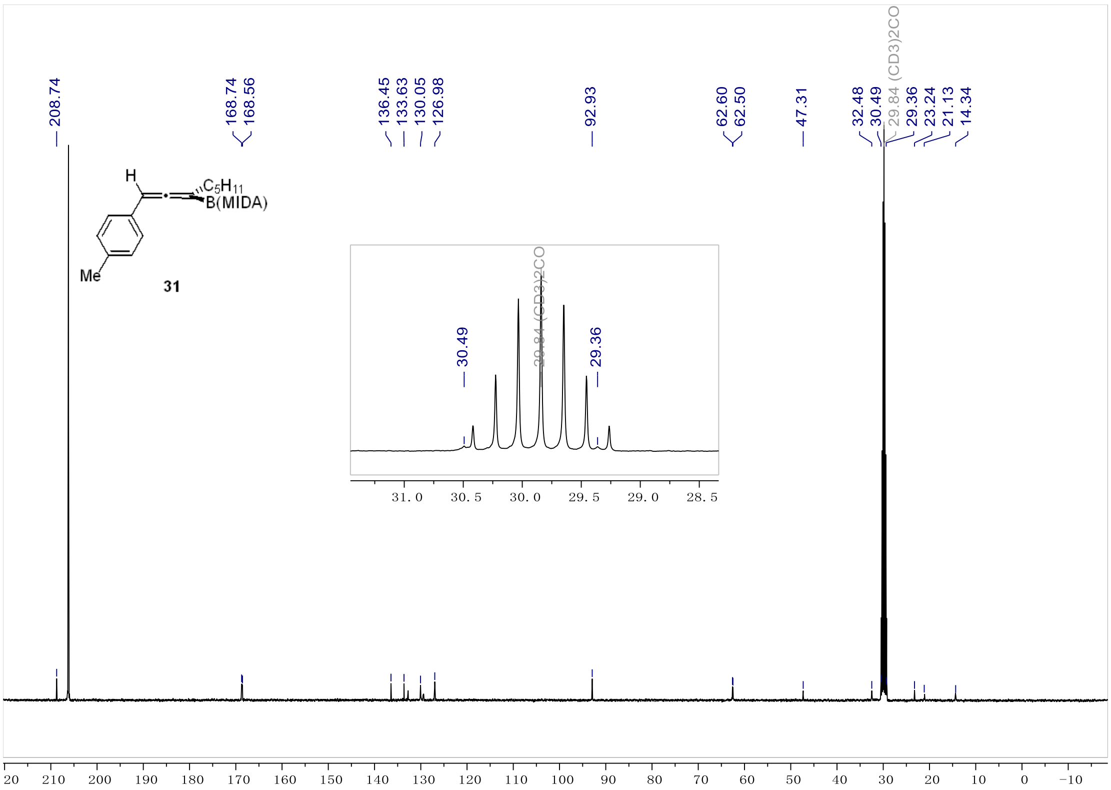

32: ^{1}H NMR (500 MHz, Acetone-d_6)

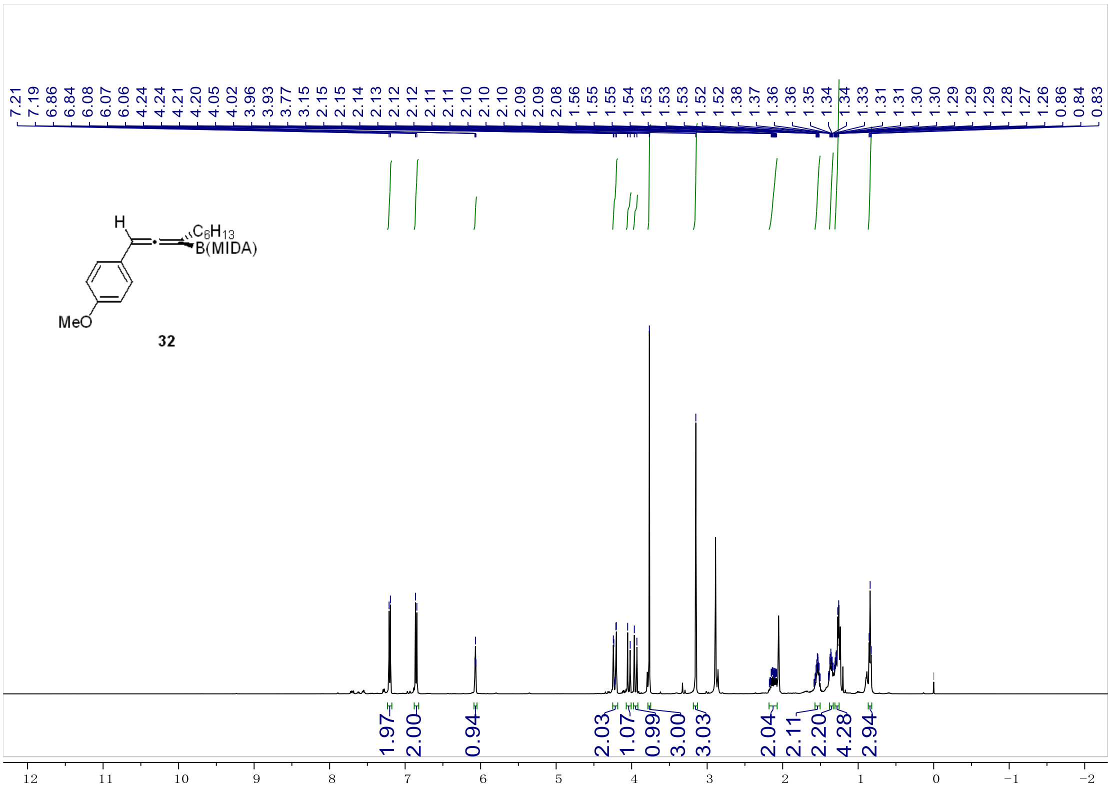

32: ^{13}C NMR (101 MHz, Acetone-d_6)

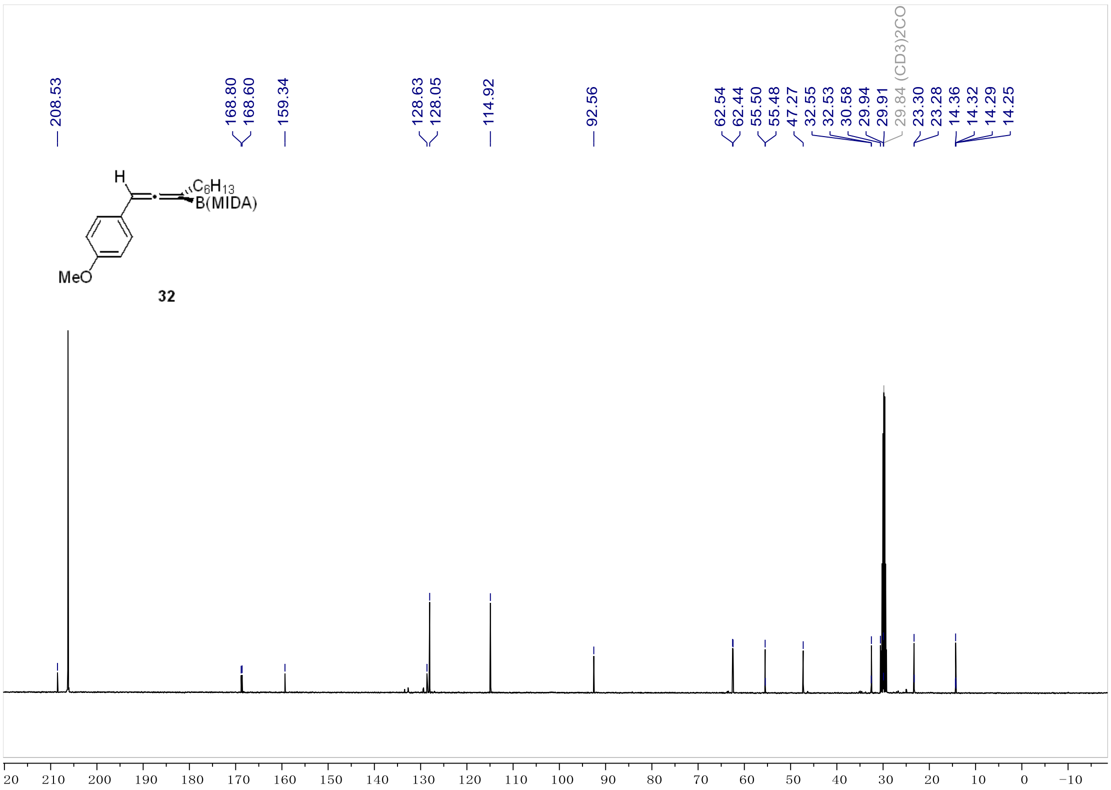

33: ^{1}H NMR (500 MHz, Chloroform-*d*)

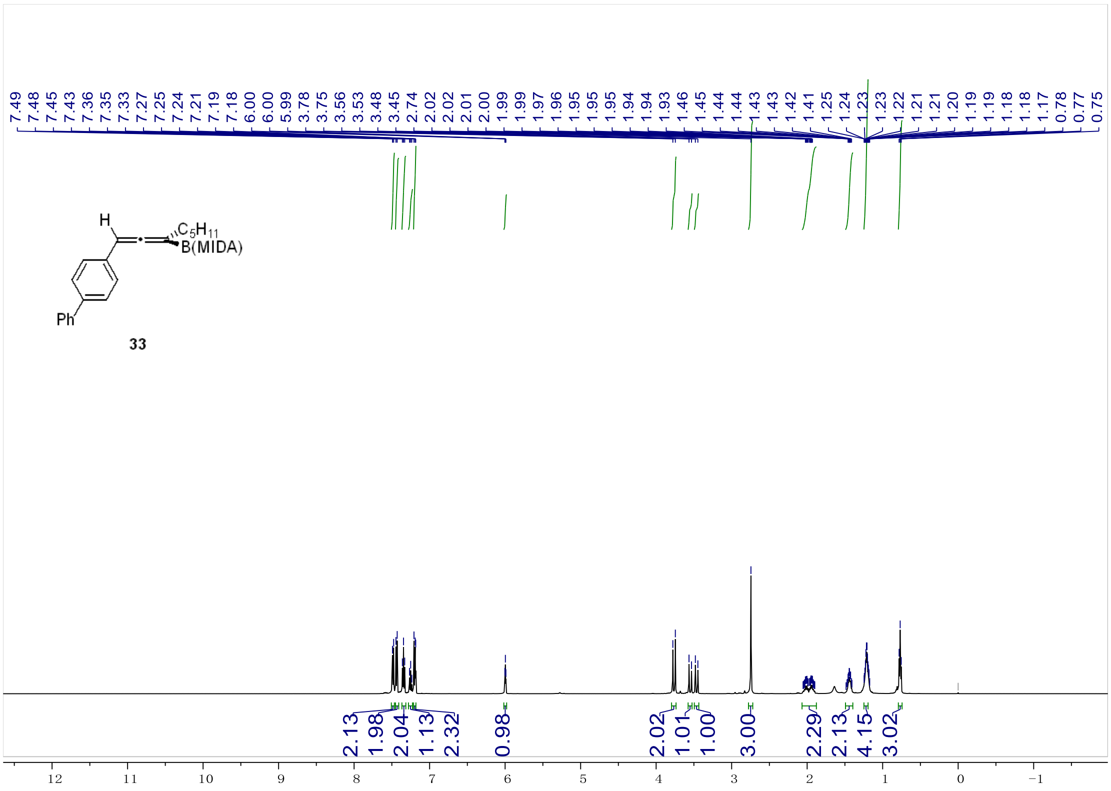

33: ^{13}C NMR (126 MHz, Chloroform-*d*)

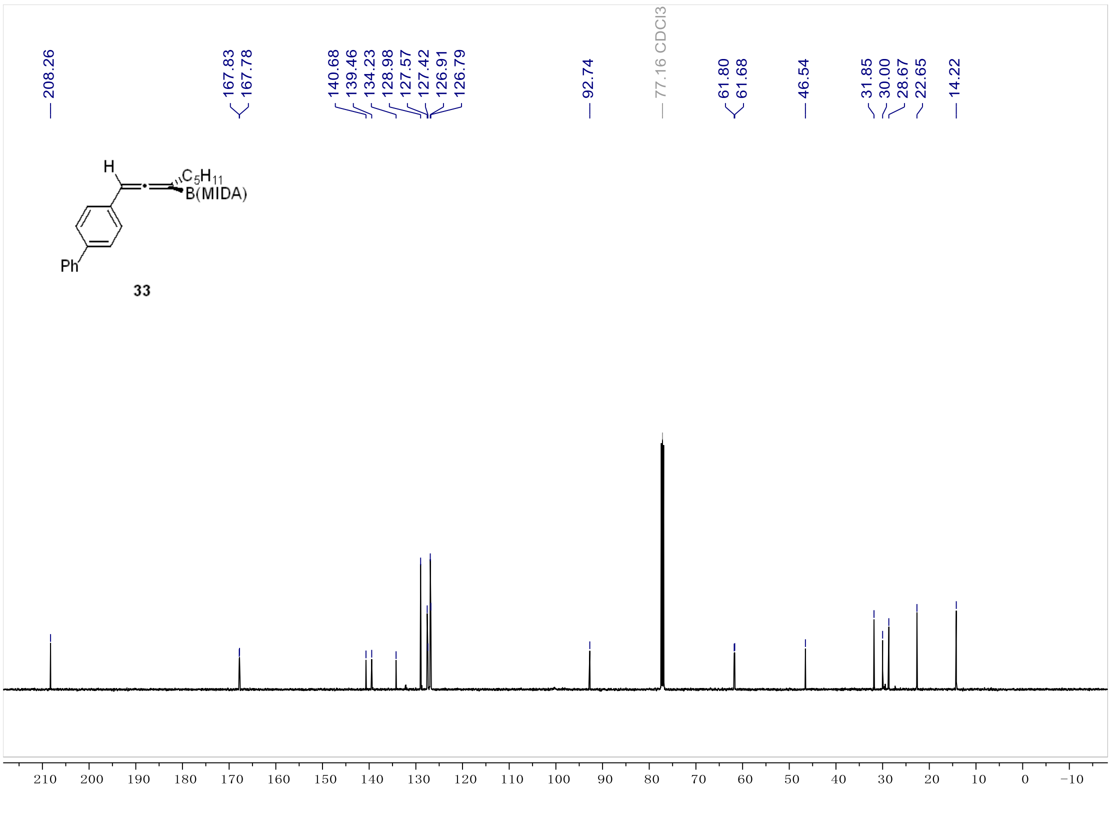

34: ^{1}H NMR (400 MHz, Chloroform-*d*)

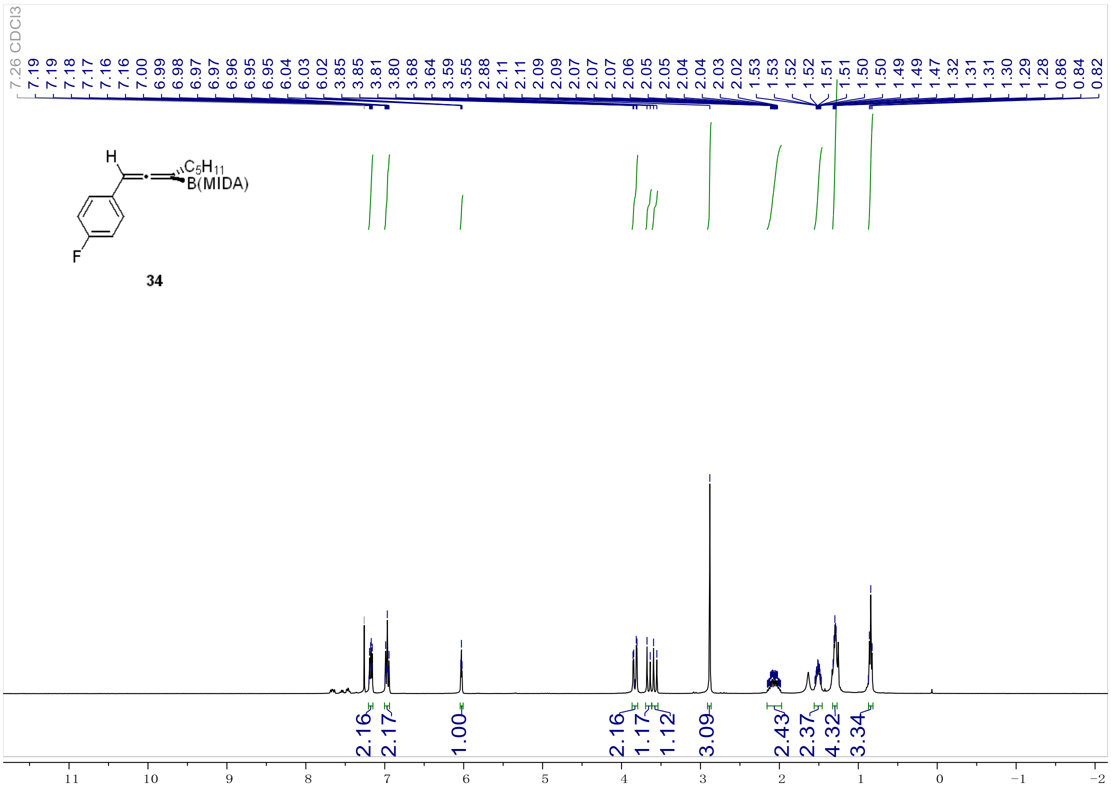

34: ^{13}C NMR (126 MHz, Chloroform-*d*)

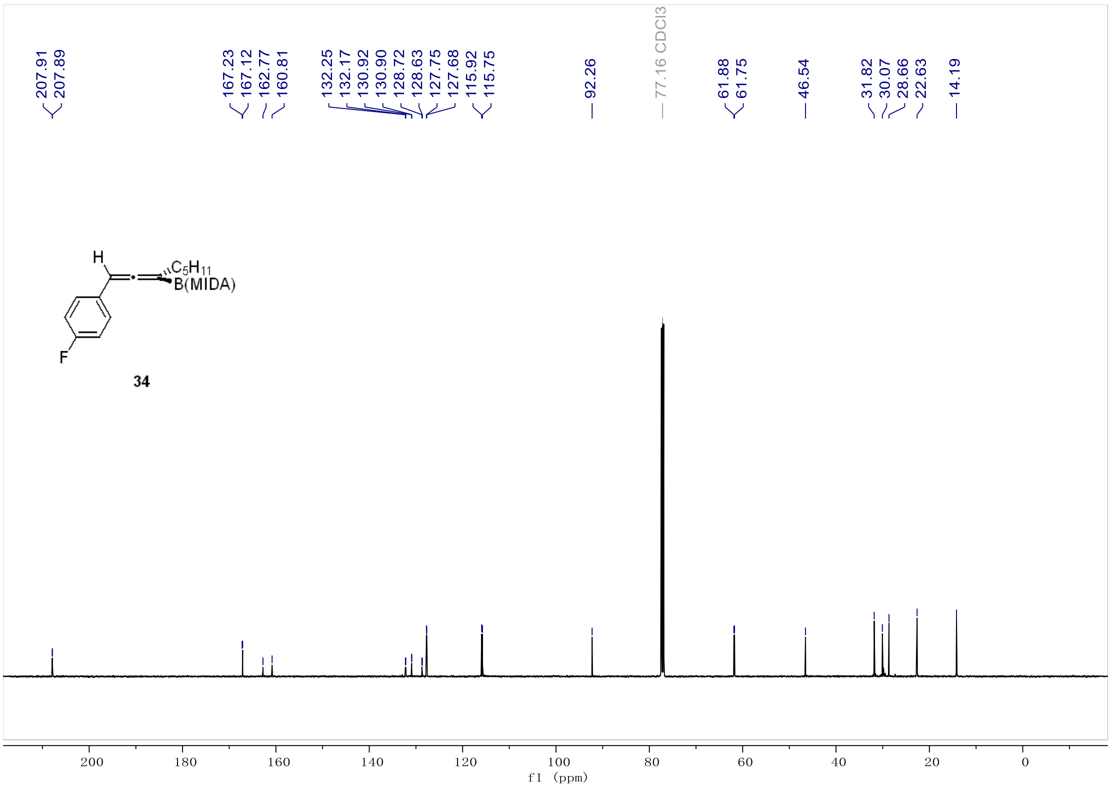

35: ^{1}H NMR (500 MHz, Acetone-d_6)

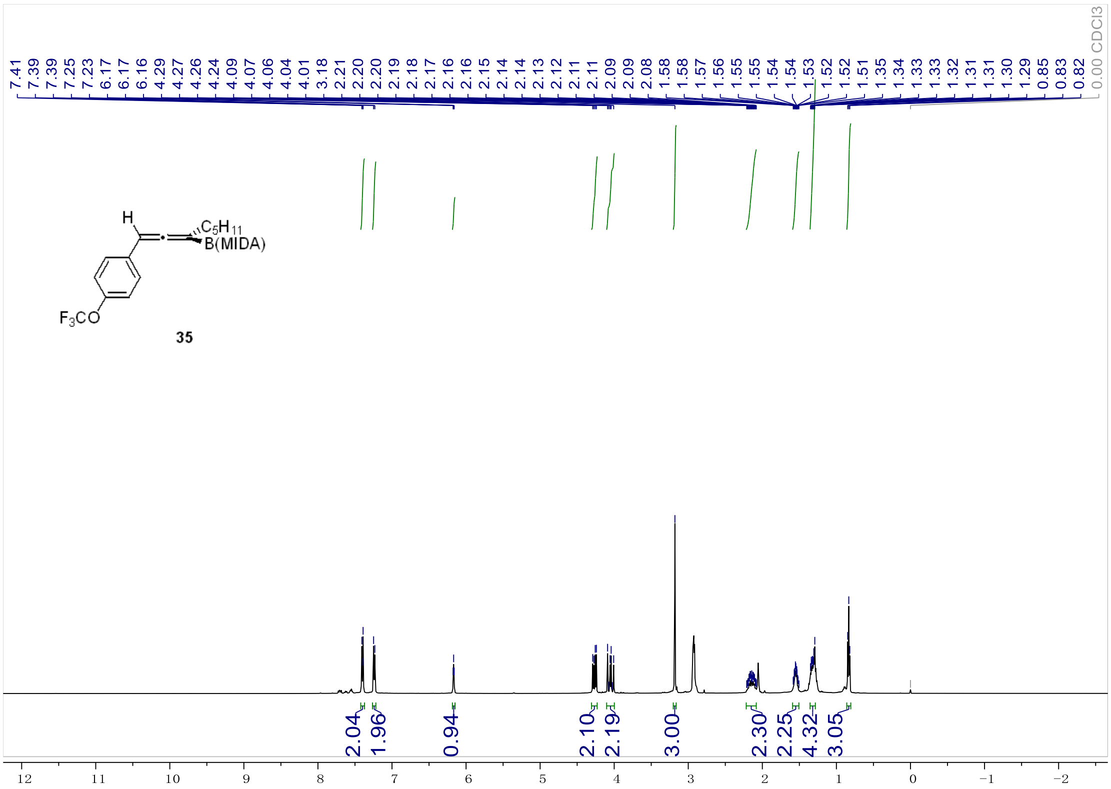

35: ^{13}C NMR (126 MHz, Acetone-d_6)

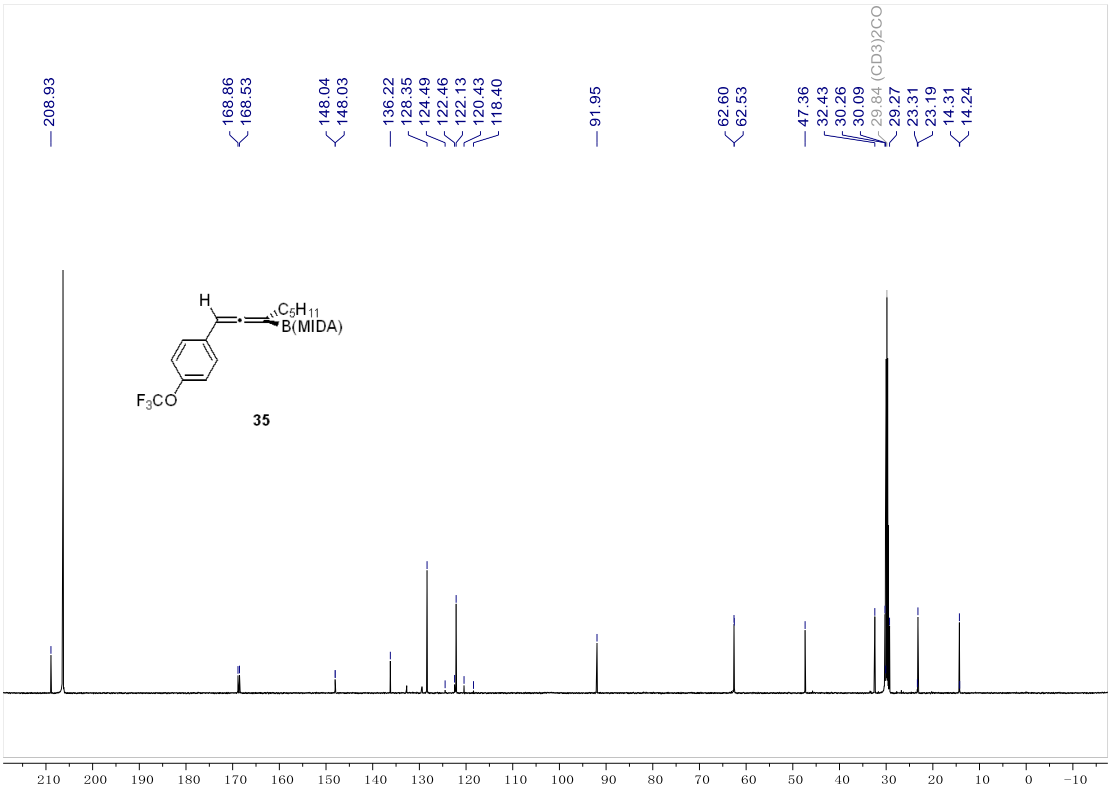

36: ^{1}H NMR (500 MHz, Chloroform-*d*)

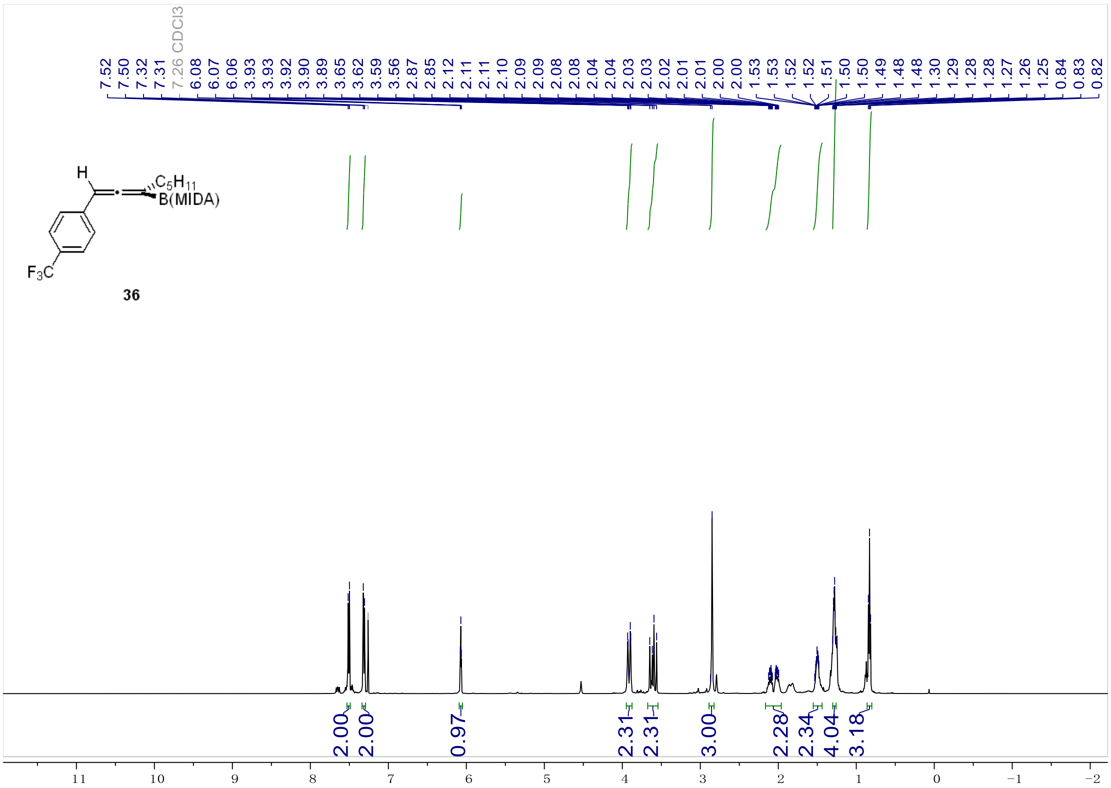

36: ^{13}C NMR (126 MHz, Chloroform-*d*)

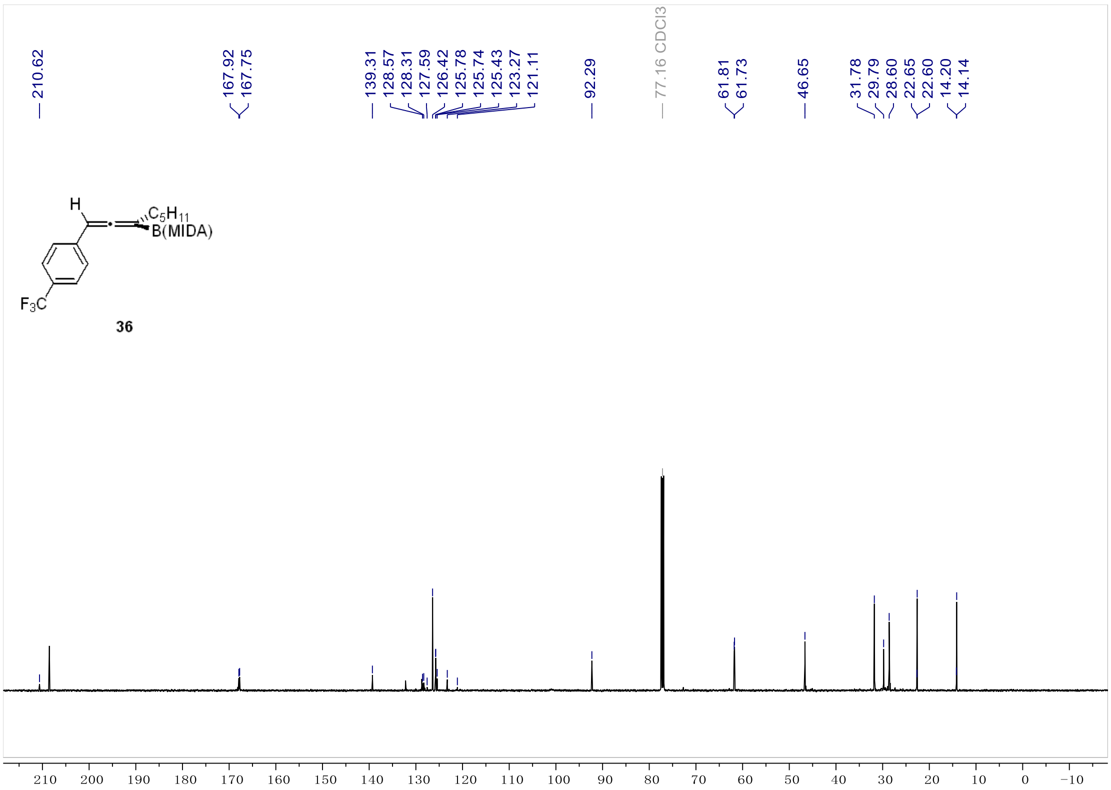

37: ^{1}H NMR (400 MHz, Chloroform-*d*)

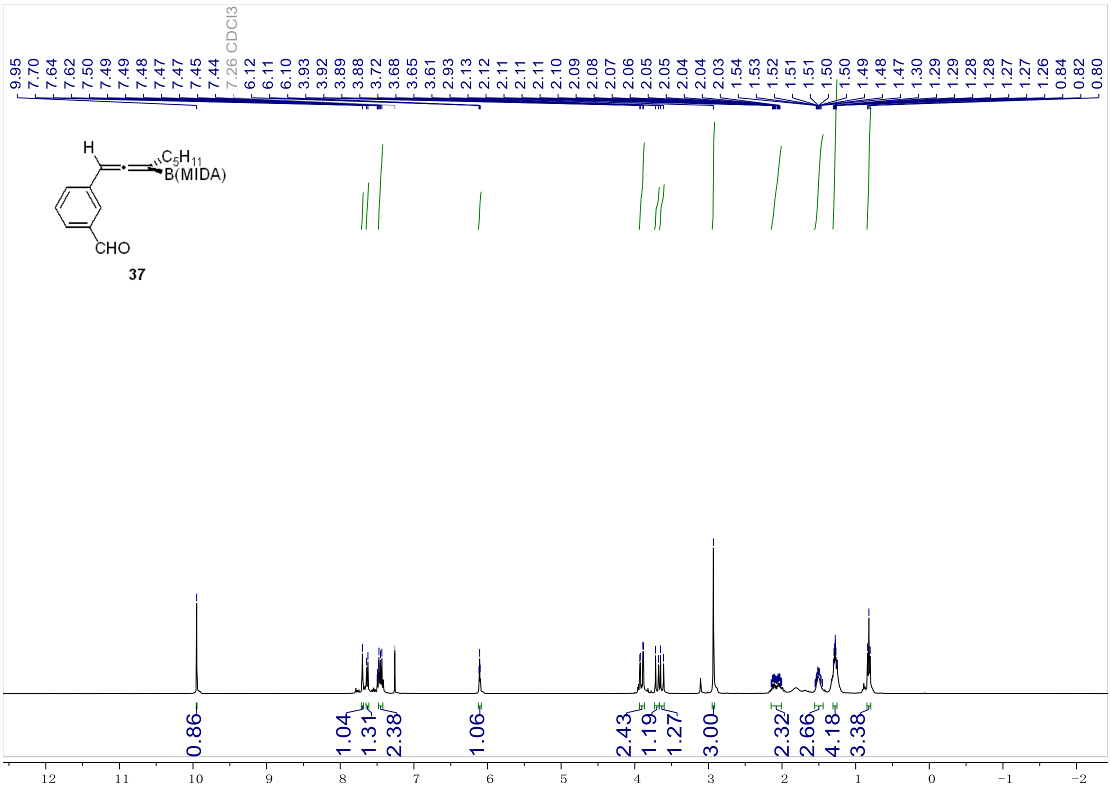

37: ^{13}C NMR (101 MHz, Chloroform-*d*)

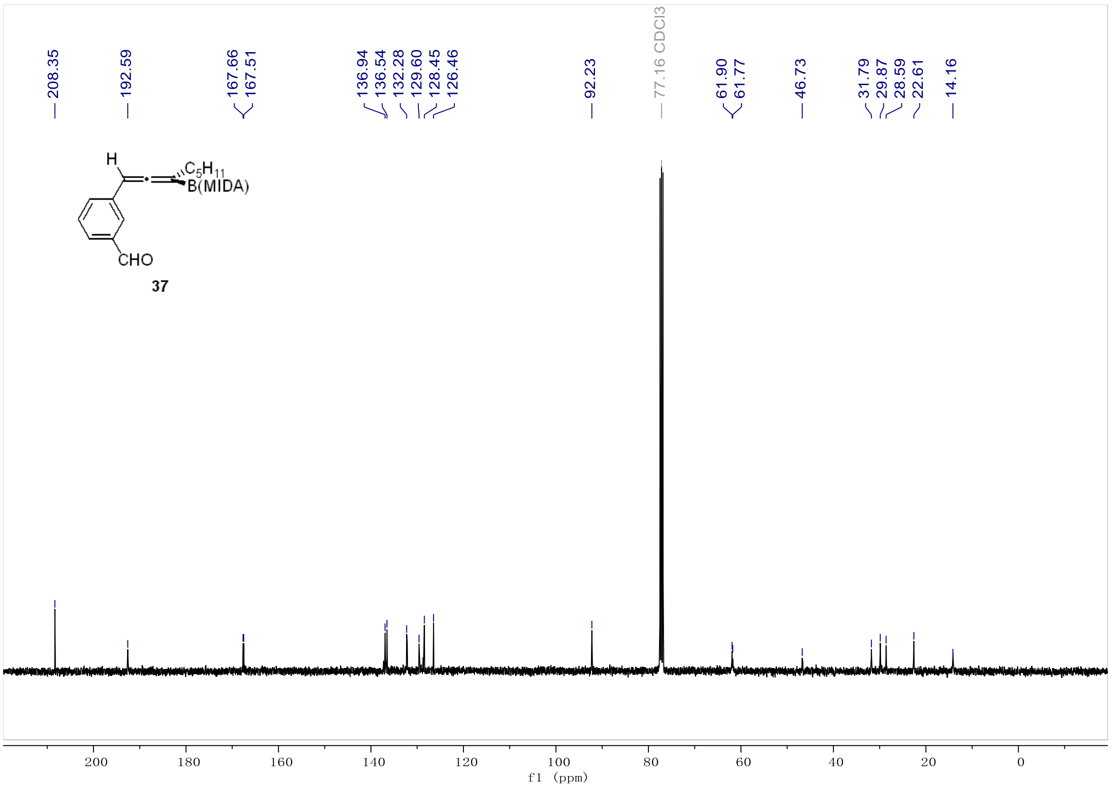

38: ^{1}H NMR (400 MHz, Acetone-d_6)

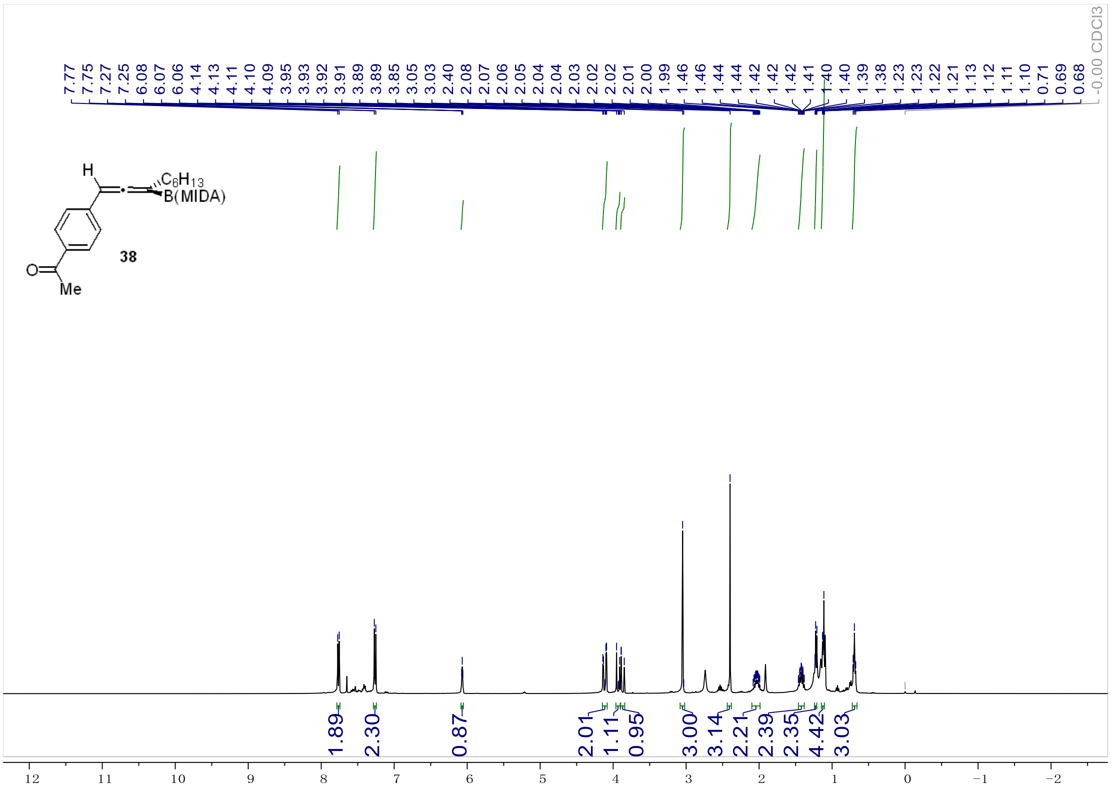

38: ^{13}C NMR (101 MHz, Acetone-d_6)

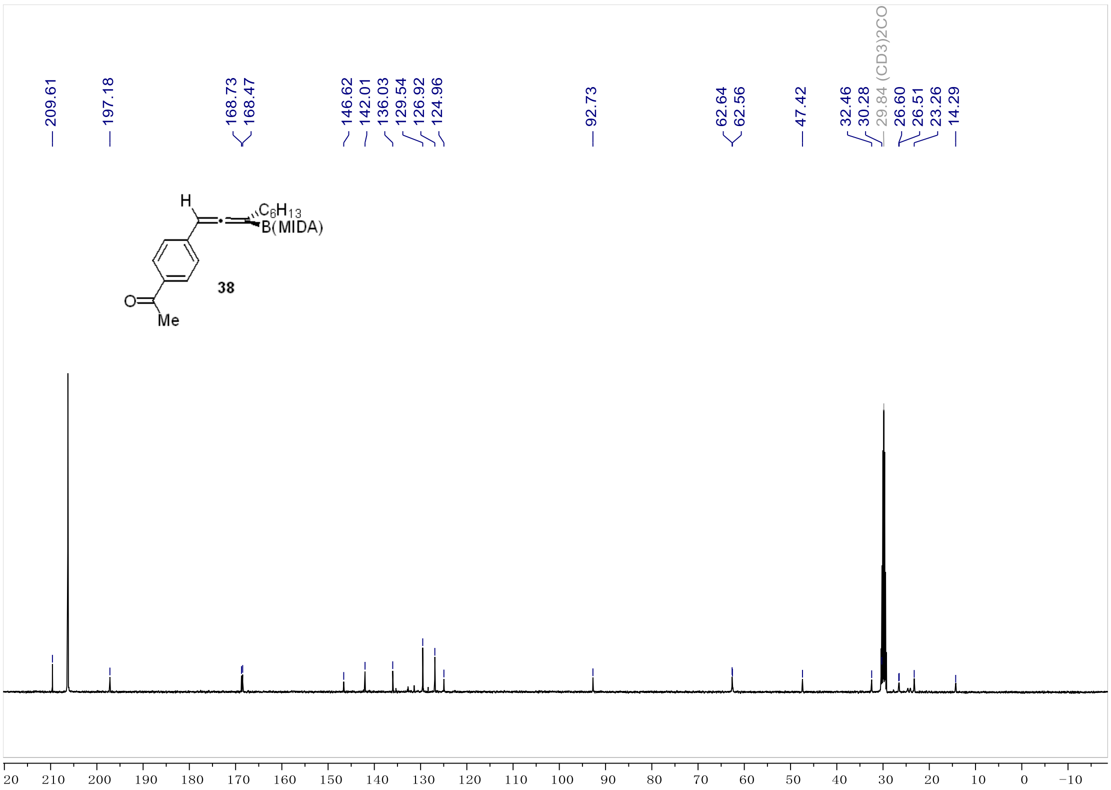

39: ^{1}H NMR (400 MHz, Acetone-d6)

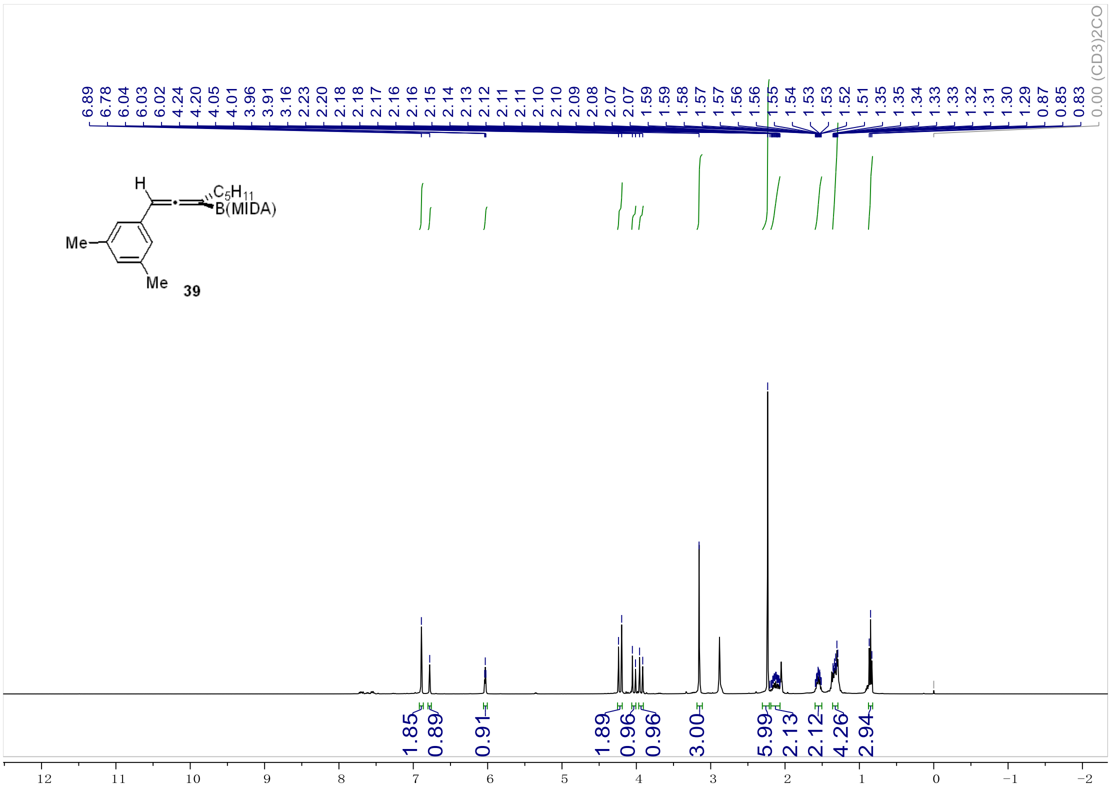

39: ^{13}C NMR (126 MHz, Acetone-*d_6*)

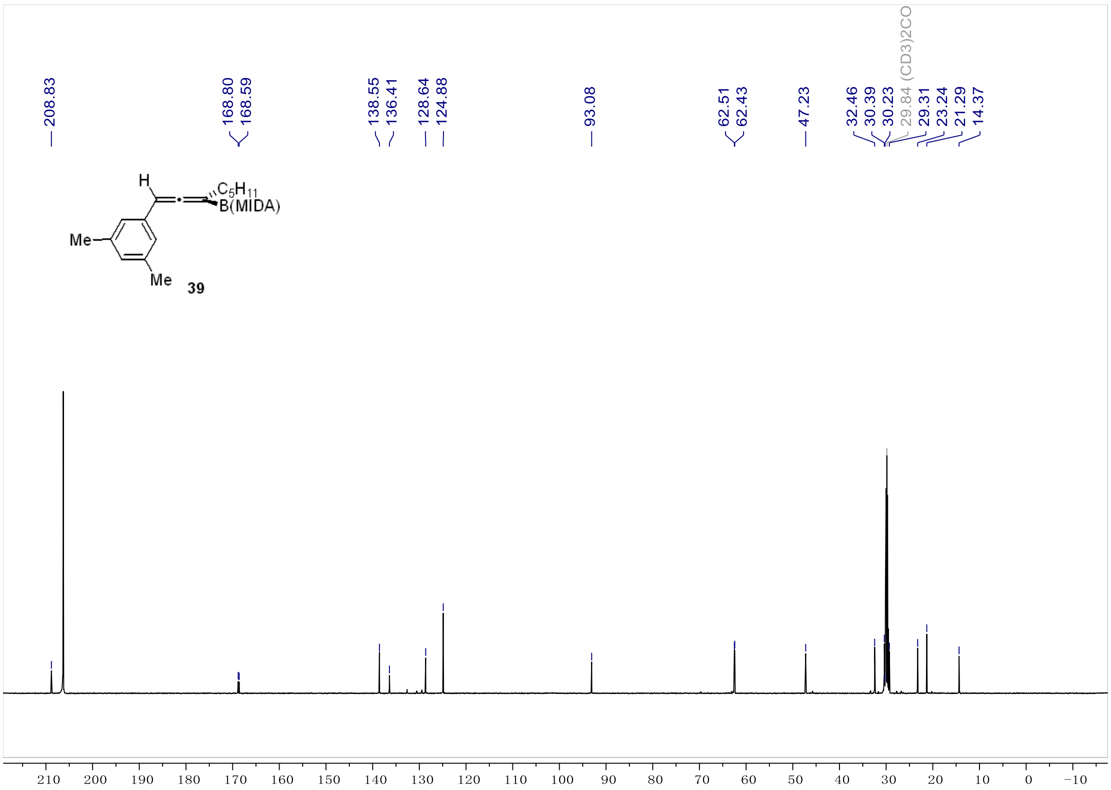

40: ^{1}H NMR (400 MHz, Chloroform-*d*)

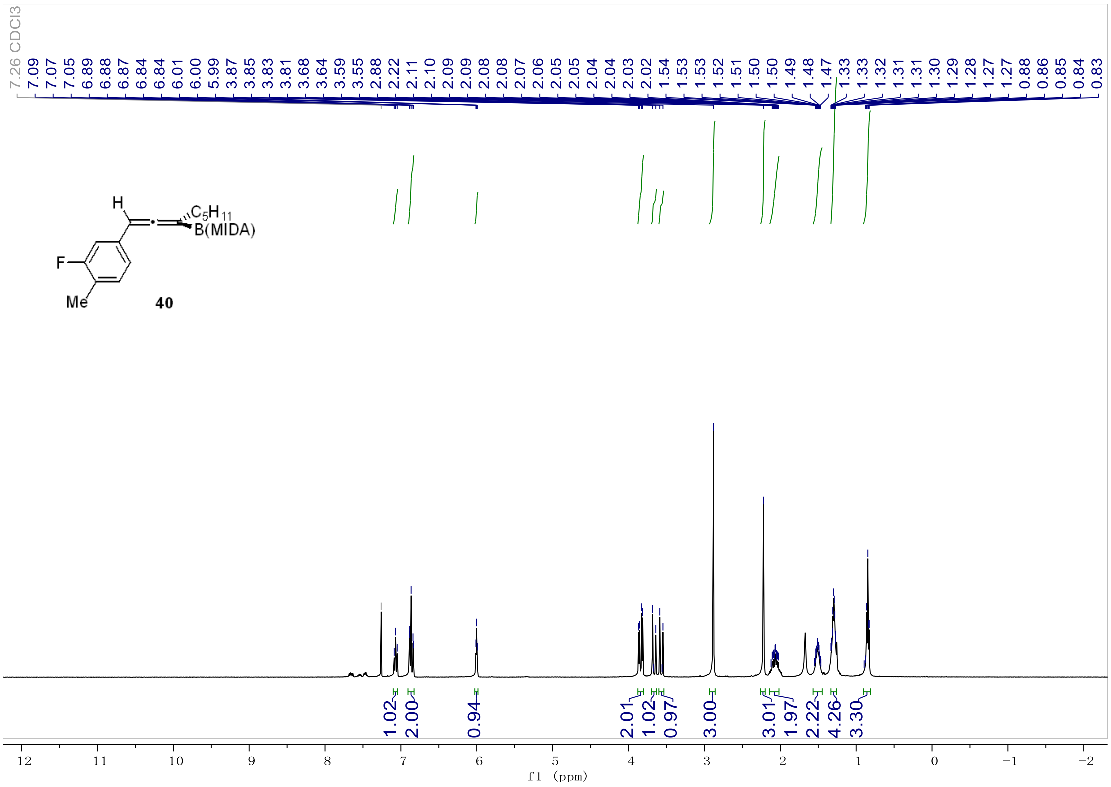

40: ^{13}C NMR (101 MHz, Chloroform-*d*)

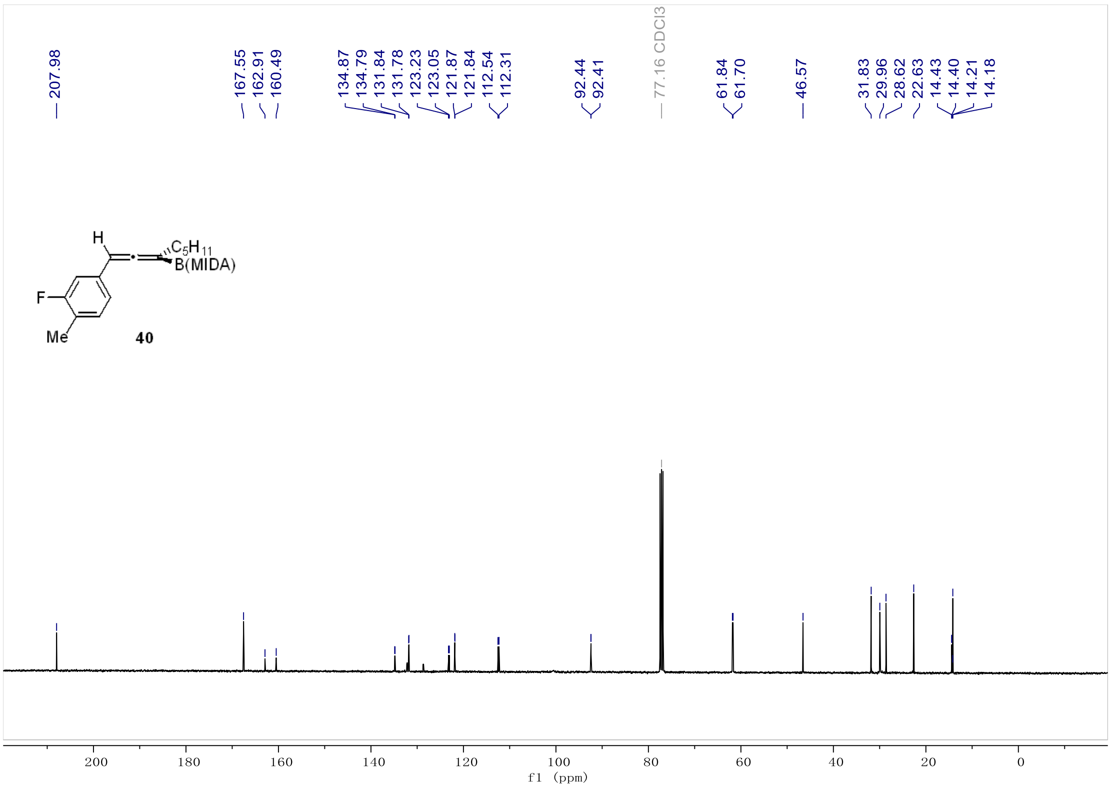

41: ¹H NMR (500 MHz, Chloroform-*d*)

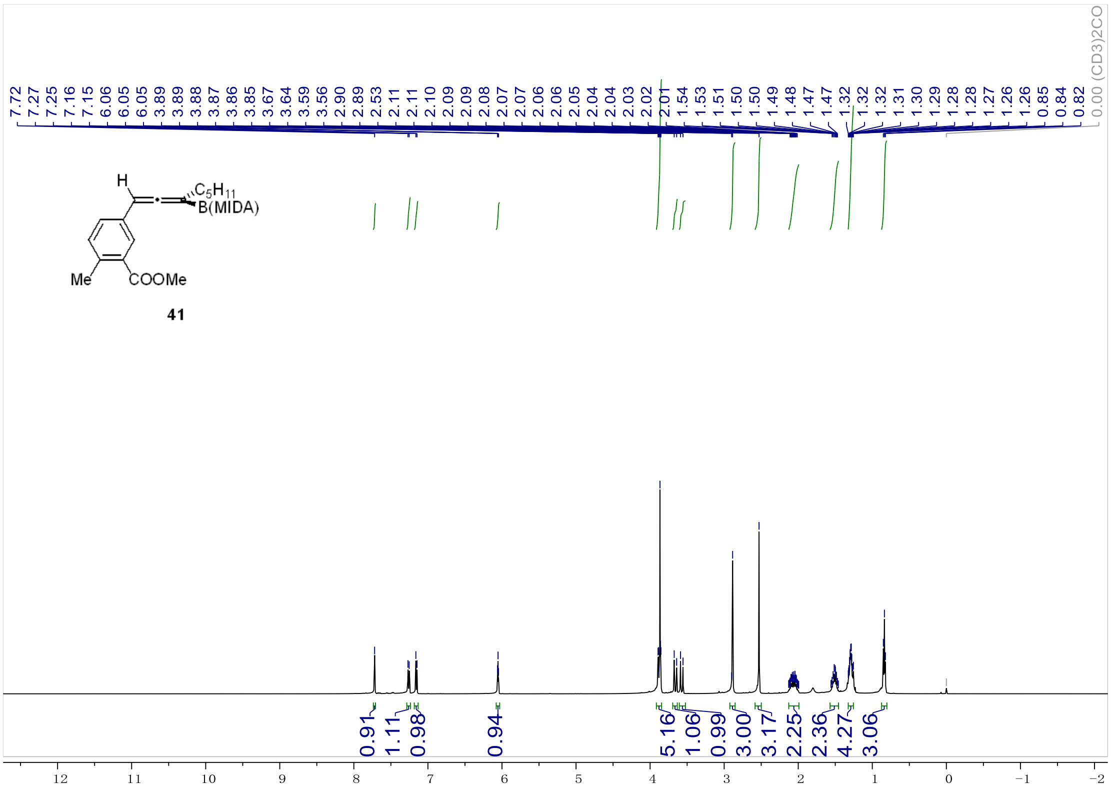

41: ¹³C NMR (126 MHz, Chloroform-*d*)

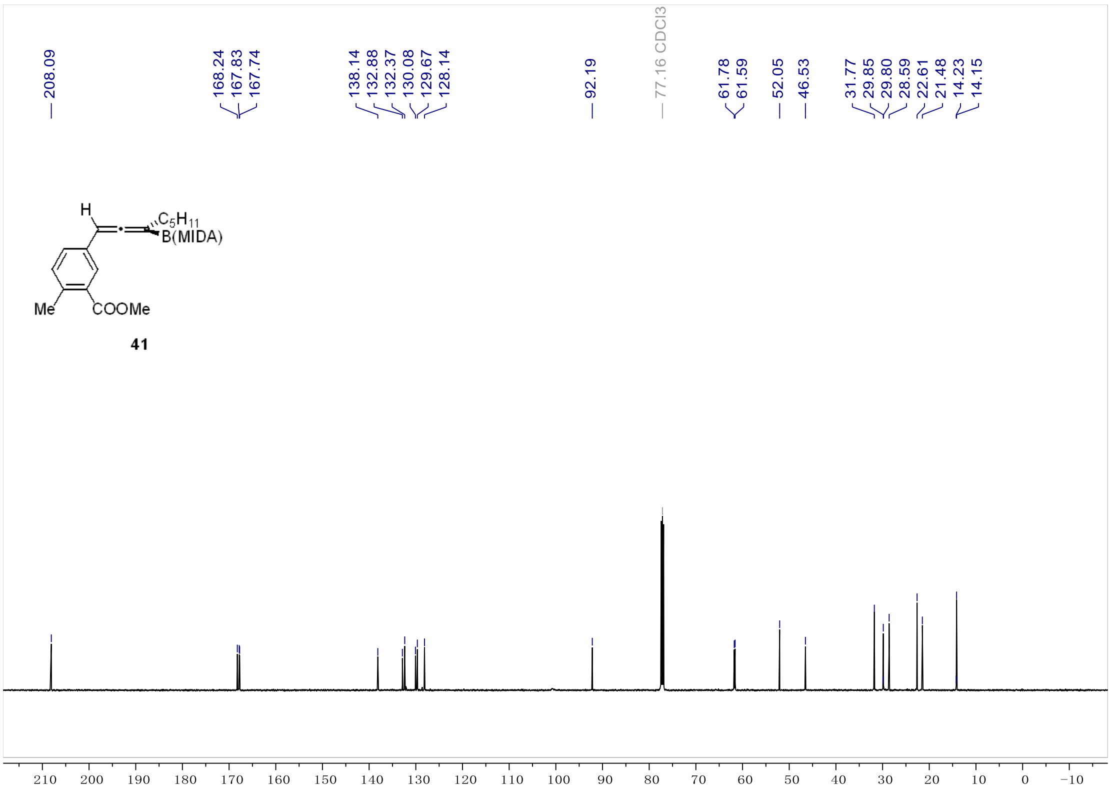

42: ^{1}H NMR (400 MHz, Chloroform-*d*)

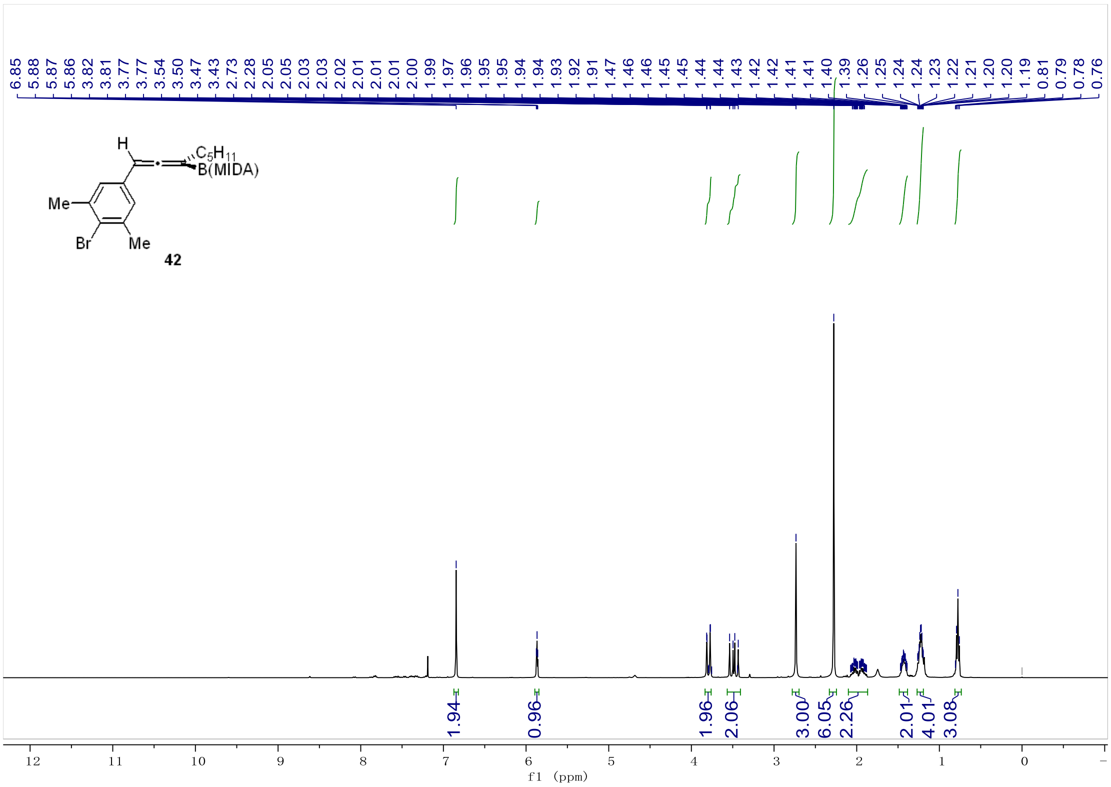

42: ^{13}C NMR (126 MHz, Chloroform-*d*)

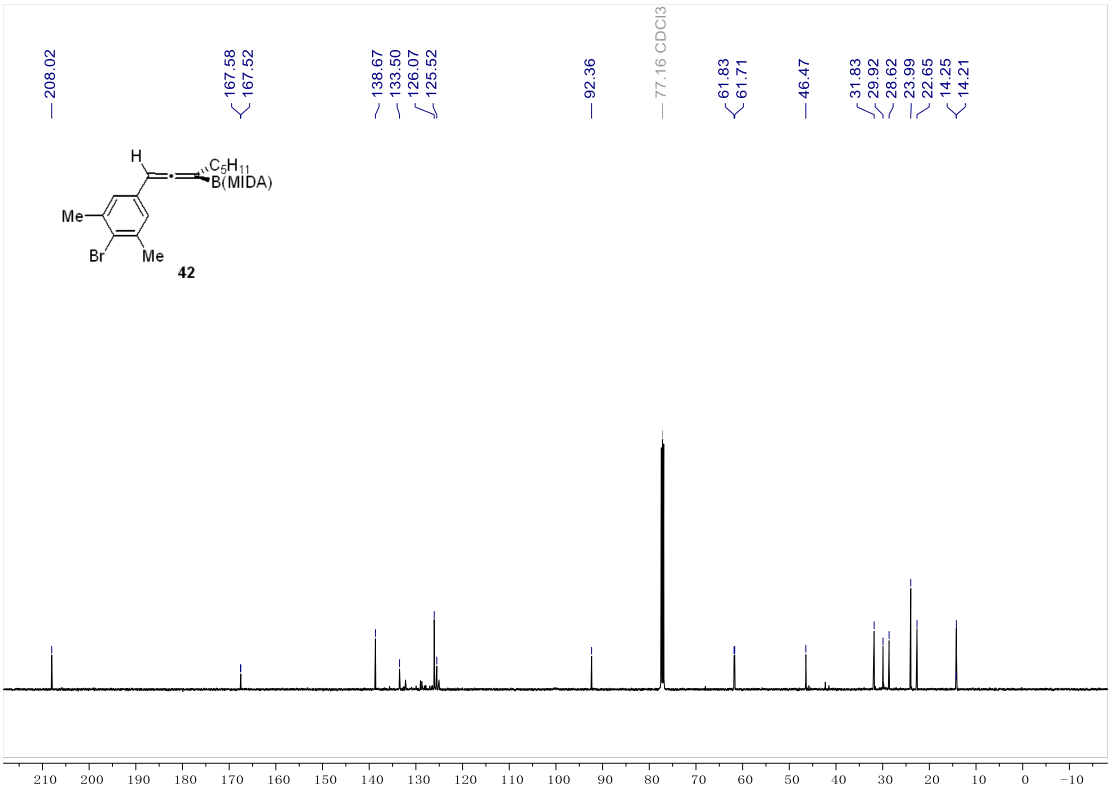

43: ^{1}H NMR (400 MHz, Acetone-d_6)

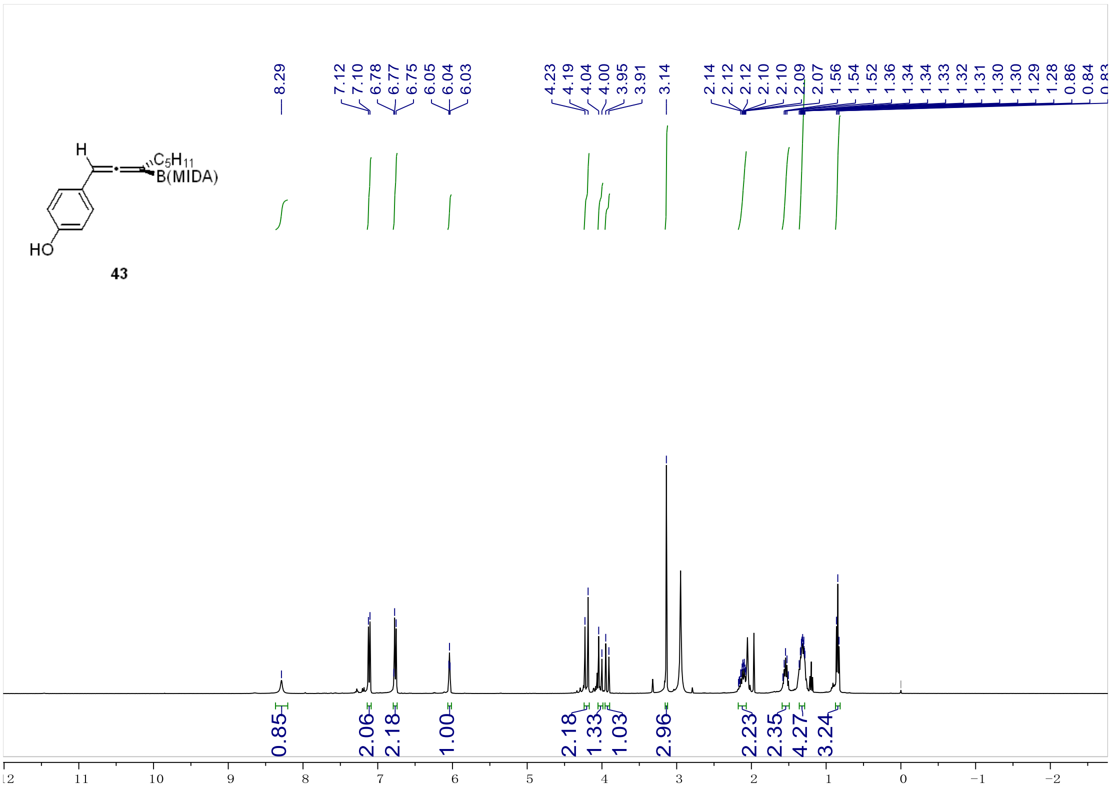

43: ^{13}C NMR (101 MHz, Acetone-d_6)

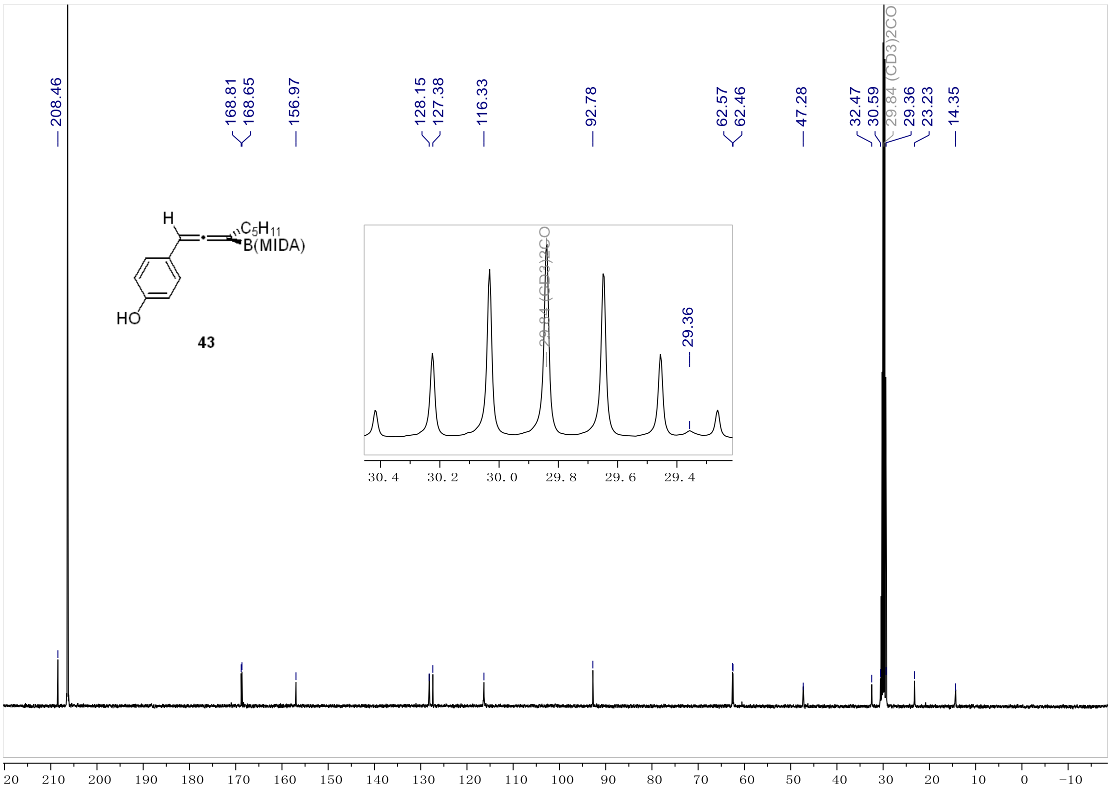

44: ^{1}H NMR (400 MHz, Chloroform-*d*)

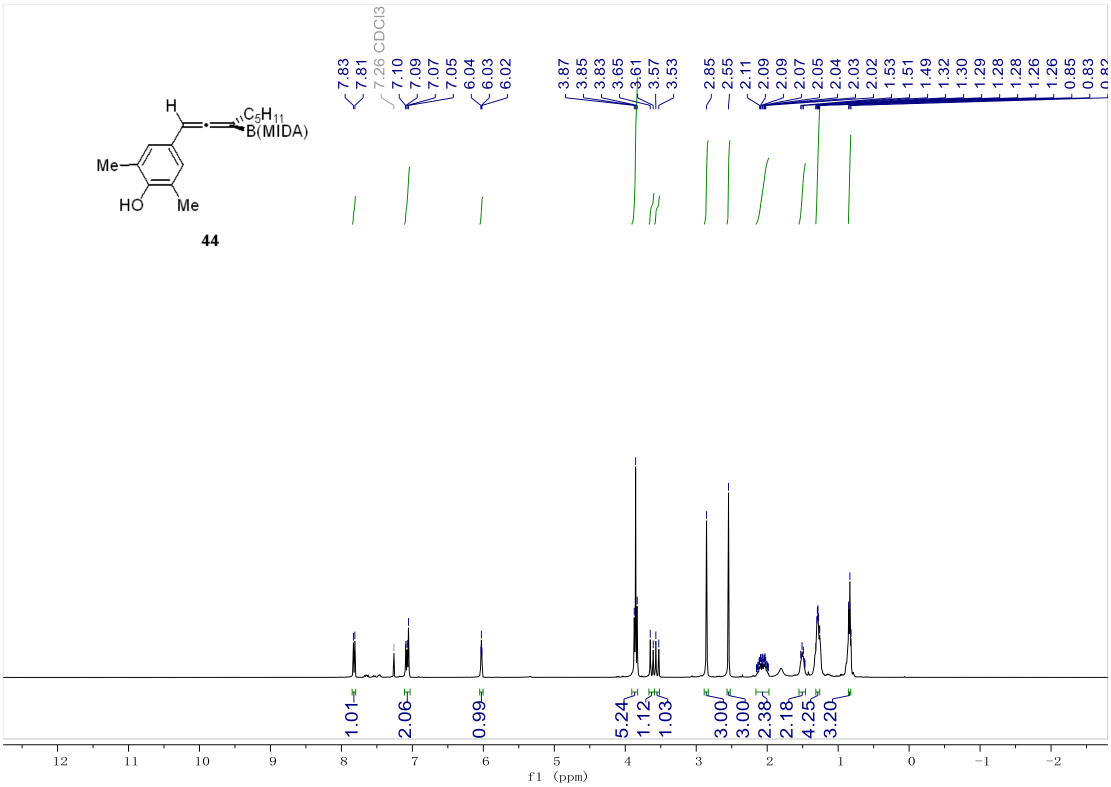

44: ^{13}C NMR (101 MHz, Chloroform-*d*)

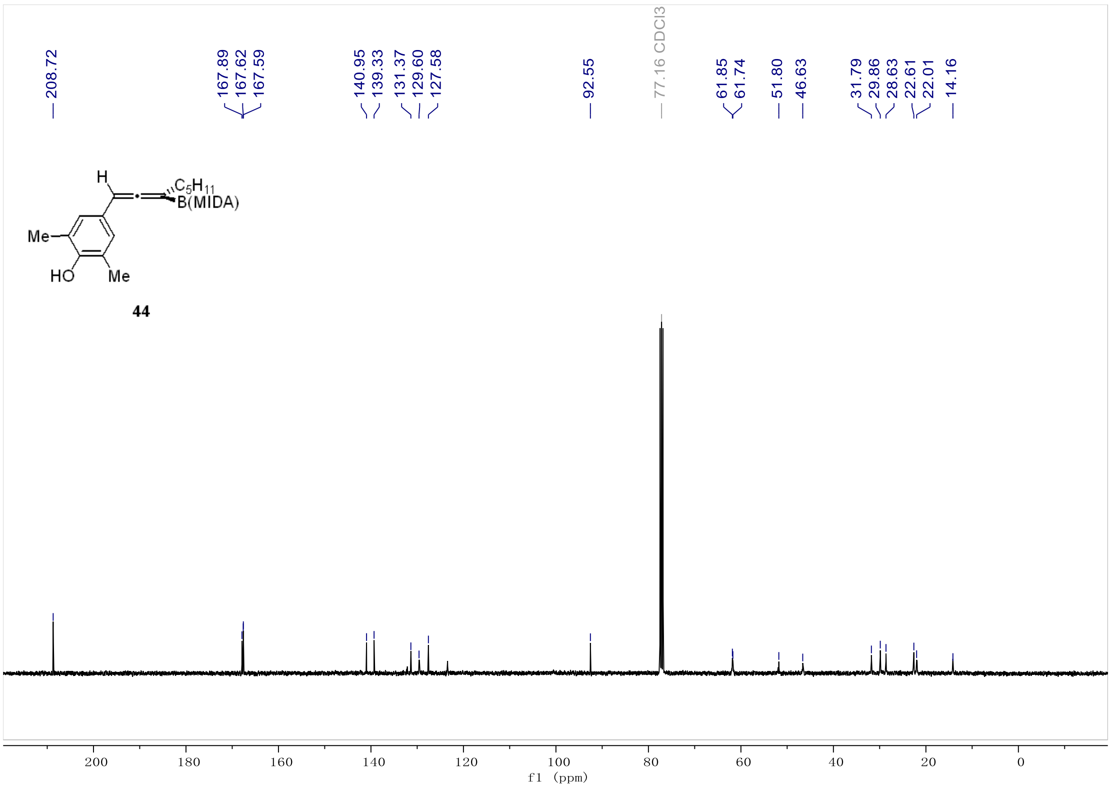

45: ^{1}H NMR (500 MHz, Chloroform-*d*)

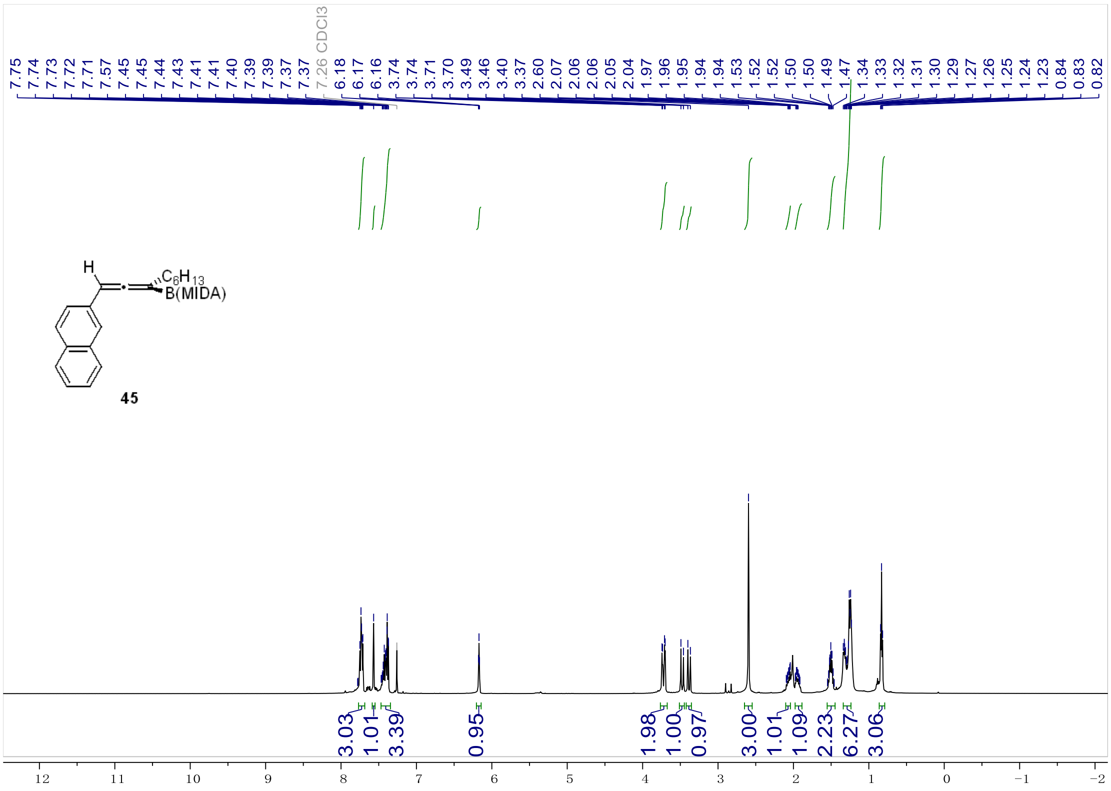

45: ^{13}C NMR (126 MHz, Chloroform-*d*)

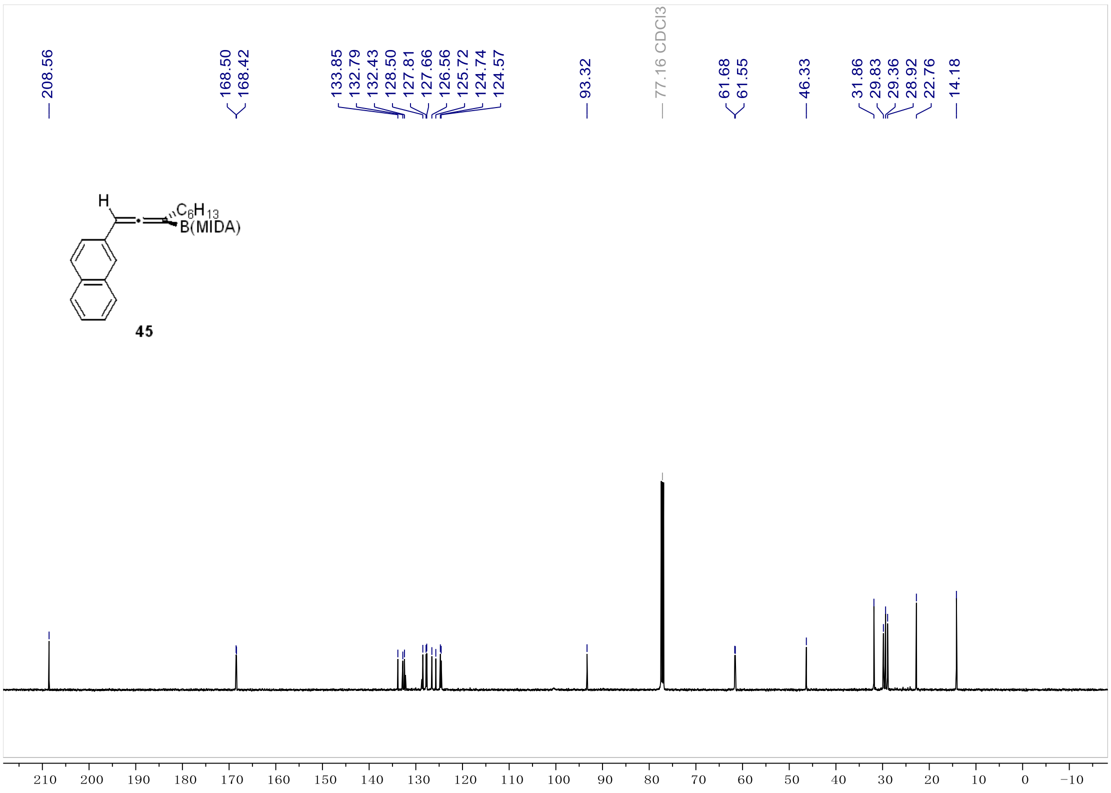

46: ^{1}H NMR (400 MHz, Acetone-d_6)

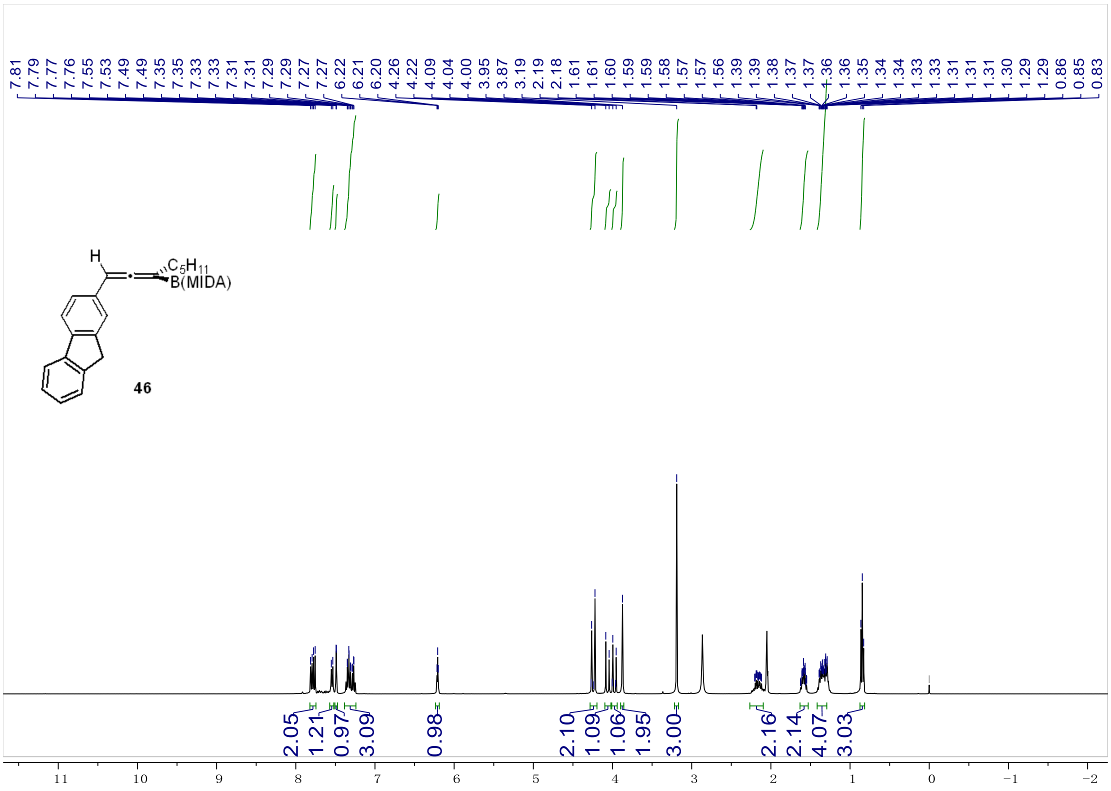

46: ^{13}C NMR (101 MHz, Acetone-d_6)

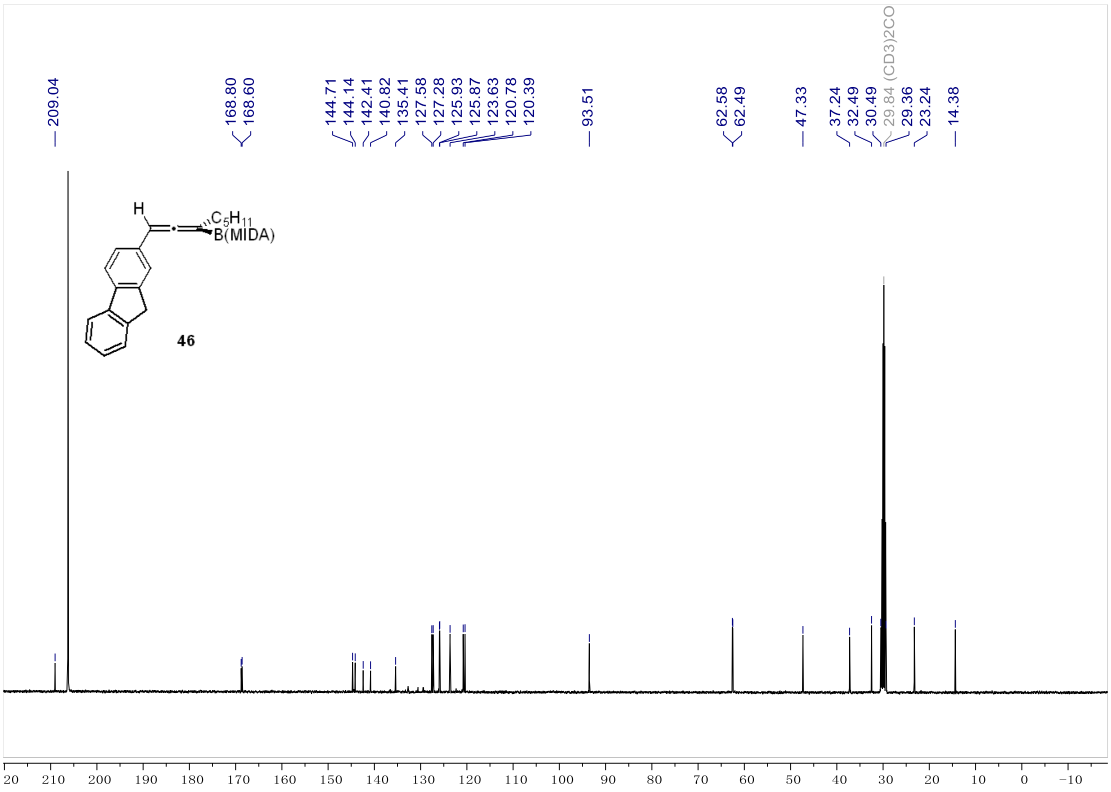

47: ^{1}H NMR (400 MHz, Acetone-d_6)

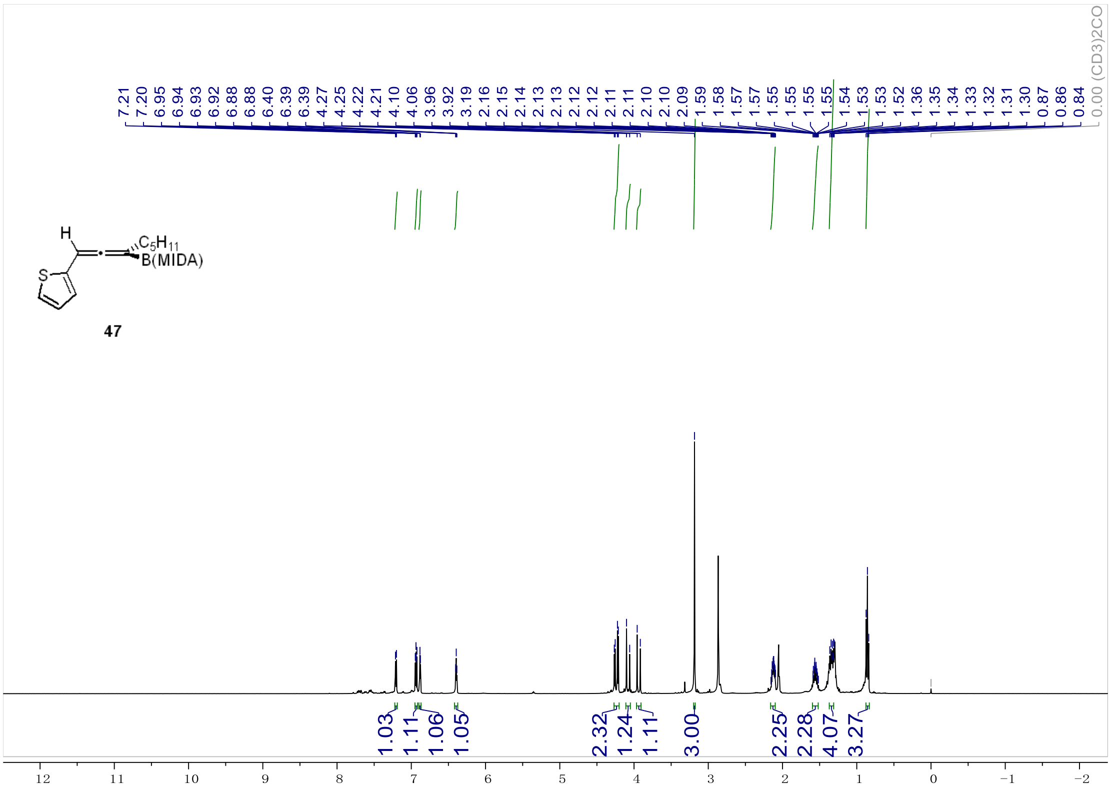

47: ^{13}C NMR (126 MHz, Acetone-d_6)

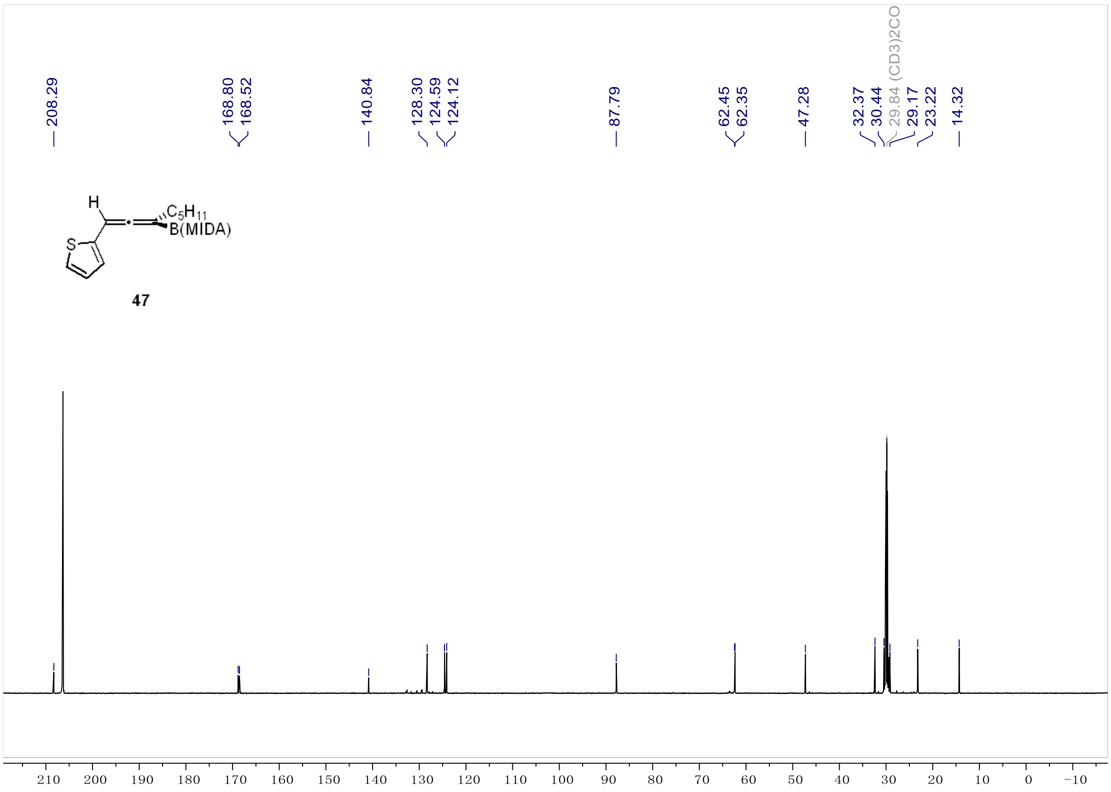

48: ^{1}H NMR (400 MHz, Chloroform-*d*)

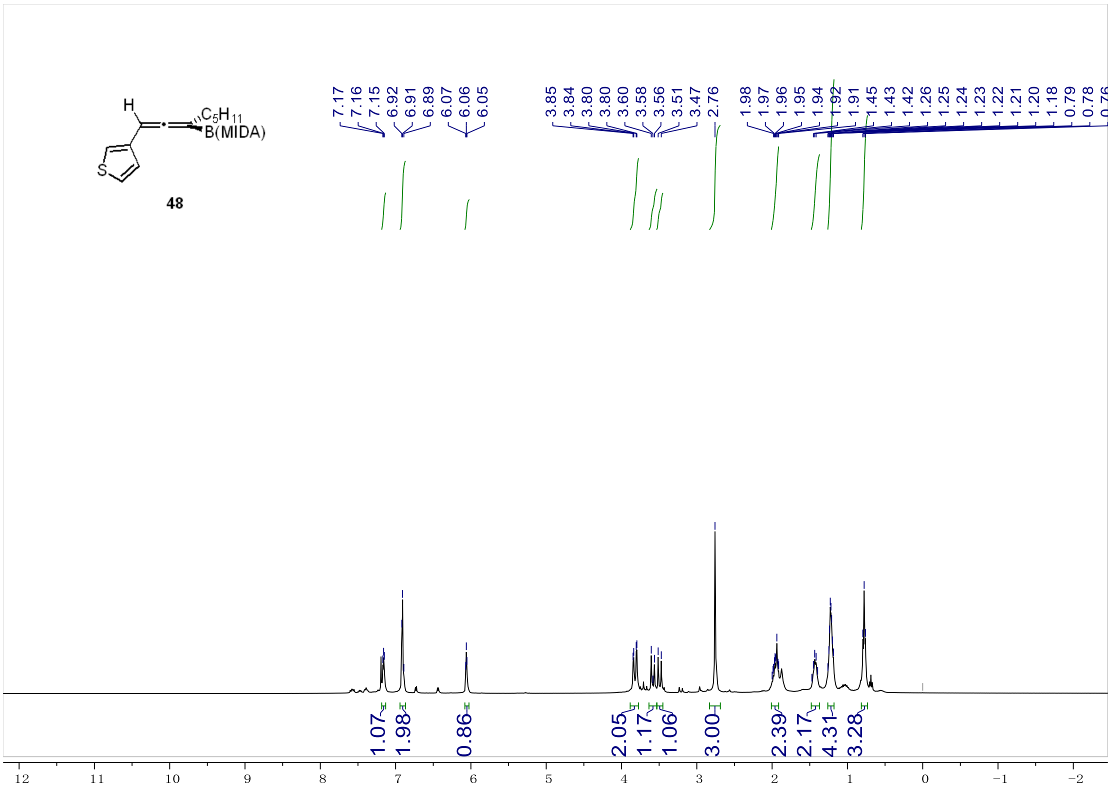

48: ^{13}C NMR (101 MHz, Chloroform-*d*)

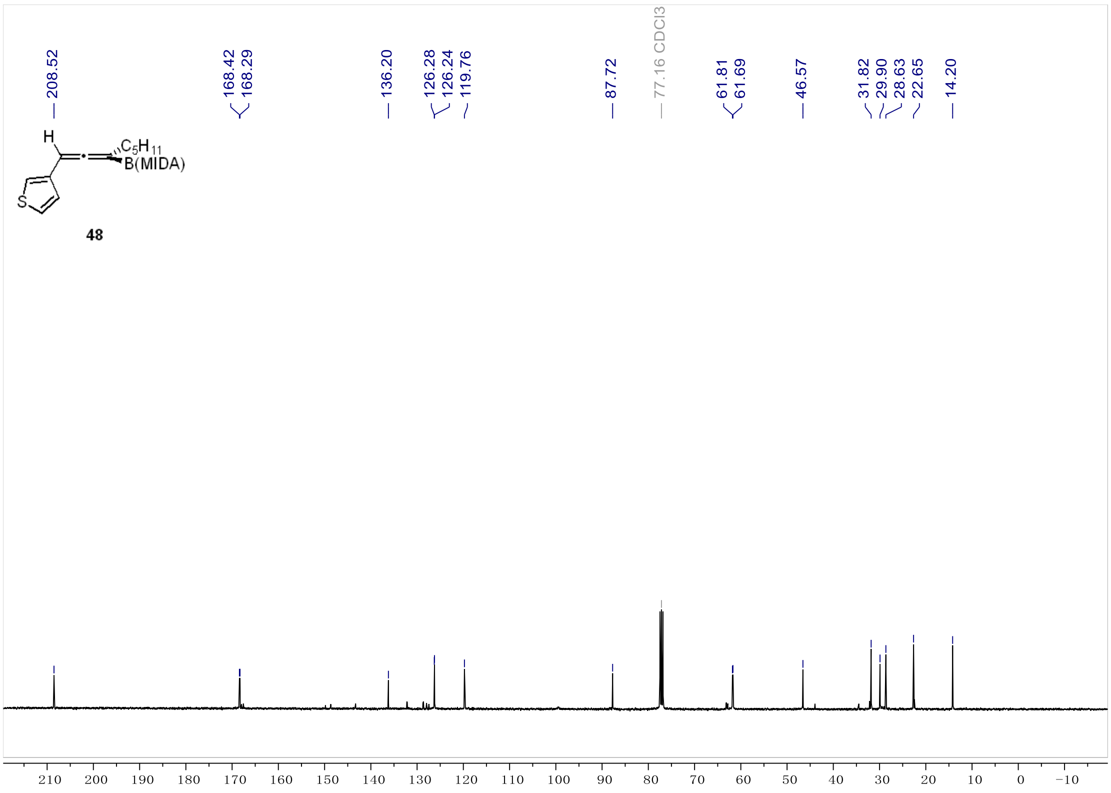

49: ^{1}H NMR (400 MHz, Chloroform-*d*)

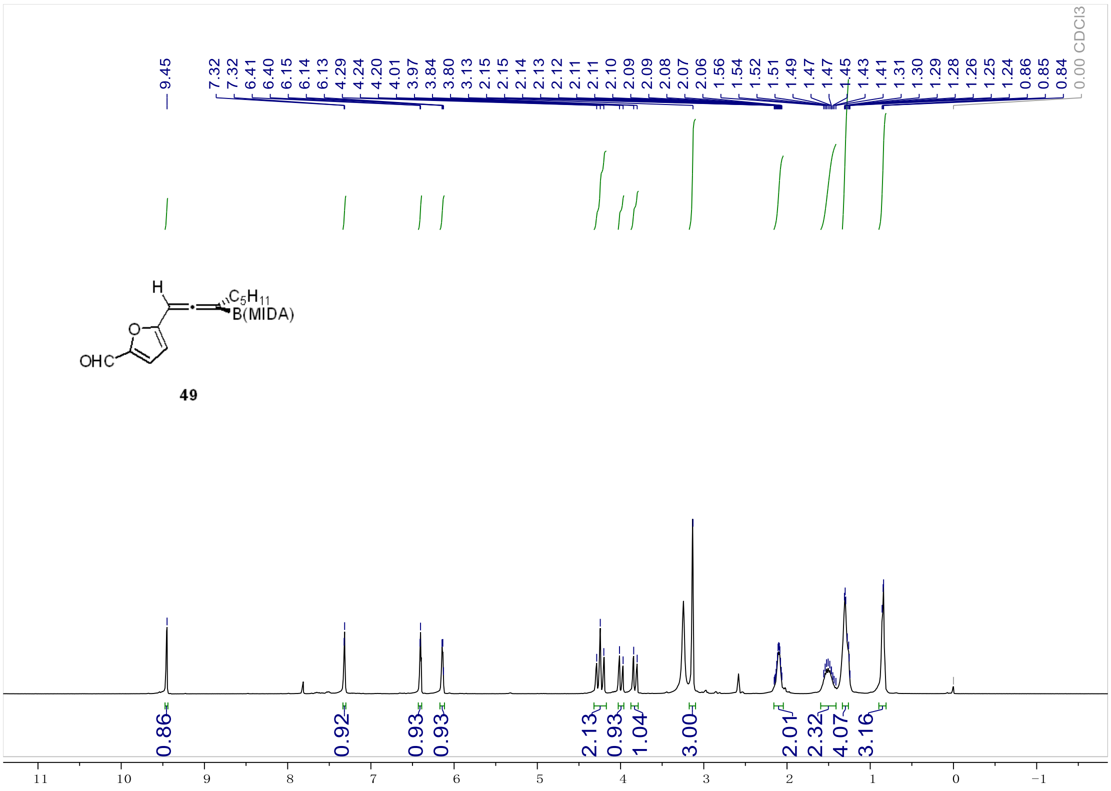

49: ^{13}C NMR (126 MHz, Chloroform-*d*)

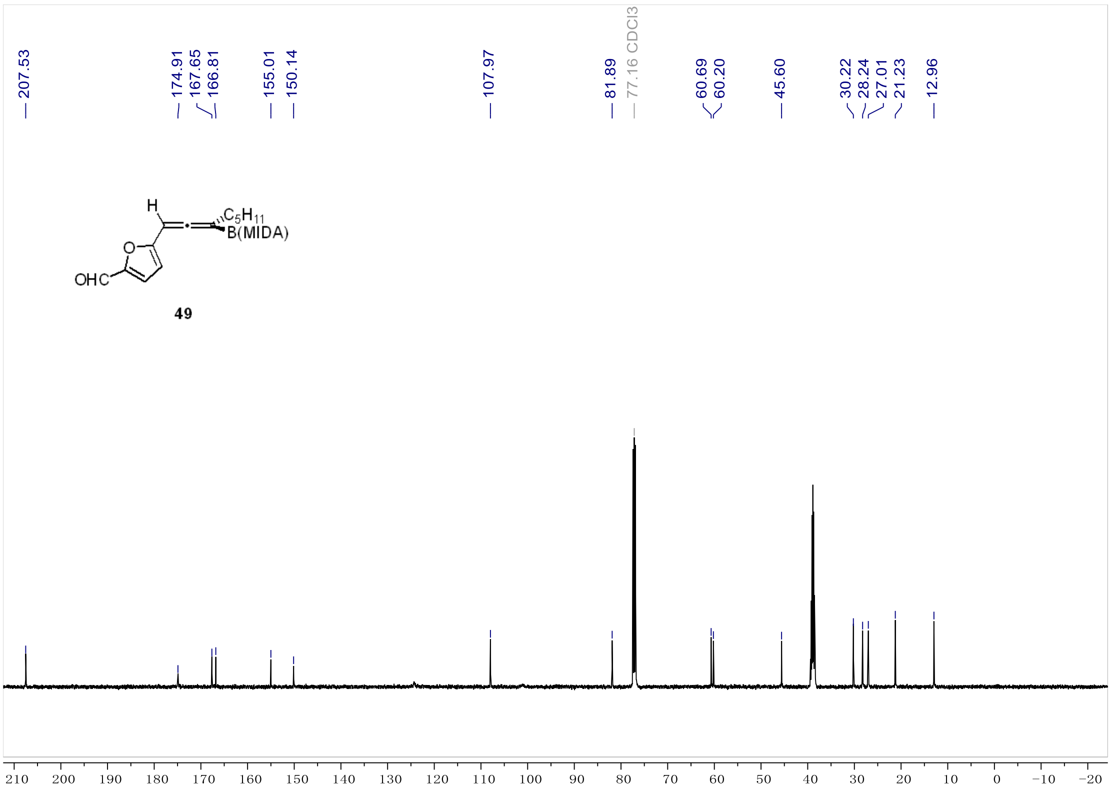

50: ^{1}H NMR (400 MHz, Acetone-d_6)

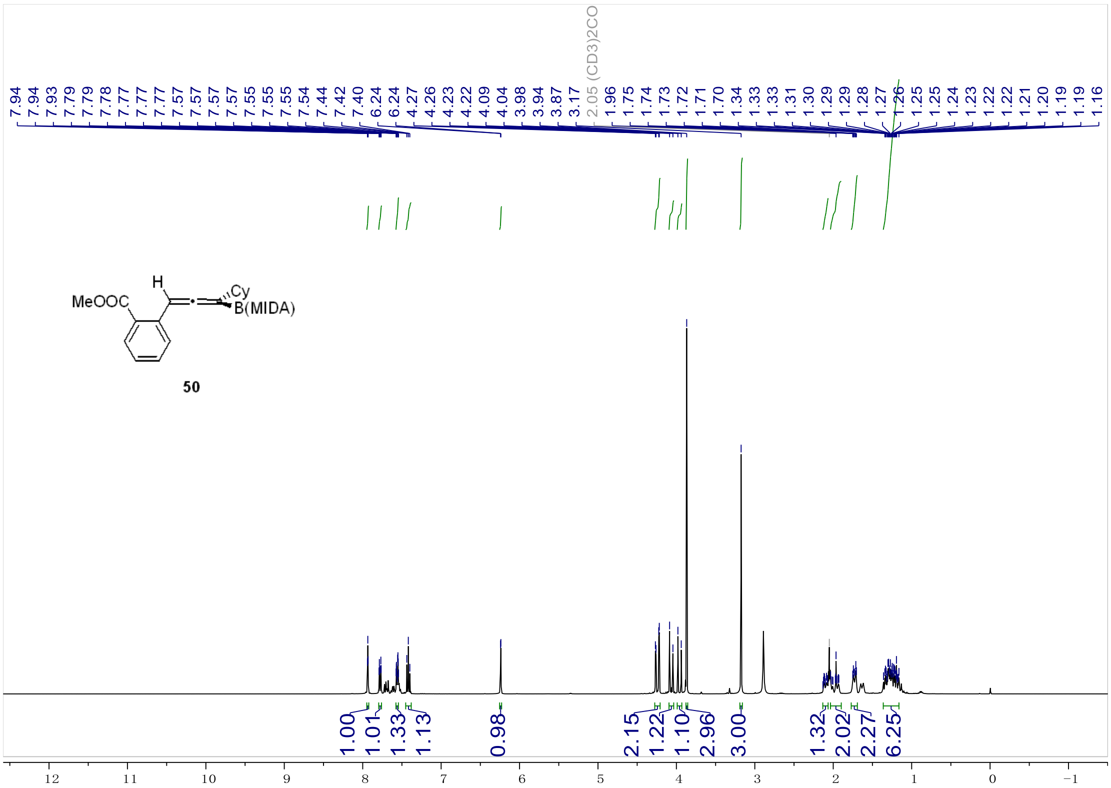

50: ^{13}C NMR (126 MHz, Acetone-d$_6$)

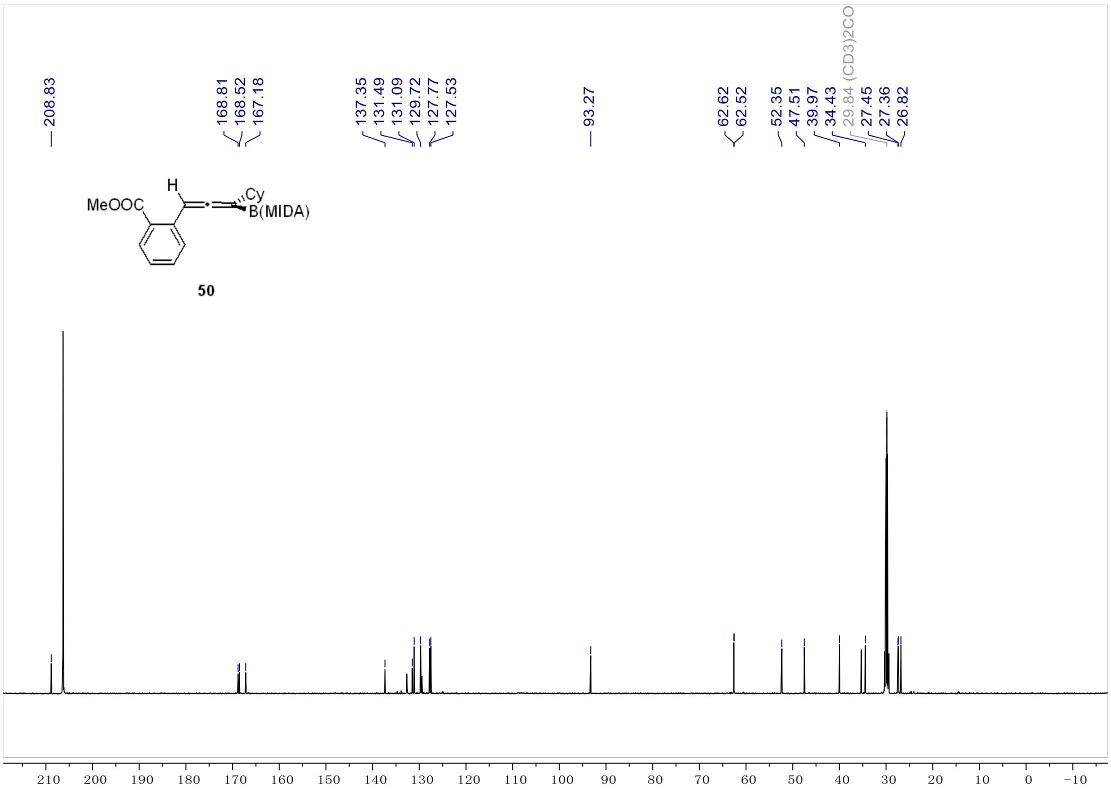

51: ^{1}H NMR (400 MHz, Chloroform-*d*)

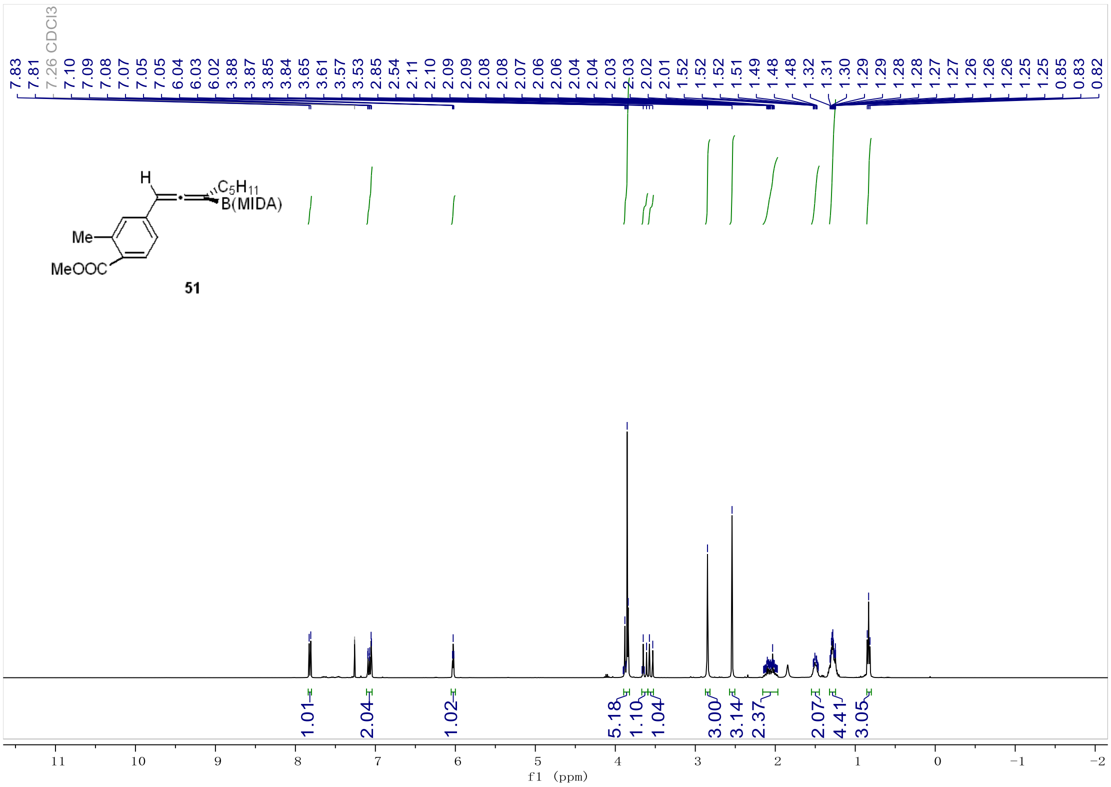

51: ^{13}C NMR (126 MHz, Chloroform-*d*)

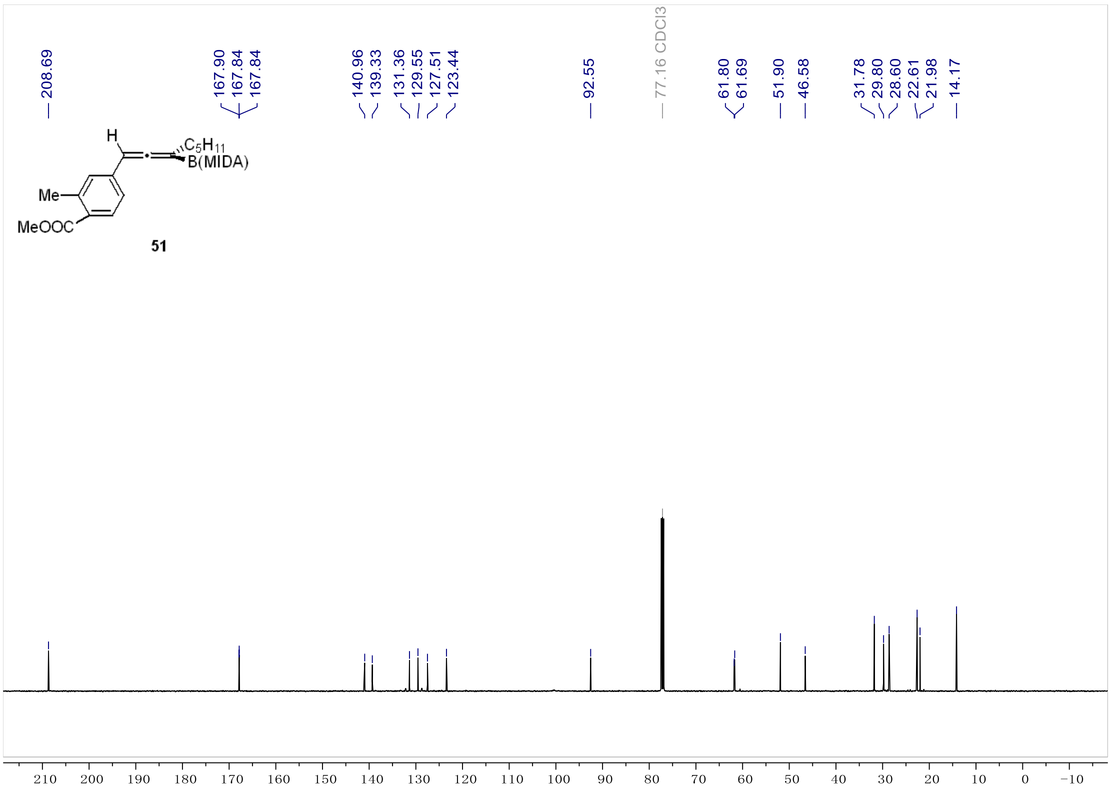

55: ^{1}H NMR (400 MHz, Chloroform-*d*)

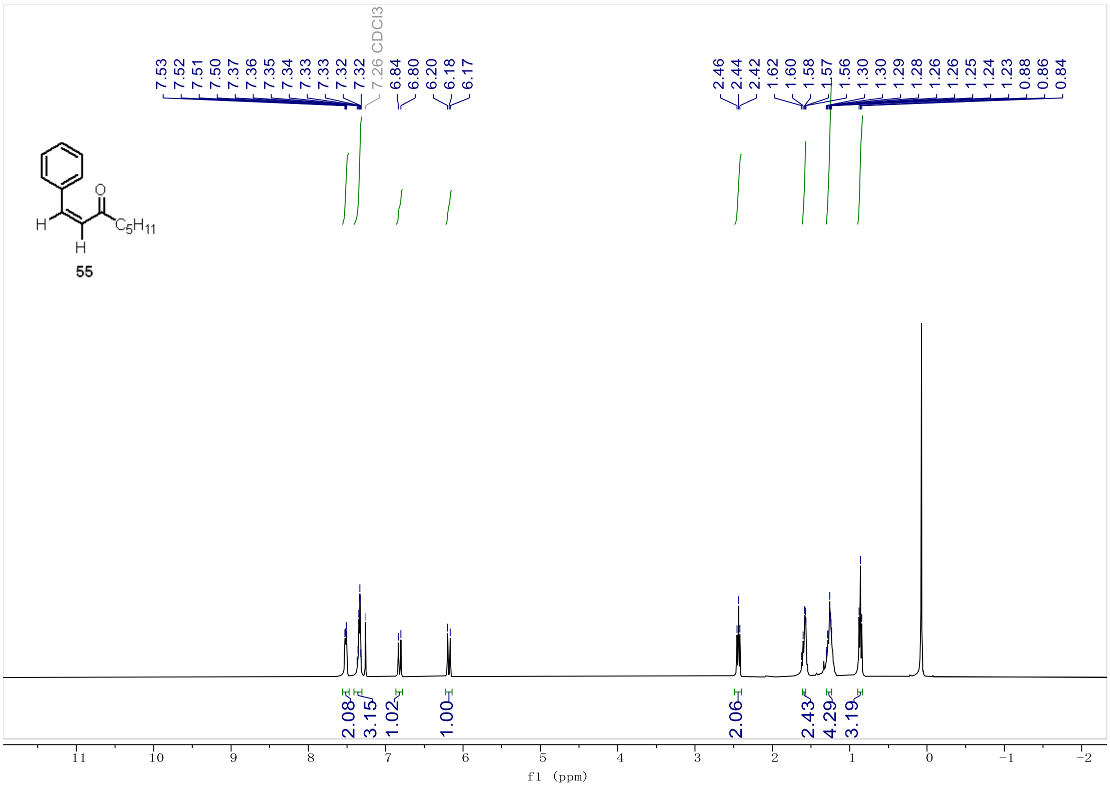

55: ^{13}C NMR (126 MHz, Chloroform-*d*)

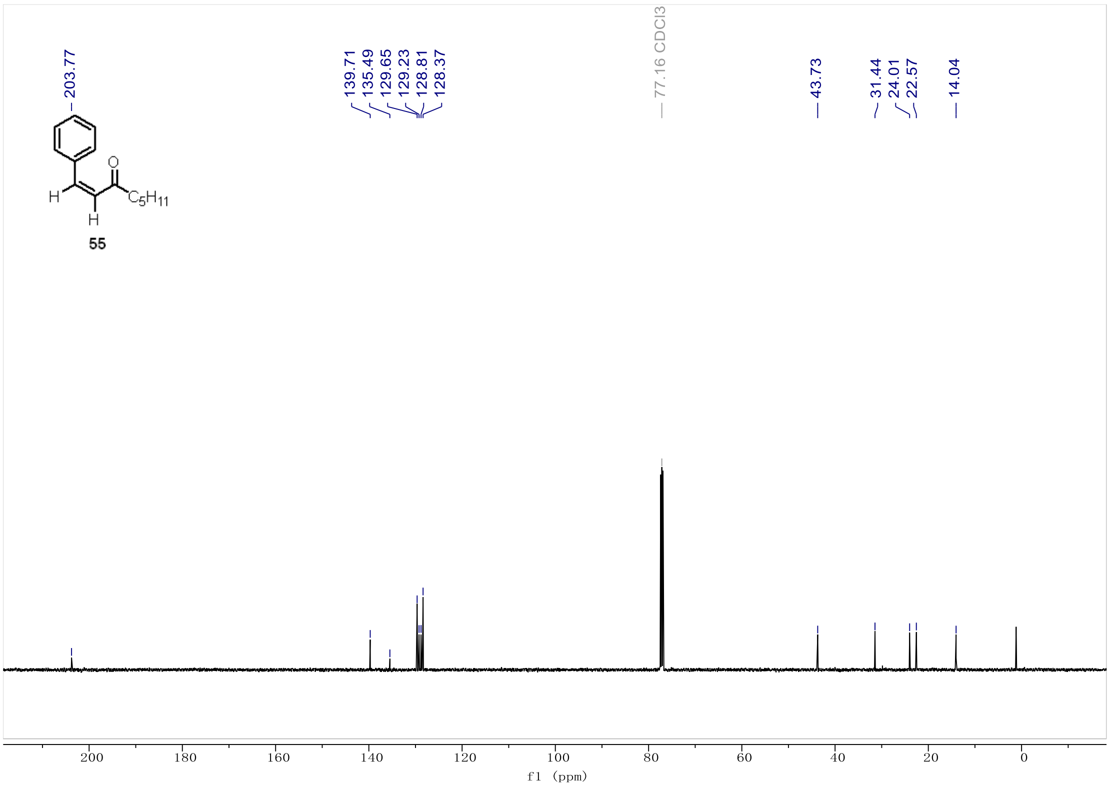

56: ^{1}H NMR (400 MHz, Chloroform-*d*)

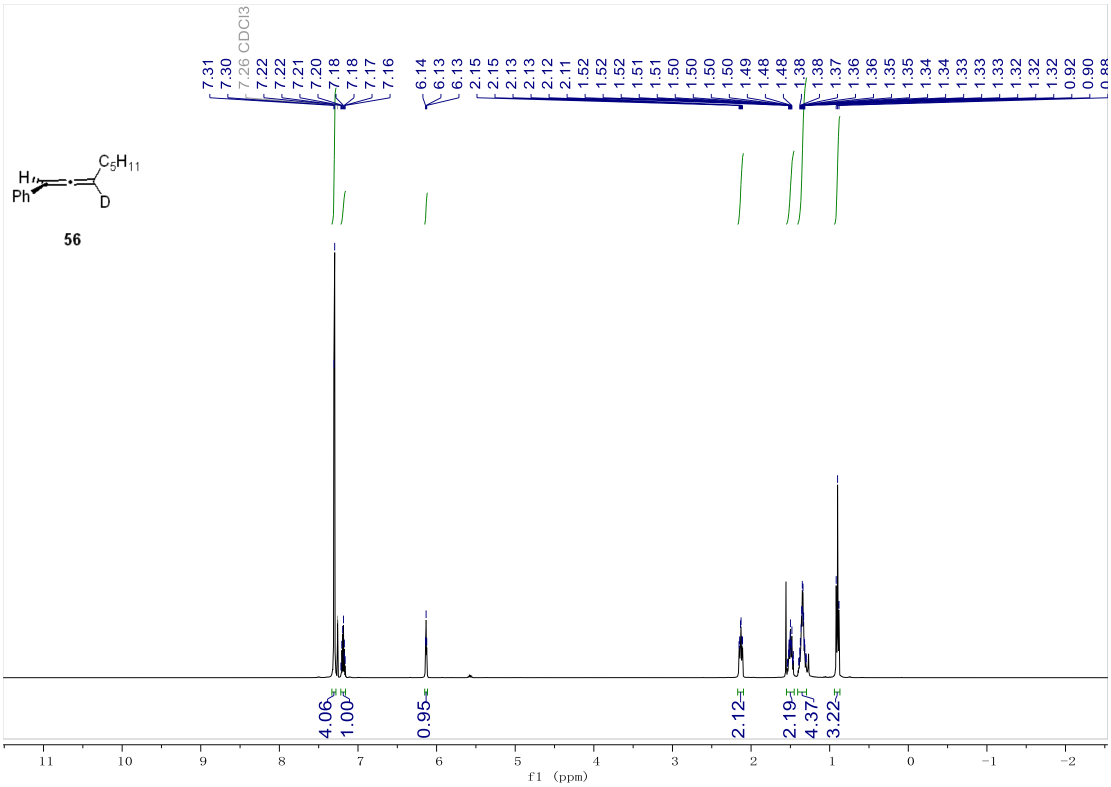

56: ^{13}C NMR (126 MHz, Chloroform-*d*)

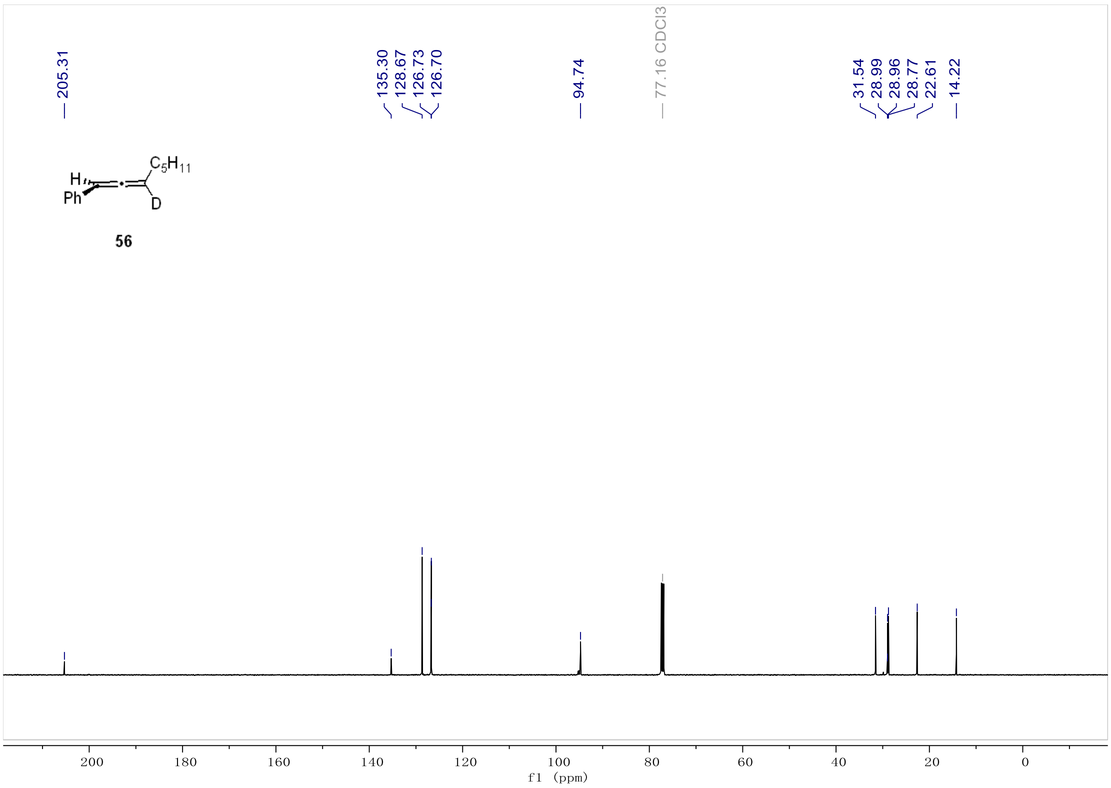

57: ^{1}H NMR (500 MHz, Chloroform-*d*)

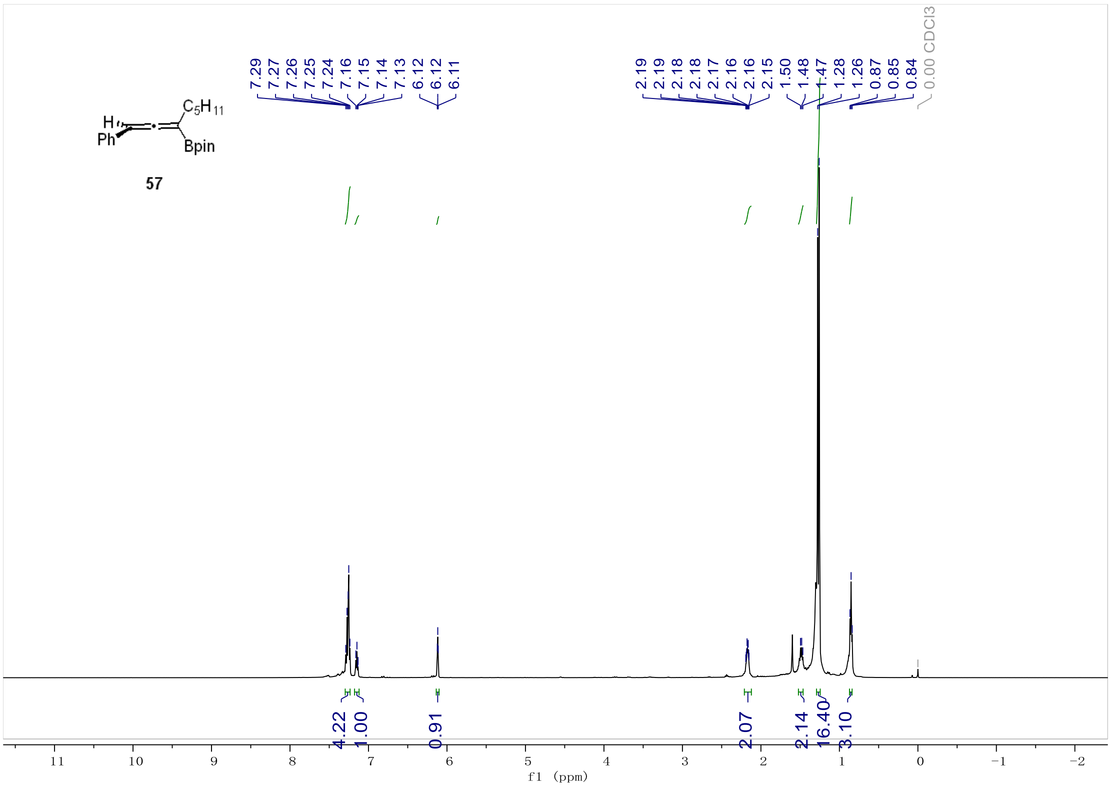

57: ^{13}C NMR (126 MHz, Chloroform-*d*)

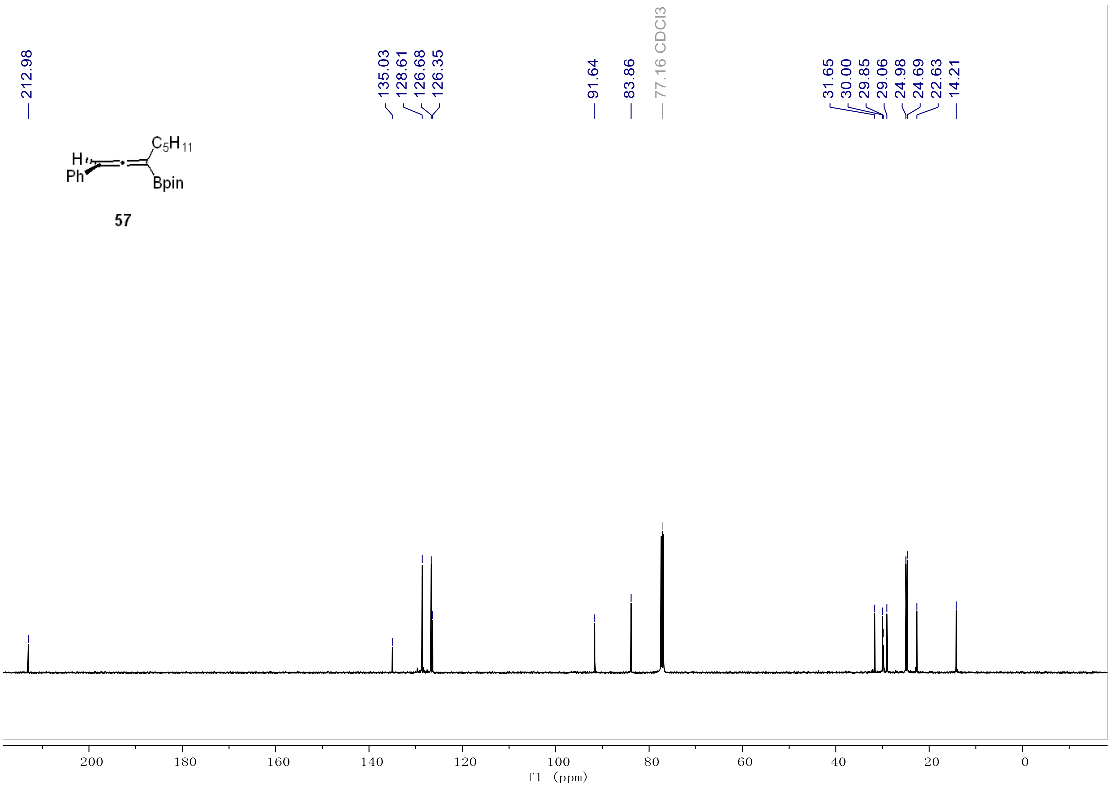

58: ^{1}H NMR (400 MHz, Chloroform-*d*)

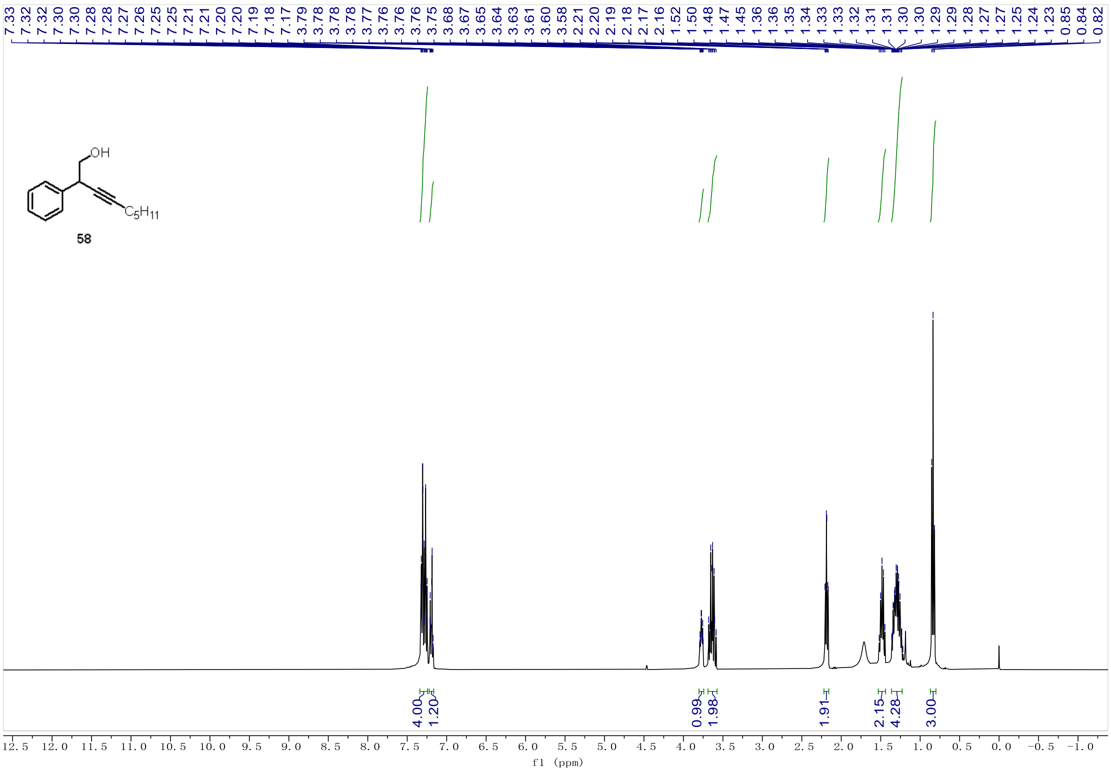

58: ^{13}C NMR (101 MHz, Chloroform-*d*)

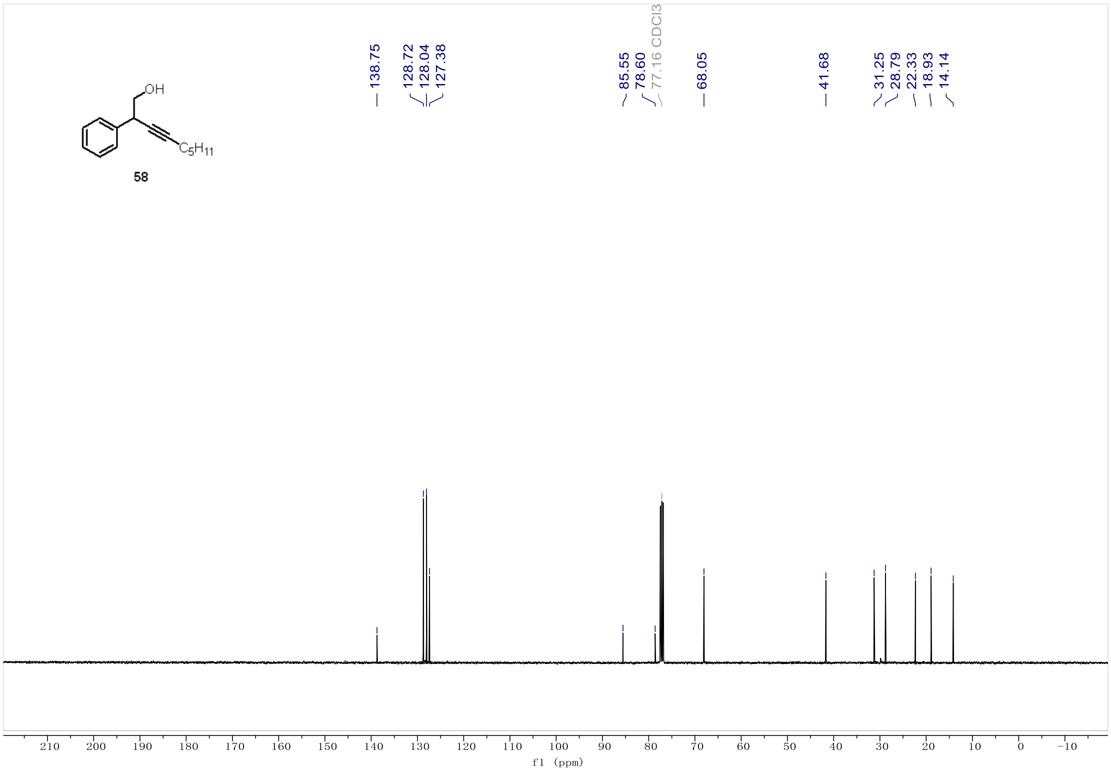

59: ^{1}H NMR (400 MHz, Chloroform-*d*)

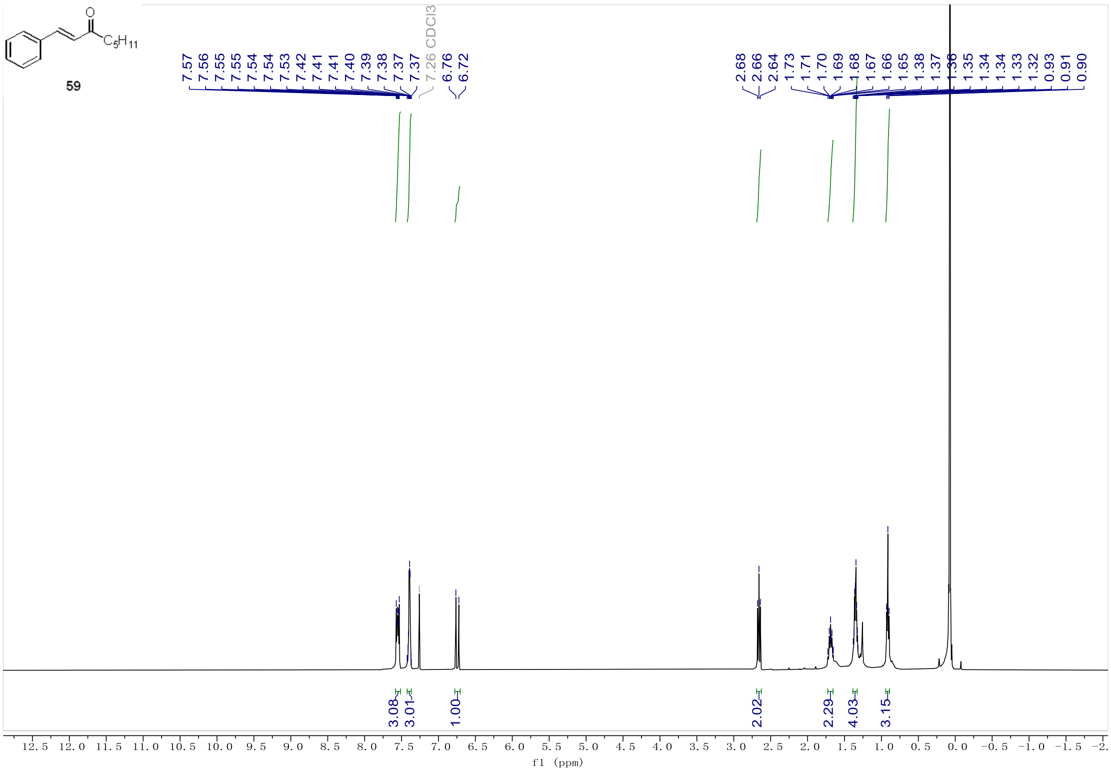

60: ^{1}H NMR (400 MHz, Chloroform-*d*)

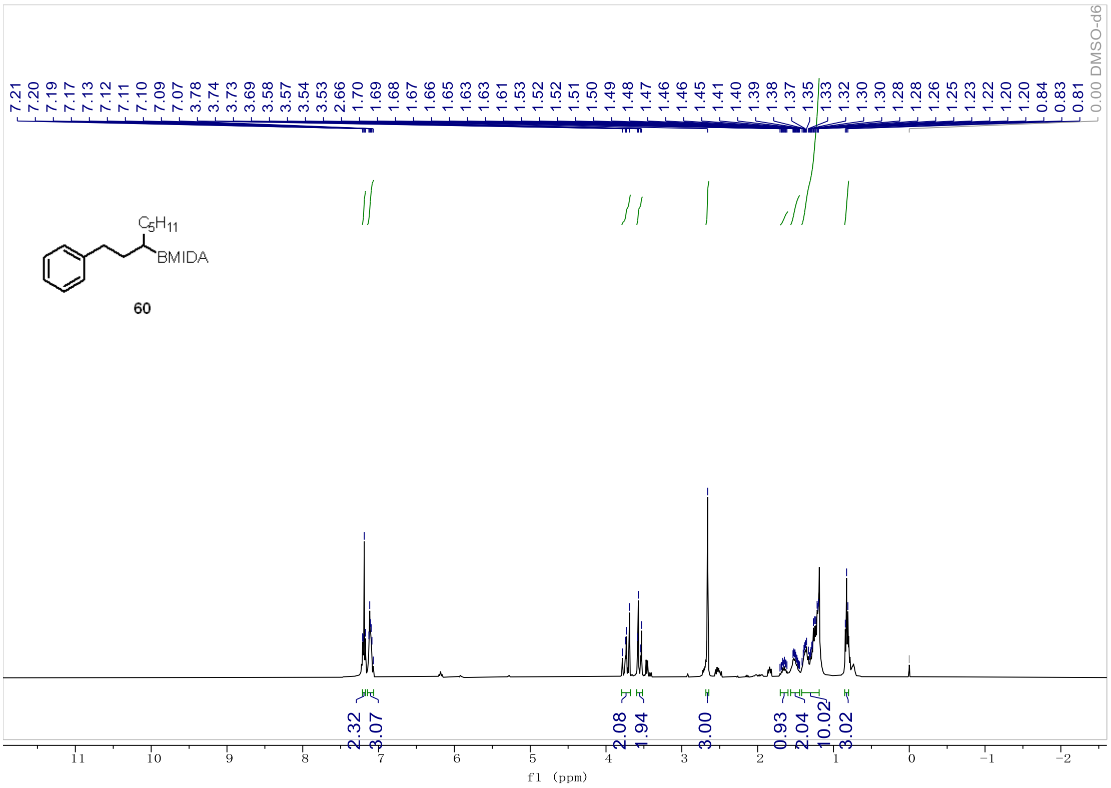

60: ^{13}C NMR (126 MHz, Chloroform-*d*)

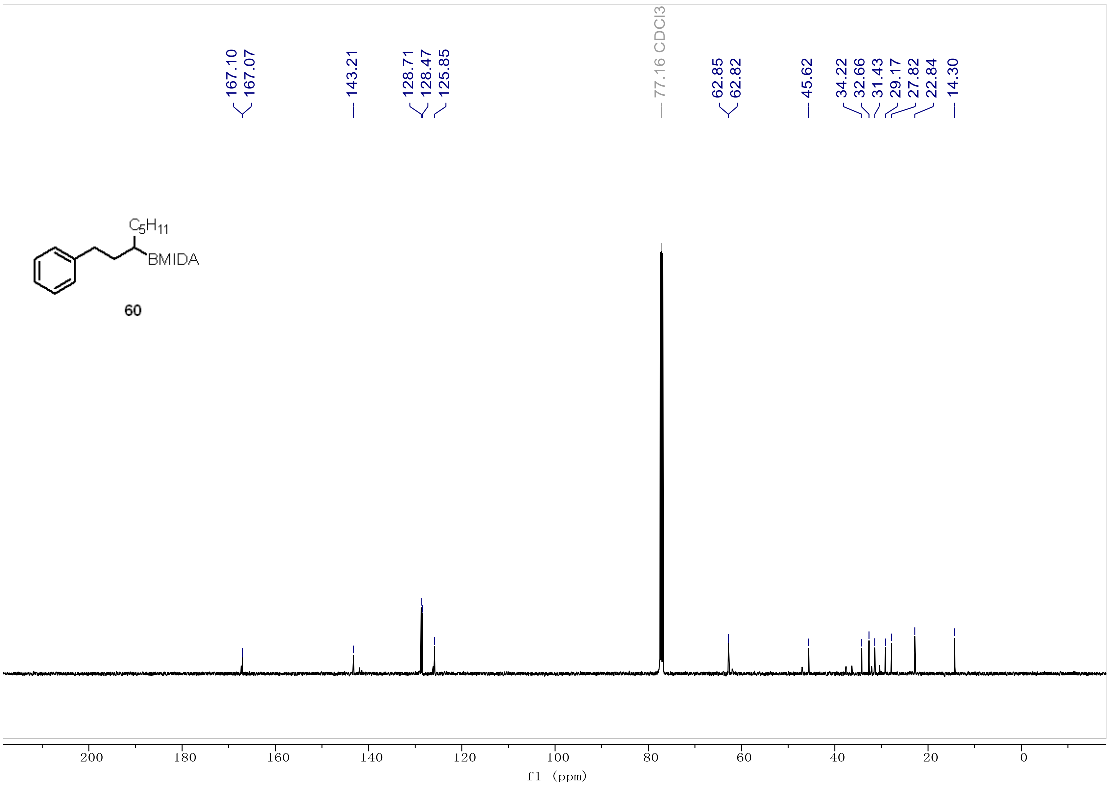

61: ^{1}H NMR (400 MHz, Acetone-d$_6$)

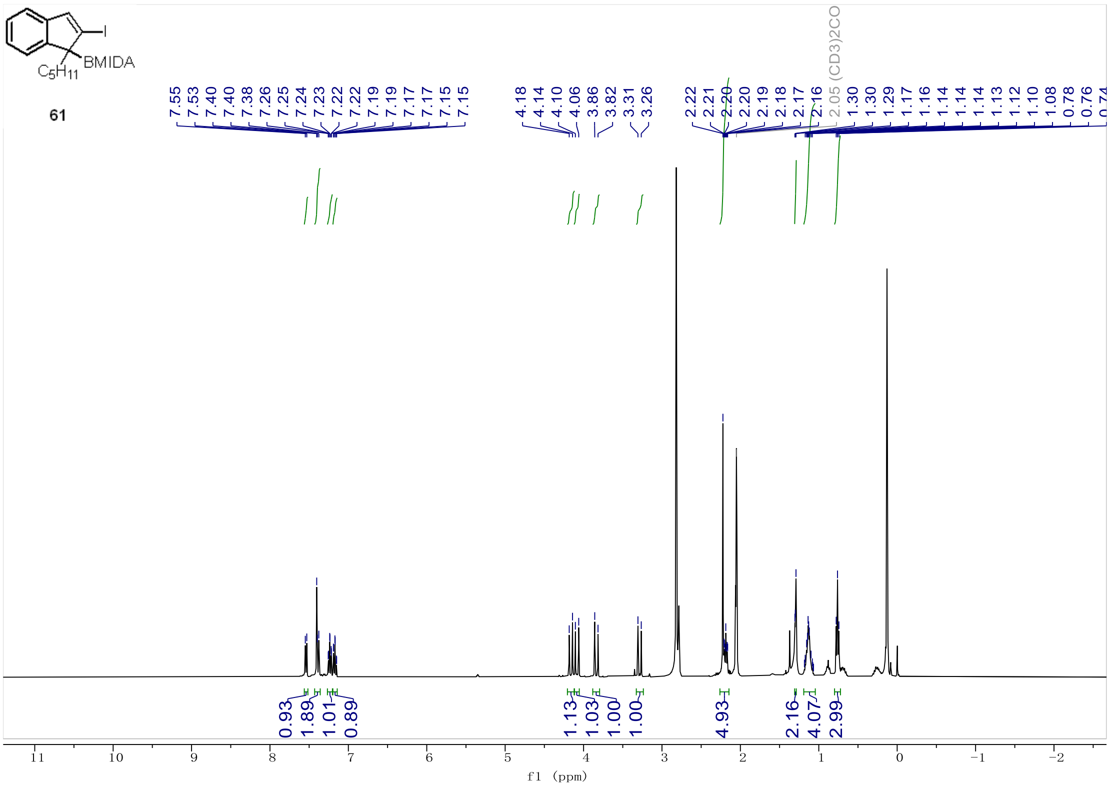

61: ^{13}C NMR (101 MHz, Acetone-*d*$_6$)

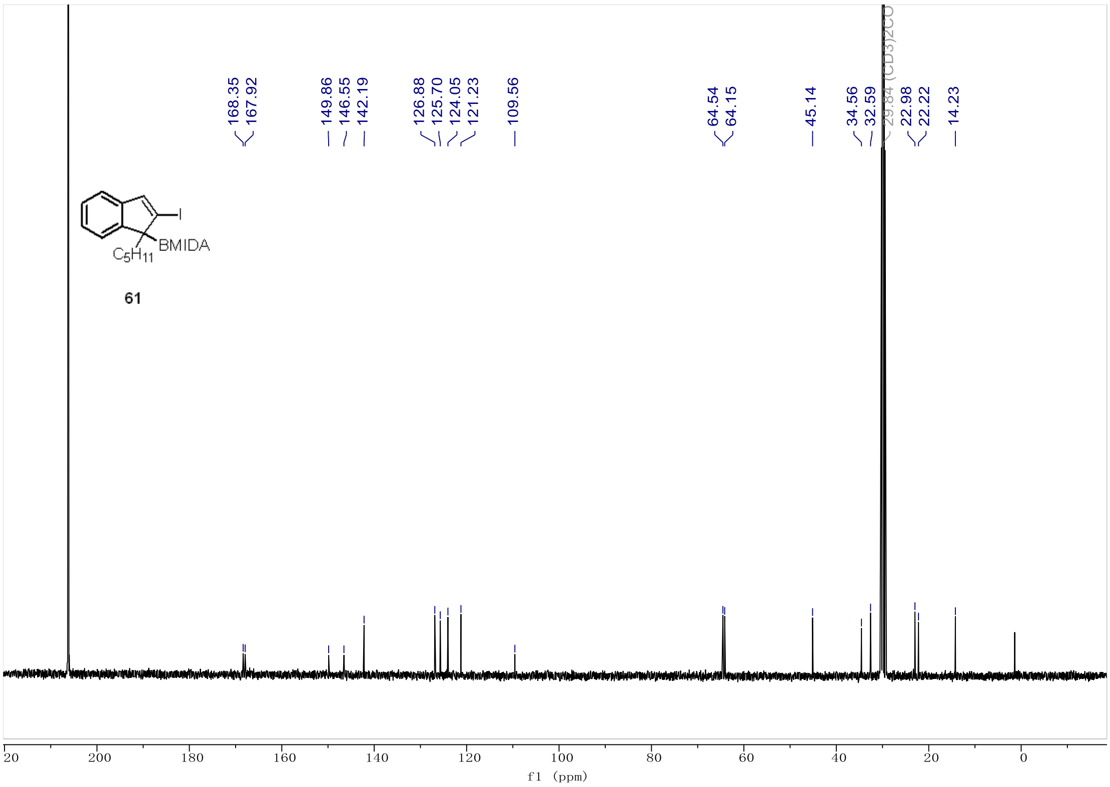

62: ^{1}H NMR (400 MHz, Chloroform-*d*)

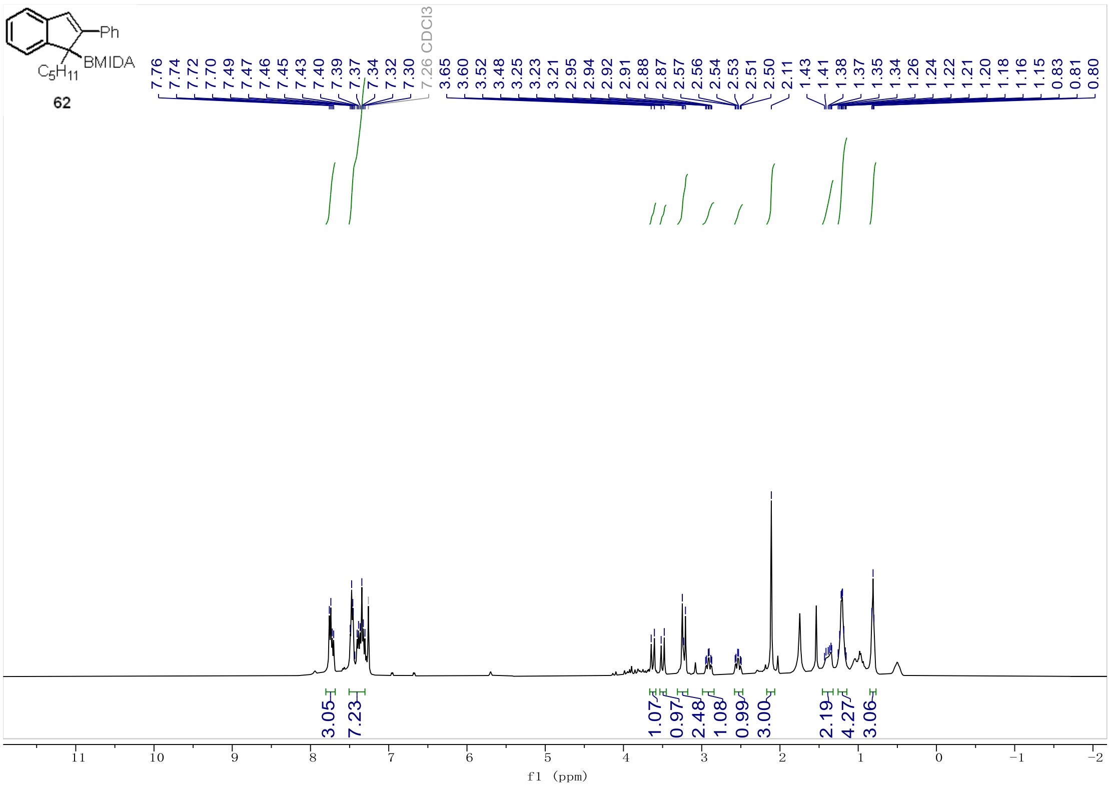

62: ^{13}C NMR (101 MHz, Chloroform-*d*)

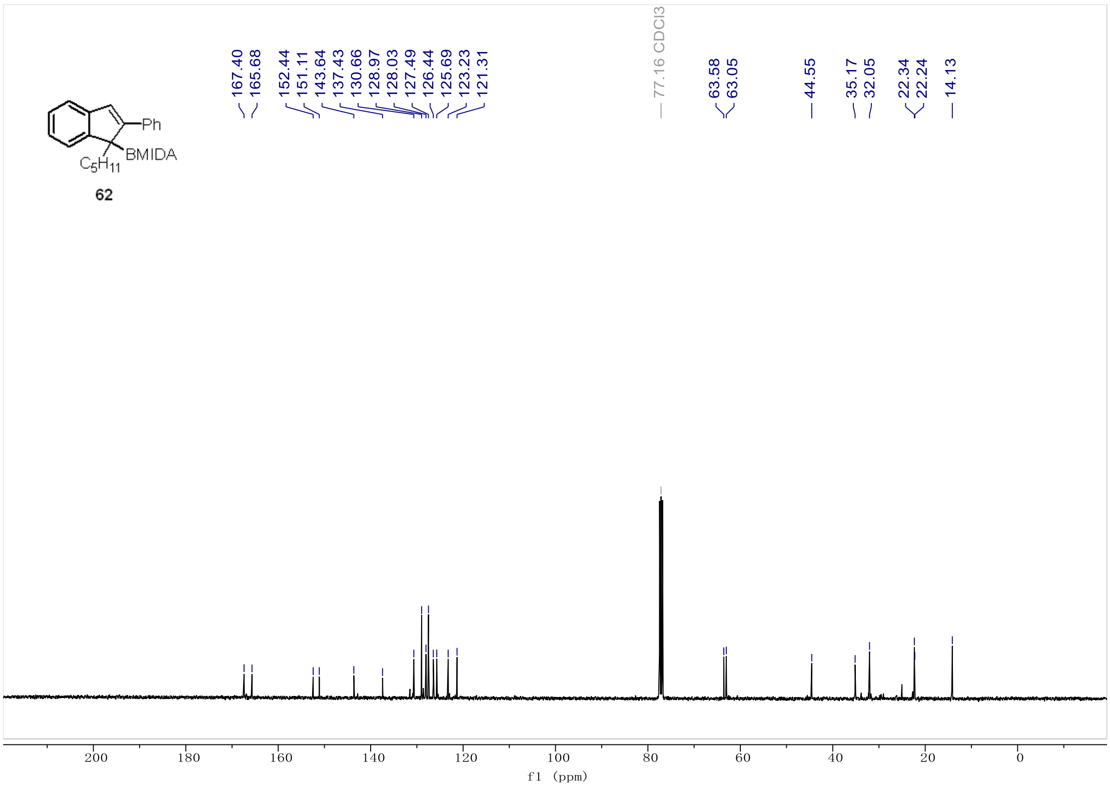

63: ^{1}H NMR (500 MHz, Chloroform-*d*)

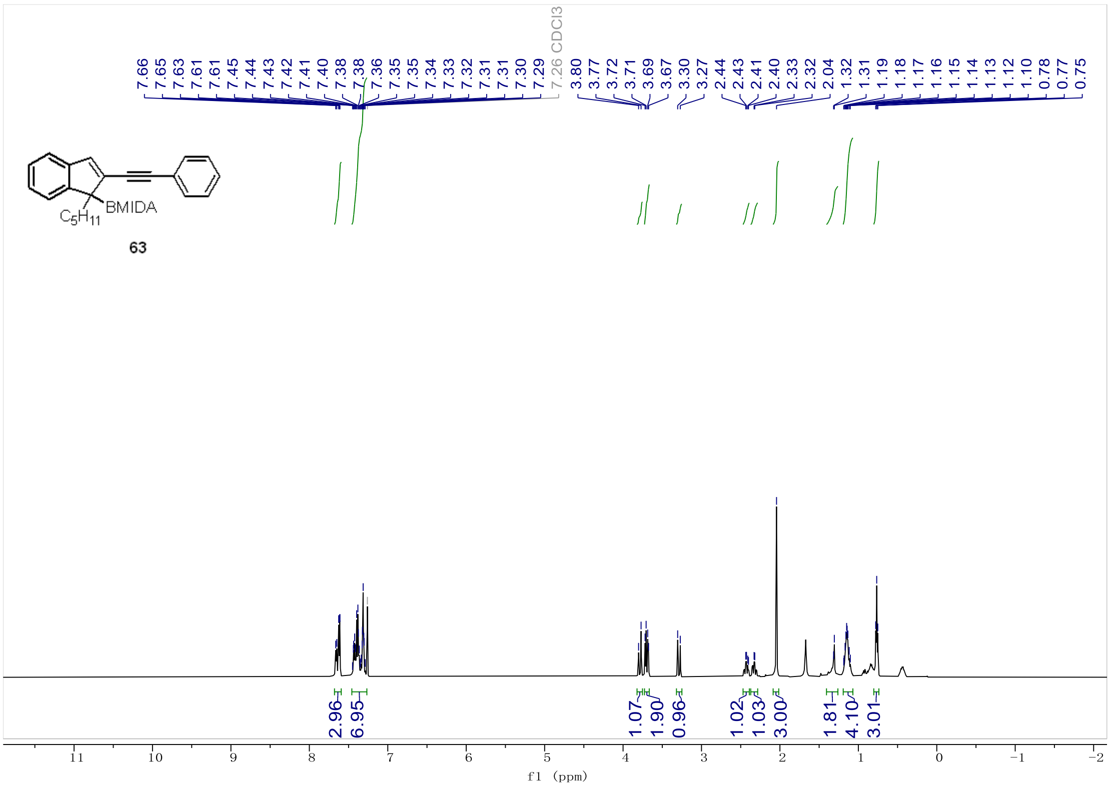

63: ^{13}C NMR (101 MHz, Chloroform-*d*)

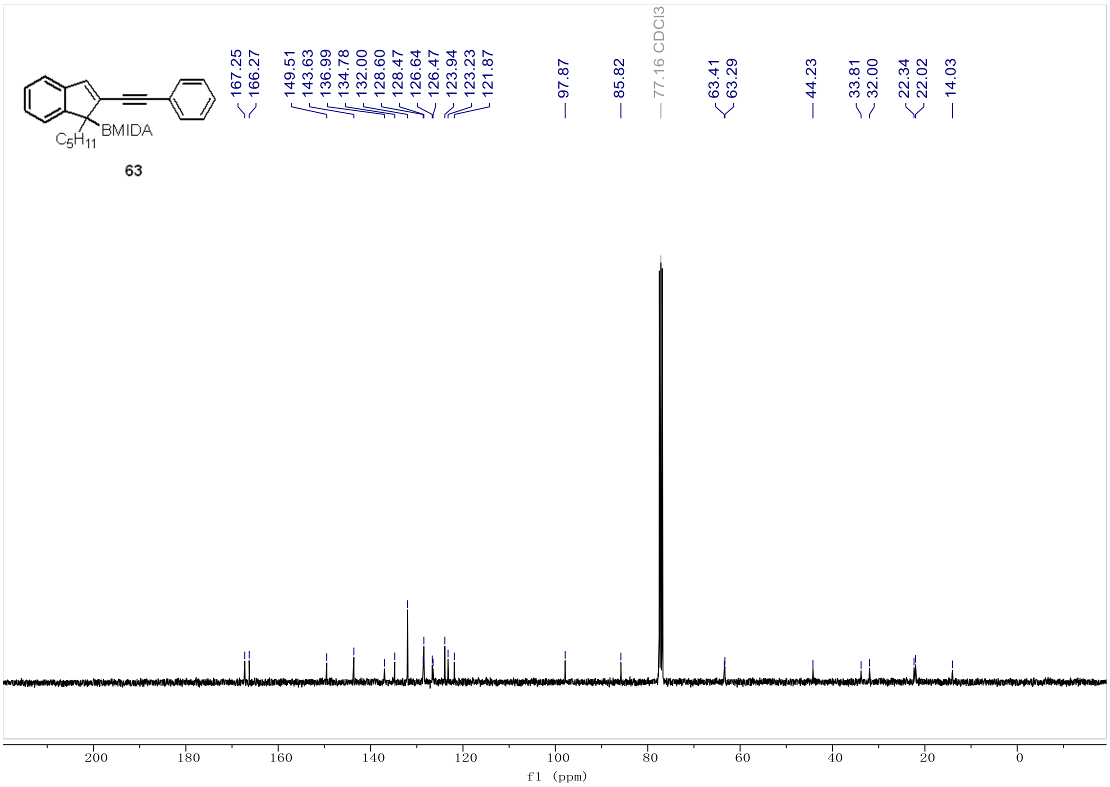

64: ^{1}H NMR (400 MHz, Chloroform-*d*)

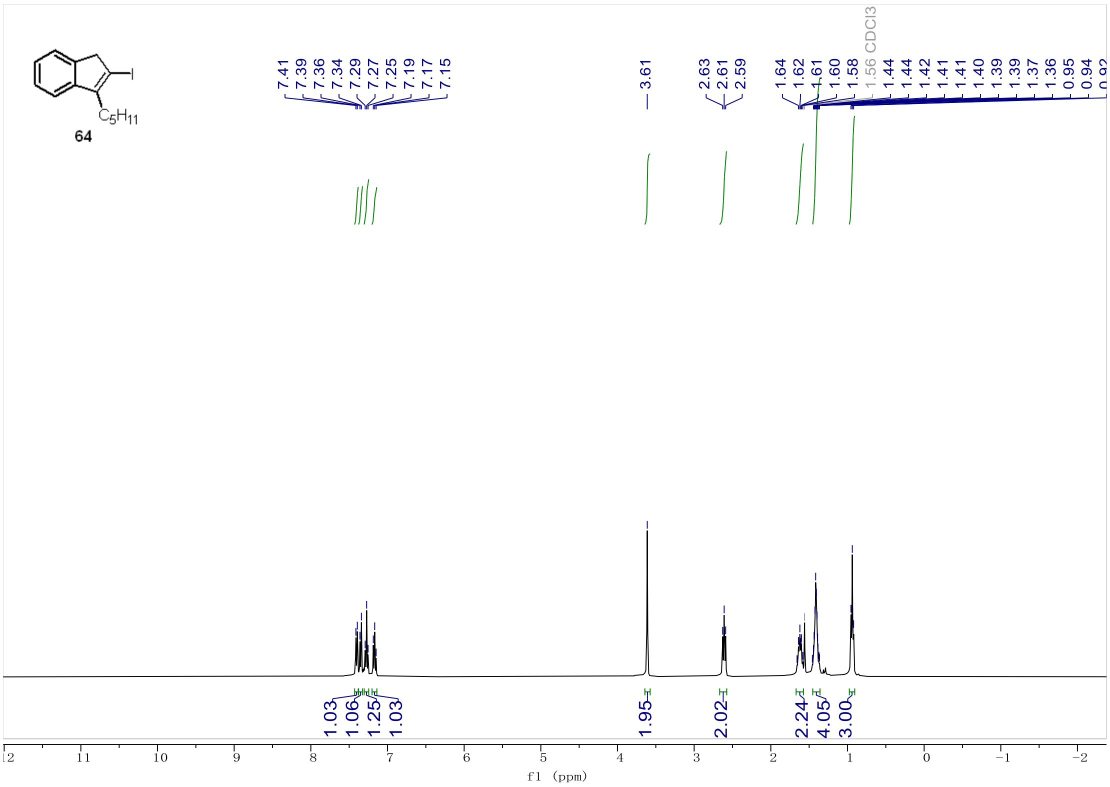

64: ^{13}C NMR (101 MHz, Chloroform-*d*)

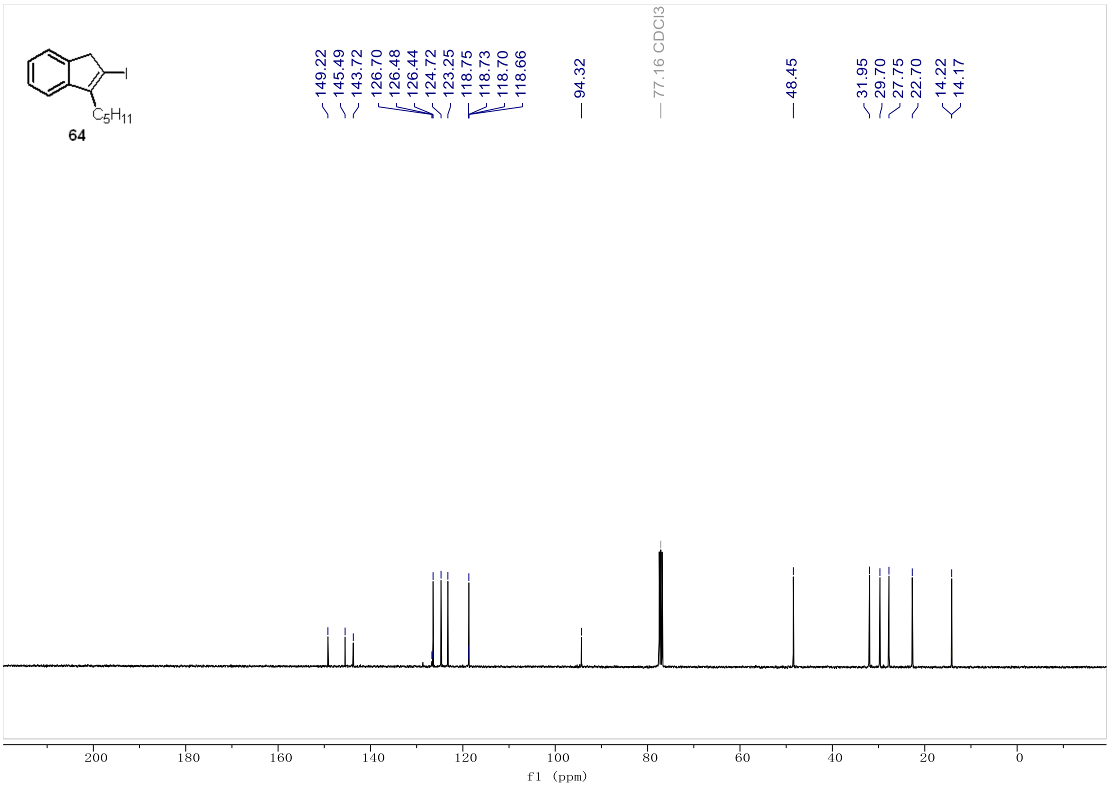

65: ^{1}H NMR (400 MHz, Chloroform-*d*)

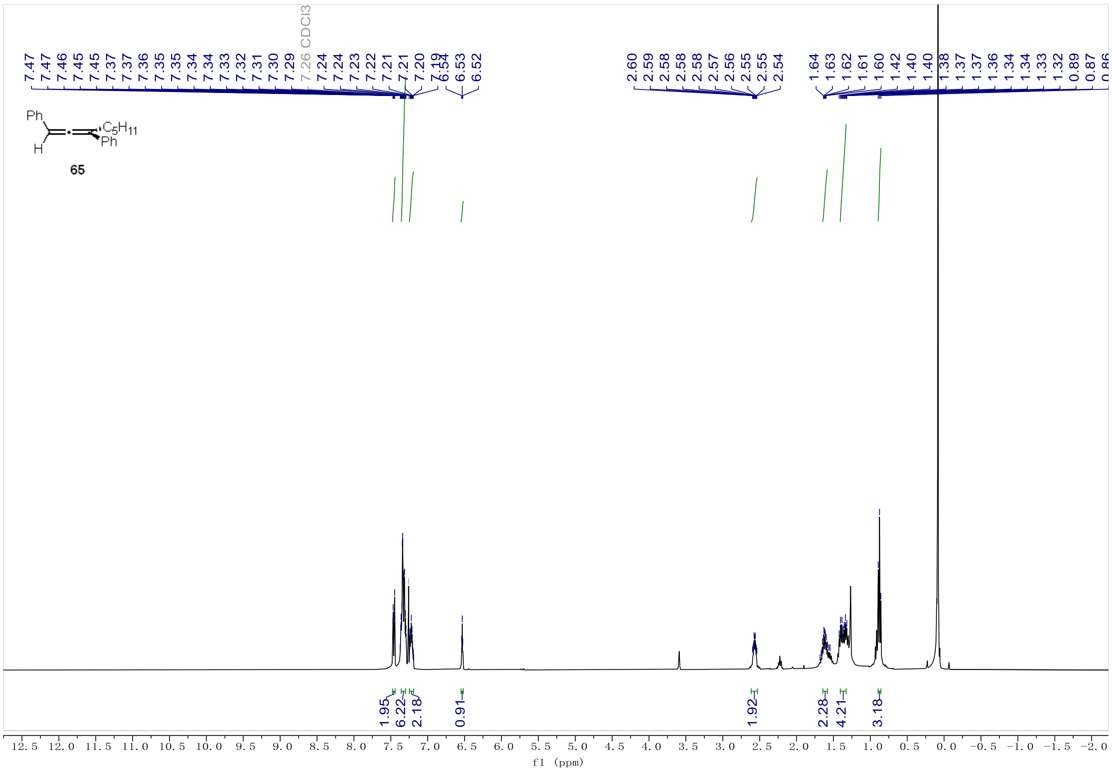

67: ^{1}H NMR (400 MHz, Chloroform-*d*)

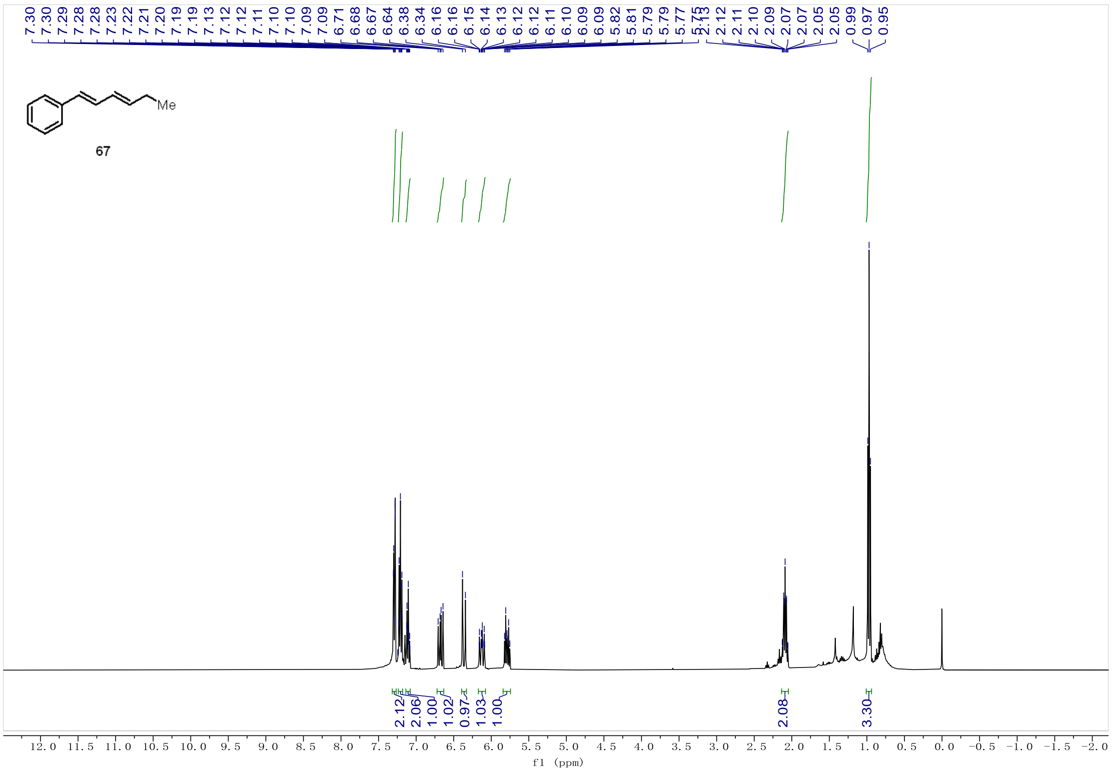

69: ^{1}H NMR (400 MHz, Chloroform-*d*)

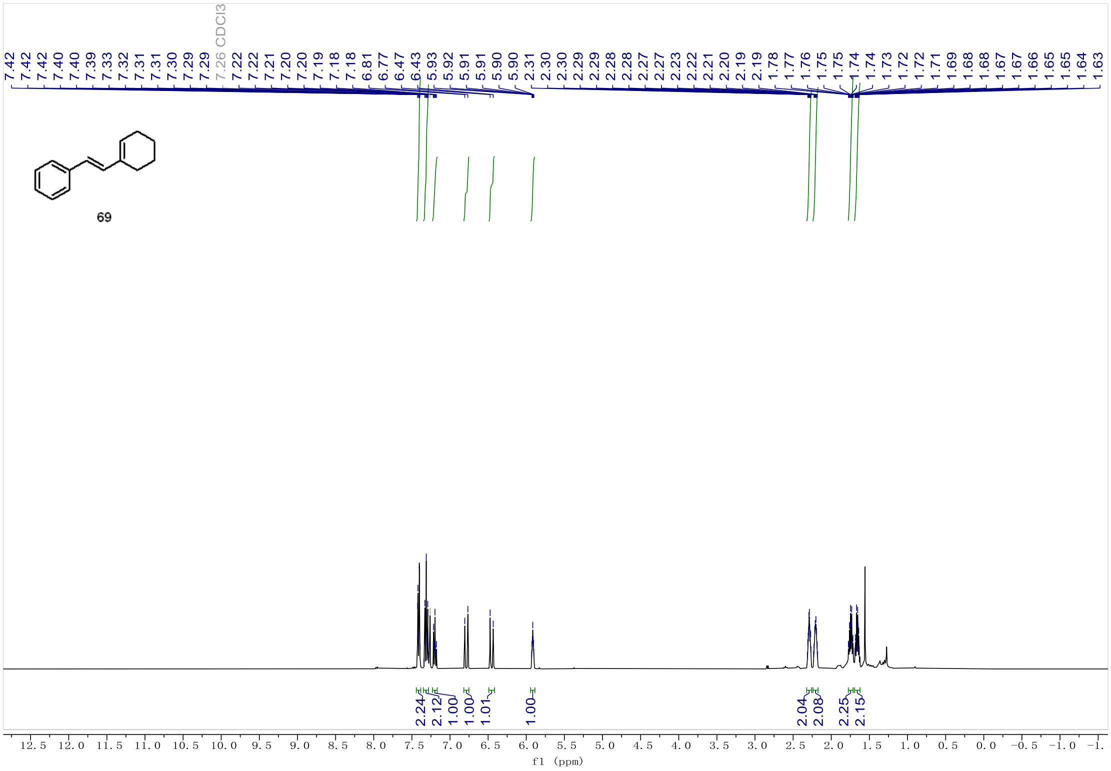

78: ^{1}H NMR (400 MHz, Chloroform-*d*)

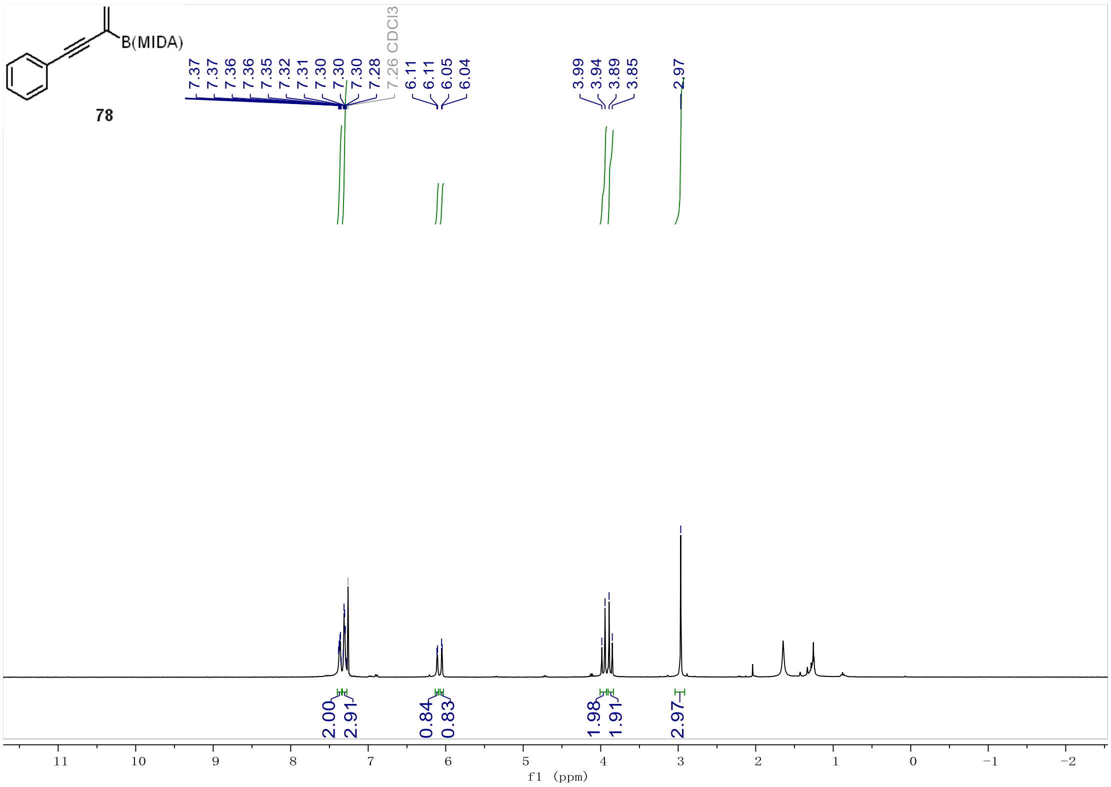

78: ^{13}C NMR (101 MHz, Acetone-*d$_6$*)

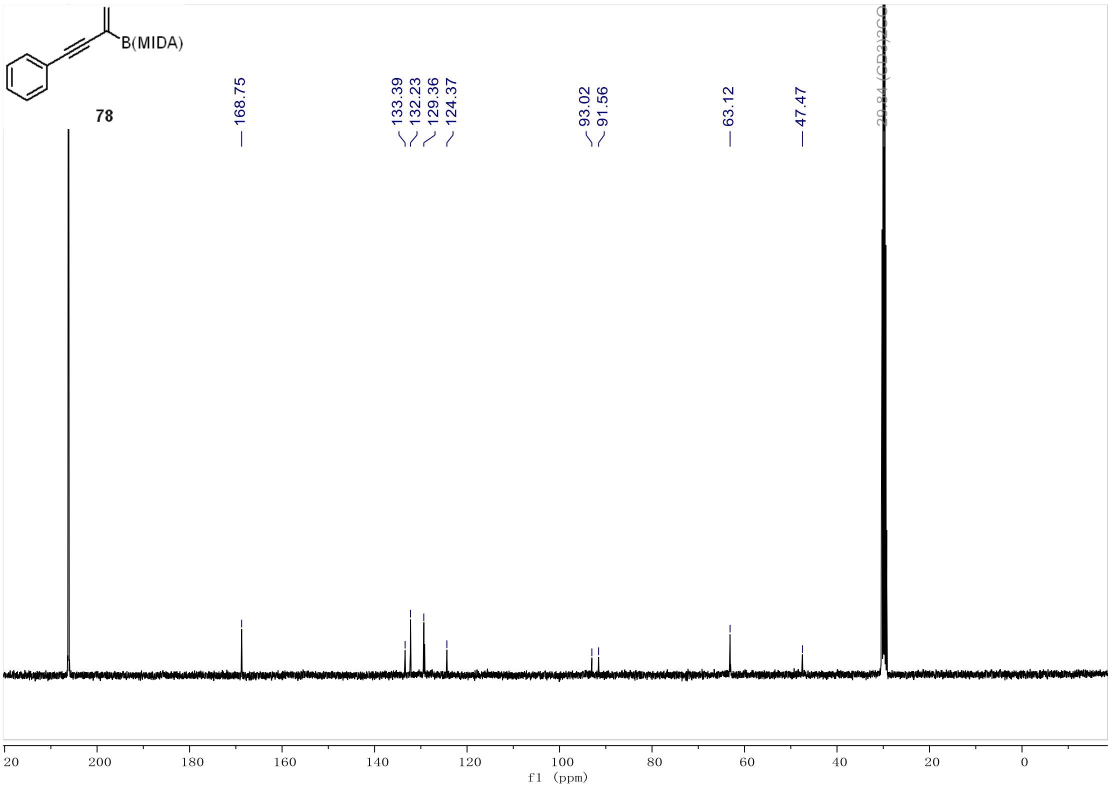

79: ^{1}H NMR (400 MHz, Chloroform-*d*)

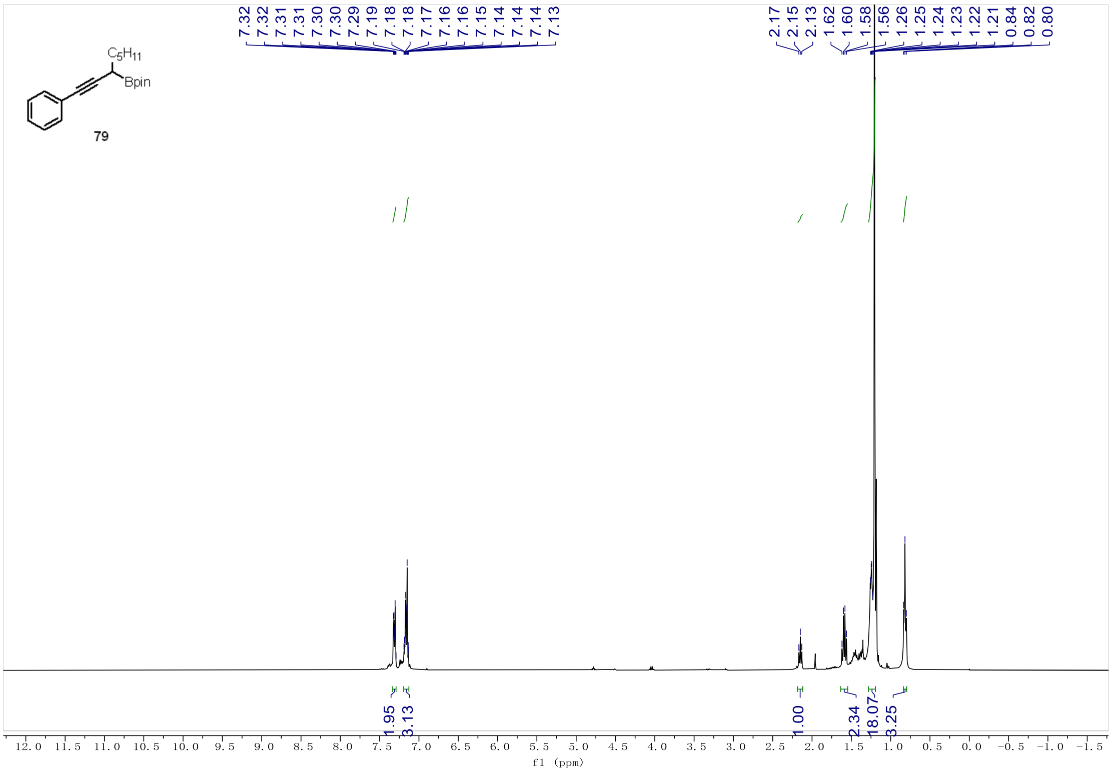

79: ^{13}C NMR (400 MHz, Chloroform-*d*)

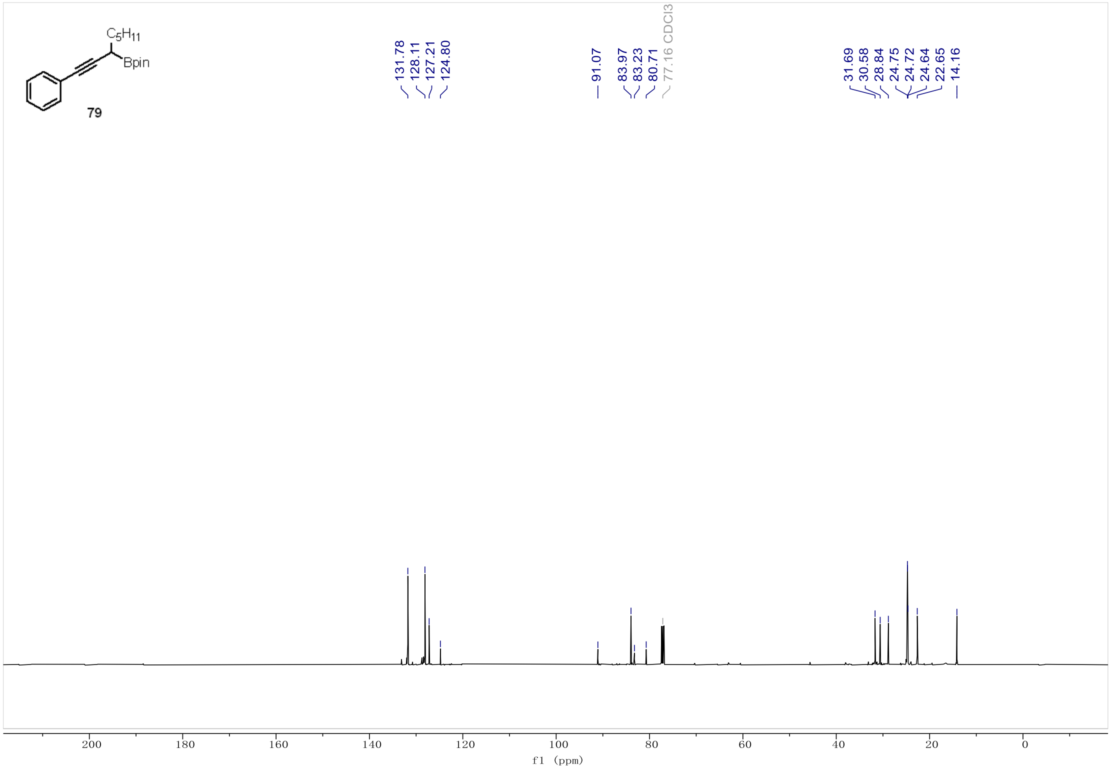

80: ^{1}H NMR (400 MHz, Chloroform-*d*)

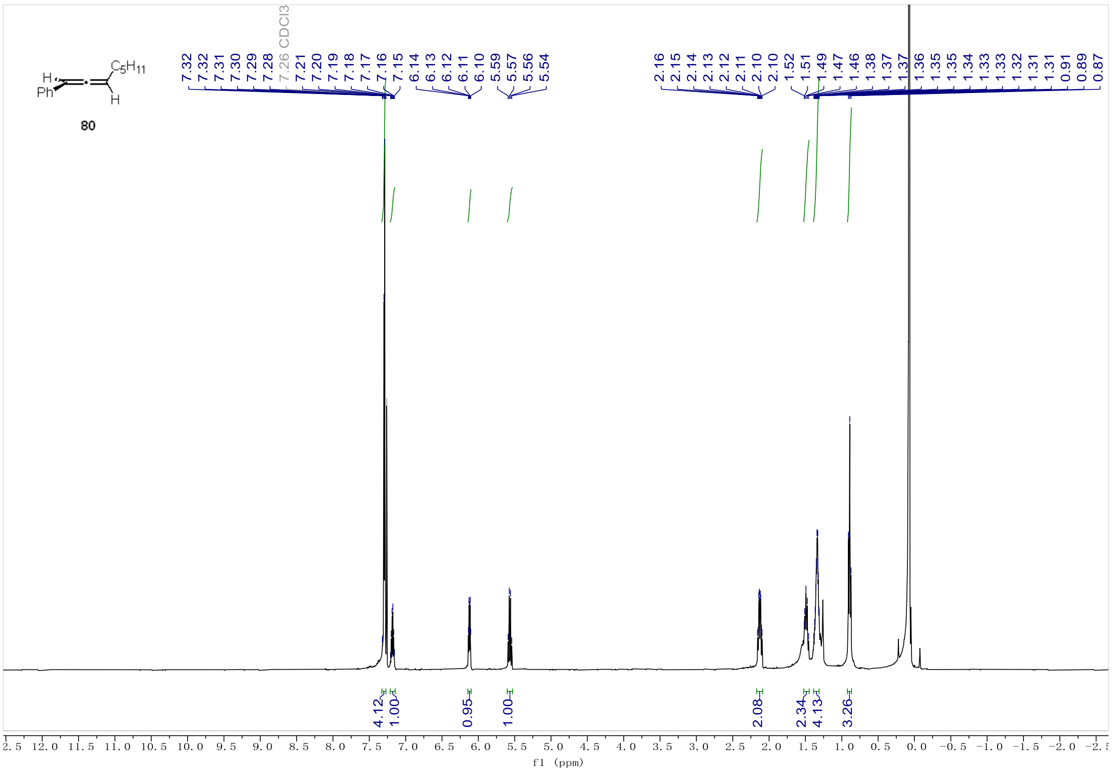

81: ^{1}H NMR (500 MHz, Chloroform-*d*)

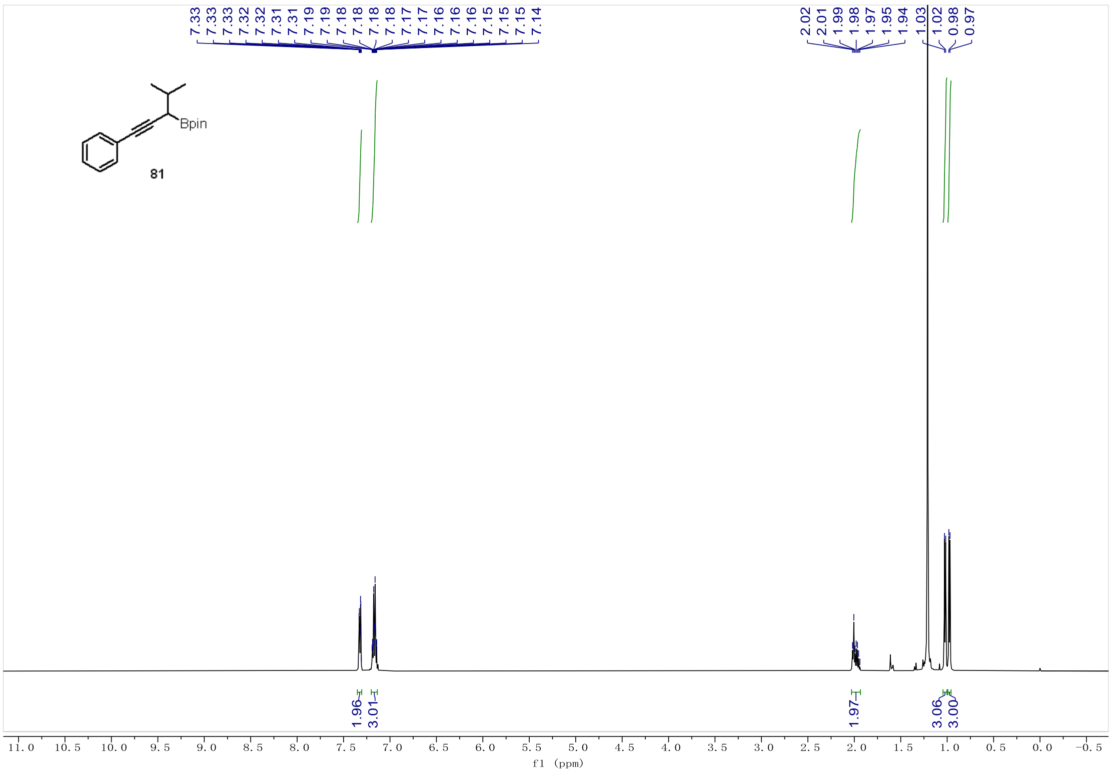

81: ^{13}C NMR (101 MHz, Chloroform-*d*)

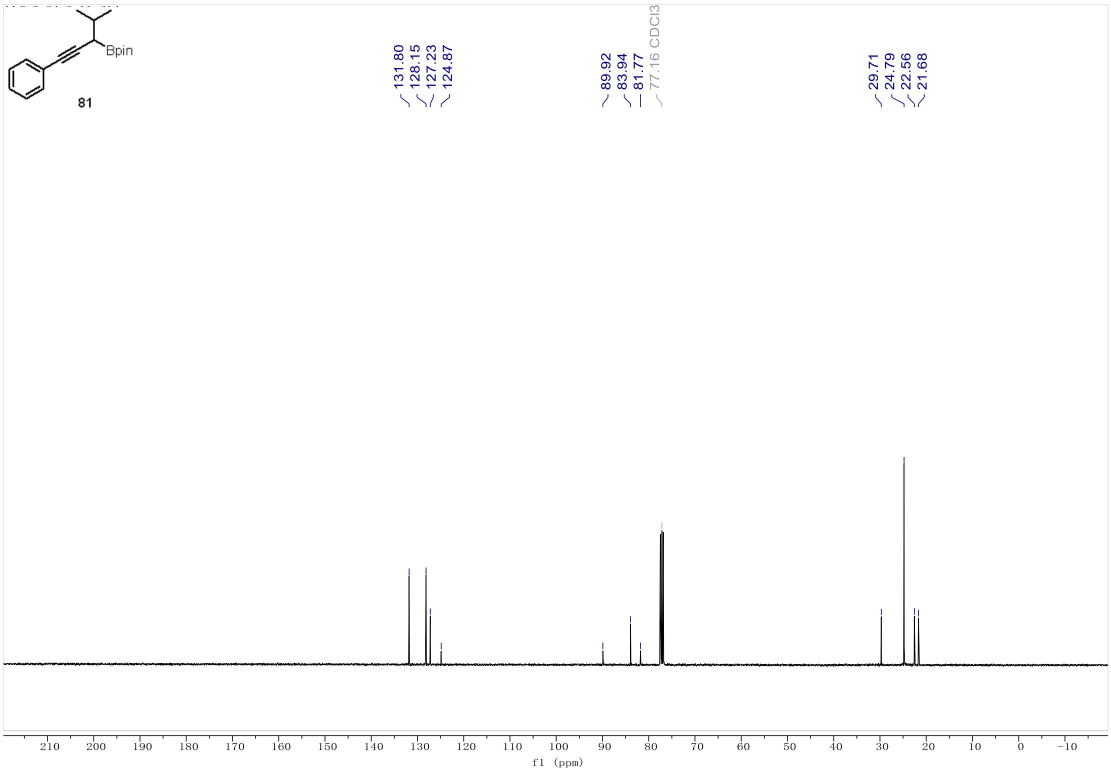

82: ^{1}H NMR (400 MHz, Chloroform-*d*)

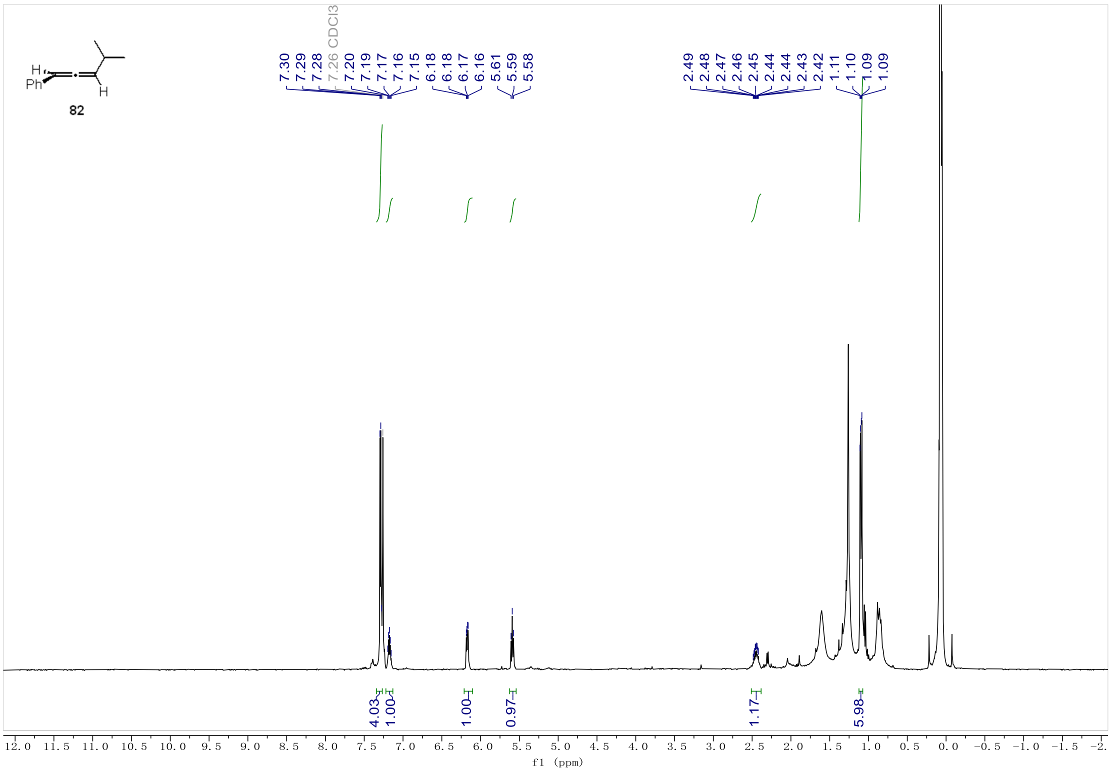

83: ^{1}H NMR (400 MHz, DMSO-d_6)

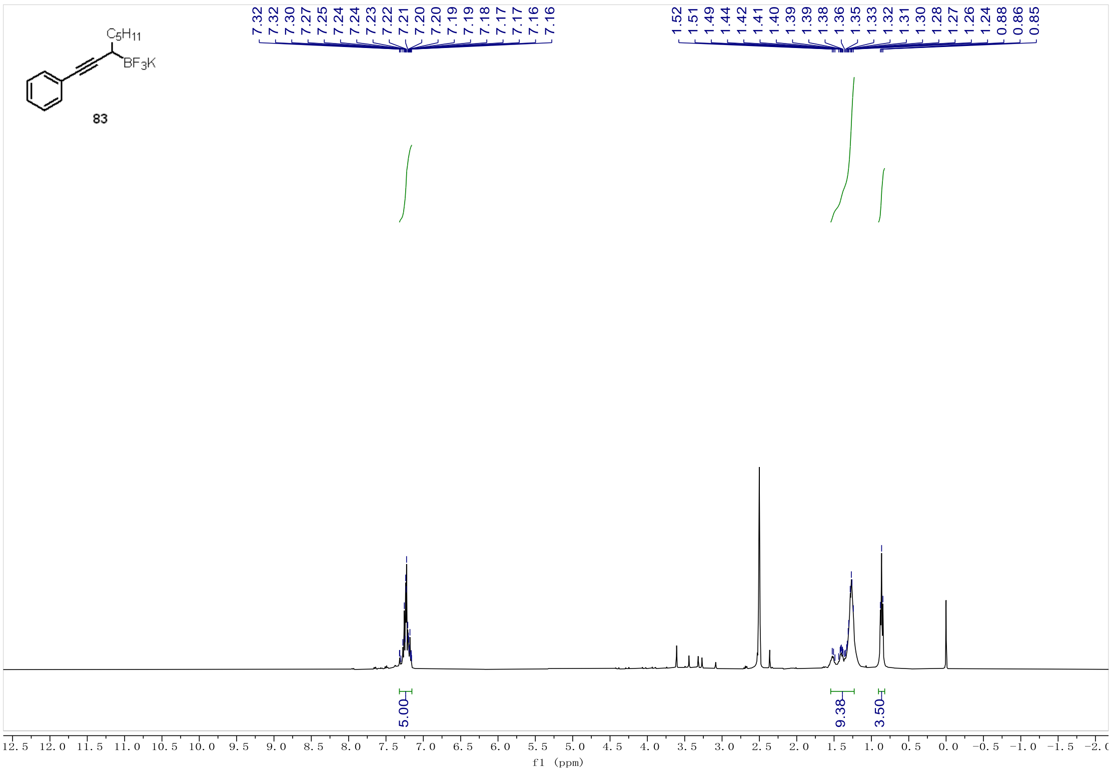

83: ^{13}C NMR (126 MHz, DMSO-d_6)

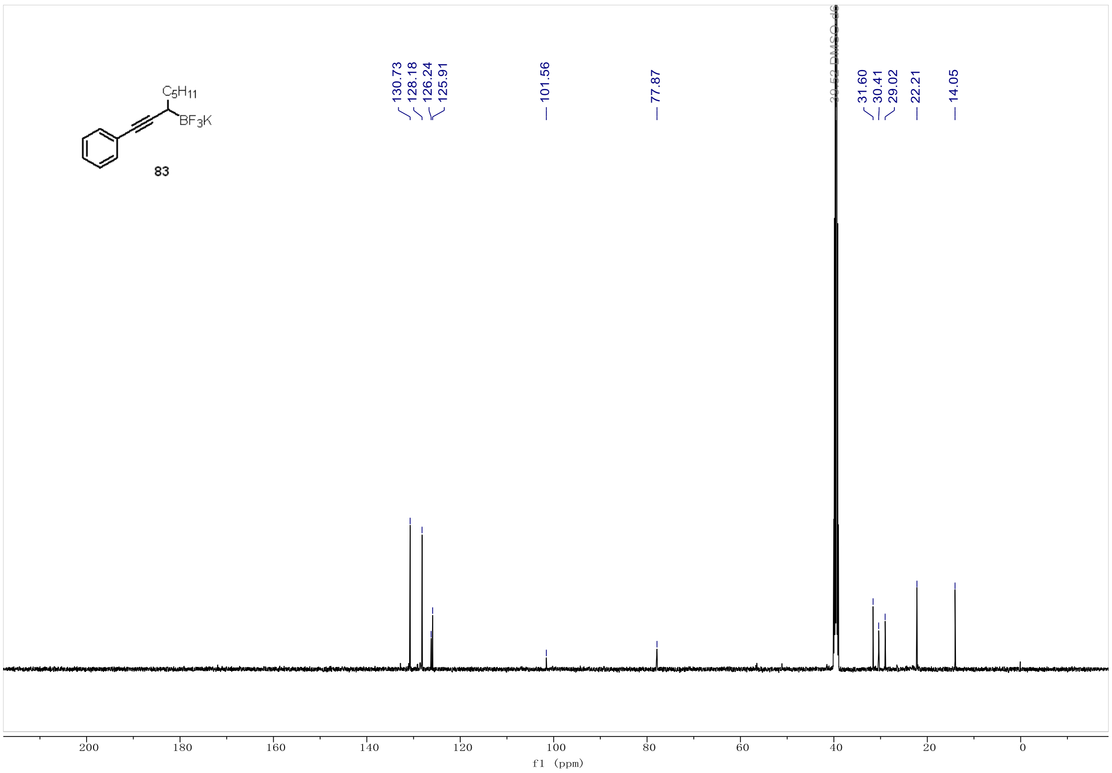

83: ^{11}B NMR (128 MHz, DMSO-d_6)

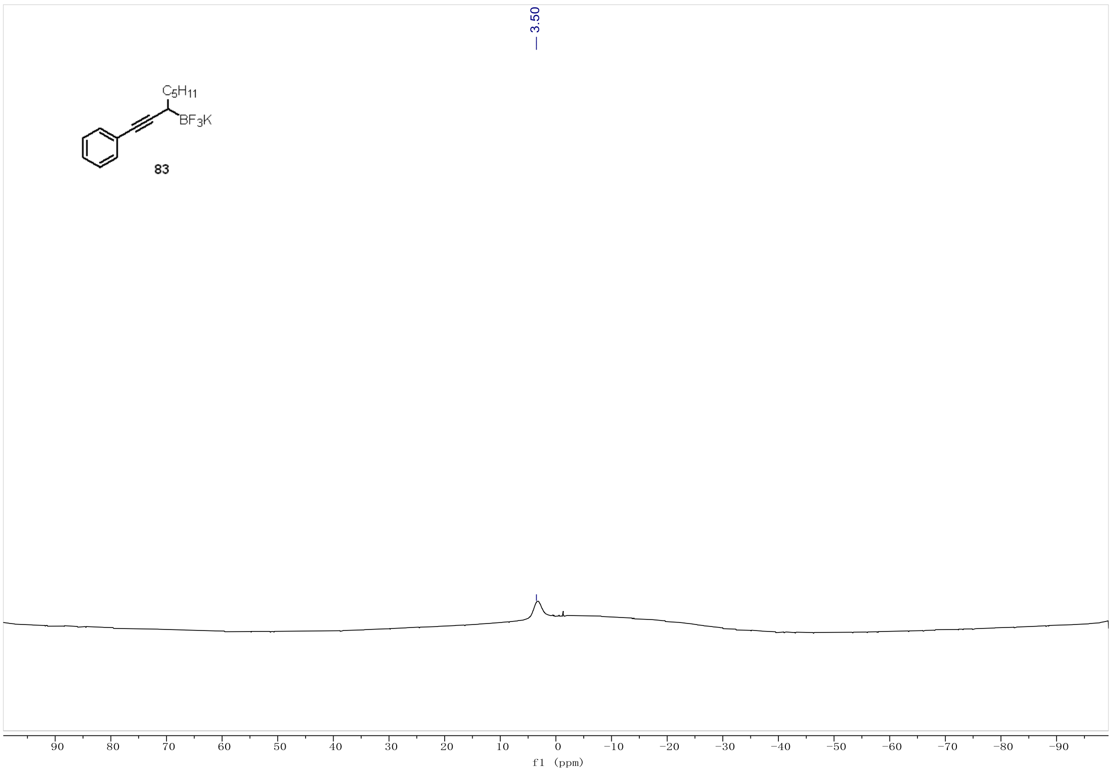

83: ^{19}F NMR (376 MHz, DMSO-d_6)

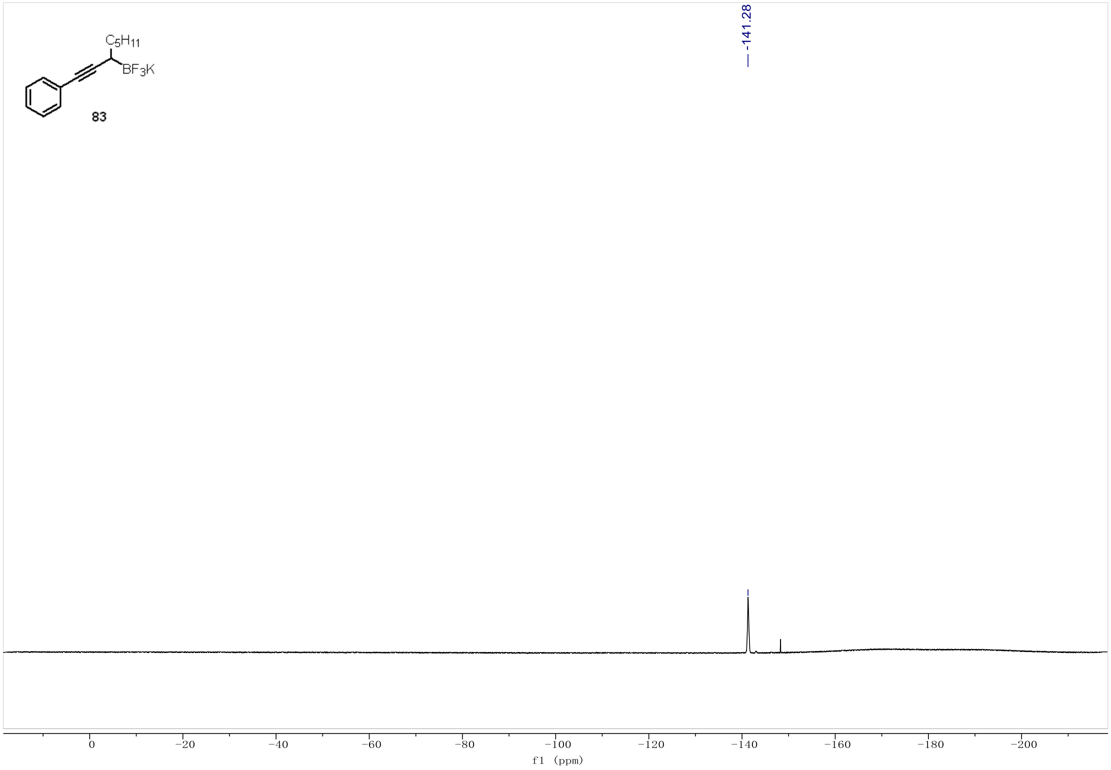

84: ^{1}H NMR (400 MHz, DMSO-d_6)

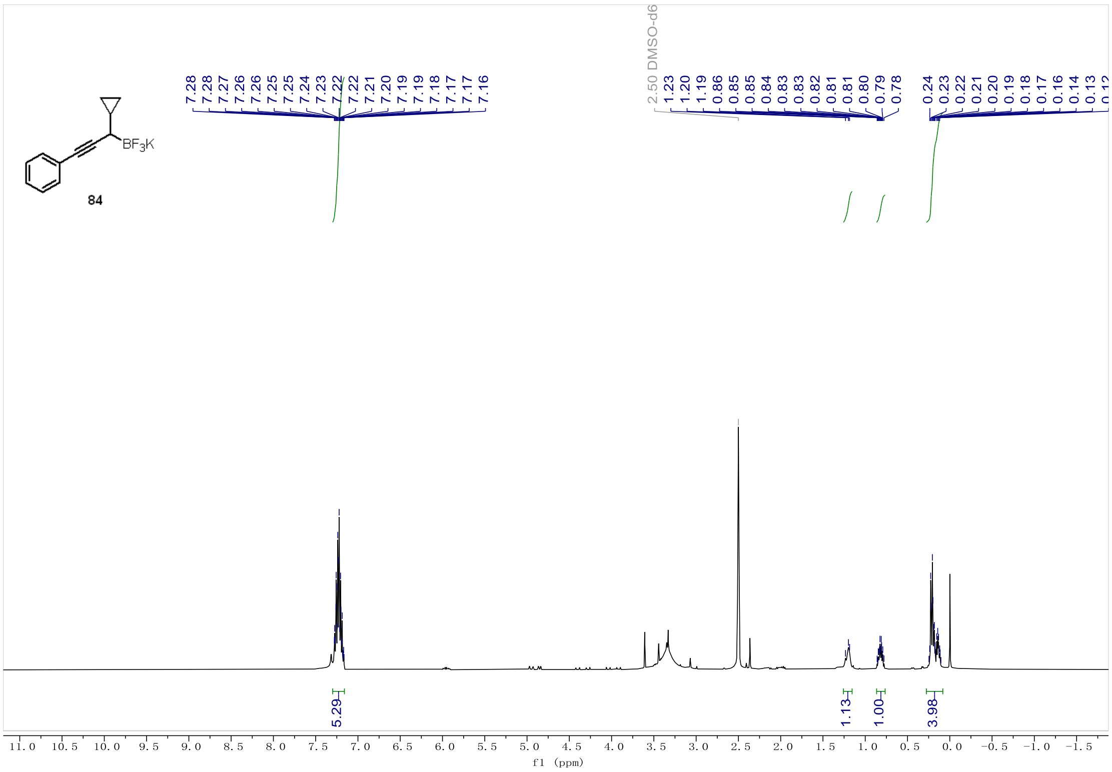

84: ^{13}C NMR (101 MHz, DMSO-d_6)

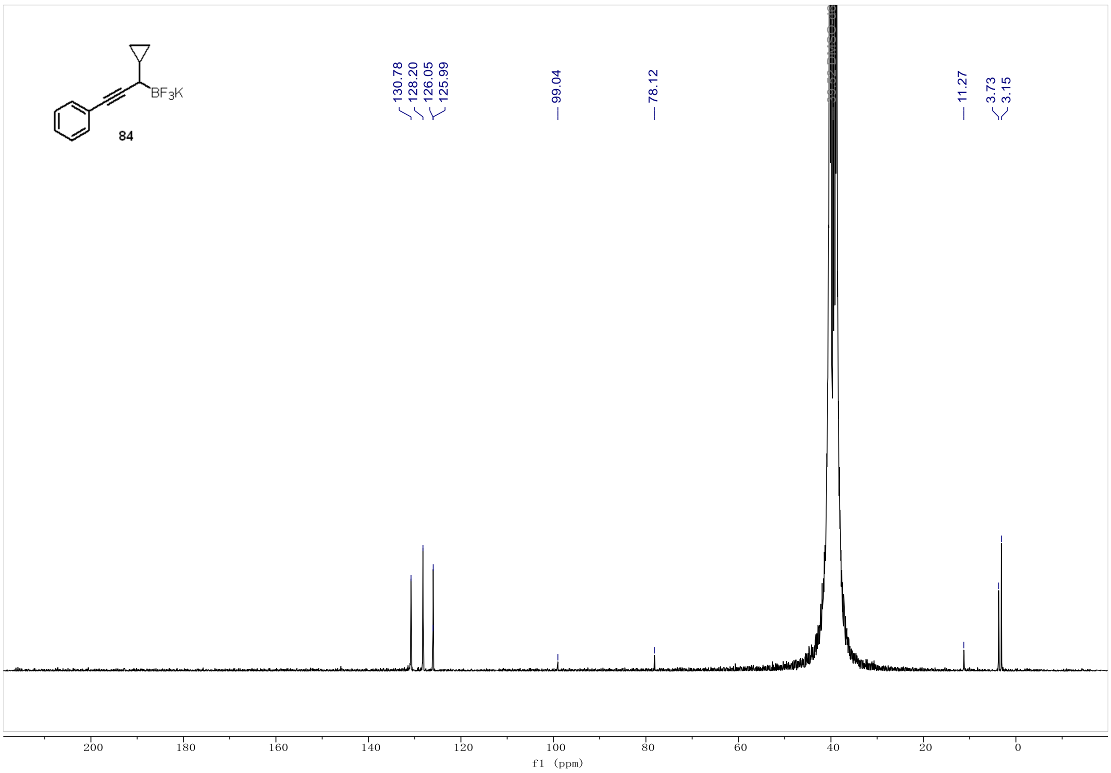

84: ^{11}B NMR (128 MHz, DMSO-d_6)

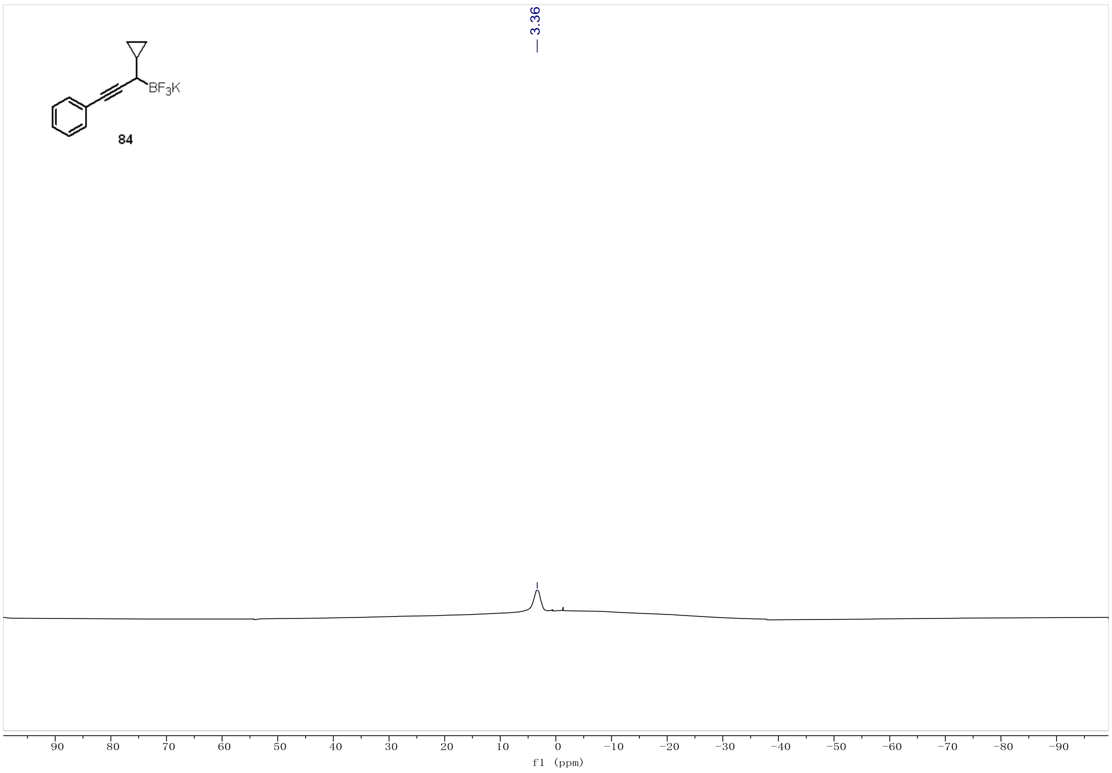

84: ^{19}F NMR (376 MHz, DMSO-d_6)

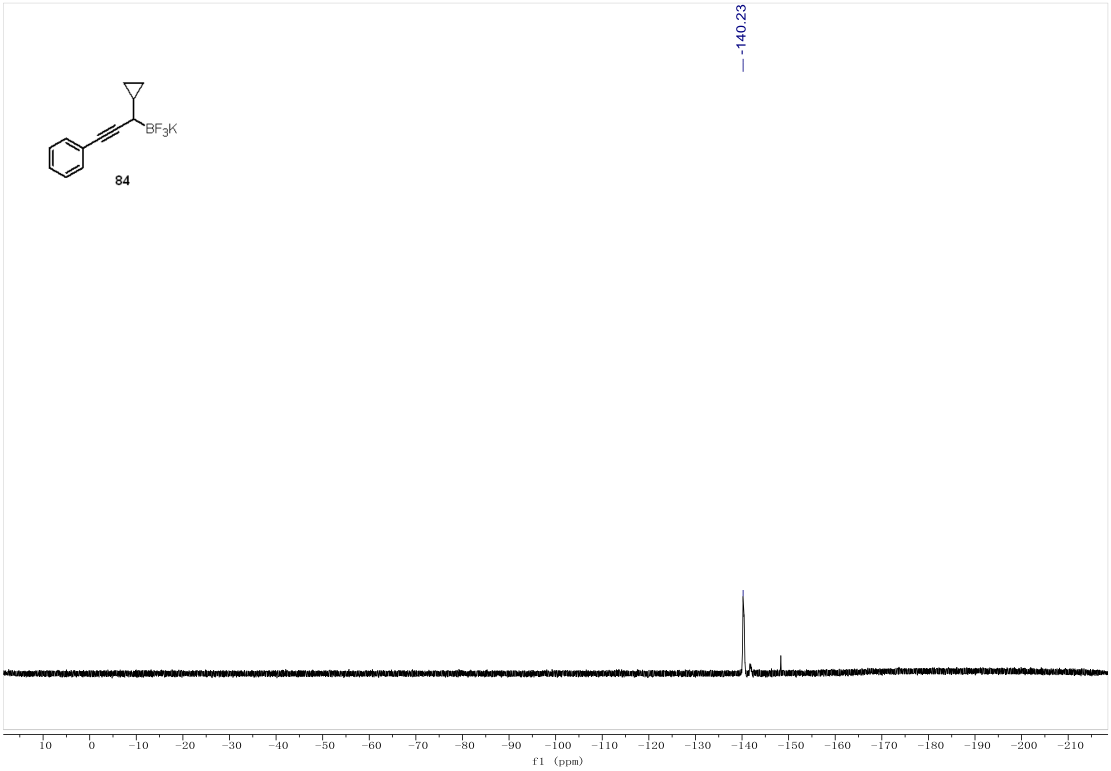

85: ^{1}H NMR (400 MHz, Chloroform-*d*)

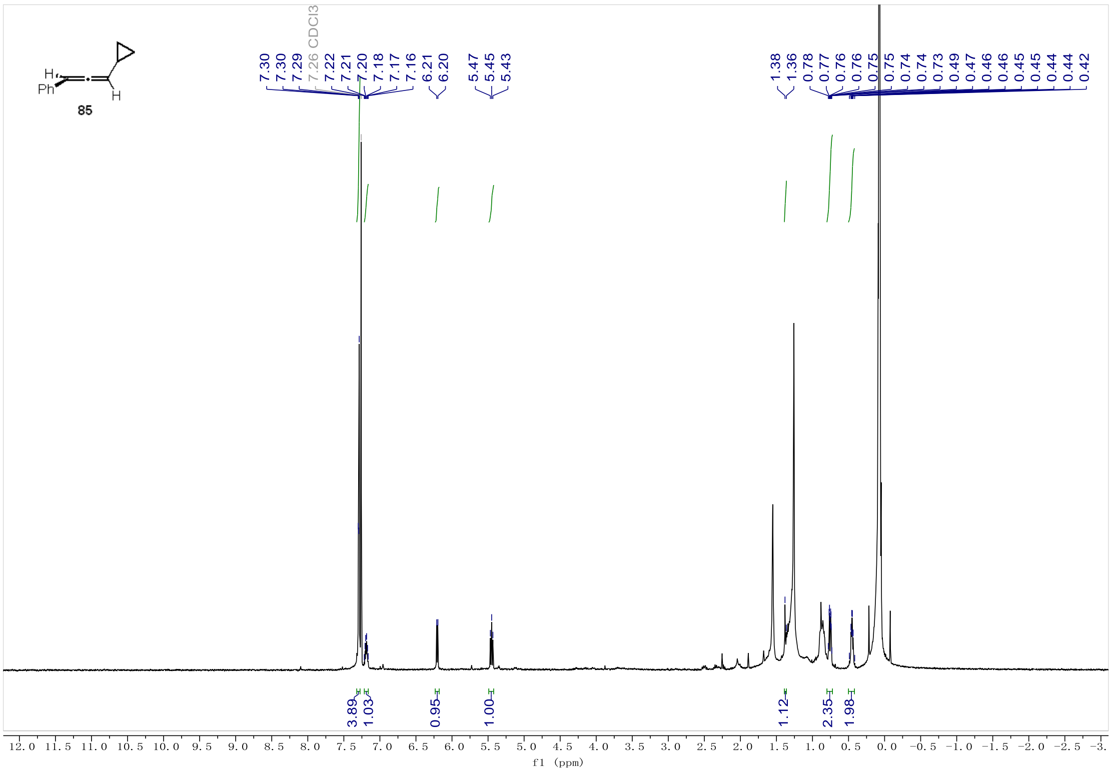

Supplement: Supplementary file 1 — Supporting Information [file ADVS-11-2308710-s001.pdf]
